# Effect of Immune Pressure on Hepatitis C Virus Evolution: Insights From a Single-Source Outbreak

**DOI:** 10.1002/hep.24076

**Published:** 2011-02

**Authors:** Shahzma Merani, Danijela Petrovic, Ian James, Abha Chopra, Don Cooper, Elizabeth Freitas, Andri Rauch, Julia di Iulio, Mina John, Michaela Lucas, Karen Fitzmaurice, Susan McKiernan, Suzanne Norris, Dermot Kelleher, Paul Klenerman, Silvana Gaudieri

**Affiliations:** 1 Centre for Forensic Science, University of Western AustraliaWestern Australia, Australia; 2 Department of Clinical Medicine and Institute of Molecular Medicine, Trinity College DublinDublin, Ireland; 3 Centre for Clinical Immunology and Biomedical Statistics, Institute of Immunology and Infectious Disease, Murdoch UniversityWestern Australia, Australia; 4 University Clinic of Infectious Diseases, University Hospital Bern and University of BernBern, Switzerland; 5 Institute of Microbiology, University Hospital Center, University of LausanneLausanne, Switzerland; 6 Nuffield Department of Clinical Medicine, Oxford UniversityOxford, United Kingdom; 7 Biomedical Research Centre, John Radcliffe HospitalOxford, United Kingdom; 8 School of Anatomy and Human Biology, University of Western AustraliaWestern Australia, Australia

## Abstract

The host's immune response to hepatitis C virus (HCV) can result in the selection of characteristic mutations (adaptations) that enable the virus to escape this response. The ability of the virus to mutate at these sites is dependent on the incoming virus, the fitness cost incurred by the mutation, and the benefit to the virus in escaping the response. Studies examining viral adaptation in chronic HCV infection have shown that these characteristic immune escape mutations can be observed at the population level as human leukocyte antigen (HLA)–specific viral polymorphisms. We examined 63 individuals with chronic HCV infection who were infected from a single HCV genotype 1b source. Our aim was to determine the extent to which the host's immune pressure affects HCV diversity and the ways in which the sequence of the incoming virus, including preexisting escape mutations, can influence subsequent mutations in recipients and infection outcomes. *Conclusion:* HCV sequences from these individuals revealed 29 significant associations between specific HLA types within the new hosts and variations within their viruses, which likely represent new viral adaptations. These associations did not overlap with previously reported adaptations for genotypes 1a and 3a and possibly reflected a combination of constraint due to the incoming virus and genetic distance between the strains. However, these sites accounted for only a portion of the sites in which viral diversity was observed in the new hosts. Furthermore, preexisting viral adaptations in the incoming (source) virus likely influenced the outcomes in the new hosts. (Hepatology 2011;53:396-405)

After infection with hepatitis C virus (HCV), outcomes are variable: spontaneous resolution of the infection is observed in approximately 30% of individuals, but for others, chronic infection develops. Factors such as age, gender, and host genetic variants have been associated with different infection outcomes^1^,^2^ (reviewed by Rauch et al.^3^). Study cohorts that capture all individuals exposed to the virus, such as HCV single-source outbreak cohorts^4^,^5^ and cohorts of individuals who have a high risk of HCV exposure,^6^ have been particularly important in delineating relevant viral and host factors associated with the outcome of HCV infection. Such studies corroborate other studies indicating that a host's T cell response to HCV, including genes involved in regulating this response, is an important correlate of infection outcome.^7-11^

T cell immune responses are stimulated by the presentation of processed viral peptides (epitopes) by human leukocyte antigen (HLA) molecules to CD4^+^ and CD8^+^ T cells. This host-virus interaction is dependent on the sequence of the viral epitope and surrounding regions, which play a role in peptide processing and presentation to T cells. Viral adaptations can reduce the binding affinity of the peptide to the HLA molecule and result in poor peptide cleavage or poor T cell recognition; these factors can subvert host immune control (reviewed by Bowden and Walker^12^). The importance of immune control in HCV infection has been illustrated in studies showing that mutations in CD8^+^ T cell epitopes contribute to viral persistence in both chimpanzees and humans.^13^,^14^ Accordingly, the extent to which the virus can adapt to the host's immune response is likely to be an important factor in determining infection outcome. These adaptations are dependent on the sequence of the incoming virus and the balance between the fitness cost incurred by these mutations^15^ and their benefit to the virus due to immune escape.

It is unclear how much genetic diversity observed in HCV is the result of host immune pressures. Recent studies have suggested that viral adaptation can be observed at both the individual level^16^,^17^ and the population level.^18^,^19^ For example, genetic studies examining HCV sequences in the context of the HLA repertoire of a host population have shown associations between specific polymorphisms across the viral genome and HLA types within individuals in a host population.^18^,^19^ These HLA-associated viral polymorphisms are thought to represent viral adaptations and tag regions of the viral genome that are under *in vivo* T cell pressure. However, HCV evolution is shaped by evolutionary forces that include genetic drift and both positive and purifying selection pressures.^20^,^21^ It is likely that all these factors exert their influence simultaneously on the virus and affect the ability of the virus to adapt to new selection pressures and/or revert in a new host.

A previous study of an Irish HCV single-source cohort showed evidence of immune selection in known T cell targets.^22^ In this study, we compared HCV sequences from 63 individuals with genotype 1b infection from this single-source outbreak^5^ to identify sites likely representing new T cell targets in the HCV genome and to determine the extent to which host immune pressures on the virus affected sequence diversity in the cohort. Knowledge of the incoming viral sequence also allowed us to determine whether preexisting viral adaptations could predict beneficial or detrimental host HLA alleles within the cohort with respect to infection outcomes.

## Patients and Methods

### Study Population

The study population was part of a cohort of women who had been infected with HCV between May 1977 and November 1978 in Ireland through the administration of anti-D immunoglobulin that had been contaminated with an HCV genotype 1b virus originating from a single individual.^5^ From this original cohort, we studied 63 individuals with chronic HCV infection; a subset (n = 15) was selected on the basis of the carriage of HLA-A*03, an allele that was previously shown to be protective in this cohort.^8^ A comparison of the HLA alleles found in this cohort and those in another Irish population is in the Supporting Information.

Serum samples from the subjects were collected between 1996 and 2002 and were stored at −80°C. Written, informed consent was obtained from participants, and local institutional review board approval was obtained by all centers contributing to the study.

#### Viral RNA Extraction

Viral RNA was extracted from serum samples with the QIAamp Viral RNA mini kit (Qiagen) or the Cobas Amplicor HCV specimen preparation kit (version 2.0, Roche) according to each manufacturer's instructions.

#### HLA Genotyping

Two-digit resolution HLA class I (HLA-A, HLA-B, and HLA-C) typing was performed at St. James Hospital (Dublin, Ireland).^8^

#### Interleukin-28B (*IL-28B*) Genotyping

Genotyping of the single-nucleotide polymorphism (SNP) rs12979860 upstream of the *IL-28B* gene was performed for 34 subjects as previously described.^23^

#### Bulk Viral Sequencing

HCV sequencing was performed as previously described.^18^,^19^ Briefly, three separate reverse-transcription PCRs were performed which overlapped to cover the core to nonstructural (NS) 5B region. The first-round products were used as templates in nested second-round polymerase chain reactions containing generic or genotype-specific primers. Amplicons were bulk-sequenced with the BigDye Terminator version 3.1 cycle sequencing kit (Applied Biosystems) according to the manufacturer's recommendations, and electropherograms were edited with Assign (Conexio Genomics). Mixtures were identified in which the secondary peak was greater than 20% of the main peak.

HCV sequences in this study have been submitted to GenBank (accession numbers HM106522 to HM106981). Supporting Information Table 1 lists the mean sequence coverage by protein.

An analysis of the viral sequences for testing the single-source nature of this outbreak can be found in the Supporting Information.

#### Ultradeep Sequencing

To identify minor quasispecies below the detection threshold of bulk sequencing methods, ultradeep sequencing was carried out with the 454 Life Sciences platform (Roche Applied Science) for two individuals (HLA-A*03^+^/HLA-B*08^−^ and HLA-A*03^−^/HLA-B*08^−^). With the previously described amplification method, polymerase chain reaction templates were obtained that covered NS3 (positions 3494-4530) and NS5A to NS5B (positions 7335-8356). Amplicons were quantified and pooled for each individual. Library preparation and sequencing were performed according to the manufacturer's protocol. Data were collected and analyzed with Roche and public license software programs. All sequence reads were aligned to the source sequence (AF313916) with GS Reference Mapper software (Roche). The threshold for mixtures was set at 1% with 100-fold or greater coverage.

#### HLA-Associated Viral Polymorphisms

Associations between HLA alleles and amino acid distributions at each residue of the HCV proteins were assessed with Fisher's exact test for classification as consensus or nonconsensus amino acid. A false discovery rate analysis was carried out, and *q* values were obtained as reported previously.^19^ Only sequences with ≥50% sequence coverage for each respective protein were used. Analyses were carried out with Spotfire S+ 8.1 (TIBCO, Somerville, MA). Associations with a *P* value ≤0.01 for Fisher's exact test of consensus versus nonconsensus are reported. An assessment of possible confounding by founder effects via viral cluster stratification and the Mantel-Haenszel procedure, as described by Rauch et al.,^19^ indicated that no correction for significant associations was necessary, and this was consistent with the sequences originating from a single source. In addition, because *P* values associated with relatively small frequencies can be affected by small numbers of misclassified cases, we restricted our analysis to associations for which there were five or more nonconsensus amino acids and five or more carriers of the HLA allele.

#### Sliding-Window Analysis

In order to identify viral escape that might not be captured with a single amino acid approach, an analysis was conducted as described previously, except that *adaptation* was defined as nonconsensus at any residue within sliding windows of nine amino acids, which represented typical peptide sizes for HLA class I molecules. Significant sites of associations were identified as strings of significant values, whereas the window slid over any residues containing strong associations or combinations of associations. We restricted the analysis to cases that had all amino acids in the window. Associations with *P* ≤ 0.01 were reported.

#### Covariation

Residue covariation was assessed with Fisher's exact test for classification as consensus or nonconsensus amino acid. Covariation based on a sequence with ≥90% coverage was reported; covarying sites had *P* ≤ 0.001 for amino acid versus amino acid comparison and *P* ≤ 0.0001 for amino acid versus nucleotide comparison. Because of the exploratory nature of this part of the analysis, no adjustment was made for multiple comparisons.

#### Peptide Prediction for HLA-Associated Viral Polymorphism Sites

Flanking sequences of the identified HLA-associated viral polymorphisms and sites of common divergence from the source sequence were entered into the epitope prediction software SYFPEITHI^24^ to identify putative epitopes based on a cutoff score of 20 with the highest scoring peptide reported. HLA-associated viral polymorphism sites were compared against published genotype 1 epitopes found in the Immune Epitope Database (http://www.immuneepitope.org).

#### Viral Sequence Diversity

Sequence diversity from the source sequence (AF313916) was determined with the Highlighter program (available at http://www.lanl.gov) for NS3 and NS5B to identify sites of synonymous and nonsynonymous substitutions for sequences with greater than 50% sequence coverage. Genetic diversity was determined with the Kimura two-parameter model, and differences in the rate of nonsynonymous and synonymous changes (ds/dn) were obtained with the modified Nei and Gojobori method with MEGA version 3.1.^25^

#### *IL-28B*–Associated Viral Polymorphisms

We assessed associations between the presence or absence of the minor allele rs12979860 and consensus or nonconsensus amino acids at each residue of the HCV proteins via Fisher's exact test. Because of the smaller number of subjects with typing available for this part of the analysis, no assessment of false discovery rates was made, and *P* ≤ 0.01 was used to indicate significance.

## Results

### HLA-Associated Viral Polymorphisms: Putative Viral Adaptations in the New Hosts Reflecting Sites of Immune Pressure

We determined whether there were associations between the expression of particular HLA alleles in subjects in this cohort and specific polymorphisms in their viral sequences (putative viral adaptations) reflecting areas under *in vivo* T cell immune pressure. We identified 29 HLA-associated viral polymorphisms with *P* ≤ 0.01 for 23 sites along the HCV genome (Table 1 and Supporting Information Fig. 3). In some instances, HLA alleles from different loci were associated with the same site, and we have previously shown that these associations can be explained in part by the linkage disequilibrium observed within the major histocompatibility complex (MHC).^18^ Among those associations shown in Table 1, three HLA-B/C combinations are associated with common MHC haplotypes. The *q* values for associations within some of the proteins are high with respect to others (particularly E2) and possibly reflect smaller sample sizes in these proteins (Supporting Information Table 1).

**Table 1:** HLA Class I-Associated Viral Polymorphisms

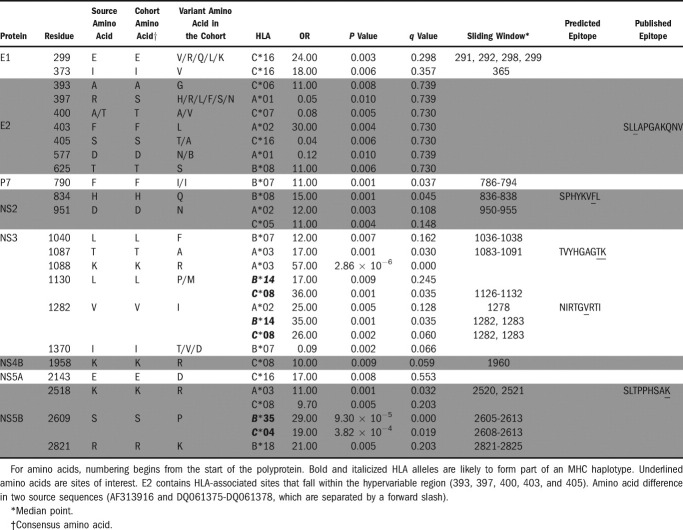

Two HLA-associated viral polymorphisms fell within previously published epitopes (HLA-A*02 epitope in E2 404 SLLAPGAKQNV and HLA-A*03 epitope in NS5B 2518 SLTPPHSAK; Table 1). Furthermore, three HLA-associated viral polymorphisms fell within predicted epitopes as determined by the peptide binding prediction program SYFPEITHI^24^ (Table 1). The limited number of matches between known epitopes and putative viral adaptation sites may be the result of the small number of published HCV epitopes in the literature and its focus on common HLA types. Several of the putative viral adaptations are associated with HLA-C alleles for which there are either no or few known HLA-restricted epitopes or characterized binding properties.

None of the associations shown in Table 1 overlap with the findings of our previous studies examining HLA-associated viral polymorphisms for genotype 1.^18^,^19^ However, the previous study had a much larger number of genotype 1a sequences in the data set than 1b sequences; because the sequences in this single-source cohort were all genotype 1b, it was likely that we would observe differential escape profiles similar to what we had seen between genotypes 1a and 3a but to a lesser extent between genotype 1 subtypes (1a and 1b). Furthermore, in contrast to the subjects in the previous cross-sectional studies, the subjects in this study were infected from a single-source strain.

#### Window Analysis Identifies Additional Areas Under T Cell Pressure

Areas under HLA-specific immune pressure that can accommodate more than one site of variation may not be detected by our initial single amino acid approach. Accordingly, a sliding-window analysis (with a size reflective of a typical HLA class I epitope) was also performed to examine areas under HLA-specific immune pressure in which more than one site might be relevant for escape. As expected, several of the HLA-associated viral polymorphisms identified with a single-site analysis were identified with the window analysis (Table 1). However, the single-site associations found in highly variable regions in E2 were not identified in the window analysis, probably because of the higher level of variation found in this region in comparison with other proteins that may occur in some cases when the variation is not related to adaptation (as tested here) and may hinder the ability to find specific HLA associations with any change(s) within a window. There were three examples [E2 and HLA-C*06 with a median position of 537, odds radio (OR) = 28; NS2 and HLA-B*08 within windows of 875-878, OR = 0.026-0.039; and NS5A and HLA-B*08 with a median position of 2132, OR = 26] for which the window analysis identified HLA-associated substitutions that were not found to be significant in the single-site analysis. These cases suggested that multiple sites within a target region may be under immune pressure (Supporting Information Fig. 4). This observation is consistent with our own study and other studies showing different escape profiles within epitopes, including the immunodominant HLA-B*08 epitope (1395-1403) in NS3^17^ and the protective HLA-B*27 epitope (2841-2849) in NS5B.^11^

Overall, the number of associations found with either the single-site analysis or the sliding-window analysis represented only a portion of the 184 variable sites across the viral genome that fit the inclusion criteria described in the methods (18 of 163 if the highly variable region in E2 is excluded because this area is likely to also be under other strong selective pressures).

#### Source and Causes of Viral Adaptation

**Fig. 1:**
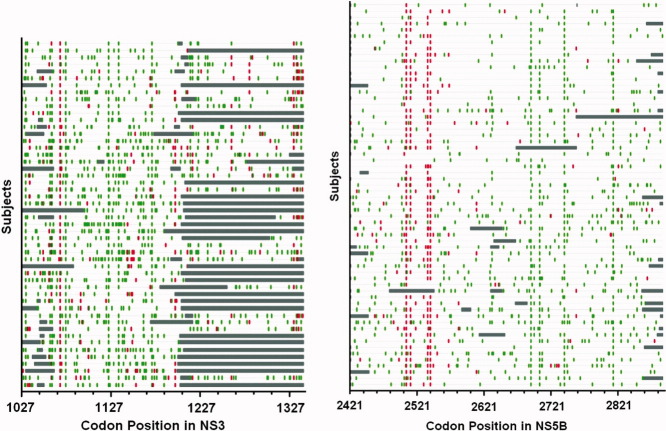
Highlighter plot of synonymous and nonsynonymous substitutions in NS3 and NS5B with respect to the source sequence (AF313916). The plot was created with Highlighter (available at http://www.lanl.gov). Red lines denote nonsynonymous substitutions, green lines indicate synonymous substitutions, and gray regions show unsequenced sections.

We then examined the pattern of synonymous and nonsynonymous changes in these sequences to determine if purifying selection was acting across the HCV genome and potentially restricting the ability of the virus to adapt to new selection pressures or revert to unadapted forms. Figure 1 shows the pattern of these changes in each individual with respect to the source within the NS3 and NS5B proteins. It is apparent that there are a greater number of synonymous changes with respect to nonsynonymous changes in this region (indicating purifying or negative selection; dS-dN for NS3 = 0.080 and for NS5B = 0.061). Similar results were observed for other proteins (data not shown).

#### Covarying Sites in the Genome Likely to Reflect Networks Within the HCV Genome

As previously suggested, purifying selection may reflect the existence of covarying sites in the HCV genome.^26^ Here we identified sites of covariance by assessing amino acid sites in a pairwise manner per protein and genome-wide for sequences with greater than 90% sequence coverage. Only results with *P* < 0.001 were reported because adjustments for multiple comparisons were not made in this analysis. Thirteen of 25 paired sites of significant covariance were within the same protein, whereas 12 of 25 fell in different proteins. For the majority of pairs of covariant sites, one or both sites fell at a reported HLA-associated viral polymorphism site, within a known epitope, or at a common site of reversion from the source. Four of the 25 paired sites fell at an HLA-associated site in Table 1. In particular, two HLA-A*03–associated sites at positions 1087 and 1088 in NS3 fell within a confirmed HLA-A*03 epitope in which variation at both sites is required to restore replicative efficacy (K.F., unpublished data, 2010); this reflects the potential compensatory nature of these covariations.

**Fig. 2:**
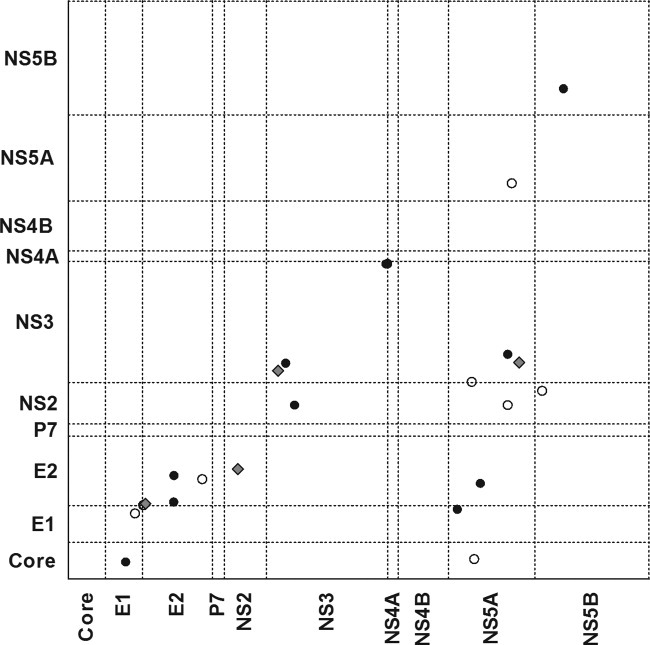
Covarying sites (*P* < 0.001) in the HCV genome represented as coordinates. Open diamonds indicate that one or both sites fall within an epitope or at an association site, and dark diamonds indicate that the sites do not fall within either. Many covariant sites fall in close proximity to one another in the genome (illustrated by the linear trend); however, there are groupings that suggest strong covariation between residues within NS5A and residues within other proteins. Sequence coverage was not found to be a function of covariant site identification.

Fig. 2 shows a linear trend for many covarying sites suggesting that many fell in close proximity to one another but not necessarily in the same protein. Interestingly, clusters of covarying sites appeared to connect sites across the genome and particularly other proteins with NS5A. One group contained sites in only one protein (NS3 sites 1644F/Y, 1647A/T, and 1656A/T), whereas another group contained sites in three proteins (NS2 908R/K, NS3 1173S/L, and NS5A 2279R/K). These links may further restrict the ability of the virus to adapt or revert quickly and suggest critical interactions between the HCV proteins. We extended this analysis to assess covariation at amino acid and synonymous sites to identify potential constraints on codon usage (and subsequent amino acid changes) and identified four amino acid sites associated with synonymous changes in other proteins.

#### Relevance of Viral Adaptations in the New Hosts and Preexisting Ones in the Source in Infection Outcomes

**Fig. 3:**
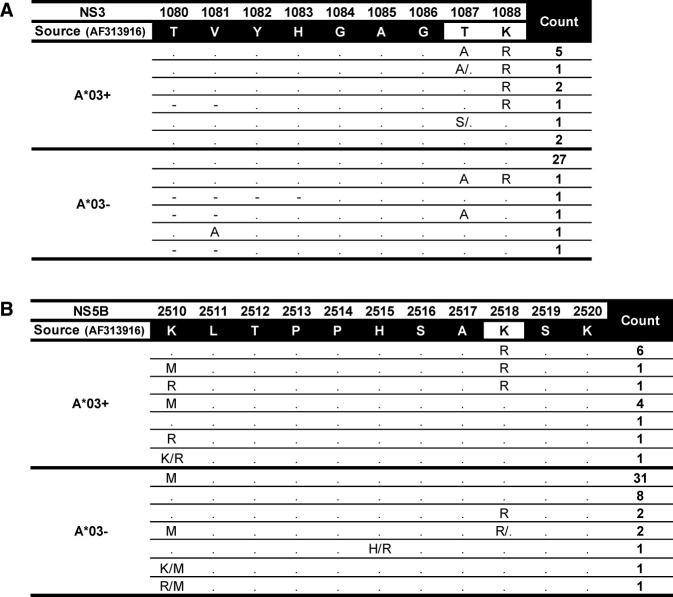
HLA-A*03–associated viral polymorphisms at (A) positions 1087 and 1088 in NS3 and (B) position 2518 in NS5B. Sequences in regions of interest (from Table 1) are displayed for HLA-A*03^+^ and HLA-A*03^−^ subjects. The sequence identity with the source sequence is identified by a dot. Amino acid mixtures at a site are separated by a forward slash. The number of individuals with a particular sequence is shown in the count column. The lysine (K) to arginine (R) substitution at 2518 (8 of 15 HLA-A*03^+^ subjects versus 4 of 47 HLA-A*03^−^ subjects) resulted in a change in the SYFPEITHI-predicted binding score from 27 to 21. Only one HLA-A*03 individual with chronic infection did not have a polymorphism at the 1087 or 1088 site in NS3 or at the 2518 site in NS5B.

Although the host immune pressure is one of several forces shaping HCV diversity, it is likely that only a small number of selected viral adaptations in the sequence may affect infection outcomes. In this cohort, HLA-A*03 was shown to be protective,^8^ and we selected chronic HCV–infected individuals with HLA-A*03 for this study to identify viral adaptations in these individuals that may have affected their infection outcomes. Three viral polymorphisms were associated with HLA-A*03 in this study (Table 1). Two of the associations were in NS3 at positions 1087 and 1088 within a predicted epitope for HLA-A*03. As mentioned previously, this epitope was subsequently shown to be a true *in vivo* target of the immune response (NS3 1080 TVYHGAGTK; K.F., unpublished data, 2010; Fig. 3A) and reflected a drop in the SYFPEITHI-predicted binding score from 34 for the wild type to 21 for the putative escape peptide. Another HLA-A*03–associated viral polymorphism at position 2518 in NS5B was within the previously characterized genotype 1a epitope SLTPPHSAK (Fig. 3B). Half of the HLA-A*03 individuals had a polymorphism at these sites in both regions. These results suggest that these two viral epitopes are important immune targets and that escape within the targets may influence the outcome.

Further analysis of the quasispecies at the NS3 1087 and 1088 sites in HLA-A*03^+^ and HLA-A*03^−^ subjects was performed with ultradeep sequencing. Table 2 reveals the lack of a source sequence at amino acid position NS3 1088 in the HLA-A*03 subject with complete amino acid replacement but 100% retention of the source sequence in the HLA-A*03^−^ subject. The two subjects had the same amino acid at position 1087 (unadapted), but codon usage was different between the two.

**Table 2:** Ultradeep Sequencing Reveals a Lack of a Source Sequence at Putative Viral Adaptation Sites (NS3 1087 and 1088) in a Subject With HLA-A3 but 100% Maintenance of the Source Sequence in an HLA-A3^−^ Subject

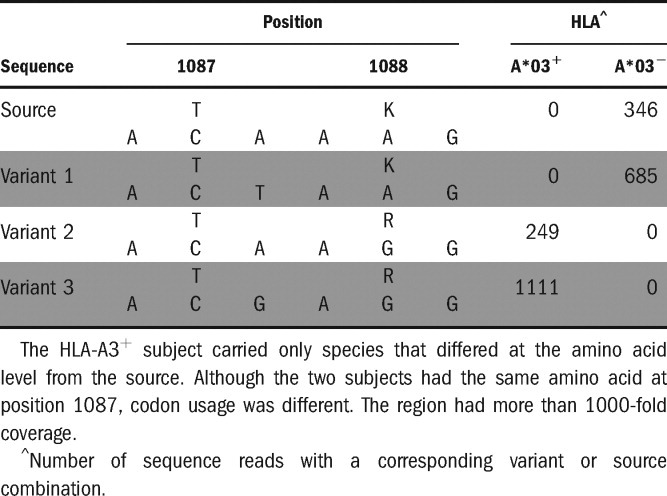

Previous studies have found other HLA alleles to be associated with chronic infection that are specific to this cohort, such as alleles HLA-A*01, HLA-B*08, and HLA-C*07^8^ (these alleles most likely correspond to a single MHC haplotype). It has been suggested that the association between infection outcomes and specific HLA alleles may be due to preexisting viral adaptations in the incoming virus that may facilitate the evasion of host immune responses with the corresponding HLA types.^27^ Here we tested this hypothesis by examining the source sequence for escape mutations within known epitopes as well as putative viral adaptations identified in our previous genetic study of chronic HCV infection.^18^,^19^

Initially, we examined the immunodominant epitope for HLA-B*08 in NS3 (1395 HSKKKCDEL) and the protective HLA-B*27 epitope in NS5B (2841 ARMILMTHF). The region in the source containing the HLA-B*27 epitope in NS5B had the unmutated form. However, the HLA-B*08 epitope in NS3 in the source sequence had a preexisting viral adaptation in the epitope (arginine at position 3), which subsequently reverted in 8 of 11 subjects without HLA-B*08 and was retained in 5 of 8 subjects who expressed HLA-B*08. Although the numbers in the two groups were not significantly different (*P* = 0.18), they supported other studies showing reversion from an arginine to lysine at position 3 in this epitope when there was no immune pressure; this is suggestive of a fitness cost.^15^ This HLA-B*08 epitope was previously studied in this cohort with similar results.^15^,^22^ The fitness cost of this substitution was further supported by the results from the ultradeep sequencing of two HLA-B*08^−^ subjects in this region, who showed complete reversion from the source escape mutation at position 3 of the epitope (Table 3).

**Table 3:** Ultradeep Sequencing Reveals a Lack of a Source Sequence at Position 1397 in the Immunodominant HLA-B*08 Epitope in NS3 (HSKKKCDEL) in Two HLA-B*08^−^ Subjects

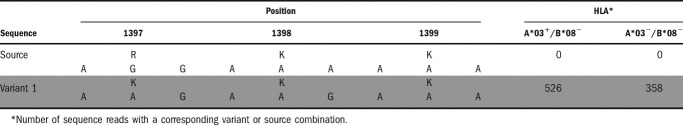

Viral adaptation in the source sequence at a site in the HLA-B*08 immunodominant epitope likely to incur a fitness cost suggests that the source may have been an HLA-B*08^+^ individual. We suggest that this could potentially reduce the ability of hosts with HLA-B*08 to control the virus via the reduction of good immune targets, and this reflects the association of this allele with poor outcomes in this cohort. Additional association sites with HLA-B*08^+^ individuals found in this study may represent alternative targets for HLA-B*08 along the HCV genome. Furthermore, Table 1 and Supporting Information Fig. 4 list HLA-associated viral polymorphisms that have an OR less than 1 and represent the maintenance of the consensus sequence (which for most sites in Table 1 is the same as the source) for the specific HLA type; this possibly reflects that the source sequence is pre-adapted at these sites. Interestingly, this occurs for alleles within the MHC haplotypes HLA-A*01, HLA-B*08, and HLA-C*07, which are associated with poor outcomes.

#### Other Selective Pressures Likely to Affect HCV Evolution

In order to determine how other host immune pressures may affect HCV evolution, we assessed possible associations between HCV polymorphisms in this cohort and an SNP that tags the *IL-28B* gene encoding interferon-λ3 and recently has been associated with infection outcome.^2^ We found one significant association between homozygosity for the major allele of rs12979860 (associated with good outcome) and variation at position 849 in NS2 (*P* = 0.006). We also tested for additional effects of the *IL-28B* SNP on the HLA-associated polymorphisms. After adjustments for HLA, among the positions identified in Table 1, *IL-28B* was associated with a polymorphism (*P* = 0.036) only at position 2609 of NS5B, which harbors the strong HLA-B*35/HLA-C*04 association. The significance of the HLA-B*35 association with nonconsensus after adjustments for the *IL-28B* SNP is *P* = 0.00004, whereas for HLA-B*35 alone, the *P* value is 0.0001. There was no significant interaction between the effects of HLA-B*35 and *IL-28B* (*P* > 0.9), and this suggests that they act independently. Further studies examining the association between variations that tag *IL-28B* and HCV evolution are warranted and should be performed on larger cohorts including subjects with different treatment and infection outcomes.

## Discussion

Here we illustrate that the incoming viral sequence, host immune pressure, and covariation play important roles in shaping HCV viral diversity. Specifically, we identified 29 significant HLA-associated viral polymorphisms (*P* ≤ 0.01; 23 sites) within the cohort that likely reflect viral adaptations. Some of these sites fall within published and/or predicted T cell epitopes. The use of a sliding-window analysis accounting for more than a single escape variant within a T cell target identified a small number of additional potential regions under T cell pressure, and this supported other studies showing that escape can require the accumulation of escape mutations^28^ or that viral escape sites are often mutually exclusive because of the fitness cost.^15^,^18^

The number of significant HLA-associated viral polymorphism sites identified in this study is only a small proportion of the sites (23/184) across the HCV genome showing variation in the cohort; this is possibly due to the relatively small sample size or suggests that the host immune pressure has a targeted influence on HCV diversity. This would be expected because the immune system sees the viral polyprotein as a set of peptides, and only a small number of these peptides are likely to be presented to the immune system. Furthermore, the lack of significant overlap with previously reported adaptations for genotypes 1a and 3a likely reflects the constraint of the incoming virus and differential viral adaptation pathways on genotype 1b versus other circulating genotypes due to the genetic distance between these strains. It should be noted that although we did not show HLA class II–associated viral polymorphisms, it is likely that, in addition to what we observed for HLA class I alleles, some of the variations correspond to the expression of specific HLA class II alleles.

To appreciate the extent to which both positive and purifying selections influence HCV diversity, we examined the number of synonymous and nonsynonymous changes across the genome for this single-source cohort. An abundance of synonymous changes indicated purifying selection that would to some extent limit the plasticity of HCV. Covariations that become fixed across the HCV genome may also restrict the ability of HCV to adapt to the host's immune response and revert when it enters a new non–HLA-matched host. We examined the genome for covarying sites and showed that although covariation did occur locally within proteins, there were also a number of sites that were linked to sites more distant in the genome. Furthermore, several of these sites were putative viral adaptation sites.

Access to the source viral sequence from this single-source cohort allowed the identification of preexisting escape mutations across the genome. A known escape mutation at position 3 of the immunodominant HLA-B*08 NS3 epitope was found in the source sequence. This mutation was for the most part retained in HLA-B*08 subjects but had reverted in most HLA-B*08^−^ subjects. Furthermore, deep sequencing revealed no traces of the escape mutant in two B*08^−^ individuals, and this supports the fitness cost that may be incurred by the escape mutation. Importantly, existing adaptation in the incoming virus may affect infection outcomes in individuals expressing the appropriate HLA type. The pre-adaptation of the source sequence to HLA-B*08 may account for the observed lack of protection of HLA-B*08 in this cohort.

The single-source cohort studied here has provided us an opportunity to obtain a better understanding of viral diversity and the ways in which different forces can shape viral diversity at the population level.

## Abbreviations

HCV: hepatitis C virus
HLA: human leukocyte antigen
IL-28B: interleukin-28B
MHC: major histocompatibility complex
NS: nonstructural
OR: odds ratio
SNP: single-nucleotide polymorphism
