# Supplementary material for: Effect of Immune Pressure on Hepatitis C Virus Evolution: Insights From a Single-Source Outbreak
Source: Hepatology. 2011 Feb;53(2):396–405. doi: 10.1002/hep.24076 (PMC3044208; doi:10.1002/hep.24076)
Supplement: Supplementary file 1 [file hep0053-0396-SD1.doc]

**Comparison of HLA frequencies in study cohort to general population**

Supporting Figure 1 shows that the HLA Class I allele frequency distribution in this cohort was similar to that found in the general Irish population (1) with a few exceptions (HLA-B*60, -C*03, -C*09, -C*10). As expected, the frequency of HLA-A*03 is higher in the HCV single source cohort, but this difference was not significant (p=0.09). The HLA-B*27 allele that has previously been associated with HCV infection clearance (2) is found at a lower frequency in this cohort whereas the HLA-B*08 allele associated with persistence in this cohort in other studies (3) has a higher allele frequency when compared to the general Irish population, however these differences were not significant.


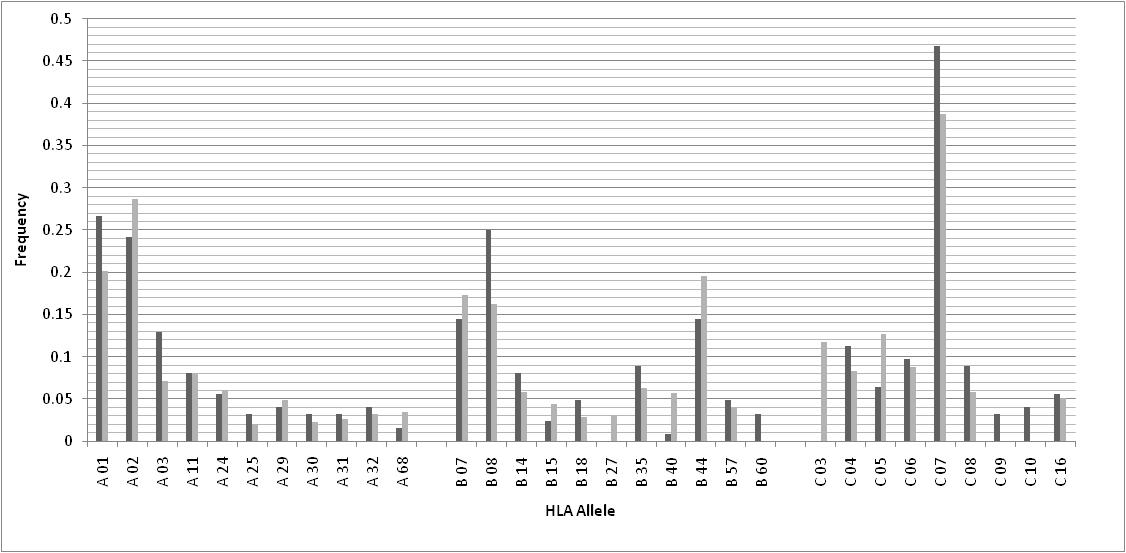


**Supporting Figure 1. HLA allele frequencies for the general Irish population and the Irish HCV single source cohort were similar with few exceptions.** Dark bars indicate general Irish population and grey bars the Irish HCV single source cohort. Northern Ireland n=2000; Irish single source cohort n=63. Significant difference in HLA allele frequency (p<0.05; Chi-squared test) between the two populations was found for HLA-B*60, -C*03, -C*09 and -C*10. Only HLA alleles present in at least 5 individuals were included.

## Confirmation of single source cohort

## Phylogenetic analysis

## A phylogenetic tree of NS3 was generated using the Neighbor-Joining method based on the p-distance model with pair-wise deletion and 1000 bootstrap replications. Sequences in this analysis included other HCV sequences generated in our laboratory (4), from the Los Alamos National Laboratory (LANL) public database (5) and previously published sequences from the Irish single source cohort (Accession numbers AB154177-AB154206 and DQ061331-DQ061378). Sequences with ≥90% sequence coverage from positions 1 to 1875 were included. All analyses were performed using MEGA v3.1 (6).

*Monophyletic cluster containing HCV sequences from subjects and source consistent with single source outbreak of a HCV genotype 1b strain*

We initially examined whether the HCV sequences generated from the subjects are consistent with a single source outbreak of a HCV genotype 1b strain as determined by others (3, 7, 8). HCV sequences from subjects were compared to HCV sequences in the public database (5) (including from our own laboratory (4)) and from subjects in this single source cohort obtained by other groups (7, 8). Supporting Figure 2 shows the monophyletic cluster corresponding to the HCV sequences obtained from the subjects in this study, the source and other HCV sequences from the same cohort. This cluster is distinct from other HCV sequences obtained from our laboratory and in the public database (cluster supported by strong bootstrap value 90%).

**Supporting Figure 2. Phylogenetic analysis of NS3 HCV genotype 1b sequences reveals a strongly supported monophyletic cluster containing sequences from subjects in the single source cohort and the source.** Sequences from the LANL database are unmarked and those previously published by our group (4) are shown as grey circles. Open circles represent single source cohort sequences obtained from this study and open triangles and squares indicate sequences obtained by others from the same cohort published by Ray et al and Ikatura et al respectively (7, 8). Dark circles refer to the cohort source sequences (DQ061375-DQ061378 and AF313916).

###### Estimate of time of transmission

###### To further validate the sequences in this study we used an established Bayesian Markov Chain Monte Carlo (MCMC) approach as implemented in the program BEAST v1.5 (9) to determine the age of the single source cohort cluster based on the generated HCV sequences. We used a previously reported substitution rate of 4.1 x10^-4^ (10) for the NS5B region that was encoded as a prior probability distribution and a constant rate molecular clock under the HKY model of nucleotide substitution. The analysis was performed using 10,000,000 chains with sampling every 1,000 chains. The results were visualized using Tracer v1.4 (11) and FigTree v1.1.2 (12). The analysis suggests the time of transmission to be between 15 and 20 years ago; consistent with the sampling time of these subjects and the time frame of the original infection.

**HLA-associated viral polymorphisms within the HCV genome: putative viral adaptations in the new hosts reflecting sites of immune pressure**

HLA-associated viral sites with p≤0.01 as identified in Table 1 are illustrated with cohort viral sequences in Supporting Figure 3.

**Supporting Figure 3. HLA-associated viral polymorphisms.** Sequences in regions of interest (from Table 1) are displayed for HLA positive and negative subjects (for relevant HLA allele). Grey amino acid residue denotes association site. Sequence identity with the source sequence identified by dot. Amino acid mixtures denoted at “X”. Number of individuals with a particular sequence is shown in the count column.

*Window analysis identifies additional areas under T-cell pressure*

**Supporting Figure 4. HLA-associated viral polymorphisms identified by sliding window analysis.** Sequence identity with the source sequence identified by a dot. Amino acid mixtures separated by a forward slash. Number of individuals with a particular sequence is listed in the count column. (A) E2 and HLA-C*06 with median position 537 of sliding window; (B) NS2 and HLA-B*08, sliding window at 875-878 and (C) NS5A and HLA-B*08, sliding window at 2132. Consensus sequence used based on our previously published sequences, except in A where LANL genotype 1b consensus was used.

**Sequence coverage**

Supporting Table 1. Nucleotide sequence coverage per subject per protein

|  |  |  |  |  |  |  |  |  |  |  |
| --- | --- | --- | --- | --- | --- | --- | --- | --- | --- | --- |
| **Sample ID** | **% sequence coverage per protein (%ambiguous nucleotide "N" in brackets)** | | | | | | | | | |
|  | **Core** | **E1** | **E2** | **P** | **NS2** | **NS3** | **NS4A** | **NS4B** | **NS5A** | **NS5B** |
| **1524** | 100 | 100 | 100 | 100 | 100 | 96.8 | 100 | 100 | 100 | 100 |
| **1525** | 100 | 100 | 100 | 100 | 100 | 82.3 | 100 | 83.6 | 90.4 | 93.7 (0.2) |
| **1526** |  |  |  | 85.7 | 100 |  |  | 89.0 | 84.4 | 98.9 |
| **1527** | 100 | 100 |  | 75.7 | 100 | 94.9 | 100 | 100 | 97.2 | 100 |
| **1528** | 100 |  | 65.3 | 100 | 100 | 100 | 100 | 96.7 | 100 | 100 |
| **1529** | 100 | 100 | 100 |  |  | 51.2 |  | 89.5 | 73.4 | 99.5 (0.2) |
| **1530** | 100 | 100 | 94.0 | 100 | 100 | 100 | 100 | 100 | 100 | 100 |
| **1531** |  |  |  | 94.2 | 100 | 56.6 |  | 98.0 | 87.7 | 90.2 |
| **1532** | 100 |  |  | 100 | 100 | 100 | 100 | 95.8 | 100 | 92.7 |
| **1533** | 100 | 100 | 100 | 100 | 99.5(0.5) | 100 | 100 | 100 | 100 | 100 |
| **1534** | 100 | 100 | 100 | 94.7 | 100 | 54.4 |  |  | 83.4 | 100 |
| **1535** | 100 | 100 | 100 | 100 | 100 | 95.1 | 100 |  | 72.6 | 99.6 |
| **1536** | 100 | 100 | 100 | 100 | 100 | 100 | 100 | 100 | 86.2 | 99.8 (0.2) |
| **1537** | 100 | 100 | 100 | 100 | 100 | 100 | 100 | 100 | 100 | 90.4 |
| **1538** | 100 |  |  | 100 | 100 | 97.6 | 100 | 100 | 78.4 | 95.9 (0.2) |
| **1539** | 100 | 100 | 100 | 100 | 100 | 100 | 100 | 98.0 | 98.4 | 100 |
| **1540** |  |  |  |  |  | 81.4 |  | 64.0 | 100 | 100 |
| **1541** |  |  |  |  | 100 |  |  |  | 75.4 | 51.5 |
| **1542** |  |  |  |  |  |  |  |  | 80.4 | 80.4 |
| **1543** |  |  | 74.9 | 94.7 | 100 | 55.7 |  | 80.7 | 98.8 | 100 |
| **1544** | 100 | 100 | 99.5 | 100 | 94.5 | 56.6 |  | 87.6 | 100 | 98.4 |
| **1545** | 100 | 100 | 98.9 | 100 | 100 | 94.2 |  | 89.3 | 89.6 | 99.8 (0.2) |
| **1546** |  |  |  | 90.0 | 100 |  |  |  | 79.8 | 100 |
| **1547** | 100 | 100 | 98.7 | 100 | 100 | 97.3 | 100 | 92.1 | 90.8 | 99.8 (0.2) |
| **1548** | 100 | 100 | 100 | 55.0 | 100 | 58.0 |  | 89.5 | 100 | 100 |
| **1550** | 100 | 100 | 100 | 93.1 | 100 | 98.6 | 100 | 92.8 | 96.8 | 77.4 |
| **1551** | 100 |  |  | 88.4 | 100 | 55.4 |  | 87.5 | 88.9 | 94.9 |
| **1552** | 100 | 100 | 80.6 | 100 | 100 | 100 | 100 | 91.7 | 85.6 | 99.8 (0.2) |
| **1554** | 100 |  |  | 100 | 100 | 58.9 |  |  | 72.1 | 99.7 |
| **1555** | 100 | 100 | 100 |  |  |  |  | 89.8 | 100 | 98.6 |
| **1556** | 100 |  |  |  | 100 |  | 64.8 | 89.5 | 94.4 | 100 |
| **1558** | 100 |  |  | 94.7 | 100 |  |  | 84.3 | 94.7 | 100 |
| **1559** |  |  |  | 93.1 | 100 | 57.3 |  |  | 83.3 | 100 |
| **1560** | 100 | 100 | 99.6 | 91.0 | 100 | 53.6 |  | 53.8 | 72.5 | 99.8 (0.2) |
| **1561** | 100 |  |  | 92.1 | 100 |  |  | 80.6 | 88.3 | 91.2 |
| **1562** | 100 | 100 | 89.9 | 70.4 | 100 | 50.3 |  | 100 | 79.4 | 99.8 (0.2) |
| **1563** | 100 | 100 |  |  | 100 (0.3) | 56.6 |  | 89.4 | 85.9 | 99.8 (0.2) |
| **1565** | 100 |  | 58.5 | 100 | 97.4 |  |  |  | 90.6 | 93.7 (0.2) |
| **1566** |  |  |  | 91.5 | 100 |  |  |  | 71.6 | 99.7 |
| **1567** |  |  |  |  |  |  |  |  | 88.2 | 74.2 |
| **1568** | 100 | 100 | 100 | 82.5 | 100 | 62.3 | 100 | 100 | 100 | 100 |
| **1569** |  |  | 76.6 | 66.1 |  |  |  |  | 80.7 | 89.3 |
| **1570** | 100 |  |  |  |  |  |  | 95.2 | 79.2 | 99.8 (0.2) |
| **1572** |  |  |  | 93.1 | 100 | 98.6 | 100 | 93.1 | 89.2 | 50.9 |
| **1573** |  |  |  | 85.2 | 100 | 75.9 | 100 |  | 80.7 | 98.1 |
| **1574** | 100 | 100 | 100 | 87.8 | 100 | 6.3 |  |  | 72.4 | 99.8 (0.2) |
| **1575** | 100 | 100 | 100 | 85.2 | 100 | 54.4 |  | 80.5 | 85.8 | 94.0 |
| **1576** | 65.1 | 100 | 100 | 63.0 | 100 |  |  | 79.6 | 84.6 | 99.7 |
| **1577** | 100 |  |  | 92.6 | 100 | 95.1 | 100 |  | 90.3 | 99.8 (0.2) |
| **1578** | 100 | 100 | 99.9 | 100 | 100 | 68.78 | 100 | 100 | 100 | 97.2 |
| **1579** |  |  |  |  |  |  |  |  | 72.7 | 95.2 (0.2) |
| **1580** | 100 | 100 | 100 | 100 | 100 | 100 | 100 | 55.0 | 74.0 | 99.7 |
| **1581** | 100 | 100 |  | 91.0 | 100 | 77.7 |  |  | 71.6 | 93.2 |
| **1582** | 100 |  | 75.8 | 88.4 | 100 | 55.9 |  | 100 | 90.0 | 94.3 |
| **1584** |  | 89.2 | 100 | 84.1 | 100 |  |  |  | 71.2 | 99.7 |
| **1585** | 100 | 100 |  |  |  |  | 100 |  | 79.8 | 98.2 |
| **1586** |  |  |  |  |  |  |  |  | 69.3 | 92.9 |
| **1587** | 100 | 100 | 100 | 100 | 100 | 50.6 |  | 55.6 | 100 | 89.7 |
| **1588** | 100 | 100 | 100 | 100 | 100 | 57.8 |  | 62.1 | 89.3 | 71.9 (0.3) |
| **1589** | 100 | 100 | 100 | 100 | 100 | 91.1 | 100 | 100 | 88.6 | 94.3 |
| **1590** | 100 | 100 | 100 |  |  |  |  | 89.7 | 89.8 | 94.3 (0.2) |
| **1592** | 100 | 100 | 100 | 76.7 | 100 | 55.3 |  | 89.5 | 94.0 (0.1) | 100 |
| **1593** | 100 | 100 | 99.7 | 100 | 100 | 94.8 |  | 89.7 | 89.1 | 99.8 (0.2) |
|  |  |  |  |  |  |  |  |  |  |  |

No value denotes less than 50% sequence coverage for the region and was not used for analysis.

**References**

1. Middleton D, Menchaca L, Rood H, Komerofsky R. New Allele Frequency Database: http://www.allelefrequencies.net. Tissue Antigens 2003:403-107.

2. Neumann-Haefelin C, McKiernan S, Ward S, Viazov S, Spangenberg HC, Killinger T, Baumert TF, et al. Dominant influence of an HLA-B27 restricted CD8+ T cell response in mediating HCV clearance and evolution. Hepatology 2006;43:563-572.

3. McKiernan SM, Hagan R, Curry M, McDonald GSA, Kelly A, Nolan N, Walsh A, et al. Distinct MHC class I and II alleles are associated with hepatitis C viral clearance, originating from a single source. Hepatology 2004;40:108-114.

4. Gaudieri S, Rauch A, Park LP, Freitas E, Herrmann S, Jeffrey G, Cheng W, et al. Evidence of viral adaptation to HLA class I-restricted immune pressure in chronic hepatitis C virus infection. J Virol 2006;80:11094-11104.

5. Kuiken C, Yusim K, Boykin L, Richardson R. The Los Alamos HCV Sequence Database. Bioinformatics 2005;21:379-384.

6. Kumar S, Nei M, Dudley J, Tamura K. MEGA: A biologist-centric software for evolutionary analysis of DNA and protein sequences. Brief Bioinform 2008;9:299-306.

7. Ray SC, Fanning L, Wang XH, Netski DM, Kenny-Walsh E, Thomas DL. Divergent and convergent evolution after a common-source outbreak of hepatitis C virus. J Exp Med 2005;201:1753-1759.

8. Ikatura J, Nattermann J, Eichbaum Q, Heckerman D, Sakamoto N, Fanning LJ, Kenny-Walsh E, et al. Viral load change and sequential evolution of entire hepatitis C virus genome in Irish recipients of single source-contaminated anti-D immunoglobulin. J Virol 2005;12:594-603.

9. Drummond AJ, Rambaut A. BEAST: Bayesian evolutionary analysis by sampling trees. BMC Evol Biol 2007;7.

10. Simmonds P. Genetic diversity and evolution of hepatitis C virus-15 years on. J Gen Virol 2004;85:3173-3188.

11. Rambaut A, Drummond AJ. TRACER version 1.3 In; 2009.

12. Rambaut A. FigTree v1.1.2 In; 2009.
