# Supplementary material for: Effect of Immune Pressure on Hepatitis C Virus Evolution: Insights From a Single-Source Outbreak
Source: Hepatology. 2011 Feb;53(2):396–405. doi: 10.1002/hep.24076 (PMC3044208; doi:10.1002/hep.24076)
Supplement: Supplementary file 2 [file hep0053-0396-SD2.doc]

>HM106522

ATGAGCACGAATCCTAAACCTCAAAGAAAAACCAAACGTAACACCAACCGCCGCCCACAGGACGTCAAGTTCCCGGGYGGTGGTCAGATCGTTGGTGGAGTTTACCTGTTGCCGCGCAGGGGCCCCAGGTTGGGTGTGCGCGCGACTAGGAAGACTTCCGAGCGGTCGCAACCTCGTGGAAGGCGACAACCTATCCCYAAGGCTCGCCGGCCCGAGGGCAGGACCTGGGCTCAGCCCGGGTACCCTTGGCCCCTCTATGGCAATGAGGGCATGGGGTGGGCAGGATGGCTCCTGTCACCCCGYGGCTCTCGGCCTAGTTGGGGCCCCACGGACCCCCGGCGYAGGTCGCGTAATYTGGGTAAGGTCATCGATACCCTCACATGCGGCTTCGCCGAYCTCATGGGGTACATTCCGCTCGTCGGCGCCCCCCTAGGGGGCGCTGCCAGGGCCCTGGCGCATGGCGTCCGGGTCCTGGAGGACGGCGTGAACTATGCAACAGGGAATTTGCCCGGTTGCTCTTTCTCTATCTTCCTCTTGGCTTTGCTGTCCTGTYTGACCATCCCAGYTTCCGCY

>HM106523

ATGAGCACGAATCCTAAACCTCAAAGAAAAACCAAACGTAACACCAACCGCCGCCCACAGGACGTCAAGTTCCCGGGTGGTGGTCAGATCGTTGGTGGAGTTTACCTGTTGCCGCGCAGGGGCCCCAGGTTGGGTGTGCGCGCGACTAGGAAGACTTCCGAGCGGTCGCAACCTCGTGGAAGGCGACAACCTATCCCCAAGGCTCGCCGGCCCGAGGGCAGGACCTGGGCTCAGCCCGGGTACCCTTGGCCCCTCTATGGCAATGAGGGCATGGGGTGGGCAGGATGGCTCCTGTCACCCCGCGGCTCTCGGCCTAGTTGGGGCCCCACGGACCCCCGGCGTAGGTCGCGTAATTTGGGTAAGGTCATCGATACCCTCACATGCGGCTTCGCCGACCTCATGGGGTACATTCCGCTCGTCGGCGCCCCCCTAGGGGGCGCTGCCAGGGCCCTGGCGCATGGCGTCCGGGTCCTGGAGGACGGCGTGAACTATGCAACAGGGAATTTACCCGGTTGCTCTTTCTCTATCTTCCTCTTGGCTTTGCTGTCCTGTTTGACCATCCCAGCTTCCGCC

>HM106524

ATGAGCACGAATCCTAAACCTCAAAGAAAAACCAAACGTAACACCAACCGCCGCCCACAGGACGTTAAGTTCCCGGGTGGYGGTCAGATCGTTGGTGGAGTTTACTTGTTGCCGCGCAGGGGCCCCAGGTTGGGTGTGCGCGCGACTAGGAAGACTTCCGAGCGGTCGCAACCTCGTGGAAGGCGACAACCTATCCCCAAGGCTCGCCGGCCCGAGGGCAGGACCTGGGCTCAGCCCGGGTACCCTTGGCCCCTTTATGGCAATGAGGGCATGGGGTGGGCAGGATGGCTCCTGTCACCCCGCGGCTCTCGGCCTAGTTGGGGCCCCACGGACCCCCGGCGTAGGTCGCGTAATTTGGGTAAGGTCATCGACACCCTCACATGCGGCTTCGCCGACCTCATGGGGTACATTCCGCTCGTCGGCGCCCCCCTAGGGGGCGCTGCCAGGGCCCTGGCGCATGGCGTCCGGGTTCTGGAGGACGGCGTGAACTATGCAACAGGGAATCTGCCYGGTTGCTCTTTCTCTATCTTCCTCTTGGCTTTGCTGTCCTGTTTGACCATCCCAGTTTCCGCT

>HM106525

ATGAGCACGAATCCTAAACCTCAAAGAAAAACYAAACGTAACACCAACCGCCGCCCACAGGACGTCAAGTTCCCGGGTGGTGGTCAGATCGTTGGTGGAGTTTACCTGTTGCCGCGCAGGGGCCCCAGGTTGGGTGTGCGCGCGACTAGGAAGACTTCCGAGCGGTCGCAACCTCGTGGAAGGCGACAACCTATCCCYAAGGCTCGCCGGCCCGAGGGCAGGACCTGGGCTCAGCCCGGGTAYCCCTGGCCCCTCTATGGCAATGAGGGCTTGGGGTGGGCAGGATGGCTCCTRTCACCCCGCGGCTCTCGGCCTAGTTGGGGCCCCACGGACCCCCGGCGTAGGTCGCGTAATCTGGGTAAGGTYATCGATACCCTCACATGCGGCTTCGCCGACCTCATGGGGTACATTCCGCTCGTCGGCKCCCCCCTAGGGGGCGCTGCCAGGGCCCTGGCGCATGGCGTCCGTGTCCTGGAGGACGGCGTGAACTACGCAACAGGGAATTTGCCCGGTTGCTCTTTCTCTATCTTCCTCTTGGCTTTRCTGTCCTGTTTGACCATCCCAACTTCCGCT

>HM106526

ATGAGCACGAATCCTAAACCTCAAAGAAAAACCAAACGTAACACCAACCGCCGCCCACAGGACGTCAAGTTCCCGGGTGGTGGTCAGATCGTTGGTGGAGTTTACCTGTTGCCGCGCAGGGGCCCCAGGTTGGGTGTGCGCGCGACTAGGAAGACTTCCGAGCGGTCGCAACCTCGTGGAAGGCGACAACCTATCCCCAAGGCTCGCCGGCCCGAGGGCAGGACCTGGGCTCAGCCCGGGTAYCCTTGGCCCCTCTATGGCAATGAGGGCATGGGGTGGGCAGGATGGCTCCTGTCACCCCGCGGCTCTCGGCCTAGTTGGGGCCCCACGGACCCCCGGCGTAGGTCGCGTAATTTGGGTAAGGTCATCGATACCCTCACATGCGGCTTCGCCGACCTCATGGGGTACATTCCGCTCGTCGGCGCCCCCCTAGGGGGCGCTGCCAGGGCCCTGGCRCATGGCGTCCGGGTTCTGGAGGACGGCGTGAACTATGCAACAGGGAATCTGCCCGGTTGCTCTTTCTCTATCTTCCTCTTGGCYTTGCTGTCCTGCTTGACCATCCCAGYTTCCGCT

>HM106527

ATGAGCACAAATCCTAAACCTCAAAGAAAAACCAAACGTAACACCAACCGCCGCCCACAGGACGTCAAGTTCCCGGGTGGTGGTCAGATCGTTGGTGGAGTTTACCTGTTGCCGCGCAGGGGCCCCAGGTTGGGTGTGCGCGCGACTAGGAAGACTTCCGAGCGGTCGCAACCTCGTGGAAGGCGACAACCTATCCCCAAGGCTCGCCGGCCCGAGGGCAGGACCTGGGCTCAGCCCGGGTACCCYTGGCCCCTCTATGGCAATGAGGGCATGGGGTGGGCAGGATGGCTCCTGTCACCCCGTGGYTCTCGGCCTAGTTGGGGCCCCACGGACCCCCGGCGTAGGTCGCGTAATTTGGGTAAGGTCATCGATACCCTCACATGCGGCTTCGCCGACCTCATGGGGTACATTCCGCTCGTCGGCGCCCCCCTAGGGGGCGCTGCCAGGGCCCTGGCGCATGGCGTCCGGGTCCTGGAGGACGGCGTGAACTAYGCAACAGGGAATTTGCCCGGTTGCTCTTTCTCTATCTTCCTCTTGGCTTTGCTGTCCTGTTTGACCATCCCAGCTTCCGCT

>HM106528

ATGAGCACGAATCCTAAACCTCAAAGAAAAACCAAACGTAACACCAACCGCCGCCCACAGGACGTCAAGTTCCCGGGTGGTGGTCAGATCGTTGGTGGAGTTTACCTGTTGCCGCGCAGGGGCCCCAGGTTGGGTGTGCGCGCGACTAGGAAGACTTCCGAGCGGTCGCAACCTCGTGGAAGGCGACAACCTATCCCCAAGGCTCGCCGGCCCGAGGGCAGGACCTGGGCTCAGCCCGGGTACCCTTGGCCCCTCTATGGCAATGAGGGCATGGGGTGGGCAGGATGGCTCCTGTCACCCCGCGGCTCTCGGCCTAGTTGGGGCCCCACGGACCCCCGGCGTAGGTCGCGTAATTTGGGTAAGGTCATCGATACCCTYACATGCGGCTTCGCCGACCTCATGGGGTACATTCCGCTCGTCGGCGCCCCCCTAGGGGGCGCTGCCAGGGCCCTGGCGCATGGCGTCCGGGTCCTGGAGGACGGCGTGAACTATGCAACAGGGAATTTGCCCGGTTGCTCTTTCTCTATCTTCCTCTTGGCCTTGCTGTCCTGTTTGACCATCCCAGTTTCCGCT

>HM106529

ATGAGCACAACTCCTAAACCTCAAAGAAAAACCAAACGTAACACCAACCGCCGCCCACAGGACGTCAAGTTCCCGGGTGGTGGTCAGATCGTTGGTGGAGTTTACCTGTTGCCGCGCAGGGGCCCCAGGTTGGGTGTGCGCGCGACTAGGAAGACTTCCGAGCGGTCGCAACCTCGTGGAAGGCGACAACCTATCCCCAAGGCTCGCCGGCCCGAGGGCAGGACCTGGGCTCAGCCCGGGTACCCTTGGCCCCTCTATGGCAATGAGGGCATGGGGTGGGCAGGATGGCTCCTGTCACCCCGCGGCTCTCGGCCTAGTTGGGGTCCCACGGACCCCCGGCGTAGGTCGCGTAACTTGGGTAAGGTCATCGATACCCTCACATGCGGCTTCGCCGACCTCATGGGGTACATTCCGCTCGTCGGCGCCCCCCTAGGGGGCGCTGCCAGGGCCCTGGCGCATGGCGTCCGGGTCCTGGAGGACGGCGTGAACTATGCAACAGGGAATTTRCCCGGTTGCTCTTTCTCTATCTTCCTCTTGGCTTTGCTGTCCTGTTTGACCATCCCAGCTTCCGCT

>HM106530

ATGAGCACGAATCCTAAACCTCAAAGAAAAACCAAACGTAACACCAACCGCCGCCCACAGGACGTCAAGTTCCCGGGTGGTGGTCAGATCGTTGGTGGAGTTTACCTGTTGCCGCGCAGGGGCCCCAGGTTGGGTGTGCGCGCGACTAGGAAGACTTCCGAGCGGTCGCAACCTCGTGGAAGGCGACAACCTATCCCCAAGGCTCGCCGGCCCGAGGGCAGGACCTGGGCYCAGCCCGGGTACCCTTGGCCCCTCTATGGCAATGAGGGCATGGGGTGGGCAGGATGGCTCCTGTCACCCCGCGGCTCTCGGCCTAGTTGGGGCCCCACGGACCCCCGGCGTAGGTCGCGYAAYTTGGGTAAGGTCATCGATACCCTCACRTGCGGCTTCGCCGACCTCATGGGGTACATTCCGCTCGTCGGCGCCCCCCTAGGGGGCGYTGCCAGGGCCCTGGCGCATGGCGTCCGGGTTCTGGAGGACGGCGTGAACTATGCAACAGGGAATMTGCCCGGTTGCTCTTTCTCTATCTTCCTCTTGGCTTTGCTGTCCTGTTTGACCATCCCAGCCTCCGCT

>HM106531

ATGAGCACAAATCCTAAACCTCAAAGAAAAACCAAACGTAACACCAACCGCCGCCCACAGGACGTCAAGTTCCCGGGTGGTGGTCAGATCGTTGGTGGAGTTTACCTGTTGCCGCGCAGGGGCCCCAGGTTGGGTGTGCGCGCGACTAGGAAGACTTCCGAGCGGTCGCAACCTCGTGGAAGGCGACAACCTATCCCCAAGGCTCGCCGGCCCGAGGGCAGGACCTGGGCTCAGCCCGGGTACCCCTGGCCCCTCTATGGCAATGAGGGCATGGGGTGGGCAGGATGGCTCCTGTCACCCCGCGGCTCTCGGCCTAGTTGGGGCCCCACGGACCCCCGGCGTAGGTCGCGTAATCTGGGTAAGGTCATCGATACCCTCACATGCGGCTTCGCCGACCTCATGGGGTACATTCCGCTCGTCGGCGCCCCCCTAGGGGGCGCTGCCAGGGCCCTGGCGCATGGCGTCCGGGTCCTGGARGACGGCGTGAACTAYGCAACAGGGAATTTGCCCGGTTGCTCTTTTTCTATCTTCCTCTTGGCTTTGCTGTCCTGTTTGACCATCCCAGCTTCCGCY

>HM106532

ATGAGCACGAATCCTAAACCTCAAAGAAAAACCAAACGTAACACCAACCGCCGCCCACAGGACGTCAAGTTCCCGGGTGGTGGTCAGATCGTTGGTGGAGTTTACCTGTTGCCGCGCAGGGGCCCCAGGTTGGGTGTGCGCGCGCCTAGGAAGACTTCCGAGCGGTCGCAACCTCGTGGAAGGCGACAACCTATCCCCAAGGCTCGCCGGCCCGAGGGCAGGACCTGGGCTCAGCCCGGGTACCCYTGGCCCCTCTATGGCAATGAGGGCATGGGGTGGGCAGGATGGCTCCTGTCACCCCGCGGCTCTCGGCCTAGTTGGGGCCCCACGGACCCCCGGCGTAGGTCGCGTAAYTTGGGTAAGGTCATCGATACCCTCACATGCGGCTTCGCCGACCTCATGGGGTACATTCCGCTCGTCGGCGCCCCCCTAGGGGGCGCTGCCAGGGCCCTGGCGCATGGCGTCCGGGTTCTGGAGGACGGCGTGAACTATGCAACAGGGAATCTGCCCGGTTGCTCTTTCTCTATCTTCCTCTTGGCTTTGCTGTCCTGTTTGACCATCCCAGCWTCCGCT

>HM106533

ATGAGCACGAATCCTAAACCTCAAAGAAAAACCAAACGTAACACCAACCGCCGCCCACAGGACGTCAAGTTCCCGGGTGGTGGTCAGATCGTTGGTGGAGTTTACCTGTTGCCGCGCAGGGGCCCCAAGTTGGGTGTGCGCGCGACTAGGAAGACTTCCGAGCGGTCGCAACCTCGTGGAAGGCGACAACCTATCCCCAAGGCTCGCCGGCCCGAGGGCAGGACCTGGGCTCAGCCCGGGTACCCTTGGCCCCTCTATGGCAATGAGGGCATGGGGTGGGCAGGATGGCTCCTGTCACCCCGCGGCTCTCGACCTAGTTGGGGCCCCACGGACCCCCGGCGTAGGTCGCGTAATTTGGGTAAGGTCATCGAYACCCTCACATGCGGCTTCGCCGATCTCATGGGGTACATTCCGCTCGTCGGCGCCCCCCTAGGGGGCGCTGCCAGGGCCYTGGCGCATGGCGTCCGGGTCCTGGAGGACGGCGTGAACTATGCAACAGGGAATTTACCCGGTTGCTCTTTCTCTATCTTCCTCTTGGCTCTGCTGTCCTGTTTGACCATCCCAGCTTCCGCT

>HM106534

ATGAGCACGAATCCTAAACCTCAAAGAAAAACCAAACGTAACACCAACCGCCGCCCACAGGACGTCAAGTTCCCGGGTGGTGGTCAGATCGTTGGTGGAGTTTACCTGTTGCCGCGCAGGGGCCCCAGGTTGGGTGTGCGCGCGACTAGGAAGACTTCCGAGCGGTCGCAACCTCGTGGAAGGCGACAACCTATCCCCAAGGCTCGCCGGCCCGAGGGCAGGACCTGGGCTCAGCCCGGGTACCCTTGGCCCCTCTAYGGCAATGAGGGCATGGGGTGGGCAGGATGGCTCCTGTCACCCCGYGGCTCTCGGCCTAGTTGGGGCCCCACGGACCCCCGGCGTAGGTCGCGTAATTTGGGTAAGGTCATCGATACCCTCACATGCGGCTTCGCCGAYCTCATGGGGTACATTCCGCTCGTCGGCGCCCCCYTAGGGGGCGCTGCYAGGGCCCTGGCGCATGGCGTCCGGGTCCTGGAGGACGGCGTGAACTATGCAACAGGGAATTTGCCCGGTTGCTCTTTCTCTATCTTCCTCTTGGCTTTGCTGTCYTGTTTGACCATCCCAGCTTCCGCT

>HM106535

ATGAGCACGAATCCTAAACCTCAAAGAAAAACCAAACGTAACACCAACCGCCGCCCACAGGACGTCAAGTTCCCGGGTGGTGGTCAGATCGTTGGTGGAGTTTACCTGTTGCCGCGCAGGGGCCCCAGGTTGGGTGTGCGCGCGACTAGGAAGACTTCCGAGCGGTCGCAACCTCGTGGAAGGCGACAACCTATCCCCAAGGCTCGCCGGCCCGAGGGCAGGGTCTGGGCTCAGCCCGGGTACCCTTGGCCCCTCTATGGCAATGAGGGCATGGGGTGGGCAGGATGGCTCCTGTCGCCCCGCGGCTCTCGGCCTAGTTGGGGCCCCACGGACCCCCGGCGTAGGTCGCGTAATTTGGGTAAGGTCATCGATACCCTCACATGCGGCTTCGCCGATCTCATGGGGTACATTCCGCTCGTCGGCGCCCCCCTAGGGGGCGCTGCCAGGGCTCTGGCGCATGGCGTCCGGGTCCTGGAGGACGGCGTGAACTATGCAACAGGGAATTTGCCCGGTTGCTCTTTCTCTATCTTCCTCTTGGCTTTGCTGTCCTGYCTGACCATCCCAGCTTCCGCT

>HM106536

ATGAGCACGAATCCTAAACCTCAAAGAAAAACCAACCGTAACACCAACCGCCGCCCACAGGACGTCAAGTTCCCGGGTGGTGGTCAGATCGTTGGTGGAGTTTACCTGTTGCCGCGCAGGGGCCCCAGGTTGGGTGTGCGCGCGACTAGGAAGACTTCCGAGCGGTCGCAACCTCGTGGAAGGCGACAACCTATCCCCAAGGCTCGCCGGCCCGAGGGCAGGACCTGGGCTCAGCCCGGGTACCCTTGGCCCCTCTATGGCAATGAGGGCATGGGGTGGGCAGGATGGCTCCTGTCACCCCGCGGCTCTCGGCCTAGTTGGGGCCCCACGGACCCCCGGCGTAGGTCGCGTAATTTGGGTAAGGTCATCGATACCCTCACATGCGGCTTCGCCGACCTCATGGGGTACATTCCGCTCGTCGGCGCCCCCCTAGGGGGCGCTGCCAGGGCCCTGGCGCATGGTGTCCGGGTCCTGGAGGACGGCGTGAACTATGCAACAGGGAATTTGCCCGGTTGCTCTTTCTCTATCTTCCTCTTGGCTTTGCTGTCCTGTTTGACCATCCCAGCTTCCGCT

>HM106537

ATGAGCACGAATCCTAAACCTCAAAGAAAAACCAAACGTAACACCAACCGCCGCCCACAGGACGTCAAGTTCCCGGGTGGTGGTCAGATCGTTGGTGGAGTTTACCTGTTGCCGCGCAGGGGCCCCAAGTTGGGTGTGCGCGCGACTAGGAAGACTTCCGAGCGGTCACAACCTCGTGGAAGGCGACAACCTATCCCCAAGGCTCGCCGGCCCGAGGGCAGGACCTGGGCTCAGCCCGGGTACCCTTGGCCCCTCTATGGCAATGAGGGCATGGGGTGGGCAGGATGGCTCCTGTCACCCCGTGGCTCTCGGCCTAGTTGGGGCCCCACGGACCCCCGGCGTAGGTCGCGTAATTTGGGTAAGGTCATCGATACCCTCACATGCGGCTTCGCCGACCTCATGGGGTACATTCCGCTCGTCGGCGCCCCCTTAGGGGGCGCTGCCAGGGCCCTGGCGCATGGCGTCCGGGTCCTGGAGGACGGCGTGAACTATGCAACAGGGAATTTGCCCGGTTGCTCTTTCTCTATCTTCCTCTTGGCTYTGCTGTCTTGTTTGACCATTCCAGCTTCCGCT

>HM106538

ATGAGCACGAATCCTAAACCTCAAAGAAAAACCAAACGTAACACCAACCGCCGCCCACAGGACGTCAAGTTCCCGGGTGGTGGTCAGATCGTTGGTGGAGTTTACCTGTTGCCGCGCAGGGGCCCCARGTTGGGTGTGCGCGCGACTAGGAAGACTTCCGAGCGGTCGCAACCTCGTGGAAGGCGACAACCTATCCCCAAGGCTCGCCGGCCCGAGGGCAGGACCTGGGCTCAGCCCGGGTATCCTTGGCCCCTCTATGGCAATGAGGGCATGGGGTGGGCAGGATGGCTCCTGTCACCCCGCGGCTCTCGGCCTAGTTGGGGCCCCACGGACCCCCGGCGTAGGTCGCGTAATTTGGGTAAGGTCATCGATACCCTCACATGCGGCTTCGCCGACCTCATGGGGTACATTCCGCTCGTCGGCGCCCCCCTAGGGGGCGCTGCCAGGGCCCTGGCGCATGGCGTCCGGGTCCTGGAGGACGGCGTGAACTATGCAACAGGGAAYTTRCCCGGTTGCTCTTTCTCTATCTTCCTCTTGGCTTTGCTGTCCTGTTTGACCATCCCAGCTTCCGCT

>HM106539

ATGAGCACAAATCCTAAACCTCAAAGAAAAACCAAACGTAACACCAACCGCCGCCCACAGGACGTCAAGTTCCCGGGTGGTGGTCAGATCGTTGGTGGAGTTTACCTGTTGCCGCGCAGGGGCCCCAGGTTGGGTGTGCGCGCGACTAGGAAGACTTCCGAGCGGTCGCAACCTCGTGGAAGGCGACAACCTATCCCCAAGGCTCGCCGGCCCGAGGGCAGGACCTGGGCTCAGCCCGGGTACCCTTGGCCCCTCTATGGCAATGAGGGCATGGGGTGGGCAGGATGGCTCCTGTCACCCCGCGGCTCTCGGCCTAGTTGGGGCCCCACGGACCCCCGGCGTAGGTCGCGTAATTTGGGTAAGGTCATCGATACCCTCACATGCGGCTTCGCCGACCTCATGGGGTACATTCCGCTCGTCGGCGCCCCCCTAGGGGGCGCTGCCAGGGCCCTGGCGCATGGCGTCCGGGTCCTGGAGGACGGCGTGAACTATGCAACAGGGAATTTGCCCGGTTGCTCTTTCTCTATCTTCCTYTTGGCTTTGCTGTCCTGTTTGACCATCCCAGCTTCCGCT

>HM106540

ATGAGCACAAATCCTAAACCTCAAAGAAAAACCAAACGTAACACCAACCGCCGCCCACAGGACGTYAAGTTCCCGGGTGGTGGTCAGATCGTTGGTGGAGTTTACCTGTTGCCGCGCAGGGGCCCCAGGTTGGGTGTGCGCRCRACTAGGAAGACTTCCGAGCGGTCGCAACCTCGTGGAAGGCGACAACCTATCCCCAAGGCTCGCCGGCCCGAGGGCAGGACCTGGGCTCAGCCCGGGTACCCTTGGCCCCTCTATGGCAATGAGGGCATGGGGTGGGCAGGATGGCTCCTGTCACCCCGYGGCTCTCGGCCTAGTTGGGGCCCCAMKGACCCCCGGCGYAGGTCGCGTAATTTGGGTAAGGTCATCGATACCCTCACATGCGGCTTCGCCGACCTCATGGGGTACATTCCGCTCGTCGGCGCCCCCCTAGGGGGCGCTGCCAGGGCCCTGGCGCATGGCGTCCGGGTCCTGGAGGACGGCGTGAACTATGCAACAGGGAATTTGCCCGGTTGCTCTTTCTCTATCTTCCTYTTGGCTTTGCTGTCCTGTTTGACCATYCCAGCTTCCGCT

>HM106541

ATGAGCACGAATCCTAAACCTCAAAGAAAAACCAAACGTAACACCAACCGTCGCCCACAGGACGTCAAGTTCCCGGGTGGCGGTCAGATCGTTGGTGGAGTTTACCTGTTGCCGCGCAGGGGCCCCAGGTTGGGTGTGCGCGCGACTAGGAAGACTTCCGAGCGGTCGCAACCTCGTGGAAGGCGACAACCTATCCCCARGGCTCGCCGGCCCGAGGGCAGGACCTGGGCTCAGCCCGGGTACCCTTGGCCCCTCTATGGCAATGAGGGCATGGGGTGGGCAGGATGGCTCCTGTCACCCCGCGGCTCTCGGCCTAGTTGGGGCCCCACGGACCCCCGGCGTAGGTCGCGTAACTTGGGTAAGGTCATCGATACCCTCACATGCGGCTTCGCCGACCTCATGGGGTACATTCCGCTCGTCGGCGCCCCCCTAGGGGGCGCTGCCAGGGCCCTGGCGCATGGCGTCCGGGTCCTGGAGGACGGCGTGAACTATGCAACAGGGAATTTGCCCGGTTGCTCTTTCTCTATCTTCCTCTTGGCTTTGCTGTCCTGTTTGACCATCCCAGCTTCTGCC

>HM106542

ATGAGCACGAATCCTAAACCTCAAAGAAAAACCAAACGTAACACCAACCGCCGCCCACAGGACGTCAAGTTCCCGGGTGGTGGTCAGATCGTTGGTGGAGTTTACCTGTTGCCGCGCAGGGGCCCCAAGTTGGGTGTGCGCGCGACTAGGAAGACTTCCGAGCGGTCGCAACCTCGTGGAAGGCGACAACCTATCCCCAAGGCTCGCCGGCTCGAGGGCAGGACCTGGGCCCAGCCCGGGTACCCTTGGCCCCTCTATGGCAATGAGGGCATGGGGTGGGCAGGATGGCTCCTGTCACCCCACGGCTCTCGGCCTAATTGGGGCCCCACGGACCCCCGGCGTAGGTCGCGTAATTTGGGTAAGGTCATCGATACCCTCACATGCGGCTTCGCCGATCTCATGGGGTACATTCCGCTCGTCGGCGCCCCCCTAGGGGGCGTTGCCAGGGCCCTGGCGCATGGCGTCCGGGTCCTGGAGGACGGCGTGAACTATGCAACAGGGAATTTGCCCGGTTGCTCTTTCTCTATCTTCCTCTTGGCTTTGCTGTCCTGCTTGACCATCCCAGCTTCCGCT

>HM106543

ATGAGCACGAATCCTAAACCTCAAAGAAAAACCAAACGTAACACCAACCGCCGCCCACAGGACGTCAAGTTCCCGGGTGGTGGTCAGATCGTTGGTGGAGTTTACCTGTTGCCGCGCAGGGGCCCCAGGTTGGGTGTGCGCGCGACTAGGAAGACTTCCGAGCGGTCACAACCTCGTGGAAGGCGACAACCTATCCCCAAGGCTCGCCAGCCCGAGGGCAGGACCTGGGCCCAGCCCGGGTACCCTTGGCCCCTCTACGGCAATGAGGGCATGGGGTGGGCAGGATGGCTCCTGTCGCCCCGTGGCTCTCGGCCTAGTTGGGGCCCCACAGACCCCCGGCGTAGGTCGCGTAATTTGGGTAAGGTCATCGATACCCTCACATGCGGCTTCGCCGACCTCATGGGGTACATTCCGCTCGTCGGCGCCCCCCTAGGGGGCGCTGCCAGGGCCCTGGCGCATGGCGTTCGGGTCCTGGAGGACGGCGTGAACTATGCAACAGGGAATTTGCCCGGTTGCTCTTTCTCTATCTTCCTCTTGGCTTTGCTGTCCTGTTTGACCATCCCAGCTTCCGCC

>HM106544

ATGAGCACGAATCCTAAACCTCAAAGAAAAACCAAACGTAACACCAACCGCCGCCCACAGGACGTCAGGTTCCCGGGTGGTGGTCAGATCGTTGGTGGAGTTTACCTGTTGCCGCGCAGGGGCCCCAGGTTGGGTGTGCGCGYGCCCAGGAAGACTTCCGAGCGGTCGCAACCTCGTGGAAGGCGACAACCTATCCCCAAGGCTCGCCGGCCCGAGGGCAGGACCTGGGCTCAGCCCGGGTACCCTTGGCCCCTCTATGGCAATGAGGGCATGGGGTGGGCAGGATGGCTCCTGTCACCCCGCGGCTCTCGGCCTAGTTGGGGCCCCACGGACCCCCGGCGTAGGTCGCGTAATTTGGGTAAGGTCATCGATACCCTCACATGCGGCTTCGCCGACCTCATGGGGTACATTCCGCTCGTCGGCGCCCCCCTAGGGGGCGCTGCCAGGGCCCTGGCGCATGGCGTCCGGGTCCTGGAGGACGGCGTGAACTATGCAACAGGGAATTTGCCCGGTTGCTCTTTCTCTATCTTCCTCTTGGCTTTGCTGTCCTGTTTGACCATCCCAGCTTCCGCT

>HM106545

ATGAGCACGAATCCTAAACCTCAAAGAAAAACCAAACGTAACACCAACCGCCGCCCACAGGACGTCAAGTTCCCGGGTGGTGGTCAGATCGTTGGTGGAGTTTACCTGTTGCCGCGCAGGGGCCCCAGGTTGGGTGTGCGCGCGACTAGGAAGACTTCCGAGCGGTCGCAACCTCGTGGAAGGCGACAACCTATCCCCAAGGCTCGCCGGCCCGAGGGCAGGACCTGGGCTCAGCCCGGGTACCCTTGGCCCCTCTATGGCAATGAGGGCCTGGGGTGGGCAGGATGGCTCCTGTCACCCCGCGGCTCTCGGCCTAGCTGGGGCCCCACGGACCCCCGGCGTAGGTCGCGTAATTTGGGTAAGGTCATCGATACCCTCACATGCGGCTTCGCCGACCTCATGGGGTACATTCCGCTCGTCGGCGCCCCCCTAGGGGGCGCTGCCAGGGCCCTGGCGCATGGCGTCCGGGTCCTGGAGGACGGCGTGAACTACGCAACAGGGAATTTGCCCGGTTGCTCTTTCTCTATCTTCCTCTTGGCTTTGCTGTCCTGTCTGACTATCCCAGCTTCCGCC

>HM106546

ATGAGCACGAATCCTAAACCTCAAAGAAAAACCAAACGTAACACCAACCGCCGCCCACAGGACGTCAAGTTCCCGGGTGGTGGTCAGATCGTTGGTGGAGTTTACCTGTTGCCGCGCAGGGGCCCCAGGTTGGGTGTGCGCGCGACTAGGAAGACTTCCGAGCGGTCGCAACCTCGTGGAAGGCGACAACCTATCCCCAAGGCTCGCCGGCCCGAGGGCAGGACCTGGGCTCAGCCCGGGTACCCTTGGCCCCTCTAYGGCAATGAGGGCHTGGGGTGGGCAGGATGGCTCCTGTCACCCCGCGGCTCYCGGCCTAGTTGGGGCCCCACGGACCCCCGGCGTAGGTCGCGTAATTTGGGTAAGGTCATCGATACCCTCACATGCGGCTTCGCCGACCTCATGGGGTACATTCCGCTCGTCGGCGCCCCCYTAGGGGGCGCTGCCAGGGCCCTGGCGCATGGCGTCCGGGTCCTGGAGGACGGCGTGAACTATGCAACAGGGAATTTGCCCGGTTGCTCTTTCTCTATCTTCCTCTTGGCTTTGCTGTCCTGTTTGACCATCCCAGCYTCCGCT

>HM106547

ATGAGCACGAATCCTAAACCTCAAAGAAAAACCAAACGTAACACCAACCGCCGCCCACAGGACGTCAAGTTCCCGGGTGGTGGTCAGATCGTTGGTGGAGTTTACCTGTTGCCGCGCAGGGGCCCCAGGTTGGGTGTGCGCGCGACTAGGAAGACTTCCGAGCGGTCGCAACCTCGTGGAAGGCGACAACCTATCCCCAAGGCTCGCCGGCCCGAGGGCAGGACCTGGGCTCAGCCCGGGTACCCTTGGCCCCTCTATGGCAATGAGGGCATGGGGTGGGCAGGATGGCTCCTGTCGCCCCGCGGCTCTCGGCCTAATTGGGGCCCCACGGACCCCCGGCGTAGGTCGCGTAATTTGGGTAAGGTCATCGATACCCTCACATGCGGCTTCGCCGACCTCATGGGGTACATTCCGCTCGTCGGCGCCCCCCTAGGGGGCGTTGCCAGGGCCCTGGCGCATGGCGTCCGGGTCCTGGAGGACGGCGTGAACTATGCAACAGGGAATCTACCCGGTTGCTCTTTCTCTATCTTCCTCTTAGCTTTGCTGTCCTGTTTGACCATCCCAGCTTCCGCT

>HM106548

ATGAGCACGAATCCTAAACCTCAAAGAAAAACCAAACGTAACACCAACCGCCGCCCACAGGACGTCAAGTTCCCGGGTGGTGGTCAGATCGTTGGTGGAGTTTACCTGTTGCCGCGCAGGGGCCCCAGGTTGGGTGTGCGCGCGACTAGGAAGACTTCCGAGCGGTCGCAACCTCGTGGAAGGCGACAACCTATCCCCAAGGCTCGCCGGCCCGAGGGCAGGACCTGGGCTCAGCCCGGGTACCCTTGGCCCCTCTATGGCAAYGAGGGCATGGGGTGGGCAGGATGGCTCCTGTCACCCCGCGGCTCTCGGCCTAGTTGGGGCCCCACGGACCCCCGGCGTAGGTCGCGTAATTTGGGTAAGGTCATCGATACCCTCACATGCGGCTTCGCCGACCTCATGGGGTACATTCCGCTCGTCGGCGCCCCCCTAGGGGGCGCTGCCAGGGCCCTGGCGCATGGCGTCCGGGTCTTGGAGGACGGCGTGAACTATGCAACAGGGAATTTGCCCGGTTGCTCTTTCTCTATCTTCCTYTTRGCTTTGCTGTCCTGCTTGACCATCCCAGCTTCCGCT

>HM106549

ATGAGCACGAATCCTAAACCTCAAAGRAAAACCAAACGTAACACCAACCGCCGCCCACAGGACGTCAAGTTCCCGGGTGGTGGTCAGATCGTTGGTGGAGTTTACCTGTTGCCGCGCAGGGGCCCCAGGTTGGGTGTGCGCGCGACTAGGAAGACTTCCGAGCGGTCGCAACCTCGTGGAAGGCGACAACCTATCCCCAAGGCTCGCCGGCCCGAGGGCAGGACCTGGGCTCAGCCCGGGTACCCTTGGCCCCTCTATGGCAATGAGGGCCTGGGGTGGGCAGGATGGCTCCTGTCACCCCGCGGCTCTCGGCCTAGTTGGGGCCCCACGGACCCCCGGCGTAGGTCGCGTAATTTGGGTAAGGTCATCGACACCCTCACATGCGGCTTCGCCGATCTCATGGGGTACATTCCGCTCGTCGGCGCCCCCCTAGGGGGCGCTGCCAGGGCCCTGGCGCATGGCGTCCGGGTCCTGGAGGACGGCGTGAACTATGCAACAGGGAATTTGCCCGGTTGCTCTTTCTCTATCTTCCTCTTGGCTTTGCTGTCCTGTTTGACCATCCCAGCTTCCGCT

>HM106550

ATGAGCACGAATCCTAAACCTCAAAGAAAAACCAAACGTAACACCAACCGCCGCCCACAGGACGTCAAGTTCCCGGGTGGTGGTCAGATCGTTGGTGGAGTTTACCTGTTGCCGCGCAGGGGCCCCAGGTTGGGTGTGCGCGCGACTAGGAAGACTTCCGAGCGGTCGCAACCTCGTGGAAGGCGACAACCTATCCCCAAGGCTCGCCGGCCCGAGGGCAGGACCTGGGCTCAGCCCGGGTACCCTTGGCCCCTCTATGGCAATGAGGGCATGGGGTGGGCAGGATGGCTCCTGTCACCCCGCGGCTCTCGGCCTAGTTGGGGCCCCACGGACCCCCGGCGTAGGTCGCGTAATTTGGGTAAGGTCATCGATACCCTCACATGCGGCTTCGCCGACCTCATGGGGTACATTCCGCTCGTCGGCGCCCCCCTAGGGGGCGCTGCCAGGGCCCTGGCGCATGGCGTCCGAGTCCTGGAGGACGGCGTGAACTATGCAACAGGGAATTTGCCCGGTTGCTCTTTCTCTATCTTCCTCTTGGCTTTGCTGTCCTGTTTGACCATTCCAGCTTCCGCT

>HM106551

ATGAGCACGAATCCTAAACCTCAAAGAAAAACCAAACGTAACACCAACCGCCGCCCACAGGACGTCAAGTTCCCGGGTGGTGGTCAGATCGTTGGTGGAGTTTACCTGTTGCCGCGCAGGGGCCCCAGGTTGGGTGTGCGCGCGACTAGGAAGACTTCCGAGCGGTCGCAACCTCGTGGAAGGCGACAACCTATCCCCAAGGCTCGCCGGCCCGAGGGCAGGACCTGGGCTCAGCCCGGGTACCCTTGGCCCCTCTATGGCAATGAGGGCATGGGGTGGGCAGGATGGCTCCTGTCACCCCGCGGCTCTCGGCCTAGTTGGGGCCCCACGGACCCCCGGCGTAGGTCGCGTAATTTGGGTAAGGTCATCGATACCCTCACATGCGGCTTCGCCGACCTCATGGGGTACATTCCGCTCGTCGGCGCCCCCCTAGGGGGCGCTGCCAGGGCCCTGGCGCATGGCGTCCGGGTCCTGGAGGACGGCGTGAACTATGCAACAGGGAATYTGCCCGGTTGCTCTTTCTCTATCTTCCTCTTGGCTTTGCTGTCCTGTTTGACCAYCCCAGCTTCCGCT

>HM106552

ATGAGCACGAATCCTAAACCTCAAAGAAAAACCAAACGTAACACCAACCGCCGCCCWCAGGACGTTAAGTTCCCGGGTGGTGGTCAGATCGTTGGTGGAGTTTACCTGTTGCCGCGCAGGGGCCCCAGGTTGGGTGTGCGCGCGCCCAGGAAGACTTCCGAGCGGTCGCAASCTCGTGGAAGGCGACAACCTATCCCCAAGGCTCGCCGGCCCGAGGGCAGRACCTGGGCTCAGCCCGGGTACCCTTGGCCCCTCTATGGCAATGAGGGCATGGGGTGGGCAGGATGGCTCCTGTCACCCCGTGGCTCTCGGCCTAGTTGGGGCCCCACGGACCCCCGGCGTAGGTCGCGTAATTTGGGTAAGGTCATCGATACCCTCACATGCGGCTTCGCCGACCTCATGGGGTACATTCCGCTCGTCGGCGCCCCCCTAGGGGGCGCTGCCAGRGCCCTGGCGCATGGCGTCCGGGTCCTGGAGGACKGCGTGAACTATGCAACAGGGAATTTGCCCGGTTGCTCTTTCTCTATCTTCCTCTTGGCTYTGCTGTCCTGTTTGACCATCCCAGCATCCGCT

>HM106553

ATGAGCACGAATCCTAAACCTCAAAGAAAAACCAAMCGTAACACCAACCGCCGCCCACAGGACGTCAAGTTCCCGGGTGGCGGTCAGATCGTTGGTGGAGTTTACCTGTTGCCGCGCAGGGGCCCCAGGTTGGGTGTGCGCGCGACTAGGAAGACTTCCGAGCGGTCGCAACCTCGTGGAMGGCGTCAACCTATCCCCAAGGCTCGCCGGCCCGAGGGCAGRACCTGGGCTCAGCCCGGGTACCCTTGGCCCCTCTATGGCAATGAGGGCATGGGGTGGGCAGGATGGCTCCTGTCACCCCGCGGYTCTCGGCCTAGTTGGGGCCCCACGGACCCCCGGCGTAGGTCGCGTAATTTGGGTAAGGTCATCGATACCCTCACATGCGGCTTCGCCGACCTCATGGGGTACATTCCGCTCGTCGGCGCCCCCCTAGGGGGCGCTGCCAGGGCCCTGGCGCATGGCGTCCGGGTCCTGGAGGACGGCGTGAACTATGCAACAGGGAATTTGCCCGGTTGCTCTTTCTCTATCTTCCTCTTGGCYTTGCTGTCCTGTTTGACCATCCCAGCTTCCGCT

>HM106554

ATGAGCACGAATCCTAAACCTCAAAGAAAAACCAAACGTAACACCAACCGCCGCCCACAGGACGTCAAGTTCCCGGGTGGTGGTCAGATCGTTGGTGGAGTTTACCTGTTGCCGCGCAGGGGCCCCAGGTTGGGTGTGCGCGCGACTAGGAAGACTTCCGAGCGGTCGCAACCTCGTGGAAGGCGACAACCTATCCCCAAGGCTCGTCGKCCCGAGGGCAGGACCTGGGCTCAGCCCGGGTACCCTTGGCCCCTCTATGGCAATGAGGGCATGGGATGGGCAGGATGGCTCCTGTCACCCCGCGGCTCTCGGCCTAGTTGGGGCCCCACGGACCCCCGGCGTAGGTCGCGTAATTTGGGTAAGGTCATCGATACCCTCACATGCGGCTTCGCCGACCTCATGGGGTACATTCCGCTCGTCGGCGCCCCCCTAGGGGGCGCTGCCAGGGCCCTGGCGCATGGCGTCCGGGTCCTGGAAGACGGCGTGAACTATGCAACAGGGAATTTGCCCGGTTGCTCTTTCTCTATCTTCCTCTTGGCTTTGCTGTCCTGYTTGACCATCCCAGCTTCCGCT

>HM106555

NNNNNNNNNNNNNNNNNNNNNNNNNNNNNNNNNNNNNNNNNNNNNNNNNNNNNNNNNNNNNNNNNNNNNNNNNNNNNNNNNNNNNNNNNNNNNNNNNNNNNNNNNNNNNNNNNNNNNNNNNNNNNNNNNNNNNNNNNNNNNNNNNNNNNNNNNNNNNNNNNNNNNNNNNNNNNNNNNNNNNNNNNNNNNNNNNNNNNNNNGGCTCGCCGGCCCGAGGGCAGGACCTGGGCTCAGCCCGGGTACCCTTGGCCCYTCTATGGYAATGAGGGCATGGGGTGGGCAGGATGGCTCCTGTCACCCCGCGGCTCTCGGCCTAGTTGGGGCCCCACGGACCCCCGGCGTAGGTCGCGYAATTTGGGTAAGGTCATCRATACCCTCACGKGCGGCTTCGCCGACCTCATGGGGTACATTCCGCTCGTCGGCGSCCCCCTAGGGGGCGCTGCCAGGGTCCTGGCGCATGGCGTCCGGGTCCTGGAGGACGGCGTGAACTATGCAACAGGGAATTTSCCCGGTTGCTCTTTCTCTATCTTCCTCTTGGCTTTGCTGTCCTGTTTRRCCATCCCAGYTTCCGCC

>HM106556

ATGAGCACGAATCCTAAACCTCAAAGAAAAACCAAACGTAACACCAACCGCCGCCCACAGGACGTCAAGTTCCCGGGTGGTGGTCAGATCGTTGGTGGAGTTTACCTGTTGCCGCGCAGGGGCCCCAGGTTGGGTGTGCGCGCGACTAGGAAGACTTCCGAGCGGTCGCAACCTCGTGGAAGGCGACAACCTATCCCCAAGGCTCGCCGGCCCGAGGGCAGGACCTGGGCTCAGCCCGGGTACCCTTGGCCCCTCTATGGCAATGAGGGCTTGGGGTGGGCAGGATGGCTCCTGTCACCCCGCGGCTCTCGGCCTAGTTGGGGCCCCACGGACCCCCGGCGTAGGTCGCGTAATTTGGGTAAGGTCATCGATACCCTCACATGCGGCTTCGCCGACCTCATGGGGTACATTCCGCTCGTCGGCGCCCCCCTAGGGGGCGCTGCCAGGGCCCTGGCGCATGGCGTCCGGGTCCTGGAGGACGGCGTGAACTATGCAACAGGGAATTTGCCCGGTTGCTCTTTCTCTATCTTCCTCTTGGCTTTGCTGTCCTGTTTGACCATCCCAGCTTCCGCC

>HM106557

ATGAGCACGAATCCTAAACCTCAAAGAAAAACCAAACGTAACACCAACCGCCGCCCACAGGACGTCAAGTTCCCGGGTGGTGGTCAGATCGTTGGTGGAGTTTACCTGTTGCCGCGCAGGGGCCCCAGGTTGGGTGTGCGCGCGACTAGGAAGACTTCCGAGCGGTCGCAACCTCGTGGAAGGCGACAACCTATCCCCAAGGCTCGTCGGCCCGAGGGCAGGACCTGGGCTCAGCCCGGGTACCCTTGGCCCCTCTATGGCAATGAGGGCATGGGGTGGGCAGGATGGCTCCTGTCACCCCGCGGCTCTCGGCCTAGTTGGGGCCCCACGGACCCCCGGCGYAGGTCGCGTAATTTGGGTAAGGTCATCGATACCCTYACATGCGGCTTCGCCGATCTCATGGGGTACATTCCGCTCGTCGGCGCCCCCTTAGGGGGCGCTGCCAGGGCCCTGGCGCATGGCGTCCGGGTCCTGGAGGACGGCGTGAACTATGCAACAGGGAATTTACCCGGTTGCTCTTTCTCTATCTTCCTCTTGGCTTTGCTGTCCTGTTTGACCATCCCAGCTTCCGCT

>HM106558

ATGAGCACGAATCCTAAACCTCAAAGAAAAACCAAACGTAACAYCAACCGCCGCCCACAGGACGTCAAGTTCCCGGGTGGTGGTCAGATCGTTGGTGGAGTTTACCTGTTGCCGCGCAGGGGCCCCAGGTTGGGTGTGCGCGCGACTAGGAAGACTTCCGAGCGGTCGCAACCTCGTGGAAGGCGACAACCTATCCCCAAGGCTCGCCGGCCCGAGGGCAGGACCTGGGCYCAGCCCGGGTACCCTTGGCCCCTCTATGGCAATGAGGGCATGGGGTGGGCAGGATGGCTCCTGTCACCCCGCGGCTCTCGGCCTAGTTGGGGCCCCACGGACCCCCGGCGTAGGTCGCGTAATTTGGGTAAGGTCATCGATACCCTCACATGCGGCTTCGCCGACCTCATGGGGTACRTTCCGCTCGTCGGCGCCCCCCTAGGGGGYGYTGCCAKGGCCCTGGCGCATGGCGTCCGGGTCCTGGAGGACGGCGTGAACTATGCAACAGGGAATTTGCCCGGTTGCTCTTTCTCTATCTTCCTCTTGGCTTTGCTGTCCTGTTTGACCATYCCAGYTTCCGCT

>HM106559

ATGAGCACGAATCCTAAACCTCAAAGAAAAACCAAACGTAACACCAACCGCCGCCCACAGGACGTCAAGTTCCCGGGTGGTGGTCAGATCGTTGGTGGAGTATACCTGTTGCCGCGCAGGGGCCCCAGGTTGGGTGTGCGCGCGACTAGGAAGACTTCCGAGCGGTCGCAACCTCGTGGAAGGCGACAACCTATCCCCAAGGCTCGCCGGCCCGAGGGTAGGACCTGGGCTCAGCCCGGGTACCCTTGGCCCCTCTATGGCAATGAGGGCATGGGGTGGGCAGGATGGCTCCTGTCACCCCGCGGCTCTCGRCCTAGTTGGGGCCCCACGGACCCCCGGCGTAGGTCGCGTAATTTGGGTAAGGTCATCGATACCCTTACATGCGGCTTCGCCGACCTCATGGGGTACATTCCGCTCGTCGGCGCCCCCCTAGGGGGCGCTGCCAGGGCCCTGGCGCATGGCGTCCGGGTCCTGGAGGACGGCGTGAACTATGCAACAGGGAATTTACCCGGTTGCTCYTTCTCTATCTTCCTCTTGGCTTTGCTGTCCTGTTTGACCATCCCAGCTTCCGCT

>HM106560

ATGAGCACGAATCCTAAACCTCAAAGAAAAACCAAACGTAACACCAACCGCCGCCCACAKGACGTTAAGTTCCCGGGTGGTGGTCAGATCGTTGGTGGAGTTTACCTGTTGCCGCGCAGGGGCCCCAGGTTGGGTGTGCGCGCGACTAGGAAGACTTCCGAGCGGTCGCAACCTCGTGGAAGGCGACAACCTATCCCCAAGGCTCGCCGGCCCGAGGGCAGGACCTGGGCCCAGCCCGGGTACCCTTGGCCCCTCTATGGCAATGAGGGCTTRGGGTGGGCAGGATGGCTCCTGTCACCCCGCGGCTCTCGGCCTAGTTGGGGCCCYACGGACCCCCGGCGTAGGTCGCGTAATTTGGGTAAGGTCATCGATACCCTCACATGCGGCTTCGCCGACCTCATGGGGTACATTCCGCTCGTCGGCGCCCCCCTAGGGGGCGCYGCCAGRGCCCTGGCGCATGGCGTCCGGGTCCTGGAGGACGGCGTGAACTATGCAACAGGGAACTTGCCCGGTTGCTCTTTCTCTATCTTCCTCTTGGCTTTGCTGTCCTGTTTGACCATCCCAGTTTCCGCC

>HM106561

ATGAGCACGAATCCTAAACCTCAAAGAAAAACCAAACGTAACACCAACCGCCGCCCACAGGACGTCAAGTTCCCGGGTGGTGGTCAGATCGTTGGTGGAGTTTACCTGTTGCCGCGCAGGGGCCCCAGGTTGGGTGTGCGCGCGACTAGGAAGACTTCCGAGCGGTCGCAACCTCGTGGACGGCGACAACCTATCCCCAAGGCTCGCCGGCCCGAGGGCAGGACCTGGGCTCAGCCCGGGTACCCTTGGCCCCTCTATGGCAATGAGGGCATGGGGTGGGCAGGATGGCTCCTGTCACCCCGCGGCTCTCGGCCTAGTTGGGGCCCCACGGACCCCCGGCGTAGGTCGCGCAATTTGGGTAAAGTCATCGATACCCTCACGTGCGGCTTCGCCGACCTCATGGGGTACATTCCGCTCGTCGGCGCCCCCCTAGGGGGCGCTGCCAGGGCCCTGGCGCACGGCGTCCGGGTCCTGGAGGACGGCGTGAACTATGCAACAGGGAATCTGCCCGGTTGCTCTTTCTCTATCTTCTTCTTGGCTTTGCTGTCCTGTTTGACCATCCCAGCTTCCGCT

>HM106562

ATGAGCACGAATCCTAAACCTCAAAGAAAAACCAAACGTAACACCAACCGCCGCCCACAGGACGTCAAGTTCCCGGGTGGTGGTCAGATCGTTGGTGGAGTTTACCTGTTGCCGCGCAGGGGCCCCAGGTTGGGTGTGCGCGCGACTAGGAAGACTTCCGAGCGGTCGCAACCTCGTGGAAGGCGACAACCTATCCCCAAGGCTCGCCGGCCCGAGGGCAGGACCTGGGCTCAGCCCGGGTACCCTTGGCCCCTCTATGGCAATGAGGGCATGGGGTGGGCAGGATGGCTCCTGTCACCCCGCGGCTCTCGGCCTAGTTGGGGCCCCACGGACCCCCGGCGTAGGTCGCGTAACTTGGGTAAGGTCATCGATACCCTCACATGCGGCTTCGCCGACCTCATGGGGTACATTCCGCTCGTCGGCGCCCCCCTAGGGGGCGCTGCCAGGGCCTTGGCGCATGGCGTCCGGGTCCTGGAGGACGGCGTGAACTATGCAACAGGGAATTTGCCCGGTTGCTCTTTCTCTATCTTCCTCTTGGCTTTGCTGTCCTGTTTGACCATCCCAGCCTCCGCT

>HM106563

ATGAGCACGAATCCTAAACCTCAAAGAAAAACCAAACGTAACACCAACCGCCGCCCACAGGACGTCAAGTTCCCGGGTGGTGGTCAGATCGTTGGTGGAGTTTACCTGTTGCCGCGCAGGGGCCCCAGGTTGGGTGTGCGCGCGACTAGGAAGACTTCCGAGCGGTCGCAACCTCGTGGAAGGCGACAACCTATCCCCAAGGCTCGCCGGCCCGAGGGCAGGACCTGGGCTCAGCCCGGGTACCCTTGGCCCCTCTATGGCGATGAGGGCATGGGGTGGGCAGGATGGCTCCTGTCACCCCGCGGCTCTCGGCCTAGTTGGGGCCCCACGGACCCCCGGCGTAGGTCGCGTAATTTGGGTAAGGTCATCGATACCCTCACATGCGGCTTCGCCGACCTCATGGGGTACATTCCGCTCGTCGGCGCCCCCCTGGGGGGCGTTGCCAGGGCCCTGGCGCATGGCGTCCGGGTCCTGGAGGACGGCGTGAAYTATGCAACAGGGAATTTGCCCGGTTGCTCTTTCTCTATCTTCCTCTTGGCTTTGCTGTCCTGTTTGACCATCCCAGCTTCCGCT

>HM106564

ATGAGCACGAAACCTAAACCTCAAAGAAAAACCAAACGTAACACCAACCGCCGCCCACAGGACGTCAAGTTCCCGGGTGGTGGTCAGATCGTTGGTGGAGTTTACCTGTTGCCGCGCAGGGGCCCCAGGTTGGGTGTGCGCGCGACTAGGAAGACTTCCGAGCGGTCGCAACCTCGTGGAAGGCGACAACCTATCCCCAAGGCTCGCCGGCCCGAGGGCAGGACCTGGGCTCAGCCCGGGTACCCTTGGCCCCTCTATGGCAATGAGGGCATGGGGTGGGCAGGATGGCTCCTGTCACCCCGCGGCTCTCGGCCTAGTTGGGGCCCCACGGACCCCCGGCGTAGGTCGCGTAATTTGGGTAAGGTCATCGATACCCTCACATGCGGCTTCGCCGATCTCATGGGGTACATTCCGCTCGTCGGCGCCCCCCTAGGGGGYGCTGCCAGGGCCCTGGCGCATGGCGTCCGGGTCCTGGAGGACGGCGTGAACTATGCAACAGGGAATTTGCCCGGTTGCTCTTTCTCTATCTTCCTCTTGGCTTTGYTGTCCTGTTTGACCATCCCAGCTTCCGCT

>HM106565

ATGAGCACGAATCCTAAACCTCAAAGAAAAACCAAACGTAACACCAACCGCCGCCCACAGGACGTCAAGTTCCCGGGTGGTGGTCAGATCGTTGGTGGAGTTTACCTGTTGCCGCGCAGGGGCCCCAGGTTGGGTGTGCGCGCGACTAGGAAGACTTCCGAGCGGTCGCAACCTCGTGGAAGGCGACAACCTATCCCCAAGGCTCGCCGGCCCGAGGGCAGGACCTGGGCTCAGCCCGGGTACCCTTGGCCCCTCTATGGCAATGAGGGCATGGGGTGGGCAGGATGGCTCCTGTCACCCCGCGGCTCTCGGCCTAGTTGGGGCCCCACGGACCCCCGGCGTAGGTCGCGTAATTTGGGTAAGGTCATCGATACCCTYACATGCGGCTTCGCCGAYCTCATGGGGTACATTCCGCTCGTCGGCGCCCCCCTAGGGGGCGCTGCCAGGGCCCTGGCGCATGGCGTCCGGGTYCTGGARGACGGCGTGAACTATGCAACAGGGAATTTGCCCGGTTGCTCTTTCTCTATCTTCCTCTTGGCTTTGCTGTCCTGTTTGACCATCCCAGCTTCCGCT

>HM106566

ATGAGCACGAATCCTAAACCTCAAAGAAAAACCAAACGTAACACCAACCGCCGCCCACAGGACGTCAAGTTCCCGGGYGGTGGTCAGATCGTTGGTGGAGTTTACCTGTTGCCGCGCAGGGGCCCCAGRYTGGGTGTGCGCGCGACTAGGAAGACTTCCGAGCGGTCGCAACCTCGTGGAAGGCGACAACCTRTCCCCAAGGCTCGCCGGCCCGAGGGCAGGACCTGGGCTCAGCCCGGGTACCCTTGGCCCCTCTATGGCAATGAGGGCATGGGGTGGGCAGGATGGCTCCTGTCACCCCGCGGCTCCCGGCCTAGTTGGGGCCCCACGGACCCCCGGCGTAGGTCGCGTAATTTGGGTAAGGTCATCGATACCCTCACATGCGGCTTCGCCGACCTCATGGGGTACATYCCGCTCGTCGGCGCCCCCYTAGGGGGYGYTGCCAGGGCCCTGGCGCATGGCGTCCGGGTCCTGGAGGACGGCGTGAACTATGCAACAGGRAATWTGCCCGGTTGCTCTTTCTCTATCTTCCTCTTGGCTTTGCTGTCCTGYTTGACCATCCCAGCYTCCGCY

>HM106567

ATGAGCACGAATCCTAAACCTCAAAGAAAAACCAAACGTAACACCAACCGCCGCCCACAGGACGTCAAGTTCCCGGGTGGTGGTCAGATCGTTGGTGGAGTTTACCTGTTGCCGCGCAGGGGCCCCAGGTTGGGTGTGCGCGCGACTAGGAAGACTTCCGAGCGGTCGCAACCTCGTGGRAGGCGACAACCTATCCCCAAGGCTCGCCGRCCCGAGGGCAGGACCTGGGCTCAGCCCGGGTACCCTTGGCCCCTCTATGGCAATGAGGGCTTGGGGTGGGCAGGATGGCTCCTGTCACCCCGCGGCTCTCGGCCTAGTTGGGGCCCCACGGACCCCCGGCGTAGGTCGCGTAATTTGGGTAAGGTCATCGATACYCTCACATGCGGCTTCGCCGACCTCATGGGGTACATYCCGCTCGTCGGCGCCCCCCTRGGGGGCGTTGCCAGGGCCCTGGCGCATGGCGTCCGGGTCCTGGAGGACGGCGTGAACTATGCAACAGGGAATTTGCCCGGTTGCTCTTTCTCTATCTTCCTCTTGGCTYTGCTGTCCTGTTTGACCATCCCGGCTTCCGCT

>HM106568

ATGAGCACGAATCCTAAACCTCAAAGAAAAACCAAACGTAACACCAACCGCCGCCCACAGGACGTCAAGTTCCCGGGTGGTGGTCAGATCGTTGGTGGAGTTTACCTGTTGCCGCGCAGGGGCCCCAGGTTGGGTGTGCGCGCGACTAGGAAGACTTCCGAGCGGTCGCAACCTCGTGGAAGGCGACAACCTATCCCCAAGGCTCGCCGGCCCGAGGGCAGGACCTGGGCTCAGCCCGGGTACCCTTGGCCCCTCTATGGCAATGAGGGCATGGGGTGGGCAGGATGGCTCCTGTCACCTCACGGCTCCCGGCCTAGTTGGGGCCCCACGGACCCCCGGCGTAGGTCGCGTAATTTGGGTAAGGTCATCGATACCCTCACATGCGGCTTCGCCGACCTCATGGGGTACATTCCGCTCGTCGGCGCCCCCCTAGGGGGCGTTGCCAGGGCCTTGGCGCATGGCGTCCGGGTCCTGGAAGACGGCGTGAACTATGCAACAGGGAATTTGCCCGGTTGCTCTTTCTCTATCTTCCTCTTGGCTTTGCTGTCCTGTTTGACCGTCCCAGCTTCCGCT
