# Supplementary material for: Effect of Immune Pressure on Hepatitis C Virus Evolution: Insights From a Single-Source Outbreak
Source: Hepatology. 2011 Feb;53(2):396–405. doi: 10.1002/hep.24076 (PMC3044208; doi:10.1002/hep.24076)
Supplement: Supplementary file 3 [file hep0053-0396-SD3.doc]

>HM106569

ATCGAAGTGCGCAACGTGTCCGGRGTGTACCATGTCACGAACGACTGCTCCAACGCAAGCATTGTGTATGAGGCAGCGGACATGATTATGCATAYCCCYGGGTGYGTGCCCTGCGTTCRGGAGRRCAACTYCTCCCGCTGCTGGGTAGCGCTCACYCCCACGCTCGCGGCCAGGAACTCCAGCATCCCCACTACGACAATACGACGTCACATCGATTTGCTCGTTGGGACCGCTGCTTTCTGCTCCGCTATGTACGTRGGAGATCTTTGCGGATCTGTTTTCCTTGTCTCTCAGCTGTTCACCTTCTCGCCTCGCCGGCATRTGACARTRCAGGACTGCAATTGTTCAATCTATCCCGGCCAYGTATCRGGTCACCGCATGGCTTGGGACATGATGATGAACTGGTCACCTACAACAGCTCTAGTGGTGTCGCAGTTACTCCGGATCCCACAAGCYATCTTGGAYGTGGTGGCGGGAGCCCACTGGGGAGTCCTGGCGGGCCTCGCCTACTAYTCCATGGTGGGGAACTGGGCTAAGGTYTTGATTGTGATGCTACTTTTTGCCGGCGTCGACGGS

>HM106570

ATCGAAGTGCGCAACGTGTCCGGAGTGTACCATGTCACGAACGAYTGCTCCAACGCAAGCATTGTGTATGAGGCAACGGACATGATTATGCATACCCCCGGATGCGTGCCCTGCGTTCGGGAGAACAACWTCTCCCGCTGCTGGGTAGCGCTCACTCCYACGCTCGCGGCCAGGAACTCCAGCATCCCCACTACGACAATACGACGCCACGTCGATTTGCTCGTTGGGGCGGCTGCTTTCTGCTCCGCTATGTACGTGGGAGATCTTTGCGGATCTGTTTTCCTTGTCTCTCAGCTGTTCACCTTCTCGCCTCGCCGGCATGAGACAGTACAGGACTGCAATTGTTCAATCTATCCCGGCCACGTATCRGGTCACCGCATGGCTTGGGACATGATGATGAACTGGTCACCTACAACAGCTCTAGTGGTGTCGCAGTTACTCCGGATCCCACAAGCTGTCGTGGACATGGTGGCGGGAGCCCACTGGGGAGTCCTGGCGGGCCTCGCCTACTATTCCATGGTGGGGAACTGGGCTAAGGTCTTGATTGTGATGCTACTTTTTGCCGGCGTCGACGGG

>HM106571

ATCGAAGTGCGCAACGTGTCCGGAGTGTACCATGTCACGAACGACTGCTCYAACGCAAGCATTGTGTATGAGGCAGCGGACATGATTATGCATACCCCCGGGTGCGTGCCCTGCGTTCGGGAGAACAACTCCTCCCGCTGCTGGGTAGCGCTCACTCCCACGCTCGCGGCCAGGAACTACAGCATCCCCACTGCGACAATACGACGTCACGTCGATTTGCTCGTTGGGACGGCTGCTTTCTGCTCTGCTATGTACGTGGGAGATCTCTGCGGATCCGTTTTTCTCGTCTCTCAGCTGTTTACTTTCTCGCCTCGCCGGCATGAGACAGTACAGGACTGCAATTGCTCAATCTATCCCGGCCACGTATCGGGTCACCGCATGGCTTGGGACATGATGATGAACTGGTCACCTACAACAGCTCTAGTGGTATCGCAGTTACTCCGGATCCCACAAGCTGTCGTGGACGTGGTGGCGGGAGCCCACTGGGGAGTCCTGGCGGGCCTCGCCTACTACTCTATGGCAGGGAACTGGGCTAAGGTCTTGATTGTGATGCTGCTTTTYGCCGGCGTCGACGGA

>HM106572

ATCGAAGTGCGCAACGTGTCCGGAGTGTACCATGTCACGAACGACTGCTCCAACGCAAGCATTGTGTATGAGGCAGCGGACATGATTATGCATACCCCCGGGTGCGTGCCCTGCGTTCGGGAGRACAACTCCTCCCGCTGCTGGGTAGCGCTCACTCCCACGCTCGCGGCCAGGAACKCCAGCATCCCCACTACGACAATACGACGTCACGTCGATTTGCTCGTTGGGAYAGCTGCYTTCTGCTCCGCTATGTACGTGGGAGAYCTCTGCGGATCTGTWYTCCTYGTCTCTCARCTGTTCACCTTCTCGCCTCGCYKGCAYGAGACAGTACAGGACTGCAATTGCTCAATCTATCCCGGCCACGTRTCGGGYCACCGCATGGCTTGGGATATGATGATGAACTGGTCACCYACAACAGCTCTAGTGGTATCGCAGYTACTCCGGATCCCACAAGCTGTCGTGGAYRTGGTRGCGGGAGCCCACTGGGGAGTCCTGGYGGGCMTCGCYTACTATTCYATGGCAGGGAACTGGGCTAAGGTCTTGATTGTGMTGCTGCTTTTTKCCGGCGTCGACGGG

>HM106573

ATYGAAGTGCGCAACGTGTCCGGAGTGTACCATGTCACGAACGACTGCTCCAACGCAAGCATTGTGTATGAGGCAGCGGACATGATYATGCAYACCCCCGGGTGCGTGCCCTGCGTTCGGGAGAACAACTCCTCCCGCTGCTGGGTAGCGCTCACTCCCACGCTCGCGGCCAGGAACTCCAGCATCCCCACTACGACAATACGACGYCACGTCGATYTGCTCGTTGGGGCGGCTGCTTTCTGCTCCGCTATGTACGTGGGAGAYCTTTGCGGATCTGTTTTCCTTGTCTCTCAGCTKTTCACCTTYTCGCCTCGYCGGCATGAGACAGTGCARGACTGCAAYTGCTCAATCTATCCCGGCCAYCTATCGGGTCACCGCATGGCTTGGGACATGATGATGAACTGGTCACCTACAACAGCTCTRGTGGTGTCGCAGTTACTCCGGATCCCACAAGCYRTCGTGGACATGGTGGCGGGAGCCCACTGGGGAGTCCTGGCGGGCCTCGCCTACTATTCCATGGTGGGGAACTGGGCTAAGGTCTTGATTGTGATGCTACTTTTTGCCGGCGTCGACGGG

>HM106574

ATCGAAGTGCGCAACGTGTCCGGAGTGTACCATGTCACCAACGACTGCTCCAACGCAAGCATTGTGTATGAGGCAGAGGACATGATTATGCACACCCCCGGGTGCGTGCCCTGCGTTCGGGAGAACAACTCCTCCCGTTGCTGGGTAGCGCTCACTCCCACGCTCGCGGCCAGGAACTCCAGCATCCCCACTACGACAATACGACGTCATGTCGATTTGCTCGTTGGGACGGCTGCTYTCTGCTCCGCTATGTAYGTGGGAGAYYTTTGCGGATCTGTTTTCCTTGTCTCTCAGCTGTTCACCTTCTCGCCTCGCCGGCATGAGACAGTACAGGACTGCAATTGCTCAATCTATCCCGGCCACATAACGGGTCACCGCATGGCTTGGGACATGATGATGAACTGGTCACCTACAACAGCTCTAGTGGTGTCGCAGTTACTCCGGATCCCACAAGCTGTCGTGGACATGGTGGCGGGAGCCCACTGGGGAGTCCTGGCGGGCCTCGCCTACTATTCCATGGTRGGGAACTGGGCTAAGGTCTTGATTGTGCTGCTACTTTTTGCCGGCGTCGACGGG

>HM106575

ATCGAAGTGCGCAACGTGTCCGGAGTGTACCATGTCACGAACGACTGCTCCAACGCAAGCATTGTGTATGARGCAGCGGACATGATTATGCATACCCCCGGGTGCGTGCCCTGCGTTCGGGAGAACAACTYCTCCCGCTGCTGGGTAGCGCTCACCCCCACGCTCGCGGCCAGGAACTCCAGCATCCCCACTACGACAATACGACGTCACGTCGATTTGCTCGTTGGGACAGCTGCTTTCTGCTCCGCTATGTACGTGGGAGATCTCTGCGGATCTGTTTTCCTCGTCTCTCAGCTGTTCACCTTCTCGCCTCGCCGGCATGAGACAGTGCAGGACTGCAATTGCTCAATCTATCCCGGCCATGTATCGGGTCACCGCATGGCTTGGGACATGATGATGAACTGGTCACCTACAACAGCTCTAGTGGTATCGCAGTTACTCCGGATCCCACAAGCTGTCGTGGATATGGTGGCGGGAGCCCACTGGGGAGTCCTGGCGGGCCTCGCCTACTATTCTATGGCAGGGAACTGGGCTAAGGTCTTGATTGTGATGCTGCTTTTTGCCGGCGTCGACGGG

>HM106576

ATCGAAGTGCGCAACGTGTCCGGAGGGTACCATGTCACGAACGACTGCTCCAACGCAAGCATTGTGTATGAGGCAGCGGACATGATTATGCATACCCCCGGGTGCGTGCCCTGCGTTCGGGAGGAYAATTCCTCCCGCTGCTGGGTAGCGCTCACTCCCACGCTCGCGGCCAGGAACTCCAGCATCCCCACTACGACAATACGACGTCACGTCGATTTGCTCGTTGGGGTGGCCGCTTTCTGTTCTGCTATGTACGTGGGAGATCTCTGCGGATCTGTTTTCCTTGTCTCTCAGCTGTTCACCTTCTCGCCTCGCCGGCATGAGACAGTACAGGACTGCAATTGCTCAATCTATCCCGGCCACGTATCRGGTCACCGCATGGCTTGGGATATGATGATGAACTGGTCACCTACAACAGCTCTAGTGGTGTCGCAGTTACTCCGGATCCCACAAGCTGTCGTGGACATAGTGGCGGGAGCCCACTGGGGAATCCTGGCGGGCCTCGCCTACTATTCCATGGTGGGGAACTGGGCTAAGGTCTTGATTGTGATGCTACTTTTTGCCGGCGTCGACGGG

>HM106577

ATCGAAGTGCGCAACGTGTCCGGAGTGTACCATGTCACGAACGACTGCTCCAACSMAAGCATTGTGTATGAGGCAGCGGACATGATYATGCATACCCCCGGKTGCGTGCCCTGCGTTCGGGAGAACAACTCCTCCCGCTGCTGGGTAGCGCTCACTCCCACGCTCGCGGCCAGGAACTCCAGCAYCCCCACYACGACAATACGACGTCACGTCGATTTGCTCGTTGGGACAGCTGCTTTCTGCTCCGCTATGTACGTGGGAGATCTCTGCGGATCTGTTTTCCTCGTCTCCCAGCTGTTCACCTTCTCGCCTCGCCGGCATGAGACGGTACAGGACTGCAATTGCTCAATCTATCCCGGCCACGTATCGGGTCACCGCATGGCTTGGGACATGATGATGAACTGGTCACCTACAACAGCTCTAGTGGTATCGCAGTTACTCCGGATCCCACAAGCTGTCGTGGACATGGTGGCGGGAGCCCACTGGGGAGTCCTGGCGGGCCTCGCCTACTATTCCATGGCAGGGAACTGGGCTAAGGTCTTGATTGTGATGCTGCTTTTTGCCGGCGTCGACGGG

>HM106578

ATCGAGGTGCGCAACGTGTCCGGAGTGTACCATGTCACGAACGACTGCTCCAACGCAAGCATTGTGTATGAGGCAGCGGACATGATTATGCATACCCCCGGGTGCGTGCCCTGCGTTCGGGARAACAACTCCTCCCGCTGCTGGGTAGCGCTCACTCCCACGCTCGCGGCCAGGAATTTCAGCATCCCCACTACGACAATACGRCGTCACGTCGACTTGCTCGTTGGGGCGGCTGCTTTCTGTTCCGCTATGTACGTGGGAGATCTTTGCGGATCTGTTTTCCTTGTCTCTCAGCTGTTCACCTTTTCGCCTCGCCGGCATGAGACAGTACAGGACTGCAATTGCTCAATCTATCCCGGCCACGTATCAGGTCACCGCATGGCTTGGGACATGATGATGAACTGGTCACCTACAACAGCTCTAGTGGTGTCGCAGTTACTCCGGATCCCACAAGCTCTCTTGGACATGGTGGCGGGAGCCCACTGGGGAGTCCTGGCGGGCCTCGCCTACTATTCCATGGTGGGGAACTGGGCTAAGGTCTTGATTGTGATGCTACTATTTGCCGGCGTCGACGGC

>HM106579

ATCGAAGTGCGCAATGCGTCCGGAGTGTACCACGTCACGAACGACTGCTCCAACGCAAGTATTGTGTATGAGGCAGCGGACATGATTATGCAYACCCCCGGGTGCGTGCCCTGCGTTCGGGAGGAGAACTCCTCCCGCTGCTGGGTAGCGCTCACTCCCACGCTCGCGGCCAGGAACTCCAGCATCCCCACTACGACMATACGACGCCACGTCGATTTGCTCGTTGGGACGGCTGCTTTCTGTTCCGCTATGTACGTGGGAGATCTTTGCGGATCTGTTTTCCTTGTCTCTCAGCTGTTCACCTTCTCGCCTCGCCGGCATGAGACAGTACAGGACTGCAATTGTTCAATCTATCCCGGCCACGTATCGGGTCACCGCATGGCTTGGGACATGATGATGAACTGGTCACCTACAACAGCTCTAGTAGTGTCGCAGTTRCTCCGGATCCCACAAGCCGTCGTGGACATGTTGGCGGGAGCCCACTGGGGAGTCCTGGCGGGCATCGCCTACTATTCCATGGTGGGGAACTGGGCTAAGGTCTTGATTGTGATGCTACTTTTTGCCGGCGTCGACGGG

>HM106580

ATCGAAGTGCGCAACGTGTCCGGGGTGTACCATGTCACGAACGACTGCTCCAACGCAAGCATTGTGTATGAGGCAGCGGACATGATTATGCACACCCCCGGGTGCGTGCCCTGTGTTCGGGAGAACAACTCCTCCCGCTGCTGGGTAGCGCTCACTCCCACGCTCGCAGCCAGGAATTCCAGCATCCCCACTACGACAATACGACGTCACGTCGATTTGCTCGTTGGGACGGCTGCTTTCTGCTCCGCTATGTACGTGGGAGATCTTTGCGGATCTGTTTTCCTTGTCTCTCAGCTGTTCACCTTCTCGCCTCGCCGGCATGAGACAGTGCAGGACTGCAATTGCTCAATCTATTCCGGCCACGTATCGGGTCACCGCATGGCTTGGGACATGATGATGAATTGGTCACCTACAACAGCTCTAGTGGTGTCGCAGTTACTCCGGATCCCACAAGCTATCTTGGACGTAGTGGCGGGAGCCCACTGGGGAGTCCTGGCGGGCCTCGCCTACTATTCCATGGTGGGGAACTGGGCTAAGGTCTTGATTGTGATGCTACTTTTTGCCGGCGTCGACGGG

>HM106581

ATCGAAGTGCGCAACGTGTCCGGAGTGTACCATGTCACGAACGACTGCTCCAACGCAAGCATTGTGTATGAGGCGGACGACGTGATTTTGCAYACCCCCGGGTGCGTGCCCTGCGTTCGGGAGAACAACCTCTCCCGCTGCTGGGTAGCGCTCACTCCCACGCTCGCGGCCAGGAACTCCAGCATCCCCACYACGACAATACGACGACACGTCGATTTGCTCGTTGGGRCAGCTGCTTTCTGCTCCGCTATGTACGTGGGAGATCTTTGCGGATCTGTTTTCCTTGTCTCTCAGCTGTTCACCTTCTCRCCTCGCCGGCATYGGACAGAACAGGACTGCAATTGCTCAATCTATCCCGGCCACGTATCGGGTCACCGCATGGCTTGGGACATGATGATGAACTGGTCACCWACAACAGCTCTRGTRGTGTCGCAGTTACTCCGGATCCCACAAGCTGTCGTGGACATGGTGGCGGGAGCCCACTGGGGAGTCCTGGCGGGCCTCGCCTACTATTCCATGGTGGGGAACTGGGCTAAGGTCTTGATTGTGATGCTACTTTTTGCCGGCGTTGACGGG

>HM106582

ATCGAAGTGCGCAACGTGTCCGGAGTGTACCATGTCACGAACGACTGCTCCAACGCAAGCATTGTGTATGAGGCAGCGGACATGATTATGCATACCCCCGGGTGCGTGCCCTGCGTTCGGGAGAACAACTCCTCCCGCTGCTGGGTAGCGCTCACTCCCACGCTCGCGGCCAGGAACTCCAGCATCCCCACTACGACAATACGACGTCACGTCGATTTGCTCGTTGGGACGGCTGCTTTCTGCTCCGCTATGTACGTGGGAGATCTTTGCGGATCTGTTTTCCTTGTCTCTCAACTGTTCACCTTCTCGCCTCGCCGGCAYGAGACAGCACAGGACTGCAATTGCTCAATCTATCCCGGCCACGTATCTGGTCATCGCATGGCTTGGGAYATGATGATGAACTGGTCACCTACAACAGCTCTAGTGGTGTCGCAGTTGCTCCGGATCCCACAAGCTGTCGTGGACATGGTGGCGGGAGCCCACTGGGGAGTCCTGGCGGGCCTCGCCTACTATTCCATGGTRGGGAACTGGGCTAAGGTCTTGGTTGTGCTGCTACTTTTTGCCGGCGTCGACGGG

>HM106583

ATYGAAGTGCGCAACGTGTCCGGAGTGTACCATGTCACGAACGACTGCTCCAACKCAAGCATTGTGTATGAGGCAGCGGACGTGATYATGCATACCCCCGGGTGCGTGCCCTGCGTTCGGGAGGASCCGTGAAACCCCAACTCCTCCCGCTGCTGGGTAGCGCTCACCCCCACGCTCGCGGCCAGGAACTCCAGCATYCCCACTACGACAATACGACGTCACGTCGATYTGCTCGTTGGGACGGCTGCTTTCTGCTCCGCCATGTACGTGGGGGATCTTTGCGGATCTGTTTTCCTTGTCTCTCAGCTGTTCACCTTCTCGCCTCGCCGGCATGAGACAGTACAAGACTGCAATTGCTCAATCTATCCCGGCCACGTATCGGGTCACCGCATGGCTTGGGACATGATGATGAACTGGTCACCTACAACAGCTCTAGTGRTGTCGCAGTTACTCCGGATCCCACAAGCCGTCGTGGACATGGTGGCGGGAGCCCACTGGGGAGTCCTGGCGGGCCTCGCCTACTATTCCATGGTGGGGAACTGGGCTAAGGTCTTAATTGTGATGCTACTTTTTGCCGGCGTCGACGG

>HM106584

ATCGAAGTGCGCAACGTGTCCGGRGTGTACCATGTCACGAACGACTGCTCCAACGCAAGCATTGTGTATGAGGCAGCGGACATGATTATGCAYAYCCCCGGGTGCGTGCCCTGCGTTCGGGAGGAGAACTCCTCCCGCTGCTGGGTAGCGCTCACTCCCACGCTCGCGGCCAGGAACTCCAGCATYCCCACYACGACAATACGACGTCATGTCGATTTGCTYGTTGGGACGGCCGCYTTCTGCTCCGCTATGTACGTGGGAGATCTTTGCGGATCTGTTTTYCTTCTCTCTCAGCTGTTCACMTTCTCGCCTCGCCGGCATGAGACAGTGCAGGACTGCAATTGCTCAATCTATCCCGGCCACGTATCGGGTCACCGCATGGCTTGGGACATGATGATGAACTGGTCACCTACAACAGCTCTAGTRGTGTCGCAGTTACTCCGGATCCCACAAGCTGTCGTGGACATAGTGGCGGGAGCCCACTGGGGAGTCCTGGCGGGCCTCGCCTACTATTCCATGGTGGGGAACTGGGCTAAGGTYTTGATTGTGATGCTRCTTTTTGCYGGCGTCGACGGR

>HM106585

TTCGAAGTGCGCAACGTGTCCGGGGTGTACCATGTCACGAACGACTGCTCCAACGCAAGCATTGTGTATGAGGCAGAGGACATGATTATGCATACCCCCGGGTGCGTGCCCTGCGTTCGGGAGGACAACTCCTCCCGCTGCTGGGTAGCGCTCACTCCCACGCTCGCGGCCAGGAACTCCAGCGTCCCCACCACGACAATACGACGTCACGTCGATTTGCTCGTTGGGACGGCTGCTCTCTGCTCCGCTATGTACGTGGGAGATCTTTGCGGATCTGTTTTCCTTGTCTCTCAGCTGTTCACCTTCTCGCCTCGCCGGCATGAGACAGTACAGGACTGCAATTGCTCAATCTATCCCGGCCACGTATCGGGGCACCGCATGGCTTGGGACATGATGATGAACTGGTCACCTACAACAGCTCTAGTGGTGTCGCAGTTACTCCGGATCCCACAAGCTATCTTGGACGTGGTGGCGGGAGCCCACTGGGGAGTCCTGGCGGGCCTCGCCTACTACTCCATGGTGGGGAACTGGGCTAAGGTCTTGATTGTGATGCTACTTTTTGCCGGCGTCGACGGG

>HM106586

ATCGAAGTGCGCAACGTGTCCGGAGTGTAYCATGTCACGAACGACTGCTCCAACGCAAGCATTGTGTATGAGGCAGCGGACATGATYATGCATACCCCCGGGTGCGTGCCCTGCGTTCGGGAGRRCAACTCCTCCCRCTGCTGGGTAGCGCTCACTCCCACGCTCGCGGCCAGGAACTCYAGCATCCCCACYACGACAATACGGCGTCACGTCGATTTGCTCGTTGGGACGGCTGCTTTCTGTTCCGCTATGTACGTGGGAGATCTTTGCGGATCTGTTTTCCTTGTCTCTCAGCTGTTCACYTTCTCGCCGCGTCAGCATRCGACRGTMCARGACTGCAATTGCTCAATCTATCCCGGCCATGTATCGGGTCACCGCATGGCTYGGGACATGATGATGAACTGGTCACCTACAACAGCTCTAGTGGTGTCGCAGTTGCTCCGGATCCCACAAGCYGTCGTGGACGTGGTGGCGGGAGCCCACTGGGGAGTCCTGGCGGGCCTCGYCTACTATTCCATGGTGGGGAACTGGGCTAAGGTTYTGGTTGTGATGCTACTTTTTGCCGGCGTCGACGGG

>HM106587

ATCGAGGTGCGCAACGTGTCCGGAGTGTACCATGTCACGAACGACTGCTCCAACGCAAGCATTGTGTATGAGGCAGCGGACATGATTATGCATACCCCCGGGTGCGTGCCCTGCGTTCGGGAGTACAACTCCTCCCGCTGCTGGGTAGCGCTCACTCCCACGCTCGCGGCCAGGAACTCCAGCATCCCYACTACGACAATACGACGACACGTCGATTTGCTCGTTGGGGCGGCTGCTTTCTGCTCCGCTATGTACGTGGGAGATCTTTGCGGATCTGTTTTCCTTGTCTCTCAGCTGTTCACCTTCTCGCCTCGCCGGCACGAGACAGTACAGGACTGCAATTGCTCAATCTATCCCGGCCACGTATCRGGTCACCGCATGGCTTGGGACATGATGATGAACTGGTCACCTACAACAGCTCTAGTGGTGTCGCAGYTACTCCGGATCCCACAAGCTGTCGTGGATATGGTGGCGGGAGCCCACTGGGGAGTCCTGGCGGGCCTCGCCTACTATTCCATGGCAGGGAACTGGGCTAAGGTCTTGGTTGTGATGCTRCTYTTTGCCGGCGTCGACGGG

>HM106588

ATCGAAGTGCGCAACGTGTCCGGARTRTAYCATGTCACGAACGACTGCTCCAACGCAAGCATTGTGTATGAGGCAGCGGACATGATTATGCATATTCCCGGGTGCGTGCCCTGCGTTCGGGAGGACAACTCCTCCCGCTGCTGGGTAGCGCTCACTCCCACGCTCGCGGCCAGGAACTCCAGCATCCCYACTACGACAATACGACGYCACGTCGATTTGCTCGTTGGGACGGCTGCTTTCTGTTCCGCCATGTACGTGGGAGATCTTTGCGGATCTGTTTTCCTTGTCTCTCAGTTGTTCACCTTCTCGCCTCGCCAGCACACGACAGTACAGGACTGCAATTGTTCAATCTATCCCGGCCACGTATCTGGCCACCGCATGGCTTGGGACATGATGATGAACTGGTCACCTACAGCAGCTCTAGTGGTATCGCAGTTACTCCGGATCCCACAAGCTATCTTGGACGTGGTGGCGGGAGCCCACTGGGGAGTCCTGGCGGGCCTCGCCTACTATTCCATGGTGGGGAACTGGGCTAAGGTCTTGGTTGTGATGCTACTTTTTGCCGGCGTCGACGGG

>HM106589

ATCGARGTGCGCAACGTGTCCGGAGTGTACCATGTCACGAACGACTGCTCCAACGCAAGCATTGTGTATGAGGCAGCGGACATGATTATGCACACCCCCGGATGYGTGCCCTGCGTTCGGGAGGACAACTCCTCCCGCTGCTGGGTAGCGCTCACTCCCACGCTCGCGGCCAGGAACTCCAGCATCCCCACTACGACAATACGACGTCACGTCGATTTGCTCGTTGGGGCGGCTGCTTTCTGCTCCGCTATGTACGTGGGAGAYCTTTGCGGATCTGTCTTCCTTRTCTCTCAGCTGTTCACCTTCTCGCCTCGCCGGCATGAGACAGTACAGGACTGCAATTGCTCAATCTATCCCGGCCACRTATCRGGCCACCGCATGGCTTGGGACATGATGATGAACTGGTCACCTACAACAGCTCTGGTGGTGTCGCAGYTACTCCGGATCCCACAAGCTGTCGTGGACATGGTGGCGGGAGCCCACTGGGGAGTCCTGGCGGGCCTCGCRTACTATTCCATGGTGGGGAACTGGGCTAAGGTYTTGRTTGTGATGCTACTTTTTGCCGGCGTCGACGGG

>HM106590

ATCGAAGTGCGCAACGTGTCCGGAGTGTACCATGTCACGAACGACTGCTCYAACGCAAGCATTGTGTATGAGGCAGCGGACATGATTATGCATACCCCCGGGTGCGTGCCCTGCGTTCGAGAGAGCAACTCCTCTCGCTGCTGGGTAGCGCTCACTCCCACGCTCGCGGCCAGGAATTCCAGCATCCCCACTACGACAATACGRCGYCACGTCGACTTGCTCGTTGGGGCGGCYGCTCTCTGCTCCGCTATGTACGTGGGRGATCTTTGCGGATCTGTTTTCCTTGTCTCTCAGCTGTTCACCTTCTCGCCTCGCCGGCATGAGACTGTACAGGACTGCAATTGCTCAATCTATCCCGGCCACGTATCGGGTCACCGCATGGCTTGGGATATGATGATGAACTGGTCACCTACAACAGCTCTAGTGGTGTCGCAGTTACTCCGGATCCCACAAGCTGTCGTGGACATGGTGGCGGGAGCCCACTGGGGAGTCCTGGCGGGCCTCGCTTACTATTCCATGGTGGGGAACTGGGCTAAGGTCTTGATTGTGATGCTACTTTTTGCCGGCGTCGACGGG

>HM106591

ATCGAAGTGCGCAACGTGTCCGGAGTGTACCATGTCACGAACGACTGCTCCAACGCAAGCATTGTGTATGAGGCAGCGGACATGATTATGCATACCCCCGGGTGCGTGCCCTGCGTTCGGGAGAACAACTCCTCCCGCTGCTGGGTAGCGCTCACTCCCACGCTCGCGGCCAGGAACTCCAGCATCCCCACYACGACAATACGGCGTCACGTCGATTTGCTCGTTGGGGCGGCTGCYTTCTGCTCCGCTATGTACGTGGGAGATCTTTGCGGATCTGTTTTCCTTGTCTCTCAGCTGTTCACCTTCTCGCCTCGCCGGCATGAGACAGTACAGGACTGCAATTGYTCAATCTATCCCGGCCAYGTRTCAGGTCACCGCATGGCTTGGGACATGATGATGAACTGGTCACCTACAACAGCTCTAGTGGTGTCGCAGTTACTCCGGATCCCACAAGCTGTCGTGGACATGGTGGCGGGAGCCCACTGGGGAGTCCTRGCGGGCCTCGCCTACTATTCCATGGTGGGGAACTGGGCTAAGGTCTTGATTGTGATGCTACTYTTTGCCGGCGTCGACGGG

>HM106592

ATCGARGTGCGCAACGTGTCCGGAGTGTACCATGTCACGAACGACTGCTCCAACGCAAGCATTGTGTATGAGGSGGCGGACATGATYATGCAYACCCCCGGGTGCGTGCCCTGCGTTCGGGAGGAYAACTCCTCCCGKTGCTGGGTAGCGCTCACYCCCACGCTCGCGGCCAGGAACTCCAGCATCCCYACTACGACAATACGRCGTCACATCGAYTTGCTYGTTGGGACGGCTGCTTTCTGCTCCGCTATGTACGTGGGAGATCTTTGCGGATCTGTTTTCCTTGTCTCTCAGCTGTTCACCTTCTCGCCTCGCCGGCATGAGACAGTACAGGACTGCAATTGCTCAATCTACCCCGGCCACGTATCGGGTCACCGCATGGCTTGGGATATGATGATGAACTGGTCACCTACAACAGCTCTAGTGGTGTCGCAGTTACTCCGGATCCCACAAGCTGTCGTGGATGTGGTGGCGGGAGCCCACTGGGGAGTCCTGGCGGGCCTCGCCTACTAYTCYATGGTGGGGAACTGGGCTAAGGTYTTAATTGTGATGCTACTYTTTGCCGGCGTCGACGGG

>HM106593

ATCGAAGTGCGCAACGTGTCCGGAATGTACCATGTCACGAACGACTGCTCCAACGCAAGCATTGTGTATGAGGCAGCGGACATGATTATGCACATCCCCGGGTGCGTGCCCTGCGTTCGGGAGGGCAACTCCTCCCGCTGCTGGGTAGCGCTCACTCCCACGCTCGCGGCCAGGAAYTCCAGCATCCCCACTACGACAATACGACGTCACGTCGACTTGCTCGTTGGGACGGCYGCTTTCTGCTCCGCTATGTACGTGGGAGATCTTTGCGGATCTGTTTTYCTTGTCTCTCAGCTGTTCACCTTCATGCCTCGCCGGCATCAGACAGTGCAGGACTGCAATTGCTCAATCTATCCCGGCCACGTAACGGGTCACCGCATGGCTTGGGACATGATGATGAACTGGTCACCTACAACAGCTCTAGTGGTGTCGCAGTTACTCCGGATCCCACAAGCTGTCTTGGACATGGTGGCGGGAGCCCACTGGGGAGTCCTGGCGGGCCTCGCCTACTATTCCATGGCGGGGAACTGGGCTAAGGTCTTGATTGTGATGYTACTTTTTGCCGGCGTCGACGGG

>HM106594

ATCGAAGTGCGCAACGTGTCCGGAGTGTACCATGTCACGAACGACTGCTCCAACGCAAGTATTGTGTATGAGGCAGCGGACATGATTATGCATACCCCCGGGTGCGTGCCCTGCGTTCGGGAGAACAACTCCTCCCGCTGCTGGGTAGCGCTCACTCCYACGCTCGCGGCCAGGAACTCCAGCRTCCCCACTACGACAATACGACRTCACGTCGATTTGCTCGTTGGGRCGGCYGCTTTCTGCTCCGCTATGTACGTGGGAGAYCTTTGCGGATCTGTTTTCCTTGTCTCTCAGCTGTTCACCTTYTCGCCTCGCCGGCATGAGACAGTACAGGACTGCAATTGCTCMATCTAYCCCGGCCACGTATCGGGTCAYCGCATGGCTTGGGACATGATGATGAATTGGTCACCTACAACAGCTYTAGTGGTGTCGCAGTTRCTCCGGATCCCACAAGCTGTCGTGGACATAGTGGCKGGAGCCCACTKGGGAGTCCTGGCGGGCCTCGTCTACTATTCCATGGYGGCGAACTGGGCTAAGGTCTTGATTGTGATGCTACTTTTYGCCGGCGTCGACGGG

>HM106595

ATCGAAGTTCGCAACGTGTCCGGAGTGTACCATGTCACGAACGACTGCTCCAACGCAAGCATTGTGTATGAGGCAGCGGACATGATCATGCAYACCCCCGGGTGCGTGCCCTGCGTTCGGGAGAACAACTCCTCCCGCTGCTGGGTAGCGCTCACTCCCACGCTYGCGGCCAGGAACTCCAGCATCCCTACYACGACAATACGACGTCACGTCGATTTGCTCGTTGGGACGGCTGCTTTCTGCTCCGCTATGTATGTGGGAGATCTTTGCGGATCCGTTTTCCTTGTCTCTCAGCTGTTCACCTTCTCGCCTCGCCGGCATGAGACAGTACAGGACTGCAATTGCTCAATCTATCCCGGCCACGTAWCGGGTCAYCGCATGGCTTGGGACATGATGATGAACTGGTCACCTACAACAGCTCTAGTGGTGTCGCAGTTGCTCCGGATCCCACAAGCCGTCGTGGACGTAGTGGTGGGGGCCCACTGGGGAATCCTGGCGGGCCTCGCCTACTATTCCATGGTGGGGAACTGGGCTAAGGTCTTGATTGTGATGCTACTYTTTTCCGGCGTTGATGGG

>HM106596

NNNNNNNNNNNNNNNNNNNNNNNNNNNNNNNNNNNNNNNNNNNNNNNNNNNNNNNNNNNNNNTGTGTATGAGGCAGACGACATGATTATGCACACCCCCGGGTGCGTGCCCTGCGTCCGGGAGGAAAACTCCTCCCGCTGCTGGGTATCGCTCACTCCCACGCTCGCGGCCAGAAACTTCAGTATCCCCACTACGACAATACGACGTCACGTCGATTTGCTCGTTGGGACGGCTGCTTTCTGCTCCGCTATGTACGTGGGAGATCTTTGCGGATCTGTTTTCCTTGTCTCCCAGCTGTTCACCTTCTCGCCTCGCCGGCACGAGACAGTACAGGACTGCAATTGCTCAATTTATCCCGGCCACGTATCAGGTCACCGCATGGCTTGGGACATGATGATGAACTGGTCCCCTACAACAGCTCTAGTGGTGTCGCAGTTACTCCGGATCCCACAAGCTCTCTTAGACGTGGTGGCGGGAGCTCACTGGGGAGTCCTGGCGGGCCTCGCCTACTATTCCATGGTGGGAAACTGGGCTAAGGTCTTGATTGTGATGCTACTTTTTGCCGGCGTCGACGGG

>HM106597

ATYGARGTGCGYAACGTGTCCGGGTTGTACCATGTCACGAACGACTGCTCCAACRCAAGCATTGTGTAYGAGGCAGCGGACATGATTATGCATACCCCCGGGTGCGTGCCCTGCGTTCGGGAGKCCAACTCCTCCCGCTGCTGGGTAGCTCTCACTCCCACGCTCGCGGCCAGGAACGCCAGCATYCCCACTACGACAATACGACGYCACGTCGATTTGCTCGTTGGGACGGCTGCCCTCTGTTCCGCTATGTACGTGGGAGAYCTTTGCGGATCTGTTTTYCTTGTCGCTCAGCTGTTTACCTTCTCGCCTCGTCGGCATGAGACAGTACAGGACTGCAATTGCTCAATGTATCCCGGCCACCTATCGGGTCACCGCATGGCYTGGGACATGATGATGAACTGGTCACCTACAACAGCTCTAGTGGTGTCGCAGTTACTCCGGATCCCACAAGCTRTCGTGGACGTGGTGGCGGGAGCCCACTGGGGAGTCCTGGCGGGCCTCGCCTACTATTCCATGGTGGGGAACTGGGCGAAGGTTTTGATTGTGATGCTACTCTTTGCCGGCGTCGACGGG

>HM106598

ATCGAAGTGCGCAACGTGTCCGGGATGTACCATGTCACGAACGACTGCTCCAACGCAAGCATTGTGTATGAGGCAGCGGACATGATTATGCACATGCCCGGGTGCGTGCCCTGCGTTCGGGAGGACAACTCCTCCCGCTGCTGGGTAGCGCTCACTCCCACGCTCGCAGCCAGGAACTCCAGCATCCCCACTACGACAATACGACGTCACGTCGATTTGCTCGTTGGGGCGGCTGCTTTCTGCTCCGCTATGTACGTGGGAGATCTTTGCGGATCTGTTCTCCTTGTCTCTCAGCTATTCACCTTCTCGCCTCGCCGGCATGAGACAGTACAGGACTGCAATTGTTCAATCTATCCCGGCCACGTATCGGGTCACCGCATGGCCTGGGACATGATGATGAACTGGTCACCTACAACAGCTCTAGTGATGTCGCAGCTACTCCGGATCCCACAAGCCGTCTTGGATGTTGTGGCGGGAGCCCACTGGGGAGTCCTGGCGGGCCTCGCCTACTATTCCATGGTGGGGAACTGGGCTAAGGTCTTGGTTGTGATGCTACTTTTTGCCGGCGTCGACGGG

>HM106599

ATYGAGGTGCGCAACGTGTCCGGAGTGTACCATGTCACGAACGACTGCTCCAACGCAAGCATTGTGTATGAGGCAGCGGACATGATTATGCATACCCCCGGGTGCGTGCCCTGCGTTCGGGAGAGCAACTCCTCCCGCTGCTGGRTAGCGCTCACTCCCACGCTCGCGGCCAGGAACTCCAGCATTCCCACTACGACAATACGACGTCACGTCGATTTKCTCGTCGGGACAGCTGCYTTCTGCTCCGCTATGTACGTGGGAGATCTTTGCGGATCTGTTTTCCTTGTCTCTCAGCTGTTCACCTTTTCGCCTCGTCGGCATTTGACAGTGCAGGACTGCAATTGCTCAATCTATCCCGGCCAYGTAACGGGTCACCGCATGGCTTGGGACATGATGATGAACTGGTCACCTACAACAGCTYTAGTAGTGTCRCAGTTACTCCGGATCCCACAAGCTGTCGTGGACATGGTGGCGGGAGCCCACTGGGGAGTCCTGGCGGGCCTCGCCTACTATTCCATGGTGGGGAACTGGGCTAAGGTCTTGGTTGTGATGCTACTTTTTGCCGGTGTCGACGGG

>HM106600

ATCGAAGTGCGCAACGTGTCCGGAGTGTACCATGTCACGAACGACTGCTCCAACGCAAGYATTGTGTATGAGGCAGCGGACATGATTATGCATACCCCCGGGTGCGTGCCCTGCGTTCGGGAGAACAACTCCTCCCGCTGCTGGGTAGCGCTCACTCCCACGCTCGCGGCCAGGAACTCCAGCATCCCCACTACGACAATACGACGCCAYGTCGATTTGCTCGTTGGGACGGCTGCTTTCTGCTCCGCTATGTACGTGGGAGATCTTTGCGGATCTGTTTTCCTTGTCTCTCAGCTGTTYACCTTCTCGCCTCGCCGGCATGAGACAGTACAGGACTGCAATTGCTCAATCTATCCCGGCCACGTATCAGGTCACCGCATGGCTTGGGACATGATGATGAATTGGTCACCTACAACRGCTCTAGTGGTGTCGCAGTTACTCCGGATCCCACAAGCTGTCTTGGACATAGTGGCGGGAGCCCACTGGGGAGTCCTGGCGGGCCTCGCYTACTATTCCATGGTRGGGAACTGGGCTAAGGTCTTGATTGTGATGCTACTTTTTGCCGGCGTCGACGGG

>HM106601

ATCGAAGTGCGCAACGTGTCCGGAGTGTAYCATGTCACGAACGACTGCTCCAACGCAAGCATTGTGTATGAGGCAGCGGACATGATYATGCATACCCCCGGGTGCGTGCCCTGCGTTCGGGAGAACAACTCCTCCCGCTGCTGGGTAGCGCTCACTCCCACGCTCGCGGCCAGGAACTCCAGCATCCCCACCACGACAATACGACGTCACGTCGATTTGCTCGTTGGGACGGCTGCTTTCTGCTCCGCTATGTACGTGGGAGATCTTTGCGGATCTGTTTTCCTTGTCTCTCAGCTGTTCACCTTCTCGCCTCGCCGGCATGAGACAGTACAGGACTGCAATTGCTCAATCTATCCCGGCCACGTATCGGGTCATCGCATGGCTTGGGACATGATGATGAACTGGTCACCTACAACAGCTCTAGTGGTGTCGCAGTTACTCCGGATCCCACAAGCCATCCTGGACGTGGTGGCGGGAGCCCACTGGGGAGTCCTGGCGGGCCTCGCCTACTATTCCATGGTGGGGAACTGGGCTAAGGTCTTGATTGTGATGCTACTTTTTGCCGGCGTCGACGGA

>HM106602

ATCGAAGTGCGCAACGTGTCCGGRGTGTACCATGTCACGAACGACTGCTCCAACGCAAGCATTGTGTATGAGGCAGCGGACATGATTATGCAYACCCCYGGGTGCGTGCCCTGCGTTCGGGAGAACAACTCCTCCCGYTGCTGGGTAGCGCTCACTCCCACGCTCGCGGCYAGGAACTCCAGCATCCCCACTACGACAATACGACGCCACGTCGATTTGCTCGTTGGGACGGCTGCTTTCTGCTCCGCTATGTACGTGGGAGATCTTTGCGGATCTGTTTTCCTTGTCTCTCAGCTGTTCACCTTCTCGCCTCGCCGGCATGAGACAGTACAGGACTGCAATTGCTCAATCTATCCCGGCCACCTAACGGGTCATCGCATGGCTTGGGACATGATGATGAACTGGTCACCTACAACAGCTCTAGTGGTGTCGCAGTTACTCCGGATCCCACAAGCTGTCGTGGACATAGTGGCGGGRGCCCACTGGGGAGTCCTGGCGGGCCTCGCCTACTATTCCATGGTGGGGAACTGGGCTAAGGTCTTGATTGTGATGCTACTTTTTGCCGGCGTCGACGGG

>HM106603

ATTGAAGTGCGCAACGCGTCCGGAGTGTACCATGTCACGAACGACTGCTCCAACGCAAGYATTGTGTATGAGGCAGCGGACATGATTATGCACAYCCCCGGGTGCGTGCCCTGCGTKCWGGAGRRYRRCTCCYCCCGCTGCTGGGTAGCGCTCACTCCYACKCTCGCGGCCAGGAACYTCAGYATCCCCACTACGACAATACGACGTCACGTCGATTTGCTCGTTGGGACGGCTGCTTTCTGCTCCGCTATGTACGTGGGAGATCTTTGCGGATCYGTTTTCCTYGTCTCTCAGCTGTTCACCTTCTCGCCTCGCCRGCATRTRACAGTACAGRRCTGCAAYTGYTCAATCTATCCCGGCCACGTAACGGGTCACCGCATGGCTTGGGACATGATGATGAACTGGTCACCTACAACAGCTCTAGTGGTGTCGCAGTTACTCCGGATCCCACAAGCTGTCGTGGACATGGTGGCGGGAGCCCACTGGGGAGTCCTGGCGGGCCTCGCCTACTATTCCATGGTGGGGAACTGGGCTAAGGTCTTGATTGTGATGCTACTTTTTGCCGGCGTCGACGGG

>HM106604

ATCGAGGTGCGCAACGTGTCCGGAGTGTACCATGTCACGAACGACTGCTCCAACACAAGCATTGTGTATGAGGCAGCGGACGTGATTATGCATATCCCCGGGTGCGTGCCCTGCGTTCGGGAGGACAACTYCTCCCGCTGCTGGGTAGCGCTCACTCCCACGCTYGCGGCCAGGAACTCCAGCATCCCCACYACGACAATACGACGTCACGTCGATTTGCTCGTTGGGACGGCTGCTTTCTGCTCCGCTATGTACGTGGGAGATCTTTGCGGATCTGTTTTCCTTGTCTCTCAGCTGTTCACCTTCTCGCCCCGCCGGCACGAGACAGTACAGGACTGTAACTGTTCAATCTAYCCCGGCCACGTAACGGGTCACCGCATGGCTTGGGATATGATGTTGAACTGGTCACCTACAACAGCTCTAGTGGTGTCGCAGTTGCTCCGGATCCCACAAGCCGTCGTGGACATGGTGGCGGGAGCCCACTGGGGAGTCCTGGCGGGCCTCGCCTACTATTCCATGGTGGGGAACTGGGCTAAGGTCTTGGTTGTGATGCTACTTTTTGCCGGCGTCGACGGG
