# Supplementary material for: Effect of Immune Pressure on Hepatitis C Virus Evolution: Insights From a Single-Source Outbreak
Source: Hepatology. 2011 Feb;53(2):396–405. doi: 10.1002/hep.24076 (PMC3044208; doi:10.1002/hep.24076)
Supplement: Supplementary file 4 [file hep0053-0396-SD4.doc]

>HM106605

CAGACYTACACGACGGGGGGGGCGCAGGCCCGTACTACCAACAGGCTTACGTCCCTCTTTACRTCTGGGCCGTCCCARAARATCCAGCTTRTAAACACCAACGGCAGCTGGCACATCAATAGGACTGCCCTGAACTGCAATGACTCCCTCAAYACYGGGTTCCTTGCCGCGCTGTTCTACACCCACAGCTTYAACTCGTCYGGATGCCYGGAGCGCATGGCCAGCTGCCGCCCCATTGACAAGTTCGTYCAGGGGTGGGGTCCCATCACTTAYGCYGAGCCGCCCAGCYTGGACCAGAAGCCYTAYTGTTGGCAYTACGCACCCCAACCGTGYGGTATTGTRCCCGCGTYGSAGGTGTGYGGTCCAGTATACTGTTTCACCCCAAGCCCTGTTGTGGTGGGGACGACCGATCGTTCCGGCGCCCCTACGTATAGRTGGGGGGAGAATGAGACGGACGTGCTGCTTCTCAACAACACGCGGCCSCCGCGAGGCAACTGGTTCGGCTGTACATGGATGAATAGCACYGGGTTCACCAAGACGTGCGGGGGCCCCCCGTGCAAYATYGGGGGGGTCGGTAATGACACCTTGACCTGCCCCACGGATTGCTTCCGGAAGCACCCCGAGGCCACTTACACCAAATGCGGWTCGGGGCCCTGGCTGACACCTAGGTGYATGGTTCACTATCCATACAGGCTTTGGCACTACCCCTGCACTGTCAATTTTACCATCTTCAAGGTTAGGATGTATGTGGGGGGTGTGGAGCACAGGCTCGACGYCGCATGCAACTGGACCCGMGGAGAGCGTTGTGACTTGGAGGACAGGGATAGRTCAGARCTTAGCCCRCTGCTRCTGTCTACAACAGAGTGGCAGGTAYTGCCCTGTTCCTTCACCACCCTACCGGCTCTGTCCACCGGCTTGATCCATCTCCATCAGAACATCGTGGACGTGCAATACCTGTACGGTATAGGGTCAGCGGTTGTCTCCTACGCRATTAAGTGGGAGTACGTCCTGTTGCTCTTCYTTTTCCTGGCGGAYGCGCGCGTCTGTGCCTGCTTGTGGATGATGCTGCTAATAGCTCAGGCTGAGGCC

>HM106606

ACGACTTACACGACRGGGGGAGCGCAGGCCCAYACCACCCGYGGGCTTGTGAGCCTCTTTGCGCCTGGGCCGTCCCAAAGCATCCAGCTTATAAACACCAACGGCAGCTGGCACATCAACAGGACTGCCCTGAACTGCAATGACTCTCTCCACACTGGGTTCCTTGCCGCGCTGTTCTACRCCCACAAATTCAACGCGTCCGGATGCCCGGAACGCATGGCCAGCTGCCGCCCCATTGACAAGTTCGCTCAGGGGTGGGGACCCATCACTTACGCTGAGCCGCCCAGCTTGGACCAGAAGCCCTACTGCTGGCACTACGCACCCCGACCGTGCGGTATCGTACCCGCGTCGCAGGTGTGCGGTCCAGTGTACTGTTTCACCCCAAGCCCTGTTGTGGTGGGGACGACCGATCGTTTCGGCGTCCCTACGTACAGCTGGGGGGAGAATGAGACGGACGTGCTGCTTCTCAACAACACGCGGCCGCCGCGAGGCAACTGGTTCGGCTGCACATGGATGAATAGCACTGGGTTCACCAAGACGTGCGGGGGCCCCCCGTGCAACATCGGGGGGGTCGGTAATGACACCTTGACCTGCCCCACGGATTGCTTCCGGAAGCACCCCGAGGCCACTTACACCAAATGCGGTTCGGGGCCCTGGCTGACACCTAGGTGCATGGTTGACTACCCATACAGGCTTTGGCACTACCCCTGCACTGTCAATTTTACCATCTTCAAGGTTAGGATGTATGTGGGGGGTGTGGAGCACAGGCTCAACGCCGCATGCAACTGGACCCGAGGAGAGCGTTGTGACTTGGAGGACAGGGATAGATCAGAGCTTAGCCCGCTGCTGCTGTCTACAACAGAGTGGCAGGTATTGCCCTGTTCCTTCACCACCCTACCGGCTCTGTCCACCGGCTTGATCCATCTCCATCGGAACATCGTGGACGTGCAATACCTGTACGGTATAGGGTCAGCGGTTGTCTCCTACGCAATTAAGTGGGAGTACGTCCTGTTGCTCTTCCTCTTCCTGGCGGACGCGCGCGTCTGTGCCTGCTTGTGGATGATGCTGCTAATAGCTCAGGCTGAGGCC

>HM106607

NNNNNNNNNNNNNNNNNNNNNNNNNNNNNNNNNNNNNNNNNNNNNNNNNNNNNNNNNNNNNNNNNNNNNNNNNNNNNNNNNNNNNNNNNNNNNNNNNNNNNNNNNNNNNNNNNNNNNNNNNNNNNNNNNNNNNNNNNNNNNNNNNNNNNNNNNNNNNNNNNNNNNNNNNNNNNNNNNNNNNNNNNNNNNNNNNNNNNNNNNNNNNNNNNNNNNNNNNNNNNNNNNNNNNNNNNNNNNNNNNNNNNNNNNNNNNNNNNNNNNNNNNNNNNNNNNNNNNNNNNNNNNNNNNNNNNNNNNNNNNNNNNNNNNNNNNNNNNNNNNNNNNNNNNNNNNNNNNNNNNNNNNNNNNNNNNNNNNNNNNNNNNNNNNNNNNNNNNNNCCCCAAGCCCTGTTGTGGTGGGGACGACCGATCGTTTCGGCGCCCCCACGTATAACTGGGGGGAGAATGAGACGGATGTGCTGCTCCTTAACAACACGCGGCCGCCGCGAGGCAACTGGTTCGGCTGCACGTGGATGAATGGCACTGGGTTCACCAAGACGTGCGGGGCCCCCCCGTGCAACATCGGGGGGGGCGGTAACGACACCTTGATCTGCCCCACGGATTGCTTCCGGAAGCACCCCGAAGCCACTTACACTAAATGCGGTTCGGGGCCCTGGCTGACGCCCAGGTGCATGGTTGACTACCCATACAGGCTTTGGCACTACCCCTGCACTGTTAATTTTACCATCTTCAAGGTTAGGATGTATGTGGGGGGTGTGGAGCACAGGCTCCAAGCCGCATGCAACTGGACCCGAGGAGAGCGTTGTGACTTGGAGGACAGGGATAGATCAGAGCTTAGCCCGCTGCTGCTGTCTACAACAGAATGGCAGGTATTGCCCTGTTCCTTCACCACCCTACCGGCTCTGTCCACCGGCTTGATCCACCTCCATCGGAACATTGTGGACGTGCAGTACCTGTACGGTATAGGGTCAGCGGTTGTCTCCTACGCGATTAAGTGGGAGTATGTCCTGTTGCTCTTCCTTTTCCTGGCAGACGCGCGCGTCTGTGCCTGCTTGTGGATGATGCTGCTAATAGCCCAGGCTGAGGCC

>HM106608

SRCACTTAYRCCACAGGGGSGGYGSAGGGCCRTACYACCTTSGGGTTTRCGAGCMTCTTTAGCSKTGGGCCGKCCCAAAGAATCCAGCTCATAAACTCCAACGGYAGCTGGCACATCAACAGGACTGCCCTGAACTGCAATGATTCCCTCAACACTGGGTTCCTTGCYGCGCTGTTCTAYGCCMASAAATTCAAYGATTCCGGATGCHYGGAGCGCATGGCCAGCTGCCGCCCCATTGACAAGTTCGCTCAGGGGTGGGGTCCCATCACTTACGCCAAGTCGCCCAGCTTAGACCAGAAGCCCTATTGTTGGCACTACGCACCYCAACCGTGYGGTATTGTACCCGCGTCGCAGGTGTGCGGTCCAGTGTACTGCTTYACCCCAAGCCCTRTTGTGGTRGGGACGACCGATCGCYTCGGCGTYCCTACGTATARCTGGGGSGAGAATGAGACGGACGTGCTGCTTCTCAACAACACGCGGCCGCCGCRAGGCAACTGGTTCGGCTGCACATGGATGAATGGCACTGGGTTCACCAAGACGTGCGGRGGCCCCCCGTGCRACATCGGGGGGGTCGGCAATGACACCYTGATMTGCCCCACGGATTGCTTCCGGAAGCACCCCGAGGCCACTTACRCCAAATGYGGYTCGGGGCCCTGGTTGACACCTAGGTGCATSGTTGACTACCCATACAGGCTTTGGCACTACCCCTGYACYGTCAAHTTTYCCATCTTCMAGRTTAGGATGTATGTGGGGGGTGTGGAGCACAGGCTCAMCGCCGCATGCAACTGGACCCGAGGARACCGTTGTGACTTGGAGGACAGGGATAGATCAGAGCTTAGCCCGCTGCTGCTGTCTACAACAGAGTGGCAGGTATTGCCCTGTTCCTTCACCACCCTACCGGCTCTGTCCACCGGCTTGATCCATCTCCAYCGGAACATTGTGGACRTACAATACCTGTACGGTATAGGGTCAGCGGTTGTCTCCTACGCAATTAAGTGGGAGTATGTCCTGTTGCTYTTCYTTTTCCTGGCGGACGCGCGCGTCTGYGCCTGCTTGTGGATGATACTGCTAATAGTTCAGGCTGAGGCC

>HM106609

NNNNNNNNNNNNNNNNNNNNNNNNNNNNNNNNNNNNNNNNNNNNNNNNNNNNNNNNNNNNNNNNNTGGGCCGTCTCARAACATCCAGCTTATAAACACCAACGGCAGCTGGCACATCAACAGGACTGCCCTGAACTGYAATGACTCCCTCAACACYGGGTTCCTTGCCGCRCTGTTCTACGCCCRCARATTCAACGCGTCCGGATGCCCGGAGCGCATGGCCAGCTGCCGCCCCATTGACAAGTTCGCTCAGGGGTGGGGTCCCATCACTTACGCTARGCCGSCCAGCTTGGACCAGAAGCCCTAYTGYTGGCACTACGCACCCCAACCRTGYGGTATYGTRCCCGCGTCGCAGGTGTGCGGTCCAGTGTAYTGTTTCACCCCAAGCCCTGTTGTGGTGGGGACGACCGATCGTTTCGGCGTCCCTACGTACAGCTGGGGGGAGAATGAGACGGACGTGCTGCTTCTCAACAACACGCGGCCGCCGCGAGGCAACTGGTTCGGCTGCACATGGATGAATGGCACTGGGTTCACCAAGACGTGYGGGGGCCCCCCGTGCAACATCGGGGGGGTCGGTAATGACACCTTGATCTGCCCCACGGATTGCTTCCGGAAGCACCCCGAGGCCACTTACACCAAATGCGGYTCGGGGCCYTGGCTGACACCTAGGTGCATGGTTGACTACCCATACAGGCTTTGGCAYTACCCCTGCACTSTCAATTTYACCATCTTCAAGGTTAGAATGTATGTGGGGGGTGTGGAGCACAGGCTCAACGCCGCATGCAACTGGACCCGAGGAGAGCGTTGTGACTTGGAGGACAGGGATAGRTCAGAGCTTAGCCCGCTGCTGCTGTCTACAACAGAGTGGCAGATATTGCCCTGTTCCTTCACCACCCTACCGGCTCTGTCCACCGGCTTGATCCATCTCCATCGGAACATCGTGGACGTGCAATACCTSTACGGTATAGGGTCAGCGGTTGTCTCCTACGCAATYAAGTGGGAGTATGTCCTGTTGCTCTTCCTTTTCTTGGCGGACGCGCGCGTCTGTGCCTGCTTGTGGATGATGCTGCTAATAGYTCAGGCTGAGGCC

>HM106610

ACGACTCACGTGACGGGGGCGGTGCAGGGCCRDACTRCCACCATGTTTRCGMRCYTCTTTACGYSTGGGCCGWMMCAAAMCATCCAGCTTGTAAACACCAAYGGCAGCTGGCACATCAACAGGACTGCCCTGAACTGCAATGACTCCCTCAASACCGGGTTCCTTGCCGCGCTGTTCTACGTCAAAGGATTCAATTCGTCTGGATGCACGGAGCGCGTGGCCAGCTGCCGCCCCATTGACAAGTTCACTCAGGGGTGGGGTCCCATCACTTACGCTGAGCCGGCCAGCTTAGACCAGAAGCCCTATTGTTGGCACTACGCACCCAAACCGTGCGGTATTGTACCCGCGTCGCAGGTGTGCGGTCCAGTGTACTGTTTCACCCCAAGCCCTGTTGTGGTGGGGACGACCGATCGYTYYGGCGYMCCTACGTATAGMTGGGGGGMGAATGAGACGGACGTGCTGCTTCTCAACAAYACGCGGCCGCCGCAAGGCAACTGGTTTGGTTGCACATGGATGAATGGCACTGGGTTCACCAAGACGTGCGGGGGCCCCCCGTGCAACATCGGGGGGGTCGGTAATGACACCTTGATTTGCCCCACGGACTGCTTCCGGAAGCACCCCGAGGCCACTTACGCCAAATGCGGTTCGGGGCCCTGGCTGACACCTAGGTGCATAGTTGACTACCCATACAGGCTTTGGCACTACCCCTGCACTGTCAATTATACCATCTTCAAGGTTAGGATGTATGTGGGGGGYGTGGAGCACAGACTCAACGCCGCGTGCAACTGGACCCGGGGAGATCGTTGTGACTTGGAGGACAGGGATAGATCAGAGCTTAGCCCGCTGCTGCTGTCTACAACAGAGTGGCAGGTAYTGCCCTGTTCCTTCACCACCCTACCGGCTCTGTCCACCGGCTTGATCCATCTCCATCAGAACATCGTGGACGTGCAATACCTGTACGGTATAGGGTCAGCGGTTGTCTCCTACGCAATTAAGTGGGAGTATGTCCTGTTGCTCTTCCTTTTCCTGGCGGACGCGCGCGTCTGTGCCTGCTTRTGGATGATGCTGCTAATAGCTCAGGCTGAGGCC

>HM106611

GGCACTTACACCACAGGGGGGGCGCAGGCCCGCACTACCCTCGGGCTTACGACCCTCTTCACGCGCGGGCCGTCCCAGGGCATCCAGCTCGTAAACTCCAACGGCAGCTGGCACATCAACAGGACTGCCCTGAACTGCAATGATTCCCTCAACACTGGGTTCCTTGCCGCGCTGTTCTACGCCCACAAGTTCAACTCGTCCGGATGCCCGGAGCGCTTGGCCAGCTGCCGCCCCATTGACAAGTTCGCTCAGGGGTGGGGTCCCATCACTTACGCTCAGCCGCGCAGCTTGGACCAGAAGCCCTATTGYTGGCACTAYGCACCCCAACCGTGCGGTATTGTACCCGCGKCGCAGGTGTGTGGTCCAGTGTAYTGYTTCACCCCAAGCCCTGTTGTGGTGGGGACGACCGATCGTTTCGGCGTCCCTACGTACAGCTGGGGGGAGAATGAGACGGACGTGCTGCTTCTCAACAACACGCGGCCGCCGCGAGGCAACTGGTTCGGCTGCACATGGATGAATGGCACYGGGTTCACCAAGACGTGCGGGGGCCCCCCGTGCAACATCGGGGGGGTCGGCAATGACACCTTGACCTGCCCCACGGATTGCTTCCGGAAGCACCCCGAGGCCACTTACACCAAATGCGGKTCGGGGCCCTGGTTGACACCGAGGTGCATGGTTGACTACCCATACAGGCTYTGGCACTACCCCTGCACTGTCAATTTTTCCATCTTCAAGGTTAGGATGTATGTGGGGGGTGTGGAGCACAGGMTCAACGCCGCATGCAACTGGACCCGGGGAGAGCGTTGTGACTTGGAGGACAGGGATAGATCAGAGCTCAGCCCGCTGCTGCTGTCTACAACAGAGTGGCAGGTATTGCCCTGTTCCTTCACCACCCTACCGGCTCTGTCCACCGGYTTGATCCATCTCCATCGGAACATCGTGGACATACAATACCTGTACGGTATAGGGTCAGCGGTTGTCTCCTACGCAATTAAGTGGGAGTATGTCCTGTTGCTCTTCCTTTTTCTGGCRGACGCGCGCGTCTGTGCCTGCTTGTGGATGATGCTGCTAATAGTTCAGGCTGAGGCC

>HM106612

GGGACTTACACCACGGGGGGGGCGCAGGCCCGTACTACCAAAGGGYTTACGCAAYTCYTCTCGBCTGGGCCGTCCCAAAACATCCAGCTTGTAAACACCAACGGCAGCTGGCACATCAACAGGACTGCCCTGAACTGCAATGAATCCCTCAACACTGGGTGGCTTGCCGCGCTGTTCTACRCCCGCAAATTCAACGCGTCCGGATGCCCGGAGCGCATGGCCAGCTGCCGCCCCATTCAGAATTTCACTCAGGGGTGGGGTCCCATCRCTTATGCYAAGCCGCYCAACTTGGACCAGAAGCCCTATTGYTGGCACTACGCACCCCAACCGTGCGGTATTGTACCCGCGTCGCAGGTGTGCGGTCCAGTGTACTGCTTCACCCCRAGCCCTGTTGTGGTGGGGACGACCGATCGTTTCGGAGTCCCYACGTACAGCTGGGGRGAGAATGAGACGGACGTGCTGCTTCTCAACAACACGCGGCCGCCGCRAGGCAACTGGTTTGGCTGCACATGGATGAATGGCACTGGGTTCACCAAGACGTGCGGGGGCCCCCCGTGCGACATCGGGGGGTCCGGCAATAACACCTTGAYCTGCCCCACGGATTGCTTCCGGAAGCACCCCGAGGCCACTTACGCTAAATGCGGTTCGGGGCCCTGGTTGACACCTAGGTGCATGGTTGACTACCCATACAGGCTTTGGCACTACCCCTGCACTGTCAATTTTTCCATCTTCACGGTYAGGATGTATGTGGGGGGTGTGGAGCACAGGCTCAAAGCCGCATGCAACTGGACCCGAGGRGAGCGTTGTGACTTGGAGGACAGGGATAGATCGGAGCTTAGCCCGCTGCTGCTGTCTACAACAGARTGGCAGGTATTGCCTTGTTCCTTCACCACCCTACCGGCTCTATCCACCGGCTTAATCCATCTCCATCAGAACATCGTGGACGTACAATACCTGTACGGTATAGGGTCAGCGGTTGTCTCCTACGCAATTAAGTGGGAGTATGTCCTGTTGCTCTTCCTYTTCCTGGCGGACGCGCGCGTCTGTGCCTGCTTGTGGATGATGCTGCTAATAGYCCAGGCTGAGGCC

>HM106613

AGCACTCGCACCATAGGGGGGGCGACGGCCCGTACTACCCAAGGGCTTACGAGCATCTTTCAGTCTGGGCCGTCCCAAAAMCTCCAGCTCATAAACTCCAACGGCAGCTKGCACATCAACAGGACTGCCCTGAACTGCAATGATTCCCTCAACACTGGGTGGCTTGCCGCGCTGTTCTACACCCACARATTCAACTCGTCCGGATGCCCGGAGCGCWTGGCCAGCTGCCGCCCCATTGACAAGTTCGCTCAGGGGTGGGGTCCCATCACTTACGCTAGGCCGCCCAACTTGGACCAGAAGCCCTATTGTTGGCACTACGCACCCCAACCGTGCGGTATTGTACCCGCGTCGCAGGTGTGCGGTCCAGTGTACTGCTTCACCCCAAGCCCTGTTGTGGTGGGGACGACCGATCGGCTCGGCGTCCCTACGTACAGMTGGGGGGAGAATGAGACGGACGTGCTGCTTCTCAACAACACGCGSCCGCCRCAAGGCAACTGGTTCGGCTGCACATGGATGAATRGCACTGGGTTCACYAAGACGTGCGGGGGCCCCCCGTGCRACATCGGGGGGGTCGGCAACGACACCTTGATCTGCCCCACGGATTGCTTCCGGAARCACCCCGAGGCCACTTACGCCAAATGCGGTTCGGGGCCCTGGTTGACACCTAGGTGCATGGTTGACTACCCATACAGGCTYTGGCACTACCCCTGCACTGTCAATTTTTCCATCTTTAAGGTYAGGATGTACGTGGGGGGTGTGGAGCACAGGCTCAACGCCGCATGCAACTGGACYCGAGGAGAGCGTTGTGACTTGGAGGACAGGGATAGATCAGAGCTTAGCCCGCTGCTGCTGTCTACAACAGAGTGGCAGGTATTGCCCTGTTCCTTCACCACCCTACCGGCTCTGTCCACYGGCTTGATCCACCTCCATCGGAACATCGTGGACGTACAATACCTGTACGGTATAGGGTCAGCGGTTGTCTCCTACGCAATTAAGTGGGAGTATATCCTGTTGCTCTTCCTCCTCCTGGCGGACGCGCGCGTCTGTGCCTGTTTGTGGATGATGCTGCTAATAGCYCAGGCTGAGGCC

>HM106614

CACACTCGCGCGGTAGGGGGGTCGCAGGCCYAYACTACCCGCGGGWTTGTGAGCCTCTTTACGAAAGGGCCGTCCCAAAAGATCCAGCTTGTAAAYACCAACGGCAGCTGGCACATCAACAGGACTGCCCTGAATTGCAATGACTCCCTCAACACCGGGTTCCTTGCCGCGCTGTTCTACACCCACAAATTCAACGCGTCCGGATGCCCGGAGCGCATGGCCAGCTGCCGCCCCATCGACAAGTTCGCTCAGGGGTGGGGTCCTATCACCTATGCTGAGCCGGCCAGCTTGGACCAGAAGCCCTATTGTTGGCACTACGCACCCCAACCGTGCGGTATTGTACCCGCGTCGCAGGTGTGCGGTCCAGTGTACTGTTTCACCCCAAGCCCTGTTGTGGTGGGGACGACCGAYCGTTTCGGCGTCCCCACGTAYAGCTGGGGGGAGAATGAGACGGACGTGCTGCTTCTCAACAACACGCGGCCGCCGCGAGGCAACTGGTTCGGCTGCACATGGATGAATGGCACTGGGTTCACCAAGACGTGTGGGGGCCCCCCGTGCAATATCGGGGGGGTCGGTAATGACACCTTGATCTGCCCCACGGATTGCTTYCGGAAGCACCCCGAGGCCACTTACACCAAATGCGGTTCGGGGCCCTGGCTGACACCTAGGTGCATGGTTGACTACCCATACAGGCTTTGGCATTACCCCTGCACTGTCAATTTTACCATCTTCAAGGTYAGGATGTATGTGGGGGGTGTGGAGCACAGGCTCAACGCCGCATGCAACTGGACCCGGGGAGAGCGTTGTGACTTGGAGGACAGGGATAGATCAGAGCTTAGCCCGCTGCTGCTGTCTACAACAGAGTGGCAGGTATTGCCCTGCTCCTTCACCACCCTACCGGCTCTGTCCACCGGCTTGATCCATCTCCATCAGAACATCGTGGACGTGCAATACCTGTACGGTATAGGGTCAGCGGTTGTCTCCTACGCAATTAAGTGGGAGTACGTCCTGTTGCTCTTCCTTTTCCTGGCGGACGCACGCGTCTGTGCCTGCTTGTGGATGATGCTGTTAATAGCTCAGGCTGAGGCT

>HM106615

CACACTTACGTGACAGGAGGGGCGCAGGCCCGTACTGYCAGCGGGCTTGCGAGCTTCTTTTCGCCTGGGCCGGCCCAAAAAATCCAGCTTGTAAACTCCAGCGGCAGCTGGCACATCAACCGCACTGCCCTGAACTGCAATGACTCCCTCAACACTGGGTTCATTGCCGCGCTGTTCTACACCTACAAATTCAACTCGTCCGGATGCCCGGAGCGCATGGCCAGCTGCCGCCCCATTGACAAGTTCGCTCAGGGGTGGGGTCCCATCACATACGCTAAGYCGCCCAGCTTAGACCAGAAGCCCTATTGTTGGCACTACGCACCCCAACCGTGCGGTATTGTACCCGCGTTGAATGTGTGCGGTCCAGTGTACTGTTTCACCCCAAGCCCTGTTGTGGTGGGGACGACCGATCGTTTCGGCGTCCCTACGTATAACTGGGGGGAGAATGAGACGGACGTGCTGCTTCTCAACAGCACGCGGCCGCCGCAGGGCTTCTGGTTCGGCTGCACATGGATGAATGGCTCTGGGTTCACCAAGACGTGCGGGGGCCCCCCGTGCAACATCGGGGGGGCCGGCAATGACACCTTGATCTGCCCCACGGATTGCTTTCGGAAGCACCCCGAGGCCACTTACACCAAATGCGGTTCGGGGCCCTGGCTGACACCTAGGTGCATCGTTGACTACCCATACAGGCTTTGGCACTACCCCTGCACTGTCAATTTTACCATCTTCAAGGTTAGGATGTATGTGGGGGGTGTGGAGCACAGGCTCAACGCCGCATGCAATTGGACCCGAGGAGAGCGCTGTGACTTGGAGGACAGGGATAGATCAGAGCTTAGCCCGCTGCTGCTGTCTACAACAGAGTGGCAGGTATTGCCCTGCTCCTTCACCACCCTACCGGCTCTGTCCACCGGCTTGATCCATCTCCATCGGAACATCGTGGACGTGCAATACCTGTACGGTATAGGGTCAGCGGTTGTCTCCTACGCAATTAAGTGGGAGTACGTCCTGTTGCTCTTCCTTTTCCTGGCGGACGCGCGCGTTTGTGCCTGCTTGTGGATGATGCTGCTGATAGCTCAGGCTGARGCC

>HM106616

NNNNNNNNNNNNNNNNNNNNNNNNNNNNNNNNNNNNNNNNNNNNNNNNNNNNNNNNNNNNNNNNNNNNNNNNNNNNNNNNNNNNNNNNNNNNNNNNNNNNNNNNNNNNNNNNNNNNNNNNNNNNNNNNNNNNNNNNNNNNNNNNNNNNNNNNNNNNNNNNNNNNNNNNNNNNNNNNNNNNNNNNNNNNNNNNNNNNNNNNNNNNNNNNNNNNNNNNNNNNNNNNNNNNNNNNNNNNNNNNNNNNNNNNNNNNNNNNNNNNNNNNNNNNNNNNNNNNNNTGAGCCGTCCAGCGTGGACCAGAAGCCCTATTGTTGGCACTACGCGCCCCGACCGTGCGGTATTGTACCCGCGTCGCAGGTGTGCGGCCCAGTGTACTGTTTCACCCCAAGCCCTGTTGTGGTGGGGACGACCGATCGYTTCGGCGTCCCTACGTATAGCTGGGGGGAGAATGAGACGGACGTGCTGCTTCTCAACAACACGCGGCCGCCGCAAGGCAACTGGTTCGGCTGCACRTGGATGAATGGCACTGGGTTCACCAAGACGTGCGGGGGCCCCCCGTGCAACATCGGGGGGGTCGGTAATCACACCTTGACCTGCCCCACGGATTGCTTCCGGAAGCACCCCGAGGCCACTTACACCAGATGCGGGTCGGGGCCTTGGCTGACACCTAGGTGCATGGTTGACTACCCATACAGGCTTTGGCACTACCCCTGCACTGTCAATTTTACCATCTTTAAGGTTAGGATGTATGTGGGGGGTGTGGAGCACAGGCTCACCGCCGCRTGCAACTGGACCCGAGGAGAACGTTGTGATTTGGAGGACAGGGATAGATCAGAGCTTAGCCCGCTGCTGCTGTCTACGACAGAGTGGCAGGTATTGCCCTGYTCCTTCACCACCCTACCGGCTCTGTCCACCGGCTTGATCCATCTCCATCGGAACATCGTGGACGTGCAATACCTGTACGGTATAGGGTCAGCGGTTGTCTCCTACGCAATTAAGTGGGAGTACGTCGTGTTGCTCTTCCTTTTCCTGGCGGACGCGCGCGTCTGTGCCTGCTTGTGGATGATGCTGCTAGTAACTCAGGCTGAGGCC

>HM106617

TGGATTATGGGGGGGGTGCAGAGCCACACTACCAGCAAGCTTGCATCCTTCTTTGCGCCCGGGCCGTCCCAAAAGATCCAGCTTATAAACACCAACGGCAGCTGGCACATCAACAGGACTGCCCTGAACTGCAATGAATCCCTCCACACCGGGTTCCTTGCCGCGCTGTTCTACACCCACAGATTCAACGCGTCCGGATGCCCGGAGCGCATGGCCAGCTGCCGCCCCATTGACAAGTTCGCTCAGGGGTGGGGTCCCATCACTTACACTAAGCCGCCCAACTTGGACCAGAGGCCCTATTGCTGGCACTACGCACCCCAACCGTGCGGTATTGTACCCGCGTCGCAGGTGTGCGGTCCAGTGTACTGTTTCACCCCAAGCCCTGTTGTGGTGGGGACGACCGATCGTTTCGGCGTCCCTACGTACAACTGGGGGGAGAATGAGACGGACGTGCTGCTTCTCAACAACACGCGGCCGCCGCAAGGCAACTGGTTCGGCTGCACATGGATGAACAGCACTGGGTTCACCAAGACGTGCGGGGGCCCCCCGTGCAACATCGGGGGGGTCGGCAATGACACCTTGATCTGCCCCACGGATTGCTTCCGGAAGCACCCCGAGGCCACTTACGCCAAATGCGGTTCGGGGCCCTGGCTGACACCTAGGTGCATGGTTGACTACCCATACAGGCTTTGGCACTACCCCTGCACCGTCAACTTTACCACCTTCAAGGTTAGGATGTACGTAGGGGGTGTGGAGCACAGGCTCGTCGCCGCATGCAACTGGACCCGAGGAGAGCGTTGTGAATTGGAGGACAGGGACAGATCAGAGCTTAGCCCGCTGCTGCTGTCCACAACAGAGTGGCAGGTATTGCCCTGTTCCTTCACCACCCTACCGGCTCTGTCCACCGGCTTGATCCATCTCCATCGGAACAACGTGGACGTGCAATACCTGTACGGTATAGGGTCAGCGGTTGTCTCCTACGCAATTAAGTGGGAGTACGTCCTGTTGCTCTTCCTTTTCCTGGCGGACGCGCGCGTCTGTGCCTGCTTGTGGATGATGCTGCTAATAGCTCAGGCTGAGGCC

>HM106618

GCGACTTATACGACGGGGGGGGCGSWAGGCCGTACTACCTTCGGGTTTGCGTCCCTCYTTACGTCCGGGCCGTCTCAAAAAATCGAGCTTGTGAACACCAACGGCAGCTGGCACATCAACAGGACTGCCCTCAACTGCAATGACTCCCTCAACACTGGGTTCYTTGCCGCGCTGTTCTACGCCCACAAATTCAACTCGTCTGGGTGCCCGGAGCGCATGGCCAGCTGCMGCCCCATTGGCGAGTTCRMTCAGGGGTGGGGYCCCATCACTTATGCTGAGCCGYCCAGCTYGGACCAGAAGCCCTATTGYTGGCAYTACGCACCCCAACCGTGCGGTATTGTACCCGCGTCGAAGGTGTGCGGTCCAGTGTACTGTTTCACCCCAAGCCCCGTTGTGGTGGGGACGACYGATCGTTYCGGCGTCCCTACGTATAGCTGGGGAGGGAATGAGACGGACGTGCTGCTTCTCAACAACACGCGGCCGCCGCGAGGCAACTGGTTCGGCTGCACATGGATGAATAGCACTGGGTACACCAAGACGTGCGGRGGCCCCCCGTGCAACATCGGGGGGNNNNNNNNNNNNACCTTGATCTGCCCCACGGATTGCTTCCGGAAGCACCCCGAGGCCACTTACACCAAATGCGGTTCGGGGCCTTGGCTGACACCTAGGTGCATGGTTGACTACCCATACAGGCTYTGGCACTACCCCTGCACTGTCAATTTYACCATCTTCAAGGTTAGGATGTAYGTGGGGGGTGTGGAGCACAGGCTYAATGCCGCATGCAACTGGACYCGAGGAGAGCGTTGTGACTTGGAGGACAGGGACAGGTCAGAGCTTAGCCCGCTGCTGYTGTCTACAACRGAGTGGCAGGTATTGCCCTGTTCCTTCACYACCCTACCGGCTCTGTCCACCGGCTTGATCCATCTCCATCAGAACATCGTGGACGTGCAATACCTGTACGGTATAGGGTCAGCGGTTGTCTCCTACGCTATYAARTGGGAGTATGTCCTGTTGCTCTTYCTTTTCCTGGCGGACGCGCGCATCTGTGCCTGCTTGTGGATGATGCTGCTAATAGTTCAGGCTGAGGCC

>HM106619

GAGACTCGCGTGACGGGGGGGACGCAGGCCCGTACCCTCTACGGGCTTACGTCCCTCTTTTCGGTTGGGCCGTCCCAAAAACTCCAGCTTGTAAACACCAATGGCAGCTGGCACATCAACAGGACTGCCCTGAACTGCAATGACTCCCTCAACACTGGGTTCCTTGCCGCACTGTTCTACCACCACAGATTCAACTCGTCCGGATGCCCGGAGCGCATGGCCAGCTGCCGCCCCATTGACGCGTTCGCTCAGGGGTGGGGTCCCATCACTTACGCYAGCSAGCCCAGCTYGGACCAGAGGCCCTATTGTTGGCACTACGCACCYCAACCGTGCGGTATTGTRCCCGCGTCGCAGGTGTGYGGTCCAGTGTACTGTTTCACCCCAAGCCCTGTTGTRGTGGGGACGACCGAYCGKTTCGGCGTCCCTACGTATAGMTGGGGGGMGAATGAGACGGACGTGCTGCTTCTCAACAACACGCGGCCGCCGCGAGGCAACTGGTTCGGCTGCACATGGATGAATRGCACTGGGTTCACCAAGACGTGCGGGGGCCCCCCGTGCAACATCGGGGGGNNNNNNNNNNNNNNCTTGAYCTGCCCCACGGATTGCTTCCGGAAGCACCCCGAGGCCACTTACACCAAATGCGGTTCGGGGCCCTGGCTGACACCTAGGTGCATGGTTGACTACCCATACAGGCTTTGGCACTACCCCTGCACTGTCAAYTTTACCATCTTCAAGGTYAGGATGTATGTGGGGGGTGTGGAGCACAGGCTCARCGCCGCATGCAACTGGACYCGAGGAGAGCGTTGTGACTTGGAGGACAGGGATAGATCAGAGCTTAGCCCGCTGCTGCTATCTACAACAGAGTGGCAGATATTGCCCTGTTCYTTCACCACCCTACCGGCTCTRTCCACCGGCTTGATCCATCTCCATCGGAACATCGTGGACGTGCAATACCTGTACGGTATAGGGTCAGCGGTTGTCTCCTACGCAATTAAGTGGGAGTATGTCCTGTTGCTCTTCCTTTWCCTGGCGGACGCGCGCGTCTGTGYCTGCTTGTGGATGATGCTGCTAATAGCTCAGGCTGAGGCC

>HM106620

CAGACTTACACGACGGGGGGGGCGTCGGGCCGTACTACCCTCGGGTTTACGTCCCTTTTTACGTCYGGRCCGTCCCAAAAACTCCAGCTCATAAACACCAACGGCAGCTGGCACATCAACAGGACTGCCCTGAGCTGCAATGACTCCCTCAACACCGGGTTCCTTGCCGCGCTGTTCTACGCCCACAAATTYAACGCGTCCGGATGCCCGGAGCGCATGGCCAGCTGCCGCCCCATCGACAAGTTCGCTCAGGGGTGGGGTCCCATCACTTACGCCGAGCCGCCCAGCTTGGACCAGAAGCCCTATTGTTGGCACTACGCACCCCGACCGTGCGGTATCGTACCCGCGTCGCAGGTGTGCGGTCCAGTGTACTGTTTCACCCCAAGCCCTGTCGTGGTGGGGACGACCGATCGTTYCGGCGTCCCYACGTAYAGCTGGGGGGCGAATGAGACGGACGTGCTGCTTCTCAACAACACGCGGCCGCCGCGAGGCAACTGGTTCGGCTGCACATGGATGAATAGCACYGGGTTCACCAAGACGTGCGGGGGCCCCCCGTGCAACATCGGGGGGGTCGGTAATAACACCTTAMTTTGCCCCACGGATTGCTTCCGGAARCACCCCGAGGCCACYTACACCAAATGCGGCTCGGGGCCCTGGYTGACACCYAGGTGCATGGTTAACTACCCATACAGGCTATGGCACTACCCCTGCACTGTCAATTTTACCATCTTCAAGGTTAGGATGTATGTGGGGGGTGTGGAGCACAGGCTCGACGCCGCATGCAATTGGACCCGAGGAGAGCGTTGTGAYTTGGAGGACAGGGATAGATCAGAGCTTAGCCCGCTGCTGCTGTCCACAACAGAGTGGCAGGTATTGCCCTGTTCCTTCACCACCCTACCGGCTCTGTCCACCGGCTTGATCCATCTCCATCAGAACATCGTGGACGTGCAATACCTGTACGGTATAGGGTCAGCGGTTGTCTCCTACGCAATTAAGTGGGAGTATGTCCTGTTGCTCTTCCTTTTCCTGGCGGACGCGCGCGTCTGTGCCTGTTTGTGGATGATGCTGCTAATAGTTCAGGCTGAGGCC

>HM106621

GMGACTCACACGATGGGGGGGACGCAGGCCCGTGCYGCCCGCGGRWTTGTGRGCCTCTTTKCGCCTGGGCCGSSCCAAAACATCCAGCTTRTAAACACCAACGGCAGCTGGCACATCAACAGGACYGCSCTGAACTGCAAYGACTCCCTCAACACYGGGTTCATYGCCGCGCTGTTCTACTCYCGCMATTTCAACGCGTCCGGATGCCCGGAGCGCATGGCCAGCTGCCGCCCCATTGACAAGTTCGCTCAGGGKTGGGGTCCCATCACTTATGCCAAGCCGCCCAGCYTGGACCAGAAGCCCTAYTGTTGGCACTACGCACCCCAACCGTGCGGTATTGTACCMGCRTCGCAGGTGTGCGGTCCAGTGTACTGTTTCACCCCAAGCCCTGTTGTGGTGGGGACGACTGATCGTTTCGGCGTYCCTACGTATAGCTGGGGGGGGAATGAGACGGACGTGCTGCTTCTCAACAACACGCGGCCGCCGCAAGGCAACTGGTTCGGCTGCACATGGATGAATGGCACCGGGTTCACCAAGACGTGCGGGGGCCCCCCGTGCAACATCGGGGGGGACGCTAAYAACACCTTGRTCTGCCCCACAGATTGCTTCCGGAAGCACCCCGARGCCACTTACRCCAAATGCGGTTCGGGGCCCTGGCTRACACCTAGGTGCATGGTTGACTACCCATACAGGCTTTGGCACTACCCCTGCACMGTCAATTTTACCATCTTCAAGRTYAGGATGTATGTGGGGGGTGTGGAGCACAGGCTCAACGCCGCATGTAACTGGACCCGAGGRGAGCGTTGTGACTTGGAGGACAGGGATAGATCAGAGCTTAGCCCGCTGCTGCTGTCTACAACAGAGTGGCAGGTATTGCCCTGTTCCTTCACCACCCTACCGGCTCTGTCCACYGGCTTGATCCATCTCCATCGGAACATCGTGGACGTGCAATACCTGTACGGTATAGGGTCAGCGGTTGTCTCCTACGCTATTAAGTGGGAGTATATCCTGTTGCTCTTCCTYTTCCTGGCGGACGCGCGCGTCTGTGCCTGCTTRTGGATGATGCTGCTAATAGCTCAGGCTGAGGCC

>HM106622

GAGACTCGCACGATGGGGGGGGCGCAGGCCCGCACTACCNNNNNNNNNNNNNNNNNNNNNNNNNNNNNNNNNNNNNNNNNNNNNNNNNNNNNNNNNNNNNNNNNNNNNNNNNNNNNNNNNNNNNNNNNNNNNNNNNNNNNNNNNNNNNNNNNNNNNNNNNNNNNNNNNNNNNNNNNNNNNNNNNNNNNNNNNNNNNNNNNNNNNNNNNNNNNNNNNNNNNNNNNNNNNNNNNNNNNNNNNNNNNNNNNNNNNGGGTGGGGTCCCATCACTTACGCCGAGCCGCCCAGCTTGGACCAGAAGCCCTATTGTTGGCACTATGCACCCCAACCGTGCGGTATTGTGCCCGCGTCGCAGGTGTGCGGTCCAGTGTACTGTTTCACCCCAAGCCCTGTTGTGGTGGGGACGACCGACCGGTTCGGCGTCCCTACGTATAACTGGGGGGAGAATGAGACGGACGTGCTGCTTCTCAACAACACGCGGCCGCCGCGAGGCAACTGGTTCGGTTGCACATGGATGAATCGCACTGGGTTCACCAAGACGTGCGGGGGCCCCCCGTGCAACATCGGGGGGGTCGGTAATGACACCTTGATCTGCCCCACGGATTGCTTCCGGAAGCACCTCGAGGCCACTTACACCAAATGTGGTTCGGGGCCCTGGCTGACACCTAGGTGCATAGTTGACTACCCATACAGGCTTTGGCACTACCCCTGCACTGTTAACTTTACCATCTTTAAGGTTAGGATGTATGTGGGGGGTGTGGAGCACAGGCTCAGCGCCGCATGCAACTGGACCCGAGGAGAGCGTTGTGACTTGGAGGACAGGGATAGATCAGAGCTTAGCCCGTTGCTGCTGTCTACGACAGAGTGGCAGGTATTGCCCTGTTCCTTCACCACCCTACCGGCTCTGTCCACCGGCTTGATCCATCTCCATCGGAACATCGTGGACGTGCAATACCTGTACGGTATAGGGTCAGCGGTTGTCTCCTACGCAATTAAGTGGGAGTACGTCCTGTTGCTCTTCCTTTTTCTGGCGGACGCGCGCGTCTGTGCCTGCTTGTGGATGATGCTGCTAATAGCTCAGGCTGAGGCC

>HM106623

GGGGGGACTTACGCGACGKGGGCGGCGCAGGCCCGTGCTGCCAACAAGTTTACGTCCTTCTTTTCGTCTGGGCCGTCNNNNAAAATCCAGCTYATAAACACCAACGGCAGCTGGCACATCAACAGGACTGCYCTGAACTGCAATGAATCCCTCAACACYGGGTGGCTTGCTGCGCTGTTCTACACCCACAAATTYAAYGCGTCCGGATGTGYGGAGCKCATGGCCAGCTGCCGCCCCATTGACAAGTTTGCTCARGGGTGGGGTCCCATCACCCACGCTACGCCGCCCAGCTTGGACCAGAAGCCCTATTGCTGGCACTACGCACCCCGACCGTGCGGTATTGTACCCGCGTCGCAGGTGTGCGGTCCAGTGTACTGTTTCACCCCAAGCCCTGTTGTRGTGGGGACGACCGATCGTTCCGGCGTCCCTACGTATARCTGGGGGGCGAATGAGACGGACGTGCTGCTTCTCAACAACACGCGGCCGCCRCGAGGCAACTGGTTCGGCTGCACATGGATGAATGGCACTGGGTTCACCAAGACGTGCGGGGGCCCCCCGTGCAACATCGGGGGGGTCGGTAATGACACCTTGATCTGCCCCACGGATTGCTTCCGGAAGCACCCCGAGGCCACTTAYRCCARATGCGGTTCGGGGCCTTGGCTGACACCTAGGTGTATGGTTCACTACCCATACAGGCTTTGGCACTACCCCTGTACTGTCAATTTTACCATCTTCAAGGTTAGGATGTATGTGGGGGGTGTGGAGCACAGGCTCGAAGCCGCATGCAACTGGACCCGAGGAGAGCGTTGTGACCTGGAGGACAGGGATAGATCAGAACTTAGCCCGCTGCTGCTGTCTACAACAGAGTGGCAGGTATTGCCCTGTTCTTTCACCACCCTACCGGCTCTGTCCACCGGCTTGATCCATCTCCATCAGAACATCGTGGACGTGCAATACCTGTACGGTATAGGGTCAGCGGTTGTCTCCTACGCAATTAAGTGGGAGTATGTCCTGTTGCTCTTCCTTTTCCTGGCGGACGCGCGSGTCTGTGCCTGCTTGTGGATGATGCTGCTAATAGCTCAGGCTGAGGCC

>HM106624

GCGACTTACACGACGGGGGGGGCGCAGGGCCGTGCTACCCACAGCTTTRCGAGCCTCTTTTCGGCTGGGCCGTCCCAAAACATCCAGCTTATAAACACCAACGGCAGCTGGCACATCAACAGGACTGCCCTGAACTGCAANNNNNNNNNNNNNNNNNNNNNNNNNNNNNNNNNNNNNNNNNNNNNNNNNNNNNNNNNNNNNNNNNNNNNNNNNNNNNNNNNNNNNNNNNNNNNNNNNNNNNNNNNNNNNNNGGGGTGGGGTCCCATCACTTAYGCTGAYCCGCCCAGCTTGGACCAGAAGCCTTATTGTTGGCACTACGCACCCCGACCGTGCGGTATYGTACCCGCGTCGCAGGTGTGCGGTCCAGTGTACTGTTTCACTCCAAGCCCTGTTGTGGTGGGGACGACCGATMGTTTCGGCGTCCCTACGTAYAGCTGGGGGGAGAAYRAGACGGACGTGCTGCTTCTCAACAACACGCGGCCGCCGCGAGGCAACTGGTTCGGCTGCACATGGATGAATRGCACTGGGTTCACTAAGACGTGCGGGGGCCCCCCGTGCAACATCGGGGGGGTCGGTAATGACACCTTGACCTGCCCCACGGATTGCTTCCGGAAGCACCCCGAGGCCACYTAYRCCAAATGYGGTTCGGGGCCCTGGCTGACACCTAGGTGCATGGTTGACTACCCATACAGGCTTTGGCACTACCCCTGCACTSTCAATTTTACCATCTTCAAGGTTAGGATGTATGTGGGGGGTGTGGAGCACAGGCTCAACGCCGCATGCAACTGGACCCGAGGAGAGCGTTGTGACTTGGAGGACAGGGATAGATCAGAGCTTAGCCCGCTGCTGCTGTCTACAACAGAGTGGCAGGTATTGCCCTGYTCCTTCACCACCCTACCBGCTCTGTCCACCGGCTTGATCCATCTCCATCRGAACATCGTGGACGTGCAATACCTRTACGGTATAGGGTCAGCGGTTGTCTCCTACGCAATYAAGTGGGAGTATGTCCTGTTGCTCTTCCTTYTYCTGGCAGACGCGCGCATCTGTGCCTGCTTGTGGATGATGGTGCTAATAGCTCARGCTGAGGCC

>HM106625

NNNNNNNNNNNNNNNNNNNNNNNNNNNNNNNNNNNNNNNNNNNNNNNNNNNNNNNNNNNNNNNNNNNNNNNNNNNNNNNNNNNNNNNNNNNNNNNNNNNNNNNNNNNNNNNNNNNNNNNNNNNNNNNNNNNNNNNNNNNNNNNNNNNNNNNNNNNNNNNNNNNNNNNNNNNNNNNNNNNNNNNNNNNNNNNNNNNNNNNNNNNNNNNNNNNNNNNNNNNNNNNNNNNNNNNNNNNNNNNNNNNNNNNNNNNNNNNNNNNNNNNNNNNNNNNNNNNNNNNNNNNNNNNNNNNNNNNNNNNNNNNNNNNNNNNNNNNNNNNNNNNNNNNNNNNNNNNNNNNNNNNNNNNNNNNNNNNNNNNNNNNNNNNNNNNNNNNNNNNNNNNNNNNNNNNNNNNNNNNNNNNNNNNNNNNNNNNNNNNNNNNNNNNNNNNNNNNNNNNNNNNNNNNNNNNNNNNCGTGCTGTTTCTCAATAACACGCGTCCGCCGCGAGGCAACTGGTTCGGCTGCACATGGATGAATAGCACTGGGTATACCAAGACGTGCGGGGGCCCCCCGTGCAACATCGGGGGGGTCGGCAATAACACCTTAATCTGCCCCACGGAYTGYTTCCGGAAGCACCCCGAGGCCACTTACACCAAATGCGGTTCGGGGCCYTGGCTGACACCTAGGTGCATGGTTGATTACCCATACAGGCTTTGGCACTACCCCTGCACCGTCAAYTTTACCATCTTCAAGGTTAGGATGTATGTGGGGGGCGTGGAGCACAGGCTCAACGCCGCGTGCAACTGGACCCGAGGGGAGCGTTGTGACTTGGCGGACAGGGATAGATCAGAGCTTAGCCCGCTGCTGCTGTCTACAACAGAGTGGCAGATATTGCCCTGTTCCTTCACCACCCTACCGGCTCTGTCCACCGGCTTGATCCATCTCCATCGGAACATCGTGGACGTGCAATACCTGTACGGTATAGGGTCAGCGGTTGTCTCCTACGCAATTAAGTGGGAGTATGTCCTGTTGCTCTTCCTTTTCCTGGCGGACGCGCGCGTCTGTGCCTGCTTGTGGATGATGCTGCTAATAGCTCAGGCTGAGGCC

>HM106626

SGGRRCACTTAYACGACRGGGGGGAMGGCGGCCYWWACTACCARAGGGYTTRYSAGYYTCYTTARCCYTGGGCCGTCCCAAAAGATCCAGCTTGTAAAYACCAACGGCAGCTGGCACATCAACAGGACTGCCCTGAAYTGCAATGARTCCCTCAMCACCGGGTGGCTTGCCGCGCTGTTCTACACCMACAAATTCAACTCGTCCGGATGCCCGGAGCGCRTGGCCAGCTGCCGCCCCATTGACAAGTTCGYTCAGGGGTGGGGTCCCATCACTTAYGCTGAGCCGCCCGGCTYGGACCAGARGCCYTATTGTTGGCACTACGCRCCCCAACCATGCGGCATTGTACCCGCGTCGCAGGTGTGCGGTCCAGTGTACTGTTTCACCCCAAGYCCYGTTGTGGTGGGGACGACCGATCGTYTCGGCGYCCCTACGTACAGCTGGGGGGAGAATGAGACGGACGTGCTGCTTCTCAACAACACGCGGCCGCCGCGAGGCAACTGGTTCGGCTGCACATGGATGAACRGCACTGGGTTCACCAAGACGTGCGGGGGCCCCCCGTGCAACATCGGGGGGGCCGGTAATGAYACCTTGAYTTGCCCCACGGATTGTTTCCGGAAGCACCCCGAGGCCACTTACACCAAGTGCGGTTCGGGGCCTTGGCTGACACCTAGGTGCATGGTTGACTAYCCATACAGGCTTTGGCACTACCCCTGCACTGTCAATTTTACCATCTTCAAGGTYAGGATGTACGTGGGGGGCGTGGAGCACAGGCTTRMCGCCGCATGCAACTGGACCCGAGGCGAGCGTTGTGACTTGGAGGACAGGGATAGATCAGAGCTTAGCCCGCTGCTGCTGTCTACAACAGAGTGGCAGGTGYTACCCTGCTCCTTCACCACCCTACCGGCTCTGTCCACCGGCTTGATCCACCTCCATCGRAACATCGTGGACGTGCAATACCTGTACGGTATAGGGTCAGCGGTTGTCTCCTACGCAATTAAGTGGGAGTATGTCCTGTTGCTCTTCCTTTTCCTGGCAGACGCGCGCGTCTGTGCCTGCTTGTGGATGATGCTGCTAATAGCTCAGGCTGAGGCC

>HM106627

NNNNNNNNNNNNNNNNNNNNNNNNNNNNNNNNNNNNNNNNNNNNNNNNNNNNNNNNNNNNNNNNNNNNNNNNNNNNNNNNNNNNNNNNNNNNNNNNNNNNNNNNNNNNNNNNNNNNNNNNNNNNNNNNNNNNNNNNNNNNNNNNNNNNNNNNNNNNNNNNNNNNNNNNNNNNNNNNNNNNNNNNNNNNNNNNNNNNNNNNNNNNNNNNNNNNNNNNNNNNNNNNNNNNNNNNNNNNNNNNNNNNNNNNNNNNNNNNNNNGGGGTCCCATCACCCACGCTACGCCGCCCAGCTTGGACCAKAAGCCCTATTGCTGGCACTACGCACCCCGACCGTGCGGTATTGTACCCGCRTCGCAGGTGTGCGGYCCAGTGTACTGTTTCACCCCAAGCCCTGTTGTAGTGGGGACGACCGATCGTTCCGGCGTCCCTACGTATAACTGGGGGGCGAATGAGACGGACGTGCTGCTTCTCAACAACACGCGGCCGCCGCGAGGCAACTGGTTCGGCTGCACATGGATGAATGGCACTGGGTTCACCAAGACGTGCGGGGGYCCCCCGTGCAACATCGGGGGGRYCGGTAATGACACCTTGATCTGCCCCACGGATTGCTTCCGGAAGCACCCCGAGGCCACWTAYRCCAAATGCGGTTCGGGGCCTTGGCTRACACCTAGGTGYATGGTTSACTACCCATACAGGCTTTGGCACTACCCCTGTACTGTCAATTTTACCATCTTCAAGGTTAGGATGTATGTGGGGGGTGTRGARCACAGGCTCRMVGCCGCATGCAACTGGACYCGAGGAGAGCGTTGTGACCTGGAGGACAGGGATAGATCAGAACTTAGCCCGCTGCTGCTGTCTACRACAGAGTGGCAGGTATTGCCCTGTTCYTTCACCACCCTACCGGCTCTGTCCACCGGCTTGATCCATCTCCATCAGAACATCGTGGACGTGCAATACCTGTACGGTATAGGGTCAGCGGTTGTCTCCTACGCAATYAAGTGGGAGTATGTCCTGTTGCTCTTCCTTTTCCTGGCGGACGCGCGCGTCTGTGCCTGCTTGTGGATGATGCTGCTAATAGCTCAGGCTGAGGCC

>HM106628

ACGACTTACGTGACGGGGGGGGCGCAGGCCCATACTACCAGCGTGTTCGCGTCCCTCCTTACGCAAGGGCCGTCCCAAAAAATCCAGCTTGTAAACACCAACGGCAGCTGGCACATCAACAGGACTGCCCTGAACTGCAATGACTCCCTCAAAACTGGGTTCATTGCCGCGCTGTTCTACGCYCACAGATTCAACGCGTCCGGATGCCCGGAGCGCATGGCCAGCTGCCGCCCCATTGACAAGTTCGCTCAGGGGTGGGGYCCCATCACTTACGATGGGYCGCCCAGCCCGGACCAGAAGCCCTATTGTTGGCATTACGCACCCMAACCGTGCGGTATTGTACCCGCGTYGCAGGTGTGCGGCCCAGTGTACTGTTTCACCCCAAGCCCTGTTGTGGTGGGGACGACCGAYCGGTTCGGTGTCCCTACGTATARCTGGGGGGAGAATGAAACGGACGTGCTGCTTCTYAACAACACGCGRCCGCCGCGAGGCAACTGGTTCGGCTGCACATGGATGAATGGCACTGGGTTCACCAAGACGTGCGGGGGCCCCCCGTGCAACATCGGGGGGGTCGGTAATAACACCTTGACCTGCCCCACGGATTGCTTCCGGAAGCACCCCGAGGCCACTTACACCAGATGCGGTTCGGGGCCCTGGCTGACGCCTAGGTGCATGGTTCATTACCCATACAGGCTTTGGCAYTACCCCTGCACTGTCAAYTTTWCCATCTTCAARGTTAGGATGTATGTAGGGGGTGTGGAGCACAGGCTCGAAGCTGCATGCAACTGGACCCGAGGAGAGCGTTGTGACTTGGAGGACAGGGATAGATCAGAGCTTAGCCCGCTGCTGCTGTCTACAACAGAGTGGCAGGTATTGCCCTGTTCCTTCACCACCCTACCGGCTCTGTCCACCGGCTTGATCCAYCTCCATCGRAACAYCGTGGACGTGCAATACCTGTAYGGTATAGGGTCRGCGGTTGTCTCCTAYGCAATTAAGTGGGAGTATGTCCTGTTGCTCTTCCTTTTCCTGGCGGACGCRCGCGTCTGTGCCTGCTTGTGGATGATGCTGCTAATAGCTCAGGCTGAGGCC

>HM106629

GMGACTTACACGACGGGAGGGGCGCAAGCCCGCAMYRTCCRCGGGTTYACGAGCCTCTTYACGTCTGGGCCGTCCCAAAAMMTCCAGCTTATAAACACCAACGGCAGCTGGCACATCAACAGGACTGCCCTGAACTGCAATGACTCCCTCAACACCGGGTTCCTTGCCGCGCTGTTCTACGTCCACMATTTCAACGCGTCCGGATGCCCGGAGCGCATGGCCAGCTGYCGCCCCATTGACAAGTTCGCYCAGGGGTGGGGTCCCATCACTTACGCTAAGCCGGCCAGCTTGGACCAGAAGCCCTACTGTTGGCACTACGCGCCCCAGCCGTGCGGTATTGTACCCGCGGCGCAGGTGTGCGGTCCAGTGTACTGTTTCACCCCGAGCCCTGTTGTGGTGGGGACGACCGATCGTTTCGGCGTCCCTACGTACAGCTGGGGGGAGAATGAGACGGACGTGCTGCTTCTCAACAACACGCGGCCGCCGCGAGGCAACTGGTTCGGCTGCACATGGATGAATAGCACTGGGTTCACCAAGACGTGCGGGGGCCCCCCGTGCAACATCGGGGGGGTCGGTAATGACACCTTGATCTGCCCCACGGATTGCTTCCGGAAGCACCCCGAGGCCACTTACACCAAATGCGGTTCGGGGCCCTGGCTGACACCTAGGTGCATGGTTGACTACCCATACAGGCTTTGGCACTACCCCTGCACTGTCAATTTTACCATCTTCAAGGTTAGGATGTACGTGGGGGGTGTGGAGCACAGGCTCAACGCCGCATGCAACTGGACCCGAGGAGAGCGTTGTGACTTGGAGGACAGGGATAGATCAGAGCTTAGCCCGCTACTGCTGTCCACGACAGAGTGGCAGGTATTGCCCTGTTCCTTCACCACCCTACCGGCTCTGTCCACCGGCTTGATCCATCTCCATCGGAACATCGTGGACGTGCAATACCTGTACGGTATAGGGTCAGCGGTTGTCTCCTACGCAATAAAGTGGGAGTATGTCCTGTTGCTCTTCCTTTTCCTGGCGGACGCGCGCGTCTGTGCCTGCTTGTGGATGATGCTGCTAATAGCTCAGGCTGAGGCC

>HM106630

RKGACYCACRTRACGGGGGGGACGCAGRGCCGYGCTGCCAGCGGGYTTGCGRGCCTCTTTACGTCTGGGCCGTCCCAAAAAATTCAGCTTGTAAACACCAACGGCAGCTGGCACATCAACAGGACTGCCCTGAACTGCAATGACTCCCTCAACACTGGGTTCCTTGCCGCGCTGTTCTACACCCGCAACTTCAACGCGTCCGGATGCCCGGAGCGCTTGGCCAGCTGCCGCCCCATTGACAAGTTCGATCAGGGGTGGGGTCCCATCACTTACGCTGAGCCGGCCAGCTTGGACCAGAAGCCCTATTGCTGGCACTACGCACCCCAACCGTGTGGTATYGTACCCGCGTCGCAGGTGTGTGGTCCAGTGTACTGTTTCACCCCAAGCCCTGTTGTGGTGGGGACGACCGATCGTTTCGGCGTCCCTACGTATAACTGGGGGGAGAATGAGACGGACGTGCTGCTTCTCAACAACACGCGGCCGCCGCAAGGCAACTGGTTCGGCTGCACCTGGATGAATGGYACTGGGTTCACCAAGACGTGCGGGGGCCCCCCGTGCAACATCGGGGGGGYCGGTAATRACACCTTGATCTGCCCCACGGACTGCTTCCGGAAGCACCCCGAGGCCACYTACACCAAATGCGGWTCGGGGCCYTGGCTGACGCCTAGGTGCATGGTTGACTACCCATACAGGCTTTGGCACTACCCCTGCACTGTCAATTWTACCATCTTCAAGRTYAGGATGTAYGTGGGGGGTGTGGAGCACAGGCTCAACGCCGCATGCAACTGGACCCGAGGAGAGCGTTGTGACYTGGAGGACAGGGATAGATCAGAGCTYAGCCCGCTGCTRCTGTCTACRACAGAGTGGCAGGTATTGCCCTGTTCCTTCACCACCCTACCGGCTCTGTCCACCGGCTTGATCCATCTCCACCGRAACATCGTGGACGTGCAATAYCTGTACGGTATAGGGTCAGCGGTTGTCTCCTACGCAATTAAGTGGGAGTATGTCCTGTTGCTCTTCYTTTTCCTGGCGGACGCGCGCGTCTGYGCCTGCTTGTGGATGATGCTGCTAATAGCTCAGGCTGAGGCC

>HM106631

GAGACCCACGTGACGGGGGGTCGCAGGGCCGTGCTGCCTTCAGTCTYACGTCCCTCTTTTCGCCCGGGGCGTCCCAGAAAATCCAGCTCATAAACACCAATGGCAGCTGGCACATCAACAGGACTGCCCTGAACTGCAATGACTCCCTCAAGACTGGGTTCATCGCCGCGCTGTTCTACACCCACAGATTCAACGCGTCCGGATGCCCGGAGCGCATGGCCAGCTGCCGCCCCATTGACAAGTTCGCTCAGGGGTGGGGTCCCATCACYTAYGCTAAGCCGCCCAGCTTGGACCAGAAGCCCTATTGTTGGCACTACGCACCCCAACCGTGCAGTATTGTGCCCGCGTCGCAGGTGTGCGGTCCAGTGTACTGTTTCACTCCAAGCCCTGTTGTGGTGGGGACGACCGATCGTCTCGGCGTCCCTACGTATAGCTGGGGGGAGAATGAGACGGACGTGCTGCTTCTCAACAACACGCGGCCGCCGCGAGGCAACTGGTTCGGCTGTACATGGATGAATAGCACTGGGTTCACCAAGACGTGCGGGGGCCCCCCGTGCAACATCGGGGGGGTCGGTAATAACACCTTGACCTGCCCCACGGATTGYTTCCGGAAGCACCCCGAGGCCACTTACACCARATGCGGTTCSGGGCCCTGGCTGACACCTAGGTGCATGGTTGACTACCCATACAGGCTTTGGCACTACCCCTGCACTGTCAATTTTACCATCTTCAAGGTTAGGATGTATGTGGGGGGTGTGGAGCACAGGCTYAAYGCCGCATGCAACTGGACCCGAGGAGAGCGTTGTGAYTTGGAGGACAGGGATAGATCAGAGCTYAGCCCGCTGCTGCTGTCTACAACAGAGTGGCAGGTATTGCCCTGTTCCTTCACCACCCTACCGGCTCTGTCCACCGGCTTGATCCATCTCCATCGGAACATCGTGGACGTGCAATACCTGTACGGTATAGGGTCAGYGGTTGTCTCCTACKCAATCAAGTGGGAGTACGTCCTGYTGCTCTTCCTTTTCCTGGCGGACGCGCGCGTCTGTGCCTGCTTGTGGATGATGCTGCTAATAGCTCARGCTGAGGCY

>HM106632

GRGACTCACGTGACGGGGGSGDYACAGGSCCGTACTGTCCGCGGGTTTACGTCCYTCTTTTCSCYYGGGCCGTCCCAGCGCATCCAGCTTRTAAACACCRACGGCAGCTGGCACATCAACAGGACTGCCCTGAACTGCAAYGAYACCCTCCACACTGGGTTCCTTGCCGCGTTGTTCTACGYCAACARATTCAACGCGTCCGGATGCCCGGAGCGCATGGCCAGCTGCCGCCCCMTTGACAAGTTYGCTCAGGGGTGGGGTCCCATCACYCAYGTTGTGCCACCCCGCTTGGACCAGAAGCCYTATTGCTGGCACTACGCACCCCAACCGTGCGGTATTGTRCCCGCGTCGCAGGTGTGCGGTCCAGTGTACTGTTTCACCCCAAGCCCTGTTGTRGTGGGGACGACCGATCGRYTCGGCGTCCCTACGTATAGCTGGGGGGAGAATGAGACRGACGTGCTGCTCCTCAACAGCACGCGGCCGCCGCAAGGCAGCTGGTTCGGCTGCACATGGATGAATGGCACTGGGTTCACCAAGACGTGCGGGGGCCCCCCGTGCAACATCGGGGGRGTCGGTAACGACACCTTGATCTGCCCCACGGATTGCTTCCGGAAGCACCCSGARGCCACTTACACCAAATGCGGTTCGGGGCCCTGGCTRACACCTAGGTGCATGGTTGACTACCCATACAGGCTTTGGCAYTACCCCTGCACTGTCAAYTTTWCCATCTTCAMGGTTAGGATGTATGTGGGGGGTGTGGAGCACAGGCTCAAAGCCGCATGCAACTGGACCCGAGGAGAGCGTTGTGACTTGGAGGACAGGGATAGATCAGAGCTTAGCCCGCTGCTGCTGTCTACAACAGAGTGGCAGGTATTGCCCTGCTCCTTCACCACCCTACCGGCTCTGTCCACCGGCTTGATCCACCTCCACCGGAACATCGTGGACGTGCAATACCTGTACGGTATAGGGTCAGCGGTTGTCTCCTACGCAATCAAGTGGGAGTATGTCCTGTTGCTCTTCCTTTTCCTGGCGGACGCGCGCRTYTGTGCCTGCTTGTGGATGATGCTGCTAATAGCTCAGGCTGAGGCC

>HM106633

NNNNNNNNNNNNNNNNNNNNNNNNNNNNNNNNNNNNNNNNNNNNNNNNNNNNNNNNNNNNNNNNNNNNNNNNNNNNNNNNNNNNNNNNNNNNNNNNNNNNNNNNNNNNNNNNNNNNNNNNNNNNNNNNNNNNNNNNNNNNNNNNNNNNNNNNNNNNNNNNNNNNNNNNNNNNNNNNNNNNNNNNNNNNNNNNNNNNNNNNNNNNNNNNNNNNNNNNNNNNNNNNNNNNNNNNNNNNNNNNNNNNNNNNNNNNNNNNNNNNNNNNNTCACTCACGTTAAGCCGCCCAGCTTGGACCAGAAGCCYTATTGTTGGCACTACGCACCCCAACCGTGCGGTATTGTACCYGCGTCGAAGGTGTGCGGTCCAGTGTACTGTTTCACCCCAAGCCCTGTTGTGGTGGGGACGACCGATCGTTCCGGCGTCCCTACATATAGCTGGGGGGGGAATGAGACGGACGTGCTGCTTCTCAACAACACGCGGCCGCCGCGAGGCAACTGGTTCGGCTGCACATGGATGAATGGCACTGGGTTCACCAAGACGTGTGGGGGCCCCCCGTGCAATATCGGGGGGGTTGGTAACGACACCTTGATCTGCCCCACGGATTGCTTCCGGAAGCACCCCGAGGCCACTTATACCAAATGCGGTTCGGGGCCCTGGCTRACACCYAGGTGCATGGTTCACTACCCATACAGGCTTTGGCACTACCCCTGCACTGTCAATTTYACCATCTTCAAGGTTAGRATGTATGTTGGGGGTGTGGAGCACAGGCTCGAAGCCGCATGCAACTGGACCCGAGGAGAGCGTTGTAATTTGGAGGACAGGGATAGATCAGAGCTTAGCCCGCTGCTGCTGTCTACAACAGAGTGGCAGGTATTGCCCTGTTCCTTCACCACCCTACCGGCTCTGTCCACCGGCTTGATCCATCTCCATCAGAACATTGTGGACGTGCAATACCTGTACGGTATAGGGTCAGCGGTYGTCTCCTACGCAATTAAGTGGGAGTATGTCCTGTTGCTCTTCCTTTTCCTGGCGGACGCGCGCGTCTGTGCCTGCTTGTGGATGATGCTGCTAATAGCTCARGCTGAGGCC

>HM106634

AGCACTTACGTGACGGGGGGGACGGCGGCCTTTACTACCAACAGTTTTGTGGCCCTCTTAAACCCTGGGCCGGCTCAAAACATCCAGCTTGTAAACACCAACGGCAGCTGGCACATCAACAGGACCGCCCTGAACTGCAATGACTCCCTCAGAACCGGGTTCCTTGCCGCGCTGTTCTACAGGAACAAATTCAACGCGTCCGGATGCCCGGAGCGCATGGCCAGCTGCCGCCCCCTTGACAAGTTTGCTCAGGGGTGGGGTCCCATCACTTACGCCAAGCCGGCCAGATTGGACCAGAAGCCCTATTGCTGGCACTACGCACCCCAACCGTGCGGTATTGTACCCGCGTTGGAGGTGTGCGGTCCAGTGTACTGTTTCACCCCAAGCCCTGTTGTGGTGGGGACGACTGATCATCGCGGCGTCCCTACGTATAACTGGGGGGGGAATGTGACGGACGTGCTGCTTCTCAACAACACGCGACCGCCGCGAGGCAACTGGTTCGGCTGTACATGGATGAATAACACTGGGTTCACCAAGACGTGCGGGGGCCCCCCGTGCAACATCGGGGGGGTCGGTAAACCCCCCCTGATCTGCCCCACGGATTGCTTCCGCAAACACCCCGAGGCCACTTACACCAAATGCGGCTCGGGGCCCTGGTTGACACCTAGGTGCATGGTTGACTATCCATACAGGCTCTGGCACTACCCCTGCACTGTCAATTTTACCATCTTTAAGGTTAGAATGTATGTGGGGGGTGTGGAGCACAGGCTCCACGCCGCATGCAACTGGACCCGAGGAGAGCGTTGCGACTTGGAGGACAGAGATAGATCAGAGCTTAGCCCGCTACTGCTGTCTACGACAGAGTGGCAGGTATTGCCCTGTTCCTTCACCACCCTACCGGCCCTATCCACCGGCTTGATCCATCTCCATCAGAACACCGTGGACGTGCAATACCTGTACGGTATAGGGTCAGCGGTTGTCTCCTACGCAATTAAGTGGGAGTATGTTTTGTTGCTCTTCCTTTTCCTGGCGGACGCGCGCGTCTGTGCCTGCCTGTGGATGATGCTGCTAATAGTTCAGGCTGAGGCC

>HM106635

TCCACTTACGTGACGGGGGGGTCGCAGGGCCGTACTATCCACAGCCTTACGAGCTTCTTCAGGTCAGGGCCGTCCCAGAAAATCCAGCTTGTAAACACCAACGGCAGCTGGCACATCAACAGGACTGCCCTGAACTGCAATGACTCCCTCAGCACTGGGTTCATTGCYGCGCTGTTCTACACCCACAGCTTCAACGCGTCCGGATGCCCAGAGCGCATGGCTAGCTGCCACCCCATTGACAGGTTTGCTCAGGGGTGGGGTCCCATCACTTATGCTGAGCCGTCCAGCTTGGACCAGAGGCCCTATTGTTGGCACTACGCGCCCAAACCGTGCGGTATTGTACCCGCGGCGCAGGTGTGCGGCCCAGTGTACTGTTTCACCCCGAGCCCTGTTGTGGTGGGGACGACCGATCGGTYCGGCGTCCCTACGTATAGCTGGGGGGAGAATGAGACGGATGTGCTGCTTCTCAACAGCACGCGGCCGCCGCAAGGCRCATGGTTCGGCTGCACATGGATGAATRGCACTGGGTTCACCAAGACGTGCGGRGGCCCCCCGTGCAACATCGGGGGGGTCGGTAATAACACCTTGATCTGCCCCACGGATTGCTTCCGGAAGCACCCCGAGGCCACTTACACCAAATGCGGTTCGGGGCCYTGGCTGACACCTAGGTGCATRGTTGACTACCCATACAGGCTTTGGCAYTACCCCTGCACTGTCAATTTYACCATCTTCAAGRTTAGGATGTATGTGGGGGGTGTGGAGCACAGGCTCARCGCCGCATGCAACTGGACCCGAGGAGAGCGTTGTGACTTGGAGGACAGGGATAGATCAGAGCTTAGCCCGCTGCTGCTGTCTACAACAGAGTGGCAGGTATTGCCCTGTTCCTTCACCACYCTACCGGCTCTGTCCACTGGCTTGATCCATCTCCATCAGAACATCGTGGACGTGCAATACCTGTACGGTATAGGGTCAGCGGTCGTCTCCTACGCAATTAAGTGGGAGTACGTCCTGTTGCTCTTCCTTTTCCTGGCGGACGCGCGCGTCTGYGCCTGCTTGTGGATGATGCTGCTAATAGCTCAGGCTGAGGCC

>HM106636

GTGACTCAYGTGTCGGGGGGGACGCAGGGCCGTGCTGCCAGCACGCTTACGRGCTTSTTTTCRCTTGGGCCGWCCCAAAAGATCCAGCTCATAAACACYAACGGCAGCTGGCACATCAACAGGACTGCCCTGAACTGCAATGACTCCCTCCAMACTGGGTTCCTTGCCGCGCTGTTCTACACCAAAAGCTTCAACKCRTCCGGRTGCCCGGAGCGCATGGCCAGCTGCCGCCCCATTGACAAGTTTGCTCAGGGGTGGGGTCCCATCACTTACGCTRATCCGCCCATCTTGGACCAGAAGCCCTATTGCTGGCACTACGCACCCCRACCGTGCGGTATTGTACCCGCGTCGCAGGTGTGCGGTCCAGTGTACTGTTTCACCCCAAGCCCTGTTGTGGTGGGGACGACCGATCGYTTCGGCGTCCCYACGTACAGGTGGGGGGAGAATGAGACGGACGTGCTGCTTCTCAACAACACGCGGCCGCCGCRAGGCAACTGGTTCGGCTGCACATGGATGAATAGCACTGGGTTCACCAAGACGTGCGGGGGCCCCCCGTGCAACATCGGGGGGGTCGGCAATGACACCTTGMTCTGCCCCACGGATTGCTTCCGGAAGCACCCCGAGGCCACTTAYACCAAATGCGGTTCGGGGCCCTGGCTGACACCTAGGTGCATGGTTGACTACCCATACAGGCTTTGGCACTACCCCTGCACTGTTAATTTTACCATCTTTAAGGTYAGGATGTATGTGGGGGGWGTGGAGCACAGGCTCAACGCCGCATGCAACTGGACTCGAGGAGAGCGTTGTGACCTGGAGGACAGRGATAGATCAGAGCTTAGCCCGCTGCTGCTGTCTACAACAGAGTGGCAGRTATTGCCCTGTTCCTTCACCACCCTACCGGCTCTGTCCACCGGCTTGATCCATCTCCATCGGAACACCGTGGACGTACAATACYTGTACGGTATAGGGTCAGCGGTTGTCTCCTACGCAATTAAGTGGGAGTATGTCCTGTTGCTCTTCCTYTTCCTGGCGGACGCGCGCGTCTGTGCCTGCTTGTGGATGATGCTGCTAATAGCYCAGGCTGAGGCC

>HM106637

GCRACTCACACGATGGGGGGGGTGRCGGGCCGTACYACCTTCRGSYTTACGTCCATGTTYASRCTTGGGCCGTCCCAAAASATCCAGCTTATAAACACCAACGGCAGCTGGCACATCAACAGGACTGCCCTGAACTGCAATGACTCCCTCMAAACTGGGTTCCTTGCCGCRCTGTTYTACACCMRCAGWTTCAACGCGTCCGGGTGTCCGGAGCGTWTGGCCAGCTGCCGCCCCATTGACMAATTYGCTCAGGGGTGGGGTCCCATCACTTACGCTGAGCCGTCCAGCTCGGACCAGAGGCCCTATTGTTGGCACTACGCACCCCAACCGTGCGGTATYGTACCCGCGTCGCAGGTGTGCGGTCCAGTGTACTGTTTCACCCCAAGCCCTGTTGTGGTGGGGACGACCGATCGTTTCGGCGTCCCTACGTAYAGCTGGGGGGAGAATGAGACGGATGTGCTGYTTCTCAACAACACGCGRCCGCCGCRRGGCAACTGGTTCGGCTGCACATGGATGAATRGCACTGGGTTCACCAAGACGTGCGGGGGCCCYCCGTGCAACATCGGGGGGGTCGGTAATRACACCTTGACCTGCCCCACGGATTGCTTCCGGAAGCACCCCGAGGCCACTTACACCAAATGCGGTTCGGGGCCCTGGCTRACACCTAGGTGCATGGTTGACTACCCATACAGGCTTTGGCACTACCCCTGCACTGTCAATTTYACCATCTTCAAGRTTAGGATGTATGTGGGGGGTGTGGAGCACAGGCTCAACGCCGCATGCAACTGGACCCGRGGAGAGCGTTGTGACTTGGAGGATAGGGAYAGATCAGAGCTTAGCCCGCTGCTGCTGTCTACAACAGAGTGGCAGRTYTTGCCCTGTTCCTTCACCACCCTACCGGCTCTATCCACCGGCTTGATCCATCTCCATCGRAACACCGTGGACGTGCAATACCTRTACGGTGTAGGGTCAGCGGTTGTCTCCTACGCAATTAAGTGGGAGTACGTCCTGTTGCTCTTCCTTTTCCTGGCGGACGCGCGCGTCTGTGCCTGCTTGTGGATGATGCTGCTAGTAGCTCAGGCTGAGGCC

>HM106638

CTGACYCACACGACGGGGGGGTCGGAGGCCTATAAAACCMACAAGTTTGTGAGCCTCTTTACGCGTGGGCCGGCCCAAAACATCCAACTYATAAACACCAACGGCAGYTGGCACATCAACAGGACTGCCCTGAACTGCAACGACTCTCTCAGCACTGGGTTCATTGCCGCGYTGTTCTACGCCAACAAATTCAAYTCGTCCGGGTGCTCGGAGCGCATGGCCAGCTGCCGCCCCATTGACAAGTTCGCTCAGGGGTGGGGTCCCATCACTYACGCTAGGCCGCCCATCYTGGAYCAGAAGCCCTATTGCTGGCACTACGCACCCCAACCGTGCGGTATTGTACCCGCGGCGCAGGTGTGCGGTCCAGTGTACTGTTTCACCCCAAGCCCYGTTGTGGTGGGGACGACCGATCGTTTCGGCGTCCCTACGTACAGCTGGGGGGAGAATGAGACGGACGTGCTGCTTCTCAACAACACGCGGCCGCCGCGAGGCAACTGGTTCGGMTGCACATGGATGAATGGCACTGGGTTCACCAAGACGTGCGGGGGCCCCCCGTGCAACATCGGGGGGGTCGGTAATGACACCTTGACCTGCCCCACGGATTGCTTCCGGAAACACCCCGAGGCCACTTACACCAAATGCGGTTCGGGGCCCTGGCTGACACCTAGGTGCATGGTTGACTACCCATACAGGCTTTGGCACTACCCCTGCACTGTCAATTTTACCATCTTCAAGGTTAGRATGTATGTGGGGGGTGTGGAGCACAGGCTCAACGCCGCATGCAACTGGACCCGAGGAGAGCGTTGTGACTTGGAGGACAGGGATAGATCAGAGCTTAGCCCGCTGCTGCTGTCTACAACAGAGTGGCAGGTATTGCCCTGTTCCTTCACCACCCTACCGGCTYTGTCCACCGGCTTGATCCATCTCCAYCGGAACATCGTGGACGTGCAATACCTGTACGGTATAGGGTCAGCGGTTGTCTCCTACGCAATTAAGTGGGAGTACGTCCTGTTGCTCTTCCTTTTCCTGGCGGACGCGCGCGTCTGTGCCTGCTTGTGGATGATGCTGCTAATAGCYCAGGCTGAGGCC

>HM106639

GGGACTTACACGACGGGGGGGGCGCAGGCCCGTGCTACCCACGGGTTTACGTCCCTCTTTTCGGTTGGGCCGTCCCAAAAGATCCAGCTTATAAACACCAACGGCRGCTGGCACATCAACAGGACTGCCCTGAACTGCAATGACTCCCTCAAGACTGGGTTTCTTGCCGCGCTGTTCTACGCCAATARGTTCAACGCGTCCGGATGCCCGGAGCGCATGGCCAGCTGCCGCCCCATTGACAAGTTCGCTCAGGGGTGGGGTCCCATCACTTACACTACACCGCCCAGCTCGGACCAGAAGCCCTATTGTTGGCACTACGCACCCCGACCGTGCGGCATTGTACCCGCGTCGCAGGTGTGCGGTCCAGTGTACTGTTTCACCCCAAGCCCTGTTGTGGTGGGGACGACCGATCGTCTCGGCGTCCCTACGTATCGCTGGGGGGAGAATGAGACGGACGTGCTGCTTCTCAACAATACGCGGCCGCCGCGAGGCAACTGGTTCGGCTGYACATGGATGAATGGYACTGGGTTCACCAAGACGTGCGGGGGCCCCCCGTGCAATATCGGGGGGGTCGGTAATGACACYTTGACCTGCCCCACGGATTGYTTCCGGAAGCACCCCGAGGCCACTTACACCAAATGCGGTTCGGGGCCCTGGCTGACACCTAGGTGCATGGTTGACTACCCATACAGGCTTTGGCACTACCCCTGCACTGTCAATTTTTCCATCTTCAAGGTTAGGATGTATGTGGGGGGTGTGGAGCACAGGCTCACCGCCGCATGCAACTGGACCCGAGGAGAGCGTTGTGACTTGGAGGACAGGGATAGATCAGAGCTTAGCCCGCTGCTGCTGTCTACGACGGAGTGGCAGGTATTGCCCTGTTCYTTCACCACCCTACCRGCTCTGTCCACCGGCTTGATCCATCTCCATCAGAACGTCGTGGACGTGCAATACYTGTAYGGTATAGGGTCGGCGGTCGTCTCCTACGCAATTAAGTGGGAGTATGTCCTGTTGCTCTTCCTTTTCCTGGCGGACGCGCGCGTCTGTGCCTGCTTGTGGATGATGTTGCTAGTAGCTCAGGCTGAGGCC

>HM106640

ACRACTACGGTRGGRGGRGCGCARGCCCGTACYACCTCCTCGYTTGTGTCCMTCYTTKCGCCTGGGCCGGCCCAAMAGATCCAGCTTRTAAACASCAACGGCAGCTGGCACATCAACAGRACTGCCCTGAACTGCAATGATTCCCTCAACACCGGGTKSCTTGCCGCGCTGTTYTACACCCRSARATTCAACTCGTCYGGATGCCCGSAGCGCATGGCCAGCTGTCGCCCCMTTGACAAGTTCGCTCAGGGGTGGGGTCCCATCACTTATGCTARGCCGGCCAGCTTGGACCAGARGCCCTATTGTTGGCACTACGCACCCCAGCCGTGCGGTATTGTACCCGCGTCGCAGGTGTGCGGTCCAGTGTACTGTTTCACCCCAAGCCCTGTTGTGGTGGGGACGACCGATCGYTCCGGCGTCCCTACGTATAGCTGGGGGGAGAATGAGACGGACGTGCTGCTTCTCAACAACACGCGGCCGCCGCGAGGCAACTGGTTCGGCTGCACATGGATGAATGGCACTGGGTTCACYAAGACGTGCGGGGGCCCCCCGTGCAACATCGGGGGGGTCGGTAATGACACCTTGATCTGCCCCACGGATTGCTTCCGRAAGCACCCCGAGGCCACTTACACCARRTGCGGTTCGGGGCCCTGGCTGACACCTAGGTGCATGGTTGACTACCCATACAGGCTTTGGCACTACCCCTGCACTGTCAATTTTTCCATCTTCAAGGTTAGGATGTACGTGGGGGGTGTGGAGCACAGGCTCAACGCCGCATGCAACTGGACCCGAGGGGAGCGTTGTGACTTGGAGGACAGGGATAGATCAGAGCTTAGCCCGCTGCTGCTGTCTACAACAGAGTGGCAGATATTGCCCTGTTCCTTCACCACCCTACCGGCTCTGTCCACTGGTTTGATCCATCTCCATCAGAACACCGTGGACGTGCAATACCTRTACGGTATAGGGTCAGCGGTTGTCTCCTACGCAATTAAGTGGGAGTATGTCCTGTTGCTCTTCCTCTTCCTGGCGGACGCGCGCGTCTGCGCCTGCTTGTGGATGATGCTGCTAATAGTTCAGGCTGAGGCC

>HM106641

GAGACTCGCGTGATGGGGGGAACGGAGGCCCGTGCTGCCCACGGGATTGTGAGCCATTTCTTTTCGCGTGGGCCGGCTCAAAAGATCCAGCTTATAAATACCAACGGCAGCTGGCACATCAACAGGACTGCCCTGAACTGCAATGAGTCCCTTAGCACTGGGTGGCTTGCYGCGCTGTTCTACACCCGCAGTTTCAACTCGTCCGGATGCCCGGAGCGCATGGCCAGCTGCCGCCCCCTTGACAAGTTCGCTCAGGGGTGGGGTCCCATCACTTACACTAAGYCGCCCAGCTTGGACCAGAGGCCCTACTGTTGGCACTACGCACCCCAACCGTGCGGTATCGTACCCGCGTCGCAGGTGTGCGGTCCAGTGTACTGTTTCACCCCAAGCCCTGTTGTGGTGGGGACGACCGATCGTTTCGGCGTCCCTACGTACAGCTGGGGGGAGAATGAGACGGACGTGCTGCTTCTCAACAACACGCGGCCGCCGCGAGKTAACTGGTTCGGCTGTACATGGATGAATAGCACTGGGTTCACCAAGACGTGCGGGGGCCCTCCGTGCAACATCGGGGGGGTCGGTAATAACACCTTGATCTGCCCCACAGATTGCTTCCGGAAGCACCCCGAGGCCACTTACACCAAATGCGGTTCGGGGCCCTGGCTGACACCTAGGTGCATGGTTGACTACCCATAYAGGCTCTGGCACTACCCCTGCACTGTCAATTTTTCCATCTTCAAGGTTAGGATGTATGTGGGGGGTGTGGAGCACAGGCTCAACGCCGCATGCAACTGGACCCGAGGAGAGCGTTGCGACTTGGAGGACAGGGATAGATCAGAGCTTAGCCCGCTGCTGCTGTCTACAACGGAGTGGCAGATATTGCCCTGTTCCTTCACCACCCTACCGGCTCTGTCCACYGGTTTGATCCATCTCCATCGGAACACCGTGGACGTGCAATACCTCTACGGTATAGGGTCAGCGGTTGTCTCCTACGCGATTAAGTGGGAGTATGTCCTGTTGCTCTTCCTTTTCCTGGCGGACGCGCGCGTCTGTGCCTGCTTRTGGATGATGCTGCTGATAGTTCAGGCTGAGGCC
