# Supplementary material for: Effect of Immune Pressure on Hepatitis C Virus Evolution: Insights From a Single-Source Outbreak
Source: Hepatology. 2011 Feb;53(2):396–405. doi: 10.1002/hep.24076 (PMC3044208; doi:10.1002/hep.24076)
Supplement: Supplementary file 5 [file hep0053-0396-SD5.doc]

>HM106642

ATGGACCGGGAGGTGGCTGCATCGTGCGGAGGCGCGGTTTTTATAGGTCTGGCACTTYTGACCTTGTCACCACACTACAAAAAGTTCCTCGCCAGGCTCATATGGTGGTTACAATACTTYATCACCAGAGCCGAGGCGCACTTGCAGGTGTGGATCCCCCCCCTCAACGTTCGGGGGGGCCGCGATGCCATCATCCTCCTCATGTGTGCGATCCATCCAGAGCTAATCTTTACCATCACCAAAATYTTGCTCGCCATACTCGGCCCGCTCATGGTGCTCCAGGCCGGCATAACCAGGGTGCCGTACTTTGTGCGCGCCCATGGGCTCATTCGTGCATGCTTGCTGGTGCGGAAAGTCGCTGGGGGTCATTATGTCCAAATGGCTTTCATGAAGCTGGCCGCACTGACAGGCACGTACGTTTAYAACCATCTTACTCCTCTGCGGGACTGGGCCCACGAGGGCCTACGAGACCTCGCAGTGGCAGTTGAGCCCGTYGTCTTCTCTGACATGGAGACCAAGATCATTACCTGGGGGGCAGACACCGCGGCGTGTGGGGACATCATCTTAGGCCTGCCCGTCTCCGCTCGCAGGGGGAGGGAGATACTTTTGGGACCGGCAGATAGTCTTGAGGGGCAGGGATGGCGACTCCTT

>HM106643

ATGGACCGGGAGATGGCTGCATCGTGCGGAGGCGCGGTTTTTATAGGTCTGGCACTCYTGACCTTGTCACCACACTACAAAATGTTCCTCGCCAGGCTCATATGGTGGTTGCAATATTTTATCACCAGAGCCGAGGCRCTCTTGCAGGTGTGGATTCCCCCCCTCAACGTTCGGGGGGGCCGCGATGCCATCATCCTCCTCATGTGCGCGATCCACCCAGAACTAATCTTYACCATCACCAAAATCTTGCTCGCCATACTCGGCCCGCTCATGGTGCTCCAGGCTGGCATAACCAGGGTGCCGTACTTTGTGCGCGCCCACGGGCTCATTCGTGCATGCATGCTGGTGCGGAAAGTCGCTGGGGGTCATTATGTTCAAATGGCTCTCATGAAGCTGGCCGCACTGACAGGCACGTACGTCTATGACCATCTCACTCCRCTGCGGGACTGGGCCCACGAGGGCCTACGAGACCTTGCAGTGGCAGTTGAGCCCGTCGTCTTCTCTGACATGGAGACCAAGATCATCACCTGGGGGGCAGAYACCGCGGCGTGTGGGGACATCATCTTAGGCCTGCCCGTCTCCGCCCGCAGGGGGAGGGAGATACTTTTGGGACCRGCAGATAGTCTTGAGGGGCCRGGGTGGCGACTCCTT

>HM106644

ATGGACCGGGAGATGGCTGCATCGTGCGGAGGCGCGGTTTTTATAGGTCTGGCACTTTTGACCTTGTCACCACACTATAAAATGTTCCTCGCCAGGCTTATATGGTGGTTACAATATTTTATCACCAGAGCCGAGGCGCACTTGCAGGTGTGGATCCCCCCCCCCAACGTTCGGGGGGGCCSCGATGCCGTCATCCTCCTCATGTGCGCGATCCACCCGGAGCTGATCTTTGCTATCACCAAAATCTTGCTCGCCATACTCGGCCCGCTCATGGTGCTCCAGGCTGGCTTAACCAGGGTGCCGTACTTCGTGCGCGCCCATGGGCTCATTCGTGCATGCGTGCTGGTGCGGAAAGTCGCTGGGGGCCATTATGTCCAAATGGCTTTCATGAGGCTAGCCGCACTGACAGGTACGTACGTTTATGACCATCTTACTCCACTGCGGGACTGGGCCCACGAGGGCTTACGAGACCTTGCAGTGGCAGTCGAGCCCGTCGTCTTCTCTGACATGGAGACCAAGATCATTACCTGGGGGGCAGACACCGCGGCGTGTGGGGACATCATCCTAGGCCTGCCCGTCTCCGCCCGCAGGGGGAGGGAGATACTTTTGGGACCGGCAGATAGCCTTGAGGGGCAGGGGTGGCGACTCCTT

>HM106645

ATGGACCGGGAGATGGCTGCATCGTGCGGAGGCGCGGTTTTCGTAGGTCTGGCACTCTTGACCTTGTCACCATACTATAAATCGCTCCTCGCCAGGCTCATATGGTGGTTACAATATTTTATCACCAGAGCCGAGGCGCACTTGCAGGTGTGGATCCCCCCCCTCAACGTCCGGGGGGGCCGCGATGCCATCATCCTCCTCATGTGCGCGATCCACCCAGAGCTAATCTTTGCCATCACCAAAATCTTGCTCGCCATACTCGGCCCGCTCATGGTGCTCCAGGCTGGCATAACCAGGGTGCCGTACTTTGTGCGCGCCCATGGGCTTGTTCGTGCATGCATACTGGTGCGGGATGTCGCTGGGGGTCATTATGTTCAAATGGCCCTTATGAAGCTGGCCGCACTGACAGGCACGTACGTTTATGACCATCTTACTCCACTACGGGACTGGGCCCACGAGGGCCTACGAGACCTTGCAGTGGCAGTTGAGCCCGTCGTCTTCTCTGACATGGAGACCAAGATCATTACCTGGGGGGCAGACACCGCGGCATGTGGGGACATCATCCTAGGCCTGCCCGTCTCCGCCCGCAGGGGGAGGGAGATACTTCTGGGGCCGGCAGATAGTCTTGAGGGACGGGGGTGGCGACTCCTT

>HM106646

ATGGACCGGGARATGGCTGCATCGTGCGGAGGCGCGGTTTTYATAGGTCTGGCRCTTTTGACCYTGTCACCACACTATAAARTGYTCCTCGCYAGGCTCATATGGTGGTTRCAATATTTTATCACCAGAGCYGAGGCGCACYTGCAGGTGTGGRTCCCCCCCCTYRACGTTCGGGGGGGCCGCGATGCCATCATCCTCCTCATGTGCRCGATCCACCCAGAGCTAATCTTTRCCATCACCAAAATCTTGCTCGCCATACTCGGCCCGCTCATGGTGCTCCAGGCCAGYHTAAYCAGGGTGCCGTACTTYGTGCGCGCCCATGGGCTCATTCGYGCATGCTTGCTGGTGCGGAAAGTCGCTGGGGGYCATTATGTCCAAATGGCTYTCATGAGGCTGGCCGCRCTGACAGGCACGTACGTTTATAACCATCTTACTCCAYTGCGGGACTGGGCYCACGAGGGCCTACGAGACCTTGCAGTGGCAGTTGAGCCCGTCRTCTTCTCTGACATGGAGACCAAGATCATTACYTGGGGGGCAGACACCGCGGCGTGTGGGGACATCATCTCAGGCCTGCCCGTCTCCGCCCGYAGGGGGAGGGAGRTAYTYTTRGGRCCGGCAGATAGCCTTGAGGGSCAGGGGTGGCGACTCCTT

>HM106647

ATGGACCGGGAGATGGCTGCATCGTGCGGAGGCGCGGTTTTCATAGGTCTGGCACTTTTGACCTTGTCACCACACTATAAAGTGTTCCTCGCCAGGCTCATATGGTGGTTACARTATTTCATCACCAGAGCCGAGGCGCACCTGCAGGTGTGGATCCCCCCCCTCAACGTTCGGGGGGGCCGCGATGCCATCATCCTCCTCATGTGCGCGATCCATCCAGAGCTAATCTTTACCATCACCAAAATCTTGCTYGCCATACTCGGCCCGCTCRCRGTGCTCCAGGYTGGCATAACCAGGGTGCCGTACTTTGTGCGCGCCCATGGGCTCATTCGTGCATGCATGCTGGTGCGGCAAGTCGCYGGGGGTCATTATGTCCAAATGGCTTTCATGAGGCTGGCCGCGCTGACAGGCACGTACGTTTATGACCATCTTACTCCACTGCGGGACTGGGCCCACGAGGGCCTACGAGACCTTGCAGTGGCAGTTGAGCCCGTTGTCTTCTCTGACATGGAGACCAAGATCATCACCTGGGGGGCAGACACCGCGGCGTGTGGGGACATCATCCTAGGCCTGCCCGTCTCCGCCCGCAGGGGGAGGGAGATATTTTTGGGACCGGCAGATAGTCTTGAGGGGCAGGGGTGGCGACTCCTT

>HM106648

ATGGACCGGGAGATGGCTGCATCGTGCGGAGGCGCGGTTTTCATAGGTCTGGCATTTTTGACCTTGTCACCACAATACAAAATGTTCCTCGCCAGRCTCATATGGTGGTTACAATATTTTATCACCAGAGCCGAGGCGCACTTGCAGGTGTGGATCCCCCCCCTCAACGCTCGAGGGGGCCGCGATGCCATTATCCTCCTCATGTGTGCGATCCACCCAGAGCTAATCTTTGCCATCACCAAAATCTTGCTCGCCATACTCGGCCCGCTCATGGTGCTCCAGGCTGGCATAACCAGGGTGCCGTACTTTGTGCGCGCCCATGGGCTCATTCGTGCATGCTTGCTGGTGCGGAAGGTCGCTGGGGGTCATTATGTCCAAATGGCTTTCATGAAGCTAGCCGCACTGACAGGCACGTACGTTTATAACCATCTTACTCCACTGCGGGACTGGGCCCACGAGGGCCTACGAGACCTTGCAGTGGCAGTTGAGCCTGTCGTCTTCTCCGACATGGAGACCAAGATCATTACCTGGGGGGCAGACACCGCGGCGTGTGGGGATATCATCTTAGGCYTACCCGTCTCCGCCCGCAGGGGGAGGGAGATACTTTTGGGACCGGCAGATAGCCTTGAGGGGCAGGGGTGGCGACTCCTT

>HM106649

ATGGACCGGGAGATGGCTGCATCGTGCGGAGGCGCGGTTTTTATAGGTCTGGCACTTTTGACCTTGTCACCACACTAYAAACTGTTCCTCGCCAGGCTCATATGGTGGTTACAATATTTTATCACCAGAGCCGAGGCGCACTTGCAGGTGTGGATCCCCCCCCTCAACGTTCGGGGGGGCCGCGATGCCATCATCCTCCTCATGTGYGCGATCCACCCAGAGCTAATTTTTGTCATCACCAAAATCTTGCTCGCCATACTYGGYCCGCTCATGGTGCTCCAGGCTGGCATAACCAAGGTGCCGTACTTTGTRCGYGCCCATGGGCTCATTCGTGCATGCATGCTGGTGCGGCARGTTGCTGGGGGTCATTATGTCCAAATGGCTYTCATGAGGCTGGGCGCACTGACAGGCACGTACRTCTATGACCATCTTACCCCRCTGCGGGACTGGGCCCACGRGGGCCTACGGGACCTTGCAGTGGCAGTTGAGCCCGTCGTCTTCTCTGACATGGAGACCAAGATCATTACCTGGGGGGCAGACACCGCGGCGTGTGGGGACATCATCTTAGGCCTGCCCGTCTCCGCCCGCAGGGGGAGGGAGATACTTTTGGGACCGGCAGATAGTTTTGAGGGGCAGGGGTGGCGACTCCTY

>HM106650

ATGGACCGGGAGATGGCTGCATCGTGCGGAGGCGCGGTCTTCGTAGGTCTGGCACTTTTGACCTTGTCACCACACTATAAAGTGTTCCTCGCCAGGATCATATGGTGGCTACAATATTTCATCACCAGAGCCGAGGCGCACTTGCAGGTGTGNNNCCCCCCCCTCAACGTTCGGGGGGGCCGCGATGCCATCATCCTCCTCATGTGCGCGGTCCACCCAGAGCTAATCTTTACCATCACCAAAATCTTGCTCGCCATACTCGGCCCGCTCACGGTGCTCCAGGCCGGCATAACCAGGGTGCCGTACTTTGTGCGCGCCCATGGGCTCATTCGTGCATGCTTGCTGGTGCGGAAGGTCGCTGGGGGTCATTATGTCCAAATGGCTTTCATGAGGCTGGCCGCACTGACAGGCACGTACGTTTATGATCATCTTACTCCACTGCGGGACTGGGCCCACGACGGCCTACGAGACCTTGCAGTGGCAGTTGAGCCCGTCGTCTTCTCTGACATGGAGACCAAGATCATTACCTGGGGGGCAGACACCGCGGCGTGTGGGGACATCATCTCAGGCCTGCCCGTCTCCGCCCGCAGGGGGAGGGAGATATTTTTGGGACCGGCAGATAGTCTTGAGGGGCAGGGGTGGCGACTCCTT

>HM106651

ATGGACCGGGAGATGGCTGCATCGTGCGGAGGCGCGGTTTTTGTRGGTCTGGCACTCTTGACCTTGTCACCACACTAYAAATTGTTCCTCGCCAGGCTCATATGGTGGTTRCAATATYTTATCACCAGAGCCGAGGCGCACTTGCARGTGTGGATCCCCCCCCTCAACGTTCGGGGGGGCCGCGATGCCATCATCCTYCTCATGTGCGCGATCCACCCAGAGCTAATCTTTGCCATCACCAAAATCTTGCTCGCCATACTCGGGCCGCTCATGGTGCTCCAGGCYGGCATAACCAGGGTGCCGTACTTTGTGCGCGCCCAAGGGCTTATTCGTGCATGCTTGCTGGTGCGGAAAGTCGCYGGGGGYCATTATGTCCAAATGGCTTTCATGAGGCTAGGCGCACTGACAGGCACGTACGTTTATGACCATCTTAGTCCMCTGCGGGACTGGGCCCACGAGGGCCTACGAGACCTYGCGGTGGCAGTTGAGCCCGTYGTCTTCTCYGACATGGAGACCAAGATCATCACCTGGGGGGCAGACRCCGCGGCGTGTGGGGACATCATCCTAGGCCTGCCCGTCTCCGCCCGCAGGGGGAGGGAGATACTTCTGGGACCGGCAGATAGTCTTGAGGGACAGGGGTGGCGRCTCCTT

>HM106652

ATGGACCGGGAGATGGCTGCATCGTGCGGAGGCGCGGTTTTTATAGGTCTGGCACTTTTGACCTTGTCACCACAGTATAAAGTGTTCCTCGCCAGGCTCATATGGTGGTTACAATATTTTATCACCAGAGCCGAGGCGCACTTGCAGGTGTGGATCCCCCCCCTCAACGTTCGGGGGGGCCGCGATGCCATCATCCTCCTCATGTGTGCGATCCACCCAGAGCTAATCTTTGCCATCACTAAAGTCTTGCTCGCCATACTCGGCCCGCTCATGGTGCTCCAGGCTGGCATAACCAGGGTGCCGTACTTYGTGCGCGCCCATGGGCTCATTCGTGCATGCTTGCTAGTGCGGAAAGTCGCTGGGGGTCATTATGTCCAAATGGCTTTCATGAGGCTGGCCGCGCTGACAGGCACGTACGTTTATAACCATCTTACTCCACTGCGGGACTGGGCCCACGAGGGCCTGCGAGACCTTGCAGTGGCAGTTGAGCCCGTCGTCTTCTCTGACATGGAGACCAAGATCATTACCTGGGGGGCAGACACCGCGGCGTGTGGGGACATCATCTCAGGCCTGCCCGTCTCCGCCCGCAGGGGGAGGGAGATACTTTTGGGACCGGCAGATAGTCTTGAGGGGCAGGGGTGGCGACTCCTT

>HM106653

ATGGACCGGGAGATGGCTGCATCGTGCGGAGGCGCGGTTTTTGTRGGTCTGGCACTCTTGACCYTGTCACCACACTATAAAGAGCTCCTCGCCAGGCTCATATGGTGGTTGCAATATTTTATCACCAGAGCCGAGGCGCACTTGCAGGTGTGGRTCCCCCCCCTCAACGTTCGGGGGGGCCGCGATGCCATCATCCTCCTCATGTGCGCGATCCACCCAGAGCTAATYTTTACCATCACCAAAATCTTGCTCGCCATACTCGGCCCRCTCATGGTACTCCAGGCTGGCATAACCAGGGTGCCGTACTTTGTGCGCGCCCATGGGCTYATTCGTGCATGCTTGCTGGTGCGGAAAGTCGCTGGGGGTCATTATGTCCAAATGGCTCTTATGAGRCTGGCCGCRCTGACAGGCACGTACGTYTATGACCATCTWACTCCACTACGGGACTGGGCCCACGARGGCCTACGAGACCTYGCGGTGGCAGTTGAGCCCGTCGTCTTCTCTGACATGGAGACCAAGATCATTACCTGGGGGGCAGACACCGCGGCRTGTGGGGACATCATCCTAGGCCTGCCCGTCTCCGCCCGCAGGGGGAGGGAGATACTTCTGGGACCGGCAGATAGTCTTGAGGGACCAGGGTGGCGACTCCTT

>HM106654

ATGGACCGGGAGATGGCTGCATCGTGCGGAGGCGCGGTTTTTATAGGTCTGGCWCTTCTGACCTTGTCACCACACTATAAAGAGTTCCTCGCCAGGCTCATATGGTGGTTACAATATTTTAYCACCAGAGCCGAGGCGCACTTGCAGGTGTGGATCCCCCCCCTCAACGTTCGGGGGGGCCGCGATGCCATCATCCTCCTCATGTGCGCGATCCACCCAGAGCTAATCTTTGCCATCACCAAAATCTTGCTCGCCATACTCGGCCCGCTCATGGTGCTCCAGGCTGGCATAACCAGGGTGCCGTACTTTGTGCGCGCCCATGGGCTCATTCGTGCATGCTTGCTGGTGCGGAAAGTCRTTGGRGGTCATTATGTCCAAATGGCTCTCATGAGGCTGGCCGCRCTGACRGGCACGTACGTTTATGACCATCTTACTCCACTGCGGGACTGGGCCCACGAGGGCCTACGAGACCTTGCAGTGGCAGTTGAGCCCGTCRTCTTCTCTGACATGGAGACCAMRATCATYACCTGGGGGGCAGACACCGCGGCGTGTGGGGAYATCATCTTAGGCCTGCCCGTCTCCGCCCGCAGGGGGAGGGAGATATTCTTGGGACCGGCAGATAGTCTTGAGGGGCAGGGGTGGCGRCTCCTT

>HM106655

ATGGACCGGGAGATGGCTGCATCGTGCGGAGGCGCGGTCTTTGTAGGTCTGGCACTTTTGACCTTGTCACCACACTATAAAGTGTTCCTCGCCAGGCTCATATGGTGGTTACAATATTTYATCACCAGAGCCGAGGCGCACCTGCAGGTGTGGATCCCCCCCCTCAACGTTCGGGGGGGCCGCGATGCCATCATCCTCCTCATGTGCGCGATCCACCCGGAGCTAATCTTTGTCATCACCAAAATCTTGCTCGCCATACTTGGCCCGCTCATGGTGCTCCAGGCTGGCATAACCAGGGTGCCGTACTTTGTGCGCGCCCACGGGCTCATTCGTGCATGCTTGCTGGTGCGGAAAGTCGCTGGGGGTCATTATGTGCAAATGGCTCTCATGAGGCTGGCCGCGCTGACAGGCACGTACGTTTATGACCATCTCACTCCACTGCGGGACTGGGCCCACGAGGGCCTACGAGACCTTGCAGTGGCAGTYGAGCCCGTCGTCTTCTCTGACATGGAGACCAAGATCATCACCTGGGGGGCAGACRCCGCGGCGTGTGGGGACATCATCTCAGGCCTGCCCGTCTCCGCCCGCAGGGGGAGGGAGATATTYCTGGGACCGRCAGATGGTCTTGAGGGGCAGGGGTGGCGACTCCTT

>HM106656

ATGGACCGGGAGATGGCTGCATCGTGCGGAGGCGCGGTTTTTATAGGTCTGGCACTYTTGACCTTGTCACCACACTAYAAAATGTTCCTCGCCAGGCTCATATGGTGGTTACAATATCTTATCACCAGAGCCGAGGCGCACTTGCAAGTGTGGATCCCCCCCCTCAACGTTCGGGGGGGCCGCGATGCCATCATCCTCCTCATGTGYGYGATCCACCCAGAGCTAATCTTTRCCATCACCAAAATCTTGCTCGCCATACTYGGCCCGCTCATGGTGCTCCAGGCTGGCATAACYAGGGTGCCGTACTTTGTGCGCGCCCATGGGCTMATTCGTGCRTGCTTGCTGGTGCGGGACGTCGCTGGGGGTCATTATGTTCAAATGGCTTTYATGAGGCTGGCCGCACTGACAGGCACGTACGTTTATGACCATCTTACTCCACTGCAGAACTGGGCCCACGAGAGCCTACGAGACCTTGCAGTGGCAGTTGAGCCCGTCGTCTTCTCTGACATGGAGACCAAGATCATTACCTGGGGGGCAGACACCGCGGCGTGTGGGGACATCATCTTAGGCCTGCCCGTCTCCGCCCGCAGGGGGAGGGAGATACTTTTGGGACCGGCAGATAGTCTTGAGGGGCAGGGGTGGCGACTCCTT

>HM106657

ATGGACCGGGAGATGGCTGCATCGTGCGGAGGCGCGGTTTTTATAGGTCTGGCACTTTTGACCTTGTCACCACACTATAAAGTGTTCCTCGCCAGGCTCATATGGTGGTTACAATATTTTATCACCAGAGCCGAGGCGCACTTGCAGGTGTGGATCCCCCCCCTCAACGTTCGGGGGGGCCGCGATGCCATCATCCTCCTCATGTGCGTGATCCACCCAGAGCTAATCTTTACCATCACCAAAATCTTGCTCGCCATACTCGGCCCGCTCATGGTGCTCCAGGCTGGCATAACCAGGGTGCCGTACTTTGTGCGCGCCCATGGGCTCATTCGTGCATGCTTGCTGGTGCGGAAAGTCGCTGGGGGTCATTATGTCCAAATGGCTTTCATGAGGCTGGCCGCACTGACAGGCACGTACGTTTATGACCATCTTGCTCCACTGCGGGACTGGGCCCACGAGGGCCTACGAGACCTAGCAGTGGCAGTTGAGCCCGTCGTCTTCTCTGACATGGAGACCAAAGTCATTACCTGGGGGGCAGACACCGCGGCGTGTGGGGACATCATCTTAGGCCTGCCCGTCTCCGCCCGCAGGGGGAGGGAGATACTTTTGGGACCAGCAGATAGTCTTGAGGGGCAAGGGTGGCGACTCCTT

>HM106658

ATGGACCGGGAGRTGGCTGCATCGTGYGGAGGCGCGGTTTTTATAGGTCTGGCACTTTTGACCTTATCACCACACTATAAAATGTTCCTCGCCAGGCTCATATGGTGGTTACAATATCTTATCACCAGAGCCGAGGCGTACYTGCAGGTGTGGRTCCCTCCCCTCAACGTTCGGGGGGGCCGCGATGCCATCATCCTCCTCACGTGCGCGATCCACCCGGAGCTAATTTTTACCATCACCAAAATCTTGCTCGCCATACTCGGCCCGCTCATGGTGTTCCAGGCTGGCATAACCAGGGTGCCGTACTTTGTGCGCGCCCATGGGCTCATTCGTGCATGCTTGCTGGTGCGGAAATTCGCTGGGGGYCATTATGTCCAAATGGCTTTCATGAGGCTGGCCGCACTGACAGGCACGTACGTTTATGACCATCTTACTCCACTGCGGGACTGGGCCCACGAGGGCCTACGAGACCTTGCAGTGGCAGTTGAGCCCGTCGTCTTCTCTGACATGGAGACCAAGGTTATTACCTGGGGGGCAGACACCGCGGCGTGTGGGGACATCATCTTAGGCCTGCCCGTCTCCKCCCGCAGRGGGAGGGAGATACACTTGGGACCGGCAGATAGTCTTGAGGGGCAGGGGTGGCGACTCCTT

>HM106659

ATGGACCGGGAGATGGCTGCATCGTGCGGAGGCGCGGTTTTTATAGGTCTGGCACTTCTGACCTTGTCACCACACTATAAATTGTTCCTCGCCAGGCTCATATGGTGGTTGCAATATTTTATCACCAGAGCCGAGGCACACTTGCAGGTGTGGATCCCCCCTCTCAACGTTCGGGGGGGCCGCGATGCCATCATTCTCCTCATGTGCGCGATCCACCCAGAGCTAATCTTTGCCATCACCAAAATCTTGCTCGCCATACTCGGTCCGCTCATGGTGCTCCAGGCTGGCATAACCAGGGTGCCGTACTTCGTGCGCGCCCATGGGCTCATCCGTGCATGCTTGCTGGTGCGGAAAGTCGCAGGGGGTCATTATGTCCAAATGGCTTTCATGAGGCTGGCCGCGCTGACAGGCACGTACGTTTATGACCATCTTACTCCGCTGCGGGACTGGGCCCACGAGGGCCTACGAGACCTTGCAGTGGCAGTTGAGCCTGTCGTCNNNNNNNNNNNNNNNNNNNNNNNNNNNNNNNNNNNNGCAGACACCGCGGCATGTGGGGACATCATCTTAGGCCTGCCCGTCTCCGCCCGCAGGGGGAGGGAGATATTTTTGGGACCAGCAGATAGTCTTGAGGGGCAGGGGTGGCGACTCCTC

>HM106660

ATGGACCGGGAGATGGCTGCATCGTGCGGAGGCGCGGTTTTTATAGGTCTGGCATTTTTGACCTTGTCACCACATTATAAAGTGTTCCTCGCCAGGCTCATATGGTGGTTACAATATTTTATCACCAGAGCCGAGGCGCTCTTGCAGGTGTGGGTCCCCTCCCTCAACGTTCGGGGGGGCCGCGATGCCATCATCCTCCTCATGTGCGCGATCCACCCAGAGCTAATCTTTGCCATCACCAAGATCTTGCTCGCCATACTCGGTCCGCTTATGGTCCTCCAGGCTGGCATAACCAGGGTGCCGTACTTTGTGCGCGCCCATGGGCTCATTCGTGCATGCCTACTGGTGCGGAACGTCGCTGGGGGTCATTATGTCCAAATGGCTTTCATGAGGCTGGCCGCACTGACAGGCACGTACGTTTATGACCATCTTGCTCCGCTGCGGGACTGGGCCCACGAGGGCCTACGAGACCTTGCAGTGGCAGTTGAGCCCGTCGTCTTCTCTGACATGGAGACCAAGATCATTACCTGGGGGGCAGACACYGCAGCGTGTGGGGACATAATCTTAGGCCTACCCGTCTCCGCCCGCAGGGGGAGGGAGATATTYTTGGGACCGGCAGAYAGTCTTGAGGGGCAGGGGTGGCGACTCCTT

>HM106661

ATGGACCGGGAGATGGCTGCATCGTGCGGAGGCGCGGTATTTATAGGYCTGGCACTCTTGACCTTGTCACCACACTATAAAGTGTTCCTCGCCAGACTCATATGGTGGTTACAATATTTTATCACCAGAGCCGAGGCGCACCTGCAGGTGTGGACCCCCCCCCTCAACGTTCGGGGGGGCCGCGATGCCATCATCCTCCTCATGTGCSCGATCCACCCAGAGCTAATCTTTTCCATCACCAAAATCTTGCTCGCCATACTCGGCCCGCTCATGGTGCTCCAGGCTGGCATAACCAGGGTGCCGTACTTTGTGCGCGCCCATGGGCTCATTCGCGTATGCGCGCTGGTGCGGCAGGTCGCTGGGGGTCATTATGTCCAAATGGCTCTCATGAAACTGGCCGCACTGACAGGCACGTACGTTTATAACCATCTTACTCCACTGCGGGACTGGGCCCACGAGGGCCTACGTGACCTTGCAGTAGCAGTTGAGCCCGTCGTCTTCTCTGACATGGAGACCAAGATCATTACCTGGGGGGCAGACACCGCGGCGTGTGGGGACATCATCTTAGGCCTGCCCGTCTCCGCCCGCAGGGGGAGGGAGATACTTTTGGGACCGGCGGATAGTCTTGATGGGCAGGGGTGGCGACTCCTT

>HM106662

ATGGACCGGGAGATGGCTGCATCRTGCGGAGGCGCGGTYTTTGTAGGYCTGGCRCTTYTGACCTTGTCACCACACTAYAAATTGTTYCTCGCCAGGCTCATATGGTGGTTRCAATATTTTATCACCAGAGCCGAGGCGCACTTGCAGGTGTGGATCCCCCCCCTCAACGTTCGGGGGGGCCGCGATGCCATCATCCTCCTCATGTGTGCRATCCACCCAGAGCTAATCTTTRCCATCACCAAARTCYTGCTCGCCATACTYGGCCCGCTCATGGTSCTCCAGGCTAGCATAACCAGGGTGCCGTACTTYGTGCGCGCCCATGGGCTCATYCGTGCATGCTTGCTGGTGCGGAAAGTYGCKGGGGGTCATTATGTCCAGATGGYTYTCATGAGGCTGGCCGCAYTGACAGGCACGTACGTTTATGACCATCTTACTCCAYTGCGGGACTGGGCCCACGAGGGCYTACGAGACCTTGCRGTGGCWGTTGAGCCCGTCRTCTTYTCTGACATGGAGACCAAGATCATTACCTGGGGGGCGGACACCGCRGCGTGTGGGGACATCATCTTAGGCCTRCCCGTCTCCGCCCGCAGGGGGAGGGAGATATTTTTGGGACCGGCRGATAGYCTTGTMGGGCAGGGGTGGCGACTCCTY

>HM106663

ATGGACCGGGAGATGGCTGCATCGTGCGGAGGCGCGGTTTTTATAGGTCTGGCACTCTTGACCTTGTCACCACACTATAAAGTGTTCCTCGCCAGGCTCATATGGTGGTTACAATATTTTATCACCAGAGCCGAGGCGCACTTGCAGGTGTGGGTCCCCCCCCTCAACGTTCGGGGGGGCCGCGATGCCATCATCCTCCTCATGTGCGCGATCCACCCAGAGCTAATCTTTGCCATCACCAAAATCTTGCTCGCCATACTCGGCCCGCTCATGGTGCTCCAGGCTGGCATAACCAGGGTGCCGTACTTTGTGCGCGCCCATGGGCTCATTCGTGCATGCATGCTGGTGCGGCAAGTCGCTGGAGGTCATTATGTCCAAATGGCTTTCATGAAGCTCGCCGCGCTGACAGGCACGTACGTTTATGACCATCTCACTCCAATGCGGGACTGGGCCCACGAGGGCCTACGAGACCTTGCAGTGGCAGTTGAGCCCGTCGTCTTCTCTGACATGGAGACCAAGATCATCACCTGGGGGGCAGACACCGCGGCGTGTGGGGACATCATCTCCGGCCTGCCCGTCTCCGCCCGCAGGGGGAGGGAGATACTTCTGGGACCGGCAGATAGTCTTGAGGGGCAGGGGTGGCGACTCCTT

>HM106664

ATGGACCGGGAGATGGCTGCATCGTGCGGAGGCGCGGTTTTTATAGGTCTGGCACTTYTGACCTTGTCACCACACTATAAAGTGTTCCTCGCCAGGCTCATATGGTGGATACAATATTTTATCACCAGAGCCGAGGCGCACTTGCAGGTGTGGATCCCCCCCCTCAACGTTCGGGGGGGCCGCGATGCCATCATCCTYCTCATGTGYGCGATCCACCCAGAGCTARTTTTTGCCATCACCAAAATCCTGCTCGCCATACTYGGCCCGCTCATGGTGCTCCAGGCTGGCATAACCAGGGTGCCGTACTTTGTGCGCGCCCATGGGCTCATCCGTGCATGCTTGCTGGTGCGGAAAGTCGCTGGGGGTCAGTATGTCCAAATGGCTTTCATGCGGCTGGCYGCATTGACAGGCACGTACGTCTATGACCATCTTACACCRCTGCGGGACTGGGCCCACGAGGGCCTACGAGACCTTGCAGTGGCAGTTGAGCCCGTCGTCTTCTCTGACATGGAGACCAAGATCATCACCTGGGGGGCAGACACCGCGGCGTGTGGGGACATCATCTCAGGCCTGCCCGTCTCCGCCCGCAGGGGGAGGGAGATATTTTTGGGACCGGCAGATAGTCTTGAGGGGCAGGGGTGGCGACTCCTT

>HM106665

ATGGACCGGGAGATGGCTGCATCGTGCGGAGGCGCGGTTTTTATAGGTCTGGCACTTCTGACCTTGTCACCACACTACAAAGCGTTCCTCGCCAGGCTCATATGGTGGTTACAATACTTTATCACCAGAGCCGAGGCGCACTTGCAGGTGTGGATCCCCCCCCTCAACGTTCGGGGGGGCCGCGATGCCATCATCCTCCTCATGTGTGCGATCCACCCAGAGCTAATTTTTACCATCACCAAAATCTTGCTCGCCATACTCGGCCCGCTCATGGTGCTCCAGGCTGGCATAACCAGGGTGCCGTACTTTGTGCGCGCCCATGGGCTCCTTCGTGCATGCATGCTGGTGCGGAAAGCCGCTGGGGGTCATTATGTCCAAATGGCTCTCATGAGACTGGCCGCGCTGACAGGCACGTACGTTTATGACCATCTTACTCCRCTGCGGGACTGGGCCCACGAGGGCCTACGAGACCTTGCAGTGGCAGTTGAGCCCGTCGTCTTCTCTGACATGGAGACCAAGATCATTACCTGGGGGGCAGACACCGCGGCGTGTGGGGACATCATCTTAGGCCTGCCCGTCTCCGCCCGCAGGGGGAGGGAGATACTTTTGGGACCGGCAGATAGTCTTGAGGGGCAGGGGTGGCGACTCCTT

>HM106666

ATGGACCGGGAGATGGCTGCATCGTGCGGAGGTGCGGTTTTTCTAGGTCTGGCACTTCTGACCTTGTCACCATACTATAAACTGTTCCTCGCCAGGCTCATATGGTGGTTACAATATTTTATCACCAGAGCCGAGGCGCACTTGCAGGTGTGGATCCCCCCCCTCAACGTTCGGGGGGGGCGCGATGCCATCATCCTCCTCATGTGCGCGATCCACCCAGAGCTAATCTTTACCATCACCAAACTTTTGCTCGCCATACTCGGCCCGCTCATGGTGCTCCAGGCTGGCATAACCAGGGTGCCGTACTTTGTGCGCGCCCATGGGCTCATTCGTGCATGCTTGCTGGTGCGGAAAGTCGCTGGGGGTCATTATGTCCAAATGGCTTTCATGAGGCTGGCCGCACTGACAGGCACGTACGTTTATGACCATCTTGCTCCACTGCGGGACTGGGCCCACGAGGGCCTACGAGACCTTGCAGTGGCAGTAGAGCCCGTCGTCTTCTCTGACATGGAGACCAAGATCATTACCTGGGGGGCAGACACCGCGGCGTGTGGGGACATCATCTTAGGCCTGCCCGTCTCCGCCCGCAGGGGGAGGGAGATACTTTTGGGACCGGCAGATAGTCTTGAGGGGCAGGGGTGGCGACTCCTT

>HM106667

ATGGACCGGGARATGGCTGCATCGTGCGGAGGCGCGGTTTTYATAGGTCTGGCACTYTTGACCTTGTCACCACACTACAAAYTGTTCCTCGCCAGRCTCATATGGTGGTTACAATATTTTATCACCAGARCCGAGGCGCACTTGCAGGTGTGGATCCCCCCCCTCAACGTTCGGGGGGGCCGCGATGCCATCATCCTCCTCATGTGCGCGATCCACCCAGAGCTAATCTTTACCATCACCAAAATCTTGCTCGCCATACTCGGCCCGCTCATGGTGCTCCAGGCTGGCATAACCAGGGTGCCGTACTTTGTGCGCGCCCATGGGCTCATTCGYGCATGCTTGCTRGTGCGGAAAGTCGCTGGRGGTCATTATGTCCAAATGGCYTTCATGAGGCTGGCYGCRCTGACAGGCACGTACGTTTATAACCATCTTACTCCAMTGCRGGACTGGGCCCACGARGGCCTRCGAGACCTTGCAGTGGCAGTTGAGCCYGTCGTCTTCTCTGACATGGAGACCARGATCATTACCTGGGGGGCAGACACCGCGGCGTGTGGGGAYATCATCTTRGGCCTGCCCGTCTCCGCCCGYAGGGGGAGGGAGATACTTTTGGGACCGGCAGATAGYCTTGAGGGGCAGGGGTGGCGACTCCTT

>HM106668

ATGGACCGGGAGATGGCTGCATCGTGCGGAGGCGCGGTTTTCATAGGTCTGGCACTTCTGACCTTGTCACCACACTATAAAGTGTTCCTCGCCAGGCTCATATGGTGGTTACAATATTTTATCACCAGAGCCGAGGCGCACTTGCAGGTGTGGATCCCCCCCCTCAACGTTCGGGGGGGCCGCGATGCYATCATCCTCCTCATGTGCGTGATCCACCCAGAGCTAATCTTTACCATCACCAAAATCTTGCTCGCCATRCTCGGCCCGCTCATGGTGCTCCAGGCTGGCATAACCAGGGTRCCGTACTTTGTACGCGCCCATGGGCTCATTCGTGCATGCATGCTGGTGCGGAAAGTCGCTGGGGGCCATTATGTCCAAATGGCTYTCATGAGGCTGGCCGCGCTGACAGGCACGTACGTYTATGACCATCTTACTCCACTGCGGGACTGGGCCCACGAGGGCCTACGAGACCTTGCAGTAGCAGTTGAGCCCGTCATCTTCTCTGACATGGAGACCAAGATCATTACCTGGGGAGCAGACACTGCGGCRTGYGGGGACATCATCTCAGGCCTGCCCGTCTCCGCCCGCAGGGGGAGGGAGATATTTTTGGGACCGGCAGATAGTCTTGAGGGGCAGGGGTGGCGACTCCTT

>HM106669

ATGGACCGGGAGATGGCTGCATCGTGCGGAGGCGCGGTTTTTATAGGTCTGGCACTTTTGACCTTGTCACCACACTATAAGGTGTTCCTCGCCAGGCTCATATGGTGGTTGCAATATTTTATCACCAGAGCCGAGGCGCATTTGCAGGTGTGGATCCCCCCCCTCAACGTTCGGGGGGGCCGCGATGCCATCATCCTCCTCATGTGCGCGATCCACCCAGAGCTAATCTTCACCATCACTAAAATCTTGCTCGCCATACTCGGCCCGCTCATGGTGCTCCAGGCTGGCATAACCAGGGTGCCGTACTTTGTGCGCGCCCATGGGCTCATTCGTGCATGCTTGCTGGTGCGGCAAGTCGCTGGGGGTCATTATGTCCAAATGGCTTTTATGAAGCTGGCCGCGTTGACAGGCACGTACGTTTATGACCATCTTACTCCACTGCAGGACTGGGCCCACGAGGGCCTACGAGACCTTGCAGTGGCAGTCGAGCCCGTCGTCTTCTCTGACATGGAGACCAAGATCATCACCTGGGGGGCAGACACCGCGGCGTGTGGGGACATCATCTCAGGTCTGCCCGTCTCCGCCCGCAGGGGGAGGGAGATACTTTTGGGACCGGCAGATAGTCTTGAGGGGCAGGGGTGGCGACTCCTT

>HM106670

ATGGACCGGGAGATGGCTGCCTCGTGCGGAGGCGCGGTTTTTATAGGTCTGGCACTTTTGACCTTATCACCACACTATAAAGAGTTTCTCGCCAGGCTCATATGGTGGTTACAATATTTTATCGCCAGAGCCGAGGCGCACTTGCAGGTGTGGATTCCCCCCCTCAACGTTCGGGGGGGCCGCGATGCCATCATCCTCCTCATGTGTGCGATCCACCCAGGGCTAATCTTTACCATCACCAAAATCTTGCTCGCCATACTCGGCCCGCTCATGGTGCTCCAGGCTGGCATAATCAAGGTGCCGTACTTTGTGCGCGCCCATGGGCTCATTCGTGCATTCTTGCTGGTGCGGAAAGTCGCTGGGGGTCATTATGTCCAAATGGCTTTCATGAAGCTGGCCGCACTGACAGGCACGTACGTTTATGACCATCTTAGTCCACTGCGGGACTGGGCCCATGAGGGCCTACGGGACCTTGCAGTGGCAGTTGAGCCCGTCGTCTTCTCTGACATGGAGACCAATATCATTACCTGGGGGGCAGACACCGCGGCGTGTGGGGACATCATCTTAGGCCTGCCCGTCTCCGCCCGGAGGGGGAGGGAGATACTTTTGGGACCGGCAGATAGCCCTGAGGGGCAGGGGTGGCGGCTCCTT

>HM106671

ATGGACCGGGAGATGGCTGCATCGTGCGGAGGTGCGGTTCTAATAGGTCTGGCACTTTTGACCTTGTCACCACAATATAAAGTGTTCCTCGCCAGGCTCATATGGTGGTTGCAATATTTTATCACCAGGGTCGAGGCGCACTTGCAGGTGTGGATCCCCCCCCTTAACGTCCGGGGGGGTCGCGACGCCATCATCCTTCTCATGTGCGCGATCCACCCAGAGCTAATCTTTACCATCACCAAAATCTTGCTCGCCATACTCGGCCCGCTCATGGTGCTCCAAGCTGGCATAACCAGGGTGCCGTACTTTGTGCGCGCCCATGGGCTCATTCGTGCATGTTTGCTGGTGCGGAAAGTCGCTGGGGGTCATTATGTCCAAATGGCTTTCATGAGGCTGGCCGCACTGACAGGCACGTACGTTTACGACCATCTTGCTCCACTGCGGGACTGGGCCCACGAGGGCCTACGAGACCTTGCAGTGGCAGTTGAGCCCGTCATCTTCTCTGACATGGAGACCAAGATCATTACCTGGGGGGCAGACACCGCGGCGTGTGGGGACATCATCCTAGGCCTGCCCGTCTCCGCCCGCAGGGGGAGGGAGGTATTTTTGGGACCGGCAGATAGTCTTGAGGGGCAGGGGTGGCGACTCCTT

>HM106672

ATGGACCGGGAGATGGCTGCATCGTGCGGAGGCGCGGTTTTTATAGGTCTGGCACTTTTGACCTTRTCACCACACTATAAACTGTTCCTCGCCAGGCTCATATGGTGGTTGCAATATTTTATCACCAGAGCCGAGGCGCACTTGCAGGTGTGGATCCCCCCCCTCAACGTTCGGGGGGGCCGCGATGCCATCATCCTCCTCATGTGTGCAATCCACCCAGAGCTAATCTTTACCATCACCAAAATYTTGCTCGCCATACTCGGCCCGCTCATGGTGCTCCAGGCTGGCATAACCAGGGTGCCGTACTTTGTGCGCGCCCATGGGCTCATTCGTGCATGCTTGCTGGTGCGGAAGGTCGCTGGGGGTCATTATGTCCAAATGGCTTTCATGAGGCTGGCCGCACTGACAGGCACGTACGTTTATGACCATCTTACTCCACTGCGGGACTGGGCCCACGAGGGCCTACGAGACCTTGCAGTGGCAGTTGAGCCCGTYGTCTTCTCTGACATGGAGACCAAGATCATTACCTGGGGGGCAGACACCGCAGCGTGTGGGGACATCATCTTGGGCCTGCCCGTCTCCGCCCGCAGGGGGAGGGAGATATTTTTRGGACCGGCAGATAGTCTTGAGGGGCAGGGGTGGCGACTCCTT

>HM106673

ATGGACCGGGAGATGGCTGCATCGTGCGGAGGCGCGGTTTTTATAGGTTTGGCACTTTTGACCTTGTCACCACACTACAAAGTGTTCCTCGCCAGGCTCATATGGTGGTTACAATATTTTATCACCAGAGTCGAGGCGCACTTGCAGGTGTGGATCCCCCCCCTCAACGTTCGGGGGGGCCGCGATGCCATCATCCTCCTCATGTGCGCGATCCACCCAGAGCTGATCTTCACCATCACCAAAAACTTGCTCGCCATACTCGGCCCGCTCATGGTGCTCCAGGCTGGCATAACCAGGGTGCCGTACTTTGTGCGCGCCCATGGGCTCATTCGTGCATGCATGCTGGCGCGGAAAGTCGCTGGGGGTCATTATGTCCAGATGGCTTTCATGAGGCTGGCCGCGCTGACAGGCACGTACGTGTATGATCATCTTACTCCACTGCGGGACTGGGCCCACGAGGGCCTACGAGACCTTGCAGTGGCAGTTGAGCCCGTCGTCTTYTCTGACATGGAGACCAAGATCATCACCTGGGGGGCAGACACCGCGGCGTGTGGGGATATCATCTTAGGCCTGCCCGTCTCCGCCCGCAGGGGGAAGGAGATACTTTTGGGACCGGCAGATAGTCTTGAGGGGCAGGGGTGGCGACTCCTT

>HM106674

ATGGACCGGGAGATGGCTGCatCGTGCGGAGGCGCGGTTTTYGTAGGTCTGGCACTTTTGACCTTATCACCACACTATAAAATGTTCCTCGCCAGGCTCATATGGTGGTTACAATATTTTATCACCAGAGCCGAGGCGCACTTGCAKGTGTGGATCCCCCCCCTCAACGTTCGGGGGGGCCGCGATGCCATCATCCTCCTCATGTGCGCGATCCATCCAGAGCTAATCTTTGCCATCACCAAAATCTTGCTCGCCATACTCGGCCCGCTCATGGTGCTCCAGGCTGGCATAACCAGGGTGCCGTACTTTGTGCGCGCCCATGGRCTCATTCGTGCATGCKTGYTAGTGCGGAAAGTCGCTGGGGGTCATTATGTCCAAATGGCTCTCATGAGGCTGGCCGCATTGACAGGCACGTACGTCTATAACCATCTTACTCCACTGCGGGACTGGGCCCACRAGGGCCTACGAGACCTTGCAGTGGCAGTTGAGCCCGTCGTCTTCTCTGACATGGAGACCAAGATCATTACCTGGGGGGCAGACACCGCGGCATGCGGGGACATCATCTTAGACCTTCCCGTCTCCGCCCGCAGGGGGAGGGAGATACTYTTGGGACCGGCAGATAGTTTTGAAGGGCAGGGGTGGCGACTCCTT

>HM106675

ATGGACCGGGAGATGGCTGCATCGTGCGGAGGCGCGGTYTTTATAGGCCTGGCACTTTTGACTTTGTCACCACACTATAAAATGTTCCTCGCCAGGCTCATATGGTGGCTACAATATTTCATCACCAGAGCCGAAGCGCACTTGCAGGTGTGGNNNNNNNNNNNNNNNNNTCGGGGGGGCCGCGACGCCGTCATCCTCCTCATGTGCGCCATCCACCCAGAGCTAATCTTTGTCATCACCAAAATCTTGCTCGCCATACTCGGCCCGCTCATGGTGCTACAGGCTGGCATAACYAGGGTGCCGTACTTTGTGCGCGCCCACGGGCTCATTCGTGCATGCATGCTGGTGCGGAAGGTCGCTGGGGGTCATTATGTCCAAATGGCTTTCATGAGGCTGGCCGCACTGACAGGCACGTACGTTTATAACCATCTTACTCCGCTGCGGGACTGGGCCCACGAGGGCCTACGAGACCTTGCAGTGGCAGTTGAGCCCGTTGTCTTCTCTGACATGGAGACCAAGATCATCACCTGGGGGGCAGACACCGCGGCGTGTGGGGACATCATCTTAGGCCTGCCCGTCTCCGCCCGCAGAGGGAGGGAGATACTTTTGGGACCGGCAGATAGTCTTGAGGGGCAGGGGTGGCGACTCCTT

>HM106676

ATGGACCGAGAGATGGCTGCATCGTGCGGAGGCGCGGTTTTTGTAGGTCTGGCACTCTTGACCTTGTCACCGCACTATAAAGTGTTCCTCGCCAAGCTCATATGGTGGTTACAATATTTTATCACCAGAGCCGAGGCGCACTTGCAGGTGTGGATCCCCCCCCTCAACGCTCGGGGGGGCCGCGATGCCATCATCCTCCTCATGTGCGCGATCTACCCAGAGCTAATCTTTGCCATCACCAAAATCTTGCTTGCCATACTTGGCCCGCTCATGGTGCTCCAGGCTGGCATAACCAGGGTGCCGTACTTTGTGCGCGCCCATGGGCTCATTCGTGCATGCATGCTGGTGCGGGATGTTGCTGGGGGCCATTATGTCCAAATGGCCTTCGTGAAACTGGCCGCACTGACAGGCACGTACGTTTATGACCATCTTGCTCCATTGCGGGACTGGGCCCACAAGGGCCTACGAGACCTCGCAGTGGCAGTTGAGCCCGTTGTCTTCTCTGACATGGAGACCAAGATCATCACCTGGGGGGCAGACACCGCGGCGTGTGGGGACATCATCTTAGGCCTGCCTGTCTCCGCCCGCAGGGGGAGGGAGATACTCCTGGGACCGGCAGATAGTCTTGACGGGCAGGGGTGGCGACTCCTT

>HM106677

ATGGACCGGGAGATGGCTGCATCGTGYGGAGGCGCGGTYTTTATAGGTCTGGCACTTCTGACCTTGTCACCACACTATAAAGTRTTCCTCGCCAGGCTCATATGGTGGTTGCAATATTTTATCACCAGAGCCGAGGCGCACTTGCAGGTGTGGATYCCCCCCCTCAACGTCCGGGGGGGCCGCGATGCCATCATCCTCCTCATGTGCGYGATCCACCCAGAGCTAATCTTTACCATCACCAAAATCTTGCTCGCCATACTCGGCCCACTCATGGTGCTCCAGGCTGGCATAACCAGGGTGCCGTACTTTGTGCGCGCCCATGGGCTCATTCGTGCATGCGTGCTGGTGCGGAAAGTYGCYGGGGGTCATTATGTCCAAATGGCCCTCATGAAGCTGGCCGCGCTGACAGGCACGTACGTTTATGACCACCTTACTCCACTGCGGGACTGGGCCCACGAGGGCCTACGAGACCTTGCAGTGGCAGTTGAGCCCGTCGTCTTYTCTGACATGGAGACCAAGATCATTACCTGGGGKGCAGACACCGCGGCGTGYGGGGACATCATCTTAGGCTTKCCCGTYTCCGCYCGCAGGGGGAAGGAGATACTTTTGGGACCGGCAGATAGTCTTGAGGGGCAGGGCTGGCGACTCCTT

>HM106678

ATGGACCGGGAGATGGCTGCATCGTGCGGAGGCGCGGTTTTTATAGGTCTGGCACTTTTGACCTTGTCACCACATTATAAAGTGTTCCTCGCCAGGCTTATATGGTGGTTGCAATACTTTATCACCAGAGCCGAGGCGCACTTGCAGGTGTGGATCCCCCCTCTCAACGTTCGGGGGGGCCGCGATGCCATCATCCTCCTCATGTGCGCGATCCACCCAGAGCTAATTTTTGCCATCACCAAAATCTTGCTTGCCATACTCGGCCCGCTCATGGTGCTCCAGGCTAGCATAACCAGGGTGCCGTACTTTGTGCGCGCCCACGGGCTCATTCGTGCATGCTTGCTGGTGCGGAAGGTCGCTGGGGGTCATTATGTCCAAATGGCTCTCATAAGGCTGGCCGCGCTGACAGGTACGTACGTTTATGACCATCTTGCTCCACTGCGGGACTGGGCTCACGAGGGCCTACGAGACCTTGCAGTGGCAGTTGAGCCCGTCGTCTTCTCTGACATGGAGACCAAGATCATTACTTGGGGGGCAGACACCGCGGCGTGTGGGGACATCATCTTAGGCCTGCCTGTCTCCGCCCGCAGGGGGAGGGAGATATTTTTGGGACCGGCAGATAGTCTTGAGGGGCAGGGGTGGCGGCTCCTT

>HM106679

ATGGACCGGGAGATGGCTGCATCGTGCGGAGGCGCGGTTTTYATAGGTCTGGCACTTTTGACCTTGTCACCACACTATAAAATGTTCCTCGCCAGGTTCATATGGTGGTTACAATATTTTATCACCAGAACCGAGGCGCTCTTGCAGGTGTGGRTTCCCCCCCTCAACGTTCGGGGGGGCCGCGATGCCATCATCCTCCTCATGTGCGCGATCCACCCAGAGCTAATCTTTGCCATCACCAAAATCTTGCTCGCCATACTYGGCCCGCTCATGGTGCTCCAGGCTGGCATAACCAGGGTGCCGTACTTTGTGCGCGCCCACGGGCTCATTCGTGCATGCTTGCTGGTGCGGAAAGYCGCTGGRGGTCATTATGTCCAAATGGCTTTYATGAGGCTGGCCGCACTGACAGGCACGTACGTYTATGACCATCTTACTCCATTGCGGGACTGGGCCCACGAGGGCCTGCGAGACCTTGCAGTGGCAGTTGAGCCCGTCGTCTTCTCTGACATGGAGACCAAGATCATTACCTGGGGGGCAGACACCKCGGCGTGTGGGGAYATCATCTTAGGCCTGCCCGTCTCTGCCCGCAAGGGGAGGGAGATACTTCTRGGACCGGCAGATAGTCTTGAGGGGCAGGGGTGGCGACTCCTT

>HM106680

ATGGACCGGGAGATGGCTGCATCGTGCGGAGGCGCGGTTTTTATAGGTCTGGCACTTYTGACCCTGTCACCACACTATAAACTGTTCCTCGCCAGGCTCATATGGTGGTTACAATATCTTATCACCAGAGCCGAGGCGCACTTGCAGGTGTGGATCCCCCCCCTCAAYGTTCGGGGGGGCCGCGATGCCATCATTCTCCTCATGTGCGCGATCCACCCAGAGCTAATCTTTACCATCACCAAAATCTTGCTCGCCATACTCGGCCCGCTCACGGTGCTCCAAGCTGGCATAACCAGGGTGCCGTACTTTGTGCGCGCCCATGGCCTCATTCGTGCATGCTTGCTGGTGCGGAAAGTCGCTGGGGGTCATTATGTCCAAATGGCTTTCATGAGGCTGGCCGCTCTGACRGGCACGTACGTTTATAACCATCTTACTCCACTGCGGGACTGGGCCCACGAGGGCCTACGAGACCTTGCAGTGGCAGTTGAGCCCGTCGTCTTCTCTGACATGGAGACCAAGATCATTACCTGGGGGGCAGACACCGCGGCGTGTGGGGACATCATCTCAGGCCTGCCCGTCTCCGCCCGCAGGGGGAGGGAGATACTTTTGGGACCGGCAGATAGTCTTGAGGGGCAGGGGTGGCGACTCCTT

>HM106681

ATGGACCGGGAGATGGCTGCATCGTGCGGAGGCGCGGTTTTTATAGGTCTGGCACTTTTGACCTTGTCACCACAATATAAAGTGTTCCTCGCCAGGCTCATATGGTGGTTACAATATTTTATCACCAGAGCCGAGGCGCACTTGCAGGTGTGGATCCCCCCCCTCAACGTTCGGGGGGGCCGCGATGCCATCATCCTCCTCATGTGCGCGATCCACCCAGACCTAATCTTTACCATCACCAAAATCTTGCTCGCCATACTCGGCCCGCTCATGGTGCTCCAAGCTGGCATAACCAGGGTGCCGTACTTTGTGCGCGCCCATGGGCTCATTCGTGCATGCTTGCTGGTGCGGAAAGTCGCTGGGGGTCATTATGTCCAAATGGCTCTCATGAGGCTGGCCGCACTGACAGGCACGTACGTTTATGACCATCTTACTCCGCTGCGGGACTGGGCCCACGAGGGCCTACGAGACCTTGCAGTGGCAGTTGAGCCTGTCGTCTTCTCTGACATGGAGACCAAGATCATTACCTGGGGGGCAGACACCGCGGCGTGTGGGGACATCATCTTWGGCCTGCCCGTCTCCGCCCGCAGGGGGAGGGAGATATTTTTGGGACCGGCAGATAGTCTTGAGGGGCAGGGGTGGCGACTCCTT

>HM106682

ATGGATCGAGAGATGGCTGCATCGTGCGGAGGCGCGGTTTTTGTAGGCCTGGCACTTTTGACCTTGTCACCACACTATAAAGTGTTCCTCGCCAGGCTCATATGGTGGTTACAATATTTTATCACCAGAGCCGAGGCGCTCTTGCAGGTGTGGATTCCCCCCCTCAACGTTCGGGGGGGCCGCGATGCCATCATCCTCCTCATGTGCGTGATCCACCCAGAGCTAATCTTTACCATCACCAAAATCTTGCTCGCCATACTCGGCCCGCTCATGGTGCTCCAGGCTGGCATAACCAGGGTGCCGTACTTTGTGCGTGCCCATGGGCTCATTCGTGCATGCTTGCTGGTGCGGAAAGTCGCTGGGGGTCATTATGTTCAAATGGCTTTCATGAAGCTGGCCGCACTGACAGGCACGTACGTTTATGACCATCTTACCCCGCTGCAGAACTGGGCCCACGAGGGCCTACGAGACCTTGCTGTGGCGGTTGAGCCCGTCGTCTTCTCTGACATGGAGACCAAGGTCATTACCTGGGGGGCAGACACCGCGGCGTGTGGGGACATCATCTCAGGCCTGCCCGTCTCCGCCCGCAGGGGGAGGGAGATACTTCTGGGACCGGCAGATAGTCTTGAGGGGCAAGGGTGGCGACTCCTT

>HM106683

ATGGACCGGGAGACGGCTGCATCGTGCGGAGGCGCGGTTATTATAGGTCTGGCACTTTTGACCTTGTCACCACACTAYAAAGTGCTCCTCGCCAGGATCATATGGTGGTTACAATATTTAATCACCAGAGCCGAGGCGCTCTTGCAGGTGTGGATCCCCCCCCTCAACGTTCGGGGGGGCCGCGATGCCATCATCCTCCTCATGTGCGCGATCCACCCAGAGCTAATCTTTGCCATCACCAAAATTTTGCTCGCCGTACTCGGCCCGCTCATGGTGCTCCAGGCTGGCATAACCAGGGTGCCGTACTTTGTGCGCGCCCATGGGCTCATTCGTGCATGCATGCTGGTGCGGCAACTCGCCGGGGGTCACTATGTCCAAATGGTTTTCATGAGGCTGGCCGCACTGACAGGCACGTACGTATATGACCATCTTGCTCCACTGCGGGACTGGGCCCATGAGGGCCTACGAGACCTTGCAGTGGCAGTTGAGCCCGTCGTCTTCTCTGACATGGAGACCAAGATCATTACCTGGGGGGCAGACACCGCGGCATGTGGGGACATCATCTTAGGCCTGCCCGTCTCCGCCCGCAGGGGGAGGGAGATACTTTTGGGGCCGGCAGATAGTCTTGAGGGGCAGGGGTGGCGACTCCTT

>HM106684

ATGGACCGGGAGATGGCTGCATCGTGCGGAGGCGCGGTTTTCATAGGTCTAGCACTTTTGACCTTGTCACCACACTATAAACCGTTCCTCGCCAGGCTCATATGGTGGTTACAATAYTTTATCACCAGAGCCGAGGCRCACTTGCAGGTGTGGATCCCCCCCCTCAACGTTCGGGGGGGCCGCGATGCCATCATCCTCCTCATGTGCGCGATCCACCCRGAGCTAATCTTTGCCATCACCAAAATCTTGCTCGCCATACTCGGCCCGCTCATGGTGCTCCAGGCTGGCATAACCAGGGTGCCGTACTTTGTGCGCGCCCATGGGCTCATTCGTGCATGCTTGCTGGTGCGGAAAGTCGCCGGGGGCCATTATGTCCAAATGGCYTTCATGAGGCTGGCCGCACTGACAGGCACGTACGTTTATGACCATCTTACTCCACTGCGGGACTGGGCCCACGAGGGCCTRCGAGACCTTGCAGTGGCAGTTGAGCCCGTCGTCTTCTCTGACATGGAGACCAAGATCATCACCTGGGGGGCAGACACCGCGGCGTGTGGGGACATCATCTYGGGCCTGCCCGTCTCCGCCCGCAGGGGGAGGGAGATATTTTTGGGACCGGCAGATAGTCTTGAGGGGCAGGGGTGGCGACTCCTT

>HM106685

ATGGACCGGGAGATGGCTGCATCGTGCGGAGGCGCGGTCTTTGTGGGTCTGGCACTTTTGACCTTGTCACCACTCTACAAAATGTGCCTCGCCAGGCTCATATGGTGGTTACAATAYTTTATCACCAGAGCCGAGGCGCAYTTGCAGGTGTGGATCCCCCCCCTCAACGTTCGGGGGGGCCGCGATGCCATCATCCTCCTCATGTGCGCGATCCACCCAGAGCTAATMTTTACCATCACCAAAATYTTGCTCGCCATACTCGGCCCGCTCATGGTGCTCCAGGCTGGCATAACCAGGGTGCCATAYTTTGTGCGCGCCCATGGGCTCATTCGTGCATGCTTGCTGATGCGGAAAGTCGCTGGGGGTCATTATGTTCAAATGGCTTTCATGAGGCTGGCCGCACTGACAGGCACGTATGTTTATGACCATCTTACTCCACTGCGGGACTGGGCCCACGAGGGCCTACGAGACCTTGCAGTGGCAGTTGAGCCCGTCRTCTTTTCTGACATGGAGACTAAAATCATCACCTGGGGGGCAGACACCGCGGCGTGTGGKGACATCATCTCAGGCCTGCCCGTCTCCGCCCGCAGGGGGAGGGAGATACTTTTGGGACCGGCAGATAGTCTTGAGGGACAGGGGTGGCGACTCCTT

>HM106686

ATGGACCGGGAGATGGCTGCATCGTGCGGAGGCGYGGTTCTCATAGGTCTGGCATTTTTGACCTTGTCACCACAGTATAARGTGTTCCTCGCCAGGCTCATATGGTGGTTRCAGTACTTKATCACCAGAGCCGAGGCGCACTTGCAGGTGTGGATCCCCCCCCTCAACGTTCGGGGGGGCCGCGATGCCATCATCCTCCTCATGTGCGCGATCCACCCRGAGCTAATCTTTRCCATCACCAARATCCTGCTCGCCATACTCGGCCCGCTCATGGTGCTCCAGGCTGGCATAACCAGGGTGCCGTACTTTGTGCGCGCCCATGGGCTCATTCGTGCATGCTTGCTGGTGCGGAAAGTCGCTGGGGGTCATTATGTCCAAATGGCCTTCATGAGGCTGGCCGCACTGACAGGCACGTACGTTTATGACCATCTTACTCCRCTGCGKGACTGGGCCCACGAGGGCCTACGAGACCTTGCGGTGGCAGTCGAGCCCGTCGTCTTCTCTGACATGGAGACCAAGGTCATTACCTGGGGGGCAGACACCGCGGCGTGTGGGGACATCATCTCAGGCCTGCCTGTCTCCGCCCGCAGGGGGAGGGAGATACTTTTGGGACCGGCAGATAGCCTTGAGGGCCAGGGGTGGCGACTCCTT

>HM106687

ATGGACCGGGAGATGGCCGCATCGTGCGGAGGCGCGGTYCTTATAGGTCTGGCACTTTTGACCTTGTCACCACACTACAAAGTGTTCCTCGCCAGGCTCATATGGTGGTTGCAATATTTTATCACCAGGGCCGAGGCGCACTTGCAGGTGTGGATCCCCCCCCTCAACGTTCGGGGGGGCCGCGATGCCATCATCCTCCTCATGTGCGCGATCCACCCAGAGCTAATCTTCACCATCACCAAAATCTTGCTCGCCATACYCGGCCCGCTCCTGGTGCTCCAGGCTGGCATAACCAGGGTGCCGTACTTTGTGCGCGCCCATGGGCTCATTCGTGCATGTTTGCTGGTGCGGAAAGTCGCTGGGGGTCATTATGTCCAAATGGCTTTCATGAGGCTGGCCGCACTGACAGGCACGTACGTTTATGACCATCTTACTCCACTGCGGGACTGGGCCCACGAGGGCCTGCGAGACCTTGCAGTGGCAGTTGAGCCCGTYGTCTTCTCTGACATGGAGACCAAGATTATCACCTGGGGGGCAGACACCGCGGCGTGTGGGGACATCATCTTGGGCCTGCCCGTCTCCGCCCGCAGGGGGAGGGAGATATTTTTGGGACCGGCAGATAGCCTTGAGGGGCAGGGGTGGAGACTCCTT

>HM106688

ATGGACCGGGAGATGGCTGCATCGTGTGGAGGCGCGGTTTTTATAGGTCTAGCACTTTTGACCTTGTCACCACACTATAAAATGTTCCTCGCCAGGTTCATATGGTGGYTACAATATCTTATCACCAGAGCCGAAGCGCACTTGCAGGTGTGGATCCCCCCCCTCAACGTTCGGGGGGGGCGCGATGCCATCATCCTCCTCATGTGCGCGATCCACCCAGAGCTAATCTTTGCCATCACCAAAAWCCTGCTCGCCATACTCGGCCCGCTCATGGTGCTCCAGGCTGGCATAACCAGGGTGCCGTACTTTGTACGCGCCCATGGGCTCATTCGTGCATGCTTGCTGGTGCGGAAAGTCGCTGGGGGTCATTATGTCCAAATGGCTTTCATGAAGCTGGGCGCGCTGACAGGCACGTAYGTTTATAACCATCTTACTCCACTGCGGGACTGGGCCCACGAGGGCCTACGAGATCTTGCAGTGGCAGTTGAGCCCGTTGTTTTCTCTGACATGGAGACCAAGATCATTACCTGGGGGGCRGACACCGCGGCGTGTGGGGACATCATCTYGGGCCTGCCCGTCTCCGCCCGYAGGGGSAGGGAGATACTYYTGGGACCGGCAGATAGTCTTGAGGGGCAGGGGTGGCGACTCCTT

>HM106689

ATGGACCGGGAGATGGCTGCATCGTGCGGAGGCGCGGTTTTTATAGGTCTGGCACTTTTGACCTTGTCACCACACTATAAAGTGTTCCTCGCCAGGCTCATATGGTGGTTACAGTATCTTATCACCAGAGCTGAGGCGCACTTGCAGGTGTGGATCCCCCCCCTCAACGTTCGGGGGGGCCGCGATGCCATCATCCTCCTCATGTGCGCGATCCACCCAGAGCTAATCTTTACCATCACCAAAATCTTGCTCGCCATACTCGGCCCGCTCATGGTACTCCAGGCTGGTATAACCCGGGTGCCGTACTTTGTGCGCGCCCATGGGCTCATTCGTGCATGCTTGCTGATGCGGAAAGTCGCTGGGGGTCATTATGTCCAAATGGCTCTCATGAAGCTGGCCGCGCTGACAGGCACGTACGTTTATGACCATCTTACTCCGCTGCGGGACTGGGCCCACGAGGGCCTACGAGACCTTGCAGTGGCAGTTGAGCCCGTCATCTTCTCTGACATGGAGACCAAGATCATCACCTGGGGAGCAGACACCGCGGCGTGTGGGGACATCATCTTAGGCCTGCCCGTCTCCGCCCGTAGGGGGAGGGAGATACTTTTGGGACCGGCAGATAGTCTTGATGGGCAGGGGTGGCGACTCCTT

>HM106690

ATGGACCGGGAGATGGCTGCATCGTGCGGAGGCGCGGTTTTTATAGGTCTGGCACTTTTGACCTTGTCACCATGCTATAAAGTGTTCCTCGCCAGGCTCATATGGTGGTTACAATATTTTATCACCAGAGCCGAGGCGCACTTGCAGGTGTGGATCCCCCCCCTCAACGTGCGGGGGGGCCGCGATGCCATCATCCTCCTCATGTGCGCGATCCACCCAGAGCTAATCTTTACCATCACCAAAGTCTTGCTCGCCATACTCGGCCCGCTCATGGTGCTCCAGGCTGGCATAACCAGGGTGCCGTACTTTGTGCGCGCCCATGGGCTCATCCGTGCATGCCTGCTGGTGCGGAAAGTCGCTGGGGGTCATTATGTCCAAATGGCTCTCATGAGGCTGGCCGCGCTGACAGGCACGTACGTTTATGACCATCTTGCTCCACTGCGGGACTGGGCCCACGAGGGCCTACGAGACCTTGCAGTGGCAGTTGAGCCCGTCGTCTTYTCTGACATGGAGACCAAGATCATTACCTGGGGGGCAGACACCGCGGCGTGTGGGGACATCATCTTAGGCCTGCCCGTCTCCGCCCGCAGGGGGAGGGAGATACTTTTGGGACCGGCAGATAGTTTTGAGGGGCAGGGGTGGCGACTCCTT

>HM106691

ATGGACCGGGAGATGGCTGCATCGTGCGGAGGCGCGGTTCTYATAGGTCTGGCACTYTTGACCTTGTCACCACACTATAAAATGTTCCTYGCCMGGCTCATATGGTGGTTACAATAYTTTATCACCAGAGCYGAGGCGCACTTGCAGGTGTGGRTCCCCCCCCTCAACGTTCGGGGAGGCCGCGATGCCRTCATCCTCCTCATGTGCGCGATCCACCCAGAGCTAATCTTTGCCATCACCAAAATCTTGCTCGCCATACTCGGCCCGCTCATGGTGCTCCAGGCTGGCATAACCAGGGTGCCGTACTTTGTGCGCGCCCATGGGCTCATTCGTGCATGCTTGCTGGTGCGGAAAGTCGCTGGGGGTCATTAYGTCCAAATGGCTTTCATGAGGCTGGCCGCRCTGACAGGCACGTACGTTTATGACCATCTTACCCCACTGCGGGACTGGGCCCACGAGGGYCTACGAGACCTTGCAGTGGCAGTTGAGCCCGTCGTCTTCTCTGACATGGAGACCAAGATCATYACCTGGGGGGCAGACACCGCRGCGTGTGGGGACATCATCYTAGGCCTGCCCGTCTCCGCCCGCAGGGGGAGGGAGATACTCTTGGGACCGGCAGATAGTCTTGAGGGGCGGGGGTGGCGACTCCTT

>HM106692

ATGGACCGGGAGATGGCTGCATCGTGCGGAGGCGCGGTTTTTATAGGTCTGTCACTCTTGACCTTGTCACCACACTATAAAATGTTCCTCGCCAGGCTCATATGGTGGTTACAATATTTTATCACCAGAGCCGAGGCGCACTTGCAAGTGTGGATCCCCCCCCTCAACGTTCGGGGGGRCCGCGATGCCATCATCCTCCTCATGTGCGYGATCCACCCAGAGYTAATCTTTGCCATCACCAAGATCTTGCTCGCCATRCTCGGCCCGCTCATGGTGCTCCAGGCTGGYATAACCAGGGTGCCGTACTTTGTGCGCGCCCATGGGCTCATACGTGCATGCWTGCTGGTGCGGAAAGTCGCTGGGGGTCATTAYGTYCAAATGGCTTTCATGAGGCTGGCCGCGCTGACAGGCACGTACGTTTATGACCATCTTACTCCACTGCAGGACTGGGCCCACAAGGGCCTGCGAGACCTTGCGGTGGCAGTTGAGCCCGTCGTTTTTTCTGACATGGAGACCAAGATCATTACCTGGGGGGCAGACACCGCGGCGTGCGGGGACATCATCTYAGGCCTGCCCGTCTCCGCCCGCAGGGGGAGGGAGATACTTTTGGGACCGGCAGATAGTCTTGAGGGGCAGGGGTGGCGACTCCTT

>HM106693

ATGGACCGGGAGATGGCTGCATCGTGCGGAGGCGCGGTTTTTATAGGTCTGGCACTTTTGACCTTGTCACCATGCTATAAAGTGTTCCTCGCCAGGCTCATATGGTGGTTACAATATCTTATCACCAGAGCCGAGGCGCACTTGCAGGTGTGGATCCCCCCCCTCAACGTGCGGGGGGGCCGCGATGCCATCATCCTCCTCATGTGCGCGATCCACCCAGAGCTAATCTTTACCATCACCAAAGTCTTGCTCGCCATACTCGGCCCGCTCATGGTGCTCCAGGCTGGCATAACCAGGGTGCCGTACTTTGTGCGCGCCCATGGGCTCATTCGTGCATGCCTGCTGGTGCGGAAAGTCGCTGGGGGTCATTATGTCCAAATGGCTCTCATGAGGCTGGCCGCGCTGACAGGCACGTACGTTTATGACCATCTTGCTCCACTGCGGGACTGGGCCCACGAGGGCCTACGAGACCTTGCAGTGGCAGTTGAGCCCGTCGTCTTCTCTGACATGGAGACCAAGATCATTACCTGGGGGGCAGACACCGCGGCGTGTGGGGACATCATCTTAGGCCTGCCCGTCTCCGCCCGCAGGGGGAGGGAGATACTTTTGGGACCGGCAGATAGCCTTGAGGGGCAGGGGTGGCGACTCCTT
