# Supplementary material for: Effect of Immune Pressure on Hepatitis C Virus Evolution: Insights From a Single-Source Outbreak
Source: Hepatology. 2011 Feb;53(2):396–405. doi: 10.1002/hep.24076 (PMC3044208; doi:10.1002/hep.24076)
Supplement: Supplementary file 6 [file hep0053-0396-SD6.doc]

>HM106694

GCGCCCATCACAGCCTATGCCCAACAGACGCGGGGCCTACTTGGCTGCATYGTTACCAGCCTCACAGGCCGGGACAAGAACCAGGTCGAGGGGGAGGTTCAAGTGGTTTCTACCGCAACACAATCTTTCCTGGCGACCTGCGTCAACGGCGTGTGTTGGACTGTCTACCACGGCGCCGGCACAAAGACCCTTGCCGGCCCAAAGGGGCCAATCACCCAAATGTACACCAATGTAGACCAGGACCTCGTCGGCTGGCAAGCGCCTCCCGGGGCGCGATCCTTGACACCGTGCACCTGTGGCAGCTCGGACCTTTACTTGGTCACGAGGCATGCTGATGTCATTCCGGTGCGCCGGCGGGGCGACGGCAGAGGAAGCCTACTCTCCCCCAGGCCCCTCTCCTACTTGAAGGGCTCTTCGGGCGGTCCGCTGCTCTGCCCCTCGGGGCATGCTGTAGGCATCTTCCGGGCTGCTGTGTGCACCCGGGGGGTTGCGAAGGCGGTGGACTTTGTGCCCGTTGAGTCTATGGAAACAACTATGCGGTCCCCGGTCTTCACGGACAACTCGTCCCCCCCGGCCGTACCGCAGACATTCCAAGTGGCCCATCTACACGCTCCCACTGGCAGCGGCAAGAGCACTAAGGTGCCGGCTGCGTATGCAGCCCAAGGGTACAAGGTACTCGTCCTGAACCCGTCTGTTGCTGCTACCCTAGGTTTCGGGGCATATATGTCTAAGGCACATGGTGTCGACCCTAACATTAGAACTGGGGTAAGGACCATCACCACGGGTGCCCCCATTACGTACTCCACCTATGGTAAGTTTCTTGCCGACGGTGGATGCTCTGGGGGCGCCTATGACATCATAATATGTGATGAGTGCCACTCAACTGACTCAACTACCATCTTGGGCATTGGCACAGTCCTGGACCAAGCGGAGACGGCTGGAGCGCGACTCGTCGTGCTCGCCACCGCCACGCCTCCGGGATCGATCACCGTGCCACATCCCAATATCGAGGAGGTGGCTCTGTCCAACGATGGAGAGATCCCCTTCTATGGTAAAGCCATCCCCATCNNNNNNNNNNNNNNNNNNNNNNNNNNNNNNNNNNNNNNNNNNNNNNNNNNNNNNNNNNNNCTCGCCGCCAAGCTGTCGAGTCTCGGGCTTAACGCTGTAGCGTATTACCGGGGTCTTGATGTGTCCGTCATACCGACCAGCGGAGACGTCGTTGTCGTGGCGACAGACGCTCTAATGACGGGTTTCACTGGCGACTTTGACTCAGTGATCGACTGTAATACATGTGTTACCCAGACAGTCGACTTCAGCTTGGATCCTACCTTCACCATTGAGACGACAACCGTGCCCCAAGACGCGGTGTCGCGCTCGCAGCGGCGAGGCAGGACTGGTAGGGGCAGGGCAGGCATCTACAGGTTCGTGACTCCAGGAGAGCGGCCCTCGGGCATGTTCGATTCCTCGGTCCTGTGTGAGTGCTATGACGCGGGCTGTGCTTGGTACGAGCTCACGCCCGCCGAGACCTCAGTTAGGTTGCGGGCTTACCTGAATACACCAGGGTTGCCCGTCTGCCAGGACCATCTGGAGTTCTGGGAGGGCGTCTTCACAGGCCTCACCCACATAGATGCCCACTTCCTGTCCCAGACCAAGCAAGCAGGAGACAACTTCCCCTACTTGGTAGCATACCAAGCTACGGTGTGCGCCAGGGCTCAGGCTCCACCCCCATCGTGGGACCAAATGTGGAAATGTCTCATACGGCTAAAACCCACGCTGCACGGGCCAACACCCCTGCTGTACAGGTTAGGAGCCGTCCAAAATGAGGTCACTCTCACACACCCCATAACCAAATTCATCATGGCATGTATGTCGGCTGACCTGGAGGTCGCCACG

>HM106695

GCGCCCATCACGGCCTATGCCCARCAGACGCGGGGCCTACTTGGCTGCATCGTCACCAGCCTCACAGGCCGGGACAAGAACCAGGTCGAGGGGGAGGTTCAAGTGGTTTCCACCGCAACNNNNNNNNNNNNNNNNNNNNNNNNNNNNNNNNNNNNNNNNNNNNNNNNNNATGGCGCCGGCACAAAGACCCTCGCCGGCCAAAAGGGCCCAATCACCCAAATGTACACCAATGTAGACCAGGACCTCGTCGGCTGGCAAGCGCCTCCCGGGGCGCGATCCTTGACACCGTGCACCTGTGGCAGCTCGGACCTCTACTTGGTCACGAGGCATGCTGATGTCATTCCGGTGCGCCGGCGGGGCGACAGCAGAGGAAGCCTACTCTCCCCTAGGCCCGTCTCCTACCTGAGGGGCTCCTCGGGTGGTCCACTGCTCTGCCCCTCGGGGCATGCTGTGGGCATCTTCCGGGCTGCTGTGTGCACCCGGGGGGTTGCGAAGGCGGTGGACTTCGTACCCGTTGAGTCTATGGAAACAACTATGCGGTCCCCGGTCTTTACAGACAACTCGTCTCCCCCGGCCGTACCGCAGACATTCCAAGTGGCCCATCTACACGCTCCCACTGGCAGCGGCAAGAGCACTAAGGTGCCGGCTGCGTATGCAGCCCAAGGGTACAAGGTACTTGTCCTGAACCCGTCTGTTGCCGCCACTYTAGGTTTYGGGGCGTATATGTCTAAGGCACATGGTGTYGACCCTAACATCAGAACTGGGGTAAGGACCATCACCACGGGTGCCCCCATTACGTACTCCACCTATGGCAAGTTTCTTGCCGACGGTGGTTGCTCTGGGGGCGCCTATGACATCATAATANNNNNNNNNNNNNNNNNNNNNNNNNNNNNNNNNNNNNNNNNNNNNNNNNNNNNNNNNNNNNNNNNNNNNNNNNNNNNNNNNNNNNNNNNNNNNNNNNNNNNNNNNNNNNNNNNNNNNNNNNNNNNNNNNNNNNNNNNNNNNNNNNNNNNNNNNNNNNNNNNNNNNNNNNNNNNNNNNNNNNNNNNNNNNNNNNNNNNNNNNNNNNNNNNNNNNNNNNNNNNNNNNNNNNNNNNNNNNNNNNNNNNNNNNNNNNNNNNNNNNNNNNNNNNNNNNNNNNNNNNNNNNNTCGGGCTTAACGCTGTAGCGTACTACCGGGGCCTTGATGTGTCCGTCATACCGACCAGCGGAGACGTCGTTGTCGTGGCAACAGACGCTCTAATGACGGGTTACACTGGCGACTTTGACTCAGTGATCGACTGCAATACATGTGTCACCCAGACAGTCGACTTCAGCTTGGACCCTACCTTCACCATTGAGACAACGACCGTGCCCCAAGACGCGGTGTCGCGCTCGCAGCGGCGAGGCAGGACTGGCAGGGGCAGGACAGGCATCTACAGGTTTGTGACTCCAGGAGAACGGCCCTCGGGCATGTTCGATTCTTCGGTCCTGTGTGAGTGCTATGACGCGGGCTGTGCTTGGTACGAGCTCACACCCGCCGAGACCACAGTTAGGTTGCGGGCTTACCTGAATACACCAGGGTTGCCCGTCTGCCAGGACCATCTGGAGTTCTGGGAGGGCGTCTTCACAGGCCTCACCCACATAGATGCCCACTTCCTGTCCCAGACTAAGCAAGCAGGAGACAACTTCCCCTACTTGGTAGCATACCAGGCTACGGTGTGCGCCAGGGCTCAGGCTCCACCCCCATCGTGGGACCAAATGTGGAAATGTCTCATACGGCTAAAGCCCACGCTGCACGGGCCAACGCCCCTGCTGTATAGGCTAGGAGCCGTCCAAAATGAGGTCGTCCTCACACACCCCATCACCAAATTCATCATGGCATGCATGTCGGCTGACCTGGAGGTCGCCACG

>HM106696

GCGCCCATCACGGCCTACGCCCAACAAACGCGGGGCCTACTTGGCTGCATCGTCACCAGCCTCACGGGCCGGGACAAGAACCAGGTGGAGGGGGAGGTTCAANNNNNNNNNNNNNNNNNNNNNNNNNNNNNNNNNNNNNNNNNNNNNNNNNNNNNNNNNNNNNNNTACCACGGCGCCGGCGCAAAGACYCTTGCCGGCCAAAAAGGCCCAATCACCCAGATGTACACCAATGTAGACCAGGACCTCGTCGGCTGGCAAGCGCCTTCCGGGGCGCGATCCTTGACACCGTGCACCTGTGGCAGCTCGGACCTTTACTTGGTCACGAGGCATGCTGATGTCATTCCGGTGCGACGGCGGGGCGAYRGMAGAGGAAGCCTACTCTCCCCCAGGCCCGTCTCCTACTTGAAGGGCTCTTCGGGTGGTCCACTKCTCTGCCCCTCAGGGCATGCTGTGGGCATYTTCCGGGCCGCCGTGTGCACCCGGGGGGTTGCGAAGGCGGTGGACTTTGTGCCCGTYGAGTCTATGGAAACAACTATGCGGTCCCCGGTCTTCACGGACAACTCGTCCCCCCCGGCCGTACCGCAGACATYCCAAGTGGCCCATCTACACGCYCCCACYGGCAGCGGCAARAGCACTAAGGTGCCGGCTGCATATGCAGCCCAAGGGTACAAGGTACTCGTCCTGAACCCKTCTGTTGCCGCCACCCTAGGTTTCGGGGCGTACATGTCCAAGGCAYATGGTGTCGACCCTAACATTAGAACTGGGGTAAGGACCATCACCACGGGTGCTCCCATTACGTACTCCACCTATGGCAAGTTCCTTGCCGACGGTGGTTGCTCTGGGGGCGCCTAYGACATCATAATWTGTGATGAGTGTCACTCAACTGACTCGACTACCATCTTGGGYATCGGCACAGTCCTGGACCAAGCGGARACKGCTGGAGCGCGACTTGTCGTACTCGCCACCGCTACGCCTCCGGGATCGGTCACCGTGCCACATCCCAATATCGAGGAGGTGGCTCTGTCCNNNNNNNNNNNNNNNNNNNNNNNNNNNNNNNNNATCCCCATCGAGACCATCAAGGGGGGGAGGCACCTCATATTTTGCCATTCCAGGAAAAAATGTGATGAGCTCGCCGCAAAGCTGTCGAGTCTCGGGCTTAACGCTGTAGCGTATTACCGGGGTCTTGACGTGTCCGTCATACCGACCAGCGGAGACGTCGTTGTCGTGGCAACAGACGCTCTAATGACGGGCTTCACTGGCGACTTTGACTCAGTGATCGACTGTAATACATGTGTCACCCAGACAGTCGACTTCAGCTTGGACCCTACCTTCACCATCGAGACAACAACCGTGCCTCAAGACGCGGTGTCGCGCTCGCAGCGGCGAGGCAGGACTGGTAGGGGCAGGTCGGGCATCTACAGGTTTGTGACTCCAGGAGAACGGCCCTCGGGCATGTTCGATTCCTCGGTCCTGTGTGAGTGCTATGACGCGGGCTGTGCTTGGTATGAGCTCACGCCCGCCGAGACCACAGTTAGGTTGCGGGCTTACCTGAATACACCAGGGTTGCCCGTCTGCCAGGACCATCTGGAGTTCTGGGAGGGTGTCTTCACAGGCCTCACCCACATAGATGCCCACTTCCTGTCCCAGACTAAGCAGGCAGGAGACAACTTCCCCTACTTGGTAGCATACCAGGCTACGGTGTGCGCCAGGGCTCAGGCTCCACCCCCATCGTGGGACCAAATGTGGAAATGTCTTATACGGCTAAAGCCCACGCTGCACGGGCCAACGCCCCTGCTGTATAGGCTAGGAGCCGTCCAAAATGAGGTCACCCTCACACATCCCATAACCAAATTTATCACAGCATGCATGTCGGCTGACCTGGAGGTTGCCACG

>HM106697

GCGCCCATCACGGCCTATGCCCAACARACGCGGGGCCTACTTGGCTGCATCATCACYAGCCTCACAGGCCGGGACAAGAACCAGGTCGAGGGGGAGGTGCAAGTGGTTTCYACCGCAACACAGTCTTTCCTGGCGACCTGCRTCAACGGCGTGTGTTGGACYGCCTACCACGGCGCCGGCACGAAGACCCTCGCCGGCCCAAAGGGCCCARTCACCCAAATGTACACCAATGTAGACCAGGACCTCGTCGGCTGGCAAGCGCCCCCTGGGGCGCGATCCTTGACACCGTGCACCTGTGGCAGCTCAGACCTTTATTTGGTCACGAGGCATGCTGATGTCATTCCGGTGCGCCGACGGGGCGACAGCAGAGGAAGYCTACTCTCCCCCAGGCCCGTCTCCTACTTGAAGGGCTCTTCGGGTGGYCCACTGCTCTGCCCCTCGGGGCATGCTGTGGGCATCTTCCGGGCTGCTGTGTGCACCCGGGGGGTTGCGAAGGCGGTGGACTTTGTGCCCGTCGAGTCCATGGAAACAACTATGCGGTCCCCGGTCTTCACGGACAAYTCGTCCCCCCCGGCCGTACCGCAGACATTCCAAGTGGCCCATCTACACGCTCCCACTGGCAGCGGCAAGAGCACTAGGGTGCCGGCTGCGTATGCAGCCCAAGGGTACAAGGTACTCGTCCTGAACCCGTCTGTTGCCGCCACCCTAGGTTTCGGGGCGTATATGTCTAAGGCACATGGTGTCGACCCCAACATCAGAACTGGGGTAAGRACCATCACTACGGGTGCCCCCATTACGTACTCCACCTATGGCAAGTTTCTTGCCGACGGTGGTTGCTCCGGGGGCGCTTATGACATCATAATATGTGATGAGTGCCACTCAACTGACTCGACTACCATCTTGGGCATCGGCACGGTCCTGGACCAAGCGGAGACGGCTGGAGCGCGACTTGTCGTACTCGCCACCGCTACGCCTCCGGGATCGGTCACCGTGCCACATCCCAATATCGAGGAGGTGGGTCTGTCCAACACTGGAGAGATCCCCTTCTATGGTAAAGCCATCCCCATCGAGGCCATCAAGGGGGGGAGGCACCTCATATTTTGCCATTCCAAGAAAAAATGTGATGAGCTCGCCGCAAAGCTGTCGGGTCTCGGGCTTAACGCTGTAGCGTATTACCGGGGTCTCGACGTGTCCGTCATACCGACCAGCGGAGACGTCGTTGTCGTGGCAACAGACGCTCTAATGACGGGTTACACTGGCGACTTTGACTCAGTGATCGACTGTAATACATGTGTCACCCAGACAGTCGACTTTAGCTTGGACCCTACCTTCACCATTGAGACGACGACCGTGCCCCAAGACGCGGTGTCGCGCTCGCAGCGGCGAGGCAGGACTGGTAGGGGCAGGGCAGGCATCTACAGGTTTGTGACTCCAGGAGAACGGCCCTCGGGCATGTTCGATTCCTCGGTCCTGTGTGAGTGCTATGACGCGGGCTGTGCTTGGTACGAGCTCACGCCCGCCGAGACCTCAGTTAGGTTRCGGGCTTACCTGAATACACCAGGGTTGCCCGTCTGCCAGGACCATCTGGAGTTCTGGGAGGGCGTCTTCACAGGCCTCACCCACATAGAYGCCCACTTCCTGTCCCAGACTAAGCAGGCAGGAGACAACTTCCCCTACTTGGTAGCATACCAGGCTACGGTGTGCGCCAGGTCTCAGGCTCCWCCCCCATCGTGGGACCAAATGTGGAAATGTCTCATACGGCTAAAGCCCACGCTGCACGGGCCAACACCCCTGCTGTATAGGCTAGGAGCCGTCCAAAATGAGATTACCCTCACACACCCCATAACYAAATTCATCATGGCATGCATGTCGGCTGACCTGGAGGTCGCCACG

>HM106698

NNNNNNNNNNNNNNNNNNNNNNNNNNNNNNNNNNNNNNNNNNNNNNNNNNNNNNNNNNNNNNNNNNNNNNNNNNNNNNNNNNNNNNNNNNNNNNNNNNNNNAGTGGTCTCTACCGCAACACAATCTTTCCTGGCGACCTGCGTCAACGGCGTGTGTTGGACTGTCTACCACGGCGCCGGCACAAAGACCCTTGCCGGCCAAAAAGGCCCAATCACCCAGATGTACACCAATGTAGACCAGGACCTCGTCGGCTGGCAGGCGCCTCCCGGGGCGCGATCCTTGACACCGTGCACCTGTGGCAGCTCGGACCTTTACTTGGTCACAAGGCATGCTGATGTTATTCCGGTGCGCCGGCGGGGCGACAGCAGAGGAAGCCTACTTTCCCCCAGGCCCATCTCCTACTTGAAGGGCTCTTCGGGTGGTCCACTGCTCTGCCCCTCGGGGCATGTTGTAGGCATCTTCCGGGCTGCTGTGTGCACCCGGGGGGTCGCGAAGGCGGTGGACTTTGTGCCCGTTGAGTCTATGGAAACAACTATGCGGTCCCCGGTCTTCACGGACAACTCGTCCCCTCCGGCCGTACCGCARACATTCCAAGTGGCCCATTTACACGCTCCCACTGGCAGCGGCAAGAGCACTAAGGTGCCGGCTGCATATGCAGCCCAAGGGTATAAGGTACTCGTCTTGAACCCRTCTGTYGCCGCCACCCTAGGTTTCGGGGCGTACATGTCTAAGGCRCATGGTGTCGACCCTAACATCAGAACTGGGGTAAGGACCATCACCACGGGTGCCCCCATTACGTACTCCACCTATGGCAAGTTCCTTGCCGACGGTGGTTGCTCTGGGGGCGCCTATGACATCATAATATGTGATGAGTGTCACTCAACTGACTCGACTACCATCTTGGGCATTGGCACAGTCCTGGACCAAGCGGAGACGGCTGGAGCGCGACTCGTCGTACTCGCCACCGCTACGCCTCCGGGATCGGTCACCGTGCCACAYCCCAATATCGAGGAGGTGGCTCTGTCCAACACTGGAGAGATCCCCTTCTATGGTAAAGCCATCCCCATCGAGNNNNNNNNNNNNNNNNNNNNNNNNNNNNNNNNNNNNNNNNNNNNNNNNNNNNNNNNNNNNNNNNNNNNNNNNNNNNNNNNNNNNNNNNNNNNNNNNNNNNNNNNNNNNNNNNNNNNNNNNNNNNNNNNNNNNNNNNNNNNNNNNNNNNNNNNNNNNNNNNNNNNNNNNNNNNNNNNNNNNNNNNNNNNNNNNNNNNNNNNNNNNNNNNNNNNNNNNNNNNNNNNNNNNNNNNNNNNNNNNNNNNNNNNNNNNNNNNNNNNNNNNNNNNNNNNNNNNNNNNNNNNNNNNNNNNNNNNNNNNNNNNNNNNNNNNNNNNNNNNNNNNNNNNNNNNNNNNNNNNNNNNNNNNNNNNNNNNNNNNNNNNNNNNNNNNNNNNNNNNNNNNNNNNNNNNNNNNNNNNNNNNNNNNNNNNNNNNNNNNNNNNNNNNNNNNNNNNNNNNNNNNNNNNNNNNNNNNNNNNNNNNNNNNNNNNNNNNNNNNNNNNNNNNNNNNNNNNNNNNNNNNNNNNNNNNNNNNNNNNNNNNNNNNNNNNNNNNNNNNNNNNNNNNNNNNNNNNNNNNNNNNNNNNNNNNNNNNNNNNNNNNNNNNNNNNNNNNNNNNNNNNNNNNNNNNNNNNNNNNNNNNNNNNNNNNNNNNNNNNNNNNNNNNNNNNNNNNNNNNNNNNNNNNNNNNNNNNNNNNNNNNNNNNNNNNNNNNNNNNNNNNNNNNNNNNNNNNNNNNNNNNNNNNNNNNNNNNNNNNNNNNNNNNNNNNNNNNNNNNNNNNNNNNNNNNNNNNNNNNNNNNNNNNNNNNNNNNNNNNNNNNNNNNNNNNNNNNNNNNNN

>HM106699

GCGCCCATCACGGCCTAYGCCCAACAGACGCGGGGCCTMCTTGGCTGTATCATCACCAGCCTCACAGGCCGGGATAAGAACCAGGTCGAGGGGGAGGTTCAAGTGGTTTCCACCGCAACACAATCYTTCCTGGCGACCTGCGTCAACGGCGTGTGTTGGACTGTCTACCACGGCGCCGGCACAAAGACCCTCGCCGGCCAAAAGGGCCCAATCACCCAAATGTACACCAATGTAGACCAAGACCTCGTCGGCTGGCAAGCGCCTCCCGGGGCGCGATCCCTGACACCGTGCACCTGTGGCAGCTCAGACCTTTACTTGGTCACGAGGCATGCTGATGTCATTCCGGTGCGCCGGCGGGGCGACAGCAGAGGAAGCCTACTTTCCCCCAGGCCCGTCTCCTACCTGAAGGGCTCTTCGGGTGGTCCACTGCTCTGCCCCTCGGGGCACGCTGTGGGCATCTTCCGGGCTGCTGTGTGCACACGGGGGGTTGCGAAGGCGGTGGACTTTGTGCCCGTTGAGTCTATGGAAACAACTATGCGGTCCCCGGTCTTCACGGACAACTCGTCCCCTCCGGCCGTACCGCAGACATTCCAAGTGGCCCATCTACACGCTCCTACTGGCAGCGGCAAGAGCACCAAGGTGCCGGCTGCGTATGCAGCCCAGGGGTACAAGGTGCTCGTCCTCAACCCGTCCGTTGCCGCCACCCTAGGTTTCGGGGCGTATATGTCTAAGGCACATGGTGTCGACCCTAACATCAGAACTGGGGTAAGGACCATCACCACGGGTGCCCCCATTACGTACTCCACCTATGGCAAGTTCCTTGCCGACGGTGGTTGCTCTGGGGGCGCCTACGACATCATAATATGTGATGAGTGCCACTCAACTGACTCGACTACTATCTTGGGCATCGGTACAGTCCTGGACCAAGCGGAGACGGCTGGAGCGCGACTCGTCGTACTCGCCACCGCTACGCCTCCGGGATCGGTCACCGTGCCACATCCCAATATTGAGGAGGTGGCTCTGTCCAACATTGGAGAGATCCCCTTCTATGGTAAAGCCATCCCCATCGAGACCATCAAAGGGGGGAGGCATCTCATATTYTGCCATTCCAARAARAAATGTGATGAGCTCGCCGCAAAGCTGTCGGGCCTCGGGCTTAAYGCTGTAGCGTATTACCGGGGYCTTGATGTGTCCGTCATACCGACYAGCGGAGACGTCGTTGTCGTGGCAACAGACGCTCTAATGACGGGTTTCACTGGCGACTTTGACTCAGTGATCGACTGTAATGTATGTGTCACCCAGACAGTCGACTTCAGCTTGGACCCTACCTTCACCATTGAGACRACGACCGTGCCCCARGACGCGGTGTCGCGCTCGCAGCGGCGAGGCAGGACCGGTAGGGGCAGGACAGGCATCTACAGGTTTGTGACTCCAGGAGAACGGCCCTCGGGCATGTTCGATTCCTCGGTCCTGTGTGAGTGCTATGACGCGGGCTGTGCTTGGTACGAGCTCACGCCCGCCGAGACCTCAGTTAGGTTGCGGGCTTACATGAACACACCAGGGTTGCCCGTCTGCCAGGACCATCTGGAGTTCTGGGAGGGCGTCTTCACAGGCCTCACCCACATAAATGCCCACTTCCTGTCCCAGACTAAGCAAGCAGGAGACAACTACCCCTACTTGGTAGCATACCAGGCTACGGTGTGCGCCAGAGCTCAGGCTCCCCCCCCATCGTGGGACCAAATGTGGAAATGTCTCATACGGCTAAAGCCCACGCTGCACGGGCCAACGCCCCTGCTGTATAGGCTAGGAGCCGTCCAGAATGAGGTCACCCTCACACACCCCATAACYAAATTCATCATGGCATGCATGTCGGCTGACCTGGAGGTCGCCACG

>HM106700

GCGCCCATCACGGCCTATGCCCAACAGACGCGGGGCCTACTTGGCTGCATTATCACCAGCCTCACAGGCCGGGACAAGAACCAGGTCGAGGGGGAGGTTCAAGTGGTTTCTACCGCGACACAATCTTTCCTGGCGACCTGCGTCAACGGCGTGTGTTGGACTGTCTATCACGGYGCCGGCACGAAGACCCTCGCCGGCCCAAAGGGCCCAATCACCCAAATGTACACCAATGTRGACCAGGACCTCGTCGGCTGGCAAGCGCCTCCCGGGGCGCGATCCTTGACACCSTGCACCTGTGGCAGCTCGGACCWTTACTTGGTCACGAGGCATGCTGATGTCATTCCGGTGCGCCGGCGGGGCGACAGCAGAGGAAGYCTACTTTCCCCCAGGCCCGTCTCCTACTTGAAAGGYTCTTCGGGCGGTCCGCTGCTCTGCCCCTCGGGGCATGCTGTGGGCATCTTCCGGGCTGCTGTGTGCACCCGGGGGGTTGCGAAGGCGGTGGACTTTGTGCCCGTCGAGTCTATGGAAACAACCATGCGGTCCCCRGTCTTCACGGACAACTCGTCCCCYCCGGCCGTACCGCAGACATTCCAAGTGGCCCATCTACACGCTCCCACTGGCAGCGGCAAGAGCACTAAGGTGCCGGCTGCGTATGCAGCCCAGGGGTACAAGGTACTCGTYCTGAACCCRTCTGTTGCCGCCACCCTAGGTTTCGGGGCGTATATGTCTAAGGCACATGGTATYGACCCTAACATCAGAACTGGGATAAGAACCATCACCACGGGTGCCCCCATTACGTACTCCACTTATGGCAAGTTCCTTGCCGACGGTGGTTGTTCTGGGGGCGCCTATGACATCATAATGTGCGATGAGTGCCACTCAGTTGACTCGACTACCATCTTGGGCATCGGCACAGTCCTGGACCAAGCGGAAACGGCTGGAGCGCGACTCGTCGTACTCGCCACCGCTACGCCTCCGGGATCGGTCACCGTGCCACATCCCAATATCGAGGAGGTGGCTCTGTCCAACACTGGAGAGATCCCCTTCTATGGTAAAGCCATCCCCATCGAGNNNNNNNNNNNNNNNNNNNNNNNNNNNNNNNNNNNNNNNNNNNNNNNNNNNNNNNNNNNNNNNNNNNNNNNNNNNNNNNNNNNNNNNNNNNNNNNNNNNNNNNNNNNNNNNNNNNNNNNNNNNNNNNNNNNNNNNNNNNNNNNNNNNNNNNNNNNNNNNNNNNNNNNNNNNNNNNNNNNNNNNNNNNNNNNNNNNNNNNNNNNNNNNNNNNNNNNNNNNNNNNNNNNNNNNNNNNNNNNNNNNNNNNNNNNNNNNNNNNNNNNNNNNNNNNNNNNNNNNNNNNNNNNNNNNNNNNNNNNNNNNNNNNNNNNNNNNNNNNNNNNNNNNNNNNNNNNNNNNNNNNNNNNNNNNNNNNNNNNNNNNNNNNNNNNNNNNNNNNNNNNNNNNNNNNNNNNNNNNNNNNNNNNNNNNNNNNNNNNNNNNNNNNNNNNNNNNNNNNNNNNNNNNNNNNNNNNNNNNNNNNNNNNNNNNNNNNNNNNNNNNNNNNNNNNNNNNNNNNNNNNNNNNNNNNNNNNNNNNNNNNNNNNNNNNNNNNNNNNNNNNNNNNNNNNNNNNNNNNNNNNNNNNNNNNNNNNNNNNNNNNNNNNNNNNNNNNNNNNNNNNNNNNNNNNNNNNNNNNNNNNNNNNNNNNNNNNNNNNNNNNNNNNNNNNNNNNNNNNNNNNNNNNNNNNNNNNNNNNNNNNNNNNNNNNNNNNNNNNNNNNNNNNNNNNNNNNNNNNNNNNNNNNNNNNNNNNNNNNNNNNNNNNNNNNNNNNNNNNNNNNNNNNNNNNNNNNNNNNNNNNNNNNNNNNNNNNNNNNNNNNNNNNNNNNNNNNNNNNNNN

>HM106701

GCGCCCATCACGGCCTAYGCCCAACAGACGCGGGGCCTACTTGGCTGCATCGTCACCAGCCTCACAGGCCGGGACAAGAACCAGGTCGAGGGGGAGGTTCAAGTGGTTTCCACCGCAACACAATCTTTCCTGGCGACCTGCGTCAACGGCGTGTGTTGGACTGTCTACCATGGCGCCGGCACAAAGACCCTCGCCGGCCAAAAGGGCCCAATCACCCAAATGTACACCAATGTDGACCAGGACCTCGTCGGCTGGCAAGCGCCTCCCGGGGCGCGATCCTTGACACCGTGCACCTGTGGCAGCTCGGACCTTTACTTGGTCACGAGGCATGCTGATGTCATTCCGGTGCGCCGGCGGGGCGACAGCAGAGGAAGCCTACTCTCCCCCAGGCCCGTCTCCTACTTGAAGGGCTCTTCGGGTGGTCCACTGCTCTGCCCCKTGGGGCATGTTGCGGGCATCTTYCGGGCTGCTGTGTGCACCCGGGGGGTTGCGAAGGCGGTGGACTTTGTGCCYGTCGAGTCTATGGAAACAACYATGCGGTCCCCGGTCTTCACGGACAAYTCGTCCCCCCCGGCCGTACCGCAGACATTCCAAGTGGCCCATCTACACGCTCCCACTGGCAGCGGCAAGAGCACTAAGGTGCCGGCTGCGTATGCAGCCCAAGGGTAYAAGGTACTYGTCCTWAACCCGTCTGTTGCCGCCACCCTAGGTTTCGGGGCGTATATGTCTAAGGCACATGGTGTCGACCCTAACATCAGAACTGGGGTAAGGACCATCACCACGGGTGCCCCCATTACGTACTCCACCTATGGCAAGTTYCTTGCCGACGGTGGTTGCTCTGGGGGCGCCTATGACATCATAATGTGTGATGAGTGCCACTCAACTGACTCGACTACCATCTTGGGYATCGGCACAGTCCTGGACCAAGCGGAGACGGCTGGAGCGCGACTCGTCGTACTCGCCACCGCTACGCCTCCGGGATCGGTCACCGTGCCACATCCCAATATCGAGGAGGTGGCTCTGTCCAACATTGGAGAGATTCCCTTCTATGGTAAAGCCATCCCCATCGAGACCATCAAGGGGGGGAGGCACCTCATATTTTGCCATTCCAGAAAGAAATGTGATGAGCTCGCCGCAAAGCTGTCGGGTCTCGGGCTTAACGCTGTAGCGTATTACCGGGGYCTTGATGTGTCCGTCATACCGACCAGCGGAGACGTCGTTGTCGTGGCAACAGACGCTCTAATGACGGGTTTYACTGGCGACTTTGACTCAGTGATCGACTGTAATACGTGTGTCACCCAGACAGTCGACTTCAGCTTGGACCCCACCTTCACCATTGARACGACGACCGTGCCTCAGGACGCAGTGTCGCGCTCGCAGCGGCGAGGCAGRACTGGTAGGGGCAGGGCAGGCATCTACAGGTTTGTGACTCCAGGAGAACGGCCCTCGGGCATGTTCGACTCTTCGGTCCTGTGTGAGTGCTATGACGCGGGCTGTGCTTGGTACGAGCTCACGCCCGCCGAGACCTCAGTTAGRCTGCGGGCTTACCTGAATACACCAGGGTTGCCCGTCTGCCAGGACCATCTGGAGTTCTGGGAGGGCGTCTTCACAGGCCTCACCCACATAGATGCCCACTTCCTGTCCCAGACTAAGCAGGCAGGAGACAACTTCCCCTACTTGGTAGCATACCAGGCTACGGTGTGCGCCAGGGCTCAGGCTCYACCYCCATCGTGGGACCAAATGTGGAARTGTCTCATACGGCTRAAGCCYACGCTGCACGGGCCAACACCCCTGCTGTATAGGCTAGGAGCCGTCCAAAATGAGGTCACCCTCACACACCCCATAACCAAATTCATCATGGCATGCATGTCGGCTGACCTGGAGGTCGCTACG

>HM106702

GCGCCCATCACGGCTTATGCCCAACAGACGCGGGGCCTACTTGGCTGCATCATCACCAGCCTCACAGGCCGGGACAAGAACCAGGTCGAGGGGGAGGTTCAAGTGGTTTCCACCGCAACACAATCTTTCCTGGCGACCTGCGTCAACGGCGTGTGTTGGACTGTCTACCACGGCGCCGGCACGAAGACCCTCGCCGGCCAAAAGGGGCCAATTACTCAAATGTACACCAATGTAGACCAGGACCTCGTCGGCTGGCAAGCGCCTCCCGGGGCGCGATCCTTGACACCGTGCACCTGTGGCAGCTCGGACCTTTACTTGGTCACTAGGCATGCTGATGTCATTCCGGTGCGCCGGCGGGGCGACAGCAGGGGAAGTCTACTCTCCCCCAGGCCCGTCTCCTATTTGAAGGGCTCTTCGGGTGGTCCACTGCTCTGCCCCTCGGGGCATGCTGTGGGCATCTTCCGGGCTGCTGTGTGCACCCGGGGGGTTGCGAAGGCGGTGGACTTTGTGCCCGTTGAGTCTATGGAAACAACTATGCGGTCCCCGGTCTTCACGGACAACTCGTCCCCTCCGGCCGTACCGCAGACATTCCAAGTGGCCCATCTACACGCTCCCACTGGCAGCGGCAAGAGCACTAAGGTACCGGCTGCGTAYGCAGCCCAAGGGTACAAGGTACTYGTTCTGAACCCGTCTGTTGCTGCCACCTTRGGTTTCGGGGCGTATATGTCTAAAGCACATGGTGTCGACCCTAACATCAGAACTGGGGTGAGGACCATCACCACGGGTGCCCCCATCACGTATTCCACCTATGGYAAGTTCCTTGCCGACGGTGGTTGCTCTGGGGGCGCCTATGACATCATAATATGTGATGAGTGCCACTCAACTGACTCGACTACCATCTTGGGCATCGGCACAGTCCTGGACCAGGCGGAGACGGCTGGAGCGCGACTCGTCGTGCTCGCCACCGCTACACCTCCGGGATCGATCACCGTACCACACCCCAATATCGAGGAGGTGGCTCTGTCCAACGTCGGAGAGATCCCCTTTTATGGTAAAGCCATCCCCATCGAGACCATCAAGGGGGGGAGGCACCTCATATTTTGCCATTCCAARAAAAAATGTGATGAGCTCGCCGCAAAGCTGTCGRGYCTCGGGCTTAACGCTGTAGCGTATTACCGGGGTCTTGATGTGTCCGTCATACCGACCAGCGGAGACGTCGTTGTCGTGGCAACAGACGCTCTAATGACGGGTTTCACTGGCGACTTTGACTCAGTRATCGACTGTAATACRTGTGTCACCCARACGGTCGACTTCAGCTTAGACCCTACCTTCACCATTGAGACGACGACCGTGCCCCAAGACGCGGTGTCACGCTCGCAGCGGCGAGGCAGGACTGGTAGGGGCAGGGCAGGCATCTACAGGTTTGTGACTCCAGGAGAACGGCCCTCGGGCATGTTCGATTCCTCGGTCCTGTGTGAGTGTTATGACGSGGGCTGTGCTTGGTACGAGCTCACGCCCGCCGAGACCTCAGTYAGGTTGCGGGCTTACCTGAAYACACCAGGGTTGCCCGTCTGCCAGGACCATCTGGAGTTCTGGGAGGGCGTCTTCACAGGCCTYACCCACATAGATGCCCACTTCCTGTCCCAGACTAAGCAGGCAGGRGACAACCTCCCCTACTTGGTAGCATACCARGCYACGGTGTGTGCCAGGGCTCAGGCTCCACCCCCGTCGTGGGACCAAATGTGGAAATGTCTCATACGGCTAAAGCCCACGCTGCACGGGCCAACACCCCTGCTGTATAGGCTAGGAGCCGTCCAAAATGAGATCACCCTCACACACCCCATAACCAAATTCATCATGGCATGCATGTCGGCTGACCTGGAGGTCGCCACG

>HM106703

GCGCCCATCACGGCCTATGCCCAACAAACACGGGGCCTACTTGGCTGCATCGTCACCAGCCTCACAGGCCGGGACAAGAACCAGGTCGAGGGGGAGGTTCAAGTGGTTTCTACCGCAACACAATCTTTCCTGGCGACCTGCGTCAACGGCGTGTGTTGGACCGTCTACCACGGCGCCGGCACAAAGACCCTTGCCGGCCCAAAAGGCCCAATCACCCAAATGTACACCAATGTGGACCAGGACCTCGTYGGCTGGCAAGCGCCTCCCGGGGCGCGATCCTTGACACCGTGCACCTGTGGCAGCTCGGACCTTTACYTGGTCACGAGGCATGCTGATGTCATTCCGGTGCGCCGGCGGGGCGACAGCAGAGGAAGCCTACTCTCCCCCAGGCCCATCTCCTACTTGAAGGGCTCTTCAGGTGGTCCACTGCTCTGCCCCTCGGGGCATGCTGTGGGCATCTTCCGGGCTGCTGTGTGCACCCGGGGGGTCGCGAAGGCGGTGGACTTTGTGCCCGTTGAGTCTATGRAAACAACTATGCGGTCCCCGGTCTTCACGGACAACTCGTCCCCCCCGGCCGTACCGCAGACATTCCAAGTGGCCCATCTACACGCTCCCACTGGCAGCGGCAAGAGCACTAAGGTGCCGGCTGCATATGCAGCCCAAGGGTACAAGGTACTCGTCCTGAACCCGTCTGTTGCCGCCACCCTAAGTTTCGGGGCGTACATGTCTAAGGCATATGGTATCGACCCTAACATCAGAACTGGGGTAAGGACCATCACCACGGGTGCCCCCATTACGTACTCCACCTATGGCAAGTTCCTTGCCGACGGCGGTTGCTCTGGGGGCGCCTATGACATCATAATATGTGATGAGTGCCACTCAACTGACTCGACTACCATCTTGGGCATCGGCACAGTCCTRGACCAAGCGGAGACGGCTGGRGCGCGACTCGTCGTACTCGCCACCGCTACGCCTCCGGGATCGGTCACCGTGCCACATCCCAATATCGAGGAGGTGGCTCTGTCCAACNNNNNNNNNNNNNNNNNNNNNNNNNNNNNNNNNNNNNNNNNNNNNNNNNNNNNNNNNNNNNNNNNNNNNNNNNNNNNNNNNNNNNNNNNNNNNNNNNNNNNNNNNNNNNNNNNNNNNNNNNNNNNNNNNNNNNNNNNNNNNNNNNNNNNNNNNNNNNNNNNNNNNNNNNNNNNNNNNNNNNNNNNNNNNNNNNNNNNNNNNNNNNNNNNNNNNNNNNNNNNNNNNNNNNNNNNNNNNNNNNNNNNNNNNNNNNNNNNNNNNNNNNNNNNNNNNNNNNNNNNNNNNNNNNNNNNNNNNNNNNNNNNNNNNNNNNNNNNNNNNNNNNNNNNNNNNNNNNNNNNNNNNNNNNNNNNNNNNNNNNNNNNNNNNNNNNNNNNNNNNNNNNNNNNNNNNNNNNNNNNNNNNNNNNNNNNNNNNNNNNNNNNNNNNNNNNNNNNNNNNNNNNNNNNNNNNNNNNNNNNNNNNNNNNNNNNNNNNNNNNNNNNNNNNNNNNNNNNNNNNNNNNNNNNNNNNNNNNNNNNNNNNNNNNNNNNNNNNNNNNNNNNNNNNNNNNNNNNNNNNNNNNNNNNNNNNNNNNNNNNNNNNNNNNNNNNNNNNNNNNNNNNNNNNNNNNNNNNNNNNNNNNNNNNNNNNNNNNNNNNNNNNNNNNNNNNNNNNNNNNNNNNNNNNNNNNNNNNNNNNNNNNNNNNNNNNNNNNNNNNNNNNNNNNNNNNNNNNNNNNNNNNNNNNNNNNNNNNNNNNNNNNNNNNNNNNNNNNNNNNNNNNNNNNNNNNNNNNNNNNNNNNNNNNNNNNNNNNNNNNNNNNNNNNNNNNNNNNNNNNNNNNNNNNNNNNNNNNNNNNNNNNNNNNNNNNNNNNNNNNNNN

>HM106704

GCGCCCATCACGGCCTATGCCCAACAGACGCGGGGGCTATTTGGCTGCATCATCACCAGCCTCACAGGCCGGGACAAGAACCAGGTCGAGGGGGAGGTTCAAGTGGTTTCCACCGCNNNNNNNNNNNNNNNNNNNNNNNNNNNNNNNNNNNNNNNNNNNNNNNNNNNNNNNNNNNNNNNNNNNNNNNNNNNNNNNNNNNNNNNNNNNNCAATCACCCAAATGTACACCAATGTAGACCARGAYCTCGTCGGCTGGCAAGCGCCTCCCGGGGCGCGATCTTTGACACCGTGCACCTGTGGCAGCTCGGACCTTTACTTGGTCACGAGGCATGCTGATGTCATTCCGGTGCGCCGGCGGGGCGACAGCAGAGGAAGYCTACTCTCCCCCAGGCCCGTCTCCTACTTGAAGGGCTCTTCGGGTGGTCCACTGCTCTGCCCCTCGGGGCACGCTGTGGGCATCTTCCGGGCTGCTGTGTGCACCCGGGGGGTTGCGAAGGCGGTGGACTTTGTGCCCGTTGAGTCTATGGAAACAACTATGCGGTCCCCGGTCTTCACGGACAACTCGTCCCCCCCGGCCGTACCGCAGACATTCCAAGTGGCCCATCTACACGCTCCCACTGGCAGCGGCAAGAGCACTAAGGTGCCGGCTGCGTATGCAGCCCAAGGGTACAAGGTACTCGTCCTGAACCCGTCCGTTGCCGCCACCCTAGGTTTCGGGGCGTATATGTCTAAGGCATATGGTGTCGACCCTAACATCAGAACTGGGGTAAGGACCATCACCACGGGTGCCCCCATTACGTACTCCACCTATGGCAAGTTTCTTGCCGACGGTGGTTGCTCTGGGGGCGCCTATGACATCATAATATGTGATGAGTGCCACTCAACTGACTCGACTACCATCTTGGGCATCGGCACAGTCCTGGACCAAGCGGAGACGGCTGGAGCGCGACTCGTCGTACTCGCCACCGCTACGCCTCCGGGATCGGTCACCGTGCCACATCCCAATATCGAGGAGGTGGCTCTGTCCAACATTGGAGAGATCCCCTTCTATGGTAAAGCCATCCCCATCGAGACCATCAAGGGGGGGAGGCACCTCATATTTTGCCATTCCAGGAAAAARTGTGATGAGCTCGCCGCAAAGCTGTCGGGTCTCGGACTTAACGCTGTAGCGTATTACCGGGGCCTTGACGTGTCCGTCATACCGACTAGCGGAGACGTCGTTGTCGTGGCAACAGACGCTCTAATGACGGGTTTCACTGGCGACTTTGACTCAGTGATCGACTGTAATACATGTGTCACCCAGACAGTCAACTTCAGCCTGGACCCTACCTTCACCATTGAGACGACGACCGTGCCCCAAGACGCAGTGTCGCGCTCGCAGCGGCGAGGCAGGACTGGTAGGGGCAGGACAGGCATCTACAGGTTTGTGACTCCAGGAGAACGGCCCTCGGGCATGTTCGATTCCTCGGTCCTGTGTGAGTGCTATGACGCGGGCTGTGCTTGGTACGAGCTCACGCCCGCCGAGACCTCAGTTAGGTTGCGGGCTTACCTGAATACACCAGGGTTGCCCGTCTGCCAGGACCATCTGGAGTTCTGGGAGGGCGTCTTCACAGGCCTCACCCACATAGATGCCCACTTCCTGTCCCAGACTAAGCAGGCGGGAGACAACTTCCCCTACTTGGTAGCATACCAGGCTACGGTGTGCGCTAGGGCTCAGGCTCCACCCCCATCGTGGGACCAAATGTGGAAATGTCTCATACGGCTAAAGCCCACGCTGCACGGGCCAACACCCCTGCTGTATAGGCTAGGAGCCGTTCAAAATGAAGTCACTCTCACACACCCCGTGACCAAATTCATCACGGCATGCATGTCGGCTGACCTGGAGGTCGCCACG

>HM106705

GCGCCCATCACGGCCTACGCCCAACAAACGCGGGGCCTACTTGGCTGCATCRTCACYAGCCTCACAGGCCGGGACAAGAACCAGGTCGARGGRGAGGTTCARGTGGTTTCYACCGCAACACAATCTTTCCTGGCGACCTGCGTCAACGGCGTGTGTTGGACTGTCTACCACGGCGCCGGCACAAAGACCCTTGCCGGCCCAAAAGGCCCAATCACCCAAATGTACACCAATGTAGACCAGGACCTCGTCGGCTGGCAAGCGCCTCCCGGGGCGCGATCCTTGACACCGTGCACCTGTGGCAGCTCGGACCTTTACTTGGTCACGAGGCATGCTGATGTCATTCCGGTGCGCCGGCGGGGCGACAGCAGAGGGAGCCTACTCTCCCCCAGGCCCATCTCTTACCTGAAGGGCTCTTCGGGTGGTCCGCTGCTCTGCCCCTCAGGGCATGCTGTGGGCATCTTCCGGGCTGCTGTGTGCACCCGGGGGGTTGCGAAAGCGGTAGACTTCGTGCCCGTTGAGTCTATGGAAACAACTATGCGGTCCCCGGTCTTCACGGACAACTCGTCTCCCCCGGCCGTACCGCAGACATTCCAAGTGGCCCATCTACACGCCCCCACTGGCAGCGGCAAGAGCACTAAGGTGCCGGCTGCATATGCAGCCCAAGGGTACAAGGTACTCGTCCTGAACCCGTCTGTTGCCGCCACCCTAGGTTTCGGGGCGTACATGTCTAAGGCACATGGTGTCGACCCTAACATCAGAACTGGGGTAAGGACCATCACCACGGGTGCCCCCATTACGTACTCCACCTATGGCAAGTTCCTTGCCGACGGTGGTTGCTCCGGGGGCGCCTATGACATCATAATATGTGATGAGTGTCACTCAACTGACTCGACTACCATCTTGGGCGTCGGCACAGTCCTGGATCAAGCGGAGACGGCTGGAGCGCGACTCGTCGTACTCGCCACCGCTACGCCTCCGGGATCGGTCACCGTGCCACATCCCAATATCGAGGAGGTGGCTCTGTCCCACACTGGAGAGATCCCCTTCTATGGTAAAGCCATCCCCATCGAGACCATCAAGGGGGGGAGGCACCTCATATTTTGTCATTCCAAGAAAAAATGTGATGAGCTCGCTGCAAAGCTGTCGGGTCTCGGGCTTAACGCTGTAGCGTATTACCGGGGTCTTGACGTGTCCGTCATACCGACCAGCGGAGACGTCGTTGTCGTGGCAACAGACGCTCTAATGACGGGCTTCACYGGCGACTTTGACTCAGTGATCGACTGTAATACATGTGTYACCCAGACAGTCGACTTCAGCTTGGAYCCTACCTTCACCATTGASACAACGACCGTGCCCCAAGACGCGGTGTCGCGCTCGCAGCGGCGAGGYAGGACYGGTAGGGGCAGGYCAGGCATCTACAGGTTTGTGACTCCRGGRGAACGGCCCTCGGGCATGTTCGATTCCTCGGTCCTGTGTGAGTGCTATGACGCGGGMTGTGCTTGGTAYGAGCTCACGCCCGCYGAGACCACAGTTAGGTTGCGGGCTTACCTGAAYACACCAGGRTTGCCCGTCTGCCAGGACCATCTGGAGTTCTGGGAGGGCGTCTTCACAGGCCTCACCCAYATAGATGCCCACTTCCTGTCCCAGACCAAGCAGGCAGGRGACAACTTCCCCTACTTGGTAGCATACCAGGCTACGGTGTGCGCCAGGGCTCAGGCTCCACCCCCATCGTGGGACCAAATGTGGAAATGTCTCATACGGCTRAAGCCCACGCTGCACGGGCCAACGCCCCTGCTGTATAGGCTAGGAGCCGTCCAAAATGATGTCATCCTYACACATCCCATAACYAAATACATCATGACATGCATGTCGGCTGACCTGGAGGTTGTCACK

>HM106706

GCGCCCATCACGGCYTAYGCCCAACAGACGCGGGGCCTAYTTGGCTGCATYGTCACCAGCCTCACAGGCCGGGACAAGAACCAGGTCGAGGGGGAGGTTCAAGTGGTTTCTACCGCGACACAATCYTTCCTGGCGACCTGCGTCAACGGCGTGTGTTGGACTGTCTACCACGGCGCCGGCGCGAGGACCCTCGCCGGCCCAAAGGGCCCAATCACCCAAATGTACACCAATGTAGACCAGGACCTCGTCGGCTGGCAAGCGCCTCCCGGGGCGCGATCCTTGACACCGTGCACCTGTGGCAGCTCGGACCWTTACTTGGTCACGAGGCATGCTGATGTCATTCCGGTGCGCCGGCGAGGCGACAGCAGAGGAAGCCTACTCTCCCCCAGGCCCGTCTCCTACTTGAAGGGCTCTTCGGGTGGTCCACTGCTCTGCCCCTCGGGCCATGCTGTGGGCATCTTTCGGGCTGCTGTGTGCACCCGGGGGGTTGCGAAGGCGGTGGACTTTGTRCCCGTTGAGTCTATGGAAACAACTATGCGGTCCCCGGTCTTCACGGACAACTCGTCCCCCCCGGCCGTACCGCAGACATTCCAAGTGGCCCATCTACACGCTCCCACTGGAAGCGGCAAGAGCACTAAGGTGCCGGCTGCGTATGCAGCCCAAGGGTATAAGGTACTCGTCCTGAACCCGTCTGTTGCCGCCACCCTAGGTTTCGGGGCGTATATGTCTAAGGCACATGGTGTCGACCCTAACATCAGAACTGGGGTAAGGACCATCACCACGGGTGCCCCCATTACGTACTCCACCTACGGCAAGTTCCTTGCCGATGGTGGTTGCTCTGGGGGCGCCTACGACATCATAATATGTGATGAGTGCCACTCAACTGACTCGACTACCATCTTGGGCATCGGCACAGTCCTGGACCAAGCGGAGACGGCTGGAGCGCGACTCGTCGTACTCGCCACCACTACGCCTCCGGGATCGGTCACCGTGCCACATCCCAATATCGAGGAGGTGGCTCTGTCCAACATTGGAGAGATCCCCTTCTATGGTAAAGCCATCCCCATCGAGACCATCAAGGGGGGGAGGCACCTCATATTTTGCCATTCCAGGAAAAAGTGTGATGAGCTCGCCGCAAAGCTGTCGGGTCTCGGGCTTAACGCTGTAGCGTATTACCGGGGTCTTGATGTGTCCGTCATACCGACCAGCGGAGACGTCGTTGTCGTGGCAACAGACGCTCTAATGACGGGTTTCACTGGCGACTTTGACTCGGTGATCGACTGTAATACATGTGTCACCCAGACAGTCGACTTCAGCTTGGACCCTACCTTTACCATCGAGACGACGACCGTGCCCCAAGACGCGGTGTCGCGCTCGCAGCGGCGAGGCAGGACTGGTAGGGGCAGGACAGGCATCTACAGGTTTGTGACTCCAGGAGAACGGCCCTCGGGCATGTTCGATTCCTCGGTCCTGTGTGAGTGCTATGACGCGGGCTGTGCTTGGTACGAGCTCACGCCCGCCGAGACCTCAGTTAGGTTGCGGGCTTACCTGAATACACCAGGGTTGCCCGTCTGCCAGGACCATCTGGAGTTCTGGGAGGGCGTCTTTACAGGCCTCACCCACATAGATGCCCACTTCCTGTCCCAGACTAAGCAGGCAGGAGACAACTTCCCCTACTTGGTAGCATACCAGGCTACGGTGTGCGCCAGGGCTCAAGCTCCACCCCCATCGTGGGACCAAATGTGGAAATGTCTCATACGGCTAAAGCCCACGCTGCACGGGCCAACACCCCTGCTGTATAGGCTAGGAGCCGTCCAAAATGAGGTCACCCTCACACACCCCATAACTAAATTCATCATGGCATGCATGTCGGCTGACCTGGAGGTCGCCACG

>HM106707

GCGCCCATCACGGCCTATGCCCARCAGACGCGGGGCCTACTTGGCTGCATCRYCACCAGCCTCACAGGCCGGGACAAGAACCAGGTCGAGGGRGAGGTTCAAGTGGTTTCCACCGCAACACAATCTTTCCTGGCGACCTGTGTCAACGGCGTGTGCTGGACTGTCTACCACGGCGCCGGCWCAAAGACCCTCGCCGGCCMAAAAGGCCCAATCACCCAAATGTACACCAATGTAGACCAGGACCTCGTCGGCTGGCAGGCGCCTCCCGGGGCGCGATCCTTGACACCGTGCACCTGTGRCASCTCGGACCWCTACTTGGTCACGAGGCATRCTGATGTCATTCCGGTGCGCCGGCGGGGCGACAGCAGAGGAAGYCTACTCTCCCCCAGRCCCRTCTCCTACCTGAAGGGCTCTTCGGGTGGTCCACTGCTCTGCCCCTCGGGGCACGCTGTGGGCATCTTCCGGGCTGCCGTRTGCACCCGGGGGGTCGCGAAGGCGGTGGACTTTGTGCCCGTTGAGTCTATGGAAACAACTATGCGGTCCCCGGTCTTCACGGACAACTCGTCCCCCCCGGCCGTRCCGCAGACATTCCAAGTGGCCCATCTACACGCTCCYACTGGCAGCGGCAAGAGCACTAAGGTGCCGGCTGCGTAYGCAGCCCAAGGGTACAAGGTACTCGTCCTGAACCCRTCTGTTGCCGCCACCCTAGGTTTCGGGGCGTATATGTCTAAGGCACATGGTGTCGACCCTAACATCAGAACTGGGGTAAGGACCATCACCACGGGCGCCCCCATTACGTAYTCCACYTATGGCAAGTTTCTTGCCGACGGTGGTTGYTCTGGGGGCGCCTATGACATCATAATWTGTGAYGAGTGCCACTCAACTGACTCGACTACCATCTTGGGCATCGGCACAGTCCTGGACCAAGCGGAGACGGCTGGAGCGCGACTCGTCGTACTCGCCACCGCTACGCCTCCGGGATCGGTCACCGTGCCACATCCCAATATTGAGGAGGTGGCTCTGTCCAMCATTGGAGAGATCCCCTTCTATGGTAAAGCCATCCCCATCGANNNNNNNNNNNNNNNNNNNNNNNNNNNNNNNNNNNNNNNNNNNNNNAAATGTGATGAACTCGCCGCAAAGTTGTCGGGCCTCGGRCTTAACGCYGTAGCGTATTACCGGGGYCTTGATGTGTCCGTCATACCGACCAGCGGAGACGTCGTTGTCGTGGCAACWGATGCTCTAATGACGGGTTWYACYGGCGACTTTGACTCAGTGATCGACTGTAATACATGTGTCACCCAGACAGTCGACTTCAGCTTGGACCCTACCTTCACCATTGAGACRACGACCGTGCCCCAAGACGCGGTGTCGCGCTCGCAGCGGCGAGGCAGGACTGGYAGGGGCAGGGCAGGCATCTACAGGTTTGTGACTCCAGGAGAACGGCCCTCGGGCATGTTCGATTCCTCGGTCCTGTGTGAGTGCTATGACGCGGGCTGTGCTTGGTACGAGCTCACGCCCGCCGAGACCTCAGTTAGGTTACGGGCTTACATGAATACACCAGGGTTGCCCGTCTGCCAGGACCATCTGGAGTTCTGGGAGGGCGTCTTCACAGGCCTCACCCACATAGATGCCCACTTCCTGTCCCAGACTAAACAGGCAGGAGACAAYTTCCCCTACTTGGTRGCATACCARGCTACGGTGTGCGCCAGGGCTCAGGCTCCACCCCCATCGTGGGACCAAATGTGGAAATGTCTCATACGGCTAAAGCCCACGCTGCACGGGCCAACACCCCTGCTGTATAGGCTAGGAGCCGTCCAAAATGARGTCACCCTCACACACCCCGTGACCAAATTCATCATGGCATGCATGTCGGCTGACCTGGAGGTCGCCACG

>HM106708

GCGCCCATYACGGCCTATGCYCAACAGACRCGGGGCCTACTYGGCTGCATCGTCACCAGCCTCACRGGCCGGGACAAGAACCAGGTCGAGGGGGAGGTTCAAGTGGTTTCCACCGCAACACAATCTTTCCTGGCGACCTGCGTCAACGGCGTGTGTTGGACTGTCTACCACGGCGCCGGCGCAAGGACCCTCGCCGGCCAAAAGGGCCCAATCACCCAAATGTACACCAATGTGGACCAGGACCTCGTCGGCTGGCAAGCGCCTCCCGGGGCGCGATCCTTGACACCRTGCACCTGTGGCAGCTCGGACCTTTACTTGGTCACGAGGCAYGCCGATGTTATTCCGGTGCGCCGGCGGGGCGACAGCAGAGGAAGCCTACTCTCCCCCAGGCCCGTCTCCTACTTGAAGGGTTCTTCGGGTGGACCACTGCTCTGCCCCTCGGGGCATGCTGTGGGCATCTTCCGGGCCGCTGTGTGCACCCGGGGGGTTGCGAAGGCGGTTGACTTTGTGCCCGTTGAGTCTATGGAAACAACTATGCGGTCCCCGGTCTTCACGGACAACTCGTCCCCCCCGGCCGTACCGCAGACATTTCAAGTGGCCCATCTACACGCTCCCACCGGCAGCGGCAAGAGCACTAAGGTGCCGGCYGCGTATGCAGCCCAAGGGTACAAGGTACTCGTCCTGAACCCGTCTGTTGCCGCCACCYTAGGTTTCGGGGCGTATATGTCTAAGGCACATGGTGTCGACCCTAACATCAGAACTGGGGTAAGAACCATCACCACGGGTGCCCCCATTACGTACTCCACCTATGGCAAGTTTCTTGCCGACGGTGGTTGCTCTGGGGGCGCCTATGACATCATAATGTGTGATGAGTGCCACTCAACTGACTCGACTACCATCTTGGGCATCGGCACAGTCCTGGACCAAGCGGAGACGGCTGGAGCGCGACTCGTCGTACTCGCCACCGCTACGCCTCCGGGATCGGTCACCGTGCCACATCCCAATATCGAGGAGGTGGCTCTGTCCAACACTGGAGAGATCCCCTTCTATGGTAAAGCCATCCCCATCGAGACCATCAAGGGGGGGAGGCACCTCATATTTTGCCATTCCAAGAAAAAATGTGATGAGCTCGCCGCAAAGCTGTCGGGTCTCGGGCTTAACGCTGTAGCGTATTACCGGGGTCTTGATGTGTCCGTCATACCGACCAGCGGAGACGTCGTTGTCGTGGCAACAGACGCCCTAATGACGGGTTTCACTGGCGACTTTGACTCAGTGATCGACTGTAATACATGTGTCACCCAGACAGTCGACTTTAGCTTGGACCCTACCTTCACCATTGAGACGACGACCGTGCCCCAAGACSCGGTGTCGCGCTCGCAGCGGCGAGGCAGGACTGGTAGGGGCAGGACAGGCACCTATAGGTTTGTGACTCCAGGGGAACGGCCCTCGGGCATGTTCGATTCCTCGGTCCTGTGTGAGTGCTATGATGCGGGCTGTGCTTGGTACGAGCTCACGCCCGCCGAGACCTCAGTTAGGTTGCGGGCTTACCTGAATACACCAGGGTTGCCCGTCTGCCAGGACCATCTGGAGTTCTGGGAGGGCGTCTTCACAGGCCTCACCCACATAGATGCCCACTTCCTGTCCCAGACTAAGCAGGCAGGAGATAACTTCCCCTACTTGGTAGCATACCAGGCTACGGTGTGCGCCAGGGCCCAGGCTCCACCCCCGTCGTGGGACCAAATGTGGAAATGTCTCATACGGCTAAAGCCCACGCTGCACGGGCCAACACCCCTGCTGTATAGGCTAGGAGCCGTCCAAAATGAGGTCACCCTCACACACCCCATAACCAAGTACATCATGGCATGCATGTCAGCTGATCTGGAGATCGCCACK

>HM106709

NNNNNNNNNNNNNNNNNNNNNNNNNNNNNNNNNNNNNNNNNNNNNNNNNNNNNNNNNNNNNNNNNNNNNNNNNNNNNNNNNNNNNNNNNNNNNNNNNNNNNNNNNNNNNNNNNNNNNNNNNNNNNNNNNNNNNNNNNNNNNNNNNNNNNNNNNNNNNNNNNNNNNNNNNNNNNNNNNNNNNNNNNNNNNNNNNNNNNNNNNNNNNNNNNNNNNACCCAAATGTACACCAATGTAGACCAGGACCTCGTCGGCTGGCAAGCGCCCTCCGGGGCGCGATCCTTGACACCGTGCACCTGTGGCAGCTCGGACCTTTACTTGGTCACGAGGCATGCTGATGTCATTCCGGTGCGCCGGCGGGGCGACAGCAGAGGAAGCCTACTCTCCCCCAGGCCCGTCTCCTACTTGAAGGGCTCTTCGGGCGGTCCACTGCTCTGCCCCTCGGGACATGCTGTGGGCATCTTCCGGGCTGCYGTGTGCACCCGGGGGGTCGCGAAGGCGGTGGACTTTGTGCCCGTTGAGTCTATGGAAACAACTATGCGGTCTCCGGTCTTCACGGACAACTCGTCCCCCCCGGCCGTACCGCAGACATTCCAAGTGGCCCATCTACACGCTCCCACTGGTAGCGGCAAGAGCACTAAGGTGCCGGCTGCATATGCGGCCCAAGGGTACAAGGTACTCGTCCTGAACCCGTCYGTTGCCGCCACCCTAAGTTTCGGGGCGTAYATGTCCAAGGCACATGGTGTCGACCCTAACATCAGAACTGGGGTAAGGACCATCACCACGGGTGCCCCCATTACGTACTCCACCTATGGCAAGTTCCTTGCCGACGGTGGTTGCTCTGGGGGCGCCTACGACATCATAATATGTGATGAGTGCCACTCAACTGACTCGACTACCATCCTGGGCATCGGCACAGTCCTGGACCAAGCGGAGACGGCTGGAGCRCGACTCGTCGTRCTCGCCACCGCTACGCCTCCGGGATCRGTCACCGTGCCACNNNNNNNNNNNNNNNNNNNNNNNNNNNNNNNNNNNNNNNNNNNNNNNNNNNNNNNNNNNNNNNNNNNNNNCGAGACCATCAAGGGGGGGAGGCACCTCATATTTTGCCATTCCAGRAAAAAATGTGATGAGCTCGCTGCGAAGCTGTCGAGCCTCGGGCTTAACGCTGTAGCGTATTACCGAGGTCTTGACGTGTCCGTCATACCGACCAGCGGAGACGTCGTTGTCGTGGCAACAGACGCTCTAATGACGGGCTTCACTGGCGACTTTGACTCAGTGATCGACTGTAATACATGTGTCACCCAGACAGTCGACTTCAGTTTGGACCCTACCTTCACCATTGAGACGACGACCGTRCCCCAAGACGCGGTGTCGCGCTCGCAGCGGCGAGGCAGGACTGGTAGGGGCAGGTCAGGCATCTAYAGGTTTGTGACTCCAGGAGAGCGGCCCTCGGGCATGTTCGATTCCTCGGTCCTGTGTGAGTGCTATGACGCGGGCTGTGCTTGGTACGAGCTCACGCCCGCCGAGACCACAGTTAGGTTGCGGGCCTACCTGAATACACCAGGGTTGCCCGTCTGCCAGGACCATCTGGAGTTCTGGGAGGGCGTCTTCACAGGCCTCACCCACATAGATGCCCACTTCCTGTCCCAGACYAAGCAGGCAGGAGACAATTTCCCCTACTTGGTAGCATACCAGGCTACGGTGTGCGCCAGGGCTCAGGCTCCACCCCCATCGTGGGACCAAATGTGGAAATGTCTCATTCGGCTAAAGCCYACRCTGCACGGGCCAACGCCCCTGCTGTATAGGCTAGGAGCCGTCCAAAACGANNNNNNNNNNNNNNNNNNNNNNNNNNNNNNNNNNNNNNNNNNNNNNNNNNNNNNNNNNNNNNNNNNNNNN

>HM106710

GCGCCCATTACGGCCTAYGCCCAACAGACGCGGGGCCTACTTGGCTGCATCGTCACCAGCCTCACAGGCCGGGACAAGAACCAGGTCGAGGGGGAGGTTCAAGTAGNNNNNNNNNNNNNNNNNTCTTTCCTGGCGACCTGCGTCAACGGCGTGTGCTGGACTGTCTACCACGGYGCCGGCGCAAGGACCCTCGCCGGCCAAAAGGGCCCAATCACCCAAATGTACACCAATGTAGACCAGGACCTCGTCGGCTGGCAAGCGCCTCCCGGGGCGCGATCCTTGACACCGTGCACCTGTGGCAGCTCGGACCTTTACCTGGTCACGAGGCATGCTGATATCATTCCGGTGCGCCGGCGGGGCGACAGCAGAGGAAGCCTACTCTCCCCCAGGCCCATCTCCTACTTGAAGGGCTCTTCGGGTGGTCCACTGCTCTGCCCCTCGGGGCATGCTGTGGGCATCTTCCGGGCTGCTGTGTGCACCCGGGGGGTTGCGAAGGCGGTGGACTTTGTGCCCGTTGAGTCTATGGAAACAACTATGCGGTCCCCGGTCTTCACGGACAACTCGACCCCCCCGGCCGTACCGCAGACATTCCAAGTGGCCCATCTACACGCTCCCACTGGCAGCGGCAAGAGCACTAAGGTGCCGGCTGCRTATGCAGCCCAAGGGTACAAGGTACTCGTCCTGAACCCGTCTGTTGCCGCYACCCTAGGTTTCGGGGCGTATATGTCTAAGGCACATGGTGTTGACCCTAACATCAGAACTGGGGTAAGGACCATCACCACGGGTGCCCCCATTACGTACTCCACCTATGGCAAGTTTCTTGCCGACGGTGGCTGTTCTGGGGGCGCCTATGACATCATAATATGTGATGAGTGCCACTCAACTGACTCGACTACCATCTTGGGCATCGGCACAGTCCTGGACCAAGCGGAGACGGCTGGAGCGCGACTCGTCGTACTCGCCACCGCTACGCCTCCGGGATCGGTCACCGTGCCRCATTCCAATATTGAGGAGGTGGCTCTGTCCAACGTTGGAGAGGTCCCCTTCTATGGTAAAGCCATCCCCATCGAGNNNNNNNNNNNNNNNNNNNNNNNNNNNNNNNNNNNNNNNNNNNNNNNNNNNNNNNNNNNNNNNNNNNNNNNNNNNNNNNNNNNNNNNNNNNNNNNNNNNNNNNNNNNNNNNNNNNNNNNNNNNNNNNNNNNNNNNNNNNNNNNNNNNNNNNNNNNNNNNNNNNNNNNNNNNNNNNNNNNNNNNNNNNNNNNNNNNNNNNNNNNNNNNNNNNNNNNNNNNNNNNNNNNNNNNNNNNNNNNNNNNNNNNNNNNNNNNNNNNNNNNNNNNNNNNNNNNNNNNNNNNNNNNNNNNNNNNNNNNNNNNNNNNNNNNNNNNNNNNNNNNNNNNNNNNNNNNNNNNNNNNNNNNNNNNNNNNNNNNNNNNNNNNNNNNNNNNNNNNNNNNNNNNNNNNNNNNNNNNNNNNNNNNNNNNNNNNNNNNNNNNNNNNNNNNNNNNNNNNNNNNNNNNNNNNNNNNNNNNNNNNNNNNNNNNNNNNNNNNNNNNNNNNNNNNNNNNNNNNNNNNNNNNNNNNNNNNNNNNNNNNNNNNNNNNNNNNNNNNNNNNNNNNNNNNNNNNNNNNNNNNNNNNNNNNNNNNNNNNNNNNNNNNNNNNNNNNNNNNNNNNNNNNNNNNNNNNNNNNNNNNNNNNNNNNNNNNNNNNNNNNNNNNNNNNNNNNNNNNNNNNNNNNNNNNNNNNNNNNNNNNNNNNNNNNNNNNNNNNNNNNNNNNNNNNNNNNNNNNNNNNNNNNNNNNNNNNNNNNNNNNNNNNNNNNNNNNNNNNNNNNNNNNNNNNNNNNNNNNNNNNNNNNNNNNNNNNNNNNNNNNNNNNNNNNNNNNNNNNNNN

>HM106711

GCGCCCATCACGGCCTATGCCCAACAGACGCGGGGCCTATTTGGCTGCATCGTCACCAGCCTCACAGGCCGGGACAAGAACCAGGTTGAGGGGGAGGTTCAAGTGGTTTCCACCGCAACACAAACTTTCCTGGCGACCTGTGTCAATGGCGTGTGCTGGACTGTCTACCACGGCGCCGGCGCAAGGACYCTCGCCGGCCAAAAAGGCCCGATCACCCAAATGTACACCAATGTGGACCAGGACCTCGTCGGCTGGCAAGCGCCCCCCGGGGCGCGATCCTTGACACCGTGCACCTGCGGCAGCTCAGACCTTTACTTGGTCACGAGGCATGCTGATGTCATTCCGGTGCGCCGGCGGGGCGACAGCAGRGGAAGYCTACTCTCCCCCAGGCCCGTCTCCTACTTGAAGGGCTCTTCGGGTGGTCCACTGCTCTGCCCCTCGGGGCATGCTGTGGGCATCTTCCGGGCTGCTGTGTGCACCCGGGGGGTTGCGAAGGCGGTGGACTTTGTGCCCGTTGAGTCTATGGAAACRACTATGCGGTCCCCGGTCTTCACGGACAACTCGTCCCCCCCGGCCGTACCGCAGACATTCCAAGTGGCCCATCTACACGCTCCCACTGGCAGCGGCAAGAGCACTAAGGTGCCGGCTGCGTATGCAGCCCAAGGGTACAAGGTACTCGTCCTGAACCCGTCCGTTGCCGCCACCCTGGGTTTTGGGGCGTATATGTCTAAGGCACATGGTGTCGACCCYAACATCAGAACTGGGGTAAGGACCATCACCACGGGTGCCCCCATTACGTACTCCACCTATGGCAAGTTCCTTGCCGACGGTGGTTGTTCGGGGGGCGCCTATGACATCATAATATGTGATGAGTGCCACTCAACTGACTCGACTACCATCTTGGGCATCGGCACAGTCCTGGACCAAGCGGAGACGGCTGGAGCGCGACTYGTCGTACTCGCCACCGCTACGCCTCCGGGATCGGTCACCGTGCCACATCCCAATATTGAGGAGGTGGCTCTGTCCAACATTGGAGAGATCCCCTTCTATGGCAAAGCCATCCCCATCGAGNNNNNNNNNNNNNNNNNNNNNNNNNNNNNNNNNNNNNNNNNNNNNNNNNNNNNNNNNNNNNNNNNNNNNNNNNNNNNNNNNNNNNNNNNNNNNNNNNNNNNNNNNNNNNNNNNNNNNNNNNNNNNNNNNNNNNNNNNNNNNNNNNNNNNNNNNNNNNNNNNNNNNNNNNNNNNNNNNNNNNNNNNNNNNNNNNNNNNNNNNNNNNNNNNNNNNNNNNNNNNNNNNNNNNNNNNNNNNNNNNNNNNNNNNNNNNNNNNNNNNNNNNNNNNNNNNNNNNNNNNNNNNNNNNNNNNNNNNNNNNNNNNNNNNNNNNNNNNNNNNNNNNNNNNNNNNNNNNNNNNNNNNNNNNNNNNNNNNNNNNNNNNNNNNNNNNNNNNNNNNNNNNNNNNNNNNNNNNNNNNNNNNNNNNNNNNNNNNNNNNNNNNNNNNNNNNNNNNNNNNNNNNNNNNNNNNNNNNNNNNNNNNNNNNNNNNNNNNNNNNNNNNNNNNNNNNNNNNNNNNNNNNNNNNNNNNNNNNNNNNNNNNNNNNNNNNNNNNNNNNNNNNNNNNNNNNNNNNNNNNNNNNNNNNNNNNNNNNNNNNNNNNNNNNNNNNNNNNNNNNNNNNNNNNNNNNNNNNNNNNNNNNNNNNNNNNNNNNNNNNNNNNNNNNNNNNNNNNNNNNNNNNNNNNNNNNNNNNNNNNNNNNNNNNNNNNNNNNNNNNNNNNNNNNNNNNNNNNNNNNNNNNNNNNNNNNNNNNNNNNNNNNNNNNNNNNNNNNNNNNNNNNNNNNNNNNNNNNNNNNNNNNNNNNNNNNNNNNNNNNNNNNNNNNNNNNNNNNNNNNN

>HM106712

GCGCCCATCACGGCCTATGCCCAACAGACGCGGGGCCTACTTGGCTGCATCGTCACCAGCCTAACAGGCCGGGACAAGAACCAGGTCGAGGGGGAGGTTCAAGTGGTTTCTACCGCAACACAATCTTTCCTGGCGACCTGCGTCAGCGGCGTGTGTTGGACTGTCTACCACGGCGCTGGCACAAAGACCCTCGCCGGCCAAAAAGGCCCAATCACCCAAATGTACACCAATGTAGACCAGGACCTCGTCGACTGGCAAGCGCCTCCTGGGGCGCGATCCTTGACACCGTGCACCTGTGGCAGCTCGGACCTTTACTTGGTCACGAGGCATGCTGATGTCATTCCAGTGCGCCGGCGGGGCGACAGCAGAGGAAGCCTACTCTCCCCCAGGCCCGTCTCCTACTTGAAGGGTTCTTCGGGTGGTCCACTGCTCTGCCCCTCGGGGCATGCTGTGGGCGTCTTCCGGGCTGCTGTGTGCACCCGAGGGGTTGCGAAGGCAGTGGACTTTGTGCCCGTCGAGTCTATGGAAACAACAATGCGGTCTCCGGTCWTCACGGACAATTCGTCCCCCCCGGCCGTACCGCAGACATTCCAAGTGGCCCATCTACACGCCCCCACTGGCAGCGGCAAGAGCACCAAGGTGCCGGCTGCGTATGCAGCCCAGGGGTATAAGGTACTCGTCCTGAACCCGTCTGTTGCCGCCACCCTGAGTTTCGGGGCGTATATGTCTAAGGCATATGGTATCGACCCTGGCATCAGAACTGGGGTAAGGACCATCACCACGGGCGCCCCCATCACGTACTCCACCTATGGCAAGTTCCTTGCCGACGGTGGTTGCTCTGGGGGCGCCTATGACATCATAATATGTGATGAGTGCCACTCAACTGACTCGACTACCATCTTGGGCATCGGCACGGTCCTTGACCAAGCGGAGACGGCTGGAGCGCGACTCGTCGTGCTCGCCACCGCTACGCCTCCGGGATCCGTCACCGTGCCACANNNNNNNNNNNNNNNNNNNNNNNNNNNNNNNNNNNNNNNNNNNNNNNNNNNNNNNNNNNNNNNNNNNNNNNNNNNNATCAAGGGGGGGAGGCACCTCATATTTTGCCATTCCAGGAAAAAATGTGATGAGCTCGCCGCAAAGCTGTCGAGTCTCGGGCTTAACGCTGTAGCGTATTACCGGGGTCTTGATGTGTCCGTCATACCGACCAGCGGAGACGTCGTTGTCGTGGCAACAGACGCTCTAATGACGGGTTTCACTGGCGACTTTGACTCAGTGATCGACTGTAACACGTGTGTCACCCAGACAGTCGACTTCAGTTTGGACCCTACCTTCACCATTGAGACGACGACTGTGCCCCAAGACGCGGTGTCGCGCTCGCAGCGGCGAGGCAGGACTGGTAGGGGCAGGACAGGCATTTACAGGTTTGTGACTCCAGGAGAACGGCCCTCGGGCATGTTCGATTCCTCGGTCCTGTGYGAGTGTTATGACGCGGGCTGTGCTTGGTACGAGCTCACGCCCGCCGAGACCTCAGTTAGGTTGCGGGCTTACCTGAATACACCAGGGTTGCCCGTCTGCCAGGACCATCTGGAGTTCTGGGAGGGCGTCTTCACAGGCCTCACCCACATAGATGCCCACTTCCTGTCCCAGACTAAGCAGGCAGGAGACAACTTCCCCTACTTGGTGGCATACCAGGCTACGGTGTGCGCCAGGGCTCAGGCTCMACCCCCATCGTGGGACCAAATGTGGAAATGTCTCATACGGCTAAAGCCYAYGYTGCACGGGCCAACACCCCTGCTGTATAGGCTAGGAGCCGTGCAAAATGAGGTCACCCTCACACACCCCATAACCAAATACATCATNNNNNNNNNNNNNNNNNNNNNNNNNNNNNNNNNN

>HM106713

GCGCCCATCACGGCCTATGCCCAACAGACRCGGGGYCTACTTGGCTGCATCGTCACCAGCCTCACAGGCCGGGACAAGAACCAGGTCGAGGGGGAGGTTCAAGTGGTTTCCACCGCRACACAGTCTTTCCTGGCGACCTGCGTCAACGGCGTGTGTTGGACTGTCTACCACGGCGCCGGCACAAAGACCCTCGCCGGCCAAAAGGGCCCAATCACCCAAATGTACACCAATGTAGACCAGGACCTCGTCGGCTGGCAAGCACCTCCCGGGGCACGATCCTTGACACCGTGCACCTGTGGCAGCTCGGACCTTTACTTGGTCACAAGGCATGCTGATGTCATTCCGGTGCGCCGGCGGGGCGACAGCAGGGGAAGCCTACTCTCCCCCAGGCCCGTCTCCTACTTGAAGGGCTCTTCGGGTGGTCCACTGCTCTGCCCCTCGGGGCATGCTGTGGGCATCTTCCGGGCTGCCGTGTGCACCCGGGGGGTTGCGAAGGCGGTGGACTTTGTACCCGTTGAGTCCATGGAAACAACTATGCGGTCCCCGGTCTTCACGGACAACTCGTCCCCTCCGGCCGTACCGCAGACATTCCAAGTGGCCCATCTACACGCTCCCACTGGCAGCGGCAAGAGCACTAAGGTGCCGGCTGCGTATGCAGCCCAAGGGTACAAGGTACTCGTCCTGAACCCGTCTGTTGCCGCCACCCTAGGTTTCGGGGCGTATATGTCTAAGGCACATGGTGTCGACCCTAACATCAGAACTGGGGTAAGGACCATCACCACGGGTGCCCCCATTACGTACTCCACCTATGGCAAGTTTCTTGCCGACGGTGGTTGCTCTGGGGGCGCCTATGACATCATAATATGTGATGAGTGCCACTCAACTGACTCTACTACCATCCTGGGCATCGGCACAGTCCTGGACCAAGCGGAGACGGCTGGAGCGCGACTCGTCGTACTCGCCACCGCTACGCCTCCGGGATCGGTYACCGTGCCACATCCCAACATCGAGGAGGTGGCTCTGTCCAACACTGGGGAGATCCCCTTCTATGGTAAAGCCATCCCCATCGAGACCATCAAAGGGGGGAGGCACCTCATNNNNNNNNNNNNNNNNNNNNNNNNNNNNNNNNNNNNNNNNNNNNNNNNNNNNCTCGGGCTTAACGCTGTGGCGTATTACCGGGGTCTTGACGTATCCGTCATACCGACCAGCGGAGACGTCGTTGTCGTGGCAACAGACGCTCTAATGACGGGTTTCACTGGCGACTTTGACTCAGTGATCGACTGTAATACATGTGTCACCCAGACAGTCGACTTCAGCTTGGACCCTACCTTCACCATTGAGACGACGACCGTGCCCCAAGACGCGGTGTCGCGCTCGCAGCGGCGAGGCAGGACTGGTAGGGGCAGGGCAGGCATCTACAGGTTTGTAACTCCAGGAGAACGGCCCTCGGGCATGTTCGATTCCTCGGTCCTATGTGAGTGCTATGACGCGGGCTGTGCTTGGTATGAGCTCACGCCCGCCGAGACCTCAGTTAGGTTGCGGGCTTACCTGAATACACCAGGGTTGCCCGTCTGCCAGGACCACCTGGAGTTCTGGGAGGGCGTCTTCACAGGCCTCACCCACATAGATGCCCACTTCCTGTCCCAGACCAAGCAGGCAGGAGACAACTTCCCCTACCTGGTAGCATACCAGGCTACGGTGTGCGCCAGGGCTCAGGCTCCACCCCCATCGTGGGACCAAATGTGGAAATGTCTCATACGGCTGAAGCCCACGCTGCACGGGCCAACACCCCTGCTGTATAGGCTAGGAGCCGTCCAAAATGAGGTCATCCTCACACACCCCATAACCAAATACATCATGACATGCATGTCGGCTGACCTGGAGGTCGTCACG

>HM106714

GCGCCCATCACGGCCTATGCCCAACAGACGCGGGGCCTACTTGGCTGCATTGTCACCAGCCTCACAGGCCGGGATAAGAACCAGGTCGAGGGGGAGGTTCAGGTGGTCTCCACCGCAACACAATCTTTCCTGGCGACTTGCGTCAACGGCGTGTGCTGGACTGTCTACCACGGCGCCGGCGCGAGGACCCTCGCCGGCCAAAAGGGCCCAATCACCCAAATGTACACCAATGTAGACCAAGACCTCGTCGGCTGGCAAGCGCCTCCCGGGGCGCGATCCTTGACACCGTGCACCTGTGGCAGCTCGGACCTTTACTTGGTCACGAGRCATGCTGATGTTATTCCGGTGSGCCGGCGGGGCGACAGCAGAGGAAGCCTACTCTCCCCCAGGCCCGTCTCCTACTTGAAGGGCTCTTCGGGTGGCCCACTGCTCTGTCCCTCGGGGCATGCTGTGGGCATCTTCCGGGCTGCTGTGTGCACCCGGGGGGTTGCGAAGGCGGTGGACTTTGTGCCCGTTGAGTCTATGGAAACAACTATGCGGTCCCCGGTCTTCACGGACAACTCGTCCCCTCCGGCCGTACCGCAGACATTCCAGGTGGCCCATCTACACGCTCCCACTGGCAGCGGCAAGAGCACTAAGGTGCCGGCTGCGTATGCAGCCCAAGGGTACAAGGTACTAGTCCTGAACCCGTCCGTTGCCGCAACCCTAGGYTTCGGGGCGTATATGTCTAAGGCACATGGTGTCGACCCTAACATCAGAACTGGRATAAGGACCATCACCACGGGTGCCCCCATTACGTACTCCACCTATGGCAAGTTYCTTGCCGACGGTGGTTGCTCTGGGGGCGCCTATGACATCATAATATGTGATGAGTGCCACTCAACTGACTCGACTACTATCTTGGGCATCGGCACAGTCCTGGACCAAGCGGAGACGGCTGGAGCGCGACTTGTCGTACTCGCCACCGCTACGCCTCCGGGATCRGTCACCGTGCCACATCCCAATATTGAGGAGGTGGCTCTGTCCAACACTGGAGAGATCCCCTTCTATGGTAAAGCCATCCCCATCGAGACCATCAAGGGGGGAAGGCACCTCATNNNNNNNNNNNNNNNNNNNNNNNNNNNNNNNNNNNNNNNNNNNNNNNNNNNNNNNNNNNNNNNNNNNNNNNNNNNNNNNNNNNNNNNNNNNNNNNNNNNNNNNNNNNNNNNNNNNNNNNNNNNNNNNNNNNNNNNNNNNNNNNNNNNNNNNNNNNNNNNNNNNNNNNNNNNNNNNNNNNNNNNNNNNNNNNNNNNNNNNNNNNNNNNNNNNNNNNNNNNNNNNNNNNNNNNNNNNNNNNNNNNNNNNNNNNNNNNNNNNNNNNNNNNNNNNNNNNNNNNNNNNNNNNNNNNNNNNNNNNNNNNNNNNNNNNNNNNNNNNNNNNNNNNNNNNNNNNNNNNNNNNNNNNNNNNNNNNNNNNNNNNNNNNNNNNNNNNNNNNNNNNNNNNNNNNNNNNNNNNNNNNNNNNNNNNNNNNNNNNNNNNNNNNNNNNNNNNNNNNNNNNNNNNNNNNNNNNNNNNNNNNNNNNNNNNNNNNNNNNNNNNNNNNNNNNNNNNNNNNNNNNNNNNNNNNNNNNNNNNNNNNNNNNNNNNNNNNNNNNNNNNNNNNNNNNNNNNNNNNNNNNNNNNNNNNNNNNNNNNNNNNNNNNNNNNNNNNNNNNNNNNNNNNNNNNNNNNNNNNNNNNNNNNNNNNNNNNNNNNNNNNNNNNNNNNNNNNNNNNNNNNNNNNNNNNNNNNNNNNNNNNNNNNNNNNNNNNNNNNNNNNNNNNNNNNNNNNNNNNNNNNNNNNNNNNNNNNNNNNNNNNNNNNNNNNNNNNNNNNNNNNNNNNNNNNNNNNNNNNNNNNNNNNNNNNNNNNN

>HM106715

GCGCCCATCACGGCYTATGCCCAACAGACGCGRGGCYTGCTYGGCTGTATCGTCACCAGCCTCACAGGCCGGGACAAGAACCAGGTCGAGGGGGAGGTTCAAGTGGTTTCCACCGCAACACAATCTTTCCTGGCGACCTGYGTCAACGGCGTGTGTTGGACTGTCTACCACGGCGCCGGCRCRAGGACCCTYGCCGGCCAAAAGGGCCCAATCACCCAAATGTACACCAATGTAGACCAGGACCTCGTCGGCTGGCAAGCGCCCCCCGGGGCGCGATCCTTGACACCSTGCACCTGTGGCAGCTCGGACCTTTACYTGGTCACGAGGCATGCTGATGTCATTCCGGTGCGCCGGCGGGGCGACAGCAGAGGAAGCCTRCTCTCCCCCAGGCCCGTCTCCTACTTGAAGGGCTCTTCGGGTGGTCCRCTGCTCTGCCCCTCGGGGCATGCYGTGGGCATCTTCCGRGCYGCTGTGTGCACCCGGGGGGTTGCRAAGGCGGTGGACTTCGTRCCCGTTGAGTCTATGGAAACAACYATGCGGTCYCCGGTCTTCACGGACAACTCGTCCCCCCCSGCCGTACCGCAGACATTCCAAGTGGCCCAYCTRCACGCTCCCACTGGCAGCGGCAARAGCACYAAGGTGCCGGCTGCGTATGCAGCCCAAGGGTACAAGGTACTYGTCCTGAACCCGTCYGTTGCCGCCACCCTAGGTTTYGGGGCGTATATGTCTAAGGCACATGGTGTCGACCCYAACATCAGAACTGGGGTAAGGACCATCACCACGGGTGCCCCCATTACGTACTCCACCTATGGCAAGTTCCTYGCCGAYGGTGGYTGCTCTGGGGGCGCCTATGACATCATAATATGTGATGAGTGCCACTCAACTGACTCGACTACCATCTTGGGCATTGGCACAGTCCTGGACCAAGCGGAGACGGCTGGAGCGCGACTYGTCGTACTCGCCACCGCTACRCCTCCGGGATCGGTCACCGTGCCACATCCCAATATCGAGGAGGTGGCTCTGTCCAACATTGGAGAGATCCCCTTCTACGGTAAAGCCATCCCCATYGAGACCATCAAGGGGGGGAGGCACCTCNNNNNNNNNNNNNNNNNNNNNNNNNNTGATGAGCTCGCCGCAAAGCTGTCGGGCCTCGGGCTTAACGCTGTAGCGTATTACCGGGGTCTTGATGTGTCCGTCATACCGACCAGCGGAGACGTCGTTGTCGTGGCAACGGACGCTCTAATGACGGGTTTCACTGGCGATTTTGACTCAGTGATCGACTGCAATACATGTGTCACCCARACAGTCGACTTCAGCTTGGACCCTACCTTCACCATTGAGACGACGACCGTGCCCCAAGACGCGGTATCGCGCTCGCAGCGGCGAGGCAGGACTGGTAGGGGCAGGGCAGGCATCTACAGGTTTGTGACTCCAGGAGAACGGCCCTCGGGCATGTTCGATTCCTCGGTCCTGTGTGAGTGCTATGACGCGGGCTGTGCTTGGTACGAGCTCACGCCCGCCGAGACCACAGTTAGGTTGCGGGCTTACCTGAATACACCAGGGTTGCCCGTCTGCCAGGACCATCTGGAGTTCTGGGAGGGCGTCTTCACAGGCCTCACCCACATAGATGCCCACTTCCTGTCCCAGACTAAGCAGGCAGGAGACAACTTCCCCTACTTGGTAGCATACCAGGCTACAGTGTGCGCCAGGGCTCAGGCTCCACCTCCATCGTGGGACCAAATGTGGAAGTGTCTCATACGGCTAAAGCCCACGCTGCACGGGCCAACACCCCTGCTGTATAGGCTAGGAGCCGTCCAAAATGAGGTCACCCTCACACACCCCATAACCAAACTCATCATGGCATGCATGTCGGCTGACCTGGAGGTCGCCACG

>HM106716

GCGCCCATCACGGCCTATGCCCAACAGACGCGGGGCCTACTTGGCTGCATCGTCACCAGCCTCACAGGCCGGGACAGGAACCAGGTCGAGGGGGAGGTTCAAGTGGTTTCCACTGCAACACAATCTTTCCTGGCGACCTGCATCAACGGCGTGTGTTGGACTGTCTACCACGGTGCCGGCACAAAGACCCTCGCCGGCCAAAAGGGSCCAATCACCCAAATGTACACCAATGTAGACCAGGACCTCGTCGGCTGGCAAGCGCCTCCCGGGGCGCGATCMTTGACACCGTGCACTTGTGGCAGCTCGGACCTYTACYTGGTTACGAGGCATGCTGATGTCATYCCGGTGSGCCGGCGGGGCGACAGCAGAGGAAGCCTACTCTCCCCCAGGCCCRTCTCCTACTTGAAGGGCTCTTCGGGTGGTCCACTGCTCTGCCCCTCGGGGCATGCTGTRGGCATCTTCCGGGCTGCTGTGTGCACCCGGGGGGTTGCGAAGGCRGTGGACTTTGTGCCTGTTGAGTCTATGGAAACAACTATGCGGTCCCCGGTCTTCACGGACAACTCGTCCCCCCCGGCCGTACCGCAGACATTCCAAGTGGCCCATCTACACGCCCCCACTGGCAGYGGCAAGAGCACTAAGGTGCCGGCTGCGTATGCAGCCCAAGGGTACAAGGTACTCGTCYTGAACCCGTCTGTYGCCGCCACCYTAGGKTTCGGGGCGTATATGTCYAAGGCACATGGTGTCGACCCTAACATCAGAACTGGGGTAAGGACCATCACCACGGGTGCCCCCATYACGTAYTCCACCTATGGCAAGTTYCTTGCCGACGGCGGTTGCTCTGGGGGCGCCTMTGACATCATAATATGTGATGAGTGCCACTCAACTGACTCGACTACCATCTTGGGCATCGGCACAGTCCTGGACCAAGCGGAGACGGCTGGAGCGCGACTCGTCGTACTCGCCACCGCTACRCCTCCGGGATCGGTCACCGTGCCACAYCCCAATATCGAGGAGGTGGCTCTGTCCAACATTGGAGAGATCCCCTTCTNNNNNNNNNNNNNNNNNNNNNNNNNNNNNNNNNNNNNNNNNNNNNNNNNNNNNNNNNNNNNNNNNNNNNNNNNNNNNNNNNNNNNNNNNNNNNNNNNNNNNNNNNNNNNNNNNNNNNNNNNNNNNNNNNNNNNNNNNNNNNNNNNNNNNNNNNNNNNNNNNNNNNNNNNNNNNNNNNNNNNNNNNNNNNNNNNNNNNNNNNNNNNNNNNNNNNNNNNNNNNNNNNNNNNNNNNNNNNNNNNNNNNNNNNNNNNNNNNNNNNNNNNNNNNNNNNNNNNNNNNNNNNNNNNNNNNNNNNNNNNNNNNNNNNNNNNNNNNNNNNNNNNNNNNNNNNNNNNNNNNNNNNNNNNNNNNNNNNNNNNNNNNNNNNNNNNNNNNNNNNNNNNNNNNNNNNNNNNNNNNNNNNNNNNNNNNNNNNNNNNNNNNNNNNNNNNNNNNNNNNNNNNNNNNNNNNNNNNNNNNNNNNNNNNNNNNNNNNNNNNNNNNNNNNNNNNNNNNNNNNNNNNNNNNNNNNNNNNNNNNNNNNNNNNNNNNNNNNNNNNNNNNNNNNNNNNNNNNNNNNNNNNNNNNNNNNNNNNNNNNNNNNNNNNNNNNNNNNNNNNNNNNNNNNNNNNNNNNNNNNNNNNNNNNNNNNNNNNNNNNNNNNNNNNNNNNNNNNNNNNNNNNNNNNNNNNNNNNNNNNNNNNNNNNNNNNNNNNNNNNNNNNNNNNNNNNNNNNNNNNNNNNNNNNNNNNNNNNNNNNNNNNNNNNNNNNNNNNNNNNNNNNNNNNNNNNNNNNNNNNNNNNNNNNNNNNNNNNNNNNNNNNNNNNNNNNNNNNNNNNNNNNNNNNNNNNNNNNNNNNNNNN

>HM106717

GCGCCCATCACGGCCTATGCCCAACAGACRCGGGGCCTACTGGGTTGCATCGTCACCAGCCTCACAGGCCGGGACAARAACCAGGTSGAGGGGGAGGTYCAAGTGGTTTCCACCGCAACACAGTCTTTCCTGGCGACCTGCCTCAACGGCGTGTGTTGGACTGTCTACCACGGCGCCGGCACAAAGACCCTCGCCGGCCTAAAGGGCCCAATYACTCAAATGTATACCAATGTAGACCAGGACCTCGTCGGCTGGCAAGCGCCTCCCGGGGCGCGATCCTTGACACCGTGCACCTGTGGCAGCTCGGACCTTTACTTGGTCACGAGGCATGCTGATGTCATTCCGGTGCGCCGGCGGGGCGACAGCAGAGGAAGCCTACTCTCCCCCAGGCCCGTCTCCTACTTAAAGGGCTCTTCGGGTGGTCCACTGCTCTGCCCCTCGGGGCATGCTGTGGGCATCTTCCGGGCTGCTGTGTGCACCCGGGGGGTTGCGAAGGCGGTGGACTTCGTGCCCGTTGAGTCTATGGAAACAACTATGCGGTCCCCGGTCTTCACGGACAACTCGTCCCCCCCTGCCGTACCGCAGACATTCCAAGTGGCCCATTTACACGCTCCCACTGGCAGCGGCAAGAGCACTAAGGTGCCGGCTGCGTATGCGGCCCAAGGGTACAAGGTGCTCGTCTTGAACCCATCTGTTGCCGCCACCCTGAGTTTCGGGGCGTACATGTCTAAGGCACATGGTATCGACCCTGGCATCAGAACTGGGGTAAGGACCATCACCACGGGTGCCCCCATTACGTATTCCACCTATGGCAAGTTTCTTGCCGACGGTGGTTGCTCTGGGGGCGCCTATGACATCATAATATGCGATGAGTGCCACTCAACTGACTCGACTACCATCTTGGGCATCGGCACAGTCCTGGACCAAGCGGAGACGGCTGGAGCGCGACTCGTCGTACTCGCCACCGCTACGCCTCCGGGATCGGTCACCGTGCCACATCCCAATATCGAGGAGGTGGCTCTGTCCAACACTGGAGAGATCCCCTTCTATGGTAAAGCCATCCCCATCGAAACCATCAAGGGGGGGAGGCACCTCATATTTTGCCATTCCAAGAAAAAATGTGATGAGCTCGCCGCAAAGCTATCGGGTCTCGGGCTTAACGCTGTAGCGTACTACCGAGGTCTYGATGTGTCCGTCATACCGACCAGCGGAGACGTCGTTGTCGTGGCAACAGACGCTCTAATGACGGGTTTCACTGGCGACTTTGACTCAGTGATCGACTGTAATACATGTGTCACCCAGACAGTCGACTTCAGCTTGGACCCTACCTTCGCCATTGAGACGACGACCGTGCCCCAGGACGCGGTGTCGCGCTCGCAGCGGCGAGGCAGGACTGGTAGGGGCAGGGCAGGCATCTACAGGTTTGTGACTCCAGGAGAGCGGCCCTCGGGCATGTTCGATTCYTCGGTCCTGTGTGAGTGCTATGACGCGGGCTGTGCTTGGTACGAGCTCACGCCCGCCGAGACCTCGGTTAGGTTGCGGGCCTACCTGAATACACCAGGGTTGCCTGTCTGCCAGGACCATCTGGAGTTCTGGGAGGCCGTCTTCACAGGCCTCACCCACATAGATGCCCACTTCCTGTCCCAGACTAAACAGGCAGGAGACAACTTCCCCTACTTGGTAGCATACCAGGCTACGGTGTGCGCCAGGGCTCAGGCCCCACCCCCATCGTGGGACCAAATGTGGAAGTGTCTCATACGGCTAAAGCCCACGCTGCACGGGCCAACGCCCCTGCTGTATAGGCTAGGAGCCGTACAAAATGAGGTCATCCTTACACACCCCATAACCAAATACATCATGGCATGCATGTCGGCTGACCTGGAGGTCGCCACG

>HM106718

GCGCCCATCACGGCCTATGCCCAACAGACGCGGGGCTTACTTGGCTGCATYRTCACCAGCCTCACAGGCCGAGACAAGAACCAGGTCGARGGGGAGGTTCAAGTGGTTTCCACYGCAACRCAATCTTTCCTGGCGACCTGCGTCAAYGGCGTGTGTTGGACTGTYTACCACGGCGCCGGCACRAAGACCCTCGCCGGCCMRAAGGGCCCAATCACCCAAATGTACACCAATGTRGACCAGGACCTCGTCGGCTGGCAAGCGCCYYCCGGGGCGCGATCCTTGACACCGTGCACCTGTGGMAGCTCGGACCTCTACTTGGTCACGAGGCATRCTGATGTCATTCCGGTGCGCCGGCGGGGCGACAGCAGGGGRAGYYTACTCTCCCCCAGGCCCGTCTCCTACTTGAAGGGCTCTTCGGGTGGTCCACTGCTCTGYCCCTCGGGGCATGCTGTGGGCATCTTCCGGGCTGCYGTGTGCACCCGGGGGGTTGCGAAGGCGGTGGACTTCGTGCCCGTTGAGTCTATGGAGACAACTATGCGGTCCCCGGTCTTCACGGACAACTCGTCCCCCCCGGCCGTACCGCAGACATTCCAAGTGGCCCATCTACACGCTCCCACTGGTAGCGGCAAGAGCACTAAGGTGCCGGCTGCTTATGCAGCCCAAGGGTAYAAGGTACTCGTCCTGAACCCGTCTGTTGCCGCCACCCTAGGKTTCGGGGCGTAYATGTCTAAAGCACATGGTGTCGACCCTAACATCAGAACCGGGGTAAGGACCATCACCACGGGTGCYCCCATTACGTAYTCCACCTATGGCAAGTTTCTTGCCGACGGTGGTTGCTCTGGGGGYGCCTAYGACATCATAATATGTGATGAGTGCCACTCAACTGAYTCGACTACCATCTTRGGYAYCGGCACAGTCCTGGACCAAGCGGAGACGGCTGGAGCRCGACTCGTCGTRCTCGCCACCGCYACGCCTCCGGGATCGGTCACCGTGCCACATCCYAAYATCGAGGAGGTGGCTCTGTCCAACATTGGAGAGATYCCCTTCTATGGYAAAGCCATCCCCATCGAGACCATCAAGGGGGGGAGGCACCTCATATTTTGCCATTCCAAGTANNNNNNNNNNNNNNNNNNNNNNNNNNNNNNNNNNNNNNNNNNNNNNNNNNNNNNNNNNNNNNNNNNNNNNNNNNNNNNNNNNNNNNNNNNNNNNNNNNNNNNNNNNNNNNNNNNNNNNNNNNNNNNNNNNNNNNNNNNNNNNNNNNNNNNNNNNNNNNNNNNNNNNNNNNNNNNNNNNNNNNNNNNNNNNNNNNNNNNNNNNNNNNNNNNNNNNNNNNNNNNNNNNNNNNNNNNNNNNNNNNNNNNNNNNNNNNNNNNNNNNNNNNNNNNNNNNNNNNNNNNNNNNNNNNNNNNNNNNNNNNNNNNNNNNNNNNNNNNNNNNNNNNNNNNNNNNNNNNNNNNNNNNNNNNNNNNNNNNNNNNNNNNNNNNNNNNNNNNNNNNNNNNNNNNNNNNNNNNNNNNNNNNNNNNNNNNNNNNNNNNNNNNNNNNNNNNNNNNNNNNNNNNNNNNNNNNNNNNNNNNNNNNNNNNNNNNNNNNNNNNNNNNNNNNNNNNNNNNNNNNNNNNNNNNNNNNNNNNNNNNNNNNNNNNNNNNNNNNNNNNNNNNNNNNNNNNNNNNNNNNNNNNNNNNNNNNNNNNNNNNNNNNNNNNNNNNNNNNNNNNNNNNNNNNNNNNNNNNNNNNNNNNNNNNNNNNNNNNNNNNNNNNNNNNNNNNNNNNNNNNNNNNNNNNNNNNNNNNNNNNNNNNNNNNNNNNNNNNNNNNNNNNNNNNNNNNNNNNNNNNNNNNNNNNNNNNNNNNNNNNNNNNNNNNNNNNNNNNNNNNNNNNNNNNNNNNNNNN

>HM106719

GCGCCCATCACGGCCTATGCCCAACAGACGCGGGGCCTACTTGGCTGCATCGTCACTAGCCTCACAGGCCGGGACAAGAACCAGGTCGAGGGGGAAGTCCAAGTGGTTTCTACCGCAACACAATCTTTCCTGGCAACCTGCATCAATGGCGTGTGTTGGACTGTCTACCACGGCGCCGGCACAAAGACCCTCGCCGGCCCAAAGGGCCCAATCACCCAAATGTACACCAATGTAGACCAGGACCTCGTCGGCTGGCAAGCGCCTCCCGGGGCGCGATCCTTGACACCGTGCACCTGTGGCAGCTCGGACCTTTACTTGGTCACGAGGCACGCTGATGTCATTCCGGTGCGCCGGCGGGGCGACAGMAGAGGAAGYTTACTCTCCCCCAGGCCTGTCTCCTACTTGAAGGGCTCTTCGGGTGGTCCACTGCTCTGCCCCTTGGGGCATAYTGTGGGCATCTTCCGGGCTGCTGTGTGCACTCGGGGGGTTGCGAAGGCGGTGGACTTTGTGCCCGTTGAGTCCATGGAAACAACTATGCGGTCCCCGGTCTTCACGGACAACTCGTCCCCCCCGGCCGTACCGCAGACATTTCAAGTGGCCCATCTACACGCTCCCACAGGCAGCGGCAAGAGCACCAAGGTGCCGGCTGCGTATGCAGCCCAAGGGTACAAGGTACTCGTCCTGAACCCGTCTGTTGCCGCCACCCTGGGTTTCGGGGCGTATATGTCTAAGGCACATGGTGTCGACCCCAACATCAGAACTGGGGTAAGGACCATCACCACAGGTGCCCCCATCACGTACTCTACYTATGGCAAGTTTCTTGCCGACGGTGGTTGYTCTGGGGGCGCYTATGACATCATAATWTGTGATGAGTGCCACTCAACTGACTCAACTACCATCTTGGGCATCGGCACAGTCCTGGACCAAGCGGAGACGGCTGGAGCGCGACTTGTCGTACTCGCCACCGCTACGCCTCCGGGATCGGTCACCGTGCCACACCCCAACATCGAGGAGGTGGCTCTGTCCAMCACTGGRGAGATCCCCTTCTATGGTAAAGCTATCCCCATCGAGACCATCAAGGGGGGNNNNNNNNNNNNNNNNNNNNNNNNNNNNNNNNNNNNNNNNNNNNNNNNNNNNNNNNNNNNNNNNNNNNNNNNNNNNNNNNNNNNNNNNNNNNNNNNNNNNNNNNNNNNNNNNNNNNNNNNNNNNNNNNNNNNNNNNNNNNNNNNNNNNNNNNNNNNNNNNNNNNNNNNNNNNNNNNNNNNNNNNNNNNNNNNNNNNNNNNNNNNNNNNNNNNNNNNNNNNNNNNNNNNNNNNNNNNNNNNNNNNNNNNNNNNNNNNNNNNNNNNNNNNNNNNNNNNNNNNNNNNNNNNNNNNNNNNNNNNNNNNNNNNNNNNNNNNNNNNNNNNNNNNNNNNNNNNNNNNNNNNNNNNNNNNNNNNNNNNNNNNNNNNNNNNNNNNNNNNNNNNNNNNNNNNNNNNNNNNNNNNNNNNNNNNNNNNNNNNNNNNNNNNNNNNNNNNNNNNNNNNNNNNNNNNNNNNNNNNNNNNNNNNNNNNNNNNNNNNNNNNNNNNNNNNNNNNNNNNNNNNNNNNNNNNNNNNNNNNNNNNNNNNNNNNNNNNNNNNNNNNNNNNNNNNNNNNNNNNNNNNNNNNNNNNNNNNNNNNNNNNNNNNNNNNNNNNNNNNNNNNNNNNNNNNNNNNNNNNNNNNNNNNNNNNNNNNNNNNNNNNNNNNNNNNNNNNNNNNNNNNNNNNNNNNNNNNNNNNNNNNNNNNNNNNNNNNNNNNNNNNNNNNNNNNNNNNNNNNNNNNNNNNNNNNNNNNNNNNNNNNNNNNNNNNNNNNNNNNNNNNNNNNNNNNNNNNNNNNNNNNNNNNNNNNNNNNNNNNNNNNNNNN

>HM106720

GCGCCCATCACGGCYTATGCTCAACAGACGCGGGGCCTATTTGGCTGCATCATCACCAGCCTCACTGGCCGAGACAAGAACCAGGTCGAGGGGGAGGTTCAAGTNNNNNNNNNNNNNNNNNNNNNNNNNNNNNNNNNNNNNNNNNNNNNNGTGTGTTGGACTGTCTACCACGGCGCCGGCACAAAGACCCTCGCCGGCCAAAAGGGCCCAATCACCCAAATGTACACCAATGTAGACCAGGACCTCGTCGGCTGGCAAGCGCCTCCCGGGGCGCGATCCTTGACACCGTGCACCTGTGGCAGCTCGGACCTTTACTTGGTCACGAGGCATGCTGATGTCATTCCGGTGCGCCGGCGGGGCGACAGCAGAGGAAGCTTACTCTCCCCCAGGCCCGTCTCCTACTTGAAGGGTTCCTCGGGTGGTCCACTGCTCTGCCCCTCGGGGCATGCTGTGGGCATTTTCCGGGCTGCTGTGTGCACCCGGGGGGTTGCGAAGGCGGTGGACTTCGTGCCCGTTGAGTCTATGGAAACAACTATGCGGTCCCCGGTCTTCACGGACAACTCGTCCCCCCCGGCCGTACCGCAGACATTCCAGGTGGCCCATCTACACGCTCCCACTGGCAGCGGCAAGAGCACCAAGGTGCCGGCTGCGTATGCAGCCCAAGGGTACAAGGTACTTGTCCTGAACCCGTCTGTTGCCGCCACCCTAGGTTTCGGGGCGTATATGTCTAAGGCACATGGTGTCGACCCTAACATCAGAACTGGGATAAGGACCATCACCACGGGTGCCCCCATTACGTACTCCACCTATGGCAAGTTCCTTGCCGACGGTGGTTGCTCTGGGGGCGCCTATGACATCATAATATGTGATGAGTGCCATGCAACTGACTCGACTACCATCTTGGGCATCGGCACAGTCCTGGACCAAGCGGAGACGGCTGGAGCGCGACTCGTCGTGCTCGCCACCGCTACGCCTCCGGGATCGGTCACCGTGCCACATCCCAATATCGAGGAGGTGGCTCTGTCCAACACTGGAGAGATCCCCTTCTATGGTAAAGCCATNNNNNNNNNNNNNNNNNNNNNNNNNNNNNNNNNNNNNNNNNNNNNNNNNNNNNNNNNNNNNNNNNNNNNNNNNNNNNNNNNNNNNNNNNNNNNNNNNNNNNNNNNNNNNNNNNNNNNNNNNNNNNNNNNNNNNNNNNNNNNNNNNNNNNNNNNNNNNNNNNNNNNNNNNNNNNNNNNNNNNNNNNNNNNNNNNNNNNNNNNNNNNNNNNNNNNNNNNNNNNNNNNNNNNNNNNNNNNNNNNNNNNNNNNNNNNNNNNNNNNNNNNNNNNNNNNNNNNNNNNNNNNNNNNNNNNNNNNNNNNNNNNNNNNNNNNNNNNNNNNNNNNNNNNNNNNNNNNNNNNNNNNNNNNNNNNNNNNNNNNNNNNNNNNNNNNNNNNNNNNNNNNNNNNNNNNNNNNNNNNNNNNNNNNNNNNNNNNNNNNNNNNNNNNNNNNNNNNNNNNNNNNNNNNNNNNNNNNNNNNNNNNNNNNNNNNNNNNNNNNNNNNNNNNNNNNNNNNNNNNNNNNNNNNNNNNNNNNNNNNNNNNNNNNNNNNNNNNNNNNNNNNNNNNNNNNNNNNNNNNNNNNNNNNNNNNNNNNNNNNNNNNNNNNNNNNNNNNNNNNNNNNNNNNNNNNNNNNNNNNNNNNNNNNNNNNNNNNNNNNNNNNNNNNNNNNNNNNNNNNNNNNNNNNNNNNNNNNNNNNNNNNNNNNNNNNNNNNNNNNNNNNNNNNNNNNNNNNNNNNNNNNNNNNNNNNNNNNNNNNNNNNNNNNNNNNNNNNNNNNNNNNNNNNNNNNNNNNNNNNNNNNNNNNNNNNNNNNNNNNNNNNNNNNNNNNNNNNNNNNNNNNNNNNNNN

>HM106721

GCGCCCATCACGGCCTATGCCCAACAGACGCGGGGCCTACTCGGCTGCATYGTYACCAGCCTCACAGGCNNNNNNNNNNNNNNNNNNNNNNNNNNNNNNNNNNNNNNNNNNNNNNNNNNNNNNNNNNNNNNNNNNNNNNNNNNNNNNNNNNNNNNNNNNNNNNTCTACCACGGCGCCGGCACGAGGACCCTCGCCGGCCAAAAGGGCCCAATCACCCAAATGTACACCAATGTAGACCAGGACCTCGTCGGCTGGCAAGCGCCTCCCGGGGCGCGATCCTTGACACCGTGCACCTGTGGCAGCTCGGACCTTTACTTGGTCACGAGGCATGCTGATGTCATTCCGGTGCGCCGGCGGGGCGACAGCAGAGGAAGTCTACTCTCCCCCAGGCCCGTCTCCTACTTGAAGGGCTCTTCGGGTGGTCCACTGCTCTGCCCCTCGGGGCATGTTGTGGGCATCTTCCGGGCCGCCGTGTGCACCCGGGGGGTTGCAAAGGCGGTGGACTTTGTGCCCGTTGAGTCTATGGAAACAACTATGCGGTCCCCAGTCTTCACGGACAACTCGTCCCCCCCGGCCGTACCGCAGACATTCCAAGTGGCCCATCTACACGCTCCCACTGGCAGCGGCAAGAGCACTAAGGTGCCGGCTGCGTACGCAGCCCAAGGGTACAAGGTACTCGTCCTGAACCCGTCTGTTGCTGCCACCCTAGGTTTCGGGGCGTATATGTCTAAGGCACATGGTGTCGACCCTAACATCAGAACAGGGGTAAGGACCATCACCACGGGTGCCCCCATTACGTACTCCACCTATGGCAAGTTTCTTGCCGACGGTGGTTGCTCTGGGGGCGCCTATGACATCATAATATGTGATGAGTGCCACTCAACCGACTCGACTACCATCTTGGGCATCGGCACAGTCCTGGACCAAGCGGAGACGGCTGGAGCGCGACTCGTCGTGCTCGCCACCGCTACGCCTCCGGGATCGGTCACCGTGCCACATCCCAATATTGAGGAGGTGGCTCTGTCCAATATTGGAGAGATCCCCTTNNNNNNNNNNNNNNNNNNNNNNNNNNNNNNNNNNNNNNNNNNNNNNNNNNNNNNNNNNNNNNNNNNNNNNNNNNNNNNNNNNNNNNNNNNNNNNNNNNNNNNNNNNNNNNNNNNNNNNNNNNNNNNNNNNNNNNNNNNNNNNNNNNNNNNNNNNNNNNNNNNNNNNNNNNNNNNNNNNNNNNNNNNNNNNNNNNNNNNNNNNNNNNNNNNNNNNNNNNNNNNNNNNNNNNNNNNNNNNNNNNNNNNNNNNNNNNNNNNNNNNNNNNNNNNNNNNNNNNNNNNNNNNNNNNNNNNNNNNNNNNNNNNNNNNNNNNNNNNNNNNNNNNNNNNNNNNNNNNNNNNNNNNNNNNNNNNNNNNNNNNNNNNNNNNNNNNNNNNNNNNNNNNNNNNNNNNNNNNNNNNNNNNNNNNNNNNNNNNNNNNNNNNNNNNNNNNNNNNNNNNNNNNNNNNNNNNNNNNNNNNNNNNNNNNNNNNNNNNNNNNNNNNNNNNNNNNNNNNNNNNNNNNNNNNNNNNNNNNNNNNNNNNNNNNNNNNNNNNNNNNNNNNNNNNNNNNNNNNNNNNNNNNNNNNNNNNNNNNNNNNNNNNNNNNNNNNNNNNNNNNNNNNNNNNNNNNNNNNNNNNNNNNNNNNNNNNNNNNNNNNNNNNNNNNNNNNNNNNNNNNNNNNNNNNNNNNNNNNNNNNNNNNNNNNNNNNNNNNNNNNNNNNNNNNNNNNNNNNNNNNNNNNNNNNNNNNNNNNNNNNNNNNNNNNNNNNNNNNNNNNNNNNNNNNNNNNNNNNNNNNNNNNNNNNNNNNNNNNNNNNNNNNNNNNNNNNNNNNNNNNNNNNNNNNNNNNNNNNNNNNNNNNN

>HM106722

GCGCCCATCACGGCCTATGCCCAACAGACGCGGGGCCTACTTGGCTGCATCGTCACCAGCCTCACAGGCCGGGACAAGAACCAGGTCGAGGGGGAGGTTCAAGTGGTTTCCACCGCAACACAATCTTTCCTGGCGACCTGCGTCAACGGYGTGTGTTGGACCGTCTACCACGGCGCCGGCACAAAGACCCTCGCCGGCCAAAAAGGCCCAATTACCCAAATGTACACCAATGTAGACCAGGACCTCGTCGGCTGGCAAGCGCCTCCCGGGGCGCGATCCTYGACACCGTGTACCTGTGGCAGCTCGGACCTGTACTTGGTCACGAGGCATGCTGATGTCATCCCGGTGCGCCGGCGGGGCGACAGMWGGGGAAGYCTACTCTCCCCCAGGCCCGTCTCCTACTTGAAGGGCTCTTCGGGTGGCCCACTACTCTGCCCTTCGGGACATGCTGTAGGCATCTTCCGGGCTGCCGTGTGCACCCGGGGAGTTGCGAAGGCGGTGGACTTTGTGCCTGTTGAGTCTATGGAGACAACTATGCGGTCCCCGGTCTTCACAGACAACTCGTCCCCCCCGGCCGTACCGCAGACATTCCAAGTGGCCCATCTACATGCGCCTACTGGCAGCGGCAAGAGTACTAAAGTGCCGGCTGCGTATGCAGCTCAAGGGTACAAAGTACTCGTCCTGAACCCATCTGTTGCCGCCACCCTAAGTTTCGGGGCGTATATGTCTAAGGCACATGGTGTCGACCCTAACATCAGAACTGGGGTAAGGACCATCACCACGGGTGCCCCCATCACGTACTCCACYTATGGCAAGTTCCTTGCCGACGGTGGYTGYTCTGGGGGCGCCTATGACATCATAATWTGTGATGAGTGCCACTCAACTGACTCGACTACCATCTTGGGCATCGGCACAGTCCTGGACCAAGCGGAGACGGCTGGAGCGCGACTCGTCGTGCTCGCCACCGCTACGCCTCCGGGATCGGTCACCGTGCCACATCCCAATATCGAGGAGGTGGCTCTGTCCAATGATGGAGAGATCCCCTTCTATGGTAAAGCCATCCCCATCGAGNNNNNNNNNNNNNNNNNNNNNNNNNNNNNNNNNNNNNNNNNNNNNNNNNNNNNNNNNNNNNNNNNNNNNNNNNNNNNNNNNNNNNNNNNNNNNNNNNNNNNNNNNNNNNNNNNNNNNNNNNNNNNNNNNNNNNNNNNNNNNNNNNNNNNNNNNNNNNNNNNNNNNNNNNNNNNNNNNNNNNNNNNNNNNNNNNNNNNNNNNNNNNNNNNNNNNNNNNNNNNNNNNNNNNNNNNNNNNNNNNNNNNNNNNNNNNNNNNNNNNNNNNNNNNNNNNNNNNNNNNNNNNNNNNNNNNNNNNNNNNNNNNNNNNNNNNNNNNNNNNNNNNNNNNNNNNNNNNNNNNNNNNNNNNNNNNNNNNNNNNNNNNNNNNNNNNNNNNNNNNNNNNNNNNNNNNNNNNNNNNNNNNNNNNNNNNNNNNNNNNNNNNNNNNNNNNNNNNNNNNNNNNNNNNNNNNNNNNNNNNNNNNNNNNNNNNNNNNNNNNNNNNNNNNNNNNNNNNNNNNNNNNNNNNNNNNNNNNNNNNNNNNNNNNNNNNNNNNNNNNNNNNNNNNNNNNNNNNNNNNNNNNNNNNNNNNNNNNNNNNNNNNNNNNNNNNNNNNNNNNNNNNNNNNNNNNNNNNNNNNNNNNNNNNNNNNNNNNNNNNNNNNNNNNNNNNNNNNNNNNNNNNNNNNNNNNNNNNNNNNNNNNNNNNNNNNNNNNNNNNNNNNNNNNNNNNNNNNNNNNNNNNNNNNNNNNNNNNNNNNNNNNNNNNNNNNNNNNNNNNNNNNNNNNNNNNNNNNNNNNNNNNNNNNNNNNNNNNNNNNNNNNNNNNNNNNNNNNNNNNN

>HM106723

GCGCCCATCACGGCCTATGCCCAACAGACGCGGGGCTTACTTGGCTGCATCGTCACCAGCCTCACAGGCCGGGACAAGAATCAGGTCGAGGGGGAGGTTCAAGTGGTTTNNNNNNNNNNNNNNNNNNNNNNNNNNNNNNNNNNNNNNNNNNNNNNNNNNNNNNNNNNNNNNNNNNNNNNNNNNNNNNNNNNNNNNNNNNNNNNNNNNNNNNNNACCCAAATGTACACCAATGTAGACCAGGACCTCGTCGGCTGGCAAGCGCCTCCCGGGGCGCGATCCTTGACACCGTGCACCTGTGGCAGCTCGGACCTTTACTTGGTCACGAGGCATGCYGATGTCATTCCGGTGCGCCGGCGGGGCGACAGCAGAGGAAGCCTACTCTCCCCCAGGCCCGTCTCCTACTTGAAGGGCTCTTCGGGTGGTCCACTGCTTTGTCCCTCGGGGCATGCTGTGGGCATCTTCCGGGCTGCTGTGTGCACCCGGGGGGTTGCGAAGGCGGTRGACTTTGTGCCCGTTGAGTCTATGGAAACAACTATGCGGTCCCCGGTCTTCACGGACAACTCGTCCCCTCCGGCCGTACCGCAGACATTCCAAGTGGCCCATCTGCACGCTCCCACTGGCAGCGGCAARAGCACTAAGGTGCCGGCTGCGTATGCAGCCCAAGGGTACAAGGTACTCGTCTTGAACCCGTCTGTTGCCGCCACCCTAGGTTTCGGGGCGTATATGTCTAAGGCACATGGTGTCGACCCYAACATCAGAACTGGGGTAAGGACCATCACCACAGGTGCCCCCATTACGTACTCCACCTATGGCAAGTTTCTTGCCGACGGTGGCTGCTCTGGGGGCGCCTATGACATCATAATATGTGATGAGTGCCACTCAACTGACTCGACTACCATCTTGGGCATCGGCACAGTCCTGGACCAAGCGGAGACGGCTGGAGCRCGACTCGTCGTACTCGCCACCGCTACGCCTCCAGGATCGGTCACCGTGCCACATCCCAATATCGAGGAGGTGGCTCTGTCCAACRCTGGRGAGRTCCCCTTCTATGGTAAGGCCATCCCCATTGAGACCATCAAGGGGGGGAGGCACCTCATNNNNNNNNNNNNNNNNNNNNNNNNNNNNNNNNNNNNNNNNNNNNNNNNNNNNNNNNNNNNNNNNNNNNNNNNNNNNNNNNNNNNNNNNNNNNNNNNNNNNNNNNNNNNNNNNNNNNNNNNNNNNNNNNNNNNNNNNNNNNNNNNNNNNNNNNNNNNNNNNNNNNNNNNNNNNNNNNNNNNNNNNNNNNNNNNNNNNNNNNNNNNNNNNNNNNNNNNNNNNNNNNNNNNNNNNNNNNNNNNNNNNNNNNNNNNNNNNNNNNNNNNNNNNNNNNNNNNNNNNNNNNNNNNNNNNNNNNNNNNNNNNNNNNNNNNNNNNNNNNNNNNNNNNNNNNNNNNNNNNNNNNNNNNNNNNNNNNNNNNNNNNNNNNNNNNNNNNNNNNNNNNNNNNNNNNNNNNNNNNNNNNNNNNNNNNNNNNNNNNNNNNNNNNNNNNNNNNNNNNNNNNNNNNNNNNNNNNNNNNNNNNNNNNNNNNNNNNNNNNNNNNNNNNNNNNNNNNNNNNNNNNNNNNNNNNNNNNNNNNNNNNNNNNNNNNNNNNNNNNNNNNNNNNNNNNNNNNNNNNNNNNNNNNNNNNNNNNNNNNNNNNNNNNNNNNNNNNNNNNNNNNNNNNNCAGGGCTCAGGCTCCACCCCCATCGTGGGACCAGATGTGGAAATGTCTCATACGGCTAAAGCCCACGCTGCACGGGCCAACACMCCTGCTGTATAGGCTAGGAGCCGTCCAAAATGAGGTCACTCTCACACACCCCATAACCAAATACATCATGACATGCATGTCGGCTGACCTGGAGGTCGTCACG

>HM106724

GCGCCCATCACGGCCTATGCCCAACAGACGCGGGGCTTACTTGGCTGCATCGTCACCAGCCTCACAGGCCGGGACAAGAACCAGGTCGAGGGGGAGGTTCAAGTGGTTTCTACCGCAACACAATCTTTCCTGGCGACCTGCGTCAACGGCGTGTGTTGGACTGTCTACCACGGCGCCGGCACWAAGACCCTYGCCGGCCCAAARGGCCCAATCACCCAAATGTACACCAATGTAGACCAGGACCTCGTCGGCTGGCAAGCGCCTCCCGGGGCGCGATCCTTGACACCGTGCACCTGTGGCAGCTCGGACCWTTACTTGGTCACGAGRCATGCTGATGTYATTCCGGTGCGCCGGCGGGGCGACAGCMGRGGAAGYCTACTCTCCCCCAGGCCCRTCTCCTACTTGAAGGGCTCTTCGGGTGGTCCRCTGCTCTGCCCCTCGGGGCATGCTGTGGGCATCTTCCGGGCTGCTGTGTGCACCCGGGGGGTTGCGAAGGCRGTGGACTTYGTGCCCGTTGAGTCTATGGAAACAACTATGCGGTCCCCGGTCTTCACGGACAACTCGTCCCCCCCGGCCGTACCGCAGACATTCCAAGTGGCCCATCTACACGCTCCYACTGGYAGCGGCAAGAGCACTAAGGTGCCGGCTGCGTATGCAGCCCAAGGGTACAAGGTACTCGTCCTGAACCCGTCTGTYGCCGCCACCCTRGGTTTCGGGGCGTATATGTCTAAGGCACATGGTGTCGACCCTAACATCAGAACTGGGGTAAGGACCATCACCACGGGTGCYCCCATTACGTACTCCACYTATGGCAAGTTCCTTGCCGACGGTGGTTGYTCTGGGGGCGCCTAYGACATCATAATWTGTGATGAGTGCCAYTCAACTGAYTCRACTACCATCTTGGGCATCGGCACAGTCCTGGACCAAGCGGAGACGGCTGGAGCGCGACTCGTCGTACTCGCCACCGCTACGCCTCCGGGATCGGTCACCGTGCCACAYCCCAATATCGAGGAGGTGGCTCTGTCCAACATTGGRGAGATCCCCTTCTACGGNNNNNNNNNNNNNNNNNNNNNNNNNNAGGGGGGGAGGCACCTCATATTYTGCCATTCCAGGAAAAAATGTGATGAGCTCGCCGCGAAGCTGTCGGGTCTCGGGCTTAACGCTGTAGCGTATTACCGGGGCCTYGATGTGTCCGTCATACCGACCAGCGGAGACGTCGTTGTCGTGGCAACAGACGCTCTAATGACGGGSTWCACTGGCGACTTTGACTCRGTRATCGACTGTAATACATGTGTCACCCAGACRGTCGACTTCAGCTTRGACCCTACCTTCACCATTGARACRACGACCGTGCCYCAAGACGCGGTGTCGCGCTCGCAGCGKCGAGGCAGGACTGGTAGGGGCAGGACGGGCATCTACAGGTTTGTGACTCCAGGAGARCGGCCYTCGGGCATGTTCGATTCCTCGGTCCTRTGTGAGTGCTATGACGCGGGYTGTGCTTGGTACGAGCTCACGCCCGCCGAGACCTCAGTTAGGTTGCGGGCTTACCTGAATACACCAGGGTTGCCCGTCTGYCAGGACCATCTGGAGTTCTGGGAGGGCGTCTTCACAGGCCTCACCCACATAGATGCCCACTTCCTGTCYCAGACTAARCAGGCAGGAGACAACTTCCCCTACTTGGTAGCATACCAGGCTACGGTGTGCGCCAGGGCTCAGGCTCCRCCCCCATCRTGGGACCAAATGTGGAAATGTCTCATACGGCTAAAGCCCACGCTGCACGGGCCAACACCCCTGCTGTAYAGGCTAGGAGCCGTCCAAAATGAGGTCACCCTCACACACCCCATAACCAAATTCATCATGGCATGCATGTCGGCTGACCTGGAGGTCGCCACG

>HM106725

GCGCCCATCACGGCCTAYGCCCAACAGACGCGGGGCCTACTTGGCTGCATCGTCACCAGCCTCACAGGCCGGGACAAGAACCAGGTYGAGGGGGAGGTTCAAGTGGTTTCCACCGCAACACAATCCTTCCTGGCGACCTGCGTCAACGGCGTGTGTTGGACTGTCTACCACGGCGCTGGCACAAAGACCCTCGCCGGCCAGAAGGGCCCAATCACCCAAATGTACACCAATGTGGACCAGGACCTCGTCGGCTGGCAAGCGCCTCCCGGGGCGCGATCCTTGACACCGTGCACCTGTGGCAGCTCGGACCTTTACTTGGTTACGAGGCATGCTGATGTCATTCCGGTGSGCCGGCGGGGCGACAGMAGAGGAAGYCTACTCTCCCCCAGGCCCGTCTCCTACTTGAAGGGCTCTTCGGGTGGTCCACTGCTCTGCCCCTCGGGACATGCGGTGGGCATCTTCCGGGCTGCTGTGTGCACCCGGGGGGTTGCGAAGGCAGTGGACTTTGTGCCCGTTGAGTCTATGGAAACAACTATGCGGTCCCCGGTCTTCACGGACAACTCGTCCCCTCCGGCCGTACCGCAGACATTCCAAGTGGCCCATCTACACGCTCCCACTGGCAGCGGCAAGAGCACTAAGGTGCCGGCTGCGTATGCAGCCCAAGGGTACAAGGTACTCGTCCTGAACCCGTCTGTTGCCGCCACCCTAGGTTTCGGGGCGTATATGTCTAAGGCACATGGTGTCGACCCTAACATCAGAACTGGGGTAAGGACCATCACCACGGGTGCCCCCATTACGTACTCCACCTATGGCAAGTTCCTTGCCGACGGTGGTTGCTCTGGGGGCGCCTATGACATCATATTATGTGATGAGTGCCACTCAACTGACTCGACTACCATCTTGGGCATCGGCACAGTCCTGGACCNNNNNNNNNNNNNNNNNNNNNNNNNNNNNNNNNNNNNNNNNNNNNNNNNNNNNNNNNNNNNNNNNNNNNNNNNNNNNNNNNNNNNNNNNNNNNNNNNNNNNNNNNNNNNNNNNNNNNNNNNNNNNNNNNNNNNNNNNNNNNNNNNNNNNNNNNNNNNNNNNNNNNNNNNNNNNNNNNNNNNNNNNNNNNNNNNNNNNNNNNNNNNNNNNNNNNNNNNNNNNNNNNNNNNNNNNNNNNNNNNNNNNNNNNNNNNNNNNNNNNNNNNNNNNNNNNNNNNNNNNNNNNNNNNNNNNNNNNNNNNNNNNNNNNNNNNNNNNNNNNNNNNNNNNNNNNNNNNNNNNNNNNNNNNNNNNNNNNNNNNNNNNNNNNNNNNNNNNNNNNNNNNNNNNNNNNNNNNNNNNNNNNNNNNNNNNNNNNNNNNNNNNNNNNNNNNNNNNNNNNNNNNNNNNNNNNNNNNGCGAGGCAGGACTGGTAGGGGCAGGACAGGCATCTACAGGTTTGTGACTCCAGGGGAACGCCCCTCGGGCATGTTCGATTCTTCGGTCCTGTGTGAGTGCTATGACGCGGGCTGTGCTTGGTACGAGCTCACACCCGCCGAGACCTCAGTTAGACTGCGGGCTTACCTGAATACACCAGGGTTGCCCGTCTGCCAGGACCACCTGGAGTTCTGGGAGGGCGTCTTCACAGGCCTCACCCACATAGATGCCCACTTCCTGTCCCAGACTAAGCAGGCAGGAGACAACTTCCCTTACTTGGTAGCATACCAGGCTACGGTGTGCGCCAGGGCTCAGGCTCCACCCCCATCGTGGGACCAAATGTGGAAATGTCTCATACGGCTAAAGCCCACGCTGCACGGGCCAACACCCCTGTTGTATAGGCTAGGAGCCGTCCAAAATGAGGTCACCCTCACACACCCCATAACCAAATTCATCATGGCATGCATGTCGGCTGACCTGGAGGTCGCCACG

>HM106726

GCGCCCATCACGGCCTATGCCCAACAGACGCGGGGCCTACTTGGCTGCATCGTCACCAGCCTCACAGGCCGGGACAAGAACCAGGTCGAGGGGGAGGTTCAAGTGGTTTCCACCGCAACACNNNNNNNNNNNNNNNNNNNNNNNNNNNNNGTGTGTTGGACTGTCTACCACGGCGCCGGCACAAAGACCCTCGCCGGCCAAAAGGGGCCAATCACCCAGATGTACACCAATGTAGACCAGGACCTCGTCGGCTGGCAAGCGCCTCCCGGGGCGCGATCCTTGACACCGTGCACCTGTGGCAGCTCGGACCTTTATTTGGTCACGAGGCATGCTGATGTCATTCCAGTGSGCCGGCGGGGCGACAGMAGAGGAAGYCTACTCTCCCCCAGGCCTGTCTCCTACTTGAAGGGCTCTTCGGGTGGTCCACTGCTCTGCCCCTCGGGGCATGCTGTGGGCATCTTCCGGGCTGCTGTGTGCACCCGGGGGGTTGCGAAGGCGGTGGACTTTGTGCCCGTTGAGTCTATGGAAACAACTATGCGGTCCCCGGTCTTCACGGACAACTCGTCCCCCCCGGCCGTACCGCAGACATTCCAAGTGGCCCATCTACACGCTCCCACTGGCAGCGGCAAGAGCACTAAGGTGCCGGCTGCGTATGCAGCCCAAGGGTACAAGGTACTTGTCCTGAACCCTTCTGTTGCCGCCACCCTAGGTTTCGGGGCGTATATGTCTAAGGCACATGGTGTCGACCCCAACATCAGAACTGGGGTAAGGACCATCACCACGGGTGCCCCCATTACGTACTCCACCTATGGCAAGTTTCTAGCCGACGGTGGTTGCTCTGGGGGCGCCTATGACATCATAATATGTGATGAGTGCCACTCAACTGACTCGACTACCATCTTGGGCATCGGCACGGTCCTGGACCAAGCGGAGACGGCTGGAGCGCGACTCGTYGTGCTCGCCACCGCTACGCCTCCGGGATCGGTCACCGTGCCACATCCCAATATCGAGGAGGTGGCTCTGTCCAACATTGGAGAGATCCCCTTCTATGGTAAAGCNNNNNNNNNNNNNNNNNNNNNNNNNNNNNNNNNNNNNNNNNNNNNNNNNNNNNNNNNNNNNNNNNNNNNNNNNNNNNNNNNNNNNNNNNNNNNNNNNNNNNNNNNNNNNNNNNNNNNNNNNNNNNNNNNNNNNNNNNNNNNNNNNNNNNNNNNNNNNNNNNNNNNNNNNNNNNNNNNNNNNNNNNNNNNNNNNNNNNNNNNNNNNNNNNNNNNNNNNNNNNNNNNNNNNNNNNNNNNNNNNNNNNNNNNNNNNNNNNNNNNNNNNNNNNNNNNNNNNNNNNNNNNNNNNNNNNNNNNNNNNNNNNNNNNNNNNNNNNNNNNNNNNNNNNNNNNNNNNNNNNNNNNNNNNNNNNNNNNNNNNNNNNNNNNNNNNNNNNNNNNNNNNNNNNNNNNNNNNNNNNNNNNNNNNNNNNNNNNNNNNNNNNNNNNNNNNNNNNNNNNNNNNNNNNNNNNNNNNNNNNNNNNNNNNNNNNNNNNNNNNNNNNNNNNNNNNNNNNNNNNNNNNNNNNNNNNNNNNNNNNNNNNNNNNNNNNNNNNNNNNNNNNNNNNNNNNNNNNNNNNNNNNNNNNNNNNNNNNNNNNNNNNNNNNNNNNNNNNNNNNNNNNNNNNNNNNNNNNNNNNNNNNNNNNNNNNNNNNNNNNNNNNNNNNNNNNNNNNNNNNNNNNNNNNNNNNNNNNNNNNNNNNNNNNNNNNNNNNNNNNNNNNNNNNNNNNNNNNNNNNNNNNNNNNNNNNNNNNNNNNNNNNNNNNNNNNNNNNNNNNNNNNNNNNNNNNNNNNNNNNNNNNNNNNNNNNNNNNNNNNNNNNNNNNNNNNNNNNNNNNNNNNNNNNNNNNNN

>HM106727

GCGCCAATCACGGCCTATGCCCAACAGACGCGGGGCCTACTTGGCTGCATCGTCACCAGCCTCACAGGCCGGGACAAGAACCAGGTCGAGGGGGAGGTCCAAGTGGTTTCCACCGCAACACAATCTTTCCTGGCGACCTGCGTCAACGGCGTGTGTTGGACTGTCTATCAYGGCGCCGGCACAAAGACCCTTGCCGGCCAAAAGGGCCCARTCACCCAAATGTACACCAATGTAGACCAGGACCTCGTCGGCTGGCAAGCGCCTCCCGGGGCGCGATCCTTGACACCGTGCACCTGTGGCAGCTCGGACCWWTACTTGGTCACGMGGCATGCTGATGTYATTCCGGTGCGCCGGCGGGGCGACAGCAGRGGGAGYCTACTCTCCCCCAGGCCCGTCTCCTACTTGAAAGGCTCTTCGGGTGGTCCGCTGCTCTGCCCTTCGGGGCAYGCTGTAGGCATCTTCCGGGCTGCTGTGTGCACCCGGGGGGTTGCGAAGGCGGTGGACTTTGTGCCCGTYGAGTCTATGGAAACAACTATGCGGTCCCCGGTCTTCACGGACAACTCGTCCCCCCCGGCCGTACCGCAGACATTCCAAGTGGCCCATCTACACGCTCCCACTGGCAGCGGCAAGAGCACCAAGGTGCCGGCTGCGTATGCAGCCCARGGGTAYAAGGTACTCGTCCTGAACCCCTCTGTTGCCGCCACCCTAGGTTTCGGGGCGTATATGTCTAAGGCACATGGTGTCGACCCTAACATCAGAACTGGGGTTAGGACCATCACCACGGGTGCCCCCATTACGTACTCCACYTATGGCAAGTTCCTTGCCGACGGTGGTTGYTCTGGGGGCGCCTATGACATCATAATWTGTGATGAGTGCCACTCAACTGACTCGACTACCATCTTGGGCATCGGCACAGTCCTGGACCAAGCGGAGACGGCTGGAGCGCGACTCGTCGTACTCGCCACCGCTACGCCTCCGGGATCGGTCACCGTGCCWCATCCCAATATCGAGGAGGTGGCTCTGTCCAATACTGGAGAGATCCCCTTCTATGGTAAAGCCATCCCCATCGAGACNNNNNNNNNNNNNNNNNNNNNNNNNNNNNNNNNNNNNNNNNNNNNNNNNNNNNNNNNNNNNNNNNNNNNNNNNNNNNNNNNNNNNNNNNNNNNAGCGTACTACCGGGGTCTTGATGTGTCCGTCATACCGACCAGCGGKGACGTCGTTGTCGTGGCAACAGACGCTCTRATGACGGGTTTCACTGGCGACTTTGACTCGGTGATCGACTGTAATACRTGTGTCACCCARACAGTCGACTTCAGCTTGGACCCTACCTTCACCATTGAGACGACGACSRTGCCCCAAGACGCGGTGTCGCGCTCGCAGCGGCGAGGCAGGACTGGTAGGGGCAGGACRGGCATCTACAGGTTTGTGACTCCGGGAGRACGACCCTCGGGCATGTTCGACTCCTCGGTCCTGTGTGAGTGCTATGACGCGGGCTGTGCKTGGTACGAGCTCACGCCCGCCGAGACCTCAGTTAGGTTGCGRGCTTACCTAAATACACCAGGGTTGCCCGTCTGCCAGGACCATCTGGAGTTCTGGGAGGGCGTCTTCACAGGCCTCACCCACATAGATGCCCACTTCCTGTCCCAGACTAAGCAGGCAGGAGACAACTTCCCCTACTTGGTAGCATACCAGGCTACGGTGTGCGCCAGGGCTCAGGCTCCACCCCCATCGTGGGACCAAATGTGGAAATGTCTCATACGGCTAAAGCCTACGCTGCACGGGCCAACACCCTTGCTGTATAGGCTGGGAACCGTCCAAAATGAGGTCACCCTCACACACCCCATAACCAAATTCATCATGGCATGCATGTCGGCTGACCTGGAGGTCGCCACG

>HM106728

GCGCCCATCACGGCCTATGCCCAACAGACGCGGGGCCTACTTGGCTGCATCGTCACCAGCCTCACAGGCCGAGACAAGAACCAGGTCGAGGGGGAGGTTCAAGTGGTYTCTACCGCAACACAATCTTTCCTGGCGACCTGCGTCAACGGCGTGTGTTGGACTGTCTACCACGGCGCCGGCACAAAGACCCTCGCCGGCCCAAAGGGCCCAATCACCCAAATGTACACCAATGTAGACCAGGACCTCGTCGGCTGGCAAGCGCCTCCCGGGGCGCGATCCCTGACACCGTGCACCTGTGGCAGCTCGGACCTTTACTTGGTCACGAGGCATGCTGATGTCATTCCGGTGSGCCGGCGGGGCGACAGMAGAGGAAGYCTACTTTCCCCCAGGCCCGTCTCCTACTTGAAGGGCTCTTCGGGTGGTCCACTGCTCTGCCCCTCGGGGCATGCTGTGGGCATCTTCCGGGCTGCTGTGTGCACCCGGGGGGTTGCGAAGGCAGTGGACTTTGTGCCCGTTGAGTCTATGGAAACAACCATGCGGTCCCCGGTCTTCACGGACAACTCGTCCCCTCCGGCCGTACCGCAGACATTCCAAGTGGCCCATCTACACGCTCCCACTGGCAGCGGCAAGAGCACTAAGGTGCCGGCTGCGTATGCAGCCCAAGGGTACAAGGTACTCGTCCTGAACCCGTCTGTTGCCGCCACCCTAGGCTTCGGGGCGTACATGTCTAAGGCACATGGTGTCGACCCTAACATCAGAACTGGGGTAAGGACCATCACCACCAGTGCCCCCATTACGTACTCCACCTATGGCAAGTTTCTTGCCGACGGTGGTTGCTCTGGGGGCGCCTACGACATCATAATWTGTGATGAGTGCCACTCAACTGACTCGACTACCATCTTGGGCATCGGCACAGTCCTGGACCAAGCGGAGACGGCTGGAGCGCGACTCGTYGTACTCGCCACCGCTACGCCTCCGGGATCGGTCACCGTGCCACATCCCAATATTGAGGAGGTGGCTCTGTCCAACAYTGGAGAGATCCCCTTCTATGGTAAAGCCATCCCCATCGAGACCATNNNNNNNNNNNNNNNNNNNNNNNNNNNNNNNNNNNNNNNNNNNNNNNNNNNNNNNNNNNNNNNNNNNNNNNNNNNNNNNNNNNNNNNNNNNNNNNNNNNNNNNNNNNNNNNNNNNNNNNNNNNNNNNNNNNNNNNNNNNNNNNNNNNNNNNNNNNNNNNNNNNNNNNNNNNNNNNNNNNNNNNNNNNNNNNNNNNNNNNNNNNNNNNNNNNNNNNNNNNNNNNNNNNNNNNNNNNNNNNNNNNNNNNNNNNNNNNNNNNNNNNNNNNNNNNNNNNNNNNNNNNNNNNNNNNNNNNNNNNNNNNNNNNNNNNNNNNNNNNNNNNNNNNNNNNNNNNNNNNNNNNNNNNNNNNNNNNNNNNNNNNNNNNNNNNNNNNNNNNNNNNNNNNNNNNNNNNNNNNNNNNNNNNNNNNNNNNNNNNNNNNNNNNNNNNNNNNNNNNNNNNNNNNNNNNNNNNNNNNNNNNNNNNNNNNNNNNNNNNNNNNNNNNNNNNNNNNNNNNNNNNNNNNNNNNNNNNNNNNNNNNNNNNNNNNNNNNNNNNNNNNNNNNNNNNNNNNNNNNNNNNNNNNNNNNNNNNNNNNNNNNNNNNNNNNCTTCCCCTACTTAGTAGCATACCAAGCTACGGTGTGCGCCAGGGCTCAGGCTCCACCCCCATCGTGGGACCAGATGTGGAAATGTCTCATACGGCTAAAGCCCACGCTGCACGGGCCAACACCCCTGCTGTATAGGCTAGGAGCCGTCCAAAATGAGGTCATCCTCACACACCCCATAACCAAATTCATCATGGCATGCATGTCGGCTGACCTGGAGGTCGCCACG

>HM106729

GCKCCCATCACGGCCTACGCCCAACAGACGCGGGGCCTACTCGGCTGCATCGTCACCAGCCTCACAGGTCGGGACAAGAACCAGGTCGAGGGGGAGGTTCAAGTGGTTTCCACCGCAACACAGTCTTTCCTGGCGGCCTGCATCAACGGCGTGTGTTGGACTGTCTACCACGGCGCCGGCACAAAGACYCTCGCCGGCCAAAARGGCCCAATCACCCAAATGTACACCAATGTAGACCAGGACCTCGTCGGCTGGCAAGCGCCTCCCGGGGCGCGRTCCTTGACACCGTGCACYTGTGGCAGCTCGGACCTTTAYTTGGTCACGAGGCATGCTGATGTCATTCCGGTGCGCCGGCGGGGCGACAGCAGAGCAAGCCTACTCTCCCCCAGGCCCGTCTCCTACTTGAAGGGCTCTTCGGGTGGYCCMCTGCTTTGCCCCTCGGGGCATGCTGTGGGCATCTTCCGGGCCGCTGTGTGCACCCGGGGGGTTGCGAAGGCGGTGGACTTTGTGCCCGTTGAGTCCATGGAAACAACTATGCGGTCCCCGGTCTTCACGGACAACTCGTCCCCCCCGGCCGTACCGCAGACATTCCAAGTGGCCCATCTACACGCTCCCACYGGCAGCGGCAARAGCACTAAGGTGCCGGCTGCGTATGCAGCCCAAGGGTACAAGGTACTCGTCCTGAACCCGTCTGTTGCCGCCACCTTAGGTTTCGGGGCGTATATGTCTAAGGCACATGGTGTCGACCCCAACATYAGAACTGGGGTAAGGACCATCACCACGGGTGCCCCCATTACRTACTCCACCTATGGCAAGTTYCTTGCCGACGGTGGTTGCTCTGGGGGCGCCTATGACATCATAATGTGTGATGAATGCCACTCAACTGACTCGACTACCATCYTGGGCATCGGCACAGTCCTGGACCAAGCGGAGACGGCTGGAGCGCGACTYGTCGTACTCGCCACCGCTACGCCTCCGGGATCGGTCACCGTGCCACATCCCAATATCGAGGAGGTGGCTCTGTCCAAYAYTGGAGAGATCCCCTTCTAYGGTAAAGCCATCCCCATCGARACCATCAAGGGGGGGAGGCACCTCATATTTTGCCATTCYAGGAARAAATGTGATGAGCTCGCCGCAAAGCTGTCGGGCCTCGGGCTTAACGCTGTAGCGTATTACCGGGGCCTYGATGTGTCCGTCATACCGRCCAGCGGAGACGTCGTTGTCGTGGCAACAGACGCTCTAATGACGGGTTTCACTGGCGAYTTTGACTCAGTGATCGACTGTAATACATGTGTCACCCAGACAGTCGACTTCAGCTTGGACCCTACCTTCACCATTGAGACGACGACCGTGCCCCAAGACGCRGTGTCGCGCTCGCAGCGGCGAGGCAGRACTGGTAGGGGYAGGACRGGCATCTACAGGTATGTGACTCCAGGAGAACGGCCCTCGGGCATGTTCGATTCCTCGGTCCTRTGTGAGTGCTATGACGCGGGCTGTGCTTGGTACGAGCTCACGCCCGCCGAGACCACAGTTAGGTTGCGGGCTTACCTGAATACACCAGGGTTGCCCGTCTGCCRGGACCATCTGGAATTCTGGGAGGGCGTCTTCACAGGCCTCACCCACATAGATGCCCACTTCCTGTCCCAGACTAAGCAGGCAGGAGACAACTTCCCCTACTTGGTAGCATACCAGGCTACGGTGTGCGCCAGGGCTCAGGCCCCACCCCCATCGTGGGACCAAATGTGGAAATGTCTCATACGGCTAAAGCCCACGCTGCACGGGCCAACACCCCTGCTGTATAGGCTAGGAGCCGTCCAAAATGAGGTTGTCCTCACACACCCCATAACCAAATACATCATGACATGCATGTCGGCTGACCTGGAGGTCACCACG

>HM106730

GCGCCCATCACGGCCTACGCCCAACAGACGCGGGGCCTACTTGGCTGCATCGTTACCAGCCTCACAGGCCGGGACAAGAACCAGGTCGAGGGGGAGGTTCAAGTGGTTTCCACCGCGACACAGTCTTTCCTGGCGACCTGCATCAACGGTGTGTGTTGGACTGTCTACCATGGCGCCGGCACAAAGACCCTCGCCGGCCCAAAGGGCCCAATCACCCAAATGTACACCAATGTAGACCAGGACCTCGTCGGCTGGCAAGCGCCTCCTGGGGCGCGWTCYTTGACACCGTGCACCTGCGGCAGTTCGGACCTYTACTTGGTCACGAGGCATGCTGATGTTATYCCGGTGCGCCGGCGGGGCGACAGCAGAGGAAGCCTACTCTCCCCCAGGCCTGTCTCTTACTTGAAGGGCTCTTCGGGCGGTCCACTGCTCTGCCCCTCGGGGCATGCTGTGGGTATCTTCCGGGCTGCTGTGTGCACCCGGGGGGTTGCGAAGGCGGTGGACTTTGTACCNNNNNNNNNNNNNNNNNNNNNNNNNNNNNNNNNNNNNNNNACGGACAACTCGTCCCCYCCGGCCGTACCGCAGACATTCCAGGTGGCCCATCTACACGCHCCTACTGGCAGCGGCAAGAGCACTAAGGTGCCGGCTGCGTATGCAGCCCAAGGGTATAAGGTACTCGTCCTGAACCCGTCTGTCGCCGCCACCCTAGGYTTCGGGGCGTATATGTCTAAGGCACATGGTGTCGACCCTGGCATCAGAACTGGGGTAAGGACCATCACCACGGGTGCCCCCATTACGTACTCCACCTATGGCAAGTTTCTTGCCGACGGTGGTTGCTCTGGGGGCGCCTATGACATCATAATATGTGATGAGTGCCACTCAACTGACTCGACTACCATCCTGGGCATCGGCACGGTCCTGGACCAAGCGGAGACGGCTGGAGCGCGACTCGTCGTACTCGCCACCGCTACGCCYCCGGGRTCGGTCACCGTGCCACATCCCAATATCGAGGAGGTGGCCCTGTCCAACGTTGGAGAGATCCCCTTCTATGGTAAAGCCATCCCCATTGAGRCCATCAAGGGGGGGAGGCACCTCATTTTTTGCCATTCCAGGAAAAAATGTGATGAGCTCGCCGCAAAGCTGTCGAGTCTCGGGCTYAACGCTGTAGCGTATTACCGGGGTCTTGATGTGTCCGTCATACCGACCAGCGGAGACGTCGTTGTCGTGGCAACAGACGCTCTGATGACGGGTTTCACTGGCGACTTTGACTCRGTGATCGACTGTAATACATGTGTCACCCAGACAGTCGACTTCAGCTTGGACCCTACCTTCACCATTGAGACGACGACCGTGCCCCAAGAYGCGGTGTCGCGCTCGCAGCGGCGAGGCAGGACTGGTAGGGGCAGGGCAGGCATCTATAGGTTTGTGACTCCAGGAGAACGGCCCTCGGGCATGTTCGATTCCTCGGTCCTGTGTGAGTGCTATGACGCGGGCTGTGCTTGGTATGAGCNNNNNNNNNNNNNNNNNNNNNNNNNNNNNNNNNNNNNNNNNNNNNNNNNNNNNNNNNNNNNNNNNNNNNNNNNNNNNNNNNNNNNNNNNNNNNNNNNNNNNNNNNNNNNNNNNNNNNNNNNNNNNNNNNNNNNNNNNNNNNNNNNNNNNNNNNNNNNNNNNNNNNNNNNNNNNNNNNNNNNNNNNNNNNNNNNNNNNNNNNNNNNNNNNNNNNNNNNNNNNNNNNNNNNNNNNNNNNNNNNNNNNNNNNNNNNNNNNNNNNNNNNNNNNNNNNNNNNNNNNNNNNNNNNNNNNNNNNNNNNNNNNNNNNNNNNNNNNNNNNNNNNNNNNNNNNNNNNNNNNNNNNNNNNNNNNNNNNNNNNNNNNNNNNNNNNNNNNNNNNNN

>HM106731

GCGCCCATCACAGCCTATGCCCAACAGACGCGGGGCCTACTTGGCTGCATCGTCACCAGCCTCACAGGCCGGGACAAGAACCAGGTCGAGGGGGAGGTTCAAGTGGTCTCCACNNNNNNNNNNNNNNNNNNNNNNNNNTGCGTCAACGGCGTGTGTTGGACTGTCTACCACGGCGCCGGCACAAAAACCCTCGCCGGCCAAAAGGGGCCAATCACCCAAATGTATACCAATGTAGACCAGGACCTCGTCGGCTGGCAGGCGCCCCCCGGGGCGCGATCCTTGACACCGTGCACCTGTGGCAGCTCGGACCTTTACTTGGTCACGAGGCATGCCGATGTCATTCCGGTGSGCCGGCGGGGCGACAGCAGGGGAAGCCTACTCTCCCCCAGGCCCGTCTCCTACTTGAAGGGCTCCTCGGGTGGTCCGCTGCTCTGCCCCTCGGGGCGTGCTGTAGGCATCTTCCGGGCTGCTGTGTGCACCCGGGGGGTTGCAAAGGCGGTGGACTTTGTGCCCGTTGAGTCTATGGAAACAACTATGCGGTCCCCGGTCTTCACGGACAACTCGTCCCCCCCGGCCGTACCGCAGACATTTCAAGTGGCCCATCTACACGCTCCCACTGGCAGCGGCAAGAGCACCAAGGTGCCGGCTGCGTATGCAGCCCAAGGGTACAAGGTACTTGTCCTGAACCCGTCTGTCGCCGCCACCCTAAGTTTCGGGGCGTATATGTCTAAAGCACATGGTGTCGACCCCAACATCAGAACTGGGGTGAGGACCATCACCACGGGTGCCCCCATTACATACTCCACCTATGGCAAGTTTCTTGCCGACGGTGGTTGCTCTGGGGGCGCCTATGACATCATAATATGCGATGAGTGCCACTCAACTGACTCGACTACCATCTTGGGCATCGGCACGGTCCTGGACCAAGCGGAGACGGCTGGAGCGCGACTCGTCGTACTCGCCACCGCTACGCCTCCGGGATCGGTCACCGTGCCACATCCCAACATCGAGGAGGTGGCTCTGTCCAACAYTGGAGAGATCCCCTTCTATGGCAAAGCCATCCCTATCGAGACCATCAAGGGGGNNNNNNNNNNNNNNNNNNNNNNNNNNNNNNNNNNNNNNNNNNNNNNNNNNNNNNNNNNNNNNNNNNNNNNNNNNNNNNNNNNNNNNNNNNNNNNNNNNNNNNNNNNNNNNNNNNNNNNNNNNNNNNNNNNNNNNNNNNNNNNNNNNNNNNNNNNNNNNNNNNNNNNNNNNNNNNNNNNNNNNNNNNNNNNNNNNNNNNNNNNNNNNNNNNNNNNNNNNNNNNNNNNNNNNNNNNNNNNNNNNNNNNNNNNNNNNNNNNNNNNNNNNNNNNNNNNNNNNNNNNNNNNNNNNNNNNNNNNNNNNNNNNNNNNNNNNNNNNNNNNNNNNNNNNNNNNNNNNNNNNNNNNNNNNNNNNNNNNNNNNNNNNNNNNNNNNNNNNNNNNNNNNNNNNNNNNNNNNNNNNNNNNNNNNNNNNNNNNNNNNNNNNNNNNNNNNNNNNNNNNNNNNNNNNNNNNNNNNNNNNNNNNNNNNNNNNNNNNNNNNNNNNNNNNNNNNNNNNNNNNNNNNNNNNNNNNNNNNNNNNNNNNNNNNNNNNNNNNNNNNNNNNNNNNNNNNNNNNNNNNNNNNNNNNNNNNNNNNNNNNNNNNNNNNNNNNNNNNNNNNNNNNNNNNNNNNNNNNNNNNNNNNNNNNNNNNNNNNNNNNNNNNNNNNNNNNNNNNNNNNNNNNNNNNNNNNNNNNNNNNNNNNNNNNNNNNNNNNNNNNNNNNNNNNNNNNNNNNNNNNNNNNNNNNNNNNNNNNNNNNNNNNNNNNNNNNNNNNNNNNNNNNNNNNNNNNNNNNNNNNNNNNNNNNNNNNNNNNNNNNNNNNNNN

>HM106732

GCGCCCATCACGGCCTATGCCCAACAGACGCGGGGCCTACTTGGCTGCATCGTCACCAGCCTCACAGGCCGGGACAAGAACCAGGTCGAGGGAGAGGTTCAGGTGGTTTCCACCGCAACACAATCTTTCCTGGCGACCTGCGTCAACGGCGTGTGTTGGACTGTCTACCACGGCGCCGGCACAAGGACCCTTGCCGGCCAAAAGGGCCCAATCACTCAAATGTACACCAATGTAGACCAGGACCTCGTCGGCTGGCAAGCGCCYCCCGGGGCGCGATCCTTGACACCGTGCACCTGTGGCAGCTCGGACCTTTACTTGGTCACGAGGCATGCTGATGTCATTCCGGTGSGCCGGCGGGGCGACAGMAGAGGAAGYCTACTCTCCCCCAGGCCCGTCTCCTACCTGAAGGGCTCTTCGGGTGGACCACTGCTCTGCCCCTCGGGGCATGCTGTAGGCATCTTCCGGGCCGCTGTGTGCACCCGGGGGGTTGCGAAGGCGGTGGACTTTGTGCCCGTTGAGTCCATGGAAACAACTATGCGGTCCCCGGTCTTCACGGAYAACTCRTCTCCCCCGGCTGTACCGCAGACATTCCAAGTGGCCCATCTACACGCTCCCACTGGCAGCGGCAAGAGCACTAAGGTGCCGGCTGCGTATGCAGCCCAAGGGTACAARGTACTCGTCCTGAACCCGTCCGTTGCCGCCACCCTAGGTTTCGGGGCGTATATGTCTAAGGCACATGGTGTCGACCCTAACATCAGAACTGGGRTGAGGACCATCACCACGGGTGCTCCCATTACGTACTCCACCTACGGCAAGTTTCTCGCCGACGGTGGTTGCTCTGGGGGCGCCTACGACATCATAATATGTGATGAGTGCCACTCAACTGACTCGACCACCATCTTGGGCATCGGCACAGTCCTGGACCAAGCGGAGACGGCTGGAGCGCGACTCGTCGTGCNNNNNNNNNNNNNNNNNNNNNNNNNNNNNNNNNNNNNNNNNNNNNNNNNNNNNNNNNNNNNNNNNNNNNNNNNNNNNNNNNNNNNNNNNNNNNNNNNNNNNNNNNNNNNNNNNNNNNNNNNNNNNNNNNNNNNNNNNNNNNNNNNNNNNNNNNNNNNNNNNNNNNNNNNNNNNNNNNNNNNNNNNNNNNNNNNNNNNNNNNNNNNNNNNNNNNNNNNNNNNNNNNNNNNNNNNNNNNNNNNNNNNNNNNNNNNNNNNNNNNNNNNNNNNNNNNNNNNNNNNNNNNNNNNNNNNNNNNNNNNNNNNNNNNNNNNNNNNNNNNNNNNNNNNNNNNNNNNNNNNNNNNNNNNNNNNNNNNNNNNNNNNNNNNNNNNNNNNNNNNNNNNNNNNNNNNNNNNNNNNNNNNNNNNNNNNNNNNNNNNNNNNNNNNNNNNNNNNNNNNNNNNNNNNNNNNNNNNNNNNNNNNNNNNNNNNNNNNNNNNNNNNNNNNNNNNNNNNNNNNNNNNNNNNNNNNNNNNNNNNNNNNNNNNNNNNNNNNNNNNNNNNNNNNNNNNNNNNNNNNNNNNNNNNNNNNNNNNNNNNNNNNNNNNNNNNNNNNNNNNNNNNNNNNNNNNNNNNNNNNNNNNNNNNNNNNNNNNNNNNNNNNNNNNNNNNNNNNNNNNNNNNNNNNNNNNNNNNNNNNNNNNNNNNNNNNNNNNNNNNNNNNNNNNNNNNNNNNNNNNNNNNNNNNNNNNNNNNNNNNNNNNNNNNNNNNNNNNNNNNNNNNNNNNNNNNNNNNNNNNNNNNNNNNNNNNNNNNNNNNNNNNNNNNNNNNNNNNNNNNNNNNNNNNNNNNNNNNNNNNNNNNNNNNNNNNNNNNNNNNNNNNNNNNNNNNNNNNNNNNNNNNNNNNNNNNNNNNNNNNNNNNNNNNNNNNNNNNNNNNNNNNNNNNNNNNNN

>HM106733

GCGCCCATCACGGCCTATGCCCAGCAGACGCGAGGCCTACTTGGCTGCATCGTCACCAGCCTCACAGGCCGGGACAAGAACCAGGTCGAGGGGGAGGTTCAAGTGGTTTNNNNNNNNNNNNNNNNNNNNNNNNNGACTTGCGTCAAYGGCGTGTGTTGGACTGTCTACCACGGCGCCGGCACAAAGACCCTCGCCGGCCAAAAGGGCCCAATCACCCAAATGTACACCAATGTAGACCAGGACCTCGTCGGCTGGCAAGCGCCTCCYGGGGCGCGATCCYTGACACCGTGCACCTGTGGCAGCTCGGACCTTTACTTGGTCACGAGGCATGCTGATGTCATTCCGGTGSGCCGGCGGGGCSACRGMAGAGGAGCYCTACTCTCCCCCAGGCCCGTCTCTTACTTGAAGGGCTCTTCGGGYGGTCCACTGCTCTGCCCCTCGGGGCATGCTGTGGGCATCTTCCGGGCTGCTGTGTGCACYCGGGGGGTTGCGAAGGCGGTAGACTTTGTGCCCGTTGAGTCTATGGAAACAACTATGCGGTCCCCGGTCTTCACGGACAACTCGTCCCCCCCGGCCGTACCGCAGACATTCCAAGTGGCCCATCTCCACGCGCCCACTGGCAGCGGCAAGAGCACTAAGGTGCCGGCTGCGTAYGCGGCCCAGGGGTACAAGGTACTCGTCCTGAACCCGTCTGTTGCCGCCACCCTRGGTTTCGGGGCGTATATGTCTAAGGCACATGGTGTCGACCCCAACATCAGAACTGGGGTAAGGACCATCACCACGGGTGSCCCCATTACGTACTCCACCTATGGCAAGTTYCTTGCCGAYGGYGGGTGCTCTGGGGGCGCCTATGACATCATAATATGTGATGAGTGCCACTCAACTGACTCGACTACCATCTTGGGCATCGGCACAGTCCTGGACCAAGCGGAGACGGCTGGAGCGCGACTTGTCGTACTCGCCACCGCTACGCCTCCGGGATCGGTCACCGTGCCACATCCCAATATCGAGGAGGTGGCTCTGTCCAACACTGGAGAGATCCCCTTCTATGGYAAAGCYATCCCCATCGAGNNNNNNNNNNNNNNNNNNNNNNNNNNNNNNNNNNNNNNNNNNNNNNNNNNNNNNNNNNNNNNNNNNNNNNNNNNNNNNNNNNNNNNNNNNNNNNNNNNNNNNNNNNNNNNNNNNNNNNNNNNNNNNNNNNNNNNNNNNNNNNNNNNNNNNNNNNNNNNNNNNNNNNNNNNNNNNNNNNNNNNNNNNNNNNNNNNNNNNNNNNNNNNNNNNNNNNNNNNNNNNNNNNNNNNNNNNNNNNNNNNNNNNNNNNNNNNNNNNNNNNNNNNNNNNNNNNNNNNNNNNNNNNNNNNNNNNNNNNNNNNNNNNNNNNNNNNNNNNNNNNNNNNNNNNNNNNNNNNNNNNNNNNNNNNNNNNNNNNNNNNNNNNNNNNNNNNNNNNNNNNNNNNNNNNNNNNNNNNNNNNNNNNNNNNNNNNNNNNNNNNNNNNNNNNNNNNNNNNNNNNNNNNNNNNNNNNNNNNNNNNNNNNNNNNNNNNNNNNNNNNNNNNNNNNNNNNNNNNNNNNNNNNNNNNNNNNNNNNNNNNNNNNNNNNNNNNNNNNNNNNNNNNNNNNNNNNNNNNNNNNNNNNNNNNNNNNNNNNNNNNNNNNNNNNNNNNNNNNNNNNNNNNNNNNNNNNNNNNNNNNNNNNNNNNNNNNNNNNNNNNNNNNNNNNNNNNNNNNNNNNNNNNNNNNNNNNNNNNNNNNNNNNNNNNNNNNNNNNNNNNNNNNNNNNNNNNNNNNNNNNNNNNNNNNNNNNNNNNNNNNNNNNNNNNNNNNNNNNNNNNNNNNNNNNNNNNNNNNNNNNNNNNNNNNNNNNNNNNNNNNNNNNNNNNNNNNNNNNNNNN

>HM106734

GCGCCCATCACGGCCTAYGCCCAACAGACGCGRGGCCTACTTGGCTGCATCATCACYAGCCTCACAGGCCGGGACAAGAACCAGGTCGAGGGGGAGGTTCARGTGGTTTCCACCGCAACACAATCTTTCCTGGCGACCTGYGTCAACGGCGTGTGTTGGACTGTCTACCACGGCGCCGGCACAAAGACCCTCGCCGGCCAAAAGGGSCCRATCACCCAAATGTACACCAATGTAGACCAGGACCTCGTCGGCTGGCAAGCGCCYYCCGGGGCGCGGTCCTTGACACCGTGCACCTGTGGCAGCTCGGACCTTTACTTGGTCACGAGGCATGCTGATGTCATCCCGGTGCGCCGGCGGGGCGACAGWAGAGGAAGYCTACTCTCCCCCAGGCCCGTCTCCTACTTGAAGGGCTCTTCGGGTGGTCCACTGCTCTGCCCCTCGGGGCATGCTGTGGGCATCTTCCGGGCTGCTGTGTGCACCCGGGGGGTTGCGAAGGCGGTGGACTTTGTGCCCGTTGAGTCTATGGAAACAACTATGCGGTCCCCGGTCTTCACGGACAACTCGTCCCCCCCGGCCGTACCGCAGACATTCCAAGTGGCCCATCTACACGCTCCCACTGGCAGCGGCAAAAGCACTAAGGTGCCGGCTGCGTATGCAGCCCAAGGGTACAAGGTACTCGTCCTCAACCCGTCTGTTGCCGCCACCCTGGGTTTCGGGGCGTATATGTCTAAGGCACATGGTGTCGACCCTAACATCAGAACTGGGGTAAGGACCATCACCACGGGTGCCCCCATTACGTATTCCACCTATGGCAAGTTTCTTGCCGACGGTGGTTGCTCTGGGGGCGCCTATGACATCATAATATGTGATGAGTGCCATTCAACTGACTCGACTACCATCTTGGGCATCGGCACAGTCCTGGACCAAGCGGAGACGGCTGGAGCGCGACTCGTCGTGCTCGCCACCGCTACGCCTCCGGGATCGGTCACCGTGCCACATCCCAATATCGAGGAGGTGGCTCTGTCCAACATCGGAGAGATCCCCTTCTATGGTAAAGCCATCCCCATCGAGACCATCAAGGGGGGGAGGCACCTNNNNNNNNNNNNNNNNNNNNNNNNNNNNNNNNNNNNNNNNNNNNNNNNNNNNNNNNNNNNNNNNNNNNNNNNNNNNNNNNNNNNNNNNNNNNNNNNNNNNNNNNNNNNNNNNNNNNNNNNNNNNNNNNNNNNNNNNNNNNNNNNNNNNNNNNNNNNNNNNNNNNNNNNNNNNNNNNNNNNNNNNNNNNNNNNNNNNNNNNNNNNNNNNNNNNNNNNNNNNNNNNNNNNNNNNNNNNNNNNNNNNNNNNNNNNNNNNNNNNNNNNNNNNNNNNNNNNNNNNNNNNNNNNNNNNNNNNNNNNNNNNNNNNNNNNNNNNNNNNNNNNNNNNNNNNNNNNNNNNNNNNNNNNNNNNNNNNNNNNNNNNNNNNNNNNNNNNNNNNNNNNNNNNNNNNNNNNNNNNNNNNNNNNNNNNNNNNNNNNNNNNNNNNNNNNNNNNNNNNNNNNNNNNNNNNNNNNNNNNNNNNNNNNNNNNNNNNNNNNNNNNNNNNNNNNNNNNNNNNNNNNNNNNNNNNNNNNNNNNNNNNNNNNNNNNNNNNNNNNNNNNNNNNNNNNNNNNNNNNNNNNNNNNNNNNNNNNNNNNNNNNNNNNNNNNNNNNNNNNNNNNNNNNNNNNNNNNNNNNNNNNNNNNNNNNNNNNNNNNNNNNNNNNNNNNNNNNNNNNNNNNNNNNNNNNNNNNNNNNNNNNNNNNNNNNNNNNNNNNNNNNNNNNNNNNNNNNNNNNNNNNNNNNNNNNNNNNNNNNNNNNNNNNNNNNNNNNNNNNNNNNNNNNNNNNNNNNNNNNNNNNNNNNNNNNNNNNNNN

>HM106735

GCGCCCATCACGGCCTATGCCCAACAGACGCGGGGCCTACTTGGCTGYATCGTCACCAGCCTCACAGGCCGGGACAAGAACCAGGTCGAGGGGGAGGTTCAANNNNNNNNNNNNNNNNNNNNNNNNNNNNNNNNNNNNNNNNNNNNNNNNNNNNNNNNNNNNNNNNNNNNNNNNNNNNNNNNNNNNNNNNNNNNNNNNNNNNNNNNCCCAATTACCCAAATGTACACCAATGTAGACCAGGACCTCGTCGGCTGGCAGGCGCCTCCCGGGGCGCGATCCCTGACACCGTGCACCTGTGGCAGCTCGGACCTTTACTTGGTCACGAGGCATGCTGATGTCATTCCGGTGCGCCGGCGGGGCGACAGMAGAGGAAGYCTACTCTCCCCCAGGCCCGTCTCCTACCTGAAGGGCTCTTCGGGTGGTCCACTGCTCTGCCCCTCGGGGCATGCTGTGGGCATCTTCCGGGCTGCCGTGTGCACCCGGGGGGTCGCGAAGGCGGTGGACTTTGTGCCCGTTGAGTGTATGGAAACAACTATGCGGTCCCCGGTCTTCACGGACAACTCGTCCCCCCCGGCCGTACCGCAGACATTCCAAGTGGCCCATCTACACGCTCCCACTGGCAGCGGCAAGAGCACTAAGGTGCCGGCTGCGTATGCAGCCCAAGGGTACAAGGTACTCGTCCTGAACCCGTCTGTTGCCGCCACCCTAGGTTTCGGGGCGTATATGTCTAAGGCACATGGTGTCGACCCTAACATCAGAACTGGGGTGAGGACCATCACCACGGGTGCCCCCATTACGTACTCCACYTATGGCAAGTTTCTYGCCGACGGTGGTTGYTCTGGGGGCGCCTATGACATCATAATWTGTGATGAGTGCCACTCAACTGACTCAACTACCATCTTGGGCATCGGCACAGTCCTGGACCAAGCGGAGACGGCTGGAGCGCGACTCGTCGTACTCGCCACCGCTACGCCTCCGGGATCGGTCACCGTGCCACATCCCAACATCGAGGAGGTGGCTCTGTCCANNNNNNNNNNNNNNNNNNNNNNNNNNNNNNNNNNNNNNNNNNNNNNNNNNNNNNNNNNNNNNNNNCTCATATTTTGCCATTCCAGGAARAAATGTGATGAGCTCGCCGCAAAGYTGTCGGGCCTCGGGCTTAACGCTGTAGCGTATTACCGGGGYCTTGATGTGTCYGTCATACCSGCCAGCGGAGACGTCGTTGTCGTGGCAACAGACGCTCTAATGACGGGTTTCACTGGCGACTTTGACTCAGTRATCGACTGTAATACATGTGTCACCCAGACAGTCGACTTCAGCTTGGACCCYACYTTCACCATTGARACRACGACCGTGCCTCAAGACGCGGTGTCGCGCTCGCAGCGGCGAGGCAGGACTGGTAGGGGCAGGRCAGGCATCTACAGRTTTGTGACTCCAGGAGAACGGCCCTCGGGCATGTTCGATTCCTCGGTCCTGTGTGAGTGCTATGACGCGGGCTGYGCTTGGTACGAGCTCACGCCYGCCGAGACCACAGTTAGGTKGCGGGCTTACCTGAATACACCAGGGTTGCCCGTCTGCCAGGAYCAYCTGGAGTTCTGGGAGGGCGTCTTCACAGGCCTCACCCACATAGATGCCCACTTCYTGTCCCAGACTAAGCARGCAGGAGACAACTTCCCCTACTTRGTAGCATACCAGGCYACGGTGTGCGCCAGGGCTCAGGCTCCACCCCCATCGTGGGACCAAATGTGGAAATGTCTCATACGGCTAAAGCCCACGCTGCACGGGCCAACACCCCTGCTGTATAGGCTAGGAGCCGTCCAAAATGAGGTCAYCCTCACACAYCCCATAACCAAATACATCATGACATGCATGTCGGCTGACCTGGAGGTCGTCACG

>HM106736

GCGCCCATCACGGCCTATGCCCAGCAGACGCGAGGCCTACTCGGCTGCATCGTCACCAGCCTCACAGGCCGGGACAAGAACCAGGTCGAGGGGGAGGTTCAAGTGGTTTCTACCGCAACACAATCTTTCCTGGCGACCTGCGTTAACGGCGTGTGTTGGACTGTTTACCACGGTGCCGGCACAAAGACCCTCGCCGGCCAGAAGGGCCCAATCACCCAAATGTACACCAATGTAGACCAGGACCTCGTMGGCTGGCAAGCGCCTCCCGGGGCGCGATCCTTGACACCGTGCACCTGTGGCAGCTCGGACCTTTACTTGGTCACGAGGCATGCTGATGTCATTCCGGTGSGCCGGCGGGGCGACAGCAGAGGAAGCCTACTCTCCCCCAGGCCCGYCTCCTACTTGAAGGGCTCTTCGGGTGGTCCACTGCTCTGCCCCTCGGGGCATGCTGTGGGCATCTTCCGGGCTGCTGTGTGCACCCGGGGGGTTGCGAAGGCGGTGGACTTTGTGCCCGTTGAGTCTATGGAAACAACTATGCGGTCCCCGGTCTTCACGGACAACTCGTCCCCCCCGGCTGTACCGCAGACATTCCAAGTGGCCCATCTACACGCCCCCACTGGCAGCGGCAAGAGCACTAAGGTGCCGGCTGCGTATGCAGCCCAAGGGTACAAGGTACTCGTCCTGAACCCGTCCGTTGCCGCCACCTTAGGTTTCGGGGCGTATATGTCTAAGGCATATGGTGTCGACCCTAACATCAGAACTGGGGTAAGGACCATCACCACGGGTGCCCCCATTACGTACTCCACYTACGGCAAATTCCTYGCCGACGGTGGTTGYTCTGGGGGCGCCTACGACATCATAATWTGTGATGAGTGCCACTCAACTGACTCGACTACCATCTTGGGCATCGGCACAGTCCTGGACCAAGCGGAGACGGCKGGAGCGCGACTCGTCGTACTCGCCACCGCTACGCCTCCGGGATCGGTCACCGTGCCACATCCCAACATCGAGGAGGTGGCTCTGTCCAACACTGGAGAGATCCCCTTCTATGGTAAAGCCATCCCCATCGAGACTATCAAGGGGGGGAGGCACCTCATATTTTGYCATTCCAGGAGGAAATGTGATGAGCTCGCCGCAAAGCTGTCGAGTCTCGGGCTTAACGCTGTAGCGTACTACCGGGGTCTTGACGTGTCCGTCATACCGACCAGCGGAGACGTCGTTGTYGTGGCAACAGACGCTCTAATGACGGGTTTCACTGGCGACTTTGACTCAGTCATCGACTGTAAYACATGTGTCACCCAGACAGTCGACTTCAGCTTGGACCCTACCTTCACCATTGAGACGACGACCGTGCCCCARGACGCGGTGTCGCGCTCGCAGCGGCGAGGCAGGACTGGTAGGGGCAGGACAGGCATCTACAGGTTTGTGACTCCGGGAGAACGGCCCTCGGGTATGTTCGATACCTCAGTCCTATGTGAGTGCTATGACGCGGGCTGTGCGTGGTACGAGCTCACGCCCGCCGAGACCACAGTTAGGTTGCGGGCTTACCTGAATACACCAGGGTTGCCCGTCTGCCAGGACCACCTGGAGTTCTGGGAGGGCGTCTTCACAGGCCTCACCCACATAGATGCCCACTTCCTGTCCCAGACTAAACAGGCAGGAGACAACTTCCCCTAYTTGGTAGCATACCAGGCTACGGTGTGCGCCAGAGCTCAGGCTCMACCCCCATCGTGGGACCAGATGTGGAAATGTCTCATACGGCTAAAGCCCACGCTGCACGGGCCAACACCCCTGCNNNNNNNNNNNNNNNNNNNNNNNNNNNNNNNNNNNNNNNNNNNNNNNNNNNNNNNNNNNNNNNNNNNNNNNNNNNNNNNNNNNNNNNNNNNNNNNNNN
