# Supplementary material for: Effect of Immune Pressure on Hepatitis C Virus Evolution: Insights From a Single-Source Outbreak
Source: Hepatology. 2011 Feb;53(2):396–405. doi: 10.1002/hep.24076 (PMC3044208; doi:10.1002/hep.24076)
Supplement: Supplementary file 7 [file hep0053-0396-SD7.doc]

>HM106737

AGCACCTGGGTGCTAGTAGGCGGAGTCCTCGCAGCTCTGGCCGCGTATTGCCTGACAACAGGCAGCGTGGTCATTGTAGGCAGGGTCATCTTGTCCGGGAAGCCGGCTATTATACCTGACAGGGAAGTCCTCTACCAGCAGTTCGATGAGATGGAAGAGTGT

>HM106738

AGCACCTGGGTGCTAGTAGGCGGGGTCCTCGCAGCTCTGGCCGCGTATTGCCTAACAACAGGCAGCGTGGTCATTGTGGGCAGGATCATCTTGTCCGGGAAGCCGGCTATTGTACCCGACAGGGAAGTCCTCTACCAGCAGTTCGATGAGATGGAAGAGTGC

>HM106739

AGCACCTGGGTGCTAGTAGGCGGAGTCCTCGCAGCTCTGGCCGCATATTGCCTGACAACAGGCAGTGTGGTCATTGTGGGCAGGATCATCTTGTCCGGAAAGCCGGTTGTTGTGCCCGACAGGGAAGTCCTCTACCAACAGTTCGATGAAATGGAAGAGTGT

>HM106740

AGCACCTGGGTGCTAGTAGGCGGAGTCCTCGCAGCTCTGGCCGCGTATTGCCTGACAACAGGCAGCGTGGTCATTGTRGGCAGGATCATCTTGTCCGGGAAGCCGGCCATTATACCCGATAGGGAAGTCCTCTACCAGCAGTTTGATGAGATGGAAGAGTGC

>HM106741

AGCACCTGGGTGCTAGTAGGCGGAGTCCTCGCAGCTCTGGCCGCGTAYTGCCTRACAACAGGCAGCGTGGTCATTGTRGGCAGGATCATCTTGTCCGGAAAGCCGGCYATTATACCWGACAGGGAAGTCCTCTACCAGCAGTTCGATGAGATGGAAGAGTGY

>HM106742

AGCACCTGGGTGCTAGTAGGCGGAGTCCTTGCAGCTCTGGCCGCGTATTGCCTGACAACAGGCAGCGTGGTCATTGTAGGCAGGATCATCTTGTCCGGAAAGCCGGCTATTGTACCTGACAGGGAAGTCCTCTACCAGCAGTTCGATGAGATGGAAGAGTGT

>HM106743

AGCACCTGGGTGCTAGTGGGCGGAGTCCTCGCAGCTCTGGCCGCGTACTGCCTGACAACAGGCAGCGTGGTCATTGTRGGCAGGATCATCTTGTCCGGAAAGCCGGCTATTGTACCYGACAGGGAAGTCCTCTACCAGCAGTTCGATGAGATGGAAGAGTGC

>HM106744

AGCACCTGGGTGCTAGTAGGCGGAGTCCTCGCAGCTCTGGCTGCGTATTGCCTAACAACAGGCAGCGTGGTCATTGTGGGCAGGATCATCTTGTCCGGAAAGCCGGCCATTCTACCCGACAGGGAAGTCCTCTACCAGCAGTTCGATGAGATGGAAGAGTGT

>HM106745

AGCACCTGGGTGCTAGTAGGCGGAGTCCTCGCAGCTCTGGCCGCGTATTGCYTRACAACAGGCAGCGTGGTCATTGTAGGCAGGATYATCTTGTCCGGRAAGCCGGCTATTGTACCCGACAGGGAAGTCCTCTACCAGCAGTTCGATGAAATGGAAGAGTGC

>HM106746

AGCACCTGGGTGCTAGTAGGCGGAGTCCTCGCGGCTCTGGCCGCGTATTGCCTGACAACAGGCAGCGTGGTCATTGTAGGCAGGATCATCTTGTCCGGAAAGCCGGCTATTGTACCCGACAGGGAAGCCCTCTACCAGCAGTTCGATGAGATGGAAGAGTGC

>HM106747

AGCACCTGGGTGCTAGTAGGCGGAGTCCTCGCAGCTCTGGCCGCGTATTGCCTGACAACAGGCAGCGTGGTCATTGTRGGCAGGATCATCTTGTCCGGRAAGCCGGCTATTGTGCCCGACAGGGAAGTCCTCTACCARCAGTTCGATGAGATGGAAGAGTGT

>HM106748

AGCACCTGGGTGCTAGTAGGCGGAGTCCTCGCAGCTCTGGCCGCGTATTGCCTGACAACAGGCAGCGTGGTCATTGTAGGCAGGATCATCTTGTCCGGGAAGCCGGCCATTGTACCCGACAGGGAAGTCCTCTACCAGCAGTTCGATGAGATGGAAGAGTGC

>HM106749

AGCACCTGGGTGCTAGTAGGTGGGGTCCTTGCAGCTCTGGCCGCGTATTGCCTGACAACAGGCAGCGTGGTCATCGTAGGCAGGATCATCTTGTCCGGAAAGCCGGCTATTGTGCCCGACAGGGAAGCCCTCTACCAGCAGTTCGATGAGATGGAAGAGTGT

>HM106750

AGCACCTGGGTGCTAGTGGGCGGAGTCGTCGCAGCTCTGGCCGCGTACTGCCTGACAACGGGCAGCGTGGTCATTGTGGGCAGGATCATCTTGTCCGGGAAGCCGGCTGTTGTACCCGACAGGGAAGTCCTCTACCAGCAGTTCGATGAGATGGAAGAGTGC

>HM106751

AGCACCTGGGTGCTAGTAGGCGGAGTCCTCGCAGCTCTGGCCGCGTATTGCCTGACAACAGGCAGCGTGGTCATTGTAGGCAGGATCATCTTGACCGGAAAGCCGGCTGTTATACCTGACAGGGAAGTCCTCTACCAGCAGTTCGATGAGATGGAAGAGTGC

>HM106752

AGCACCTGGGTGCTAGTAGGCGGAGTCCTCGCAGCTCTGGCCGCGTATTGCCTGACAACAGGCAGCGTGGTCATTGTAGGCAGGATCATCTTGTCTGGAAAGCCGNNNNNNNNNNNNNNNNNNNNNNNNNNNNNNNNNNNNNNNNNNNNNNNNNNNNNNNNN

>HM106753

AGCACCTGGGTGCTAGTGGGCGGGGTCCTTGCAGCTCTGGCCGCGTATTGCCTGACAACAGGCAGCGTGGTCATCGTAGGCAGGATCATCTTGTCCGGAAAGCCGGCTATTGTACCCGACAGGGAGGTCCTCTACCAGCAGTTCGATGAGATGGAAGAGTGC

>HM106754

AGYACCTGGGTGCTAGTAGGCGGRGTCCTCGCAGCTCTGGCYGCGTATTGCCTGACAACAGGCAGCGTGGTCATTGTAGGCAGGATCGTCTTGTCCGGRAAGCCGGYYRTTGTACCCGACAGGGAAGTCCTCTACCAGCAGTTCGATGARATGGAAGAGTGC

>HM106755

AGCACTTGGGTGCTAGTAGGCGGAGTCCTCGCCGCTCTGGCCGCGTATTGCCTGACAACAGGCAGCGTGGTCATTGTAGGCAGGATCATCTTGTCCGGAAAGCCGGCCATTGTACCCGACAGGGAAGTCCTCTACCAACAGTTCGATGAAATGGAAGAGTGC

>HM106756

AGCACCTGGGTGCTAGTAGGCGGAGTCATCGCAGCTCTGGCCGCGTATTGCTTGACAACAGGCAGCGTGGTCATCGTAGGCAGGATCGTCTTGTCTGGGAAGCCGGCTATTGTGCCCGACAGGGAAGTCCTCTACCAGCAGTTCGATGAGATGGAAGAGTGC

>HM106757

AGCACCTGGGTGCTAGTGGGCGGAGTCCTTGCAGCTCTGGCCGCGTATTGCCTGACAACAGGCAGCGTGGTCATTGTAGGCAGGATCATCTTGTCCGGAAAGCCGGCTATTGTACCTGACAGGGAAGTCCTCTACCAGCAGTTCGATGAGATGGAAGAGTGT

>HM106758

AGCACCTGGGTGCTAGTGGGCGGGGTCCTCGCAGCTCTGGCCGCGTATTGCCTGACAACAGGCAGCGTGGTCATCGTAGGCAGGATCATCTTGTCCGGGAAGCCGGCCATCGTACCCGACAGGGAAGTCCTCTACCAGCAGTTCGATGAGATGGAAGAGTGT

>HM106759

AGTACTTGGGTGCTCGTYGGYGGCGTTCTGGCTGCTCTGRCCGCRTATTGCCTATCCACAGGCTGCGTGGTCATAGTAGGCAGGATTGTTTTGTCCGGGAAGCCGGCGRTCATACCYGACAGGGARGTCCTCTACCGGGAGTTCGATGAGATGGAAGAGTGC

>HM106760

AGCACCTGGGTGCTAGTRGGCGGRGTCCTCGCAGCTCTGGCCGCGTATTGCCTGACAACAGGCAGCGTGGTCATTGTRGGCAGGATCATCTTGTCCGGRAAGCCGGCTATTGTRCCYGACAGGGAAGTCCTCTACCARCAGTTCGATGAGATGGAAGAGTGC
