# Supplementary material for: Effect of Immune Pressure on Hepatitis C Virus Evolution: Insights From a Single-Source Outbreak
Source: Hepatology. 2011 Feb;53(2):396–405. doi: 10.1002/hep.24076 (PMC3044208; doi:10.1002/hep.24076)
Supplement: Supplementary file 8 [file hep0053-0396-SD8.doc]

>HM106761

GCCACACACCTCCCTTACATTGAACAAGGAATGCAGCTCGCCGAACAATTCAAGCAGAAGGCACTCGGGTTGTTACAGACAGCCACCAAGCAAGCAGAGGCTGCTGCTCCSGTGGTGGAGTCCAAGTGGCGAGCCCTTGAGAGCTTCTGGGCGAAGCATATGTGGAATTTCATCAGCGGGATACAATAYTTAGCAGGCTTGTCCACTCTGCCTGGKAACCCCGCGATAGCATCAYTGATGGCATTCACAGCCTCTATCACCAGCCCGCTCACYACCCAACATACCCTCCTGTTCAAYATYTTGGGGGGATGGGTGGCCGCCCAACTCGCCCCYCCCAGCGCTGCTTCTGCTTTCGTAGGCGCCGGCATYGCCGGCGCGGCCGTTGGCAGCATAGGCCTTGGGAAGGTRCTGGTGGACATCTTGGCGGGTTATGGAGCGGGGGTRGCAGGCGCCCTCGTGGCCTTTAAGGTCATGAGTGGTGAGATGCCYTCCACTGAGGACTTAGTCAAYYTGCTCCCTGCCATCCTCTCCCCTGGTGCCCTAGTCGTCGGGGTAGTGTGCGCWGCAATACTGCGYCGGCATGTGGGCCCAGGGGAGGGGGCYGTGCAGTGGATGAACCGGCTGATAGCGTTCGCTTCGCGGGGTAACCAYGTCTCCCCCACGCACTATGTGCCCGAGAGCGACGCTGCRGCGCGTGTYACTCAGATCCTCTCCAGYCTTACCATCACTCAGCTGCTGAAGAGGCTCCATCAGTGGATTAAYGAGGACTGCTCCACGCCATGC

>HM106762

GCCACGCACCTCCCTTACNNNNNNNNNNNNNNNNNNNNNNNNNNNNNNNNNNNNNNNNNNNNNNNNNNNNNNNNNNNNNNNNNNNNNNNNNNNNNNNNNNNNNNNNNNNNNNNNNNNNNNNNNNNNNNNNNNNNNNNNNNNNNNNNNTGGGCGAAGCATATGTGGAATTTCATCAGCGGGATACAATACTTAGCAGGCTTGTCCACTCTGCCTGGGAACCCCGCAATAGCATCACTGATGGCATTCACAGCCTCCATCACCAGCCCGCTCACCACCCAACATACCCTCCTGTTCAACATCTTGGGGGGATGGGTGGCCGCCCAACTTGCCCCCCCCAGCGCTGCTTCTGCTTTCGTAGGCGCCGGCATCGCCGGCGCGGCCGTTGGCAGCATAGGCCTTGGGAAGGTGCTAGTGGACATCTTGGCGGGTTACGGAGCGGGGGTGGCAGGCGCCCTCGTGGCCTTCAAGGTCATGAGTGGCGAGATGCCTTCCACTGAGGACTTGGTCAACTTGCTCCCTGCCATCCTCTCTCCTGGTGCCCTAGTCGTCGGAGTAGTGTGCGCAGCAATACTGCGCCGGCATGTGGGCCCAGGGGAGGGGGCTGTGCAGTGGATGAACCGGCTGATAGCGTTTGCCTCGCGGGGTAACCACGTCTCCCCCACGCACTATGTGCCCGAGAGCGACGCTGCAGCGCGTGTCACTCAGATCCTCTCCAGCCTTACCATCACTCAGCTGCTGAAGAGGCTCCATCAGTGGATCAATGAGGACTGCTCCACGCCATGC

>HM106763

NNNNNNNNNNNNNNNNNNNNNNNNNNNNNNNNNNNNNNNNNNNNNNNNNNNNNNNNNNNNNNNNNNNNNNNNNNNNNNNNNNNNNNCAAGCAAGCAGAGGCTGCTGCTCCCGTGGTGGAGTCCAAGTGGCGGGCCCTTGAGAGTTTCTGGGCGAAGCATATGTGGAATTTCATCAGCGGGATACAGTACYTAGCAGGCTTGTCYACTCTGCCTGGGAACCCCGCAATAGCATCACTGATGGCTTTCACAGCCTCTATCACCAGCCCGCTCACCACCCAACATACCCTCCTGTTCAACATYTTGGGGGGATGGGTGGCCGCCCAACTCGCCCCCCCCGGCGCCGCTTCTGCTTTCGTAGGCGCCGGCATCGCCGGCGCAGCCGTCGGCAGCATAGGCCTTGGAAAGGTGCTAGTGGACATCCTGGCGGGTTACGGAGCGGGGGTGGCAGGCGCCCTCGTGGCCTTTAAGGTCATGAGTGGTGAGATGCCTTCCACTGAAGACTTGGTCAACTTGCTCCCTGCYATCCTCTCCCCTGGTGCCCTAGTCGTCGGGGTRGTGTGCGCAGCAATACTGCGYCGGCATGTGGGCCCAGGGGAGGGGGCTGTGCAGTGGATGAACCGGCTGATAGCGTTCGCTTCGCGGGGTAACCACGTCTCCCCCACGCACTATGTGCCCGAGAGCGACGCTGCAGCGYGTGTCACTCAGATCCTCTCCAGCCTTACCATCACTCAGCTGCTGAGGAGGCTCCACCAGTGGATTAAYGAGGACTGCTCCACGCCATGC

>HM106764

GCCACACATCTCCCTTACATCGAACAAGGAATGCAGCTCGCCGAACAATTCAAGCAGAAGGCACTCGGGTTGCTACAGACAGCCACTAAGCAAGCAGAGGCTGCTGCTCCCGTGGTGGAGTCCAAGTGGCGAGCCCTTGAGAGCTTCTGGGCGAAGCATATGTGGAATTTCATCAGCGGGATACAATACTTAGCAGGCTTGTCCACTCTGCCTGGGAACCCCGCAATAGCATCACTGATGGCATTCACAGCCTCTATCACCAGCCCGCTTACCACCCACCATACCCTCCTGTTCAACATCTTGGGGGGATGGGTGGCTGCCCAACTCGCCCCCCCCGGCGCTGCTTCTGCTTTCGTAGGCGCCGGCATCGCCGGCGCGGCCGTTGGCAGCATAGGCCTTGGGAAGGTGCTAGTGGACATCTTGGCGGGTTACGGAGCGGGGGTGGCAGGCGCCCTCGTGGCCTTTAAGGTCATGAGTGGTGAGATGCCTTCCACTGAGGACCTGGTCAACTTGCTCCCTGCCATCCTCTCCCCTGGTGCCCTGGTCGTCGGAGTAGTGTGCGCAGCAATACTGCGTCGGCATGTGGGCCCAGGGGAGGGGGCTGTGCAGTGGATGAACCGGCTGATAGCGTTCGCTTCGCGGGGTAACCATGTCTCCCCCACGCACTATGTGCCCGAGAGCGACGCTGCAGCGCGTGTCACTCAGATCCTCTCCAGCCTTACCATCACTCAGCTGTTGAGGAGGCTCCACCAGTGGATTAACGAGGACTGCTCCACGCCATGC

>HM106765

GCCACACACCTCCCTTACATTGAACARGGAATGCAGCTCGCCGAWCAATTCAAGCAGAAGGCACTCGGGTTGTTACAGACAGCCACCAAGCAAGCAGAGGCTGCTGCTCCCGTGGTGGAGTCCAAGTNNNNNNNNNNNNNNNNNNNNNNNNNNAAGCATATGTGGAATTTCATCAGCGGGATACAATACTTAGCAGGCTTGTCCACCCTGCCTGGGAACCCCGCAATAGCATCACTGATGGCATTCACAGCCTCTATCACCAGCCCGCTCACCACCCAGCATACCCTCCTGTTCAACATTTTGGGGGGATGGGTGGCCGCCCAACTCGCCCCCCCCAGCGCTGCTTCTGCTTTCGTAGGCGCTGGCATCGCCGGCGCGGCCGTTGGCAGCATAGGCCTTGGGAAGGTGCTAGTGGACATTTTGGCGGGCTATGGAGCGGGGGTGGCAGGCGCCCTCGTGGCCTTCAAGGTCATGAGCGGTGAGATGCCTTCCACTGAGGACTTGGTCAACTTGCTCCCTGCCATCCTCTCCCCTGGTGCCCTAGTTGTCGGGGTAGTGTGCGCAGCAATACTGCGTCGGCATGTGGGCCCAGGGGAGGGGGCTGTGCAGTGGATGAACCGGCTGATAGCGTTCGCTTCGCGGGGTAACCATGTCTCCCCCACGCACTATGTGCCCGAGAGCGACGCTGCAGCGCGTGTCACTCAGATCCTCTCCAGCCTTACCATCACTCAGCTGCTGAAGAGGCTCCATCAGTGGATCAACGAGGACTGCTCCACGCCATGC

>HM106766

NNNNNNNNNNNNNNNNNNNNNNNNNNNNNNNNNNNNNNNNNNNNNNNNNNNNNNNNNNNNNNNNNNNNNNNNNNNNNNNNNNCCACCAAGCAAGCAGAGGCYGCTGCTCCCGTGGTGGAGTCCAAGTGGCGAGCCCTTGAGAGCTTCTGGGCGAAGCATATGTGGAAYTTCATCAGCGGGATACAATAYTTAGCAGGCTTGTCCACTCTGCCAGGGAACCCCGCAATAGCATCACTGATGGCATTCACAGCCTCTATCACCAGCCCGCTTACCACCCARCAYACCCTCCTGTTCAACATCTTGGGGGGATGGGTGGCYGCYCAACTCGCCCCYCCCAGCGCTGCTTCTGCTTTCGTAGGCGCCGGCATCGCCGGCGCGGCCGTTGGCAGCATAGGCCTTGGGAAGGTGCTAGTGGACATCTTGGCGGGYTATGGRGCGGGGGTGGCRGGCGCCCTCGTGGCCTTYAAGGTCATGAGTGGCGAGATGCCTTCCACTGAGGACTTGGTCAACTTGCTCCCTGCCATCCTCTCCCCTGGCGCCCTRGTCGTCGGGGTAGTGTGCGCAGCAATACTGCGTCGRCATGTGGGCCCAGGGGAGGGGGCTGTGCAGTGGATGAACCGGCTGATAGCGTTCGCTTCGCGGGGTAACCAYGTCTCCCCCACGCACTATGTGCCCGAGAGCGACGCTGCRGCGCGTGTCACTCAGATCCTCTCCAGCCTTACCATCACYCAGCTGCTGAAGAGGCTCCACCAGTGGATTAACGAGGACTGCTCCACGCCGTGC

>HM106767

GCCACACACCTCCCTTACATTGAACAAGGAATGCAGCTCGCCGAACAATTCAAGCAGAAGGCACTCGGGYTRYTGCAGACAGCCACCAAGCAAGCAGAGGCTGCCGCCCCCGTGGTGGAGTCCAAGTGGCGAGCCCTTGAGAGCTTCTGGGCGAAGCATATGTGGAATTTCATCAGCGGGATACAATACTTAGCAGGCTTGTCCACTCTGCCTGGGAACCCCGCAATAGCATCACTGATGGCATTCACAGCCTCTATCACCAGCCCGCTCACCACCCAACATACCCTCCTGTTCAACATTTTGGGGGGATGGGTGGCCGCCCAACTCGCCCCCCCCAGCGCCGCTTCTGCTTTCGTAGGCGCCGGCATCGCCGGCGCGGCCGTCGGCAGCATAGGCCTTGGGAAGGTACTAGTGGACATCTTGGCGGGTTATGGAGCGGGGGTGGCAGGCGCCCTCGTGGCCTTTAAGGTCATGAGTGGTGAGATGCCTTCCACTGAGGACTTGGTCAACTTGCTCCCTGCCATCCTCTCCCCTGGTGCCCTAGTCGTCGGTGTAGTGTGCGCAGCAATACTGCGCCGGCATGTGGGCCCAGGGGAGGGGGCTGTGCAGTGGATGAATCGGCTGATAGCGTTCGCCTCGCGGGGTAACCACGTTTCCCCCACGCACTATGTGCCCGAGAGCGACGCTGCAGCGCGTGTCACTCAGATCCTCTCCAGCCTCACCATCACCCAGCTGCTGAAGAGGCTCCACCAGTGGATTAACGAGGACTGCTCCACGCCATGC

>HM106768

NNNNNNNNNNNNNNNNACATTGAACAAGGAATGCAGCTCGCCGAACAATTCAAGCAGAAGGCACTCGGGTTGTTACAAACAGCCACTAAGCAAGCAGAGGCTGCTGCTCCCGTGGTGGAGTCCAAGTGGCGAGCCCTTGAGAGCTTCTGGGCGAAGCATATGTGGAATTTCATCAGCGGGATACAATACTTAGCAGGCTTGTCCACTCTGCCTGGGAACCCCGCAATAGCATCACTGATGGCATTCACAGCCTCTATCACCAGCCCGCTCACCACCCAACAYACCCTCCTGTTTAACATTCTGGGGGGATGGGTGGCCGCCCAACTCGCCCCCCCTAGCGCTGCCTCTGCTTTCGTAGGCGCCGGCATYGCCGGCGCGGCCGTTGGCAGCATAGGCCTTGGGAAGGTGCTAGTGGACATCTTGGCGGGTTATGGAGCGGGGGTGGCRGGCGCCCTCGTGGCCTTTAAAGTCATGAGTGGTGAGATGCCCTCCACTGAGGACTTGGTCAACTTGCTCCCTGCCATCCTCTCCCCTGGTGCCCTAGTCGTCGGGGTAGTGTGCGCAGCAATACTGCGYCGGCAYGTGGGCCCAGGGGAGGGGGCTGTGCAGTGGATGAACCGGCTGATAGCGTTCGCTTCGCGGGGTAACCACGTCTCCCCCACGCACTATGTGCCCGAGAGCGACGCCGCAGCACGTGTCACCCAGATCCTTTCCAGCCTTACCATCACTCAGCTGCTGAAGAGGCTCCATCAGTGGATYAACGAGGACTGCTCCACGCCATGC

>HM106769

GCCACACACCTCCCTTACATTGAACAAGGAATGCAGCTCGCCGAACAATTCAAGCAGAAGGCACTCGGGTTATTACAGACAGCCACTAAGCAAGCAGAGGCTGCTGCTCCCGTAGTNNNNNNNNNNNNNNNNNNNNNNNNNNNNNNNNNGGCGAAGCATATGTGGAATTTCATCAGCGGGATACAATACTTAGCAGGCTTGTCCACTCTGCCTGGGAACCCCGCAATAGCATCACTGATGGCATTCACAGCCTCTATTACCAGCCCGCTCACCACCCAACATACCCTCCTGTTCAACATCTTGGGAGGGTGGGTGGCTGCCCAACTCGCCCCGCCCGGCGCTGCTTCTGCTTTCGTAGGCGCCGGCATCGCCGGCGCGGCCGTTGGCAGCATAGGCCTTGGGAAGGTGCTAGTGGACATCTTGGCGGGTTAYGGAGCGGGGGTGGCAGGCGCCCTCGTGGCCTTTAAGGTCATGAGTGGTGAAATGCCTTCCACCGAGGACTTGGTCAATTTGCTCCCTGCCATCCTCTCCCCTGGTGCCCTAGTCGTCGGGGTGGTGTGCGCAGCAATACTGCGTCGGCATGTGGGCCCAGGGGAGGGGGCTGTGCAGTGGATGAACCGGCTGATAGCGTTCGCTTCGCGGGGTAACCACGTCTCCCCCACGCACTATGTGCCCGAGAGCGACGCTGCTGCGCGTGTCACTCAGATCCTCTCCAGCCTTACCATCACTCAGCTGTTGAAGAGGCTCCATCAGTGGATTAACGAGGACTGCTCCACGCCATGT

>HM106770

GCCACACACCTCCCTTACATCGAACAAGGAATGCAGCTCGCCGAACAATTCAAGCAGAAGGCACTCGGGTTGTTGCAGACAGCCACCAAGCAAGCAGAGGCTGCTGCTCCCGTGGTGGAGTCCAAGTGGCGAGCYCTTGAGAGCTTCTGGGTGAAGCATATGTGGAATTTYATCAGCGGGATACAATACTTAGCAGGCTTGTCCACCCTGCCTGGGAACCCCGCAATAGCATCACTRATGGCATTCACAGCYTCCATCACCAGCCCGCTCACCACCCAACACACCCTCCTGTTCAACATYTTGGGGGGATGGGTGGCCGCCCAACTCGCYCCCCCCAGTGCTGCCTCTGCATTCGTRGGCGCCGGCATCGYCGGCGCGGCCGTTGGCAGCATAGGCCTTGGGAAGGTGYTWGTGGACATCTTGGCGGGTTATGGAGCGGGGGTGGCAGGCGCCCTCGTGGCCTTTAAGGTCATGAGTGGTGAGATGCCTTCCACTGAGGACTTGGTCAACTTGCTCCCTGCCATCCTCTCCCCTGGYGCCCTAGTTGTCGGGGTAGTGTGCGCAGCAATACTGCGTCGGCATGTAGGCCCAGGGGAGGGGGCTGTGCAGTGGATGAACCGGCTGATAGCGTTTGCATCGCGGGGYAAYCATGTCTCCCCCACGCACTATGTGCCCGAGAGCGACGCTGCAGCGCGTGTCACYCAGATCCTCTCCRGYCTTACCATCACCCAGCTGCTGAAGAGGCTCCAYCAGTGGATTAACGAGGACTGCTCCACGCCGTGC

>HM106771

GCCACGCATCTCCCTTACATCGAACAAGGAATGCAGCTCGCCGAACAATTYAARCAGAAGGCWCTCGGGTTGTTACAGACRGCCACCAAGCAAGCAGAGGCTGCTGCTCCYGTGGTGGAGTCCAAGTGGCGAGCCCTTGAGAGCTTCTGGGCGAAGCATATGTGGAAYTTCATCAGCGGGATACAATAYTTAGCAGGCTTGTCCACTCTGCCTGGGAACCCCGCAATAGCATCACTGATGTCATTCACAGCCTCTATCACCAGCCCGCTTACCACCCAACACACCCTCCTGTTTAACATCTTGGGGGGATGGGTGGCTGCCCAACTCGCCCCCCCCGGCGCTGCTTCTGCTTTCGTAGGCGCCGGYATYGCCGGCGCGGCCGTTGGCAGCATAGGCCTTGGGAGGGTGCTAGTGGACATCTTGGCGGGTTATGGAGCGGGGGTGGCAGGCGCCCTCGTGGCCTTTAAGGTCATGAGTGGCGAGATGCCTTCCACTGAGGACTTGGTCAACTTGCTCCCTGCCATCCTCTCCCCTGGTGCCCTAGTCGTCGGGGTAGTGTGCGCAGCAATACTRCGCCGGCATGTGGGCCCGGGGGAGGGGGCTGTGCAGTGGATGAACCGGCTGATAGCGTTCGCTTCGCGGGGTAACCACGTCTCCCCCACGCACTATGTGCCCGAGAGCGACGCTGCAGCGCGTGTCACTCAGATTCTCTCCAGCCTTACCATCACTCAGCTGTTGAGAAGGCTCCACCAGTGGATTAACGAGGACTGCTCCACGCCATGC

>HM106772

GCCACACACCTCCCCTACATCGAACAAGGAATGCAGCTCGCCGAACAATTCAAGCAGAAGGCACTCGGGTTGTTACAGACAGCCACTAAGCAAGCAGAGGCTGCTGCTCCCGTGGTGGAGTCCAAGTGGCGAGCCCTTGAGAGCTTCTGGGCGAAGCATATGTGGAATTTCATCAGCGGGATACAATACTTAGCAGGCTTGTCCACTCTGCCTGGGAACCCCGCAATAGCATCACTGATGGCATTCACAGCCTCTATCACCAGCCCGCTCACCACCCAACATACCCTCYTGTTYAACATCYTGGGRGGATGGGTRGCCGCCCAGCTCGCCCCCCCCAGCGCTGCTTCTGCTTTCGTAGGCGCCGGCATCGCCGGCGCGGCCGTTGGCAGCATAGGCCTTGGGAAGGTGCTAKTGGACATCTTGGCGGGTTATGGAGCGGGGGTGGCAGGCGCCCTCGTGGCCTTTAAGGTCATGAGTGGTGAGATGCCTTCCACTGAGGACTTGGTCAATTTGCTCCCTGCCATCCTCTCCCCTGGTGCCCTAGTCGTCGGGGTAGTCTGCGCAGCAATACTGCGTCGGCATGTGGGCCCAGGGGAGGGGGCTGTGCAGTGGATGAACCGGCTGATAGCGTTCGCTTCGCGGGGTAACCACGTCTCCCCCACGCACTATGTGCCCGAGAGCGACGCTGCAGCRCGTGTCACTCAGATCCTCTCCAGCCTTACCATCACACAGCTGCTGAGGAGGCTCCATCAGTGGATTAACGAGGACTGCTCCACGCCATGC

>HM106773

GCCACACACCTCCCTTACATTGAACAAGGAATGCAGCTCGCCGAACAATTCAARCAGAAGGCACTCGGGTTGCTACAGACAGCCACYAAGCAAGCAGARGCTGCTGCTCCCGTGGTGGAGTCYAAGTGGCGAGCCCTTGAGAGCTTCTGGGCGAAGCATATGTGGAATTTCATCAGCGGGATACAATACTTAGCAGGCTTGTCCACTCTGCCTGGGAACCCCGCAATAGCATCACTGATGGCATTCACAGCCTCTGTCACCAGCCCGCTCACMACCCARCATACCCTCCTGTTCAACATCYTGGGGGGATGGGTGGCCGCCCAACTCGCCCCCCCCRGCGCTGCTTCTGCTTTCGTAGGCGCCGGCATCGCCGGCGCGGCCGTTGGCAGCATAGGCCTTGGGAAGGTGCTRGTGGACATYTTGGCGGGTTATGGAGCGGGGGTGGCAGGCGCCCTCGTGGCCTTTAAGGTCATGAGTGGTGAGATGCCTTCCACTGAGGACTTRGTCAACTTGCTCCCTGCCATCCTCTCCCCTGGTGCCCTAGTCGTCGGGGTAGTGTGCGCAGCAATACTGCGTCGGCAYGTGGGCCCAGGGGAGGGGGCTGTGCAGTGGATGAACCGGCTGATAGCGTTCGCTTCGCGGGGTAACCACGTCTCCCCCACGCACTATGTGCCYGAGAGCGACGCTGCAGCGCGTGTCACTCAGATCCTCTCCAGCCTTACCATCACTCAGYTGCTGARGAGGCTCCATCAGTGGATTAACGAGGACTGCTCCACGCCATGC

>HM106774

GCCACACACCTCCCTTACATTGAACAAGGAATGCAGCTCGCCGAACAATTCAAGCAGAAGGCACTCGGGTTGCTACAGACGGCCACCAAGCAAGCAGAGGCTGCTGCTCCCGTGGTGGAGTCCAAGTGNNNNNNNNNNNNNNNNTTTTGGGCGAAGCATATGTGGAATTTCATCAGCGGGATACAATACTTAGCAGGCTTGTCCACTCTGCCTGGGAACCCCGCAATAGCATCACTGATGGCATTCACRGCCTCCATCACCAGCCCGCTCACCACCCAACACACCCTCCTGTTCAACATTCTGGGGGGATGGGTGGCCGCCCAACTCGCCCCCCCCAGCGCTGCTTCCGCTTTCGTAGGCGCCGGCATCGCCGGCGCGGCCGTTGGCAGCATAGGTCTTGGGAAGGTGCTAGTGGACATCTTGGCGGGTTATGGAGCGGGGGTGGCAGGCGCCCTCGTGGCCTTTAAGGTCATGAGTGGTGAGATGCCTTCCACTGAGGATTTGGTCAACTTGCTCCCTGCCATCCTCTCCCCTGGTGCCCTAGTCGTCGGAGTAGTGTGCGCAGCAATACTGCGTCGGCATGTGGRCCCAGGGGAGGGGGCGGTGCAGTGGATGAACCGGCTGATAGCGTTCGCTTCGCGGGGTAACCATGTCTCCCCCACGCACTATGTGCCCGAGAGCGACGCTGCAGCGCGTGTCACTCAGATCCTCTCCAGCCTTACCATCACTCAACTGTTGAAGAGGCTCCATCAGTGGATTAACGAGGACTGCTCCACGCCATGC

>HM106775

NNNNNNNNNNNNNNNNNNNNNNNNNNNNNNNNNNNNNNNNNNNNNNNNNNNNNNNNNNNNNNNNNNNNNNNNNNNNNNNNNNNNNNNNNNNNNNNNNNNNNNNNNNNNNNNNNNNNNNNNNNNNNNNNNNNNNNNNNNNNNNNNNNNNNNNNNNNNNNNNNNNNNNNNNNNNNNNNNNNNNNNNNNNNNNNNNNNNNNNNNNNNNNNNNNNNNNNNNNNNNNNNNNNNNNNNNNNNNNNNNNNNNNNNNNNNNNNNNNNNNNNNNNNNNNNNNNNNNNNNNNACCCTCCTGTTCAACATCTTGGGGGGATGGGTGGCTGCCCAACTTGCCCCCCCCAGCGCTGCTTCTGCTTTCGTAGGCGCCGGCATCGCCGGCGCGGCCGTTGGCAGCATAGGCCTTGGGAAGGTGCTAGTGGACATTTTGGCGGGTTATGGAGCTGGGGTGGCAGGCGCCCTCGTGGCCTTTAAGGTCATGAGTGGCGAGATGCCTTCCACTGAGGACTTGGTCAACTTGCTCCCTGCCATCCTCTCCCCTGGYGCCCTAGTCGTCGGRGTAGTGTGCGCAGCAATACTCCGTCGGCATGTGGGCCCAGGGGAGGGGGCCGTGCAGTGGATGAACCGGCTGATAGCGTTCGCTTCGCGGGGTAACCATGTCTCCCCCACGCACTATGTGCCTGAGAGCGACGCTGCAGCGCGTGTCACTCAGATCCTCTCCAGCCTTACCATCACTCAGCTGCTGAAGAGGCTCCACCAATGGATTAATGAGGACTGCTCCACGCCATGC

>HM106776

NNNNNNNNNNNNNNNNNNNNNNNNNNNNNNNNNNNNNNNNNNNNNNNNNNNNNNNNNNNNNNNNNNNNNNNNNNNNNNNNNNNNNNNNNNNNNNNNNNNNNNNNNNNNNNNNNNNNNNNNNNNNNNNNNNNNNNNNNNNNNNNNNNNNNNNCGAAGCATATGTGGAATTTCATCAGCGGGATACAATACTTAGCAGGCTTGTCCACTCTGCCTGGAAACCCCGCAATAGCATCACTGATGGCATTCACAGCCTCTATCACCAGCCCGCTCACCACCCAGCATACCCTCCTGTTCAACATTTTGGGGGGATGGGTGGCCGCCCAACTCGCCCCCCCCAGCGCCGCTTCTGCTTTCGTAGGCGCCGGCATCGCCGGCGCGGCCGTTGGCAGYATAGGCCTTGGGAAGGTGCTAGTGGACATCTTGGCGGGTTATGGAGCGGGRGTGGCAGGCGCCCTCGTGGCCTTTAAGGTCATGAGTGGTGAGATGCCTTCCACTGAGGACTTGGTCAATTTGCTCCCTGCCATCCTCTCCCCTGGTGCTCTCGTCGTCGGGGTAGTGTGCGCTGCAATACTGCGCCGGCATGTGGGCCCAGGGGARGGGGCTGTGCAGTGGATGAACCGGCTGATAGCGTTCGCGTCGCGGGGTAACCACGTCTCCCCCACGCACTATGTGCCCGAGAGCGACGCTGCAACGCGTGTCACTCAGATCCTCTCCAGCCTTACCATCACTCAGCTGCTGAAGAGGCTCCATCAGTGGATTAACGAGGACTGCTCCACGCCATGC

>HM106777

NNNNNNNNNNNNNNNNNNNNNNNNNNNNNNNNNNNNNNNNNNNNNNNNNNNNNNNNNNNNNNNNNNNNNNNNNNNNNNNNNNNNNNNNNNNNNNNNNAGGCTGCTGCYCCCGTGGTGGAGTCCAAGTGGCGAGCCCTTGAGAGCTTCTGGGCGAAGCATATGTGGAATTTCATCAGCGGGATACAATACTTAGCAGGCTTGTCCACYCTGCCTGGGAAYCCCGCAATAGCATCAYTGATGGCATTCACAGCCTCYATCACCAGCCCGCTCACCACCCAACATACCCTCCTRTTCAACATYTTGGGGGGATGGGTGGCCGCCCAACTCGCCCCCCCCGGCGCYGCTTCTGCTTTCGTAGGCGCCGGCATCGCCGGCGCGGCCGTTGGCAGCATAGGCCTTGGGAAGGTGCTAGTGGAYATCTTGGCGGGYTATGGAGCGGGRGTRGCAGGYGCCCTCGTGGCCTTYAAGGTCATGAGTGGWGAGATGCCTTCCACTGAGGACTTGGTCAACTTGCTCCCTGCCATYCTCTCCCCTGGTGCCCTAGTYGTCGGGGTAGTGTGCGCAGCAATACTGCGTCGGCACGTGGGCCCAGGGGAGGGGGCTGTGCAGTGGATGAACCGGCTGATAGCGTTCGCTTCGCGGGGTAACCACGTCTCCCCCACGCACTATGTGCCCGAGAGCGACGCTGCAGCGCGTGTYACTCAGATCCTCTCTAGCCTTACCATCACTCAGYTGCTGAGGAGGCTCCATCAGTGGATTAAYGAGGACTGCTCCACGCCATGC

>HM106778

NNNNNNNNNNNNNNNNNNNNNNNNNNNNNNNNNNNNNNNNNNNNNNNNNNNNNNNNNNNNNNNNNNNNNNNNNNNNNNNNNNNNACCAAGCAAGCAGAGGCTGCTGCTCCCGTGGTGGAGTCCAAGTGGCGAGCCCTTGAGAGCTTCTGGGCGAAGCATATGTGGAATTTCATCAGCGGGATACAATACTTAGCAGGCTTGTCCACTCTGCCTGGGAACCCCGCAATAGCATCACTGATGGCATTCACAGCCTCTATCACCAGCCCGCTCACCACCCAACATACCCTCCTGTTTAACATCTTGGGGGGATGGGTGGCCGCCCAACTCGCCCCCCCCAGCGCCGCTTCTGCTTTCGTGGGCGCCGGCATCGCCGGCGCGGCCGTTGGCAGTATAGGCCTTGGGAAGGTGCTAGTGGACATTTTGGCGGGCTATGGAGCGGGGGTGGCAGGCGCCCTCGTGGCCTTTAAGGTCATGAGTGGTGAGATGCCTTCTACTGAGGACTTGGTCAACTTGCTCCCTGCCATCCTCTCCCCCGGTGCCATAGTCGTCGGGGTAGTGTGCGCTGCAATACTGCGTCGGCATGTGGGCCCAGGGGAAGGGGCTGTGCAGTGGATGAACCGGCTGATAGCGTTCGCCTCGCGGGGCAACCACGTCTCCCCCACGCACTATGTGCCCGAGAGCGACGCTGCAGCGCGTGTCACCCAGATCCTCTCCAGCCTTACCATCACTCAGCTGCTGAGGAGGCTCCATCAGTGGATTAACGAGGACTGCTCTACGCCATGC

>HM106779

GCCACACACCTCCCTTACATTGAACAAGGAATGCAGCTCGCCGAACAATTCAAGCAGAAGGCNNNNNNNNNNNNNNNNNNNNNNNNNNNNNNNNNNNNNNNNNNNNNNNNNNNNNNNNNNNNNNAGTGGCGAGCCCTTGAGAGCTTCTGGGCAAAGCATATGTGGAATTTCATCAGCGGGATACAGTACTTAGCAGGCTTGTCCACTCTGCCTGGGAACCCCGCAATAGCATCYCTGATGGCATTCACAGCCTCTATCACCAGCCCGCTCACCACCCAACATACCCTCCTGTTCAACATCTTGGGGGGATGGGTGGCCGCCCAACTCGCCCCCCCCAGCGCCGCTTCTGCTTTCGTAGGTGCCGGCATTGCCGGCGCGGCCGTTGGCAGCATAGGCCTTGGGAAGGTGCTAGTAGACATCTTGGCGGGTTATGGAGCGGGGGTGGCAGGCGCCCTCGTGGCCTTTAAGGTCATGAGCGGTGAGATGCCTTCCACTGAGGACTTGGTCAACTTGCTCCCTGCCATCCTCTCCCCTGGTGCCCTAGTCGTCGGGGTAGTGTGCGCAGCAATACTGCGCCGGCATGTGGGCCCAGGGGAGGGGGCTGTGCAGTGGATGAACCGGCTGATAGCGTTCGCTTCGCGGGGTAACCACGTCTCCCCCACSCACTATGTGCCCGAAAGCGACGCTGCAGCGCGTGTCACTCAGATCCTCTCCAGCCTTACCATCACTCAGCTGCTGAAGAGGCTCCATCAGTGGATTAACGAGGACTGCTCCACGCCGTGC

>HM106780

NNNNNNNNNNNNNNNNNNNNNNNNNNNNNNNNNNNNNNNNNNNNNNNNNNNNNNNNNNNNNNNNNNNNNNNNNNNNNNNNNNCCACCAAGCAAGCAGAGGCTGCTGCTCCCGTGGTGGAGTCCAAGTGGCRTGCCCTTGAGAGCTTCTGGGCGAAGCATATGTGGAATTTCATCAGCGGGATACAATACTTAGCAGGCTTGTCCACTCTGCCTGGGAACCCCGCGATAGCATCACTGATGGCATTCACAGCCTCCATCACCAGCCCGTTCACCACCCAACATACCCTCCTGTTCAACATCTTGGGGGGATGGGTGGCCGCCCAACTCGCCCCCCCCAGCGCCGCTTCTGCTTTCGTAGGCGCCGGCATYGCCGGCGCGGCCGTTGGCAGCATAGGCCTTGGGAAGGTGTTAGTGGACATCTTGGCGGGTTATGGGGCGGGGGTGGCAGGCGCCCTCGTGGCCTTTAAAGTCATGAGTGGTGAGATGCCTTCCACTGAGGACTTGGTCAACTTGCTCCCTGCCATCCTCTCCCCTGGTGCCCTAGTTGTCGGGGTAGTGTGCGCAGCAATACTGCGTCGGCATGTGGGCCCAGGGGAGGGGGCTGTGCAGTGGATGAACCGGCTGATAGCGTTCGCYTCGCGGGGTAACCATGTCTCCCCCACGCACTATGTGCCCGAGAGCGACGCTGCAGCGCGTGTCACTCGGATCCTCTCCAGCCTTACCATCACCCAGCTACTGAGGAGGCTCCATCAGTGGATCAACGAGGACTGCTCCACGCCATGC

>HM106781

GCCTCACACCTCCCTTACATYGAACAAGGAATGCAGCTCGCCGAACAATTCAAGCAGAAGGCACTCGGGTTGTTGCAGACRGCCACCAAGCAANNNNNNNNNNNNNNNNNNNNNNNNNNNNNNNNNNNNNNNNNNNNNNNNNNNNNNNNNGCGAAGCATATGTGGAACTTCATCAGCGGGATACAATACTTAGCAGGCTTGTCCACTCTGCCTGGGAACCCCGCAATAGCATCACTGATGGCATTCACAGCCTCTATCACCAGCCCACTCACCACCCAACAYACCCTCCTGTTCAAYATCTTGGGGGGATGGGTGGCCGCCCAGCTCGCCCCTCCCGGCGCTGCTTCTGCTTTCGTAGGCGCCGGCATTGCCGGCGCGGCCGTTGGCAGCATAGGCCTTGGGAAGGTGCTAGTGGACATCTTGGCGGGYTATGGAGCGGGGGTGGCAGGCGCCCTCGTGGCTTTTAAGGTCATGAGYGGTGAGATGCCTTCCACTGAGGACTTGGTCAACTTGCTCCCTGCTATCCTCTCCCCTGGTGCCCTAGTCGTCGGGGTAGTGTGCGCAGCAATACTGCGTCGGCATGTGGGCCCAGGGGAGGGGGCTGTGCAGTGGATGAACCGGCTGATAGCGTTCGCTTCGCGGGGCAACCATGTCTCCCCCACGCACTATGTGCCCGAGAGCGACGCTGCGGCGCGTGTCACTCAGATCCTCTCCAGCCTTACCATCACTCAGCTGCTGAAGAGGCTCCACCAGTGGATTAACGAGGACTGCTCCACACCATGT

>HM106782

NNNNNNNNNNNNNNNNNNNNNNNNNNNNNNNNNNNNNNNNNNNNNNNNNNNNNNNNNNNNNNNNNNNNNNNNNNNNNNNNNNNNNNNNNNNNNNNNNNGGCTGCTGCTCCCGTGGTGGAGTCCAAGTGGCGAGCCCTTGAGAGCTTCTGGGCGAAGCATATGTGGAAYTTCATCAGCGGGATACAATACYTAGCAGGCTTGTCCACTCTGCCTGGGAACCCCGCAATAGCATCACTGATGGCATTCACAGCCTCYATCACCAGCCCGTTCACCACCCAACATACCCTCCTGTTCAACATYTTGGGGGGATGGGTGGCCGCCCAACTCGCCCCTCCCAGCGCYGCTTCTGCTTTCGTAGGCGCCGGCATCGCCGGTGCGGCCGTTGGCAGYATAGGCCTTGGGAAGGTGCTAGTGGACATCTTGGCGGGTTATGGAGCGGGGGTGGCGGGCGCCCTCGTGGCCTTYAAGGTCATGAGTGGTGAGATGCCTTCCACTGAGGACTTGGTCAACTTGCTCCCTGCTATCCTCTCCCCTGGTGCCCTAGTCGTCGGGGTAGTGTGCGCAGCAATACTGCGTCGGCATGTGGGCCCAGGGGAGGGGGCTGTGCAGTGGATGAACCGGCTGATAGCGTTCGCTTCGCGGGGTAACCACGTCTCCCCCACGCACTATGTGCCCGAGAGCGACGCTGCAGCGCGTGTCACTCAGATCCTTTCCAGCCTTACCATCACTCAGCTGCTGAAGAGGCTCCACCAGTGGATTAACGAGGACTGCTCCACGCCATGC

>HM106783

GCTACACACCTCCCTTACATTGAACAAGGAATGCAGCTCGCCGAACAATTCAAGCAGAAAGCACTCGGGTTGTTACAGACAGCCACCANNNNNNNNNNNNNNNNNNNNNNNNNNNNNNNNNNNNNNNNNNNNNNNNNNNNNNNNNNNNNNNNNAAGCATATGTGGAATTTCATCAGCGGGATACAATACTTAGCAGGCTTGTCCACCCTGCCTGGGAACCCCGCAATAGCATCACTGATGGCATTCACAGCCTCCATCACCAGCCCACTCACCACCCAACATACCCTCCTGTTCAACATCTTGGGGGGATGGGTGGCCGCCCAACTCGCCCCCCCCAGCGCTGCTTCTGCTTTCGTAGGCGCTGGCAYCGCCGGCGCGGCCGTTGGCAGCATAGGCCTTGGGAAGGTGCTGGTGGACATCTTGGCGGGTTATGGAGCGGGGGTGGCGGGCGCCCTCGTGGCCTTTAAGGTCATGAGCGGTGAGATGCCTTCCACTGAGGACTTGGTCAACTTGCTCCCTGCCATCCTCTCCCCTGGTGCCCTAGTCGTCGGGGTAGTGTGCGCAGCAATACTGCGTCGGCACGTGGGCCCAGGGGAAGGGGCTGTGCAGTGGATGAACCGGCTGATAGCATTCGCTTCGCGGGGTAACCACGTCTCCCCCACGCACTACGTGCCCGAGAGCGATGCTGCAGCGCGTGTCACTCAGATCCTCTCCAGCCTTACCATCACTCAGCTGTTGAAGAGGCTCCATCAGTGGATTAACGAGGACTGTTCCACGCCATGT

>HM106784

NNNNNNNNNNNNNNNNNNNNNNNNNNNNNNNNNNNNNNNNNNNNNNNNNNNNNNNNNNNNNNNNNNNNNNNNNNNNNNNNNNCCACTAAGCAAGCAGAGGCTGCTGCTCCCGTGGTGGAGTCCAAGTGGCGAGCCCTTGAGAGCTTCTGGGCGAAGCATATGTGGAATTTCATCAGCGGGATACAATACTTAGCAGGCTTGTCCACTCTGCCTGGGAACCCCGCAATAGCATCACTGATGGCATTCACAGCCTCCATCACCAGCCCGCTCACCACCCAACAYACCCTCCTGTTCAACATTTTGGGGGGATGGGTGGCCGCCCAACTCGCCCCCCCCAGCGCCGCTTCTGCTTTCGTAGGCGCCGGCATCGCCGGCGCGGCCGTTGGCAGCATAGGCCTTGGGAAGGTGCTAGTGGACATCTTGGCGGGCTATGGAGCGGGGGTGGCAGGCGCCCTCGTGGCCTTYAAGGTCATGAGTGGTGAGATGCCTTCCACTGAGGACTTGGTCAACTTGCTCCCTGCCATCCTCTCCCCTGGTGCCCTAGTCGTCGGGGTAGTGTGCGCAGCAATACTGCGGCGGCATGTGGGCCCAGGGGAGGGGGCTGTGCAGTGGATGAACCGGCTGATAGCGTTCGCTTCGCGGGGTAACCACGTTTCCCCCACGCACTATGTGCCYGAGAGCGACGCTGCAGCGCGTGTCACTCAGATCCTCTCCAGCCTTACCATCACTCAGCTGTTGAAGAGGCTCCATCAGTGGATCAACGAGGACTGCTCCACGCCATGT

>HM106785

NNNNNNNNNNNNNNNNNNNNNNNNNNNNNNNNNNNNNNNNNNNNNNNNNNNNNNNNNNNNNNNNNNNNNNNNNNNNNNNNNNNNNNNNNNNNNNNNNNNNNNNNNNNNNNNNNNNNNNNNNNNAAGTGGCGAGCCCTTGAGAGCTTCTGGGCGAAGCATATGTGGAATTTCATCAGCGGGATACAATACTTAGCAGGCTTGTCCACTCTGCCTGGGAACCCCGCAATAGCATCACTGATGGCATTCACAGCCTCTATCACCAGCCCGCTCACCACCCARCATACCCTCCTGTTCAACATCTTGGGGGGATGGGTGGCCGCCCAACTCGCCCCCCCCAGCGCTGCTTCTGCTTTCGTAGGCGCCGGCATCGCCGGCGCGGCCGTTGGCAGCATAGGCCTTGGGAAGGTGCTAGTGGACATCTTGGCGGGTTATGGAGCGGGGGTGGCRGGCGCCCTCGTGGCCTTTAARGTCATGAGTGGTGAGATGCCTTCCACTGAGGACTTGGTCAACTTGCTCCCTGCCATCCTCTCCCCTGGTGCCCTAGTCGTCGGAGTAGTGTGCGCAGCAATACTGCGTCGRCATGTGGGCCCAGGGGAGGGGGCCGTGCAGTGGATGAACCGGCTGATAGCGTTTGCATCGCGGGGCAACCATGTCTCCCCCACGCACTATGTGCCCGAGAGCGACGCTGCAGCGCGTGTCACTCAGATCCTCTCCAGCCTTACCATCACTAAGCTGCTGAAGAGGCTCCATCAGTGGATTAATGAGGACTGCTCCACGCCATGT

>HM106786

NNNNNNNNNNNNNNNNNNNNNNNNNNNNNNNNNNNNNNNNNNNNNNNNNNNNNNNNNNNNNNNNNNNNNNNNNNNNNNNNNNNNNNNNNNNNNNNNNNNNNNNNNNNNNNNNNNNNNNNNNNNNNNNNNNNNNNNNNNNNNNNNNNNNNNNNNNNNNNNNNNNNNNNNNNNNNNNNNNNNNNNNNNNNNNNNNNNNNNNNNNNNNNNNNNNNNNNNNNNNNNNNNNNNNNNNNNNNNNNNNNNNNNNNNNNNNNNNNNNNNNNNNNNNNNNNNNNNNNNNNNNNNNNNNNNNNNNNNNNNNNNNNNNNNNNNNNNNNNNNNNNNNNNNNNNNNNNNNNNNNNNNNNNNNNNNNNNNNGGCGCCGGCATCGCCGGCGCGGCCGTTGGCAGCATAGGCCTTGGGARGGTGCTAGTGGACATCTTGGCGGGTTAYGGAGCGGGGGTGGCAGGCGCCCTCGTGGCCTTTAAGGTCATGAGTGGTGAGATGCCTTCCACTGAGGACTTGGTCAACTTGCTCCCTGCCATCCTCTCCCCTGGTGCCCTAGTYGTCGGAGTAGTGTGCGCAGCAATACTGCGTCGGCATGTGGGCCCAGGGGAGGGGGCTGTGCAGTGGATGAACCGGCTGATAGCGTTCGCTTCGMGGGGTAACCACGTCTCCCCCACGCACTATGTGCCCGAGAGCGACGCTGCAGCGCGTGTCACTCAGATCCTCTCCAGCCTTACCATCACTCAGCTGCTGAAGAGGCTCCATCAGTGGATCAACGAGGACTGCTCCACGCNNNNN

>HM106787

NNNNNNNNNNNNNNNNNNNNNNNNNNNNNNNNNNNNNNNNNNNNNNNNNNNNNNNNNNNNNNNNNNNNNNNNNNNNNNNNNNNNNNNNNNNNNNNNNNNNNNNNNNNNNNNNNNNNNNNNNNNNNNNNNNNNNNNNNNNNNNNNNNNNNNNNGAAGCATATGTGGAAYTTCATCAGCGGGATACAATACTTAGCAGGCTTGTCCACTCTGCCTGGGAACCCCGCAATAGCATCACTGATGGCGTTCACAGCCTCYATCACCAGCCCGCTCACCACCCAACATACCCTCCTGTTCAACATTTTGGGGGGATGGGTGGCCGCCCAACTCGCTCCCCCCAGCGCTGCTTCTGCTTTCGTAGGCGCCGGCATCGCCGGMGCGGCCGTTGGCAGYATAGGCCTTGGGAAGGTGCTGGTGGACATCTTGGCGGGTTATGGAGCGGGRGTGGCGGGCGCCCTCGTGGCCTTTAAGGTTATGAGTGGYGAGATGCCTTCCACTGAGGACTTGGTCAACTTGCTCCCTKCCATCCTCTCCCCTGGTGCCCTAGTCGTCGGGGTAGTGTGYGCAGCAATACTGCGTCGGCATGTGGGCCCAGGGGAGGGGGCYGTGCAGTGGATGAACCGGCTGATAGCGTTCGCTTCGMGGGGTAACCACGTCTCCCCCACGCACTATGTGCCYGAGAGCGACGCTGCAGCAYGTGTCACTCAGATCCTCTCCAGCCTTACCATCACTCAGCTGCTGAAGAGGCTCCATCAGTGGATTAACGAGGACTGCTCCACGCCATGC

>HM106788

GCCACACACCTCCCTTACATTGAACAAGGACTACAGCTCGCCGAACAATTCAAGCAGAAGGCACTCGGGTTGCTGCAGACAGCCACCAAGCAAGCAGAGGCTGCTGCTCCCGTGGTGGAGTCCAAGTGGCGAGCCCTTGAGAGCTTCTGGGCGAAGCAYATGTGGAATTTYRTCAGCGGGATACAATACCTAGCAGGCTTGTCCACTCTGCCTGGGAACCCCGCAATAGCATCACTGATGGCATTCACAGCCTCTATCACCAGCCCGCTCACCACCCAACAYACCCTCCTGTTCAACATCTTGGGAGGATGGGTGGCCGCCCAACTCGCCCCCCCCAGCGCTGCTTCTGCCTTCGTAGGCGCCGGCATCGCCGGCGCGGCCGTTGGCAGCATAGGCCTTGGGAAGGTGCTAGTGGACATCTTGGCRGGTTATGGAGCGGGGGTGGCAGGCGCCCTYGTGGCCTTTAAGGTCATGAGCGGTGAGATGCCCTCCACTGAGGACTTGGTCAACTTGCTCCCTGCCATTCTCTCCCCTGGTGCCCTGGTCGTCGGAGTAGTGTGCGCAGCAATACTACGTCGGCATGTGGGCCCAGGGGAGGGGGCTGTGCAGTGGATGAACCGGCTGATAGCGTTCGCGTCGCGGGGTAACCAYGTYTCCCCCACGCACTATGTGCCCGAGAGCGACGCTGCAGCGCGTGTCACTCAAATCCTCTCCAGCCTTACCATCACTCAGCTGYTGAGGAGGCTCCAYCAGTGGATTAACGAGGACTGCTCCACGCCATGC

>HM106789

NNNNNNNNNNNNNNNNNNNNNNNNNNNNNNNNNNNNNNNNNNNNNNNNNNNNNNNNNNNNNNNNNNNNNNNNNNNNNNNNNNNCACCAAGCAAGCAGAGGCTGCTGCTCCCGTGGTGGAGTCCAAGTGGCGAGCCCTTGAGAGCTTCTGGGCGAAGCACATGTGGAATTTCATTAGCGGGATACAATACTTAGCAGGCTTGTCCACTCTGCCTGGGAACCCCGCAATAGCATCACTAATGGCATTTACAGCCTCTATCACCAGCCCGCTCACCACCCAACATACCCTCCTGTTCAACATTTTGGGGGGATGGGTGGCCGCCCAACTCGCCCCCCCCAGCGCTGCTTCTGCTTTCGTAGGCGCTGGCATCGCCGGCGCGGCTGTTGGCAGCATAGGCCTCGGGAAGGTGCTAGTGGACATCTTGGCGGGTTACGGRGCGGGGGTGGCAGGCGCCCTCGTGGCCTTTAAGGTCATGAGTGGTGAGATGCCTTCCACTGAGGACTTAGTCAACTTGCTCCCTGCCATCCTCTCCCCTGGTGCCCTAGTCGTCGGGGTAGTGTGCGCAGCAATACTGCGTCGGCATGTGGGCCCAGGGGAGGGGGCTGTGCAGTGGATGAACCGGCTGATAGCGTTTGCTTCTCGGGGTAACCACGTTTCCCCCACGCACTACGTGCCCGAGAGCGACGCTGCAGCGCGTGTCACTCAGATCCTCTCCAGCCTTACCATCACTCAGCTGCTGAAGAGGCTCCATCAGTGGATTAACGAGGACTGCTCCACACCATGC

>HM106790

GCCACGCACCTCCCTTACATTGAACAAGGAATGCAGCTCGCCGAACAATTCAAGCAGAAGGCGCTCGGGTTGCTACAAACAGCCACCAAGCAAGCAGAGGCTGCTGCTCCCGTGGTGGAGTCCAAGTGGCGAGCCCTTGAGAGCTTCTGGGCGAAGCATATGTGGAATTTCATCAGCGGGATACAATACTTAGCAGGCTTGTCCACTCTGCCTGGGAACCCCGCAATAGCATCACTGATGGCATTCACAGCCTCCATCACCAGCCCGCTCACCACCCAACATACCCTCCTGTTCAACATCTTGGGGGGATGGGTGGCCGCCCAACTCGCCCCCCCCAGCGCTGCTTCTGCCTTCGTAGGCGCCGGCATCGCCGGCGCGGCCGTTGGCAGTATAGGCCTTGGGAAGGTACTGGTGGACATCCTGGCGGGTTATGGAGCGGGGGTGGCAGGCGCCCTCGTGGCCTTTAAGGTCATGAGTGGTGAGATGCCTTCCACTGAGGACTTGGTCAACTTGCTCCCTGCCATCCTCTCCCCTGGTGCCCTGGTCGTCGGAGTAGTGTGCGCAGCAATACTGCGTCGGCATGTGGGCCCAGGGGAGGGGGCTGTGCAGTGGATGAATCGGCTGATAGCGTTCGCTTCGCGGGGTAACCACGTCTCCCCCACGCACTATGTGCCCGAGAGCGACGCTGCAGCGCGTGTCACTCAGATCCTCTCCAGCCTTACCATCACTCAGCTGCTGAAGAGGCTCCATCAGTGGATCAACGAGGACTGCTCCACGCCATGC

>HM106791

NNNNNNNNNNNNNNNNNNNNNNNNNNNNNNNNNNNNNNCGCCGAACAATTCAAGCAGAAGGCACTCGGGTTGCTACAGACAGCCACCAAGCAAGCAGAAGCTGCTGCTCCCGTGGTGGAGTCCAAGTGGCGAGCCCTTGAGAGCTTCTGGGCGAAGCATATGTGGAATTTCRTCAGCGGGATACAATACCTAGCAGGCTTGTCCACTCTGCCTGGCAACCCCGCAATAGCATCACTGATGGCATTCACAGCCTCCATCACCAGCCCGCTCACCACCCAACATACCCTCCTGTTCAACATCCTGGGAGGATGGGTGGCCGCCCAACTCGCCCCCCCCAGCGCCGCTTCTGCTTTCGTAGGCGCCGGCATCGCCGGCGCGGCCGTTGGCAGCATAGGCCTTGGGAAGGTGTTAGTGGACATYTTGGCGGGTTATGGAGCGGGGGTGGCAGGCGCCCTCGTGGCCTTTAAGGTCATGAGTGGTGAGATGCCTTCCACTGAGGACTTGGTCAACTTGCTYCCTGCCATCCTCTCCCCTGGTGCCCTAGTTGTCGGGGTAGTSTGCGCAGCAATACTGCGTCGGCACGTGGGCCCAGGGGAGGGGGCTGTGCAGTGGATGAACCGGCTGATAGCGTTCGCTTCGCGGGGTAACCACGTTTCCCCCACGCACTATGTGCCCGAGAGCGACGCTGCAGCGCGTGTCACTCAGATCCTCTCCAGCCTTACCATCACTCAGCTGTTGAAGAGGCTCCATCAGTGGATCAACGAGGACTGCTCCACGCCATGC

>HM106792

GCCACACAYCTCCCTTACATYGARCAAGGAATGCAGCTCGCCGAACAATTCAAGCAGAAGGCACTCGGGTTNNNNNNNNNNNNNNNNNNNNNNNNNNNNNNNNNNNNNNNNNNNNNNNNNNNNNNNTGGCGAGCCCTTGAGAGCTTCTGGGCGAAGCATATGTGGAATTTCATCAGCGGGATACAATACTTAGCAGGCTTGTCCACTCTGCCTGGGAACCCCGCAATAGCATCACTGATGGCATTCACAGCCTCCATCACCAGCCCGTTCACCACCCAACACACCCTCCTGTTCAACATTCTGGGGGGATGGGTGGCCGCCCAACTCGCCCCCCCCAGCGCTGCTTCTGCTTTCGTAGGCGCCGGCATTGCCGGTGCGGCCGTTGGCAGCATAGGCCTTGGGAAGGTGCTAGTGGACATCTTGGCGGGTTATGGAGCGGGGGTGGCAGGCGCCCTCGTGGCCTTTAAGGTCATGAGTGGTGTGATGCCTTCCACTGAGGACTTGGTCAACTTGCTCCCTGCCATCCTCTCCCCTGGTGCCCTAGTCGTCGGGGTAGTGTGCGCAGCAATACTGCGYCGGCATGTGGGCCCAGGGGAGGGGGCTGTGCAGTGGATGAACCGGCTGATAGCGTTCGCTTCGCGGGGTAACCAYGTCTCCCCCACGCACTATGTGCCCGARAGCGACGCTGCAGCKYGTGTCACTCAGATCCTCTCYAGCCYWACCATCACTCAGCTGCTGAGGAGGCTCCACCAGTGGATCAACGAGGACTGCTCCACGCCATGC

>HM106793

NNNNNNNNNNNNNNNNNNNNNNNNNNNNNNNNNNNNNNNNNNNNNNNNNNNNNNNNNNNNNNNNNNNNNNNNNNNNNNNNNNNNNNNNNNNNNNNNNNNNNNNNNNNNNNNNNNNNNNNNNNNNNNNNNNNNNNNNNNNNNNNNNNNNNNNNNAAGCATATGTGGAATTTCATCAGCGGGATACAATACTTAGCAGGCTTGTCCACTCTGCCTGGGAACCCCGCAATAGCATCACTGATGGCATTCACAGCCTCTATCACCAGCCCGCTTACCACCCAACATACCCTCCTGTTCAACATCTTGGGGGGATGGGTGGCCGCCCAACTTGCCCCCCCCAGCGCTGCTTCTGCTTTCGTAGGCGCCGGCATCGCCGGCGCGGCCGTTGGCAGCATAGGCCTTGGGAAGGTGCTAGTGGACATCTTGGCGGGTTATGGAGCGGGGGTGGCAGGCGCCCTCGTGGCCTTTAAGGTCATGAGTGGTGAGATGCCTTCCACTGAGGACTTGGTCAACTTGCTCCCTGCCATCCTCTCCCCTGGTGCCCTAGTCGTCGGGGTAGTGTGCGCAGCAATACTGCGCCGGCATGTGGGCCCAGGGGAGGGGGCTGTGCAGTGGATGAACCGGCTGATAGCGTTCGCTTCGCGGGGTAACCACGTTTCCCCCACGCACTATGTGCCCGAGAGCGACGCTGCAGCGCGTGTCACTCAGATCCTCTCCAGCCTTACCATCACTCAGCTGCTGAAGAGGCTCCATCAGTGGATTAATGAGGACTGCTCCACGCCATGC

>HM106794

NNNNNNNNNNNNNNNNNNNNNNNNNNNNNNNNNNNNNNNNNNNNNNNNNNNNNNNNNNNNNNNNNNNNNNNNNNNNNNNNNNNNNNNNNNNNNNNNNNNNNNNNNNNNNNNNNNNNNNNNNNNNNNNNNNNNNNNNNNNNNNNNNNNNNNNNNNNNNNNNTGTGGAATTTCATCAGCGGGATACAATACTTRGCAGGCTTGTCCACTYTGCCTGGSAACCCCGCAATAGCATCACTGATGGCATTCACAGCCTCTATCACCAGCCCGCTCACCACCCAACATACCCTCCTGTTCAACATYTTGGGGGGATGGGTGGCCGCYCAACTCGCCCCCCCCAGCGCYGCTTCTGCTTTCGTAGGCGCCGGCATCGCCGGCGCGGCCGTTGGCAGYATAGGCCTTGGGAAGGTGYTRGTGGACATCTTGGCGGGTTATGGAGCGGGGGTGGCAGGCGCCCTCGTGGCCTTTAAGGTCATGAGTGGTGAGATGCCTTCCACYGAGGACTTGGTCAACTTGCTCCCTGCCATCCTCTCCCCTGGTGCCCTAGTCGTYGGGGTAGTGTGCGCAGCAATACTRCGYCGGCATGTRGGCCCAGGRGAGGGGGCTGTGCAGTGGATGAACCGRCTGATAGCGTTCGCTTCGCGGGGTAACCACGTCTCCCCYACGCACTATGTGCCCGAGAGCGACGCTGCAGCCCGTGTCACTCAGATCCTCTCCAGCCTTACCATCACTCAGCTGCTGAAGAGGCTCCATCAGTGGATCAAYGAGGACTGTTCCACKCCATGC

>HM106795

GCCACACACCTCCCTTACATTGAACAAGGAATGCAGCTCGCCGAACAATTCAAGCAGAAGGCACTCGGGTTGTTACAGACAGCCACCAAGCAAGCAGAGGCTGCTGCTCCCGTGGTGGAGTCCAAGTGGCGAGCCCTTGAGAGCTTCTGGGCGAAGCATATGTGGAAYTTCATCAGCGGGATACAATACTTAGCAGGCTTGTCCACTCTGCCTGGGAACCCCGCAATAGCATCACTGATGGCATTCACAGCCTCTATCACCAGCCCGCTCACCACCCAACACACCCTCCTGTTCAACATTTTGGGGGGATGGGTGGCCGCCCAACTCGCCCCCCCCAGCGCTGCTTCTGCTTTCGTAGGCGCCGGCATCGCCGGCGCGGCCGTTGGCAGCATAGGCCTGGGGAAGGTGCTAGTGGACATCTTGGCGGGTTATGGAGCAGGAGTGGCAGGCGCCCTCGTGGCCTTTAAGGTCATGAGTGGTGAGRTGCCCTCCACTGAGGACTTGGTCAACTTGCTCCCTGCCATCCTCTCCCCTGGCGCCCTAGTCGTCGGGGTAGTGTGCGCAGCAATACTGCGCCGGCATGTGGGCCCGGGGGAGGGGGCTGTGCAGTGGATGAACCGGCTGATAGCGTTCGCTTCGCGGGGTAACCACGTTTCCCCCACGCACTATGTGCCCGAGAGCGACGCTGCAGCGCGTGTCACTCAGATCCTCTCCAGCCTTACCATCACTCAGCTGCTGAAGAGGCTCCATCAGTGGATTAACGAGGACTGCTCCACGCCATGC

>HM106796

GCCACACACCTTCCTTACATTGAACAAGGAATGCAGCTCGCCGAACAATTCAAGCANNNNNNNNNNNNNNNNNNNNNNNNNNNNNNNNNNNNNNNNNNNNNNNNNNNNNNNNNNNNNNNNNNNNNNNNNNNNNNNNNNNNNNNNNNNNNNNNNNNNNNNNNNNNNNNNNNNNNNNNNNNNNNNNNNNNNNNNNNNNNNNNNNNNNNNNNNNNNNNNNNNNNNNNNNNNNNNNNNNNNNNNNNNNNNNNNNNNNNNNTCACCAGCCCGCTCACCACCCAACATACCCTTCTGTTYAACATCTTGGGGGGATGGGTGGCCGCCCAACTCGCCCCCCCCAGCGCTGCTTCTGCTTTCGTGGGCGCCGGCATCGCCGGCGCGGCCGTTGGCAGCATAGGCCTTGGGAAGGTGTTAGTGGACATCTTGGCGGGTTATGGAGCGGGGGTGGCAGGTGCCCTTGTGGCCTTTAAGGTCATGAGTGGTGAGATGCCTTCCACTGAGGACTTGGTCAACTTGCTCCCTGCCATCCTCTCCCCTGGTGCCCTAGTYGTTGGGGTGGTGTGCGCAGCGATACTGCGTCGGCATGTGGGCCCAGGGGAGGGGGCTGTGCAGTGGATGAACCGGCTGATAGCGNNNNNNNNNNNNNNNNNNNNNNNNNNNNNNNNNNNNNNNNNNNNNNNNNNNNNNNNNNNNNNNNNNNNNNNNNNNNNNNNNNNNNNNNNNNNNNNNNNNNNNNNNNNNNNNNNNNNNNNNNNNNNNNNNNNNNNNNNNNNNNNNNNNNNNNNN

>HM106797

GCAACACACCTCCCTTACATTGAACAAGGAATGCAACTCGCCGAACAATTCAAGCAGAAGGCACTCGGGTTGTTGCAGACAGCCACCAAGCAAGCAGAGGCTGCTGCTCCCGTGGTGGAGTCCAAGTGGCGAGCCCTTGAGAGTTTCTGGGCGAAGCATATGTGGAATTTCATCAGCGGGATACAATACTTGGCAGGCTTGTCCACTCTGCCTGGGAACCCCGCAATAGCATCACTGATGGCATTTACAGCCTCCATCACCAGCCCGCTCACCACCCAGCATACCCTCCTGTTCAACATCTTGGGGGGATGGGTGGCCGCCCAACTCGCCCCCCCCAGCGCTGCTTCTGCTTTCGTAGGCGCCGGCATCGCCGGCGCGGCCGTTGGCAGCATAGGCCTTGGGAAGGTGCTAGTGGACATCTTGGCGGGTTATGGAGCGGGGGTAGCAGGCGCCCTCGTGGCCTTTAAGGTCATGAGTGGTGAGATGCCCTCCACTGAGGACTTGGTCAACTTGCTCCCTGCCATCCTCTCCCCTGGTGCCCTAGTCGTCGGGGTAGTGTGCGCAGCAATACTGCGTCGGCACGTGGGTCCAGGGGAGGGGGCTGTGCAGTGGATGAATCGGCTGATAGCGTTCGCTTCGCGGGGCAACCATGTCTCCCCCACGCACTATGTGCCCGAGAGCGACGCCGCAGCGCGTGTCACTCAGATCCTCTCCAGCCTTACCATCACTCAGCTGCTGAAGAGGCTCCATCAGTGGATCAACGAGGACTGCTCCACGCCATGC

>HM106798

NNNNNNNNNNNNNNNNNNNNNNNNNNNNNNNNNNNNNNNNNNNNNNNNNNNNNNNNNNNNNNNNNNNNNNNNNNNNNNNNNNNNNNNNNNNNNNNNNNNNNNNNNNNNNNNNNNNNNNNNNNNNNNNNNNNNNNNNNNNNNNNNNNNNNNNNNNNNNNNNNNNNNNNNNNNNNNNNNNNNNNNNNNNNNNNNNNNNNNNNNNNNNNNNNNNNNNNNNNNNNNNNNNNNNNNNNNNNNNNNNNNNNNNNNNNNNNNNNNNNNNNNNNNNNNNNNNNNNNNNNNNNNNNNNNNNNNNNNNNNNNNNNNNNNNNNNNNNNNNNNNNNNNNNNNNNNNNNNNNNNNNNNNNNGCTTTCGTAGGCGCCGGTATTGCCGGCGCGGCCGTTGGCAGCATAGGYCTTGGGAAGGTGCTAATGGACATCTTGGCRGGTTATGGAGCGGGRGTRGCAGGCGCCCTCGTGGCCTTTAAGGTCATGAGTGGTGAGATGCCTTCCACTGAGGACTTGGTCAACTTGCTCCCTGCCATCCTCTCCCCTGGTGCCCTGGTCGTCGGAGTAGTGTGCGCAGCAATACTGCGTCGGCATGTGGGCCCAGGGGAGGGGGCTGTGCAGTGGATGAACCGGCTGATAGCGTTCGCTTCGCGGGGCAACCATGTTTCCCCTACGCACTACGTGCCCGAGAGCGACGCTGCAGCGCGTGTCACTCAGATCCTCTCCAGCCTTACCATCACTCAGCTGCTGAGGAGGCTCCATCAGTGGATTAACGAGGACTGCTCCACGCCATGC

>HM106799

NNNNNNNNNNNNNNNNNNNNNNNNNNNNNNNNNNNNNNNNNNNNNNNNNNNNNNNNNNNNNNNNNNNNNNNNNNNNNNNNNNCCACCAAGCAAGCAGAGGCTGCTGCTCCCGTGGTGGAGTCCAAGTGGCGAGCCCTTGAGAGCTTCTGGGCGAAGCACATGTGGAATTTCATCAGCGGGATACAATACTTAGCAGGCTTGTCCACTCTGCCTGGGAACCCCGCAATAGCATCACTGATGGCATTCACAGCCTCTATCACCAGCCCGCTCACCACCCAGCATACCCTCCTGTTCAACATYTTGGGGGGATGGGTGGCCGCCCAACTCGCCCCYCCCAGCGCCGCTTCTGCTTTCGTAGGCGCCGGCATCGCCGGTGCGGCCGTTGGCAGCATAGGCCTTGGGAAGGTGCTAGTGGACATCTTGGCAGGTTATGGAGCGGGGGTGGCAGGCGCCCTCGTGGCCTTTAAGGTCATGAGTGGCGAGATGCCTTCTACTGAGGACTTGGTCAACTTGCTCCCTGCCATCCTCTCCCCTGGTGCCCTAGTCGTCGGRGTAGTGTGCGCAGCAATACTGCGTCGGCATGTGGGCCCTGGGGAGGGGGCTGTGCAGTGGATGAACCGGCTGATAGCGTTCGCTTCGCGGGGTAACCACGTCTCCCCCACGCACTATGTGCCTGAGAGCGATGCYGCAGCGCGYGTCACTCAGATCCTCTCTAGCCTTACCATCACTCAGCTGCTGAAGAGGCTCCATCAGTGGATTAAYGAGGACTGCTCCACGCCATGT

>HM106800

NNNNNNNNNNNNNNNNNNNNNNNNNNNNNNNNNNNNNNNNNNNNNNNNNNNNNNNNNNNNNNNNNNNNNNNNNNNNNNNNNNNNNNNNNNNNNNNNNNNNNNNNNNNNNNNNNNNNNNNNNNNNNNNNNNNNNNNNNNNNNNNNNNNNNNNNNNNNNNNNNNNNNNNNNNNNNNNNNNNNNNNNNNNNNNNNNNNNNNNNNNNNNNNNNNNNNNNNNNNNNNNNNNNNNNNNNNNNNNNNNNNNNNNNNNNNNNNNNNNNNNNNNNNNNNNNNNNNNNNNNNNNNNNNNNNNNNNNNATCCTGGGGGGATGGGTGGCCGCCCAACTCGCCCCCCCCAGCGCTGCTTCTGCTTTCGTAGGCGCCGGCATCGCCGGCGCGGCCGTTGGCAGCATAGGCCTTGGGAGGGTGCTAGTGGACATCTTGGCGGGTTATGGAGCGGGGGTGGCAGGCGCCCTCGTGGCCTTTAAGGTCATGAGTGGTGAGATGCCCTCCACTGAGGACTTGGTCAACTTGCTCCCTGCCATCCTCTCCCCTGGTGCCCTAGTCGTAGGGGTAGTGTGCGCAGCAATACTGCGYCGGCATGTGGGCCCAGGGGAGGGGGCTGTGCAGTGGATGAACCGGCTGATAGCGTTCGCTTCGCGGGGTAACCACGTCTCCCCCACGCACTATGTGCCCGAAAGCGACGCTGCAGCGCGTGTCACTCAGATCCTCTCCAGCCTTACCATCACTCAGCTGCTGAAGAGGCTCCATCAGTGGATTAACGAGGACTGCTCCACGCCATGC

>HM106801

GCCACRCACCTCCCTTACATTGAACAAGGAATGCAGCTCGCCGAACAATTCAAGCAGAAGGCACTCGGGTTGTTACARACAGCCACCAAGCAAGCAGAGGCTGCTGCTCCCGTGGTGGAGTCCAAGTGGCGAGCCCTTGAGAGCTTCTGGGCGAAGCATATGTGGAATTTCATCAGCGGGATACAGTACTTAGCAGGCTTGTCCACTCTGCCTGGGAACCCCGCAATAGCATCACTGATGGCATTCACAGCCTCCATCACCAGCCCGCTCACCACCCAACACACCCTCCTGTTCAACATTTTGGGGGGATGGGTGGCCGCCCAACTCGCCCCCCCCAGCGCCGCTTCTGCTTTCGTAGGCGCCGGCATCGCCGGCGCGGCCGTTGGCAGCATAGGCCTTGGGAAGGTGCTAGTGGACATCTTGGCGGGTTATGGAGCGGGGGTGGCAGGCGCCCTCGTGGCCTTTAAGGTCATGAGTGGTGAGATGCCTTCCACTGAGGACTTGGTCAACTTGCTCCCTGCCATCCTCTCTCCTGGTGCCCTAGTCGTCGGGGTAGTGTGCGCAGCAATACTGCGCCGGCATGTGGGCCCAGGGGAGGGGGCTGTGCAGTGGATGAACCGGCTGATAGCGTTCGCTTCGCGGGGTAACCACGTTTCCCCCACGCACTATGTGCCCGAGAGCGACGCTGCAGCGCGTGTCACTCAGATCCTCTCCAGCCTTACCATCACTCAGCTGCTGAAGAGGCTCCACCAGTGGATTAACGAGGACTGCTCCACGCCATGC

>HM106802

NNNNNNNNNNNNNNNNNNNNNNNNNNNNNNNNNNNNNNNNNNNNNNNNNNNNNNNNNNNNNNNNNNNNNNNNNNNNNNNNNGCCACCAAGCAAGCAGAGGCTGCTGCTCCCGTGGTGGAGTCCAAGTGGCGAGCCCTTGAGAGCTTCTGGGCGAAGCATATGTGGAATTTCATCAGCGGGATACAATACTTAGCAGGCTTGTCCACTCTGCCTGGRAACCCCGCAATAGCATCACTGATGGCTTTCACAGCCTCCATCACYAGCCCGCTCACCACCCAACATACCCTCCTGTTCAACATCCTGGGGGGATGGGTGGCCGCCCAACTCGCCCCCCCCAGCGCTGCTTCTGCTTTCGTAGGCGCCGGCATCGCCGGCGCGGCCGTTGGCAGYATARGCCTTGGGAAGGTGYTAGTGGACATCTTGGCGGGCTATGGAGCGGGGGTGGCAGGCGCCCTCGTGGCCTTTAAGGTCATGAGYGGTGAGATGCCTTCCACTGAGGACTTGGTCAACTTGCTCCCTGCCATCCTCTCCCCTGGTGCCCTAGTCGTCGGGGTAGTGTGCGCAGCAATACTGCGTCGGCATGTGGGCCCAGGRGAGGGGGCTGTGCAGTGGATGAACCGGCTGATAGCGTTCGCTTCGCGGGGTAACCACGTCTCCCCCACGCACTATGTGCCYGAGAGCGACGCTGCAACGCGTGTCACTCAGATCCTATCCAGCCTTACCATCACTCAGTTGCTGAAGAGGCTCCATCAGTGGATTAATGAGGACTGCTCCACGCCATGC

>HM106803

NNNNNNNNNNNNNNNNNNNNNNNNNNNNNNNNNNNNNNNNNNNNNNNNNNNNNNNNNNNNNNNNNNNNNNNNNNNNNNNNNGCCACCAAGCAAGCAGAGGCTGCTGCTCCCGTGGTGGAGTCCAAGTGGCGAGCCCTTGAGAGCTTCTGGGCGAAGCATATGTGGAATTTCATCAGCGGGATACAATACTTGGCAGGCTTGTCCACTCTGCCTGGGAACCCCGCAATAGCATCACTGATGGCATTCACAGCCTCTATCACCAGCCCGCTCACCACCCAACATACCCTCCTGTTCAACATCTTGGGGGGATGGGTGGCCGCCCAACTCGCCCCCCCCAGCGCCGCTTCTGCTTTCGTAGGCGCCGGCATCGCCGGTGCGGCCGTTGGCAGCATAGGCCTTGGGAAGGTGCTAGTGGACATCTTGGCGGGTTATGGAGCGGGGGTGGCAGGCGCCCTCGTGGCCTTTAAGGTCATGAGTGGTGAGATGCCTTCCACTGAGGACTTGGTYAACTTGCTCCCTGCCATCCTCTCCCCTGGTGCCCTAGTTGTTGGGGTAGTGTGCGCAGCAATACTGCGTCGGCACGTGGGCCCAGGGGAGGGGGCTGTGCAGTGGATGAACCGGCTGATAGCGTTCGCTTCGCGGGGTAACCACGTCTCCCCCACGCACTATGTGCCCGAGAGCGACGCTGCAGCAYGTGTCACTCAGATCCTCTCCAGCCTTACCATCACTCAGTTGCTGAAGAGGCTCCACCAGTGGATTAACGAGGACTGCTCCACGCCGTGC

>HM106804

NNNNNNNNNNNNNNNNNNNNNNNNNNNNNNNNNNNNNNNNNNNNNNNNNNNNNNNNNNNNNNNNNNNNNNNNNNNNNNNNAGCCACCAAGCAAGCAGAGGCTGCTGCTCCCGTGGTGGAGTCYAAGTGGCGAGCCCTTGAGAGCTTCTGGGCGAAGCATATGTGGAATTTCATCAGCGGGATACAATACTTAGCAGGCTTGTCCACTCTGCCYGGGAACCCCGCGATAGCATCRCTGATGGCATTCACAGCCTCTATCACCAGCCCGCTCACCACCCAACATACCCTYCTGTTCAACATCTTGGGGGGATGGGTGGCCGCCCAACTCGCCCCCCCCAGCGCTGCTTCTGCTTTCGTAGGCGCCGGCATCGCCGGCGCGGCCGTTGGCAGCATAGGCCTTGGGAAGGTGCTAGTGGACATCTTGGCGGGTTATGGMGCGGGGGTGGCRGGCGCYCTCGTGGCCTTTAAGGTCATGAGTGGTGAGATGCCYTCCACTGAAGACTTGGTCAACTTGCTCCCTGCCATCCTCTCCCCYGGTGCCYTAGTCGTCGGGGTAGTGTGCGCAGCAATACTGCGTCGGCATGTGGGCCCAGGGGAGGGGGCTGTGCAGTGGATGAAYCGGCTGATAGCRTTCGCTTCGCGGGGTAACCACGTCTCCCCCACGCACTATGTGCCCGAAAGCGACGCTGCAGCGCGTGTCACTCAGATCCTTTCCAGCCTTACCATCACYCAGCTGTTGAAGAGGCTCCATCAGTGGATTAACGAGGACTGCTCCACRCCATGC
