# Supplementary material for: Effect of Immune Pressure on Hepatitis C Virus Evolution: Insights From a Single-Source Outbreak
Source: Hepatology. 2011 Feb;53(2):396–405. doi: 10.1002/hep.24076 (PMC3044208; doi:10.1002/hep.24076)
Supplement: Supplementary file 9 [file hep0053-0396-SD9.doc]

>HM106805

TCCGGCTCGTGGCTAAGGGACGTTTGGGACTGGATATGCACGGTGTTAACTGATTTTAAGACCTGGCTCCAGTCCAAGCTCCTGCCGCGGTTACCGGGAGTYCCCTTCCTCTCATGYCAACGTGGGTACAAGGGCATTTGGCGGGGAGACGGCATCATGCAAACCACCTGCCCATGTGGAGCRCAGATCWCCGGACATGTCAAAAACGGTTCCATGAGGATCGCTGGGCCTAGGACCTGCAGCAACACGTGGCATGGAACATTCCCCATCAACGCATACACYACGGGCCCCTGCACGCCYTCCCCGGCRCCCAATTATTCCAGGGCGCTGTGGAGGGTGGCTGCTGAGGAGTACGTGGAGGTTACGCGGGTGGGGGATTTCCACTACGTGACGGGCATGACCACTGAYAACGTAAARTGCCCRTGCCAGGTTCCGGCCCCCGAATTCTTCACGGAGGTGGATGGGGTGCRGCTGCACAGGTAYGCTCCRCCGWGCAAACCYCTCCTACGGGAGGAGGTCACATTCCAGGTCGGGCTCAACCAATACCTGGTCGGGTCACAGCTCCCATGYGAGCCCGAACCGGATGTARCGGTGCTCACTTCCATGCTCACCGACCCCTCCCACATCACAGCAGAGRCGGCTAAGCGTAGGCTGGCCAGGGGGTCTCCCCCCTCCTTAGCCAGCTCTTCAGCTAGCCAGTTGTCTGCGCCTTCCTTGAAGGCGACATGCACTACCCGTCATGAYTCCCCAGATGCTGACCTCATCGAGGCCAATCTCCTGTGGCGGCAGGAGATGGGCGGRAACATCACCCGCGTGGAATCAGAGAACAAGGTAGTAATTCTGGACTCTTTCGAGCCGCTTCRAGCGGAGGAAGATGAGAGGGAAGTATCYGTTCCGGCGGAAATCCTGCGGAGATCCAGGAAATTYCCCCGAGCRATGCCCATATGGGCAAGGCCGGATTAYAACCCTCCACTGGTRGAGYCCTGGAAGGAYCCGGACTAYGTCCCTCCGGTGGTACATGGGTGCCCATTGCCACCTACTAAGGCCCCTCCAATACCACCTCCACGGAGGAARAGGACGATYGTCCTGACAGARTCCACCGTGTCTTCTGCCTTGGCTGAGCTCGCCACAAAGACCTTCGGCAGCTCCGAATCGTCGGCCGYCGACAGCGGCACGGCGACYGGCYCTCCWGAYCAGCYCTCCGACGACGGTGACGCAGGGTCCGATGTTGAGTCGTACTCCTCCATGCCCCCCCTTGAGGGGGAGCCGGGGGATCCCGATCTCAGCGACGGGTCTTGGTCTACCGTGAGCGAGGAAGCTAGTGAGGACGTCGTCTGCTGY

>HM106806

TCCGGCTCGTGGCTAAGGGACGTTTGGGACTGGATATGCACGGTATTAAGTGATTTTAAGACCTGGCTCCAGTCCAAGCTCCTGCCGCGGTTACCGGGAGTCCCCTTCCTCTCATGTCAACGTGGGTACAAGGGCGTTTGGCGGGGAGACGGCATCATGCAAACCACCTGCCAATGTGGAGCGCAAATCACCGGACATGTCAAAAACGGTTCCATGAGGATCKTTGGGCCTAGGACCTGCAGCAACACGTGGCATGGAACATTCCCCATCAACGCGTACACCACGGGCCCCTGCACGCCCTCCCCGGCGCCCAAYTATTCCAGGGCGCTGTGGAGGGTGGCTGCTGAGGAGTACGTGGAGGTTACGCGGGTGGGGGATTTCCACTACGTGACGGGCATGACCACTGACAACGTAAAGTGCCCATGCCAGGTTCCGGCCCCCGAATTCTTCACGGAGGTGGATGGGGTACGGCTGCACAGGTAYGCTCCGCCGTGCAARCCTCTCCTACGGGAGGAGGTCACATTCCRGGTCGGGCTCAACCAATACCTGGTCGGGTCACAGCTCCCGTGTGAGCCCGAGCCGGATGTAGCGGTGCTCACTTCCATGCTCACCGACCCCTCCCACATTACAGCAGAGACGGCTAAGCGTAGGCTGGCCAGGGGGTCTCCCCCCTCCTTGGCCAGCTCTTCAGCTAGCCAGTTGTCTGCGCCTTCCTTGAAGGCAACATGCACTACCCATCATGACTCCCCAGATGCTGACCTCATCGAGGCCAATCTCCTGTGGCGGCAGGAGATGGGCGGGAACATCACCCGTGTGGAGTCAGAGAACAAAGTAGTGATTCTGGACTCTTTCGAGCCGCTTCGAGCGGAGGAAGATGAGAGGGAAGTATCCGTTCCGGCGGAAATCCTGCGGAAATCCAGGAAATTYCCCCGAGCGATGCCCATATGGGCAAGGCCGGATTACAACCCYCCACTGMTAGAGTCCTGGAAGGACCCGGACTACGTCCCTCCGGTGGTACATGGGTGCCCATTGCCACCTACTAAGGCCCCTCCAATACCACCTCCACGGAGGAAGAGGACGGTTGTCCTGACAGAATCCACCGTGTCTTCTGCCTTGGCTGAGCTCGCCACAAAGACCTTCGGCAGCTCCGAATCGTCGRCCGTCGACAGCGGCACGGCGACCGCCCCTCCTGACCAGCCCTCCGACGACGGCNNNNNNNNNNNNNNNNNNNNNNNNNNNNNNNNNNNNNNNNNNNNNNNNNNNNNNNNNNNNNNNNNNNNNNNNNNNNNNNNNNNNNNNNNNNNNNNNNNNNNNNNNNNNNNNNNNNNNNNNNNNNNNNNN

>HM106807

TCCGGYTCGTGGCTAAGGGACGTTTGGGACTGGATATGCACGGTGTTAACTGACTTTAAGACCTGGCTCCAGTCYAAGCTCCTGCCGCGGTTACCGGGAGTCCCCTTCCTCTCATGCCAACGTGGATACAAGGGCATCTGGCGGGGAGACGGCATCATGCAAACCACCTGCCCATGTGGAGCGCAGATCACCGGACATGTCAAAAACGGTTCCATGAGGATCGTTGGGCCTAGGACCTGCAGCAACACGTGGCATGGAACATTCCCCATCAACGCGTACACCACGGGCCCCTGCACGCCCTCCCCKGCGCCCAATTATTCCAGGGCGCTGTGGAGGGTAGCTGCTGAGGAGTACGTGGAGGTYACGCGGGTGGGGGATTTCCACTACGTGACGGGCATGACCACTGACAACGTAAAGTGCCCATGTCAGGTTCCGACTCCCGAATTCTTCACGGAGGTGGATGGGGTGCGGCTGCACAGGTACGCTCCGCCGTGCAAACCTCTCCTACGGGAGGAGGTCACGTTCCAGGTCGGGCTCAACCAATACCTGGTCGGGTCACAGCTCCCATGTGAGCCCGAACCGGATGTAGCGGTGCTYACTTCCATGCTCACCGACCCCTCCCACATCACAGCAGAGGCGGCTAAGCGTAGGCTGGCCAGGGGGTCTCCCCCCTCCTTAGCCAGCTCTTCGGCTAGCCAGTTGTCTGCGCCTTCCTTGAAGGCGACATGCACTACCCGTCATGACTCCCCAGATGCTGACCTCATCGAGGCAAATCTCCTGTGGCGGCAGGAGATGGGCGGGAACATCACCCGCGTGGAGTCAGAGAAYAAGGTAGTGATTCTGGACTCTTTCGAGCCGCTTCGAGCGGAGGAAGATGAGAGGGAATTATCCGTTCCGGCGGAAATCCTGCNNNNNNNNNNNNNNNNNNNNNNNNNNNNNNNNNNNNNNNNNNNNNNNNNNNNNNNNNNNNNNNNNNNNNNNNNNNNNNNNNNNNNNNNNNNNNNNNNNNNNNNNNNNNNNNNNNNNNNNNNNNNNNNNNNNNNNNNNNNNNNNNNNNNNNNNNNNNNNNNNNNNNNNNNNNNNNNNNNNNNNNNNNNNNNNNNNNNNNNNNNNNNNNNNNAGCTCGCCACGAAGACCTTCGGCAGCTCCGAATCGCCGTCCGTCGACAGCGGYACGGCGACCGCCCCTCCTGACCAGCCCTCCGACGACGGCGGCGCAGGGTCCGATGTTGAGTCGYACTCCTCCATGCCCCCCCTTGAGGGGGAGCCGGGGGATCCCGATCTCAGCGACGGGTCTTGGTCCACCGTGAGCGAGGAAGCTAGTGAGGACGTCGTCTGCTGC

>HM106808

TCCGGCTCGTGGCTAAGGGACGTTTGGGACTGGATATGCACGGTGTTAACTGATTTTAAGACCTGGCTCCAGTCCAAGCTCCTGCCACGATTACCGGGGGTCCCCTTTCTCTCATGCCAACGTGGGTACAAGGGCGTCTGGCGGGGAGACGGCATCATGCAAACCACCTGCCCGTGTGGAGCGCAGATCACCGGACATGTCAAGAACGGTTCCATGAGGATCGTTGGGCCTAGGACCTGCAGCAACACGTGGCATGGAACATTCCCCATCAACGCGTACACCACGGGCCCCTGCACGCCCTCCCCGGCGCCCAATTACTCCAGGGCGCTGTGGAGGGTGGCTGCTGAGGAGTACGTGGAGGTTACGCAGGTGGGGGATTTCCACTACGTGACGGGCATGACCACTGACAACGTAAAGTGCCCATGCCAGGTTCCGGCCCCCGAATTCTTCACGGAGGTGGATGGGGTGCGGCTGCACAGGTAYGCTCCGCCGTGTAAACCTCTCCTACGGGAGGAGGTCACATTCCAGGTCGGGCTCAACCAATACCTGGTCGGGTCACAGCTCCCATGTGAGCCCGAACCGGATGTAGCGGTGCTCACTTCCATGCTCACCGACCCCTCCCATATCACWGCAGAAGCGGCTAAGCGYAGGCTGGCTAGGGGGTCTCCCCCCTCCTTRGCCAGCTCYTCAGCTAGCCAGYTGTCTGCGCCCTCCCTGAAGGCGACATGCACTACCCGTCATGACTCCCCAGATGCTGACCTCATTGAGGCCAATCTCCTGTGGCGACAGGAGATGGGCGGGAACATCACCCGCGTGGAGTCAGAGAATAAGGTAGTAATTCTGGACTCTTTCGAGCCGCTTCGAGCGGAGGAAGATGAGAGGGAAGTATCCGTTCCGGCGGAAATCCTGCGGARATCCAGGAAATTTCCCCGAGCAATGCCCATATGGGCAAGGCCGGATTACAACCCTCCACTGGTAGAGTCCTGGAAGGACCCGGACTATGTCCCTCCGGTGGTACATGGGTGCCCATTACCACCTACTAAGGCCCCTCCAATACCACCTCCACGGAGGAAAAGGACGGTTGTCCTGACAGAATCCACCGTGTCCTCTGCCCTGGCTGAGCTCGCCACAAAGACCTTCGGCAGCTCCGGATCGTCGGCCGTCGACAGCGGCACGGCGACCGCCCCTCCTGACCAACCCTCCGACGANNNNNNNNNNNNNNNNNNNNNNNNNNNNNNNNNNNNNNTGCCCCCCCTTGAGGGGGAGCCGGGGGATCCCGATCTCAGCGACGGGTCTTGGTCTACCGTGAGCGAGGAGGCTAGTGAGGATGTTGTCTGCTGC

>HM106809

TCCGGCTCGTGGCTAAGGGACGTTTGGGACTGGATATGCACGGTGTTAACTGATTTTAAGACCTGGCTCCAGTCCAAGCTCCTGCCGCGGTTACCGGGAGTACCCTTCTTCTCATGCCAACGTGGGTACAAGGGCATCTGGCGGGGAGACGGCATCATGCAAACCACCTGCCCATGTGGAGCGCAGATCACCGGACATGTCAAGAACGGTTCCATGAGGATCGTTGGGCCTAGGACCTGCAGCAACACGTGGCATGGAACATTCCCCATCAACGCGTACACCACGGGCCCCTGCACGCCCTCCCCGGCGCCCAATTATTCCAGGGCGCTGTGGAGGGTGGCTGCTGAGGAGTACGTGGAGGTTACGCGGGTGGGGGATTTCCACTACGTGACGGGCATGACCACTGACAACGTAAAGTGCCCATGYCAGGTYCCAGCCCCCGAATTCTTCACGGAGGTGGATGGGGTGCGGCTGCACAGGTACGCTCCGCCGAGCAAACCTCTCCTACGGGAGGAGGTCACATTCCAGGTCGGGCTCAACCAATACCTGGTTGGGTCACAGCTCCCATGTGAGCCCGAACCGGATGTAGCGGTGCTTACTTCCATGCTCACCGACCCCTCTCACATCACAGCAGAGGCGGCTAAGCGTAGGCTGGCCAGGGGGTCTCCCCCCTCCTTAGCCAGTTCTTCAGCTAGCCAGTTGTCTGCGCCTTCTTTGAAGGCAACGTGCACTACCCGTCATGACTCCCCAGATGCTGACCTCATCGAGGCCAATCTCCTGTGGCGGCAGGAGATGGGCGGGAACATCACTCGCGTGGAGTCAGAGAACAAGGTAGTAATTCTGGACTCTTTCGAACCGCTTCGAGCGGAGGARGATGAGAGGGAAGTATCCGTTCCGGCGGAAATCCTGCGGAGATCCAGGAAATTTCCCCGAGCGATGCCCATATGGGCAAGGCCGGATTACAACCCTCCACTGATAGAGTCCTGGAAGGACCCGGACTACGTCCCTCCCGTGGTACATGGGTGCCCATTGCCACCTACTAAGGCCCCTCCAATACCACCTCCACGGAGGAAAAGGACGGTTGTCCTGACAGAATCCACCGTATCTTCTGCCTTGGCTGAGCTCGCCACAAAGACCTTCGGCAGCTCCGAATCGTCGGCTGTCGACAGCGGCACAGCGACCGCCCCACCYGACCAGCTCTCCGACGACGGCGACGCAAGGTCCGATGTTGAGTCGTACTCCTCCATGCCCCCCCTTGAGGGGGAGCCGGGGGATCCCGATCTCAGCGACGGGTCTTGGTCTACCGTGAGCGAGGAAGCTGGTGAGGACGTCGTYTGTTGC

>HM106810

TCCGGCTCGTGGCTAAGGGACGTTTGGGACTGGATATGYACGGTGTTARCTGATTTTAAGACCTGGCTCCAGTCCAAGCTYCTGCCGCGGTTACCGGGGGTCCCCTTYCTCTCATGCCAACGTGGGTACAAGGGCGTCTGGCGGGGAGACGGCATCATGCAAACCACCTGCCCATGTGGAGCGCAGATYACCGGACATGTCAAAAACGGTTCCATGAGGATCGTTGGGCCTAGGACCTGTAGCAACACGTGGCATGGAACATTCCCCATCAACGCGTACACCACGGGCCCCTGCACGCCCTCTCCGGCGCCCAATTACTCCAGGGCGCTGTGGAGGGTGGCTGCTGAGGAGTACGTGGAGGTTACGCAGGTGGGGGATTTCCACTACGTGACGGGCATGACCACTGACAACGTAAARTGCCCATGTCAGGTTCCGGCCCCCGAGTTCTTCACGGAGGTGGAYGGGGTACGGCTGCACAGGTACGCTCCGCCGTGCAAACCTCTCCTACGGGAGGAGGTCACATTCCAGGTCGGGCTCAACCAATACCTGGTCGGGTCACAGCTCCCRTGTGAGCCCGAACCRGATGTAGCGGTGCTCACTTCTATGCTCACCGACCCCTCCCATATCACAGCAGAAGCGGCTAAGCGTAGGCTGGCCAGGGGGTCTCCCCCCTCCTTGGCCAGCTCTTCAGCTAGCCAGTTGTCTGCGCCTTCCTTGAAGGCGACATGCACTACCCGTCATGACTCCCCAGATGCTGACCTCATTGAGGCCAATCTCCTGTGGCGGCAGGAGATGGGCGGAAACATCACCCGCGTGGAGTCAGAGAATAAGGTAGTAATTYTGGACTCTTTCGAGCCGCTCCGAGCGGAGGAAGATGAGAGGGAAGTRTCCGTTCCGGCGGAAATCCTGCGGAGATCCAGGAAATTTCCCCGAGCGATGCCCATATGGGCAAGGCCGGACTACAACCCTCCACTGGTAGAGTCNNNNNNNNNNNNNNNNNNNNNNNNNNNNNNNNNNNNNNNNNNNNNNNNNNNNNNNNNNNNNNNNNNNNNNNNNNNNNNNNNNNNNNNNNNNNNNNNNNNNNNNNNNNNNNNNNNNNNNNNNNNNNNNNNNNNNNNNNNNNNNNNNNNNNNNNNNNNNNNNNNNNNNNNNNNNNNNNNNNNNNNNNNNNNNNNNNNNNNNNNNNNNNNNNNNNNNNNNNNNNNNNNNNNNNNNNNNNNNNNNNNNNNNNNNNNNNNNNNNNNNNNNNNNNNNNNNNNNNNNNNNNNNNNNNNNNNNNNNNNNNNNNNNNNNNNNNNNNNNNNNNNNNNNNNNNNNNNNNNNNNNNNNNNNNNNNNNNNN

>HM106811

TCCGGTTCGTGGCTAAGGGACGTTTGGGACTGGATATGCACAGTGCTAACTGATTTTAAGACCTGGCTCCAGTCCAAGCTCCTGCCGCGGTTACCAGGAGTTCCCTTCCTCTCATGCCAACGTGGGTACAAGGGCATCTGGCGGGGGGACGGCATCATGCACACCACTTGCCCATGTGGAGCGCAGATCACCGGACATGTCAAAAACGGTTCCATGAGGATCGTTGGGCCTAGAACCTGCAGCAACACGTGGCATGGAACATTCCCCATCAACGCATACACCACGGGCCCCTGCACGCCCTCCCCGGCGCCCAATTATTCCAGGGCGCTGTGGAGGGTGACTGCYGAGGAGTACGTGGAGGTTACGCGGGTGGGGGATTTCCACTACGTGACGGGCATGACCACTGACAACGTAAAGTGCCCATGCCAGGTTCCGGCCCCCGAATTCTTCACRGAGGTGGATGGGGTGCGGCTGCACAGGTACGCTCCGCCGTGCAAACCYCTCCTACGGGAGGAGGTCACATTCCAGGTCGGGCTCAACCAATACCTGGTCGGGTCACAGCTCCCWTGYGAGCCCGAACCGGATGTAGCGGTGCTCACTTCCATGCTCACCGACCCCTCCCACATCACAGCAGAGGCGGCTAAGCGTAGGCTGGCCAGGGGGTCTCCCCCCTCYTTRGCCAGCTCTTCAGCTAGCCAGTTGTCTGCGCCTTCCTTGAAGGCGACATGCACTACCCAYCATGACTCCCCAGATGCTGACCTCGTCGAGGCCAATCTCCTGTGGCGGCAGGAGATGGGCGGRAACATCACCCGCGTGGAGTCAGAGAACAAGGTAGTAATTCTGGACTCTTTCGAGCCGCTYCGAGCGGAGGAAGATGAGAGGGAAGTATCCGTTCCGGCGGARATCCTGCGGAGATCCAGGAARTTCCCCCGAGCGATGCCCATATGGGCAAGGCCGGATTACAACCCTCCACTGGTGGAGTCCTGGAAGGACCCGGACTACGTCCCTCCGGTGGTRCATGGGTGCCCATTGCCACCTACTAAGGCCCCTCCRATACCACCTCCMCGGAGGAAAAGGACGGTTGTCCTGACAGAATCCACCGTGTCTTCTGCCTTGGCTGAGCTCGCCACAAAGACCTTCGGCAGCTCCGAATCGTCGGCCGTCGACAGCGGCACGGCGACCGCCCCTCCTGAYCAGCCCTCCGACGACGGCGACGCAGGGTCCGATGTTGAGTCGTACTCCTCCATGCCCCCCCTTGAGGGGGAGCCGGGGGATCCCGATCTCAGCGACGGGTCTTGGTCTACCGTGAGCGAGGAAGCTAGTGAGGACGTCGTCTGYTGC

>HM106812

TCCGGCTCGTGGCTAAGGGATGTTTGGGACTGGATATGCACGGTGTTAACYGAYTTTAAGACCTGGCTCCAGTCCAAGCTCCTGCCGCGGTTACCGGGAGTCCCCTTCCTCTCATGCCAACGTGGGTACAAGGGCATCTGGCGGGGAGAYGGCATCATGCAAACCACCTGCCCATGTGGAGCGCAGATCACTGGACATGTNNNNNNNNNNNNNNNNNNNNNNNNNNNNNNNNNNNNCTGCAGCAACACGTGGCATGGRACATTCCCCATCAACGCATACACCACGGGGCCCTGCACGCCCTCCCCGGCGCCCAATTATTCCAGGGCGTTGTGGAGGGTAGCTGCTGAGGAGTACGTGGAAGTTACGCGGGTGGGGGATTTCCACTACGTGACGGGCATGACCACTGACAACGTAAAGTGCCCATGCCAGGTTCCGGCCCCCGAATTCTTCACGGAGGTGGATGGGGTGCGGCTGCACAGGTACGCTCCGCTGTGCAAACCTCTCCTACGGGAGGAGGTCACATTCCAGGTCGGGCTCAACCAATACTTGGTTGGGTCACAGCTCCCATGTGAGCCCGAACCGGATGTAGCGGTGCTCACCTCCATGCTCACCGAYCCCTCCCACATCACAGCAGAGGCGGCTAAGCGTAGGCTGGCCAGGGGGTCTCCCCCCTCCCTGGCCAGCTCTTCAGCTAGCCAGTTGTCTGCGCCTTCCTTGAAGGCGACATGCACTACCCGTCATGACTCCCCGGACGCTGACCTCATCGAGGCCAATCTCCTGTGGCGGCAGGAGATGGGCGGGAACATCACCCGCGTGGAGTCAGAGAACAAGGTAGTAATTCTGGACTCTTTCGAGCCGCTTCGAGCGGAGGAAGAYGAGAGGGAAGTRTCCGTCCCGGCGGAAATCCTGCGGAGATCCAGGAAATTTCCCCGRGCGATGCCCGTATGGGCAAGGCCGGATTACAACCCTCCACTGGTAGAGTCCTGGAAGGACCCGGACTACGTCCCTCCGGTGGTACATGGGTGCCCATTRCCACCTRCTAAGGCCCCTCCAATACCACCTCCACGGAGGAAAAGGACGGTTGTCCTGACAGAGTCCACCGTGTCTTCTGCCTTGGCTGAGCTCGCCACAAAGACCTTCGGCAGCTCCGAATCGTCGGCCGTCGATAGCGGCACGGCGACCGCCTCTCCAGACCAGCCCTCCGACGNNNNNNNNNNNNNNNNNNNNNNNNNNNNNNNNNNNNNNNNNNNNNNNNNNNNNNNNNNNNNNNNNNNNNNNNNNNNNNNNNNNNNNNNNNNNNNNNNNNNNNNNNNNNNNNNNNNNNNNNNNNNNNNNNGCTGC

>HM106813

TCCGGTTCGTGGCTAAGGGATGTTTGGGACTGGATTTGCACGGTGTTAACCGATTTTAAGACCTGGCTCCAGTCTAAGCTCCTGCCGCGGTTACCGGGAGTCCCCTTCCTCTCATGCCAACGTGGGTACAAGGGCATCTGGCGGGGAGACGGCATCATGCAAACCACCTGCCCATGTGGAGCGCAGATCACCGGACATGTCAAAAACGGTTCCATGAGGATCKTTGGGCCTAGGACCTGCAGCAACACGTGGCATGGAACATTCCCCATCAACGCATACACCACGGGCCCCTGCACGCCCTCCCCGGCGCCCAATTATTCCAGGGCGCTGTGGAGGGTGGCTGCTGAGGAGTACGTGGAGGTTACGCGGGTGGGGGATTTCCACTACGTGACGGGCATGACCACTGACAACGTAAAGTGCCCATGCCAGGTTCCGGCCCCCGAATTCTTCACGGAGGTGGATGGGGTGCGGCTGCACAGGTACGCTCCGCCGTGCAAACCTCTCCTACGGGAGGAGGTCACATTCCAGGTCGGGCTCAACCAATACCTGGTCGGGTCACAGCTCCCATGTGAGCCCGARCCGGATGTAGCGGTGCTCACTTCCATGCTCACCGACCCCTCCCACATCACAGCAGAGGCGGCTAAGCGTAGGCTGGCYAGGGGGTCTCCCCCCTCCTTRGCCAGCTCTTCAGCTAGCCAGCTGTCTGCGCCTTCCTTGAAGGCGACATGCACTACCCGTCATGACTCCCCAGATGCTGACCTCATCGAGGCCAATCTCCTGTGGCGGCAGGAGATGGGCGGGAACATCACCCGCGTGGAGTCAGAGAACAAGGTAGTAATTCTGGACTCTTTTGAACCGCTTCGAGCGGAGGAAGATGAGAGGGAAGTATCCGTTCCGGCGGAAATCCTGCGGAGATCCAAGAAATTTCCCCGAGCGATGCCCATATGGGCAAGGCCGGATTACAACCCTCCATTGGTAGAGTCCTGGAAGGACCCGGACTATGTCCCTCCGGTGGTACATGGGTGCCCATTGCCACCTACTAAGGCCCCTCCAATACCACCTCCACGGAGGAAAAGGACGGTTGTCCTGACAGAATCCACCGTGTCTTCTGCCCTGGCTGAGCTCGCCACAAAGACCTTCGGCAGCTCCGAATCGTCGGCCGTCGACAGCGGCACGGCGACCGCCCCTCCTGAYCAGGCCTCCGACRACGGCGACGCAGGGTCCGATGTTGAGTCGTACTCCTCCATGCCCCCCYTKGAGGGGGAGCCGGGGGATCCCGATCTCAGCGACGGGTCTTGGTCTACCGTGAGCGAGGAGGCKAGTGAGGACGTCGTCTGCTGC

>HM106814

TCCGGCTCGTGGCTAAGGGACGTTTGGGACTGGATATGCAYGGTGTTAACTGATTTTAAGACCTGGCTCCAGTCCAAGCTCCTGCCGCGGTTGCCGGGAGTCCCCTTCYTCTCATGCCAACGTGGGTACAAGGGCATCTGGCRGGGAGACGGCATCATGCAAACCACCTGCCCATGTGGAGCGCAGATCACCGGACACGTCAAAAACGGTTCCATGAGGATCGTTGGGCCTAGGACCTGTAGCAACACGTGGCATGGRACATTCCCCATCAACGCATACACCACGGGCCCCTGCACGCCCTCCCCGGTGCCCAATTATTCCAGGGCGCTGTGGAGGGTGGCTGCTGAGGAGTACGTGGAGGTTACGCGGGTGGGGGATTTCCACTACGTGACGGGCATGACCACTGACAACGTAAAGTGCCCATGCCAGGTTCCGGCCCCCGAATTCTTCACGGAGGTGGATGGGGTGCGGCTGCACAGGTACGCTCCGCCGTGCAAGCCTCTCCTACGGGAGGAGGTCACATTCCAAGTCGGGCTTAACCAATACTTGGTTGGGTCACAGCTCCCATGTGAGCCCGAACCGGATGTAGCAGTGCTCACTTCCATGCTCACCGACCCCTCCCACATCACAGCRGAGGCGGCTAAGCGTAGGCTGGCCAGGGGGTCTCCCCCCTCCTTAGCCAGCTCTTCAGCTAGCCAGTTGTCTGCACCTTCCTTGAAGGCGACATGCACTRCCTGTCATGACTCCCCAGATGCTGCCCTCATCGAGGCCAATCTCCTGTGGCGGCAGGAGATGGGCGGGAACATCACCCGCGTGGAGTCAGAGAACAAGGTAGTAATTCTGGACTCTTTCGAGCCGCTTCGAGCGGAGGAAGATGAGGGGGAAGTATCCGTTCCGGCGGAAATCCTGCGGAGATCCAGGAAATTTCCCCGAGCGATGCCCATATGGGCAAGGCCGGATTACAACCCTCCACTGGTAGAGTCCTGGAAGGACCCGGACTACGTCCCTCCGGTGGTACATGGGTGCCCATTGCCACCTACTAAAGCCCCTCCRATACCACCTCCACGGAGGAAAAGGACGATTGTCCTGACAGAATCCACCGTATCTTCTGCCTTGGCTGAGCTCGCCACAAAGACCTTCGGCAGCTCCGAATCGTCGGCCGTTGACAGCGGTACGGCGACCGCCCCTCCTGACCAGSCCTCCGACGACGGCGGYGCAGGGTCCGATGTTGAGTCGTACTCCTCCATGCCCCCCCTTGAGGGGGAGCCGGGGGATCCCGATCTCAGCGACGGGTCTTGGTCTACCGTGAGCGAGGAAGCTAGTGAGGACGTCGTCTGCTGC

>HM106815

NNNNNNNNNNNNNNNNNNNNNNNNNNNNNNNNNNNNNNNNNNNNNNNNNNNNNNNNNNNNNNNNNNNNNNNNNNNNNNNNNNNNNNNNNNNNNNNNNNNNNNNNNNNNNNNNNNNNNNNNNNNNNNNNNNNNNNNNNNNNNNNNNNNNNNNNNNNNNNNNNNNNNNNNNNNNNNNNNNNNNNNNNNNNNNNNNNNNNNNNNNNNNNNNNNNNNNNNNNNNNNNTTGGGCCTAGGACCTGCAGCAACACGTGGCATGGGACATTCCCCATCAACGCGTACACCACGGGCCCCTGCACGCCCTCCCCGGCGCCCAATTACTCCAGGGCGCTGTGGAGGGTGGCTGCTGAGGAGTACGTGGAGGTCACGCAGGTGGGGGATTTCCACTACGTGACGGGCATGACCACCGACAACGTAAAGTGCCCATGCCAGGTTCCGGCCCCCGAATTCTTCACRGAGGTGGATGGGGTGCGGCTGCACAGGTACGCTCCGCCGTGCAAACCTCTCCTACGGGAGGAGGTCACATTCCAGGTCGGGCTCAACCAATACCTGGTCGGGTCACAGCTCCCRTGYGAGCCCGAACCGGATGTGGCGGTGCTCACTTCCATGCTCACCGACCCCTCCCATATCACAGCAGAGACGGCYAAGCGTAGGCTGGCCAGGGGGTCTCCCCCCTCCTTGGCCAGCTCTTCAGCTAGCCAGTTGTCTGCGCCTTCCTTGAAGGCGACATGCACTACCCGTCATGACTCCCCAGATGCTGACCTCATTGAGGCCAATCTCCTGTGGCGGCAGGAGATGGGCGGGAACATCACCCGCGTGGAGTCAGAGAATAAGGTAGTAATTCTGGACTCTTTCGAGCCGCTTCGAGCGGAGGAAGATGAGAGGGAAGTATCCGTTCCGGCGGAAATCCTGCGGARATCCAGGAAATTTCCCCGGGCGATGCCCATATGGGCAAGGCCGGATTACAACCCTCCACTGGTRGAGTCCTGGAAGGACCCGGACTACGTCCCTCCGGTGGTACATGGGTGCCCGTTACCACCCACTAAGGCCCCTCCAATACCACCTCCACGGAGRAAAAGGACGGTTGTCCTGACAGAATCCACCGTGTCTTCYGCCTTGGCTGAGCTCGCCACRAAGACCTTCGGCAGCTCCGAATCGTCGGCCGTCGACAGCGGCACGGCGACCGCCCCTCCTGACCAGCCCTCCGACGAYGGCGACGCAGGGTCCGATGTTGAGTCGTACTCCTCCATGCCCCCCCTTGAGGGGGAGCCGGGGGATCCCGATCTCAGCGACGGGTCTTGGTCTACCGTGAGCGAGGAGGCTAGTGAGGACGTCGTCTGCTGC

>HM106816

NNNNNNNNNNNNNNNNNNNNNNNNNNNNNNNNNNNNNNNNNNNNNNNNNNNNNNNNNNNNNNNNNNNNNNNNNNNNNNNNNNNNNNNNNNNNNNNNNNNNNNNNNNNNNNNNNNNNNNNNNNNNNNNNNNNNNNNNNNNNNNNNNNNNNNNNNNNNNNNNNNNNNNNNNNNNNNNNNNNNNNNNNNNNNNNNNNNNNNNNNNNNNNNNNNNNNNNNNNNNNNNNNNNNNNNNNNNNNNNNAGCAACACGTGGCATGGRACATTCCCCATCAACGCATACACCACGGGCCCCTGCACGCCCTCCCCGGCGCCCAATTATTCTAGGGCGCTGTGGAGGGTGGCCGCTGAGGAGTACGTGGAGGTTACGCGGGTGGGGGATTTCCACTACGTGACGGGCATGACCACTGACAACGTGAAGTGCCCATGCCAGGTTCCGGCCCCCGAATTCTTCACGGAGGTGGATGGGGTGCGGCTGCACAGGTACGCTCCGCCGTGCAAACCTCTCCTACGGGAGGAGGTCACATTCCAGGTCGGGCTCAACCAATACCTGGTYGGGTCACAGCTCCCATGTGAGCCCGAACCGGATGTAGCGGTGCTTACTTCCATGCTCACCGACCCCTCCCACATCACAGCAGAGGCGGCTAAGCGTAGGCTGGCCAGGGGGTCTCCCCCCTCCTTGGCCAGCTCTTCAGCTAGCCAGTTGTCTGCGCCTTCCTTGAAGGCGACATGCACTACCCATCATGACTCCCCAGATGCTGACCTCATCGAGGCCAATCTCCTGTGGCGGCAGGAGATGGGCGGGAACATCACCCGCGTGGAGTCAGAGAACAAGGTAGTAATTCTGGATTCCTTCGAGCCGCTTCGAGCGGAGGAAGATGAGAGGGAAGTATCCGTTCCGGCGGAAATCCTGCGGAGATCCAGGAAATTTCCCCGGGCGATGCCCATATGGGCAAGGCCGGATTACAACCCTCCACTGGTGGAGTCCTGGAAAGACCCGGACTACGTCCCTCCGGTGGTACATGGGTGCCCATTGCCACCTACTAAGGCCCCTCCAATACCACCTCCACGGAGGAAAAGGACGGTCGTCCTGACAGAATCCACCGTGTCCTCTGCTTTGGCTGAGCTCGCCACAAAGACCTTCGGCAGCTCCGAATCGTCGGCCGTCGACAGTGGCACGGCGACTGCCCCTCCTGACCAGCCCTCCGACGACGGCGNNNNNNNNNNNNNNNNNNNNNNNNNNNNNNNNNNNNNNNNNNNNNNNNNNNNNNNNNNNNNNNNNNNNNNNNNNNNNNNNNNNNNNNNNNNNNNNNNNNNNNNNNNNNNNNNNNNNNNNNNNNNNNNN

>HM106817

TCCGGCTCGTGGCTRAGGGAYGTTTGGGACTGGATATGCACGGTGYTAAGTGATTTTAAGACCTGGCTCCAGTCCAAGCTCCTGCCGCAATTACCGGGGCTCCCCTTCCTCTCATGCCAACGTGGGTACAAGGGYGTCTGGCGGGGAGACGGCATCATGCAAACCACCTGCCCATGTGGAGCGCAGATCACCGGACATGTCAAGAACGGTTCCATGAGGANNNNNNNNNNNNNNNNNNNNNNNNNNNNNNNNNNNNNNNNNTTTCCCATCAACGCGYACACCACGGGCCCCTGCACGCCCTCCCCGGCGCCCAATTAYTCCAGGGCGCTGTGGAGGGTGGCTGCTGAGGAGTACGTGGAGGTTACGCGGGTGGGGGATTTCCACTACGTGACGGGCATGACCACTGACAACGTAAAGTGCCCATGCCAGGTTCCGGCCCCTGAATTYTTCACRGAGGTGGAYGGGGTGCGGCTGCACAGGTACGCCCCGYCGTGYAAACCTCTCCTACGGGAGGAGGTCACATTCCAGGTCGGGCTCAACCAATACCTGGTCGGGTCACAGCTCCCATGYGAGCCCGAACCGGATGTRGCGGTGCTCACTTCCATGCTCACCGACCCCTCCCAYATCACAGCAGAAACGGCTAAGCGTAGGCTGGCCAGGGGGTCTCCCCCCTCCTTAGCCAGCTCTTCAGCTAGCCAGTTGTCTGCGCCTTCCTTGAAGGCGACATGCACTACCCGTCATGACTCCCCAGATGCTGACCTCATTGAGGCCAATCTCCTGTGGCGGCAGGAGATGGGCGGGAACATCACCCGCGTGGAGTCAGAGAATAAGGTAGTAATTCTGGACTCTTTCGAGCCGCTTCGAGCGGAGGAAGATGAGAGGGAARTATCCGTTCCGGCGGAAATCCTGCGGARATCCAGRAAATTTCCCCGAGCGATGCCCATATGGGCAAGGCCGGATTACAACCCTCCACTGGTRGAGCCCTGGAAGGACCCGAACTATGTCCCTCCGGTGGTACATGGGTGCCCATTACCACCTASTAAGGCCCCTCCAATACCACCTCCACGGAGGAARAGGACGGTTGTCCTGACAGAATCCACCGTGTCTTCTGCCTTGGCWGAGCTCGCCACAAAGACCTTTGGCAGCTCTGAATCGTCGGCCGYCGACAGCGGCACGGCGACCGCCYCTCGTGACCAGNNNNNNNNNNNNNNNNNNNNNNNNNNNNNNNNNNNNNNNNNNNNNNNNNNNNNNNNNNNNNNNNNNNNNNNNNNNNNNNNNNNNNNNNNNNNNNNNNNNNNNNNNNNNNNNNNNNNNNNNNNNNNNNNNNNNNNNNNNNNNNNN

>HM106818

TCCGGCTCGTGGCTAAGGGACGTTTGGGACTGGATATGCACGGTGTTAACTGATTTTAAGACCTGGCTCCAGTCCAAGCTCCTGCCGCGGTTACCGGGAGTCCCCTTCCTCTCATGCCAACGTGGGTACAAGGGCATCTGGCGGGGAGACGGCATCATGCAAACCACCTGCCCGTGTGGAGCGCARATCACCGGACATGTCAAAAACGGTTCCATGAGGATCGTCGGGCCYAGGACCTGCAGCAACACGTGGCATGGAACATTCCCCATCAACGCATACACCACGGGCCCCTGCACGCCCTCCCCGGCGCCCAATTATTCCAGGGCGCTGTGGAGGGTGGCTGCTGAGGAGTACGTGGAGGTTACGCGGGTGGGGGATTTCCACTACGTGACGGGCATGACCACTGACAACGTAAAGTGCCCATGCCAGGTTCCGGCCCCCGAATTCTTCACGGAGGTGGATGGGGTGCGGCTGCACAGGTACGCYCCGCCGTGYARACCTCTCCTACGGGAGGAGGTTACATTCCAGGTCGGGCTCAACCAATACCTGGTCGGGTCACAGCTACCATGCGAGCCCGAACCGGATGTAGCGGTGCTCACTTCCATGCTCACCGACCCCTCCCACATCACAGCAGAGGCGGCTAAGCGTAGGCTGGCCAGGGGGTCTCCCCCCTCCTTGGCCAGCTCTTCAGCTAGCCAGTTGTCTGCGCCYTCTTTGAAGGCGACATGCACTACCCRTCATGACTCCCCAGATGCYGACCTCATYGAGGCCAATCTCCTGTGGCGGCAGGAGATGGGCGGGAACATCACCCGCGTGGAGTCAGAGAACAAGGTAGTAATTCTGGACTCTTTCGAGCCGCTTCGAGCGGAGGAAGATGAGAGGGAAGTATCCGTTCCGGCGGAAATCCTGCGGAAATCCAGGAAATTTCCCCGAGCGATGCCCATATGGGCAAGGCCGGATTACAACCCTCCACTGGTAGAGTCCTGGAAGGACCCGGACTACGTCCCTCCGGTGGTACATGGGTGCCCAYTGCCACCTACTAAGGCCCCTCCAATACCACCTCCACGGAGGAARAGGACGGTTGTCCTGACAGAATCCACCGTGTCTTCTGCCTTGGCTGAGCTCGCCACAAAGACCTTCGGCAGCTCCGAATCGTCGGCCGTCGACAGCGGCACGGCGACCGCCCCTCCTGACCAGSCCTCCGACGACGGCGACGCAGGGTCCGACGYTGAGTCGTATTCCTCCATGCCCCCCCTTGAGGGGGAGCCGGGGGATCCCGATCTCAGCGACGGGTCTTGGTCTACCGTGAGCGAGGAGGCTAGTGAGGACGTCGTCTGCTGC

>HM106819

TCCGGCTCGTGGCTAAGGGACGTTTGGGACTGGATATGCACGGTGTTAACYGAYTTTAAGACCTGGCTCCARTCCAAGCTCCTGCCGCGGTTACCGGGAGTCCCCTTCCTCTCATGCCAACGTGGRTACAAGGGYATCTGGCGGGGAGACGGCATCATGCAWACCACCYGCCCATGTGGAGCGCARATCACCGGACATGTCAAAAACGGNNNNNNNNNNNNNNNTGGGCCTAGGACCTGCAGCAACACGTGGCATGGAACATTCCCCATCAACGCRTACACCACGGGCCCCTGCACGCCCTCCCCCGCGCCCAAYTATTCCAGGGCGCTGTGGAGGGTGGCTGCMGAGGAGTACGTGGAGGTTASRMGGGTGGGGGATTTCCACTACGTGACGGGCATGACYRCTGACAACRTAAARTGCCCATGCCAGGTTCCGGCCCCCGAATTCTTCACGGAGGTGGATGGGGTACGGCTGCACAGGTACGCTCCGCCGTGCARACCTCTCCTACGGGAGGAGGTCACATTCCAGGTCGGGCTCAACCAATACCTGGTYGGGTCACAGCTCCCATGYGAGCCCGAACCRGATGTARCRGTGCTCACTTCCATGCTCACCGACCCCTCCCACATCACAGCAGAGGCAGCTAAGCGYAGGCTGGMCAGGGGGTCTCCCCCCTCCTTAGCCAGCTCTTCAGCTAGCCAGYTGTCTGCGCCTTCYTTGAAGGCRACATGCACTACCCGTCATGACTCCCCAGATGCTGACCTCATCGAGGCCAATCTCCTGTGGCGACAGGAGATGGGCGGGAACATCACCCGCGTGGAGTCAGARAACAAGGTAGTAATTCTGGACTCTTTCGAGCCGCTTCGRGCGGAGGAAGATGAGAGGGAAGTATCCGTTCCGGCGGARATCCTGCGRAGATCCAGGAARTTYCCCCGRGCGATGCCCATATGGGCAAGGCCGGATTACAACCCTCCACTGGTGGAGTCCTGGAAGRACCCGGACTACGTCCCTCCGGTGGTACATGGGTGCCCATTACCACCTACTAAGGCCCCTCCAATACCACCTCCANNNNNNNNNNNNNNNNNNNNNNNNNNNNNNNNNNNNNNNNNNNNNNNNNNNNNNNNNNNNNNNNNNNNNNNNNNNNNNNNNNNNNNNNNNNNNNNNNNNNNNNNNNNNNNNNNNNNNNNNNNNNNNNNNNNNNNNNNNNNNNNNNNNNNNNNNNNNNNNNNNNNNNNNNNNNNNNNNNNNNNNNNNNNNNNNNNNNNNNNNNNNNNNNNNNNNNNNNNNNNNNNNNNNNNNNNNNNNNNNNNNNNNNNNNNNNNNNNNNNNNNNNNNNNNNNNNNN

>HM106820

TCCGGCTCGTGGCTAAGGGACGTTTGGGACTGGATATGCWCGGTGTTAACTGACTTTAAGACCTGGCTCCAGTCCAAGCTCCTGCCGCGGTTACCGGGAGTCCCCTTCCTCTCATGCCAACGTGGGTACAAGGGCATCTGGCGGGGAGACGGCATCATGCAAACCACCTGCCCATGTGGAGCGCTGATCACCGGACATGTCAAAAACGGTTCCATGAGGATCNNNNNNNNNNNNNNNNNNNNNNACACGTGGCATGGRACATTCCCCATCAACGCGTACACCACGGGCCCCTGCACGCCCTCCCCGGCGCCCAATTACTCCAGGGCGCTGTGGAGGGTGGCTGCTGAGGAGTACGTGGAGGTTACGCGGGTGGGGGATTTCCACTACGTGACGGGCATGACCACTGACAACGTAAAGTGCCCATGCCAGGTTCCGGCCCCYGAATTTTTCACGGAGGTGGATGGGGTGCGGCTGCACAGGTACGCTCCGCCAAGCAAACCTCTCTTACGGGAGGAGGTCACGTTCCAGGTCGGGCTCAACCAATACCTGGTCGGGTCACAGCTCCCATGTGAGCCCGAACCGGATGTAGCGGTGCTCACTTCCATGCTCACCGACCCCTCCCACATCACAGCAGAGGCGGCTAAGCGTAGGCTGGCCAGGGGGTCTCCCCCCTCCTTAGCCAGCTCTTCAGCTAGCCAGCTGTCTGCGCCTTCCTTGAAGGCGACATGCACTACCCGTCATGACTCCCCAGACGCTGACCTCATCGAGGCCAATCTCCTGTGGCGGCAGGAGATGGGCGGGAACATCACCCGCGTGGAGTCAGAGAACAAGGTARTAATTCTGGACTCTTTCGAGCCGCTTCGAGCGGAGGAAGATGAGAGGGAAGTATCCGTTCCGGCGGAAATCCTGCGGAGATCCAGGAAATTTCCCCGAGCGATGCCCATATGGGCAAGGCCGGATTACAACCCTCCACTGGTAGAGTCCTGGAAGGACCCGGACTACGTCCCTCCGGTGGTACATGGGTGCCCATTGCCACCTACTAAGGCCCCTCCAATACCACCTCCACGGAGGAAAAGGACGGTTGTCCTGACGGAATCCACCGTGTCTTCTGCCTTGGCTGAGCTCGCCACAAAGACCTTCGGCAGCTCCGAATCGTCGGCCGTCGACAGCGGCACGGCGACCGSCCCTCCTGACCAGCCCTCCGACGACGGCGACGCAGGGTCCGATGTTGAGTCGTACTCCTCCATGCCCCCCCTTGAGGGGGAGCCGGGGGATCCCGATCTCAGCGACGGGTCTTGGTCTACCGTGAGCGAGGAAGCTAGTGAGGACGTCGTCTGCTGC

>HM106821

TCCGGCACGTGGCTAAGGGACGTTTGGGACTGGATATGCACGGTGTTAACYGATTTTAAGACCTGGCTCCAGTCCAAGCTCCTGCCGCGGTTRCCGGGGGTCCCYTTCCTCTCATGCCAGCGTGGGTACAAGGGMGTCTGGCGGGGAGACGGCATCATGCAYACCACCTGCCCATGTGGAGGACAGATCACCGGACATGTCAAAAACGGTTCCATGAGGATCGTTGGGCCTAGGACCTGCAGCAACACGTGGCATGGAACATTCCCCATCAACACATACACCACGGGTCCCTGCACGCCCTCCCCGGCGCCCAATTACTCCAGGGCGCTGTGGAGGGTGGCTGCTGAGGAATACGTGGAGGTTACACAGGTGGGGGATTTCCACTACGTGACGGGCATGACCACTGACAACGTAAAGTGCCCATGCCAGGTTCCGGCCCCCGAATTCTTCACGGAGGTGGATGGGGTGCGGCTGCACAGGTACGCTCCGCCGTGCAAACCCCTGCTACGGGAGGAGGTCACATTCCAGGTCGGGCTCAACCAATACCTGGTCGGGTCACAGCTCCCRTGTGAGCCCGAACCGGATGTAGCGGTGCTCACTTCCATGCTCACCGACCCCTCCCACATCACAGCAGAAACGGCTAAGCGYAGGCTGGCCAGGGGGTCTCCCCCCTCCTTGGCCAGCTCTTCAGCTAGCCAGTTGTCTGCGCCTTCCTTGAAGGCGACATGCACTACCCGTCATGACTCCCCAGATGCTGACCTCATTGAGGCCAATCTCCTGTGGCGGCAGGAGATGGGCGGGAACATCACCCGCGTGGAGTCAGAGAATAAGGTAGTAATTCTGGACTCTTTCGAGCCGCTTCGAGCGGAGGAAGATGAGAGGGAAGTATCCGTTCCGGCGGAAATCCTGCGAAGAACCAGGAAGTTTCCCCGAGCGATGCCCATATGGGCAAGGCCGGATTACAACCCTCCACTGGTGGAGTCCTGGAAGGACCCGGACTACGTCCCTCCGGTGGTACACGGGTGCCCATTACCACCTACTAAGGCCCCTCCAATACCACCTCCACGGAGGAAAAGGACGGTTGTCCTGACAGAATCCACCGTGTCTTCTGCYCTGGCTGAGCTCGCCACAAAGACCTTCGGCAGCTCCGAATCGTCGGCCGTCGACAGCGGCACGGCGACCGCCCCTCCTGACCAGCCCTCCGACGATGGCGACGCAGGGTCCGACGTYGAGTCGTACTCCTCCATGCCCCCCCTTGAGGGGGAGCCGGGGGATCCCGATCTTAGCGACGGRTCTTGGTCTACCGTGAGCGAGGAGGCCAGTGAGGACGTCGTCTGCTGC

>HM106822

NNNNNNNNNNNNNNNNNNNNNNNNNNNNNNNNNNNNNNNNNNNNNNNNNNNNNNNNNNNNNNNNNNNNNNNNNNNNNNNNNNNNNNNNNNNNNNNNNNNNNNNNNNNNNNNNNNNNNNNNNNNNNNNNNNNNNNNNNNNNNNNNNNNNNNNNNNNNNNNNNNNNNNNNNNNNNNNNNNNNNNNNNNNNNNNNNNNNNNNNNNNNNNNNNNNNNNNNNNNNNNNNNNNNNNNNNNNNNNNNNNNNNNNNNNNNNNNNNNNNNNNNCCCATCAACGCATACTCCACGGGCCCCTGCACACCCTCCCCGGCGCCCAATTATTCCAGGGCGCTGTGGAGGGTGGCTGCTGAGGAGTACGTGGAGRTTACGCGGGTGGGGGATTTCCACTACGTGACGGGCATGACYACTGACAACGTAAAGTGCCCATGCCAGGTTCCGGCCCCTGAATTCTTCACGGAGGTGGATGGGGTGCGGYTGCACAGGTAYGCTCCGCCGTGCAAACCTCTCCTACGGGAGGAGGTCACATTCCAGGTCGGGCTYAACCAATACCTGGTTGGGTCACAGCTCCCATGCGAGCCCGAACCGGATGTGGCGGTGCTTACTTCCATGCTCACCGACCCCTCCCACATCACAGCAGAGGCGGCTAAGCGTAGGCTGGCCAGGGGGTCTCCCCCCTCCTTGGCCAGCTCTTCAGCTAGCCAGTTGTCTGCGCCTTCTTTGAAGGCGACATGCACCACCCGTCATGACTCCYCAGATGCTGACCTCATCGAGGCCAATCTCCTGTGGCGGCAGGAGATGGGCGGGGACATCACCCGCGTGGAGTCAGAGAACRAGGTGGCAAAYCTGGACTCTTTCGAGCCGCTTCGAGCGGAGGAAGATGAGAGGGAAGTRTCCGTTCCGGCGGAAATCCTGCGGAGATCCAGGAAACTTCCCCSRGCGATGCCCATATGGGCAAGGCCGGATTACAACCCTCCACTGATAGAGYCCTGGAAGGACCCGGACTACGTCCCCCCGGTGGTGCATGGGTGCCCACTACCACCTACTAAGGCCCCTCCAATACCACCTCCACGGAGGAAAAGGACGGTCGTCCTGACAGAATCCACCGTGTCTTCTGCCCTGGCTGAGCTYGCCACAAAGACCTTCGGCAGCTCTGAATCGTCGGCCGCCGNNNNNNNNNNNNNNNNNNNNNNNNNNNNNNNNNNNNNNNNNNNNNNNNNNNNNNNNNNNNNNNNNNNGTCGTACTCCTCCATGCCCCCCCTCGAGGGGGAGCCGGGRGATCCCGATCTCAGCGACGGGTCTTGGTCYACCGTGARCGAGGAAGCTGGTGAGGACGTCGTCTGCTGC

>HM106823

NNNNNNNNNNNNNNNNNNNNNNNNNNNNNNNNNNNNNNNNNNNNNNNNNNNNNNNNNNNNNNNNNNNNNNNNNNNNNNNNNNNNNNNNNNNNNNNNNNNNNNNNNNNNNNNNNNNNNNNNNNNNNNNNNNNNNNNNNNNNNNNNNNNNNNNNNNNNNNNNNNNNNNNNNNNNNNNNNNNNNNNNNNNNNNNNNNNNNNNNNNNNNNNNNNNNNNNNNNNNNNNNNNNNNNNNNNNNNNNNNNNNNNNNNNNNNNNNNNNNNNNCCCCATCAACGCATACACCACGGGCCCCTGCACGCCCTCCCCGGCGCCCAATTATTCTAGGGCGCTGTGGAGGGTGWSTGCTGAGGAGTACGTGGAGGTTACGCGGGTGGGGGATTTCCACTACGTGACGGGCATGACCACTGACAACGTAAAGTGCCCATGCCAGGTTCCGGCCCCCGAATTCTTCACGGAGGTGGATGGGGTGCGGCTGCACAGGTACGCTCCGCCGTGCAAAGCTCTCCTACGGGAGGAGGTCACTTTCCAGGTYGGGCTCAACCAATACCTGGTCGGGTCACAGCTCCCATGTGAGCCYGAACCAGATGTAGCRGTGCTCACTTCCATGCTCACCGACCCCTCCCACATCACAGCAGAGACAGCTAAGCGTAGGCTGGCCAGGGGGTCYCCCCCCTCCTTAGCCAGCTCTTCAGCTAGCCAGTTGTCTGCGCCTTCCTTGAAGGCRACATGCACTACCCGTCATGACTCCCCAGATGCTGACCTCATCGAGGCCAATCTCCTGTGGCGGCAGGAGATGGGCGGGAACATCACCCGCGTGGAGTCAGAGAACAAGGTAGTAATTCTGGACTCTTTCGAACCGCTTCGAGCGGAGGAAGATGAGAGGGAAGTATCCATTCCGGCGGAAATCCTGCGGARATCCAGGAAATTYCCCCGAGCGATGCCCATATGGGCAAGGCCGGATTACAACCCYCCAYTGATAGAGTCCTGGAAGGACCCGGACTACGTCCCTCCGGTGGTRCATGGGTGCCCACTACCACCTACTAAGGCCCCTCCAATACCACCTCCACGGAGGAAAAGGACGGTTGTCCTGACAGAGTCCACCGTGTCTTCTGCCTTGGCTGAGCTCGCCACAAAGACCTTYGGCAGCTCCGGATCGTCGGCCGTCGACAGCGGCACGGCGACCGCCCCTCCTGACCAGCTCTCCGACGACGGCGACGCAGGGTCCGATGTTGAGTCGTACTCCTCCATGCCCCCCCTYGAGGGGGAGCCGGGGGATCCCGATCTCAGCGACGGGTCTTGGTCTACCGTGAGCGAGGAAGCTAGTGAGGACGTCGTCTGCTGC

>HM106824

GCCGGCTCGTGGCTAAGGGATGTTTGGGACTGGATATGCACGGTGTTAACCGATTTTARGACCTGGCTCCAGTCCARGCTCCTGCCGCGGTTACCGGGAGTCCCCTTCCTCTCATGCCAAYGCGGGTACAAGGGCATCTGGCGGGGAGACGGCATCATGCAAACCACCTGCCCATGTGGAGCGCAGATCACCGGACACGTCAAAAACGGTTCCATGAGGATCNNNNNNNNNNNNNNNNGCAGCAACACGTGGCATGGAACGTTCCCCATCAACGCGTACACCACGGGCCCCTGCACGCCCTCCCCGGCGCCCAATTATTCTAGGGCGTTGTGGAGGGTGGCTGCTGAGGAGTACGTGGAGGTCACGCGGGTGGGGGATTTCCACTACGTGACGGGCATGACCACTGACAACGTAAAGTGCCCATGYCAGGTTCCGGCCCCCGAATTCTTCACGGAGGTGGATGGGGTGCGGCTGCACAGGTACGCTCCGCCGTGCAAACCTCTCCTACGGGAGGAGGTCACATTCCAGGTCGGGCTCAACCAATACCTGGTTGGATCGCAGCTCCCATGTGAGCCCGAACCGGATGTGGCGGTACTCACTTCCATGCTCACCGACCCCTCCCACATCACAGCAGAGGCGGCTAAGCGTAGGCTGGCCAGGGGGTCTCCCCCCTCCTTGGCCAGCTCTTCAGCTAGCCAGTTGTCTGCGCCTTCCTTGAAGGCGACATGCACTACCCGTCATGACTCCCCAGATGCTGACCTCATCGAGGCCAATCTCCTGTGGCGGCAGGAGATGGGCGGGAACATCACCCGTGTGGAGTCAGAGAACAAGGTAGTAATTCTGGACTCTTTCGAGCCGCTTCGAGCGGAGGAAGATGAGAGGGAAGTATCCGTTCCGGCGGAAATCCTGCGGAGATCCAGGAATTTTCCCCGAGCGTTGCCCGTATGGGCAAGGCCGGATTACAACCCTCCACTGGTGGAGTCCTGGAAGGACCCGGACTACGTCCCTCCGGTGGTACAYGGGTGCCCATTGCCACCTACTAAGGCCCCTCCAATACCACCTCCACGGAGGAAAAGGACGGTTGTCCTGACAGAATCCACCGTGTCTTCTGCCTTGGCTGAGCTCGCCACAAAGACCTTCGGCAGCTCCGAATCGTCGGCCGTCGATAGCGGCACGGCGACCGCCCCTCCCGACCAGCCCCTCGACAACGGCGACACAGGGTCCGACGTTGAGTCGTACTCCTCCATGCCCCCCCTTGAGGGGGAGCCGGGGGATCCCGATCTCAGCGACGGGTCTTGGTCTACAGTGAGCGAGGAAGCTAGTGAGGACGTCGTCTGCTGC

>HM106825

TCCGGCTCGTGGCTAAGGGACGTTTGGGACTGGATATGCACGGTGTTARCTGATTTTAAGACCTGGCTCCAGTCCAAGCTCCTGCCGCGGTTRCCGGGAGTCCCCTTYTTCTCATGCCAACGTGGGTACAAGGGCATCTGGCGRGGAGACGGCATCATGCAAACCACCTGCCCATGTGGAGCGCARATYACCGGACATGTCAAAAACGGTTCCATGAGGATCGTTGGGCCTAGGACCTGCAGCAACACGTGGCATGGAACTTTCCCCATCAACGCGTACACCACGGGCCCYTGCACGCCCTCYCCGGCGCCCAATTATTCCAGGGCGCTGTGGAGGGTGGCTGCTGAGGAGTACGTGGAGGTTACGCGGGTGGGGGATTTCCACTACGTGACGGGCATGACCACTGACAACGTAAAGTGCCCATGCCAGGTTCCGGCCCCYGAATTCTTCACGGAGGTGGATGGGGTGCGGCTGCACAGGTACGCTCCGTCGTGCAAACCTCTCCTACGGGAGGAGGTCACATTCCRGGTCGGGCTCAACCAATACCTGGTCGGGTCACAGCTCCCATGCGAGCCCGAACCGGATGTGGCGGTGCTCACTTCCATGCTCACCGACCCCTCCCACATCACAGCAGAGGCGGCTAAGCGTAGGCTGGCCAGGGGGTCTCCCCCCTCCTTGGCCAGCTCGTCAGCTAGCCAGCTGTCTGCGCCTTCCTTGAAGGCGACATGCACTACCCGTCATGACTCCCCAGATGCTGACCTCATCGAGGCCAATCTCCTGTGGCGGCAGGAGATGGGCGGGAACATCACCCGCGTGGAGTCAGAAAACAAGGTAGTAATTCTGGACTCTTTCGAGCCGCTTCGAGCGGAGGAAGATGAGAGGGAAGTATCCGTTCCGGCGGARATCCTGCGGAAATCCAGGAAATTTCCCCGAGCGATGCCCATATGGGCAAGGCCGGATTACAACCCTCCACTGGTAGAGTCCTGGAAGGACCCGGACTACGTCCCTCCGGTGGTACATGGGTGCCCATTGCCACCTACTAAGGCCCCTCCAATACCACCTCCACGGAGGAAAAGGACGGTTGTCCTGACAGAATCCACCGTGTCTTCTGCCTTGGCTGAGCTCGCCACAAAGACCTTCGGCAGCTCTGAATCRTCGGCCGTYGACAGCGGCACGGCGACCGCCCCTCCTGACCAGCCCTCCGACGACGGCGACGCAGGGTCCGATGTTGAGTCGTACTCCTCCATGCCCCCCCTTGAGGGGGAGCCGGGGGATCCCGACCTCAGCGACGGGTCTTGGTCTACCGTGAGCGAGGAAGCTAGTGAGGACGTCGTCTGCTGC

>HM106826

TCCGGCTCGTGGCTAAGGGACGTTTGGGACTGGATATGCACGGTGTTAAGTGATTTTAAGACCTGGCTCCAGTCCAAGCTCCTGCCGCAGTTACCGGGAGTCCCCTTTCTCTCATGCCAACGTGGGTACAAGGGCATCTGGCGGGGAGACGGCATCATGCATACCACCTGCCCATGTGGAGCGCAGATCACCGGACATGTCAAAAATGGTTCCATGAGGATCGTTGGGCCTAGGACCTGCAGCAACACGTGGCATGGAACATTCCCCATCAACGCATACACCACGGGCCCCTGCACGCCCWCCCCGGCGCCCAATTATTCCAGGGCGCTGTGGAGGGTGWCTGCTGAGGAGTACGTGGAGGTTGYGCGGGTGGGGGATTTCCACTACGTGACGGGCATGACCACTGACAACGTAAARTGCCCATGCCAGGTTCCAGCCCCCGAATTCTTCACGGAGGTGGATGGGGTGCGGTTGCACAGGTACGCTCCGCCGTGCAAACCTCTCCTACGGGAGGAGGTCACATTCCAGGTCGGGCTCAACCAATACCTGGTCGGGTCACAGCTCCCGTGTGAGCCCGAACCGGAYGTRGCGGTGCTCACTTCCATGCTCACCGACCCCTCCCACATYACAGCAGAGRCGGCTAAGCGTAGGCTGGCCAGGGGGTCTCCCCCCTCCTTGGCCAGCTCTTCAGCTAGCCAGYTATCTGCGCCTTCCTTGAAGGCAACATGCACTACYCGTCATGACTCCCCAGATGCTGACCTCATCGAGGCCAATCTCCTGTGGCGGCAGGAGATGGGCGGGAACATCACCCGCGTGGAGTCAGAGAACAAAGTAGTAATTCTGGACTCTTTCGAGCCGCTTCGAGCGGAGGAAGATGAGAGGGAAGTATCCGTTCCGGCGGAAATCCTGCGGACATCCAGGAAATTCCCCCGRGCGATGCCCATATGGGCAAGGCCGGATTACAACCCTCCACTGRTAGAGTCCTGGAAGGACCCGGACTACGTCCCTCCGGTGGTRCATGGGTGCCCATTGCCACCTACTAAGGCCCCTCCAATACCACCTCCACGGAGGAAAAGGACGGTTGTCCTGACAGAATCCACCGTGTCTTCTGCCTTGGCTGAGCTCGCCACAAAGACCTTCGGCAGTTCCGAATCGTCGGCCGTCGACAGCGGCACGGCGACCGCCCCTCCTGACCAGCCCTNNNNNNNNNNNNNNNNNNNNNNNNNNNNNNNNNNNNNNNNNNNNNNNNNNNNNNNNNNNNNNNNNNNNNNNNNNNNNNNNNNNNNNNNNNNNNNNNNNNNNNNNNNNNNNNNNNNNNNNNNNNNNNNNNNNNNNNNNNNN

>HM106827

NNNNNNNNNNNNNNNNNNNNNNNNNNNNNNNNNNNNNNNNNNNNNNNNNNNNNNNNNNNNNNNNNNNNNNNNNNNNNNNNNNNNNNNNNNNNNNNNNNNNNNNNNNNNNNNNNNNNNNNNNNNNNNNNNNNNNNNNNNNNNNNNNNNNNNNNNNNNNNNNNNNNNNNNNNNNNNNNNNNNNNNNNNNNNNNNNNNNNNNNNNNNNNNNNNNNNNNNNNNNNNNNNNNNNNNNNNNNNNNNNNNNNNNNNNNNNNNNNNNNNNNNNNNNNNNACGCATACACCACGGGCCCTTGCACGCCCTCCCCGGCGCCCAATTATTCCAAGGCGCTGTGGAGGGTGGCTGCTGAGGAGTACGTGGAGGTTACGCGGGTGGGGGATTTCCACTACGTGACGGGCATGACCACTGACAGTGTCAAGTGCCCATGCCAGGTTCCGGCCCCCGAATTCTTCACGGAGGTGGATGGGGTGCGGCTGCACAGGTACGCTCCACCATGCAAACCTCTCCTACGGGAGGAGGTCACRTTCCAGGTCGGGCTCAACCAGTACCTGGTCGGGTCACAGCTCCCATGTGAGCCCGAACCGGATGTAGCGGTGCTCACTTCCATGCTCACCGACCCCTCCCACATCACAGCAGAGACAGCTAAGCGTAGGCTGGCTAGGGGGTCTCCCCCCTCCTTAGCCAGCTCTTCAGCTAGCCAGTTGTCTGCGCCTTCCTTGAAGGCGACATGCACTACCCGTCATGACTCCCCAGACGCTGACCTCATCGAGGCCAATCTCCTGTGGCGGCAGGAGATGGGCGGGAACATCACCCGCGTGGAGTCAGAGAACAAGGTAGTAATTCTGGACTCTTTCGAGCCGCTTCGAGCGGAGGAAGACGAGAGGGAAGTATCCGTTCCGGCGGAAATCCTGCGGAAATCCAGGAAATTCCCTCGAGCGATGCCCATATGGGCAAGGCCAGACTACAACCCTCCACTGGTGGAGTCCTGGAAGGACCCGGACTACGTCCCTCCGGTGGTACATGGGTGCCCGTTGCCACCTATTAGGGCCCCTCCAATACCACCCCCACGAAGGAAGAGGACGGTTGTCCTGACAGAATCCACCGTGTCTTCTGCCTTGGCCGAGCTCGCCACAAAGACCTTCGGCAGCTCCGAGTCGTCGGCCGTCGACAGCGGCACGGCGACCGCCCCTCCTGACCAGCCCTCCGACGACGGCGACGCAGGGTCCGATGTTGAGTCGTACTCCTCCATGCCCCCCCTTGAGGGGGAGCCGGGGGATCCCGACCTCAGCGACGGGTCCTGGTCTACCGTGAGCGAGGAAGCTAGTGAGGACGTCGTCTGCTGC

>HM106828

TCCGGCTCGTGGCTAAGGGACGTTTGGGACTGGATATGCACGGTGTTAACTGATTTTAAGACCTGGCTCCAGTCCAAGCTCCTGCCGCGGTTACCGGGGGTCCCTTTCTTCTCATGCCAACGTGGGTACAGAGGCATCTGGCGGGGAGACGGCATCATGCAAACCACCTGCCCATGTGGAGCGCAGATCACCGGACATGTCAAAAACGGTTCCATGAGGATCGTCGGGCCTAGGACCTGCAGCAACACGTGGCATGGRACATTCCCCATCAACGCATACACCACGGGCCCCTGCACGCCYTCCCCGGCGCCCAATTATTCCAGGGCGCTKTGGAGGGTGGCTGCTGAGGAGTACGTGGAGGTTRYGCGGGTGGGGGACTTCCACTACGTGACGGGCATGACCACTGACAACGTAAAGTGCCCATGCCAGGTTCCGGCCCCCGARTTCTTCACGGAGGTGGATGGGGTGCGGCTGCACAGGTACGCTCCGSCGTGCARACCYCTCCTACGGGAGGAGGTCACATTCCAGGTCGGGCTCAACCAATACCTGGTTGGGTCACAGCTCCCATGYGAGCCYGAACCGGATGTRGCGGTGCTCACTTCCATGCTCACCGACCCCTCCCACATCACAGCAGAGACGGCTAAGCGTAGGCTGGCCAGGGGGTCTCCCCCCTCCTTGGCCAGCTCTTCAGCTAGCCAGTTGTCTGCGCCTTCCTTGAAGGCGACATGCACTACCCGTCATGACTCCCCAGATGCTGACCTCATCGAGGCCAATCTCCTGTGGCGGCAGGAGATGGGCGGGAACATCACCCGCGTGGAGTCAGAGAACAAGGTAGTAATTCTGGACTCTTTCGARCCGCTKCGAGCGGAGGAAGAYGAGAGGGAAGTATCCGTTCCGGCGGAAATCCTGCGGARATCCAGGAAATTTCCCCGAGCGATGCCCATATGGGCAAGGCCGGATTACAACCCTCCACTGGTRGAGTCCTGGAAGGACCCGGACTACGTCCCTCCGGTGGTGCATGGGTGCCCATTRCCACCTACTAAGGCCCCTCCRATACCACCTCCACGGAGGAAAAGGACGGTTGTCCTGACAGAATCCACCGTGTCYTCTGCCTTGGCTGAGCTCGCCACAAARACCTTCGGCAGCTCCGAATCGTCGGCCGTCGACAGCGGCACGGCGACCGCTCCTCCTGACCAGCCCTCCGACGACGGCGACGCNNNNNNNNNNNNNNNNNNNNNNNNNNNNNNNNNNNNNNNNNNNNNNNNNNNNNNNNNNNNNNNNNNNNNNNNNNNNNNNNNNNNNNNNNNNNNNNNNNNNNNNNNNNNNNNNNNNNNNNNNNNN

>HM106829

TCCGGCTCGTGGCTAAGGGAYGTTTGGGACTGGATATGCACGGTGTTRACYGACTTYAAGACCTGGCTCCAGTCCAAGCTCCTGCCGCGGTTGCCGGGAGTCCCCTTCTTCTCATGCCAACGTGGGTACAAGGGCATCTGGCGGGGAGACGGCATCATGCAAACCACCTGCCCATGTGGAGCGCAGATCACCGGACATGTCAAAAACGGTTCCATGAGGATCGTTGGGCCTAGGACCTGCAGCAACACGTGGCATGGAACATTCCCCATCAACGCRTACACCACGGGCCCCTGCACGCCCTCCCCGGCGCCCAATTATTCCAGGGCGCTGTGGAGGGTGACTGCTGAGGAGTACGTGGAGGTTACGCGGGTGGGGGATTTCCACTACGTGACGGGCATGACCACTGACAACGTAAAGTGCCCATGTCAGGTTCCGGCTCCCGAATTCTTCACGGAGGTGGATGGGGTGCGGCTGCACAGGTACGCTCCGCCGTGCAAACCCCTCCTGCGGGAGGAGGTCACATTCCAGGTCGGGCTCAACCAATACCTGGTCGGGTCACAGCTCCCATGCGAGCCCGAACCGGATGTAGCGGTGCTCACTTCCATGCTCACCGACCCCTCCCACATCACAGCAGAGGCGGCTAAGCGTAGGCTGGCCAGGGGGTCTCCCCCCTCCTTGGCCAGCTCTTCAGCTAGCCAGTTGTCTGCGCCTTCCTTGAAGGCGACATGCACTACCCGTCATGACTCCCCAGATGCTGACCTCATCGAGGCCAATCTCCTGTGGCGGCAGGAGATGGGCGGGAACATCACCCGCGTGGAGTCGGAGAACAAGGTAGTAATTCTGGACTCTTTCGAGCCGCTTCGAGCGGAGGAAGATGAGAGGGAAGTATCCGTTCCGGCGGAAATCCTGCGGAGATCCAGGAAATTTCCCCGAGCGATGCCCGTATGGGCAAGGCCGGATTACAACCCTCCACTGGTGGAGTCCTGGAAGGACCCGGACTACGTCCCTCCGGTGGTACATGGGTGCCCATTGCCACCTACTAAGGCCCCTCCAATACCACCTCCACGGAGGAAAAGGACGGTTGTCCTGACAGAATCCACCGTGTCCTCTGCCTTGGCTGAGCTCGCCACAAAGACCTTCGGCAGCTCCGAATCGTCGGCCGTCGACAGCGGCACGGCAACCGCCCCTCCTGACCAGCCCTCCGACGACGGCGACRCAGGGTCCGACGTTGAGTCGTACTCCTCCATGCCCCCCCTTGAGGGGGAGCCGGGGGATCCCGATCTCAGCGACGGGTCTTGGTCCACCGTGAGCGAGGARGCYAGTGAGGACGTCGTCTGCTGC

>HM106830

TCCGGCTCGTGGCTAAGGGACGTTTGGGACTGGATATGCACGGTGTTAACTGATTTTAAGACCTGGCTCCAGTCCAAGCTCCTGCCGCAGTTGCCGGGAGTTCCCTTCYTCTCATGCCAACGTGGGTACAAGGGTATCTGGCGGGGAGAYGGCATCATGCAAACCACCTGCCCATGTGGAGCGCAGATCACCGGACATGTCAAAAMCGGTTCCATGAGGATCNNNNNNNNNNNNNNNNNNNNNNNNACGTGGCATGGAACATTCCCCATCAACGCRTACACCACGGGCCCCTGCACGCCCTCCCCGGCGCCCRATTATTCCAGGGCGCTGTGGAGGGTGGCTGCTGAGGAGTACGTGGARGTTACGCGGGTRGGGGATTTCCACTACGTGACGGGCATGACCACTGACAACGTRAARTGCCCATGCCAGGTTCCGGCCCCCGAATTCTTCACGGAGGTGGATGGGGTGCGGCTGCACAGGTAYGCTCCGCCGTGCARACCTCTCCTACGGGTGGAGGTCACATTCCRGGTCGGGCTCAACCAATAYCTGGTCGGGTCACAGCTCCCATGYGAGCCCGAACCGGATGTAGCGGTGYTCACTTCCATGCTCACCGACCCCTCCCACATCACAGCAGAGGCGGCTAAGCGYAGGCTGGCCAGGGGGTCTCCCCCCTCCTTGGCCAGCTCTTCAGCTAGCCAGTTGTCTGCGCCTTCCYTGAARGCGACATGCACTACCCGTCAYGACTCCCCAGATGCTGACCTCATCGAGGCCAATCTCCTGTGGCGACAGGAGATGGGCGGGAACATCACCCGCGTGGAGTCAGAGAACAAGGTAGTAATTCTGGACTCTTTCGAGCCGCTTCGAGCGGAGGAAGATGAGAGGGAAGTATCCGTTCCGGCGGAAATCCTGCGGAGATCCAGGAAATTTCCCCRAGCKATGCCCRTATGGGCAAGGCCGGATTACAACCCTCCACTGGTRGAGTCCTGGAAGGACCCGGACTATGTCCCCCCGGTGGTRCACGGGTGCCCATTGCCACCTRCTAMGRCCCCTCCRATACCACCTCCACGGAGGAAAAGGACGGTTGTCCTGACAGAATCCACCGTGTCTTCTGCCTTGGCTGAGCTCGCCACAAAGACCTTCGGCAGCTCYGAATCGTCGGCCGTCGACAGCGGCACGGCGACCGCCCCTNNNNNNNNNNNNNNNNNNNNNNNNNNNNNNNNNNNNNNNNNNNNNNNNNNNNNNNNNNNNNNNNNNNNNNNNNNNNNNNNNNNNNNNNNNNNNNNNNNNNNNNNNNNNNNNNNNNNNNNNNNNNNNNNNNNNNNNNNNNNNNNNNNNNNNNNN

>HM106831

TCCGGCTCGTGGCTAAGGGAYGTTTGGGACTGGATATGYACGGTGTTAACTGATTTTAAGACCTGGCTCCAGTCCAAGCTCCTGCCGCGGTTRCCGGGAGTCCCCTTCCTCTCATGCCAACGAGGGTACAAGGGCATCTGGCGGGGAGACGGCATCATGCAMACCACCTGCCCATGTGGAGCGCAGATCACCGGACATGTCAAAAATGGTTCCATGAGGATCGTTGGGCCTAGGACCTGCAGCAACNNNNNNNNNNNNNCATTCCCCATCAACGCGTACACCACGGGCCCCTGCACGCCCTCCCCGGCGCCCAATTATTCCAGGGCGCTGTGGAGGGTGWCTGCTGAGGAGTACGTGGAGGTTACGCGGGTGGGGGATTTCCACTACGTGACGGGCATGACCACTGACAACGTAAAGTGCCCATGCCAGGTCCCGGCCCCCGAATTCTTCACGGAGGTGGATGGGGTGCGCCTGCACAGGTACGCTCCGCCGTGCAAACCTCTCCTACGGGAGGAGGTCACATTCCAGGTCGGGCTCAACCAATACCTGGTCGGGTCACAGCTCCCATGTGARCCCGAACCGGATGTAGCGGTGCTCACTTCCATGCTAACCGACCCCTCCCACATCACAGCAGAGGCGGCTAAGCGTAGGCTGGCCAGAGGGTCTCCCCCCTCCTTAGCCAGCTCTTCAGCTAGCCAGTTGTCTGCGCCTTCCTTGAAGGCGACATGCACTACCCATCATGACTCCCCTGATGCTGACCTCATCGAGGCCAATCTTCTGTGGCGGCAGGAGATGGGCGGGAACATCACCCGCGTGGAGTCAGAGAACAAGGTAGTAATTCTGGACTCTTTCGAGCCGCTTCGAGCGGAGGAAGATGAGAGGGAAGTATCCGTCCCGGCAGAGATCCTGCGAAGATCCAGGAAATTTCCCCGGGCGATGCCCATATGGGCAAGGCCGGATTACAACCCTCCACTGGTAGAGTCCTGGAAGGACCCGGACTACGTCCCTCCGGTGGTACATGGGTGCCCATTGCCACCTGCTAAGGCCCCTCCAATACCACCTCCACGGAGGAAAAGGACGGTTGTCCTGACAGAATCCACCGTGTCTTCTGCCTTGGCTGAGCTCGCCACAAAGACCTTCGGCAGCTCCGAATCATCGGCCGTCGACAGCGGCACGGCGACCGCCCCTCCTGACCAGCCCTCCGANNNNNNNNNNNNNNNNNNNNNNNNNNNNNNNNNNNNNNNNNNNNNNNNNNNNNNNNNNNNNNNNNNNNNNNNNNNNNNNNNNNNNNNNNNNNNNNNNNNNNNNNNNNNNNNNNNNNNNNNNNNNNNNNNNNNNNNN

>HM106832

TCCGGCTCGTGGCTAAGGGACGTTTGGGACTGGATATGCACGGTGTTAACTGATTTTAAGACCTGGCTCCAGTCTAAGCTCCTGCCGCGGTTACCGGGAATCCCCTTCCTCTCATGCCAACGTGGGTACAAAGGCATCTGGCGGGGAGACGGCATCATGCAAACCACCTGCCCATGTGGANNNNNNNNNNNNNNNNNNNNNNNNNNNNNNNNNNNNNNNNNNNNNNNNNNNNNNNCCTGCAGCAACACGTGGCATGGGACATTCCCCATCAACGCATACACCACGGGCCCCTGCACGCCCTCCCCGGCGCCCAATTATTCCAGGGCGCTGTGGAGGGTGGCTGCTGAGGAGTACGTGGAGGTTACGCAGGTGGGGGATTTCCACTACGTGACGGGCATAACCACTGACAACGTAAAGTGCCCATGCCAGGTTCCGGCCCCCGAATTCTTCACGGAGGTGGATGGGGTGCGGCTGCACAGGTACGCTCCGCCGTGCAGACCTCTCCTACGGGAGGACGTCACATTCCAGGTCGGGCTCAACCRATACCTGGTCGGGTCACAGCTCCCATGTGAGCCCGAACCGGATGTAGCGGTGCTCACTTCCATGCTCACCGACCCCTCCCACATTACAGCAGAGACGGCTAAGCGTAGGCTAGCCAGGGGGTCTCCCCCCTCCTTGGCCAGCTCTTCAGCTAGCCAGTTGTCTGCGCCTTCCTTGAAGGCAACATGCACTACCCGTCATGACTCTCCAGATGCTGACCTCATCGAGGCCAATCTCCTGTGGCGGCAGGAGATGGGCGGGAACATCACCCGCGTGGAGTCAGAGAATAAGGTAGTAATCCTGGACTCTTTCGAGCCGCTTCGAGCGGAGGAAGATGAGAGGGAAGTATCCGTTCCGGCGGAAATCCTGCGGAGATCCAGGAAATTTCCCCGAGCGATGCCCATATGGGCAAGGCCGGATTACAACCCTCCACTGGTAGAGCCCTGGAAGGACCCGGACTACGTCCCTCCGGTGGTACATGGGTGCCCGTTGCCACCTACTAAGGCCCCTCCAATACCACCTCCACGGAGGAAAAGGACGGTTGTCTTGACAGAATCTACCGTGTCCTCTGCCTTGGCTGAGCTCGCCACAAAGACCTTCGGCAGCTCCGACTCGTCGGCCGTCGACAGCGGCACGGCGACCGCCCCTCCTGACCAGGCCTCCNNNNNNNNNNNNNNNNNNNNNNNNNNNNNNNNNNNNNNNNNNNNNNNNNNNNNNNNNNNNNNNNNNNNNNNNNNNNNNNNNNNNNNNNNNNNNNNNNNNNNNNNNNNNNNNNNNNNNNNNNNNNNNNNNNNNNNNNNN

>HM106833

NNNNNNNNNNNNNNNNNNNNNNNNNNNNNNNNNNNNNNNNNNNNNNNNNNNNNNNNNNNNNNNNNNNNNNNNNNNNNNNNNNNNNNNNNNNNNNNNNNNNNNNNNNNNNNNNNNNNNNNNNNNNNNNNNNNNNNNNNNNNNNNNNNNNNNNNNNNNNNNNNNNNNNNNNNNNNNNNNNNNNNNNNNNNNNNNNNNNNNNNNNNNNNNNNNNNNNNNNNNNNNNNNNNNNNNNNNNNNTGCAGCAACACGTGGCATGGAACATTCCCCATCAACGCATACACCACGGGCCCCTGCACGCCCTCCCCGGCGCCCAATTATTCCAGGGCGCTGTGGAGGGTGGCTGCTGAGGAGTACGTGGAGGTTACGCGGGTGGGGGATTTCCACTACGTGACGGGCATGACCACTGACAACGTAAAGTGCCCATGCCAGGTTCCGGCCCCCGAATTCTTCACGGAGGTGGATGGGGTGCGGCTGCACAGGTAYGCYCCGCCRTGCAAACCTCTCCTACGGGAGGAGGTCACATTCCAGGTCGGGCTCAACCAATACCTGGTCGGGTCACAGCTCCCATGTGAGCCCGAACCGGATGTAGCGGTGCTCACTTCCATGCTCACCGACCCCTCCCAYATCACAGCAGAGGCGGCTAAGCGTAGGCTGGCYAGRGGGTCWCCCCCCTCCTTAGCCAGCTCTTCAGCTAGCCAGTTGTCTGCGCCTTCCTTGAAGGCGACATGCACTACCCGTCATGACTCCCCAGATGCTGACCTCATCGAGGCCAATCTCCTGTGGCGGCAGGAGATGGGCGGGAACATCACCCGCGTGGAGTCAGAGAACAAGGTRGTAATTCTGGACTCTTTCGAGCCGCTKCGAGCGGAGGAAGATGAGAGGGAAGTATCCGTTCCRGCGGAAATCCTGCGGAGATCCAGGAAATTCCCYCGAGCGATGCCCATATGGGCAAGGCCRGATTACAACCCTCCACTGGTGGAGTCCTGGAAGGACCCGGAYTACGTCCCTCCRGTGGTACATGGGTGCCCATTGCCACCTACTAAGGCCCCTCCAATACCACCTCCACGGAGGAAAAGGACGGTTGTCCTGACAGAATCCACCGTGTCTTCTGCCTTGGCCGAGCTCGCCACAAAGACCTTYGGCRGCTCCGAATCGTCGGCCGTCGACAGCGGCACGGCGACCGCYCCTCCTGACCAGTCCTCCNNNNNNNNNNNNNNNNNNNNNNNNNNNNNNNNNNNNNNNNNNNNNNNNNNNNNNNNNNNNNNNNNNNNNNNNNNNNNNNNNNNNNNNNNNNNNNNNNNNNNNNNNNNNNNNNNNNNNNNNNNNNNNNNNNNNNNNNNN

>HM106834

TCCGGCTCGTGGCTAAGGGACGTTTGGGACTGGATATGCACGGTGTTAWCYGAYTTTAAGACCTGGCTCCAGTCCAAGCTCCTGCCGCAGTTACCGGGARTCCCCTTCTTCTCATGCCAACGTGGGTACAAGGGCATCTGGCGGGGAGACGGCATCATGCAYACCACCTGCCCATGTGGAGCGCAGATCACCGGACATGTCAAAAACGGTTCCATGAGGATCGTTGGGCCTAGAACCTGTAGCAACACGTGGCNNNNNNNNTTCCCCATCAACGCATACACTACGGGCCCCTGCACGCCCTCTCCAGCGCCCAATTATTCCAGGGCGCTGTGGAGGGTGGCTGCTGAGGAGTACGTGGAGGTTGCGCGGGTGGGGGATTTCCACTACGTGACGGGCATGACCACTGACAACGTAAAGTGCCCATGCCAGGTTCCGGCCCCCGAATTCTTCACAGAGGTGGATGGGGTRCGGCTGCACAGGTAYGCTCCGCCGTGCAGGCCTCTCCTACGGGAGGAGGTCACATTCCAGGTCGGGCTCAACCAATACCTGGTCGGGTCACAGCTCCCGTGTGAGCCCGAACCGGATGTAGCGGTGCTCACTTCCATGCTCACCGACCCCTCCCACATCACAGCAGAGACGGCTAAGCGTAGGCTGGCCAGGGGGTCTCCCCCCTCCTTAGCCAGCTCTTCAGCTAGCCAGTTGTCTGCGCCYTCCTTGAAGGCGACATGCACTACCCATCATGACTCCCCAGATGCTGACCTCATCGAGGCCAATCTCCTGTGGCGGCAGGAGATGGGCGGGAACATCACCCGCGTGGAGTCAGAGAACAAGGTAGTAATTCTGGACTCTTTCGAGCCGCTTCGAGCGGAGGAAGATGAGAGGGAAGTATCCGTTCCGGCGGAAATCCTGCGGAGATCCAGGAAATTYCCCCGAGCGATGCCCATATGGGCAAGGCCGGATTACAACCCTCCACTGGTGGAGTCCTGGAAGGACCCGGACTACGTCCCTCCAGTGGTACATGGGTGCCCATTGCCACCTACTAAGGCCCCTCCAATACCACCTCCACGGAGGAAAAGGACGGTTGTCCTGACAGAATCCACCGTGTCCTCTGCCTTGGCTGAGCTCGCCACAAAGACCTTCGGCAGCTCCGAATCGTCGGCCGTCGACAGNNNNNNNNNNNNNNNNNNNNNNNNNNNNNNNNNNNNNNNNNNNNNNNNNNNNNNNNNNNNNNNNNNNTACTCCTCCATGCCCCCCCTTGAGGGGGAGCCGGGGGATCCCGATCTCAGCGACGGGTCTTGGTCTACCGTGAGCGAGGAAGCTAGTGAGGACGTCGTCTGCTGC

>HM106835

TCCGGCTCGTGGCTAAGGGACGTTTGGGACTGGATATGCACGGTGTTAACTGATTTTAAGACCTGGCTCCAGTCCAAGCTCCTGCCGCGGTTACCGGGACTCCCCTTCCTCTCATGCCAACGTGGATACAAGGGCATTTGGCGGGGAGACGGCATCATGCAAACCACCTGCCCATGTGGTGCGCAGATCACCGGACATGTCAAAAACGGTTCCATGAGGATCGTCGGGCCTAGGACCTGCAGCAACACGTGGCATGGRACATTCCCCATCAACGCATACACCACGGGCCCCTGCACGCCCTCCCCGGCGCCCAATTATTCCARGGCGCTGTGGAGRGTGACTGCTGAGGAGTACGTGGAGGTTACGCGGGTGGGGGATTTCCACTACGTGACGGGCATGACCACTGACAACGTAAAGTGCCCATGCCAGGTTCCGGCCCCTGAATTCTTCACGGAGGTGGATGGGGTGCGACTGCACAGGTACGCTCCRCCGTGCAAACCTCTCCTACGGGAGGAGGTCACATTCCAGGTCGGGCTCAACCAATATTTGGTCGGGTCACAGCTCCCATGCGAGCCCGAACCGGATGTRRCGGTGCTCACTTCCATGCTCACCGACCCCTCCCACATCACAGCAGAGGCGGCTAAGCGTAGGYTRGCCAGGGGGTCTCCCCCCTCCTTAGCCAGCTCYTCAGCTAGCCAGYTGTCTGCGCCTTCCTTGAAGGCGACATGCACTACCCGTCATGACTCCCCAGATGCTGACCTCATCGAGGCCAATCTCCTGTGGCGGCAGGAGATGGGCGGGAACATCACCCGYGTGGAGTCAGAGAACAAGGTAGTGATTCTGGACTCTTTCGAGCCGCTTCGAGCGGARGAAGATGAGAGGGAAGTATCTGTTCCGGCGGAAATCCTGCGGAGATCYAGGAAATTTCCCCGAGCGCTGCCCATATGGGCGAGGCCGGATTACAACCCACCACTGGTAGAGTCCTGGAARGACCCGGACTAYGCCCCYCCGGTGGTACATGGGTGCCCRTTGCCGCCTACTARGGCCCCTCCGATACCACCTCCRCGGAGGAAAAGGACGGTTGTCCTAACAGAATCCACCGTGTCTTCTGCTTTGGCTGAGCTCGCCACAAAGACCTTCGGCAGCTCMGAATCGTCGGCCRTCGACAGCGGCACGGCGACCGCCCCTCCTGRCCAGTCCTCCGACGNNNNNNNNNNNNNNNNNNNNNNNNNNNNNNNNNNNNNNNNNNNNNNNNNNNNNNNNNNNNNNNNNNNNNNNGATCTCAGCGACGGGTCTTGGTCTACCGTGAGCGAGGAAGCYAGTGAGGACGTCGTCTGCTGC

>HM106836

NNNNNNNNNNNNNNNNNNNNNNNNNNNNNNNNNNNNNNNNNNNNNNNNNNNNNNNNNNNNNNNNNNNNNNNNNNNNNNNNNNNNNNNNNNNNNNNNNNNNNNNNNNNNNNNNNNNNNNNNNNNNNNNNNNNNNNNNNNNNNNNNNNNNNNNNNNNNNNNNNNNNNNNNNNNNNNNNNNNNNNNNNNNNNNNNNNNNNNNNNNNNNNNNNNNNNNNNNNNNNNNNNGGCCCTAGGACCTGCAGCAACACGTGGCATGGAACATTCCCCATCAATGCGTACACCACCGGCCCCTGCACGCCCTCCCCGGCGCCTAATTATACCAAGGCGCTGTGGAGGGTGGCTGCTGAGGAGTACGTGGAGGTTACGCAGGTGGGGGATTTCCACTACGTGACGGGCATGACCACTGACAACGTAAAGTGCCCATGCCAGGTTCCGGCCCCCGAATTCTTCACGGAGGTGGATGGGGTGCGGCTGCACAGGTACGCTCCGCCGTGCAGACCTCTCCTACGGGAGGAGGTCACATTCCAGGTCGGGCTCAACCAATACCTGGTCGGGTCACAGCTCCCATGTGAACCCGAGCCGGACGTAGCGGTGCTCACTTCCATGCTCACCGACCCCTCCCACATCACGGCAGAGACGGCTAAGCGTAGGCTGGCCAGGGGGTCTCCCCCCTCCTTGGCCAGCTCCTCAGCTAGCCAGTTGTCTGCGCCTTCCTTGAAAGCGACATGCACTGCCCGTCATGACTCCCCAGATGCTGACCTCATCGAGGCCAATCTCCTGTGGCGGCAGGAGATGGGCGGGAACATCACCCGCGTGGAGTCAGAGGACAAGGTAGTAATTCTGGACTCTTTCGAGCCGCTTCGAGCGGAGGAAGATGAGAGGGAAGTATCCGTTCCGGCGGAAATCCTGCGGAGATCCAAGAAATTTCCCCGAGCGATGCCCATATGGGCAAGGCCGGATTACAACCCTCCACTGGTAGAGTCCTGGAAGGACCCGGACTACGTCCCTCCGATGGTACATGGGTGCCCACTGCCGCCTACCAAGGCCCCTCCAATACCACCTCCACGGAGAAAAAGGACGGTTGTCCTGACAGAGTCMACYGTGTCTTCTGCCTTGGCTGAGCTCGCCACAAAGACCTTCGGCAGCTCCGGATCGTCGGCTGTTGACAGCGGCACGGCGACCGCCCCTCTTGACCAGCCCTCCGATGACGGCGACGCAGGGTCCGATGTTGAGTCGTACTCCTCCATGCCCCCCCTTGAGGGGGAGCCGGGGGATCCCGATCTCAGCGACGGGTCCTGGTCTACCGTGAGCGAGGAAGCTAGTGAGGACGTCGTCTGTTGC

>HM106837

NNNNNNNNNNNNNNNNNNNNNNNNNNNNNNNNNNNNNNNNNNNNNNNNNNNNNNNNNNNNNNNNNNNNNNNNNNNNNNNNNNNNNNNNNNNNNNNNNNNNNNNNNNNNNNNNNNNNNNNNNNNNNNNNNNNNNNNNNNNNNNNNNNNNNNNNNNNNNNNNNNNNNNNNNNNNNNNNNNNNNNNNNNNNNNNNNNNNNNNNNNNNNNNNNNNNNNNNNNNNNNNNNGGGCCTAGGACYTGCAGCAACACGTGGCATGGAACATTCCCCATCAACGCAYACACCACGGGCCCCTGCACGCCCTCCCCGGCGCCCAATTATTCCAGGGCGCTGTGGAGGGTGGCTGCYGAGGAGTACGTGGAGGTTACGCGGGTGGGGGATTTCCACTACGTGACGGGCATGACCACTGACAACGTAAAGTGCCCATGCCAGGTTCCRGCCCCCGAATTCTTCACGGAGGTGGATGGGGTGCGGCTGCACAGGTACGCYCCRCCGTCCAAACCTCTCCTACGGGAGGAGGTCACMTTCCAGGTCGGGCTCAACCAATACCTGGTCGGGTCACAGCTCCCATGTGAGCCCGARCCGGATGTAGCGGTGCTCACYTCCATGCTCACCGACCCCTCCCACATCACAGCAGAGGCGGCTAAGCGTAGGCTGGCCAGGGGGTCWCCCCCCTCCTTRGCCAGCTCTTCAGCTAGCCAGTTGTCTGCGCCTTCCTTGAAGGCGACATGCACTACCCGTCATGACTCCCCAGATGCTGACCTCATCGAGGCCAATCTCCTGTGGCGGCAGGAGATGGGCGGGAACATCACCCGCGTGGAGTCAGAGAACAAGGTAGTAATYCTGGACTCTTTCGAGCCGCTTCGAGCGGAGGAAGATGAGAGGGAAGTATCCGTTCCGGCGGAAATCCTGCGGAGATCCAGGAAATTTCCCCGAGCGATGCCCATATGGGCAAGGCCGGATTACAACCCTCCACTGATAGAGYCCTGGAAGGACCCGGACTACGTCCCTCCGGTGGTACATGGGTGCCCATTRCCACCTRCTAAGGCCCCTCCAATACCACCTCCACGGAGGAAAAGGACGGTTGTCCTGACAGAATCCACCGTGTCTTCTGCCTTGGCTGAGCTCGCYACGAAGACCTTCGGCAGCTCCGAATCGTCGGYCGYCGACAGCGGCACGGCGACCGCCCCTCCTGACCAGNNNNNNNNNNNNNNNNNNNNNNNNNNNNNNNNNNNNNNNNNNNNNNNNNNNNNNNNNNNNNNNNNNNNNNNNNNNNNNNNNNNNNNNNNNNNNNNNNNNNNNNNNNNNNNNNNNNNNNNNNNNNNNNNNNNNNNNNNNNNNNNN

>HM106838

TCCGGCTCGTGGCTGAGGGACGTTTGGGACTGGATATGCACGGTGTTAACTGATTTTAAGACCTGGCTCCAGTCCAAGCTCCTGCCGCGGTTACCGGGAGTTCCCTTCCTCTCATGCCAACGTGGGTACAAGGGCGTCTGGCGAGGAGACGGCATCATGCAAACCACCTGCCCATGTGGAGCGCAGATCACCGGACATGTCAAAAACGGTTCCATGAGGATNNNNNNNNNNNNNNNNNNNNNNNNNNNNNNNNNNNNNNNNNNNNNNNNNNNCGCATACACCACGGGCCCCTGCACGCCCTCCCCGGCGCCCAATTATTCCARGGCGCTGTGGAGGGTGKCYGCTGAGGAGTACGTGGAGGTYACGCGGGTGGGGGATTTCCACTACGTGACGGGCAYGACCACTGACAACGTAAAGTGCCCATGCCAGGTTCCGGCCCCCGAATTCTTCACRGAGGTGGATGGGGTGCGGCTGCACAGGTACGCYCCGCCGTGCAAACCTCTCCTACGGGAGGAGGTCACATTCCRGGTCGGGCTCAACCAATACCTGGTCGGGTCACAGCTCCCATGTGAGCCCGAACCGGATGTAGCGGTGCTCACTTCCATGCTCACCGACCCCTCCCACATYACAGCAGAGGCGGCTRRGCGTAGGCTGGCCAGGGGGTCTCCCCCCTCCTTGGCCAGCTCTTCAGCTAGCCAGTTGTCTGCGCCTTCCTTGAAGGCGACATGCACTACCCGTCATGACTCCCCAGATGCYGACCTCATCGAGGCCAATCTCCTGTGGCGGCAGGAGATGGGCGGGAACATCACCCGCGTGGAGTCAGAGAACAAGGTAGTAATTCTGGACTCTTTCGAGCCGCTTCGAGCGGARGAAGATGAGAGGGAAGTATCCGTTCCGGCGGAAATCCTGCGGAGATCCAGGAARTTTCCCCGGGCGATGCCCATATGGGCAAGGCCGGATTACAACCCTCCACTGGTAGAGTCCTGGAAGGACCCGGACTACGTCCCTCCGGTGGTACATGGGTGCCCRTTGCCACCTACTAAGGCCCCTCCAATACCACCTCCACGGAGGAARAGGACGGTTGTCCTRACAGAATCCACCGTGTCTTCTGCCTTGGCTGAGCTCGCCACGAAGACCTTCGGCAGCTCTGAATCGTCGGCCNNNNNNNNNNNNNNNNNNNNNNNNNNNNNNNNNNNNNNNNNNNNNNNNNNNNNNNNNNNNNNNNNNNNNNNNNNNNNNNNNNNNNNNNNNNNNNNNNNNNNNNNNNCGGGGGATCCCGATCTCAGCGACGGGTCTTGGTCTACCGTGAGYGAGGAAGCTRGTGAGGACGTCGTCTGCTGC

>HM106839

TCCGGCTCGTGGCTAAGGGACGTTTGGGACTGGATATGCACGGTGTTAAGTGATTTTAAGACCTGGCTCCAGTCCAAGCTCCTGCCGCGGTTACCGGGAGTCCCCTTCYTCTCATGCCAACGTGGGTACAAGGGCGTCTGGCGGGGAGACGGCATCATGCAAACCACCTGCCCATGTGGAGCGCAGATCACCGGACATGTCAAAAACGGTTCCATGAGGATCGTTGGCCCTAGGACCTGCAGCAACACGTGGCATGGRACATTCCCCATCAACGCRTACACCACGGGCCCCTGCACGCCCTCCCCGGCGCCCAATTATTCCAGGGCGCTGTGGAGGGTGTCTGCTGAGGAGTACGTGGAGGTTACGCGGGTGGGGGATTTCCACTACGTGACGGGCATGACCACTGACAACGTAAARTGCCCATGCCAGGTTCCGGCCCCCGAATTCTTCACGGAGGTGGATGGGGTGCGGCTGCACAGGTACGCTCCGCCGTGCAAACCTCTCCTACGGGATGAGGTCACATTCCAGGTCGGGCTCAACCAATACGTGGTCGGGTCACAGCTCCCATGYGAGCCCGAACCGGATGTAGCGGTGCTCACCTCCATGCTCACCGACCCCTCCCACATCACAGCAGAGRCGGCTAAGCGYAGGCTGGCCAGGGGGTCTCCCCCCTCCTTRGCCAGYTCCTCAGCTAGCCAGCTGTCTGCGCCTTCCTTGAAGGCGACATGCACTACCCGCCATGACTCCCCAGATGCTGACCTCATCGAGGCCAATCTCCTGTGGCGGCAGGAGATGGGCGGGAACATCACCCGCGTGGAGTCAGAGAACAAGGTAGTAATTCTGGACTCTTTCGAGCCGCTTCGAGCGGAGGAAGATGAGAGGGAAGTATCCGTTCCGGCGGARATCCTGCGGAGATCCAGGAAATTCCCCCGRGCGATGCCCATATGGGCAAGGCCGGATTACAACCCTCCACTGSTGGAGCCCTGGAAGGACCCGGACTACGTCCCTCCGGTGGTGCATGGGTGCCCATTGCCACCTGCTAAGGCCCCTCCAATACCACCTCCNNNNNNNNNNNNNNNNNNNNNNNNNNNNNNNNNNNNNNNNNNNNNNNNNNNNNNNNNNNNNNNNNNNNNNNNNNNNNNNNNNNNNNNNNNNNNNNNNNNNNNNNNNNNNNNNNNNNNNNNNNNNNNNNNNNNNNNNNNNNNNNNNNNNNNNNNNNNNNNNNNNNNNNNNNNNNNNNNNNNNNNNNNNNNNNNNNNNNNNNNNNNNNNNNNNNNNNNNNNNNNNNNNNNNNNNNNNNNNNNNNNNNNNNNNNNNNNNNNNNNNNNNNNNNNNNNNNNN

>HM106840

TCCGGCTCGTGGCTAAGGGACGTTTGGGACTGGATATGCACGGTGTTAAGTGATTTTAAGACCTGGCTCCAGTCCAAGCTCCTGCCGCGGTTACCGGGAGTCCCCTTCCTCTCATGCCAACGTGGGTACAAGGGCATCTGGCGGGGAGAYGGCATCATNNNNNNNNNNNNNNNNNNNNNNNNNNNNNNNNNNNNNNNNNNNNNNNNNNNNNNNNNNNNNNNCGTTGGGCCTAGGACCTGCAGCAACACGTGGCATGGRACRTTCCCCATCAACGCATACACCACGGGCCCYTGCACGCCCTCCCCGRYGCCMAATTATTCCAGGGCGCTGTGGAGGGTAGCTGCTGAGGAGTACGTGGAGGTTACGCGGGTGGGGGATTTCCACTACGTGACAGGCATGACCACTGACAACSTAAAGTGCCCATGCCAGGTTCCGGCCCCCGAATTCTTCACGGAGKTGGATGGGGTGCGGCTGCAYAGGTACGCTCCGCCGTGCAAACCTCTCCTACGGGAGGAGGTCACATTYCTGGTCGGGCTCAACCARTACCTGGTCGGGTCACAGCTYCCATGTGAGCCCGAACCGGATGTAGCGGTGCTCACYTCCATGCTCACCGACCCCTCCCACATCACAGCAGAGRCGGCTARGCGTAGGCTGGCCAGGGGGTCTCCCCCCTCCTTAGCCAGCTCATCRGCTAGCCAGTTGTCTGCGCCTTCCTTGAAGGCGACATGCRCTACCCGTCATGACTCCCCAGATGCTGACCTCATCGAGGCCAATCTCCTGTGGYGGCAGGAGATGGGCGGGAACATCACCCGCGTGGAGTCAGAGAACAAGGTAGTAATTCTGGACTCTTTCGAGCCGCTTCGAGCRGAGGCAGACGAGAGGGAAGTATCCGTTCCGGCGGAAATCCTGCGGAAATCCAGGAAATTYCCYCRAGCGATGCCCATATGGGCAAGGCCGGATTACAACCCTCCACTGATAGAGTCCTGGAAGGACCCGGACTACGTCCCTCCGRTGGTACATGGGTGCCCATTGCCACCTYCTAAGGCCCCTCCAATACCACCTCCACGGAGGAAAAGGACGGTTGTCCTGACAGAATCCACCGTGTCTTCTGCCTTGGCTGAGCTYGCCACAAAGACCTTCGGCAGCTCCGRAYCGTCGGCCGCCGACAGCGGCACGGCGACCGCCCCTCCTGACCAGCCCTCTGACGACGGCGANNNNNNNNNNNNNNNNNNNNNNNNNNNNNNNNNNNNNNNNNNNNNNNNNNNNNNNNNNNNNNNNNNNNNNNNNNNNNNNNNNNNNNNNNNNNNNNNNNNNNNNNNNNNNNNNNNNNNNNNNNNNNNN

>HM106841

TCCGGCTCGTGGCTGAGGGACGTTTGGGACTGGATATGCACGGTGTTATCTGATTTTAAGACCTGGCTCCAGTCCAAGCTCCTGCCGCGGTTACCGGGAGTCCCCTTTCTCTCATGCCAACGTGGGTATAGGGGCGTCTGGCGGGGAGACGGCATCATGCAAACCACCTGCCCATGTGGAGCGCAAATCACCGGACATGTCAAAAACGGTTCCATGAGGATCGTTGGGCCTAGGACCTGCAGCAACACGTGGCATGGAACATTCCCCATCAACGCGTACACCACGGGCCCCTGCACGCCCTCCCCGGCGCCCAATTATTCCAGGGCGCTGTGGAGGGTGGCTGCTGAGGAGTACGTGGAGGTCACGCGGGTGGGGGATTTCCACTACGTGACGGGCATGACCACTGACAACGTAAAGTGCCCATGCCAGGTTCCGGCCCCCGAATTCTTCACGGAGGTGGATGGGGTGCGGCTGCACAGGTACGCTCCGCCGTGCAAACCTCTTCTACGGGACGAGGTCACATTCCAGGTCGGGCTCAACCAATACCTGGTCGGGTCACAACTCCCATGTGAGCCCGAACCGGATGTAGCGGTGCTCACTTCCATGCTCACCGACCCCTCCCACATCACAGCAGAGGCGGCTAAGCGTAGGCTGGCCAGGGGGTCTCCCCCCTCCTTGGCCAGTTCCTCAGCTAGCCAGTTGTCTGCGCCTTCCTTGAAGGCGACATGCACTACCCGTCATGACTCCCCAGATGCCGACCTTATCGAGGCCAATCTCCTGTGGCGGCAGGAGATGGGCGGGAACATCACCCGCGTGGAGTCAGAGAACAAGGTAGTAATTCTGGACTCTTTTGAGCCGCTTCGAGCGGAGGAAGATGAAAGGGAAGTATCCGTTGCGGCGGAAATCCTGCGGAAATCCAGGAAATTTCCCCGAGCGCTGCCCATATGGGCAAGGCCGGATTACAACCCTCCACTGGTGGAGTCCTGGAAGGACCCGGACTACGTCCCTCCGGTGGTACATGGGTGCCCATTGCCACCTACTAAGACCCCTCCAATACCACCTCCACGGAGGAAGAGGACGGTTGTTTTGACAGAATCCACCGTGTCTTCTGCCTTGGCTGAGCTCGCCACAAAGACCTTCGGCAGCTCCGAATCGTCGGCCGTCGACAGCGGCACGGCGACCGGCCCTCCTGACCAGCCCTCCGATGACGGCGACNNNNNNNNNNNNNNNNNNNNNNNNNNNNNNNNNNNNNNNNNNNNNNNNNNNNNNNNNNNNNNNNNNNNNNNNNNNNNNNNNNNNNNNNNNNNNNNNNNNNNNNNNNNNNNNNNNNNNNNNNNNNNN

>HM106842

NNNNNNNNNNNNNNNNNNNNNNNNNNNNNNNNNNNNNNNNNNNNNNNNNNNNNNNNNNNNNNNNNNNNNNNNNNNNNNNNNNNNNNNNNNNNNNNNNNNNNNNNNNNNNNNNNNNNNNNNNNNNNNNNNNNNNNNNNNNNNNNNNNNNNNNNNNNNNNNNNNNNNNNNNNNNNNNNNNNNNNNNNNNNNNNNNNNNNNNNNNNNNNNNNNNNNNNNNNNNNNNNNNNNNNNNNNNNNNNNAGCAACACGTGGCATGGAACATTCCCCATCAACGCATACACCACGGGCCCCTGCACGCCCTCCCCGGCGCCCAATTATTCCAGGGCGCTGTGGAGGGTGKCTGCTGAGGAGTACGTGGAGGTTACGCGGGTGGGGGATTTCCACTACGTGACGGGCATGACCACTGACAACGTAAAGTGCCCATGCCAGGTTCCGGCCCCCGAATTCTTCACGGAGGTGGATGGGGTGCGGCTGCACAGGTACGCTCCGCCATGTAAACCTCTCCTAAGGGAGGAGGTCACATTCCAGGTCGGGCTCAACCAATACCTGGTCGGGTCACAGCTCCCATGCGAGCCCGAACCGGATGTAGCRGTGCTCACTTCCATGCTCACCGACCCCTCCCATATCACAGCAGAGGCGGCTAAGCGTAGGCTGGCCAGGGGGTCTCCCCCCTCCTTGGCCAGCTCTTCAGCTAGCCAGCTGTCTGCGCCTTCCTTGAAGGCGACATGCACTACCCATCATGACTCCCCAGACGCTGACCTCATCGAGGCCAATCTCCTGTGGCGGCAGGAGATGGGCGGGAACATCACCCGCGTGGAGTCAGAGAACAAGGTAGTAATTCTGGACTCTTTCGAACCGCTTCGAGCGGAGGAAGATGAGAGGGAAGTGTCCGTTCCGGCGGAAATCCTGCGGAGATCCAGGAAATTTCCCCGGGCGATGCCCATATGGGCAAGGCCGGATTACAACCCTCCACTGGTAGAGTCCTGGAAGGACCCGGACTACGTCCCTCCGGTGGTACATGGGTGCCCACTGCCACCTACTAAGGCCCCTCCAATACCACCTCCACGGAGGAARAGGACGGTTGTCCTGACAGAATCCACCGTGTCTTCTGCCTTAGCTGAGCTCGCTACAAAGACCTTCGGCAGCTCCGAATCGTCGGCCGTCGACAGCGGCACGGCGACCGCCCCTCCTGACCAGCCNNNNNNNNNNNNNNNNNNNNNNNNNNNNNNNNNNNNNNNNNNNNNNNNNNNNNNNNNNNNNNNNNNNNNNNNNNNNNNNNNNNNNNNNNNNNNNNNNNNNNNNNNNNNNNNNNNNNNNNNNNNNNNNNNNNNNNNNNNNNNN

>HM106843

TCCGGCTCGTGGCTAAGGGACGTTTGGGACTGGATATGCACGGTRTTAACTGATTTTAAGACCTGGCTCCAGTCCAAGCTCCTGCCGCGGTTACCGGGGATCCCCTTCCTCTCATGTCAACGTGGGTACAAGGGCGTCTGGCGGGGAGACGGCATCATGCAAACCACCTGCCCATGTGGAGCGCAGATCACCGGACATGTCAAGAACGGTTCCATGAGGATCGTTGGGCCTAGGACCTGTAGCAACACGTGGCATGGAACATTCCCCATCAACGCGTACACCACGGGCCCCTGCACGCCCTCCCCGGCGCCCAATTACTCCAGGGCGTTGTGGAGGGTGGCTGCTGAGGAGTACGTGGAGGTTACGCAGGTGGGAGATTTCCACTACGTGACGGGCATGACCACTGACAACGTAAAGTGCCCATGCCAGGTTCCGGCCCCCGAATTCTTCACGGAGGTGGATGGGGTGCGGCTGCACAGGTACGCTCCGCCGTGCAAACCTCTCCTACGGGAGGAGGTCACATTCCAGGTCGGGCTCAACCAATACCTGGTCGGGTCACAGCTCCCATGCGAGCCCGAACCGGATGTAGCAGTGCTCACTTCCATGCTCACCGACCCCTCCCATATCACAGCAGAAGCGGCTAAGCGTAGGCTGGCCAGGGGGTCTCCCCCCTCCTTAGCCAGCTCTTCAGCTAGCCAGTTGTCTGCGCCTTCCTTGAAGGCGACATGCACTACCCGTCATGACTCCCCAGATGCAGACCTCATTGAGGCTAATCTCCTGTGGCGGCAGGAGATGGGCGGGAACATCACCCGCGTGGAGTCAGAGAATAAGGTAGTAATACTGGACTCTTTCGAGCCGCTTCGAGCGGAGGAAGATGAGAGGGAAGTATCCGTTCCGGCGGAGATCCTGCGAAGATCCAGGAAATTTCCCCGGGCGATGCCCATATGGGCAAGGCCGGATTACAACCCTCCACTGGTAGAGTCCTGGAAGGACCCGGACTATGTCCCTCCGGTGGTACATGGGTGCCCATTACCACCTACTAAGGCCCCTCCAATACCACCTCCACGGAGGAAAAGGACGGTTGTCCTGACAGAATCCACCGTGTCTTCTGCCTTGGCCGAGCTYGCCGCAAAGACCTTCGGCAGCTCCGAATCGTCGGCCGTCGACAGCGGCACGGCGACCNNNNNNNNNNNNNNNNNNNNNNNNNNNNNNNNNNNNNNNNNNNNNNNNNNNNNNNNNNNNNNNNNNNNNNNNNNNNNNNNNNNNNNNNNNNNNNNNNNNNNNNNNNNNNNNNNNNNNNNNNNNNNNNNNNNNNNNNNNNNNNNNNNNNNNNNNNNNNNN

>HM106844

TCCGGCTCGTGGTTAAGGGACGTTTGGGACTGGATATGCACGGTGTTAACTGATTTTAAGACCTGGCTCCAGTCCAAGCTCCTGCCGCGGTTACCGGGAGTCCCCTTCCTCTCATGCCAACGTGGGTACAAGGGCATCTGGCGGGGAGACGGCATCATGCAAACCACCTGCCCATGTGGAGCGCAAATCACCGGACATGTCAAAAATGGTTCCATGAGGATCGTTGGGCCTAGAACCTGCAGCAACACGTGGCATGGRACATTCCCCATCAACGCGTACACCACGGGCCCYTGCACGCCCTCCCCGGCGCCCAAYTATTCTAGGGCGCTGTGGMGGGTGKCTGCTGAGGAGTACGTGGAGGTTACGCGGGTGGGGGATTTCCAYTACGTGACGGGCATGACCACTGACAACGTAAAGTGCCCATGCCAGGTTCCGGCCCCCGAATTCTTCACGGAGGTGGATGGGGTGCGGCTGCACAGGTACGCTCCGCCGTGCAGACCTCTCCTACGGGAGGAGGTCACATTCCAGGTCGGGCTCAACCAATACCTGGTCGGGTCACAGCTCCCATGTGAGCCCGARCCGGATGTAGCGGTGCTCACTTCCATGCTCACCGACCCCTCCCACATCACAGCAGAGRCGGCTAAGCGTAGGCTGGCCAGGGGGTCTCCCCCCTCCTTGGCCAGCTCTTCAGCTAGCCAGTTGTCTGCGCCTTCYTTGAAGGCGACATGCACTACCCGTCATGACTCCCCAGATGCTGACCTCATCGAGGCCAATCTCCTGTGGCGGCAGGAGATGGGCGGRAACATCACCCGCGTGGAGTCAGARAACAAGGTAGTAATTCTGGACTCTTTCGAGCCGCTTCGAGCGGAGGAAGATGAGAGGGAAGTRTCCGTTCCGGCGGAAATCCTGCGGARATCCAGGAAATTTCCCCGRGCGATGCCCATATGGGCAAGGCCGGATTACAACCCTCCACTGGTRGAGTCCTGGAAGGACCCGGACTACGTCCCTCCGGTGGTRCATGGGTGCCCATTRCCACCTACTAAGRCYCCTCCRATACCACCTCCACGGAGGAAAAGGACGGTTGTCCTGACAGAATCCACCGTGTCTTCTGCCTTGGCTGAGCTCGCCACAAAGACCTTCGGCAGCTCCGARTCGTCGGCCGTCGACAGCGGCACGGCGACCGCCCCTCCTGACCAGCCCTCCGACGACGGCGACGCAGGGTCCGATGTTGAGTCGTACTCCTCCATGCCCCCCCTTGAGGGGGAGCCGGGGGATCCCGATCTCAGCGACGGGTCTTGGTCTACCGTGAGCGAGGAAGCTAGTGAGGACGTCGTCTGCTGC

>HM106845

NNNNNNNNNNNNNNNNNNNNNNNNNNNNNNNNNNNNNNNNNNNNNNNNNNNNNNNNNNNNNNNNNNNNNNNNNNNNNNNNNNNNNNNNNNNNNNNNNNNNNNNNNNNNNNNNNNNNNNNNNNNNNNNNNNNNNNNNNNNNNNNNNNNNNNNNNNNNNNNNNNNNNNNNNNNNNNNNNNNNNNNNNNNNNNNNNNNNNNNNNNNNNNNNNNNNNNNNNNNNNNNNNNNNNNNNNNNNNNNNNNNNNNNNNNNNNNNNNNNNATTCCCCATCAACGCATACACCACGGGCCCCTGCACRCCCTCCCCGGCGCCCAATTATTCCAGGGCGCTGTGGAGGGTGGCTGCCGAGGAGTACGTGGAGGTTACGCGGGTGGGGGATTTCCACTACGTGACGGGCATGACCACTGACAACGTAAAGTGCCCATGTCAGGTCCCGGCCCCCGAATTCTTCACGGAGGTGGATGGGGTGCGGCTGCACAGGTACGCTCCGCCATGCAGACCTCTCCTACGGGAGGAGGTCACATTCCAGGTCGGGCTCAACCAATACCTGGTTGGGTCACAGCTCCCATGCGAGCCCGAGCCGGATGTAGCGGTGCTCACTTCCATGCTCACCGATCCCTCCCACATCACAGCAGAGGCGGCTAAGCGTAGGCTGGCCAGGGGGTCTCCCCCCTCCTTGGCCAGCTCTTCAGCTAGCCAGTTGTCTGCGCCTTCCTTGAAGGCGACATGCACTACCCATCATGACTCCCCAGATGCTGACCTCATCGAGGCCAATCTCCTGTGGCGGCAGGAGATGGGCGGGAACATCACCCGCGTGGAGTCAGAGAACAAGGTAGTAATTCTGGACTCTTTCGAGCCGCTTCGAGCGGAGGAAGATGAGAGGGAAGTATCCGTTCCGGCGGAAATCCTGCGGAAATCCAGGAAGTTTCCCCGAGCGATGCCCATATGGGCAAGGCCGGATTACAACCCTCCACTAGTAGAGTCCTGGAAGRACCCGGACTACGTCCCTCCGGTGGTACATGGGTGCCCATTGCCACCYACTAAGGCCCCTCCAATACCACCYCCACGGAGGAAAAGGACGGTTGTCCTGACAGAATCCACCGTGTCTTCTGCATTGGCTGAGCTCGCCACAAAGACCTTCGGCAGCTCCGAATCGTCGGCCGTCGACAGCGGCACGGCGACCGCCCCTCCYGACCAGCCCTCCGACGACGGCGACACAGGGTCCGATGTTGAGTCGTACTCCTCCATGCCCCCCCTTGAGGGGGAGCCGGGGGATCCCGATCTCAGCGACGGGTCTTGGTCTACCGTGAGCGAGGAAGTTAGTGAGGACGTCGTCTGCTGC

>HM106846

TCYGGCTCGTGGCTAAGGGACGTTTGGGACTGGATATGCACGGTGTTAACTGATTTTAAGACCTGGCTCCAGTCCAAGCTCCTGCCGCGGTTACCGGGAGTCCCCTTCCTCTCATGCCAACGTGGNNNNNNNNNNNNNNNNNNNNNNNNNNNNNNNNNNNNNNNNNNNNNNNNNNNNNNNNNNNNNNNNNNNNNNNNNNNNNNNNNNNNNNNNNNNNNNNNNNNNNNNNNNNNNNNNNNNNNNNNNNNNNNNNNNNNNNNATTCCCCATCAACGCATACACCACGGGCCCCTGCACGCCCTCCCCGGCGCCCAATTATTCCAGGGCGCTGTGGAGGGTGGCTGCTGAGGAGTACGTGGAGGTTACGCGGGTGGGGGATTTCCACTACGTGACGGGCATGACCACTGACAACGTAAAGTGCCCATGCCAGGTTCCGGCCCCCGAATTCTTCACGGAGGTGGATGGGGTGCGGCTGCACAGGTACGCTCCACCATGCAAACCTCTCCTACGGGAGGAGGTCACATTCCAGGTCGGGCTCAACCAATACCTGGTCGGGTCGCAGCTCCCGTGCGAGCCCGAACCGGATGTAGCGGTGCTCACTTCCATGCTCACCGACCCCTCCCACATCACAGCAGAGACGGCCAAGCGCAGGCTGGCTAGGGGGTCTCCCCCCTCCTTAGCCAGCTCTTCAGCTAGCCAGTTGTCTGCGCCTTCCTTGAAGGCGACCTGCACTACCCGTCATGACTCCCCAGATGCTGACCTCATCGAGGCCAATCTCCTGTGGCGGCAGGAGATGGGCGGGAACATCACCCGCGTGGAGTCAGAGAACAAGGTGGTAATTCTGGACTCTTTCGAGCCGCTTCGAGCGGAGGAAGATGAGAGGGAAGTATCCGTTCCGGCGGAAATCCTGCGGAGATCCAGGAAATTTCCCCGGGCGATGCCCGTGTGGGCAAGGCCGGATTACAACCCTCCACTGGTAGAGTCCTGGAAGGACCCGGACTACATCCCTCCGGTGGTGCATGGGTGCCCATTGCCACCTRCTAAGGCCCCTCCAATACCACCTCCACGGAGGAAAAGGACGGTTGTCCTGACAGAATCCACCGTGTCTTCCGCCCTGGCTGAGCTCGCCACAAAGACCTTCGGCAGCTCCGAATCGTCGGCCGTCGACAGCGGCACGGCGACCGSCCCTCCTGACCANNNNNNNNNNNNNNNNNNNNNNNNNNNNNNNNNNNNNNNNNNNNNNNNNNNNNNNNNNNNNNNNNNNNNNNNNNNNNNNNNNNNNNNNNNNNNNNNNNNNNNNNNNNNNNNNNNNNNNNNNNNNNNNNNNNNNNNNNNNNNNNNN

>HM106847

TCCGGYTCGTGGCTSAGGGAYGTTTGGGACTGGATATGCACGGTGTTARCTGATTTTAAGACCTGGCTCCAGTCCAAGCTCCTGCCGCGGTTACCGGGAGTCCCCTTCCTCTCRTGCCAACGTGGGTACAAGGGCATYTGGCGGGGAGACGGCATCATGCAAACCACCTGCCCATGTGGAGCACARATCACCGGACATGTCAAAAACGGTTCCATGAGGATCGTTGGGCCTARGACCTGCAGCAACACGTGGCATGGAACATTCCCCATCAACGYRTACACCACGGGCCCYTGCACKCCCTCCCCGGCGCCMAATTATTCCAGGGCGCTGTGGAGGGTGGCYGCTGAGGAGTACGTGGAGGTTACGCGGGTGGGGGATTTCCACTACGTGACGGGCATGACCACTGACAACGTAAAGTGCCCATGCCAGGTTCCGGCCCCCGARTTYTTCACRGAGGTGGAYGGGGTRCGGCTGCACAGGTACGCYCCGCCGTGCAAACCTCTCCTACGGGAGGAGGTCACATTCCAGGTCGGGCTCAACCAATAYCTGGTYGGATCACAGCTCCCATGYGAGCCCGARCCGGATGTRGCGGTGCTCACTTCCATGCTCACCGACCCCTCCCACATYACAGCAGAGRCGGCTAAGCGTAGGCTGGCCAGGGGGTCTCCCCCCTCCTTRGCCAGCTCYTCAGCTAGCCAGTTGTCTGCGCCTTCTTTGAAGGCRACATGCACTACCCGTCATGACTCCCCAGAYGCTGACCTCATCGAGGCCAATCTTCTGTGGCGGCAGGAGATGGGCGGGAACATCACCCGCGTGGAGTCAGAGAACAAGGTAGTAATTCTGGACTCTTTCGAGCCGCTYCGAGCGGAGGAWGATGAGAGGGAAGTRTCCGTTCCKGCGGAAATCCTGCTGARATCCAGGAARTTTCCCCSAGCGATGCCCRTATGGGCAAGGCCGGATTACAACCCTCCACTGGTAGAGTCCTGGAAGGACCCGGACTACGTCCCTCCGGTGGTACATGGGTGCCCATTRCCACCTACTAAGGCCCCTCCAATACCACCTCCACGGAGGAAAAGGACGGTTGTCCTGACAGAAAGCWCCGTGTCYTCTGCCTTGGCTGAGCTCGCCGCAAAGACCTTCRGCAGCTCCGAATCGTCGGCCGTCGACAGTGGCACGGCRACCGCCCCTCCTGACCAGNNNNNNNNNNNNNNNNNNNNNNNNNNNNNNNNNNNNNNNNNNNNNNNNNNNNNNNNNNNNNNNNNNNNNNNNNNNNNNNNNNNNNNNNNNNNNNNNNNNNNNNNNNNNNNNNNNNNNNNNNNNNNNNNNNNNNNNNNNNNNNNN

>HM106848

NNNNNNNNNNNNNNNNNNNNNNNNNNNNNNNNNNNNNNNNNNNNNNNNNNNNNNNNNNNNNNNNNNNNNNNNNNNNNNNNNNNNNNNNNNNNNNNNNNNNNNNNNNNNNNNNNNNNNNNNNNNNNNNNNNNNNNNNNNNNNNNNNNNNNNNNNNNNNNNNNNNNNNNNNNNNNNNNNNNNNNNNNNNNNNNNNNNNNNNNNNNNNNNNNNNNNNNNNNNNNNNNNNNNNNNNNNNNNNNNNNNNNNNNNNNNNNNNNNNNATTCCCCATCAACGCATACACCACGGGCCCCTGCACGCCCTCCCCGGCGCCCAATTATTCCAGGGCGCTGTGGAGGGTGACTGCTGAGGAGTACGTGGAGGTTACGCGGGTGGGGGATTTCCACTACGTGACGGGCATGACCACTGACAACGTAAAGTGCCCATGCCAGGTTCCGGCCCCCGAATTCTTCACGGAGGTGGATGGGGTGCGGCTGCACAGGTACGCTCCGCCGTGCAAACCTCTCCTACGGGATGAGGTTACATTCCAAGTYGGGCTCAACCAATACSTGGTCGGGTCACARCTCCCATGCGAGCCCGAACCGGATGTAGCGGTGCTCACYTCCATGCTCACCGACCCCTCCCACATCACAGCAGAGRCGGCTAAGCGTAGGCTGGCTAGGGGGTCYCCCCCCTCCTTAGCYAGCTCTTCAGCTAGCCAGTTGTCTGCGCCTTCYTTGAAGGCGACWTGCACTACCCRTCATGACTCCCCAGATGCTGACCTCATCGAGGCCAATCTCCTGTGGCGGCAGGAGATGGGCGGGAACATCACCCGCGTGGAGTCAGAGAACAAGGTAGTAATYCTGGACTCTTTYGAGCCGCTYCGAGCGGAGGAAGATGAGAGGGAAGTATCCGTTCCTGCGGAAATCCTGCGGAGATCCAGGAAATTYCCCCGRGCRATGCCAATATGGGCAAGGCCGGATTACAACCCTCCACTGGTGGAGTCCTGGAAGGACCCGGACTACGTCCCTCCGGTGGTACATGGGTGCCCGTTGCCACCTACTAAGGCCCCTCCAATACCACCTCCACGGAGGAAAAGGACGGTTGTCCTGACAGAATCCACCGTDTCYTCTGCCTTGGCTGARCTYGCCACAAAGACCTTCGGCAGCTCCGAATCGTCGGCCGTCGACAGCGGCACGGCGACCGCTCCTCCTGACCAGCCCTCCGACGACGGCGACGCAGGGTCCGAYGTTGRGTCGTAYTCCTCCATGCCCCCCCTTGAGGGGGAGCCGGGGGATCCCGATCTCAGCGACGGGTCTTGGTCTACCGTGAGCGAGGAAGCTAGTGAGGACGTCGTCTGCTGC

>HM106849

NNNNNNNNNNNNNNNNNNNNNNNNNNNNNNNNNNNNNNNNNNNNNNNNNNNNNNNNNNNNNNNNNNNNNNNNNNNNNNNNNNNNNNNNNNNNNNNNNNNNNNNNNNNNNNNNNNNNNNNNNNNNNNNNNNNNNNNNNNNNNNNNNNNNNNNNNNNNNNNNNNNNNNNNNNNNNNNNNNNNNNNNNNNNNNNNNNNNNNNNNNNNNNNNNNNNNNNNNNNNNNNNNNNNNNNNNNNNNTGCAGCAACACGTGGCATGGAACATTCCCCATCAACGCRTACACCACGGGCCCCTGCACGCCTTCCCCGGCGCCCAATTATTCCAGGGCGCTGTGGAGGGTGGCTGCTGAGGAGTACGTGGAGGTTACGCGGGTGGGGGATTTCCACTACGTGACGGGCATGACCACTGACAACGTAAAGTGCCCATGCCAGGTTCCGGCCCCCGAATTCTTCACGGAGGTGGATGGGGTGCGGCTGCACAGGTACGCTCCGCCGWGCAAACCYCTCCTACGGGAGGAGGTCACATTCCAGGTCGGGCTCAACCARTAYCTGGTCGGGTCACAGCTCCCATGYGARCCYGAACCGGATGTAGCGGTGCTCACTTCCATGCTCACCGACCCCTCCCACATCACRGCAGAGGCGGCTAAGCGTAGGCTGGCYAGAGGGTCTCCCCCCTCYTTAGCCAGCTCTTCAGCTAGCCAGTTGTCTGCGCCTTCCTTGAAGGCGACATGTACTACYCRTCATGACTCCCCAGATGCTGACCTCATCGAGGCYAATCTCCTGTGGCGRCAGGAGATGGGCGGGAACATCACCCGCGTGGAGTCAGAGAACAAGGTAGTAATTCTGGACTCTTTCGAGCCGCTCCGAGCGGAGGAAGATGAGAGGGAAGTATCCGTTCCGGCGGAAATCCTGCGGASATCCAGGAAATTTCCCCGAGCGATGCCCATATGGGCRAGGCCAGATTACAACCCTCCACTGATAGAGTCCTGGAAGGACCCGGACTACGTCCCTCCGGTGGTACATGGGTGCCCATTGCCACCTACTAAGGCCCCTCCAATACCACCTCCACGGAGGAAAAGGACGGTTGTCCTGACAGAATCCACCGTGTCTTCTGCCTTGGCTGAGCTCGCCACAAAGACCTTCGGCAGCTCTGAATCGTCGGCCGYCGACAGCGGCACGGCGACCGCCCCTCCTGACCAGCCCTCCGACGNNNNNNNNNNNNNNNNNNNNNNNNNNNNNNNNNNNNNNNNNNNNNNNNNNNNNNNNNNNNNNNNNNNNNNNNNNNNNNNNNNNNNNNNNNNNNNNNNNNNNNNNNNNNNNNNNNNNNNNNNNNNNNNNNNNNNN

>HM106850

TCCGGCTCGTGGCTAAGGGATGTTTGGGACTGGATATGCACGGTGTTAAGTGATTTTAAGACCTGGCTCCAGTCCAAGCTCCTGCCGCGGTTACCGGGAGTCCCCTTCCTCTCATGCCAACGTGGGTACAAGGGCATCTGGCGGGGAGACGGCATCATGCAAACCACCTGCCCATGTGGANNNNNNNNNNNNNNNNNNNNNNNNNNNNNNNNNNNNNNNNNNNNNNNNNNNNNNNCCTGCAGCAACACGTGGCATGGAACATTCCCCATCAACGCACACACCACGGGCCCCTGCACGCCCTCCCCTGCGCCCAATTATTCCAGGGCGCTGTGGAGGGTGGCTGCTGAGGAGTACGTGGAGGTTACGCGGGTGGGGGATTTCCACTACGTGACGGGCATGACCACTGACAACGTAAAGTGCCCATGCCAGGTTCCGGCCCCCGAATTCTTCACGGAGGTGGATGGGGTGCGGCTGCACAGGTACGCTCCGCCGTGCAGACCTCTCCTACGGGAGGAGGTCACATTCCAGGTCGGGCTCAACCAATACCTGGTCGGGTCACAGCTCCCATGTGAGCCCGAACCGGATGTAGCGGTGCTCACCTCCATGCTCACCGACCCCTCCCACATCACAGCAGAGGCGGCTAAGCGTAGGCTGGCCAGGGGGTCTCCCCCCTCCTTGGCCAGCTCTTCAGCTAGCCAGTTGTCTGCGCCTTCCTTGAAGGCGACATGCACTACCCGTCATGACTCCCCAGATGCTGACCTCATCGAGGCCAATCTCCTGTGGCGACAGGAGATGGGCGGGAACATCACCCGCGTGGAGTCAGAGAACAAGGTAGTAATTCTGGACTCTTTCGAGCCGCTTCGAGCGGAGGAAGATGAGAGGGAAGTATCCGTTCCGGCGGAGATCCTGCGGAAATCCAGGAAATTTCCCCGAGCGATGCCCATATGGGCAAGGCCGGATTACAACCCTCCACTGGTGGAGCCCTGGAAGGACCCGGACTACGTCCCTCCGGTGGTGCATGGGTGCCCATTGCCACCTACTAAGGCCCCTCCAATACCACCTCCACGGAGGAAAAGGACGGTTGTCCTGACAGAATCCACCGTGTCTTCTGCCTTGGCTGAGCTCGCCACAAAGACCTTCGGCAGCTCCGAATCGTCGGCCGYCGACAGCGGCACGGCGACCGCCCCTCCTGACCAGCCCTCCGANNNNNNNNNNNNNNNNNNNNNNNNNNNNNNNNNNNNNNNNNNNNNNNNNNNNNNNNNNNNNNNNNNNNNNNNNNNNNNNNNNNNNNNNNNNNNNNNNNNNNNNNNNNNNNNNNNNNNNNNNNNNNNNNNNNNNNNN

>HM106851

TCCGGCTCGTGGCTRAGGGACGTYTGGGACTGGATATGCACGGTGTTAACYGATTTTAAGACCTGGCTCCAGTCCAAGCTCCTGCCGCGGTTACCGGGAGTCCCYTTCCTCTCATGCCAACGTGGGTACAAGGGCGTYTGGCGGGGAGACGGCATCATGMMAACCACYTGCCCATGTGGAGCGCAGATCACCGGACATGTCAAAAACGGTTCNNNNNNNNNNNNNNNNNNNNNNNNNNNNAGCAACACGTGGCATGGAACATTYCCCATCAACGCGTACACCACGGGCCCTTGCACGCCCTCCCCGGCGCCCAATTATTCCAGGGCGCTATGGAGGGTGGCTGCTGAGGAGTACGTGGAGGTYACGCRGGTGGGGGATTTCCACTACGTGACGGGCATGACCACYGACAACGTAAAGTGCCCATGYCAGGTTCCGGCCCCCGAATTCTTCACGGAGGTGGATGGGGTGCGGCTGCACAGGTACGCTCCGCCGTGCAAGCCTCTCCTRCGGGAGGAGGTCACATTCCAGGTCGGGCTCAAYCAATACCTGGTCGGGTCACAGCTCCCATGTGAGCCCGAACCGGATGTRGCGGTGYTCACTTCCATGCTCACCGACCCCTCCCACATCACAGCAGAGACGGCTAAGCGTAGGCTGGCCAGGGGGTCTCCCCCCTCCTTAGCCAGCTCTTCAGCTAGCCAGTTGTCTGCGCCTTCCTTGAAGGCGACATGCACTACCCRTCATGACTCCCCAGATGCTGACCTCATCGAGGCCAATCTCCTGTGGCGGCAGGAGATGGGCGGGAACATCACCCGCGTGGAGTCAGAGAACAAGGTAGTAATTYTGGACTCYTTCGAGCCGCTTCGAGCGGAGGAAGATGAGAGGGAARTATCTGTTCCGGCGGAAATCCTGCGGAGATCCAGGAAATTTCCCCGAGCGATGCCCATATGGGCAAGGCCGGATTACAACCCTCCAYTGATAGAGTCCTGGAARRACCCGGACTACGTCCCTCCGGTGGTRCATGGGTGCCCATTGCCACCTACYAAGGCCCCTCCAATACCACCACCACGGAGGAAAAGGACGGTTGTCCTRACAGAATCCACCGTGTCTTCTGCYTTGGCTGARCTCGCCGCAAAGACCTTCGGCAGCTCCGAGTCGTCGGCCGNNNNNNNNNNNNNNNNNNNNNNNNNNNNNNNNNNNNNNNNNNNNNNNNNNNNNNNNNNNNNNNNNNNNNNNNNNNNNNNNNNNNNNNNNNNNNNNNNNNNNNNNNNNNNNNNNNNNNNNNNNNNNNNNNNNNNNNNNNNNNNNNNNNNNNNNNNNNNNNNNNNNNNNNNNNNNNNNNNN

>HM106852

TCCGGCTCGTGGCTAAGRGACGTTTGGGACTGGATATGCACGGTGTTAACTGATTTTAAGACCTGGCTCCAGTCCAAGCTCCTGCCGCGGTTACCGGGAGTCCCCTTCCTCTCATGCCAACGTGGGTACAAGGGTATCTGGCGGGGAGACGGCATCATGCAAACCACCTGCCCATGTGGAGCACAGATCACCGGACATGTCAAAAACGGTTCCATGAGGATCGTTGGGCCTAGGACCTGCAGCAACACGTGGCAYGGAACATTCCCCATCAACGCATACACCACGGGCCCCTGCACGCCCTCCCCGGCGCCCAATTATTCCAGRGCGCTGTGGAGGGTGGCTGCTGAGGAGTACGTGGAGGTTACGCGGGTGGGGGATTTCCACTACGTGACGGGCATGACCACCGACAACGTAAAGTGCCCATGCCAGGTTCCGGCCCCCGAATTCTTCACGGAGGTAGATGGGGTGCGGCTGCACAGGTACGCTCCGCCRTGCAAACCTCTCCTACGGGAGGAGGTCGCATTCCAGGTCGGGCTCAACCAATACCTGGTCGGGTCACAGCTCCCATGYGAGCCCGAACCGGATGTAGCGGTGCTCACTTCCATGCTCACCGAYCCCTCCCACATCACAGCAGAGGCGGCTAAGCGYAGGCTGGCCAGGGGGTCTCCTCCCTCCTTRGCCAGCTCTTCMGCTAGCCAGTTGTCTGCGCCTTCCTTGAAGGCGACATGCACTACCCGTCATGACTCCCCAGATGCTGACCTCATCGAGGCCAATCTCCTGTGGCGGCAGGAGATGGGCGGGAACATCACCCGCGTGGAGTCAGAGAACAAGGTAGTAATTCTGGACTCTTTCGAGCCGCTTCGAGCGGAGGAAGATGAGAGGGAAGTATCCGTTGCKGCGGAAATYCTGCGGAAATCCCGGARATTYCCCCGAGCGATGCCCATATGGGCAAGGCCGGATTACAACCCTCCACTGGTAGAGTCCTGGAAGGACCCGGACTACGTCCCTCCRGTGGTACATGGGTGCCCATTGCCACCTACTAAGGCCCCTCCAATACCACCYCCACGGAGGAAAAGGACGGTTGTCCTGACAGAATCCATTGTGTCTTCYGCCTTGGCTGAGCTCGCCACRAAGACCTTCGGCAGCTCSGAATCGTCGGCCGTCGACAGCGGCACGGCGACCGCCCCTCCTGACCAGCCCTCCGACGACGGNNNNNNNNNNNNNNNNNNNNNNNNNNNNNNNNNNNNNNNNNNNNNNNNNNNNNNNNNNNNNNNNNNNNNNNNNNNNNNNNNNNNNNNNNNNNNNNNNNNNNNNNNNNNNNNNNNNNNNNNNNNNNNNNNN

>HM106853

TCCGGCTCGTGGCTAAGGGACGTTTGGGACTGGATATGTACGGTGCTAACCGATTTTAAGACCTGGCTCCAGTCCAAGCTCCTGCCGCGGTTGCCGGGAGTCCCCTTCCTCTCATGCCAACGTGGGTACAAGGGCGTCTGGCGGGGAGACGGCATCATGCAAACCACCTGCCCATGTGGAGCGCAGATCACCGGACATGTCAAAAACGGGTCCATGAGGATCGTTGGGCCTAGGACCTGCAGCAACACGTGGCAYGGAACATTCCCCATCAACGCRYACACCACGGGGCCCTGCACGCCCTCCCCGGCGCCCAATTATTCCAGGGCGCTGTGGAGGGTGGCTGCTGAGGACTACGTGGAGGTTACGCGGGTGGGGGATTTCCACTACGTGACGGGCATGACCACTGACAACGTAAAGTGCCCATGCCAGGTTCCGGCCCCCGAATTCTTCACGGAGGTGGATGGGGTGCGGCTGCACAGGTACGCTCCGCCGTGCAAACCTCTCCTGCGGGAGGAGGTCACATTCCTGGTCGGGCTCAACCAGTACCTGGTCGGGTCACAGCTCCCATGTGAGCCCGAACCGGATGTAGCGGTGCTTACTTCCATGCTCACCGACCCCTCCCACATCACAGCAGAGRCGGCTAAGCGTAGGCTGGCCAGGGGGTCTCCCCCCTCCTTRGCCAGCTCTTCAGCTAGCCAGYTGTCTGCGCCTTCCTTGAAGGCGACATGCACTACCCGTCAYGACTCCCCAGATGCTGACCTCATCGAGGCCAACCTCCTGTGGCGRCAGGAGATGGGCGGGAACATCACCCGCGTGGAGTCAGAGAACAAGGTAGTAATTCTGGACTCTTTCGAGCCGCTTCGAGCGGAGGAAGATGAGAGGGAAGTATCCGTTCCGGCGGAAATCCTGCGGAGATCCAGGAAATTTCCCCGAGCGATGCCCATATGGGCAAGGCCGGATTACAACCCYCCACTGATAGAGTCCTGGAAGGACCCGGACTAYGTTCCTCCGGTGGTACATGGGTGCCCATTGCCACCTACTAAGGCCCCTCCAATACCACCTCCACGGAGGAAAAGGACGGTYGTCCTGACAGAATCCACCGTGTCTTCTGCCYTGGCTGAGCTCGCCACAAAGACCTTCGGCAGCTCCGAATCGTCGGCCGYCGACAGCGGCACGGCGACCGCCCCTCCTGACCAGCYCTCCGACGACGGCGACRCAGGGTCCGATGTTGAGTCGTACTCCTCCATGCCCCCCCTTGAGGGGGAGCCGGGGGATCCCGATCTCAGCGACGGGTCTTGGTCTACCGTGAGCGAGGAAGCTGGTGAGGAYGTCGTCTGCTGC

>HM106854

NNNNNNNNNNNNNNNNNNNNNNNNNNNNNNNNNNNNNNNNNNNNNNNNNNNNNNNNNNNNNNNNNNNNNNNNNNNNNNNNNNNNNNNNNNNNNNNNNNNNNNNNNNNNNNNNNNNNNNNNNNNNNNNNNNNNNNNNNNNNNNNNNNNNNNNNNNNNNNNNNNNNNNNNNNNNNNNNNNNNNNNNNNNNNNNNNNNNNNNNNNNNNNNNNNNNNNNNNNNNNNNNNGGGCCTAGGACCTGCAGCAACACGTGGCATGGAACATTCCCCATCAACGCATACACCACGGGCCCCTGCACGCCCTCCCCGGCGCCCAACTATTCCAGGGCGCTGTGGAGGGTGGCTGCTGAGGAGTACGTGGAGGTTACGCGGGTGGGGGATTTCCACTACGTGACGGGCATGACCACTGACAACGTAAAGTGCCCATGCCAGGTTCCGGCCCCCGARTTCTTCACGGAGGTGGATGGGGTACAGCTGCACAGGTACGCTCCGCCSTGCAAACCCCTCCTACGGGAGGAGGTCACGTTCCARGTCGGGCTCAACCAATAYCTGGTCGGGTCACAGCTCCCATGTGAGCCCGARCCGGATGTAGCRGTGYTCACTTCCATGCTCACCGACCCCTCCCACATCACAGCAGAGGCGGCTAAGCGTAGGCTGGCCAGGGGGTCTCCCCCCTCCTTAGCCAGCTCTTCAGCTAGCCAGTTGTCTGCGCCTTCYTTGAAGGCGACATGCACTACCCATCATGACTCYCCAGATGCTGACCTCATCGAGGCCAATCTCCTGTGGCGGCAGGAGATGGGCGGGAACATCACCCGCGTGGAGTCAGAGAACAAGGTAGTAATTCTGGACTCTTTCGAGCCGCTTCGAGCGGAGGAAGATGAGGGGGAAGTATCCGTTCCGGCAGAAATCCTGCGGAGATCCAGGAGATTTCCCCGAGCGATGCCCATATGGGCAAGGCCGGATTACAACCCYCCACTGRTAGAGTCCTGGAAGGACCCGGACTACGTCCCTCCGGTGGTACACGGGTGCCCATTGCCACCYACTAAGGCCCCTCCAATACCACCCCCACGGAGGAAGAGGACGGTTGTCCTGACAGAATCCACTGTGTCTTCTGCCTTGGCTGAGCTCGCCACAAAGACCTTCGGCAGCTCCGAATCGTCGGCCGYCGACAGCGGTACGGCGACCGCCCCTCCTGACCAGGCNNNNNNNNNNNNNNNNNNNNNNNNNNNNNNNNNNNNNNNNNNNNNNNNNNNNNNNNNNNNNNNNNNNNNNNNNNNNNNNNNNNNNNNNNNNNNNNNNNNNNNNNNNNNNNNNNNNNNNNNNNNNNNNNNNNNNNNNNNNNNN

>HM106855

NNNNNNNNNNNNNNNNNNNNNNNNNNNNNNNNNNNNNNNNNNNNNNNNNNNNNNNNNNNNNNNNNNNNNNNNNNNNNNNNNNNNNNNNNNNNNNNNNNNNNNNNNNNNNNNNNNNNNNNNNNNNNNNNNNNNNNNNNNNNNNNNNNNNNNNNNNNNNNNNNNNNNNNNNNNNNNNNNNNNNNNNNNNNNNNNNNNNNNNNNNNNNNNNNTTCCATGAGGATCGTTGGGCCTAGGACCTGCAGCAACACGTGGCATGRAACATTTCCCATCAACGCGTACACCACGGGCCCCTGCACGCCCTCCCCRGCGCCCAATTATTCCAGGGCGCTGTGGAGGGTGGCTGCTGAGGAGTACGTGGAGGTYACGCGGGTGGGGGATTTCCACTACGTGACGGGCATGACCACTGACAACGTGAAGTGCCCRTGCCAGGTTCCGGCCCCTGAATTYTTCACAGAGGTGGATGGGGTGCGGCTGCACAGGTACGCTCCGCCGTGCAAACCTCTCCTACGGGAGGAGGTCACATTCCAGGTCGGGCTCAACCAGTACCTGGTCGGGTCACAGCTCCCATGYGAGCCTGAACCGGATGTRGCGGTGCTCACYTCYATGCTCACCGACCCCTCCCACATCACAGCAGAGRCGGCTAAGCGTAGGCTGGCCAGGGGGTCTCCCCCCTCCTTGGCCAGYTCTTCAGCTAGCCAGCTGTCTGCGCCTTCYTTGAAGGCGACATGCACTACCCGTCACGACTCCCCAGATGCTGACCTCATCGAGGCCAATCTCCTGTGGCGGCAGGAGATGGGCGGGAACATCACCCGCGTGGAGTCAGAGAACAAGGTAGTAATTCTGGACTCTTTCGAGCCGCTTCGAGCGGAGGAAGATGAGAGGGAAGTATCCGTTCCGGCGGARATCCTGCGGARATCCAGGAAATTYCCCCGRGCGATGCCCGTATGGGCAAGGCCRGATTACAACCCTCCACTGGTRGAGTCCTGGAARGACCCGGACTACGTYCCTCCSGTGGTACATGGGTGCCCATTGCCACCTACTAAGGCCCCTCCAATACCACCTCCACGGAGGAAAAGGACGGTTGTCCTGACAGAATCCACCGTGTCTTCTGCCYTGGCTGAGCTCGCCGCAAAGACCTTCGGCAGCTCYGAATCGTCGGCCGTCGACAGCGGCACGGCGACCGCCCCTCCTGACCAGACCNNNNNNNNNNNNNNNNNNNNNNNNNNNNNNNNNNNNNNNNNNNNNNNNNNNNNNNNNNNNNNNNNNNNNNNNNNNNNNNNNNNNNNNNNNNNNNNNNNNNNNNNNNNNNNNNNNNNNNNNNNNNNNNNNNNNNNNNNNNNN

>HM106856

NNNNNNNNNNNNNNNNNNNNNNNNNNNNNNNNNNNNNNNNNNNNNNNNNNNNNNNNNNNNNNNNNNNNNNNNNNNNNNNNNNNNNNNNNNNNNNNNNNNNNNNNNNNNNNNNNNNNNNNNNNNNNNNNNNNNNNNNNNNNNNNNNNNNNNNNNNNNNNNNNNNNNNNNNNNNNNNNNNNNNNNNNNNNNNNNNNNNNNNNNNNNNNNNNNNNNNNNNNNNNNNNNNNNNNNNNNACCTGTAGCAACACGTGGCATGGAACATTCCCCATCAACGCACACACCACGGGCCCCTGCACRCCCTCCCCGGCGCCCAATTATTCCAGGGCGCTGTGGAGGGTGGCTGCTGAGGAGTACGTGGAGGTTACGCGGGTGGGGGATTTCCACTACGTGACGGGCATGACCACTGACAACGTAAAGTGCCCATGCCAGGTTCCGGCCCCCGAATTCTTCACGGAGTTGGATGGGGTGCGGCTGCACAGGTACGCTCCGCCGTGCAAACCTCTCCTACGGGAGGAGGTCACATTCCAGGTCGGGCTCAACCAATACCTGGTCGGGTCACAGCTCCCATGTGAGCCCGAACCGGATGTRGCGGTGCTCACTTCCATGCTCACTGACCCCTCCCACATCACAGCAGAGACGGCTAAGCGTAGGCTGGCCAGGGGGTCTCCCCCCTCCTTGGCCAGCTCTTCAGCTAGCCAATTGTCTGCGCCTTCCTTGAAGGCGACATGCACTACCCGTCATGACTCCCCAGATGCTGACCTCATCGAGGCCAATCTCCTGTGGCGGCAGGAGATGGGCGGGAATATCACCCGCGTGGAGTCAGAGAACAAGGTAGTAATTCTGGACTCTTTCGAGCCGCTTCGAGCGGAGGAAGATGAGAGGGAAGTGTCCGTTCCGGCGGARATCCTGCGGAGATCCAGGAAATTTCCCCGGGCGATGCCCATATGGGCAAGGCCGGATTATAACCCTCCAYTGATAGAGYCCTGGAAGGACCCGGACTACGTCCCTCCGGTGGTACATGGATGYCCATTGCCACCTACTAAGGYCCCTCCAATACCACCTCCACGGAGGAAAAGGACGGTTGTCCTGACAGAATCCACCGTGTCTTCTGCCTTGGCTGAGCTYGCCACGAAGDCATTCGGCAGCTCCGAATCGTCGGCCGTCGACAGCGGCACGGCGACCGCCCCTCCTGANNNNNNNNNNNNNNNNNNNNNNNNNNNNNNNNNNNNNNNNNNNNNNNNNNNNNNNNNNNNNNNNNNNNNNNNNNNNNNNNNNNNNNNNNNNNNNNNNNNNNNNNNNNNNNNNNNNNNNNNNNNNNNNNNNNNNNNNNNNNNNNNNNNN

>HM106857

TCCGGCTCGTGGCTAAGGGACGTTTGGGACTGGATATGCACGGTGTTAACTGATTTTAAGACCTGGCTCCAGTCCAAGCTCCTGCCGCGGTTGCCAGGAGTCCCCTTCCTCTCATGCCAACGTGGGTACAGGGGCATCTGGCGGGGAGACGGCATCATGCAAACCACCTGCCCATGTGGAGCGCAGATCACCGGACATGTCAAAAACGGTTCCATGAGGATCGTTGGGCCTAGGACCTGYAGCAACACGTGGCATGGAACATTCCCCATCAACGCATACACCACGGGCCCCTGCACACCCTCCCCGGCACCCAATTATTCCAGGGCGCTGTGGAGGGTGACTGCTGAGGAGTACGTGGAGGTTACGCGGGTGGGAGATTTCCACTACGTGACGGGCATGACCACTGACAACGTAAAGTGCCCATGCCAGGTTCCAGCCCCCGAATTCTTCACGGAGGTGGATGGGGTGCGGCTGCACAGGTATGCTCCGCCGTGCAAACCTCTCCTACGGGAGGAGGTCACATTCCAGGTCGGGCTCAACCAATACCTGGTTGGRTCACAGCTCCCATGTGAGCCCGAACCGGATGTGGCGGTGCTCACCTCCATGCTCACCGATCCCTCCCACATCACAGCAGAGGCGGCTAGGCGTAGGCTGGCCAGGGGGTCTCCCCCCTCCTTRGCCAGCTCTTCAGCTAGCCAGTTGTCTGCGCCTTCCTTGAAGGCGACATGCACTACCCGTCATGACTCCCCAGATGCTGACCTCATCGAGGCCAATCTCCTGTGGCGGCAGGAGATGGGCGGGAACATCACCCGCGTGGAGTCAGAGAACAAGGTAGTAATTCTGGACTCTTTCGAGCCGCTTCGAGCGGAGGAAGATGAGAGGGAAGTATCCGTTCCGGCGGAAATCCTGCGGAGATCCAGAAAATTTCCCCGAGCGATGCCCATATGGGCAAGGCCGGATTATAACCCTCCACTGATAGAGTCCTGGAAGGACCCGGACTATGTCCCTCCGGTGGTACATGGGTGCCCATTGCCACCTACTAAGGCCCCTCCAATACCACCTCCACGGAGGAAAAGGACGGTTGTCCTGACAGAATCCACCGTGTCTTCTGCCTTGGCTGAGCTCGCCACAAAGACCTTCGGCAGCTCCGARTCGTCGGCCGTCGACAGCGGCACGGCGACCGCCCCTCCTGACCAGCTCTCCGACNNNNNNNNNNNNNNNNNNNNNNNNNNNNNNNNNNNNNNNNNNNNNNNNNNNNNNNNNNNNNNNNNNNNNNNNNNNNNNNNNNNNNNNNNNNNNNNNNNNNNNNNNNNNNNNNNNNNNNNNNNNNNNNNNNNNNNN

>HM106858

NNNNNNNNNNNNNNNNNNNNNNNNNNNNNNNNNNNNNNNNNNNNNNNNNNNNNNNNNNNNNNNNNNNNNNNNNNNNNNNNNNNNNNNNNNNNNNNNNNNNNNNNNNNNNNNNNNNNNNNNNNNNNNNNNNNNNNNNNNNNNNNNNNNNNNNNNNNNNNNNNNNNNNNNNNNNNNNNNNNNNNNNNNNNNNNNNNNNNNNNNNNNNNNNNNNNNNNNNNNNNNNNNNNNNNNNNNNNNTGCAGCAACACGTGGCATGGGACATTCCCCATCAACGCGTACACCACGGGCCCCTGCWCGCCTTCCCCAGCGCCCAATTATTCYAGGGCGCTGTGGAGGGTGGCTGCTGAGGAGTACGTGGAGRTTACGCGGGTGGGGGATTTCCACTACGTGACGGGCATGACCACTGACAACGTAAAGTGCCCATGCCAGGTTCCGGCCCCCGAATTCTTCACKGARGTGGATGGGGTRCGGYTGCACAGGTAYGCTCCSCCGAGCAAACCTCTTCTACGGGAGGAGGTCACATTCCAGGTCGGGCTCAACCAATACCTGGTCGGGTCACARCTCCCATGTGAGCCCGARCCGGATGTAGCGGTGCTCACTTCCATGCTYACCGACCCCTCCCACATYACAGCAGARACGGCTAAGCGTAGGCTGGCYAGGGGGTCTCCCCCCTCCTTGGCCAGCTCTTCAGCTAGCCAGTTGTCTGCGCCTTCCTTGAAGGCGACATGCACTACCCGTCATGACTCCCCAGATGCTGACCTCATYGAGGCCAATCTCCTGTGGCGRCAGGAGATGGGCGGGAACATCACCCGCGTGGAGTCAGAGAACAAGGTAGTAATTCTGGACTCTTTCGAGCCGCTTCGAGCGGAGGAAGATGAGAGGGAAGTATCCGTTCCGGCGGAAATCCTGCGRAGATCCAGGARATTYCCCCGAGCGMTGCCCATATGGGCAAGGCCGGATTACAACCCTCCACTGATAGAGTCCTGGAAGGACCCGGACTAYGYCCCTCCGGTGGTACAYGGGTGCCCATTGCCACCTACYAAGGCCCCYCCAATACCACCTCCACGGAGGAAAAGGACGGTTGTCCTGACAGAATCCACYGTGTCTTCTGCCTTGGCTGAGCTCGCCACAAAGACCTTCGGCAGCTCYGAATCGTCGGCYRTCGACAGCGGCACGGCGACCGCCCCTCCTNNNNNNNNNNNNNNNNNNNNNNNNNNNNNNNNNNNNNNNNNNNNNNNNNNNNNNNNNNNNNNNNNNNNNNNNNNNNNNNNNNNNNNNNNNNNNNNNNNNNNNNNNNNNNNNNNNNNNNNNNNNNNNNNNNNNNNNNNNNNNNNNNNNNNN

>HM106859

NNNNNNNNNNNNNNNNNNNNNNNNNNNNNNNNNNNNNNNNNNNNNNNNNNNNNNNNNNNNNNNNNNNNNNNNNNNNNNNNNNNNNNNNNNNNNNNNNNNNNNNNNNNNNNNNNNNNNNNNNNNNNNNNNNNNNNNNNNNNNNNNNNNNNNNNNNNNNNNNNNNNNNNNNNNNNNNNNNNNNNNNNNNNNNNNNNNNNNNNNNNNNNNNNNNNNNNNNNNNNNNNNNNNNNNNNNNNNNNNNNNNNNNNNNNNNNNNNNNNNNNNNNNNNNNNCGCRTACACCACGGGCCCTTGCACGCCCTCCCCGGCGCCCAATTACTCCAGGGCGCTGTGGAGGGTGGCTGCTGAGGAGTACGTGGAGGTTACRCGGGTGGGGGATTTCCACTACGTGACGGGCATGACCACTGACAACATAAAGTGCCCATGCCAGGTTCCGGCCCCYGAATTCTTCACGGAGGTGGATGGAGTGCGGCTGCACAGGTACGCTCCGCCGTGCAAACCCCTCYTACGGGAGGASGTCACATTCCAGGTCGGGCTCAACCAATACCTGGTCGGGTCACAGCTCCCATGTGAGCCCGAACCGGATGTRGCRGTGCTCACTTCTATGCTCACCGACCCCTCCCACATCACAGCAGAGACGGCTAAGCGTAGGCTGGCCAGGGGGTCTCCCCCCTCCTTGGCCAGCTCYTCAGCTAGCCAGTTGTCTGCGCTTTCCTTGAAGCCGACDTGCACTGCCCGTCATGGCTCCCCGGATGTTGACCTCGTCGAGGCCAATCTCATGTGGCGGCAGGAGATGGGCGGGAACATCACCCGCGTGGAGTCAGAGAACAAGGTRGTAATYTTGGACTCTTTTGAGCCGCTTCGAGCGGAGGARGATGAGAGGGAGGTRTCCGTTCCGGCGGAAATCCTGCGGAAATCCAGGAAATTTCCCCWAGCGGTGCCCATATGGGCAAGGCCGGATTACAACCCTCCACTGTTAGAGTCCTGGAARCACCCGGACTACGTYCCTCCGGTGGTACAYGGGTGCCCATTGCCACCTACCAAGGCCCCTCCAATACCACCTCCACGGAGGAAAAGGACGGTTGTCCTGACAGARTCCACCGTGTCTTCTGCCTTRGCTGAGCTCGCCACAAAGACCTTCGGCAGCYCCGAAYTGCCGGCCGTCGACARCGGCACGGCGACCGCCCCTCCTGACCAGGTCYCCGACGACGGCGATGCAGGGTCCGATGCTGAGTCGTACTCTTCCATGCCCCCCCTTGAGGGGGAGCCGGGGGATCCTGATCTCAGCGACGGGTCTTGGTCTACCGTGAGTGAGGAWACTGGTGAGGGCCTCGTCTGCTGC

>HM106860

NNNNNNNNNNNNNNNNNNNNNNNNNNNNNNNNNNNNNNNNNNNNNNNNNNNNNNNNNNNNNNNNNNNNNNNNNNNNNNNNNNNNNNNNNNNNNNNNNNNNNNNNNNNNNNNNNNNNNNNNNNNNNNNNNNNNNNNNNNNNNNNNNNNNNNNNNNNNNNNNNNNNNNNNNNNNNNNNNNNNNNNNNNNNNNNNNNNNNNNNNNNNNNNNNNNNNNNNNNNNNNNNNNNNNNNNNNNNNNNNNNNNNNNNNNNNNNNNNNNNNTTCCCCATCAACGCRTACACCACGGGCCCCTGCACGCCCTCCCCGGCGCCCAATTATTCCAGGGCGCTGTGGAGGGTGACTGCTGAGGAGTACGTGGAAGTTACGCGGGTGGGGGATTTCCACTACGTGACGGGCATGACCACTGACAACGTAAAGTGCCCATGCCAGGTTCCGGCCCCCGAATTCTTCACGGAGGTGGATGGGGTGCGGCTGCACAGGTACGCTCCGCCGTGCAAACCTCTCCTACGGGAGGAGGTCACATTCCAGGTCGGGCTCAACCAATACCTGGTCGGGTCACAGCTCCCATGTGAGCCYGAACCGGATGTGACGGTGCTCACTTCCATGCTCACCGACCCCTCCCACATCACAGCAGARGCGGCTAAGCGTAGGCTGGCCAGGGGGTCTCCCCCCTCCTTAGCCAGCTCTTCAGCTAGCCAGTTGTCTGCGCCTTCCTTGAAGGCGACGTGCACTACCCGTCATGACTCCCCAGATGCTGACCTCATCGAGGCCAATCTCCTGTGGCGGCAGGAGATGGGCGGGAACATCACCCGCGTGGAGTCAGAGAACAAGGTAGTAATTCTGGACTCTTTCGAGCCGCTTCGAGCGGAGGAAGATGAGAGGGAAGTATCCGTTCCGGCGGAAATCCTGCGGAGATCCAGGAAGTTTCCCCGAGCGATGCCCATATGGGCAAGGCCGGATTACAACCCTCCACTGGTAGAGTCCTGGAAGGACCCGGACTACGTCCCTCCGGTGGTACATGGGTGCCCATTGCCACCTACTAAGGCCCCCCCAATACCACCTCCACGGAGGAAAAGGACGGTTGTCCTGACAGAATCCACCGTGTCTTCTGCCTTGGCTGAGCTCGCCACAAAGACCTTCGGCAGCTCCGGATCGTCGGCCGTCGACAGCGGCACGGCGACCGCCCCTCNNNNNNNNNNNNNNNNNNNNNNNNNNNNNNNNNNNNNNNNNNNNNNNNNNNNNNNNNNNNNNNNNNNNNNNNNNNNNNNNNNNNNNNNNNNNNNNNNNNNNNNNNNNNNNNNNNNNNNNNNNNNNNNNNNNNNNNNNNNNNNNNNNNNNNNN

>HM106861

TCCGGCTCGTGGCTGAGGGACGTTTGGGACTGGATATGCACGGTGTTAGCTGATTTTAAGACCTGGCTCCAGTCCAAGCTCCTGCCGCGATTACCGGGAGTCCCCTTCCTCTCATGCCAACGTGGGTACAGGGGCATCTGGCGGGGAGACGGCATCATGCAAACCACCTGCCCATGTGGAGCACAGATCACCGGACATGTCAAAAACGGTTCCATGAGGATCGTTGGGCCTAGGACCTGYAGCAACRCGTGGCATGGAACATTCCCCATCAACGCATACACCACGGGCCCCTGCACGCCCTCTCCGGCACCCAATTATTCCAGGGCGCTGTGGAGGGTGGCTGCTGAGGAGTACGTGGAGGTTACGCAGGTGGGGGATTTCCACTACGTGACGGGCATGACCACTGACAACGTAAAGTGCCCATGCCAGGTCCCGGCCCCTGAATTCTTCACAGAGGTGGACGGGGTGCGGCTGCACAGGTACGCTCCGCCGTGCAAACCTCTCCTACGGGAGGAGGTCACATTCCAGGTCGGGCTCAACCAATACCTGGTCGGGTCACAGCTCCCATGTGAGCCCGAACCGGATGTAGCGGTGCTCACCTCCATGCTCACCGACCCCTCCCACATCACAGCAGAGACGGCTAAGCGTAGGTTGGCCAGGGGGTCTCCCCCCTCCTTGGCCAGTTCTTCAGCTAGCCAGTTGTCTGCGCCTTCCTTGAAGGCGACATGCACTACCCGCCATGACTCCCCAGATGCTGACCTCATCGAGGCCAATCTCCTGTGGCGGCAGGAGATGGGCGGGAACATCACCCGCGTGGAGTCAGAGAACAAGGTAGTAATTCTGGACTCTTTCGAGCCGCTTCGAGCGGAGGAAGATGAGAGGGAAGTATCCGTTCCGGCGGAAATCCTGCGAAGATCCAGGAAATTTCCCCGAGCGATGCCCATATGGGCAAGGCCGGATTACAACCCTCCACTGGTGGAGTCCTGGAAGAACCCGGACTACGTCCCTCCGGTGGTACATGGGTGCCCATTGCCACCTACTAAGGCCCCTCCAATACCACCTCCACGGAGGAAAAGGACGGTTGTCCTGACAGAATCCACCGTGTCTTCTGCCTTGGCTGAGCTCGCCACAAAGACCTTCGGCAGCTCCGAATCGTCGGCCGTCGACAGCGGCACGGCGACCGCCCCTCCCGACCAGTCCTCTGGCGACGGCGACGCAGGGTCCGATGTTGAGTCGTACTCCTCCATGCCCCCCCTTGAGGGGGAGCCGGGGGATCCCGATCTCAGCGATGGTTCCTGGTCTACCGTGAGCGAGGAAGCTAGTGAGGACGTCGTCTGCTGC

>HM106862

TCCGGCTCGTGGCTAAGGGACGTTTGGGACTGGATATGCACGGTGTTAACTGATTTTAAGACCTGGCTCCAGTCTAAGCTCCTGCCGCGGTTACCGGGAGTCCCCTTCCTCTCATGCCAACGTGGGTATAAGGGCATCTGGCGGGGAGACGGCATCATGCAAACCACCTGCCCATGTGGAGCGCAGATCACCGGACATGTCAAAAACGGTTCCATGAGGATCGTTGGGCCTAGGACCTGCAGCAACACGTGGCATGGAACATTYCCCATCAACGCATACACCACGGGCCCCTGCACGCCCTCCCCGGCGCTCAATTATTCCAGRGCGCTGTGGAGGGTGGCTGCTGAGGAGTACGTGGAGGTTACGCGGGTGGGGGACTTCCACTACGTGACGGGCATGACCACTGACAACGTAAAGTGCCCATGCCAGGTTCCGGCCCCCGAATTCTTCACGGAGGTGGATGGGGTGCGACTGCACAGGTACGCTCCGCCGTGCAAACCTCTCCTRCGGGATGAGGTCACATTCCAGGTCGGGCTCAACCAATACTTGGTCGGGTCACAGCTCCCATGTGAGCCCGAACCGGATGTAGCGGTGCTCACTTCCATGCTCACCGACCCCTCCCACATCACAGCAGAGACGGCTAAGCGTAGGCTGRCCAGAGGGTCTCCCCCCTCCTTGGCCAGCTCTTCAGCTAGCCAGTTGTCTGCGCCTTCCTTGAAGGCGACATGCACTACCCGTCATGACTCCCCAGATGCCGACCTCATCGAGGCCAATCTCCTGTGGCGGCAGGAGATGGGCGGGAACATTACCCGCGTGGAGTCAGAGAACAAGGTAGTAATTCTGGACTCTTTCGARCCGCTTCGAGCGGARGAAGATGAGAGGGAAGTRTCCGTTCCGGCGGAAATCCTGCGGAGATCCAGGAAATTTCCCCGAGCGATGCCCATATGGGCAAGGCCGGATTACAACCCTCCACTGGTGGAGYCCTGGAAGGACCCGGACTACGTCCCTCCGGTAGTACATGGGTGCCCATTACCACCTACTAAGGCCCCTCCAGTACCACCTCCACGGAGGAAGAGGACGGTTGTCCTGACAGAATCCACCGTGTCTTCCGCCTTGGCTGAGCTCGCCACGAAGACCTTCGGCAGCTCCGGATCGTCGGCCGTTGACAGCGGCACGGCGACCGCCTCTCCTGACCAGCCCTCCGACGACGGCGACGCRNGTCCNNNNNNNNNNNNNNNNNNNNNNNNNNNNNNNNNNNNNNNNNNNNNNNNNNNNNNNNNNNNNNNNNNNNNNNNNNNNNNNACCGTGAGCGAGGAAGCTAGTGAGGACGTCGTCTGCTGT

>HM106863

TCCGGCTCGTGGCTAAGGGATGTTTGGGACTGGATATGCACGGTGTTAACTGACTTTAAGACCTGGCTCCAGTCCAAGCTCCTGCCGCGGTTACCGGGAGTCCCCTTCCTCTCATGCCAACGTGGGTACAAGGGCATTTGGCGGGGGGACGGCATCATGCTAACCACCTGCCCATGTGGAGCGCAGATCACCGGACATGTCAAGAACGGTTCCATGAGGATCGTTGGGCCTGGGACCTGCAGCAACACGTGGCATGGAACATTCCCCATCAACGCATACACCACGGGCCCCTGCACGCCCTCCCCGGCRCCCAATTATTCCAGGGCGCTGTGGAGGGTGGCTGCTGAGGAGTACGTGGAGGTYACGCGGGTGGGGGATTTCCACTACGTGACGGGCATGACCACTGACAACGTAAAGTGCCCATGCCAGGTYCCGGCCCCCGAATTCTTCACGGAGGTGGATGGGGTGCGGCTGCACAGGTACGCTCCGCCATGCARRCCYCTYCTACGGGAGGAGGTCACATTCCAGGTCGGGCTCAACCAATACTTGGTCGGGTCACAGCTCCCATGCGAGCCCGAACCRGATGTAGCGGTGCTCACTTCCATGCTCACCGACCCCTCCCACATCACAGCAGAGRCGGCTAAGCGTAGGCTGGMCAGGGGGTCTCCCCCCTCCTTAGCCAGCTCTTCAGCTAGCCAGTTGTCTGCGCCTTCCTTGAAGGCGACATGCACTACCCGTCATGACTCCCCAGATGCTGACCTCATCGAGGCCAATCTCCTGTGGCGRCAGGAGATGGGCGGGAACATCACCCGCGTGGAGTCAGAGAACAAGGTAGTAATTCTGGACTCTTTCGAGCCGCTTCGAGCGGAGGAAGATGAGAGGGAAGTATCCGTTCCGGCGGARATCCTGCGGAGATCCAGGAAATTYCCCCGAGCGATGCCCATATGGGCAAGGCCGGATTACAACCCTCCACTGGTGGAGTCCTGGAAGGACCCGGACTACGTCCCTCCGGTGGTACATGGGTGCCCATTGCCACCTACTAAGACCCCTCCAATACCACCTCCACGGAGGAARAGGACGGTTGTCCTGACAGAATCCACCGTGTCTTCTGCCTTGGCTGAGCTCGCCACAAAGACCTTCGGCAGCTCCGAATCGTCGGCCGTCGACAGCGGCACGGCGACCGCCCCTCCTGACCAGNNNNNNNNNNNNNNNNNNNNNNNNNNNNNNNNNNNNNNNNNNNNNNNNNNNNNNNNNNNNNNNNNNNNNNNNNNNNNNNNNNNNNNNNNNNNNNNNNNNNNNNNNNNNNNNNNNNNNNNNNNNNNNNNNNNNNNNNNNNNNNNN

>HM106864

TCCGGCTCGTGGCTAAGGGACGTTTGGGACTGGATATGCACGGTGTTAACTGATTTTAAGACCTGGCTCCAGTCCAAGCTCCTGCCGCGGTTACCGGGAGTCCCCTTCCTCTCATGCCAACGTGGGTACAAGGGCATCTGGCGGGGAGACGGCATCATGCAAACCACCTGTCCATGTGGAGCGCAGATCACCGGACATGTCAAAAACGGTTCCATGAGGATCGTTGGGCCTAGGACCTGCAGCAACACGTGGCATGGAACATTCCCCATCAACGCACACACCACGGGCCCCTGCACGCCCTCCCCGGCGCCYAATTATTCYARGGCGCTGTGGAGGGTGGCTGCTGAGGAGTACGTGGAGGTTACGCGGGTGGGGGACTTCCACTACGTGACGGGCATGACCACTGACAACGTAAAGTGCCCATGCCAGGTTCCGGCCCCCGAATTCTTCACGGAGGTGGATGGGGTGCGGCTGCACAGGTACGCTCCGCCGTCCAAACCTCTCCTACGGGAGGAGGTCACATTCCAGGTCGGGCTCAACCAATAYCTGGTBGGGTCACAGCTCCCATGTGAGCCCGAACCGGATGTRGCGGTGCTYACTTCCATGCTCACCGACCCCTCCCACATCACAGCAGAGRCGGCTAAGCGYAGGCTGGCCAGGGGGTCTCCCCCCTCCTTRGCCAGCTCTTCAGCTAGCCAGTTGTCTGCGCCTTCCTTGAAGGCGACATGCACTACCCRTCACGACTCCCCAGAYGCTGACCTCATCGAGGCYAATCTCCTGTGGCGGCAGGAGATGGGCGGGAACATCACCCGCGTGGAGTCAGAGAAYAAGGTAGTAATTYTGGACTCTTTCGAGCCGCTTCGAGCGGAGGAAGATGAGAGGGAAGTGTCCGTTCCGGCGGAAATCCTGCGGAGATCCAGGAAATTTCCCCSRGCGATGCCCATATGGGCGAGGCCGGATTACAACCCTCCACTGSTAGAGYCCTGGAAGGAYCCGGACTACGTCCCTCCGGTGGTACATGGGTGYCCRTTGCCACCTACTAAGGCCCCTCCMRTACCACCTCCACGGAGGAAAAGGACGGTTGTCCTGACAGAATCTACCGTGTCTTCTGCCTTGGCTGAGCTCGCCACAAAGACCTTCGGCAGCTCCGAATCGTCGGCCGYCGACAGCGGYACGGCGACCGCCCCTNNNNNNNNNNNNNNNNNNNNNNNNNNNNNNNNNNNNNNNNNNNNNNNNNNNNNNNNNNNNNNNNNNNNNNNNNNNNNNNNNNNNNNNNNNNNNNNNNNNNNNNNNNNNNNNNNNNNNNNNNNNNNNNNNNNNNNNNNNNNNNNNNNNNNNNNN

>HM106865

TCCGGCTCGTGGCTAAGGGACGTTTGGGACTGGATATGCACGGTGTTAACYGAYTTTAAGACCTGGCTCCAGTCCAAGCTCCTGCCGCGGTTACCGGGAGTCCCCTTCCTCTCATGCCAACGTGGGTACAAGGGCATCTGGCGAGGAGACGGCATYATGCAAACCACCTGCCCATGTGGAGCGCAGATCACCGGACATGTCAAAAACGGTTCCATGAGGATCGTTGGGCCTAGGACCTGCAGCAACACGTGGCATGGAACATTYCCCATCAACGCATACACCACGGGCCCCTGCACGCCCTCCCCGGCGCCCAATTATTCCAGGGCGCTGTGGAGGGTGGCTGCTGAGGAGTACGTGGAGGTTACGCGGGTGGGGGATTTCCACTACGTGACGGGCATGACCACTGACAACKTAAAGTGCCCATGCCAGGTTCCGGCCCCCGAATTCTTCACAGAGGTGGATGGGGTGAAGCTRCACAGGTACGCTCCSCCGTGCARGCCTCTCCTACGGGAGGAGGTCACATTCCAGGTCGGGCTCAACCAATACCTGGTCGGGTCACAGCTCCCATGYGAGCCCGAACCGGATGTARCGGTGCTYACTTCCATGCTCACCGAYCCCTCCCACATCACAGCAGAGRCGGCTAAGCGTAGGCTGGMCAGGGGGTCTCCCCCCTCCYTGGCCAGCTCTTCAGCYAGCCAGTTGTCTGCGCCTTCCTTGAAGGCRACATGCACTACCCGTCAYGACTCCCCAGATGCTGACCTCATCGAGGCCAATCTCCTGTGGCGGCAGGAGATGGGCGGGAACATCACCCGCGTGGAGTCAGAGAACAAGGTAGTAATTCTGGACTCTTTCGAGCCGCTTCGAGCGGAGGAAGAYGAGAGGGAAGTATCCGTTCCGGCGGAAATCCTRCGGASATCYAGGAAATTTCCCCGRGCGATGCCCATATGGGCAAGGCCGGATTACAACCCTCCAYTGGTGGAGTCCTGGAAGGACCCGGACTACGTCCCTCCGGTGGTACAYGGGTGCCCAYTRCCACCTACTAAGGCCCCTCCAATACCACCTCCACGGAGGAAAAGGACGGTTGTCCTGACAGAATCCACYGTGTCTTCYGCCTTGGCTGAGCTCGCCACAAAGACCTTCGGCAGCTCCGAATCGTCGGCCGTCGACAGCGGCACGGCGACCGCCCCTCCTGACCAGCCCTCCGNNNNNNNNNNNNNNNNNNNNNNNNNNNNNNNNNNNNNNNNNNNNNNNNNNNNNNNNNNNNNNNNNNNNNNNNNNNNNNNNNNNNNNNNNNNNNNNNNNNNNNNNNNNNNNNNNNNNNNNNNNNNNNNNNNNNNNNNN

>HM106866

TCCGGCTCGTGGCTAAGGGACGTTTGGGACTGGATATGCACGGTGTTAACTGATTTTAAGACCTGGCTCCAGTCCAAGCTCCTGCCGCGGTTACCGGGAATCCCCTTCTTCTCATGCCAACGTGGGTACAAGGGTATCTGGCGGGGAGACGGCATTATGCAAACCACCTGCCCATGTGGAGCGCAGATCACCGGACATGTCAAAAACGGTTCCATGAGGATCGTTGGGCCTAGGACCTGCAGCAACACGTGGCAYGGAACATTCCCCATCAACGCATACACCACGGGCCCCTGCACGCCCTCCCCGGCGCCCAATTATTCCAGGGCGCTGTGGAGGGTGGCTGCTGAGGAGTACGTGGAGGTTACGCGGGTGGGGGATTTCCACTACGTGACGGGCATGACCACTGACAACGTAAAGTGCCCATGCCAGGTTCCGGCCCCYGAATTCTTCACRGAGRTRGATGGGGTGCGGCTGCACAGGTACGCTCCGCCGTGCARCCCTCTCCTACGGGAGGAGGTCACATTCCAGGTCGGGCTCAACCARTACYTGGTCGGGTCACAGCTCCCATGTGAGCCCGAGCCGGATGTAGCGGTGCTCACTTCCATGCTCACCGACCCCTCCCACATCACAGCAGAGGCGGCTAAGCGTAGGCTGGCCAGGGGGTCTCCCCCCTCTTTAGCCAGCTCTTCAGCTAGCCAGYTGTCTGCGCCTTCCTTGAAGGCGACATGCACTACCCRTCATGACTCCCCAGATGCTGACCTCATCGAGGCCAATCTCCTGTGGCGGCAGGAGATGGGCGGRAACATCACCCGCGTGGAGTCAGAGAACAAGGTAGTAATTCTGGACTCTTTCGAGCCGCTTCGAGCGGAGGAAGATGAGAGGGAARTATCCGTTCCGGCGGAAATCCTGCGGAGACCCAGGAAATTCCCCCGAGCGATGCCCATATGGGCAAGGCCGGATTATAACCCTCCACTGATAGAGTCCTGGAAGGACCCGGACTACGTCCCTCCGGTGGTACATGGGTGCCCATTGCCACCTACTAAGGCCCCTCCAATACCACCTCCACGGAGGAARAGGACGGTYGTCCTGACAGAATCCACCGTGTCTTCTGCCTTGGCTGAGCTCGCCACAAAGACCTTCGGCAGCTCCGAATCGTCGACTGTCGRCAGCGGCACGGCGACCGCCCCTCCTGACCNNNNNNNNNNNNNNNNNNNNNNNNNNNNNNNNNNNNNNNNNNNNNNNNNNNNNNNNNNNNNNNNNNNNNNNNNNNNNNNNNNNNNNNNNNNNNNNNNNNNNNNNNNNNNNNNNNNNNNNNNNNNNNNNNNNNNNNNNNNNNNNNNN

>HM106867

TCCGGCTCGTGGCTAAGGGACGTTTGGGACTGGATATGCACGGTGTTAACTGAYTTTAAGACCTGGCTCCAGTCYAAGCTCCTGCCGCGGTTACCGGGAGTCCCCTTYCTCTCRTGCCAACGTGGGTACAAGGGCATYTGGCGGGGAGACGGCGTSATGCARACCACCTGCCCATGTGGAGCGCAGATCACCGGACATGTCAAAAMCGGTTCCATGAGGATCGTTGGGCCYAGGACCTGCAGCAACACGTGGCATGGRACATTCCCCATCAACGCGTACACCACGGGCCCCTGCACGCCCTCCCCGGCTCCCAATTATTCCAGGGCGCTGTGGAGGGTGGCTGCTGAGGAGTACGTGGAGGTTACGCGGGTGGGGGACTTCCACTACGTGACGGGCATGACCACAGACAACGTRAAGTGCCCATGYCAGGTTCCGGCCCCCGAATTCTTCACGGAGGTGGATGGGGTGCGGCTGCACAGGTACGCTCCGCCGTGCAARCCCCTCCTACGGGAGGAGGTCACATTCCAGGTCGGGCTCAACCAATACCTGGTTGGGTCACAGCTCCCATGYGAGCCCGAACCGGATGTGGCGGTGCTCACTTCCATGCTCACCGACCCCTCCCACATCACAGCAGAGGCGGCTAAGCGTAGGCTGGCCAGGGGGTCTCCCCCCTCCTTAGCCAGCTCTTCAGCTAGCCAGTTGTCTGCGCCTTCCTTGAAGGCGACATGCACTACCCGTCATGACTCCCCAGATGCTGACCTCATCGAGGCCAATCTCCTGTGGCGGCAGGAGATGGGCGGAAACATCACCCGYGTGGAGTCAGAGAACAAGGTAGTAATTCTGGACTCTTTCGARCCGCTTCGAGCGGAGGAAGATGAGAGGGAAGTATCCGTTCCGGCGGAAATCCTGCGGAGATCCAGGAAATTTCCCCGAGCGATGCCCATATGGGCAAGGCCGGATTACAACCCTCCACTGGTAGAGTCCTGGAAGGACCCGGACTACGTCCCTCCGGTGGTACATGGGTGCCCATTGCCACCTACTAAGGCCCCTCCAGTGCCACCTCCACGGAGGAAAAGGACGGTTGTCCTGACAGAATCCACCGTGTCTTCTGCCTTGGCTGAGCTCGCCACAAAGACCTTCGGCAGCTCCGAATCGTCGGCCGTTGAYAGCGGTACGGCGAGCGGCCCTCCTGATCAGCCCTCCGACGACGGCGACGCAGGATCCGACGTTGAGTCGTATTCCTCCATGCCCCCCCTTGAGGGRGAGCCGGGGGATCCCGATCTCAGCGACGGGTCTTGGTCTACYGTGAGCGAGGAAGCTAGTGAGGACGTCGTCTGCTGC
