# Supplementary material for: Effect of Immune Pressure on Hepatitis C Virus Evolution: Insights From a Single-Source Outbreak
Source: Hepatology. 2011 Feb;53(2):396–405. doi: 10.1002/hep.24076 (PMC3044208; doi:10.1002/hep.24076)
Supplement: Supplementary file 10 [file hep0053-0396-SD10.doc]

>HM106868

TCGATGTCCTACACATGGACRGGCGCYYTGATCACRCCATGCTCCGCGGAGGAAAGCAAGCTGCCCATCAACGCGTTGAGCAACTCTTTGCTGCGYCAYCAYAACATGGTCTATGCCACRACRTCTCGCAGCGCRAGCCAGCGGCAGAAGAAGGTCACTTTTGACAGRCTGCAGGTCCTGGACGACCATTACCGGGACGTGCTCAAGGAGATGAAGGCGAAGGCGTCCACGGTTAAGGCTAAACTTCTATCCGTAGARGAAGCCTGCATGCTGACGCCCCCACACTCGGCCAAATCCAAATTTGGCTATGGGGCAAAGGACGTCCGGAACCTATCCAGCAAGGCCATTAACCACATCCRCTCCGTGTGGAAGGACTTGYTGGAAGACACTGAGACACCAATTGAYACYACCATCATGGCAAAAAATGAGGTTTTCTGYGTCCAACCAGAGAAAGGAGGCCGCAARCCAGCTCGCCTTATCGTRTTCCCAGAYTTGGGGGTTCGTGTGTGCGAGAARATGGCYCTTTATGAYGTGGTCTCCACCCTTCCTCAGGCCGTGATGGGCYCCTCATACGGGTTCCAGTACTCTCCTGGACAGCGGGTCGAGTTCCTGGTGAATGCCTGGAAAAAGAAGAARAACCCTATGGGCTTCGCRTATGACACCCGCTGTTTTGACTCAACGGTCACCGAGAGTGACATCCGTGTTGAGGAGTCAATCTACCAATGTTGTGACCTGGCCCCCGAAGCCAGACAGGCCATAAGGTCGCTCACAGAGCGGCTTTATATCGGGGGTCCCCTGACTAATTCAAAAGGGCAGAACTGCGGTTATCGTCGGTGCCGCGCGAGCGGTGTGCTGACGACCAGCTGCGGCAATACCCTCACATGTTACTTAAAGGCCTCTGCAGCCTGTCGAGCCGCCAAGCTCCAGGACTGCACGATGCTCGTGTGCGGAGACGACCTTGTCGTTATCTGTGAAAGCGCGGGAACCCAGGAGGACGCGGCGAGCCTACGAGTCTTCACGGAGGCTATGACTAGGTACTCTGCCCCCCCCGGGGACCCGCCTAAACCAGAATACGACTTGGAGTTGATAACATCATGCTCCTCCAATGTGTCGGTCGCGCACGATGCATCTGGCAAAAGGGTGTACTACCTCACCCGTGACCCCACCACCCCACTTGCACGGGCTGCGTGGGAGACAGCTAGACACACTCCAGTCAACTCCTGGCTAGGCAACATCATCATGTATGCGCCCACCTTATGGGCAAGGATGATCCTGATGACTCACTTCTTCTCCATCCTTCTAGCTCAGGAACAACTTGAAAAGGCTCTAGATTGTCAGATCTATGGGGCCTGTTACTCCATTGAGCCACTTGACCTACCTCAGATCATTCAGCGACTCCATGGNNNNNNNNNNNNNNNNNNNNNNNNNNNNNNNNNNNNNNNNNNNNNNNNNNNNNNNNNNNNNNNNNNNNNNNNNNNNNNNNNNNNNNNNNNNNNNNNNNNNNNNNNNNNNNNNNNNNNNNNNNNNNNNNNNNNNNNNNNNNNNNNNNNNNNNNNNNNNNNNNNNNNNNNNNNNNNNNNNNNNNNNNNNNNNNNNNNNNNNNNNNNNNNNNNNNNNNNNNNNNNNNNNNNNNNNNNNNNNNNNNNNNNNNNNNNNNNNNNNNNNNNNNNNNNNNNNNNNNNNNNNNNNNNNNNNNNNNNNNNNNNNNNNNNNNNNNNNNNNNNNNNNNNNNNNNNNNNNNNNNNNNNNNNNNNNNNNNNNNNNNNNNNNNNN

>HM106869

NNNATGTCCTACACATGGACAGGCGCCCTAATCACGCCATGCTCCGCGGAGGAAAGCAAGCTGCCCATCAACGCGTTGRGCAACTCTTTGCTGCGTCACCACAACATGGTCTATGCCACAACATCTCGCAGCGCAAGCCAGCGGCAGAAGAAGGTCACCTTTGACAGACTGCAGGTCCTGGACGACCATTACCGGGACGTGCTCAAGGAGATGAAGGCGAAGGCGTCCACAGTTAAGGCTAAACTTCTATCCGTAGAAGAAGCCTGCATGCTGACGCCCCCACACTCGGCCAAATCCAAATTTGGCTATGGGGCAAAGGACGTCCGGAACCTATCCAGCAAGGCCATTGACCACATCCGCTCCGTGTGGAAGGACTTGTTGGAAGACACTGAGACACCAATTGACACCACCATCATGGCAAAAAATGAGGTTTTCTGCGTCCAACCAGAGAAAGGAGGCCGCAAGCCAGCTCGCCTTATCGTATTCCCAGACTTGGGGGTTCGTGTGTGCGAGAAAATGGCCCTTTATGACGTGGTCTCCACCCTTCCTCAGGCCGTGATGGGCTCCTCATACGGGTTCCARTACTCTCCTGGACAGCGGGTCGAGTTCCTGGTGAATGCCTGGAAAAAAAAGAAAAACCCTATGGGCTTCGCATATGACACCCGCTGTTTTGACTCAACGGTCACCGAGAGTGACATCCGTGTTGAGGAGTCAATCTACCAATGTTGTGACCTGGCCCCCGAAGCCAGACAGGCCATAAGGTCGCTCACAGAGCGGCTTTATATCGGGGGTCCCCTGACTAACTCAAAAGGGCAGAACTGCGGTTATCGCCGGTGCCGCGCGAGCGGCGTGCTGACGACCAGCTGCGGTAATACCCTCACATGTTACTTGAAGGCCTCTGCAGCCTGTCGAGCTGCCAAGCTCCAGGACTGCACGATGCTCGTGTGCGGAGACGACCTTGTCGTTATCTGTGAGAGCGCGGGAACCCAGGAGGATGCGGCGAGCCTACGAGTCTTCACGGAGGCTATGACTAGGTACTCTGCCCCCCCCGGGGACCCGCCCAAACCAGAATACGACTTGGAGTTGATAACATCATGCTCCTCCAATGTGTCGGTCGCGCACGATGCATCTGGCAAAAGGGTGTACTACCTCACCCGTGACCCCACCACCCCACTTGCACGGGCTGCGTGGGAGACAGCTAGACACACTCCAGTCAACTCCTGGCTAGGCAACATCATCATGTATGCGCCCACCTTATGGGCAAGGATGATTCTGATGACTCACTTCTTCTCCATCCTTCTAGCTCAGGAACAACTTNNNNNNNNNNNNNNNNNNNNNNNNNNNNNNNNNNNNNNNNNNNNNNNNNNNNNNNNNNNNNNNNNNNNNNNNNNNNNNNNNNNNNNNNNNNNNNNNNNNNNNNNNNNNNNNNNNNNNNNNNNNNNNNNNNNNNNNNNNNNNNNNNNNNNNNNNNNNNNNNNNNNNNNNNNNNNNNNNNNNNNNNNNNNNNNNNNNNNNNNNNNNNNNNNNNNNNNNNNNNNNNNNNNNNNNNNNNNNNNNNNNNNNNNNNNNNNNNNNNNNNNNNNNNNNNNNNNNNNNNNNNNNNNNNNNNNNNNNNNNNNNNNNNNNNNNNNNNNNNNNNNNNNNNNNNNNNNNNNNNNNNNNNNNNNNNNNNNNNNNNNNNNNNNNNNNNNNNNNNNNNNNNNNNNNNNNNNNNNNNNNNNNNNNNNNNNNNNNNNNNNNNNNNNNNNNNNNNNNNNNNNNNNNNNNNNNNNN

>HM106870

TCGATGTCCTACACGTGGACAGGCGCCCTGATCACGCCATGCTCCGCGGAGGAAAGCAAGCTGCCCATCAACGCGTTGAGCAACTCCTTGCTGCGTCACCACAACATGGTCTATGCCACAACATCTCGCAGCGCAAGCCAGCGGCAGAGGAAGGTCACCTTCGACAGACTGCAGGTCCTGGACGACCATTACCGGGACGTGCTCAAGGAGATGAAGGCGAAGGCGTCCACAGTTAAGGCTAAACTTCTATCTGTAGAAGAAGCCTGCATGCTGACGCCCCCACAYTCGGCCAAATCCAAATTTGGCTATGGGGCAAAGGACGTCCGGAACCTATCCAGCAAGGCCATTAACCACATCCACTCCGTGTGGAAGGACTTGCTGGAAGACACTGAGACACCAATTGACACCACCATCATGGCAAAAAATGAGGTTTTCTGTGTCCAACCAGAGAAAGGAGGCCGCAAACCAGCTCGCCTTATCGTRTTCCCAGACTTGGGGGTTCGTGTGTGCGAGAAAATGGCCCTTTATGACGTGGTCTCCACCCTTCCTCAGGCCGTGATGGGCCCCTCATACGGGTTYCAGTACTCTCCTGGACAGCGGGTCGAGTTCCTGGTGARTGCCTGGAAAAAAAAGAAGAACCCYATGGGCTTCGCATATGACACCCGCTGTTTYGACTCAACAGTCACYGAGAGTGACATCCGTGTTGAGGAGTCAATCTACCAATGTTGTGACTTGGCCCCCGAAGCCAGACAGGCCATAAGGTCGCTCACAGAGCGGCTTTATATCGGGGGYCCCCTGACTAAYTCAAAGGGGCAGAACTGCGGTTATCGCCGGTGCCGCGCRAGCGGTGTGCTGACGACTAGCTGCGGTAATACCCTCACATGTTACTTGAAGGCCTCTGCAGCCTGTCGAGCTGCCAAGCTCCAGGACTGCACGATGCTCGTGTGCGGAGACGACCTTGTCGTTATCTGTGAAAGCGCGGGAACCCAGGAGGATGCGGCAAGCCTACGAGTCTTCACGGAGGCTATGACTAGGTACTCTGCCCCCCCCGGGGACCCGCCCAAACCAGAATACGACTTGGAGTTGATAACATCATGCTCCTCCAATGTGTCGGTCGCGCACGATGCATCTGGCAAAAGGGTGTACTACCTCACCCGTGACCCCACCACCCCACTTGCACGGGCTGCGTGGGAGACAGCTAGACACACTCCAGTCAACTCCTGGCTAGGCAACATCATCATGTATGCGCCCACTTTATGGGCGAGGATGATTCTGATGACTCATTTCTTCTCCATCCTCCTAGCTCAGGAACAGCTTGAAAAGGCTCTAGATTGCCAGATCTACGGGGCCTGTTACTCCATTGAGCCACTTGACCTACCTCAGATCNNNNNNNNNNNNNNNNNNNNNNNNNNNNNNNNNNNNNNNNNNNNNNNNNNNNNNNNNNNNNNNNNNNNNNNNNNNNNNNNNNNNNNNNNNNNNNNNNNNNNNNNNNNNNNNNNNNNNNNNNNNNNNNNNNNNNNNNNNNNNNNNNNNNNNNNNNNNNNNNNNNNNNNNNNNNNNNNNNNNNNNNNNNNNNNNNNNNNNNNNNNNNNNNNNNNNNNNNNNNNNNNNNNNNNNNNNNNNNNNNNNNNNNNNNNNNNNNNNNNNNNNNNNNNNNNNNNNNNNNNNNNNNNNNNNNNNNNNNNNNNNNNNNNNNNNNNNNNNNNNNNNNNNNNNNNNNNNNNNNNNNNNNNNNNNNNNNNNNNNNNNNNNNNNNNNNNNNNNNNNNNNNNN

>HM106871

TCGATGTCCTACACATGGACAGGCGCCCTGATCACRCCATGCTCCGCGGAGGAAAGCAAGCTGCCCATCAACGCGTTGAGCAACTCTTTGCTGCGTCACCACAATATGGTCTATGCCACAACATCTCGCAGCGCAAGCCAGCGGCAGAAGAAGGTCACCTTTGACAGACTGCAGGTCCTGGACGACCATTACCGGGACGTGCTCAAGGAGATGAAGGCGAAGGCGTCCACGGTTAAGGCTAAACTTCTACCCGTAGAAGAAGCCTGCAARCTGACGCCCCCACACTCGGCCAAATCCAAATTTGGCTATGGGGCAAAGGACGTCCGGAACCTATCCAGCAAGGCCGTTAACCACATCCTCTCCGTGTGGAAGGACTTACTGGAAGACACTGAGACACCAATTGACACCACCATCATGGCAAAAAATGAGGTTTTCTGCGTCCAACCAGAAAAAGGAGGCCGYAAGCCAGCTCGCCTTATCGTATTCCCAGACTTGGGGGTTCGTGTGTGCGAGAARATGGCYCTTTATGACGTAGTCTCCACCCTTCCTCAGGCCGTGATGGGCTCCTCATAYGGGTTCCAGTACTCTCCTGGACAGCGGGTCGAGTTCTTGGTGAATGCCTGGAAAAARAAGAAAAACCCTATGGGCTTCGCATATGACACCCGCTGTTTTGACTCAACGGTCACYGAGAGTGACATCCGCGTTGAGGARTCAATCTACCAATGTTGTGACTTGGCCCCCGAAGCTAGACAGGCTATAAGGTCGCTCACAGAGCGGCTTTATATCGGGGGTCCCCTGACTAACTCAAAAGGGCAGAACTGCGGTTATCGCCGGTGCCGCGCGAGCGGYGTGCTGACGACTAGCTGCGGTAATACCCTCACATGTTACTTGAAGGCCTCTGCAGCCTGTCGAGCTGCCAAGCTCCAGGACTGCACGATGCTCGTGTGCGGAGACGACCTCGTCGTTATCTGTGAAAGCGCGGGAACCCAGGAGGACGCGGCGAGCCTACGAGTCTTCACGGAGGCTATGACTAGGTACTCTGCCCCCCCCGGGGACCCGCCCAAACCAGAATACGACTTGGAGTTGATAACATCATGCTCCTCCAATGTGTCGGTCGCGCACGAYGCATCTGGCAAAAGGGTGTACTACCTCACCCGTGAYCCCACCACCCCACTTGCGCGGGCTGCGTGGGAGACAGCTAGACACACTCCAGTCAACTCCTGGCTAGGCAACATCATCATGTACGCGCCCACCTTATGGGCAAGGATGATCCTGATGACTCAYTTCTTCTCCATCCTTCTAGCTCAGGAACAACTCGAAAAGGCTCTAGATTGYCAGATCTACGGGGCCTGTTACTCCATTGAGCCACTTGACCTACCTCAGATCATTCAGCGACTCCATGGTCTTAGCGCATTCTCACTCCATAGTTACTCTCCAGGGGAAATCAATAGGGTGGCTGCATGCCTCAGGAAACTTGGGGTACCACCCTTGCGAGTCTGGAGACATCGGGCCAGAAGTGTCCGCGCTAAGCTACTGTCCCRGGGRGGGAGGGCTGCCAMCTGTGGCAAATACCTCTTCAACTGGGCAGTAAGGACCAAGCTCAAANNNNNNNNNNNNNNNNNNNNNNNNNNNNNNNNNNNNNNNNNNNNNNNNNNNNNNNNNNNNNNNNNNNNNNNNNNNNNNNNNNNNNNNNNNNNNNNNNNNNNNNNNNNNNNNNNNNNNNNNNNNNNNNNNNNNNNNNNNNNNNNNNNNNNNNNNNNNNNNNNNNNNNNN

>HM106872

TCGATGTCCTACACRTGGACAGGCGCTCTGATCACACCATGCTCCGCGGAGGAAAGCAMGTTGCCCATCAACGCGTTGAGCAACTCCTTGCTGCGTCACCACAACATGGTCTATGCCACAACATCCCGCAGCGCAAGCCAGCGGCARAAGAAGGTCACCTTTGACAGACTGCAGGTCCTGGATGACCATTACCAGGACGTGCTCAAGGAGATGAAGGCGAAGGCGTCCACGGTTAAGGCTAAACTTCTATCCGTAGAAGAAGCCTGCATGCTGACACCCCCACATTCGGCCAAATCCAAATTTGGCTATGGGGCGAAGGACGTCCGGAACCTATCCAGCAAGGCCATTAACCACATCCGCTCCGTGTGGAAGGACTTGCTGGAAGACACTGAGACACCAATTGACACCACCATCATGGCAAAAAATGAGGTTTTCTGCGTCCAACCAGAGAAGGGAGGCCGCAAGCCAGCTCGCCTTATCGTATTCCCAGACTTGGGGGTTCGTGTGTGCGAGAAAATGGCCCTYTATGACGTGGTCTCCACCCTTCCTCAGGCCGTGATGGGCTCCTCATACGGGTTCCAGTACTCTCCTGGACAGCGGGTCGAGTTCCTGGTGAATGCCTGGAAGAAAAAGAAGAACCCTATGGGCTTCGCATATGACACCCGCTGTTTTGACTCAACGGTCACTGAGAGTGACATCCGTGTTGAGGAGTCAATCTACCAATGTTGTGACTTGGCCCCCGAAGCCAGACAGGCTATAAGGTCGCTCACAGAGCGGCTTTACATCGGGGGTCCCCTGACTAACTCAAAAGGGCAGAACTGCGGTTATCGCCGGTGCCGCGCGAGCGGTGTGCTGACGACTAGCTGCGGTAATACCCTCACATGTTACTTGAAGGCCTCTGCAGCCTGTCGAGCTGCCAAGCTCCAGGACTGCACGATGCTCGTGTGCGGGGACGACCTTGTCGTTATCTGTGAGAGCGCGGGGACCCAGGAGGACGCGGCGAGCCTACGAGTCTTCACGGAGGCTATGACTAGGTACTCTGCCCCCCCTGGGGACCCGCCCAAACCAGAATACGACTTGGAGTTAATAACATCATGCTCCTCCAATGTGTCGGTCGCGCACGATGCATCTGGCAAAAGGGTGTATTACCTCACCCGCGACCCCACCACCCCGCTCGCACGGGCTGCGTGGGAGACAGCTAGACACACTCCTGTTAACTCCTGGCTAGGCAACATCATCATGTATGCGCCCACCTTATGGGCAAGGATGATCCTGATGACTCACTTCTTCTCCATCCTTCTAGCTCAGGAACAACTTGAAAAGGCTCTAGATTGTCAGATCTACGGGGCCTGTTACTCCATTGAGCCACTCGACCTACCTCAGATCATTCAGCGACTCCATGGNNNNNNNNNNNNNNNNNNNNNNNNNNNNNNNNNNNNNNNNNNNNNNNNNNNNNNNNNNNNNNNNNNNNNNNNNNNNNNNNNNNNNNNNNNNNNNNNNNNNNNNNNNNNNNNNNNNNNNNNNNNNNNNNNNNNNNNNNNNNNNNNNNNNNNNNNNNNNNNNNNNNNNNNNNNNNNNNNNNNNNNNNNNNNNNNNNNNNNNNNNNNNNNNNNNNNNNNNNNNNNNNNNNNNNNNNNNNNNNNNNNNNNNNNNNNNNNNNNNNNNNNNNNNNNNNNNNNNNNNNNNNNNNNNNNNNNNNNNNNNNNNNNNNNNNNNNNNNNNNNNNNNNNNNNNNNNNNNNNNNNNNNNNNNNNNNNNNNNNNNNNNNNNNNN

>HM106873

NNNATGTCCTAYACATGGACGGGCGCCCTGATCACGCCATGCTCCGCGGAGGAAAGCAAGCTGCCCATCAACGCGTTGAGCAACTCTYTGCTGCGTCACCACAACATGGTCTATGCCACAACATCYCGCAGCGCAAGCCARCGGCAGAAGAAGGTCACCTTTGACAGACTGCAGGTCCTGGACGACCATTACCGGGACGTGCTCAAGGAGATGAAGGCGAAGGCGTCCACRGTTAAGGCTAAACTTCTATCCRTAGAAGAAGCCTGCAAGCTGACGCCCCCACACTCGGCCAAATCCAAATTTGGTTATGGGGCAAAGGACGTCCGGAACCTATCCAGCAAGGCCGTYAACCACATCCTCTCCGTGTGGAAGGACTTGCTGGAAGACACTGAGACACCAATTGACACCACCATCATGGCAAARAATGAGGTTTTCTGYGTCCAACCAGAGAAAGGAGGCCGCAAGCCAGCTCGCCTTATCGTATTCCCAGACTTGGGGGTTCGTGTRTGCGAGAAGATGGCCCTTTAYGACGTRGTCTCCACCCTTCCTCAGGCCGTGATGGGCTCCTCATACGGGTTCCAGTACTCTCCTGGACAGCGGGTCGAGTTCCTGGTGAATGCCTGGAAGAAAAAGAARAACCCYATGGGYTTCGCATATGACACCCGYTGTTTTGACTCAACGGTCACCGAGAGTGACATCCGTGTTGAGGAGTCAATCTACCAATGTTGTGACTTGGCCCCCGAAGCCAGACAGGYCATAAGGTCGCTCACAGAGCGGCTTTATATCGGGGGTCCCCTGACTAACTCAAAGGGGCAGAACTGCGGTTATCGCCGSTGCCGCGCGAGCGGCGTGCTGACGACTAGCTGCGGTAATACCCTCACATGTTACTTGAAGGCCTCTGCAGCCTGTCGAGCTGCCAAGCTCCAGGACTGCACGATGCTCGTGTGCGGAGACGACCTCGTCGTWATCTGTGAAAGCGCGGGAACCCARGAGGACGCGGCGAGCCTACGAGTNNNNACGGAGGCTATGACYAGGTACTCTGCYCCCCCCGGGGACCCGCCCMAACCAGAATACGACTTGGAGTTGATAACATCATGCTCCTCCAATGTGTCGGTCGCGCACGATGCATCTGGCAARAGGGTGTACTACCTCACCCGTGACCCCACCACCCCACTTGCGCGGGCYGCGTGGGAGACAGCTAGACACACTCCAGTCAAYTCCTGGCTAGGCAACATCATYATGTATGCGCCCACCYTRTGGGCAAGGATGATCCTGATKACYCACTTCTTCTCCATCCTTCTAGCYCAGGAACAACTAGAAAAGGCTYTAGATTGYCAAATCTACGGGGCCTGTTACTCCATTGAGCCACTCGACCTACCTCAGATCATTCAGCGACTCCATGGTCTTAGCGCATTTTCACTCCATAGTTACTCTCCAGGTGAAATCAATAGGGTGGCTGCGTGCCTCAGAAARCTTGGGGTACCACCCTTGCGAGTCTGGAGACATCGGGCCAGAAGTGTTCGCGCTAAGCTACTGTCCCRGGGGGGGAGGGCTGCCAACTGTGGCAAATACCTCTTCAACTGGGCAGTAARGACCAAGCTCAAACNNNNNNNNNNNNNNNNNNNNNNNNNNNNNNNNNNNNNNNNNNNNNNNNNNNNNNNNNNNNNNNNNNNNNNNNNNNNNNNNNNNNNNNNNNNNNNNNNNNNNNNNNNNNNNNNNNNNNNNNNNNNNNNNNNNNNNNNNNNNNNNNNNNNNNNNNNNNNNNNNNNNNNN

>HM106874

TCGATGTCCTACACATGGACAGGCGCCCTGATYACGCCATGCTCCGCGGAGGARAGCAAGCTGCCCATCAACGCGTTGAGCAACTCTTTGCTGCGYCACCACAACATGGTCTAYGCCACAACATCTCGCAGCGCAAGCCAGCGGCAGAAGAAGGTCACYTTTGACAGAYTGCAGGTCCTGGACGACCATTACCGGGACGTGCTCAAGGAGATGAAGGCGAAGGCGTCCACGGTTAAGGCTAAACTTCTATCCGTAGAAGAAGCCTGCATGCTGACGCCCCCACAYTCGGCCAAATCCAAATTTGGCTATGGGGCAAAGGACGTCCGGARCCTATCCCGCAAGGCCACTGACCACATCCGCTCCGTGTGGAAGGACTTGCTGGAAGACACTGARACACCAATTGACACCACCATCATGGCAAAAAATGAGGTTTTCTGCGTCCAACCAGAGAAAGGAGGCCGCAAGCCAGCTCGCCTTATCGTATTCCCAGACTTGGGGGTTCGTGTGTGCGAGAAAATGGCCCTTTATGACGTGGTCTCCACCCTTCCTCAGGCCGTGATGGGCTCCTCATACGGGTTCCAGTACTCTCCTGGACAGCGGGTCGAGTTCCTGGTGAATGCCTGGAAAAAAAAGAAAAACCCTATGGGCTTCGCATATGACACCCGCTGTTTTGACTCAACGGTCACCGAGAGTGACATCCGTGTTGAGGAGTCAATCTACCAATGTTGTGACTTGGCCCCCGAAGCCAGACAGGCCATAAGGTCGCTCACAGAGCGGCTTTACATCGGGGGTCCCCTGACTAACTCAAAAGGGCAGAACTGCGGTTATCGCCGGTGCCGCGCAAGCGGTGTGCTGACGACCAGCTGCGGTAACACCCTCACATGTTACTTGAAGGCCTCTGCAGCCTGTCGAGCTGCCAAGCTCCAGGACTGCACGATGCTCGTGTGCGGAGACGACCTTGTCGTTATCTGTGAAAGCGCGGGAACCCAGGAGGACGAGGCGAGCCTACGAGTCTTCACGGAGGCTATGACTAGGTACTCTGCCCCCCCCGGGGACCCGCCCAAACCAGAATACGACTTGGAGTTGATAACATCATGCTCCTCCAATGTGTCGGTCGCGCACGATGCATCTGGCAAAAGGGTGTACTACCTCACCCGTGACCCCACCACCCCGCTTGCACGGGCTGCGTGGGAGACAGCTAAACACACTCCAGTCAATTCCTGGCTAGGCAACATCATCATGTAYGCGCCCACCCTATGGGCAAGGATGATCCTGATGACTCACTTCTTCTCCATCCTTCTAGCYCAGGAACAACTTGAAAAGGCTCTAGATTGTCAGATCTACGGGGCCTGYTACTCCATTGAGCCACTTGACCTACCTCAGATCATTCAACGACTCCATGGNNNNNNNNNNNNNNNNNNNNNNNNNNNNNNNNNNNNNNNNNNNNNNNNNNNNNNNNNNNNNNNNNNNNNNNNNNNNNNNNNNNNNNNNNNNNNNNNNNNNNNNNNNNNNNNNNNNNNNNNNNNNNNNNNNNNNNNNNNNNNNNNNNNNNNNNNNNNNNNNNNNNNNNNNNNNNNNNNNNNNNNNNNNNNNNNNNNNNNNNNNNNNNNNNNNNNNNNNNNNNNNNNNNNNNNNNNNNNNNNNNNNNNNNNNNNNNNNNNNNNNNNNNNNNNNNNNNNNNNNNNNNNNNNNNNNNNNNNNNNNNNNNNNNNNNNNNNNNNNNNNNNNNNNNNNNNNNNNNNNNNNNNNNNNNNNNNNNNNNNNNNNNNNNNN

>HM106875

TCGATGTCCTACACATGGACAGGCGCCCTTATCACGCCATGCTCCGCGGAGGAAAGCAAGCTGCCCATCAACGCGTTGAGCAACTCCTTGCTGCGTCACCACAACATGGTCTATGCCACAACATCTCGCAGCGCAAGCCARCGGCAGAAGAAGGTCACYTTTGACAGACTGCAGGTCCTGGACGACCAYTACCGGGACGTGCTTAAGGAGATGAAGGCGAAGGCGTCCACGGTTAAGGCTAAGCTTCTATCCGTAGAAGAAGCCTGCATGCTGACGCCCCCACACTCGGCCAAATCCAAATTTGGCTATGGGGCAAAGGACGTCCGGAACCTATCCAGCAAGGCCATTAACCACATCCGCTCCGTGTGGAAGGACTTGCTGGAAGACACTGAGACACCAATTGACACCACCATCATGGCAAAGAATGAGGTTTTCTGCGTCCAACCAGAGAAAGGAGGCCGCAAGCCAGCTCGCCTTATCGTATTCCCAGACTTGGNNNNNNNNNNNNNNNNNNNNNNNNNNNNNNNNNNNNNNNNNNNNACCCTTCCTCAGGCCGTGATGGGCTCCTCATACGGATTCCAGTACTCTCCTGGACAGCGGGTCGAGTTCCTGGTGAATGCCTGGAAGAAAAAGAAGAACCCTATGGGCTTCGCATATGACACCCGCTGCTTTGACTCAACAGTCACCGAGAGTGACATCCGTGTTGAGGAGTCAATCTACCAATGTTGTGACTTGGCCCCCGAAGCCAGACAGGCCATAAGGTCGCTCACAGAGCGGCTTTATATCGGGGGTCCCCTGACTAACTCAAAAGGGCAGAACTGCGGTTATCGCCGGTGCCGCGCGAGCGGTGTGCTGACGACTAGCTGCGGTAATACCCTCACATGTTATTTGAAGGCCTCTGCAGCCTGTCGAGCTGCCAAGCTCCAGGACTGCACGATGCTCGTGTGCGGAGACGACCTTGTCGTTATCTGTGAAAGCGCGGGAACCCAGGAGGACGCGGCGAGCCTACGAGTCTTCACGGAGGCTATGACTAGGTACTCTGCCCCCCCCGGGGACCCGCCCAAACCAGAATACGATTTGGAGTTGATAACATCATGCTCCTCCAATGTGTCGGTCGCGCACGATGCATCTGGTAAAAGGGTGTACTACCTCACCCGTGACCCCACCACCCCACTTGCACGGGCTGCGTGGGAGACAGCTAGACACACTCCAGTCAACTCCTGGCTAGGCAACATCATCATGTATGCGCCCACCTTATGGGCAAGGATGATCCTGATGACTCACTTTTTCTCCATCCTTCTAGCTCAGNNNNNNNNNNNNNNNNNNNNNNNNNNNNNNNNNNNNNNNNNNNNNNNNNNNNNNNNNNNNNNNNNNNNNNNNNNNNNNNNNNNNNNNNNNNNNNNNNNNNNNNNNNNNNNNNNNNNNNNNNNNNNNNNNNNNNNNNNNNNNNNNNNNNNNNNNNNNNNNNNNNNNNNNNNNNNNNNNNNNNNNNNNNNNNNNNNNNNNNNNNNNNNNNNNNNNNNNNNNNNNNNNNNNNNNNNNNNNNNNNNNNNNNNNNNNNNNNNNNNNNNNNNNNNNNNNNNNNNNNNNNNNNNNNNNNNNNNNNNNNNNNNNNNNNNNNNNNNNNNNNNNNNNNNNNNNNNNNNNNNNNNNNNNNNNNNNNNNNNNNNNNNNNNNNNNNNNNNNNNNNNNNNNNNNNNNNNNNNNNNNNNNNNNNNNNNNNNNNNNNNNNNNNNNNNNNNNNNNNNNNNNNNNNNNNNNNNNNNNNNNNNN

>HM106876

TCGATGTCCTACACATGGACAGGCGCCCTGATCACGCCATGCTCCGCGGAGGAAAGCAAGCTGCCCATCAACGCGTTGAGCAAYTCTTTGCTGCGTCACCACAAYATGGTCTATTCCACAACATCTCGCAGCGCAAGCCAGCGRCAGAAGAAGGTCACCTTTGACAGACTGCAGGTCCTGGACGACCATTACCGGGACGTGCTCAAGGAGATGAAGGCGAAGGCGTCCACAGTTAAGGCTAAACTTCTATCCRTAGAAGARGCCTGCAAGCTGACGCCCCCACACTCGGCCAGATCCAAATTTGGCTATGGGGCAAAGGACGTCCGGAACCTATCCAGCAAGGCCGTTAACCACATCCGCTCCGTGTGGAAGGACTTGCTGGAAGACACTGAGACACCGATTGACACCACCATCATGGCAAAAAATGAGGTTTTCTGCGTCCAACCAGAGAAAGGAGGCCGCAAGCCAGCTCGCCTTATCGTATTCCCAGACTTGGGGGTTCGTGTGTGCGAGAAAATGGCCCTTTATGACGTGGTCTCCACCCTYCCTCAGGCCGTGATGGGCTCCTCATACGGGTTCCAGTACTCTCCTGGACAGCGGGTCGAGTTCCTGGTGAATGCCTGGAAAAAAAAGAAAAACCNNNNNNNNNNNNNNNNNNNNNNNNNNNNNNNNNNNNNNNNNNNNNNNNNNNNNNNNNNNNNNNNNNNNNNNNNNNNNNNNNNNNNNNNNNNNNNNNNNNNNNAAGCCAGACAGGCCATACGGTCGCTCACAGAGCGGCTTTATATCGGGGGTCCCCTGACTAATTCAAAAGGGCAGAACTGCGGTTATCGCCGGTGCCGCGCGAGCGGTGTGCTGACGACCAGCTGCGGTAATACCCTCACATGTTACTTGAAGGCCTCTGCAGCCTGTCGAGCTGCTAAGCTCCAGGACTGCACGATGCTCGTGTGCGGAGACGACCTTGTCGTTATCTGCGAAAGCGCGGGAACCCAGGAGGACGCGGCGAGCCTACGAGTCTTCACGGAGGCTATGACTAGGTACTCTGCCCCCCCYGGGGACCCGCCCAAACCAGAATACGACTTGGAGTTGATAACATCATGCTCCTCTAATGTGTCGGTCGCGCACGATGCATCTGGCAAAAGGGTGTACTACCTCACCCGTGACCCCACCACCCCACTTGCACGGGCTGCGTGGGAGACAGCTAGACACACTCCAGTCAACTCCTGGCTAGGCAACATCATCATGTATGCGCCCACCYTGTGGGCAAGGATGATCCTGATGACTCACTTCTTCTCCATCCTTCTAGCTCAGGAACAGCTTGAAAAGGCTCTGGATTGTCAGATCTACGGGGCCTGTTACTCCATTGAACCACTTGACCTACCTCAGATCATTCAGCGACTCCATGGNNNNNNNNNNNNNNNNNNNNNNNNNNNNNNNNNNNNNNNNNNNNNNNNNNNNNNNNNNNNNNNNNNNNNNNNNNNNNNNNNNNNNNNNNNNNNNNNNNNNNNNNNNNNNNNNNNNNNNNNNNNNNNNNNNNNNNNNNNNNNNNNNNNNNNNNNNNNNNNNNNNNNNNNNNNNNNNNNNNNNNNNNNNNNNNNNNNNNNNNNNNNNNNNNNNNNNNNNNNNNNNNNNNNNNNNNNNNNNNNNNNNNNNNNNNNNNNNNNNNNNNNNNNNNNNNNNNNNNNNNNNNNNNNNNNNNNNNNNNNNNNNNNNNNNNNNNNNNNNNNNNNNNNNNNNNNNNNNNNNNNNNNNNNNNNNNNNNNNNNNNNNNNNNNN

>HM106877

TCGATGTCCTACACATGGACGGGTGCCCTGATCACACCATGCTCCGCGGAGGAAAGCAAGCTGCCCATYAACGCGTTGAGCAATTCTTTGCTGCGTCACCACAACATGGTCTATGCCACAACATCTCGCAGTGCAAGCCAGCGGCAGAAGAAGGTCACCTTTGACAGACTGCAGGTCCTGGACGACCATTACCGGGACGTGCTCAAGGAGATGAAGGCGAAGGCGTCCACRGTTAAGGCCAAACTTCTATCYGTAGAAGAAGCCTGCATGCTGACGCCCCCACACTCGGCCAAATCCAAATTTGGCTATGGGGCAAAGGACGTCCGGAACCTATCCAGCAAGGCCACTAACCACATCCGCTCCGTGTGGAAGGACTTGYTGGAAGACACTGAGACACCAATTGACACTACCATCATGGCAAAAAATGAGGTTTTCTGCGTCCAACCAGAGAAAGGAGGCCGCAAGCCAGCTCGCCTTATCGTATTCCCAGATTTGGGGGTTCGTGTGTGCGAGAAAATGGCCCTTTATGACGTGGTCTCCACCCTTCCTCAGGCCGTGATGGGCCCCTCATACGGGTTCCAGTACTCTCCTGGACAGCGGGTCGAGTTCCTGGTGAATGCCTGGAAAAAAAAGAAAAACCCTATGGGCTTCGCATATGACACCCGCTGTTTCGACTCAACGGTCACCGAGAGTGACATCCGTGTTGAGGAGTCAATCTACCAATGTTGTGATTTGGCCCCCGAAGCCAGACAGGCCATAAGGTCGCTCACAGAGCGGCTTTATATCGGGGGTCCCCTGACCAAYTCAAAAGGGCAGAACTGCGGTTATCGCCGGTGCCGCGCGAGCGGCGTGCTGACGACCAGCTGCGGTAATACCCTCACATGTTAYTTGAAGGCCTCTGCAGCCTGTCGAGCTGCCAAGCTCCAGGACTGCACGATGCTCGTGTGCGGAGACGACCTTGTCGTTATCTGTGAAAGCGCGGGAACCCAGGAGGAYGCGGCGAGCCTACGAGTYTTCACGGAGGCTATGACTAGGTACTCTGCCCCCCCCGGGGACCCGCCCAAACCAGAATACGACTTGGAGTTGATAACATCATGCTCCTCCAATGTGTCGGTCGCGCACGATGCATCTGGCAAAAGGGTGTACTATCTCACCCGTGACCCCACCACCCCACTTGCACGGGCTGCGTGGGAGACGGCTAAACACTCYCCAGTCAACTCATGGCTAGGCAACATCATCATGTATGCGCCCACCTTATGGGCAAGGATGATCCTGATGACTCACTTCTTCTCCATCCTTCTAGCTCAAGAACAACTTGAAAAGGCTATAGATTGTCAGATCTACGGGGCCTATTACTCCATTGAGCCACTTGACCTACCTCAGATCATTCAGCGACTCCATGGTCTTAGCGCATTTTCACTCCATAGTTACTCTCCAGGTGAAATCAATAGGGTGGCTGCATGCCTCAGAAAACTTGGGGTACCACCCTTGCGAGTYTGGAGACATCGGGCCAGAAGTGTCCGCGCTAAGCTTCTGTCCCAGGGGGGGAGGGCTGCCAACTGTGGCAAATACCTCTTCAACTGGGCAGTAAGGACCAAGCTCAAACNNNNNNNNNNNNNNNNNNNNNNNNNNNNNNNNNNNNNNNNNNNNNNNNNNNNNNNNNNNNNNNNNNNNNNNNNNNNNNNNNNNNNNNNNNNNNNNNNNNNNNNNNNNNNNNNNNNNNNNNNNNNNNNNNNNNNNNNNNNNNNNNNNNNNNNNNNNNNNNNNNNNNNN

>HM106878

TCGATGTCCTACACATGGACAGGCGCCCTGATCACGCCATGCTCCGCGGAGGAAAGCAAGCTSCCCATCAACGCGYTGAGCAACTCTTTKCTGCGTCACCACAACRTGGTCTATGCCACAACATCKCGCAGCGCAAGCCAGCGGCAGAAGAAGGTCACCTTTGACAGACTGCAGGTCCTGGRCGACCATTACCGGGACGTGCTCAAGGAGATGAAGGCGAAGGCGTCCACAGTTAAGGCTAGACTTCTACCCGTAGAAGAAGCCTGYAAGCTGACGCCCCCACAYTCGGCCAAATCCAAATTTGGCTATGGGGCAAAGGACGTCCGGAACCTATCCAGCAAGGCCGTYAACCACATCCGCTCCGTGTGGAAGGACTTGCTGGAAGACACTGAGACACCAATTGACACCACCATCATGGCAAARAATGAGGTTTTYTGCGTCCAACCAGAGAAAGGAGGCCGCAAGCCAGCTCGCCTTATCGTDTTCCCAGACTTGGGGGTTCGTGTGTGCGAGAAAATGGCYCTTTACGACGTGGTCTCCACCCTYCCTCAGGCCGTGATGGGCTCCTCATACGGGTTCCAGTACTCTCCTGGACAGCGGGTCGAGTTCCTGGTGAATGCCTGGAAAAAAAAGAAGAACCCTATGGGCTTCGCATATGACACCCGCTGTTTTGACTCAACGGTCACCGAGAGTGACATCCGTGTTGAGGAGTCAATCTACCAATGTTGTGACTTGGCCCCCGAAGCCAGACAGGCCATAAGGTYGCTCACAGAGCGGCTTTATATCGGGGGTCCCCTRACTAATTCAAAGGGGCAGAACTGCGGYTATCGCCGGTGCCGCGCGAGCGGCGTGCTGACGACCAGCTGCGGTAATACCCTCACATGTTACTTGAAGGCCTCTGCAGCCTGTCGAGCTGCCAAGCTCCAGGACTGCACGATGCTCGTGTGCGGAGACGACCTYGTCGTTATCTGYGAAAGCGCGGGAACCCAGGAGGACGCGGCARSCCTACGAGTCTTCACGGAGGCTATGACTAGGTACTCTGCCCCCCCCGGGGACCCGCCCAAACCAGAATACGACTTGGAGYTGATAACATCATGCTCCTCCAATGTGTCGGTCGCGCACGATGCATCTGGCAAAAGGGTGTACTAYCTCACCCGTGACCCCACCACCCCACTTGCGCGGGCTGCGTGGGAGACAGCTAGACACACTCCAGTCAAYTCCTGGCTAGGCAACATCATCATGTATGCGCCCACCTTATGGGCAAGGATGATTCTGATGACTCACTTCTTCTCCATCCTTCTAGCTCAGGAACAACTTGAAAAGGCTCTAGATTGTCAGATCTACGGGGCCTGTTACTCCATTGAGCCACTTGACCTACCTCAGATCATTCAGCGACTCCATGGTCTTAGCGCATTTTCACTCCATAGTTACTCTCCAGGTGAAATCAATAGGGTGGCTGCRTGCCTCAGAAAACTTGGGGTACCACCCTTGCGAGTCTGGAGACATCGGGCCAGAAGTGTCCGCGCTAAGCTACTGTCCCAAGGGGGGAGGGCTGCCAACTGTGGCAAATAYCTCTTCAACTGGGCAGTAAGGACCAAGCTCAANNNNNNNNNNNNNNNNNNNNNNNNNNNNNNNNNNNNNNNNNNNNNNNNNNNNNNNNNNNNNNNNNNNNNNNNNNNNNNNNNNNNNNNNNNNNNNNNNNNNNNNNNNNNNNNNNNNNNNNNNNNNNNNNNNNNNNNNNNNNNNNNNNNNNNNNNNNNNNNNNNNNNNNNN

>HM106879

NNNNNGTCCTACACATGGACAGGCGCCCTGATCACGCCATGCGCCGCGGAGGAAAGCAAGCTGCCCATCAACGCGTTGAGCAACTCCTTGCTGCGTCACCACAACATGGTCTAYGCCACAACATCTCGCAGCGCAAGCCAGCGGCAGAAGAAGGTCACCTTTGACAGACTGCAGGTCCTGGATGATCATTACCGGGACGTGCTCAAGGAGATGAAGGTGAAGGCGTCCACGGTTAAGGCTAAACTTCTATCCGTAGAAGAAGCCTGCATGCTGACGCCCCCACACTCGGCCAAATCCAAATTTGGCTATGGGGCAAAGGACGTCCGGAACCTATCCAGTAAGGCCATTAACCACATCCGCTCCGTGTGGAAGGACTTGCTGGAAGACACTGAGACACCAATTGACACCACCATCATGGCAAAAAATGAGGTTTTCTGCGTCCAACCAGAGAAAGGAGGCCGCAAGCCAGCTCGCCTTATCGTGTTCCCAGACTTGGGGGTTCGTGTGTGCGAGAAAATGGCCCTTTATGATGTGGTCTCCACCCTTCCTCAGGCCGTGATGGGCTCCTCATACGGGTTCCAGTACTCTCCTGGACAGCGGGTCGAGTTCCTGGTGAATGCCTGGAAAAAAAAGAAAAACCCTATGGGCTTCGCATATGACACCCGTTGTTTTGACTCAACGGTCACCGAGAGTGACATCCGTGTTGAGGAGTCAATCTACCAATGTTGTGACTTGGCCCCCGAGGCCAGACAGGCCATAAGGTCGCTCACAGAGCGGCTTTATATCGGGGGTCCCCTGACTAACTCAAAAGGGCAGAACTGCGGTTATCGCCGGTGCCGCGCGAGCGGTGTGCTGACGACTAGCTGCGGTAATACCCTCACATGTTACTTGAAGGCCTCTGCAGCCTGTCGAGCTGCCAAGCTCCAGGACTGCACGATGCTCGTGTGCGGAGACGACCTTGTCGTTATCTGTGAAAGCGCGGGAACCCAGGAGGACGCGGCAAGCCTACGAGTCTTCACGGAGGCTATGACTAGGTACTCTGCCCCCCCCGGGGACCCGCCCAAACCAGAATACGACTTGGAGTTGATAACATCATGCTCCTCCAACGTGTCGGTCGCGCACGACGCATCTGGCAAGAGGGTGTACTACCTCACCCGTGACCCCACCACCCCACTTGCGCGGGCTGCGTGGGAGACAGCTAGACACACTCCAGTCAATTCCTGGCTAGGCAACATCATCATGTATGCGCCCACCCTATGGGCAAGGATGATCCTGATGACTCACTTCTTCTCCATCCTTCTAGCTCAGGAACAACTTGAAAAGGCTCTAGATTGTCAGATYTACGGGGCCTGTTACTCCATTGAGCCACTTGACCTACCTCAGATCATTCAACGACTCCATGGNNNNNNNNNNNNNNNNNNNNNNNNNNNNNNNNNNNNNNNNNNNNNNNNNNNNNNNNNNNNNNNNNNNNNNNNNNNNNNNNNNNNNNNNNNNNNNNNNNNNNNNNNNNNNNNNNNNNNNNNNNNNNNNNNNNNNNNNNNNNNNNNNNNNNNNNNNNNNNNNNNNNNNNNNNNNNNNNNNNNNNNNNNNNNNNNNNNNNNNNNNNNNNNNNNNNNNNNNNNNNNNNNNNNNNNNNNNNNNNNNNNNNNNNNNNNNNNNNNNNNNNNNNNNNNNNNNNNNNNNNNNNNNNNNNNNNNNNNNNNNNNNNNNNNNNNNNNNNNNNNNNNNNNNNNNNNNNNNNNNNNNNNNNNNNNNNNNNNNNNNNNNNNNNNN

>HM106880

NNNATGTCCTACACATGGACAGGCGCCCTGATCACGCCATGCTCCGCGGAGGAAAGCAAGCTACCCATCAACGCGTTGRGCAACTCTTTGCTGCGTCACCAYAACATGGTCTATGCYACAACATCTCGCAGCGCAAGCCARCGGCAGAAGAAGGTCACCTTTGACAGACTGCAGGTCCTGGACGACCATTACCRGGACGTGCTCAAGGAGATGAAGGCGAAGGCGTCCACGGTTAAGGCTAGACTTCTACCYGTAGAAGAAGCCTGCAARCTGACGCCCCCACACTCGGCCAAATCCAAATTTGGCTATGGGGCAAAGGACGTCCGGAACCTATCCAGCAAGGCCGTTAACCACATCCTCTCCGTGTGGAAGGACTTGCTGGAAGACACTGAGACACCAATTGACACCACCATCATGGCAAAAAATGAGGTTTTCTGCGTCCAACCGGAGAAAGGAGGCCGCAAGCCAGCTCGCCTTATCGTATTCCCGGATTTGGGRGTTCGTGTGTGCGAGAAAATGGCCCTTTATGACGTAGTCTCCACCCTTCCTCAGGCCGTGATGGGCTCCTCATACGGGTTCCAGTACTCTCCTGGACAGCGGGTCGAGTTCCTGGTGAATGCCTGGAAAAARAAGAARAACCCTATGGGCTTCGCATATGACACCCGCTGTTTTGACTCAACGGTCACCGAGAGTGACATCCGTGTTGAGGAGTCAATCTACCAATGTTGTGACTTGGCCCCCGAAGCCAGACAGGCTATAAGGTCGCTCACAGAGCGGCTTTATATCGGGGGTCCCCTGACTAAYTCAAAGGGGCAGAACTGCGGTTATCGCCGGTGCCGCGCGAGCGGCGTGCTGACGACCAGCTGCGGTAATACCCTCACATGTTACTTGAAGGCCTCTGCAGCCTGTCGAGCTGCYAAGCTCCAGGACTGCACRATGCTCGTGTGCGGAGACGACCTTGTCGTTATCTGTGAAAGCGCGGGAACCCAGGAGGACGCGGCGAGCCTACGAGTCTTCACGGAGGCTATGACTAGGTACTCTGCCCCCCCCGGGGACCCGCCCAAGCCAGAATACGACTTGGAGTTGATAACATCATGCTCCTCCAATGTGTCGGTCGCGCACGATGCATCTGGCAAAAGGGTGTACTATCTCACCCGAGACCCCACCACCCCACTTGCGCGGGCTGCGTGGGAGACAGCTAGACACACTCCTGTCAACTCCTGGCTAGGCAACATCATCATGTATGCGCCCACCTTGTGGGCAAGGATGATCCTGATGACTCACTTCTTCTCCATCCTTCTAGCTCAGGAACAACTTGAAAAGGCTCTAGATTGTCAGATCTACGGGGCCTGTTACTCCATTGAGCCACTTGACCTACCTCARATCATTCAGCGACTCCATGGNNNNNNNNNNNNNNNNNNNNNNNNNNNNNNNNNNNNNNNNNNNNNNNNNNNNNNNNNNNNNNNNNNNNNNNNNNNNNNNNNNNNNNNNNNNNNNNNNNNNNNNNNNNNNNNNNNNNNNNNNNNNNNNNNNNNNNNNNNNNNNNNNNNNNNNNNNNNNNNNNNNNNNNNNNNNNNNNNNNNNNNNNNNNNNNNNNNNNNNNNNNNNNNNNNNNNNNNNNNNNNNNNNNNNNNNNNNNNNNNNNNNNNNNNNNNNNNNNNNNNNNNNNNNNNNNNNNNNNNNNNNNNNNNNNNNNNNNNNNNNNNNNNNNNNNNNNNNNNNNNNNNNNNNNNNNNNNNNNNNNNNNNNNNNNNNNNNNNNNNNNNNNNNNNN

>HM106881

TCGATGTCCTACACATGGACAGGCGCCCTGATCACACCATGCTCCGCGGAGGAAAGCAAGCTGCCCATCAACGCGTTGAGCAACTCTTTGCTGCGTCACCACAACATGGTTTATGCCACAACATCTCGCAGCGCAAGCCAGCGGCAGAAGAAGGTCACCTTTGACAGACTGCAGGTCCTGGATGACCATTACCGGGACGTGCTCAAGGAGATGAAGGCGAAGGCGTCCACRGTTAAGGCTAAACTTCTATCCGTAGAAGAAGCCTGCAAGCTGACGCCCCCACACTCGGCCAGATCCAAATTTGGCTAYGGGGCAAAGGACGTCCGGAACCTATCCAGCAAGGCCATTAACCACATCCGCTCCGTGTGGAAGGACTTGCTGGAAGACACTGAGACACCAATTGACACTACCATCATGGCAAAAAATGAGGTTTTCTGCGTCCAACCAGAGAAAGGAGGCCGCAAGCCAGCTCGCCTTATCGTATACCCAGACTTGGGGGTTCGTGTGTGCGAGAAAATGGCCCTTTATGACGTGGTCTCCACCCTTCCTCAGGCCGTGATGGGCTCCTCATACGGGTTCCAGTACTCTCCTGGACAGCGGGTCGAGTTCCTGGTGAATGCCTGGAAAAAAAAGAAGAACCCTATGGGCTTCGCATATGACACCCGCTGTTTTGACTCAACGGTCACCGAGAGTGACATCCGTGTTGAGGAGTCAATCTACCAATGTTGTGACTTGGNNNNNNNNNNNNNNNNNNNNNNNNNNNNNNNNNNNNNNNNNNNNNNNNNNNNNNNNCCCCTGACTAACTCAAAAGGGCAGAACTGCGGCTATCGCCGGTGCCGCGCGAGCGGTGTGCTGACGACCAGCTGCGGTAATACCCTCACATGTTACTTGAAGGCCTCTGCAGCCTGTCGAGCTGCCAAGCTCCAGGACTGCACGATGCTTGTGTGCGGAGACGACCTYGTCGTTATCTGTGAAAGCGCGGGAACCCAGGAGGACGCGGCGAGCCTACGAGTCTTCACGGAGGCTATGACTAGGTACTCTGCCCCCCCCGGGGACCCGCCCAAACCAGAATACGACTTGGAGTTGATAACATCATGCTCCTCCAATGTGTCGGTCGCGCACGATGCATCTGGCAAAAGGGTGTACTACCTCACCCGTGACCCCACCACCCCACTTGCACGGGCTGCGTGGGAGACAGCTAGACACACTCCAGTCAACTCCTGGCTAGGCAACATCATCATGTATGCGCCCACCTTATGGGCAAGGATGATCCTGATGACTCACTTCTTCTCCATCCTTCTAGCACAGGAACAACTTRAAAAANNNNNNNNNNNNNNNNNNNNNNNNNNNNNNNNNNNNNNNNNNNNNNNNNNNNNNNNNNNNNNNNNNNNNNNNNNNNNNNNNNNNNNNNNNNNNNNNNNNNNNNNNNNNNNNNNNNNNNNNNNNNNNNNNNNNNNNNNNNNNNNNNNNNNNNNNNNNNNNNNNNNNNNNNNNNNNNNNNNNNNNNNNNNNNNNNNNNNNNNNNNNNNNNNNNNNNNNNNNNNNNNNNNNNNNNNNNNNNNNNNNNNNNNNNNNNNNNNNNNNNNNNNNNNNNNNNNNNNNNNNNNNNNNNNNNNNNNNNNNNNNNNNNNNNNNNNNNNNNNNNNNNNNNNNNNNNNNNNNNNNNNNNNNNNNNNNNNNNNNNNNNNNNNNNNNNNNNNNNNNNNNNNNNNNNNNNNNNNNNNNNNNNNNNNNNNNNNNNNNNNNNNNNNNNNNNNNNNNNNNNNNNNNNNN

>HM106882

NNNATGTCYTACACATGGACAGGCGCCCTGATCACRCCRTGCTCCGCGGARGAAAGCAAGCTGCCCATCAACGCGTTGAGCAACTCTTTGCTGCGTCACCAYAACATGGTCTATGCCACAACATCTCGCAGCGCAAGCCARCGGCAGAAGAAGGTCACCTTTGACAGACTGCAGGTCCTGGACGACCATTACCGGGACGTGCTCAAGGAGATGAAGGCGAAGGCGTCCACMGTCAAGGCTAARCTTCTATCCGTAGAAGAAGCCTGCAAGCTGACGCCCCCACACTCGGCCAGATCCAAATTTGGCTATGGGGCAAAGGACGTCCGGAACCTATCCAGCAAGGCCATTAACCACATCCGCTCCGTGTGGAAGGACTTGCAGGAAGACACTGAGACACCAATTGACACCACCATCATGGCAAAGAATGAGGTTTTCTGCATCCAACCAGAGAARGGAGGCCGCAAGCCAGCTCGCCTTATCGTATTCCCAGACTTGGGGGTYCGTGTGTGCGAGAAAATGGCCCTYTATGACGTGGTCTCCACCCTTCCTCAGGCCGTGATGGGCTCCTCATACGGRTTCCAGTACTCTCCYGGACAGCGGGTCGAGTTCCTGGTRAATGCCTGGAARAAAAAGAAGAACCCTATGGGCTTCGCATATGACACCCGCTGTTTTGACTCAACGGTCACYGAGAGTGACATCCGTGTTGAGGAGTCAATCTACCAATGTTGTGACYTGGCCCCCGAAGCCAGACAGGCCATAARGTCGCTCACAGAGCGGCTYTAYATCGGGGGTCCCCTGACTAACTCAAAAGGGCAGAACTGCGGYTATCGCCGGTGCCGCGCGAGCGGCGTGCTGACGACYAGCTGCGGTAATACCCTCACATGTTACTTGAAGGCCTCTGCAGCCTGTCGAGCTGCCAAGCTCCAGGACTGCACGATGCTCGTGTGCGGAGACGACCTTGTCGTTATCTGTGAAAGTGCGGGAACCCAGGAGGACGCGGCGARCCTACGAGTCTTCACGGAGGCTATGACTAGGTAYTCTGCCCCCCCCGGGGACCCGCCCAAACCAGAATACGACTTGGAGYTGATAACATCATGCTCCTCCAATGTGTCGGTCGCGCACGATGCATCTGGCAAAAGGGTGTACTACCTCACCCGTGACCCCACCACCCCAYTWGCACGGGCTGCGTGGGAGACAGCTAGACACACTCCAGTCAACTCCTGGCTAGGCAACATCATCATGTATGCGCCCACCTTATGGGCAAGGATGATCCTGATGACYCAYTTCTTCTCCATCCTTCTAGCTCAGGAACAACTTGAAAAGGCTCTAGATTGTCAGATCTACGGGNNNNNNNNNNNNNNNNNNNNNNNNNNNNNNNNNNNNNNNNNNNNNNNNNNNNNNNNNNNNNNNNNNNNNNNNNNNNNNNNNNNNNNNNNNNNNNNNNNNNNNNNNNNNNNNNNNNNNNNNNNNNNNNNNNNNNNNNNNNNNNNNNNNNNNNNNNNNNNNNNNNNNNNNNNNNNNNNNNNNNNNNNNNNNNNNNNNNNNNNNNNNNNNNNNNNNNNNNNNNNNNNNNNNNNNNNNNNNNNNNNNNNNNNNNNNNNNNNNNNNNNNNNNNNNNNNNNNNNNNNNNNNNNNNNNNNNNNNNNNNNNNNNNNNNNNNNNNNNNNNNNNNNNNNNNNNNNNNNNNNNNNNNNNNNNNNNNNNNNNNNNNNNNNNNNNNNNNNNNNNNNNNNNNNNNNNNNNNNNNNNNNNNNNNNNNNNNNNNNNNNNNNN

>HM106883

TCGATGTCCTACACATGGACAGGCGCCCTGATCACGCCATGCTCCGCGGAGGAAAGCAAGCTGCCCATCAACGCGTTGAGCAACTCTTTGCTGCGTCACCACAACATGGTCTATGCCACAACATCTCGCAGCGCAAGCCAGCGGCAGAARAAGGTCACATTTGACAGACTGCAGGTCCTGGACGACCATTACCGGGAYGTGCTCAAGGACATGAAGGCGAAGGCGTCCACRGTTAAGGCMAAACTTCTATCCGTGGAAGAAGCCTGCATGCTGACGCCCCCACACTCGGCCAAATCCAAATTTGGCTATGGGGCAAAGGACGTCCGGAACCTATCCAGCAAGGCCATTAACCACATCCAGTCCGTGTGGAAGGACTTGCTGGAAGACACTGAGACACCAATTGACACCACCATCATGGCGAAAAATGAGGTTTTCTGCGTCCAACCAGAGAAAGGAGGCCGCAAGCCAGCTCGCCTTATCGTGTTCCCAGACTTGGGGGTTCGTGTGTGCGAGAAAATGGCCCTYTATGATGTGGTCTCCACCCTTCCTCAGGCCGTGATGGGCTCCTCATACGGGTTCCAGTACTCTCCTGGACAGCGGGTCGAGTTCCTGGTGAATGCCTGGAAGAAAAAGAARAACCCTATGGGCTTCGCATATGACACCCGCTGTTTTGACTCAACGGTCACCGAGAGTGACATCCGTGTTGAGGAGTCAATCTACCAATGTTGTGACTTGGCCCCCGAAGCCAGACAGGCCATAAGGTCGCTCACAGAGCGGCTTTATATCGGGGGTCCCCTGACTAACTCAAAAGGGCAGAACTGCGGTTATCGCCGGTGCCGCGCGAGCGGTGTGCTGACGACCAGCTGCGGTAATACCCTCACATGTTACTTGAAGGCCTCTGCAGCCTGTCGAGCTGCCAAGCTCCAGGACTGCACGATGCTCGTGTGCGGAGACGACCTTGTCGTTATCTGTGAAAGCGCGGGAACCCAGGAGGACGCGGCGAGCCTACGAGTCTTCACGGAGGCTATGACTAGGTACTCTGCCCCCCCCGGGGACCCGCCCAAGCCAGAATACGACTTGGAGTTGATAACATCATGTTCCTCCAATGTGTCGGTCGCGCACGATGCATCTGGCAAAAGGGTGTACTACCTCACCCGTGACCCCACCACCCCACTTGCACGGGCGGCGTGGGAGACAGCTAGACACACTCCAGTTAACTCCTGGCTAGGCAACATCATCATGTATGCGCCCACCTTATGGGCAAGGATGATCCTGATGACTCACTTCTTCTCCATCCTTCTAGCTCAGGAACAACTTGAAAAGGCTCTAGATTGTCAGATCTACGGGGCCTGTTACTCCATTGAGCCACTTGACCTACCTCAGATCATTCAGCGACTCCATGGNNNNNNNNNNNNNNNNNNNNNNNNNNNNNNNNNNNNNNNNNNNNNNNNNNNNNNNNNNNNNNNNNNNNNNNNNNNNNNNNNNNNNNNNNNNNNNNNNNNNNNNNNNNNNNNNNNNNNNNNNNNNNNNNNNNNNNNNNNNNNNNNNNNNNNNNNNNNNNNNNNNNNNNNNNNNNNNNNNNNNNNNNNNNNNNNNNNNNNNNNNNNNNNNNNNNNNNNNNNNNNNNNNNNNNNNNNNNNNNNNNNNNNNNNNNNNNNNNNNNNNNNNNNNNNNNNNNNNNNNNNNNNNNNNNNNNNNNNNNNNNNNNNNNNNNNNNNNNNNNNNNNNNNNNNNNNNNNNNNNNNNNNNNNNNNNNNNNNNNNNNNNNNNNNN

>HM106884

TCGATGTCCTACACATGGACAGGCGCCCTGATCACGCCATGCTCCGCGGAGGAAAGCAAGCTGCCCATCAACGCGTTGAGCAACTCTYTGCTGCGTCACCACAACATGGTTTATGCCACAACATCTCGCAGCGCAAGCCAGCGGCAGAAGAAGGTCACCTTTGACAGACTGCAAGTCCTGGACGACCATTACCGGGACGTGCTCAAGGAGATGAAGGCGAAGGCGTCTACAGTTAAGGCTAAGCTTCTATCCGTAGAAGAAGCCTGCAAGCTGACGCCCCCACACTCGGCCAAATCCAAATTTGGCTATGGGGCAAAGGACGTCCGGAACCTATCCAGCAAGGCCGTTAACCACATCCTCTCCGTGTGGAAGGACTTGCTGGAAGACACTGAGACACCAATTGATACCACCATCATGGCAAAAAATGAGGTTTTCTGCGTCCAACCAGAGAAAGGAGGCCGCAAGCCAGCTCGCCTTATCGTATTCCCAGACTTGGGGGTTCGTGTGTGCGAGAAAATGGCCCTTTACGACGTAGTCTCCACTCTTCCTCAGGCCGTGATGGGCTCCTCATACGGGTTCCAGTACTCTCCTGGACAGCGGGTCGAGTTCCTGGTGAATGCCTGGAAAAAGAAGAAAAACCCTATGGGCTTGGCATATGACACCAGTTGCTTTGACTCAACGGTCACYGAGAGTGACATCCGTGTTGAGGAGTCAATCTACCAATGTTGTGACCTGGCCCCCGAAGCCAGACAGGCCATAAGGTCGCTCACAGAGCGGCTTTATATCGGGGGTCCCCTGACTAACTCAAAGGGGCAAAACTGCGGTTATCGCCGGTGCCGCGCGAGCGGTGTACTGACGACCAGCTGCGGTAATACCCTCACATGTTACTTGAAGGCCTCTGCAGCCTGTCGAGCTGCCAAGCTCCAGGACTGCACGATGCTCGTGTGCGGAGACGACCTYGTCGTTATCTGTGAGAGCGCGGGAACCCAAGAGGACGCGGCGAGCCTACGAGTCTTCACGGAGGCTATGACTAGGTACTCTGCCCCCCCTGGGGACCCGCCCAARCCAGAATACGACTTGGAGTTGATAACATCATGCTCCTCCAATGTGTCGGTCGCGCACGATGCATCTGGCAAAAGGGTATACTACCTCACCCGTGACCCCACCACCCCACTTGCGCGGGCTGCGTGGGAGACAGCTAGACACACTCCAGTCAACTCCTGGCTAGGCAACATCATCATGTATGCGCCCACCTTGTGGGCAAGGATGATCCTGATGACTCACTTTTTCTCCATCCTTCTAGCTCAGGAACAACTTGAAAAGGCTCTAGATTGTCAGATCTACGGGGCCTGTTACTCCATTGAGCCACTTGACCTACCTCAGATCATTCAGCGACTCCATGGNNNNNNNNNNNNNNNNNNNNNNNNNNNNNNNNNNNNNNNNNNNNNNNNNNNNNNNNNNNNNNNNNNNNNNNNNNNNNNNNNNNNNNNNNNNNNNNNNNNNNNNNNNNNNNNNNNNNNNNNNNNNNNNNNNNNNNNNNNNNNNNNNNNNNNNNNNNNNNNNNNNNNNNNNNNNNNNNNNNNNNNNNNNNNNNNNNNNNNNNNNNNNNNNNNNNNNNNNNNNNNNNNNNNNNNNNNNNNNNNNNNNNNNNNNNNNNNNNNNNNNNNNNNNNNNNNNNNNNNNNNNNNNNNNNNNNNNNNNNNNNNNNNNNNNNNNNNNNNNNNNNNNNNNNNNNNNNNNNNNNNNNNNNNNNNNNNNNNNNNNNNNNNNNNN

>HM106885

TCGATGTCCTACACATGGACAGGCGCCYTGATCACGCCRTGCTCCGCGGAGGAAAGCAAGCTGCCCATCAACGCGTTGAGCAACTCTTTGCTGCGTCACYRCAACAYGATCTATGCCACAACATCTCGCAGCGCRAGCCWGCGGCAGAAGAAGGTCACCTTTGACAGACTGCAGGTCCTGGACGATCAYTACCGGGACGTGCTCAAGGAGATGAAGGCGAAGGCGTCCACGGTTAAGGCTAAACTTCTATCCGTAGAAGAAGCCTGCAAGCTGACGCCCCCACRYTCGGCCAAATCCAAATTTGGCTATGGGGCAAAGGACGTCCGGAGCCTRTCCAGCAAGGCCATTAACCACATCAACTCCGTGTGGAAGGACTTGCTGGAAGACACTGAGACACCAATTGACACCACCATCATGGCAAARAAYGAGGTTTTCTGCGTCCAACCAGAGAAAGGAGGCCGCAAGCCAGCTCGCCTTATCGTATTCCCAGACTTGGGGGTTCGTGTGTGCGAGAARATGGCCCTTTATGATGTGGTCTCCACCCTTCCTCAGGCCGTGATGGGCTCCTCATACGGGTTCCAGTACTCTCCTGGACAGCGGGTCGAGTTCCTGGTGAATGCCTGGAAGAAAAARAARAACCCCATGGGTTTCGCATATGACACCCGCTGTTTTGACTCAACGGTCACCGAGAGTGACATCCGTGTTGAGGAGTCAATCTACCANNNNNNNNNNNNNNNNNNNNNNNNNNNNNNNNNNNNNNNNNNNNNNNNNNNNNNNNNNNNNNNNNNNNNNNNNNNNNNNNNNNNNNNNNNNNNNNNNNNNNNNNNNNNNNNNNNNNNNNNNNNNNNNNNNNNNNNNNNNNNNNNNNNNNNNNNNNNNNNNNNNNNNNNNNNNNNNNNNNNNNNNNNNNNNNNNNNNNNNNNNNNNNNNNNNNNNNNNNNNNNNNNNNNNNNNNNNNNNNNNNNNNNNNNNNNNNNNNNNNNNNNNNNNNNNNNNNNNNNNNNNNNNNNNNNNNNNNNNNNNNNNNNNNNNNNNNNNNNNNNNNNNNNNNNNNNNNNNNNNNNNNNNNNNNNNNNNNNNNNNNNNNNNNNNNNNNNNNNNNNNNNNNNNNNNNNNNNNNNNNNNNNNNNNNNNNNNNNNNNNNNNNNNNNNNNNNNNNNNNNNNNNNNNNNNNNNNNNNNNNNNNNNNNNNNNNNNNNNNNNNNNNNNNNNNNNNNNNNNNNNNNNNNNNNNNNNNNNNNNNNNNNNNNNNNNNNNNNNNNNNNNNNNNNNNNNNNNNNNNNNNNNNNNNNNNNNNNNNNNNNNNNNNNNNNNNNNNNNNNNNNNNNNNNNNNNNNNNNNNNNNNNNNNNNNNNNNNNNNNNNNNNNNNNNNNNNNNNNNNNNNNNNNNNNNNNNNNNNNNNNNNNNNNNNNNNNNNNNNNNNNNNNNNNNNNNNNNNNNNNNNNNNNNNNNNNNNNNNNNNNNNNNNNNNNNNNNNNNNNNNNNNNNNNNNNNNNNNNNNNNNNNNNNNNNNNNNNNNNNNNNNNNNNNNNNNNNNNNNNNNNNNNNNNNNNNNNNNNNNNNNNNNNNNNNNNNNNNNNNNNNNNNNNNNNNNNNNNNNNNNNNNNNNNNNNNNNNNNNNNNNNNNNNNNNNNNNNNNNNNNNNNNNNNNNNNNNNNNNNNNNNNNNNNNNNNNNNNNNNNNNNNNNNNNNNNNNNNNNNNNNNNNNNNNNNNNNNNNNNNNNNNNNNNNNNNNNNNNNNNNNNNNNNNNNNNNNNNNNNNNNNNNNNNNN

>HM106886

TCGATGTCCTACACATGGACGGGCGCCCTGATCACGCCATGCTCCGCGGAGGAAAGCAAGCTGCCCATCAACGCGTTGAGCAATTCTCTGCTGCGTCAYCAYAACATGGTCTATGCCACAACATCTCGCAGCGCAAGCCAGCGGCAGAAGAAGGTCACCTTTGACAGACAGCAGGTCCTGGACGACCATTACCGGGACGTGCTCAAGGAGATGAAGGCGAAGGCGTCCACRGTTAAGGCTAAACTCCTATCCGTAGAAGAAGCCTGTATGCTGACGCCCCCACACTCGGCCAGATCCAAATTTGGCTATGGGGCRAAGGACGTCCGGAACCTATCCAGCAAGGCCATTAACCACATCCGCTCCGTGTGGAAGGACTTGCTGGAAGACACTGAGACACCAATTGACACCACCATCATGGCAAAAAATGAGGTTTTCTGTGTCCAACCAGAGAAAGGAGGCCGCAAGCCAGCTCGCCTTATCGTATTCCCAGACTTGGGGGTTCGTGTGTGCGAGAARATGGCCCTTTATGACGTGGTCTCCACYCTTCCTCAGRCCGTGATGGGCCCCTCATACGGGTTYCAGTACTCTCCTGGACAGCGGGTCGAGTTCCTGGTGAATGCCTGGAARAAAAAGAARAACCCTATGGGCTTCGCATATGACACYCGCTGTTTTGACTCAACGGTCACCGAGAGTGACATCCGTGTTGAGGAGTCAATCTACCAATGTTGTGACTTGGCCCCCNNNNNNNNNNNNNNNNNNNNNNNNNNNNNNNNNNNNNNNNNNNNNNNNNNNNNNNNNNNNNNNNNNNNNNNNNNNNNNNNNNNNNNNNNNNNNNNNNNNNNNNNNNNNNNNNNNNNNNNNNNNNNNNNNNNNNNNNNNNNNNNNNNNNNNNNNNNNNNNNNNNNNNNNNNNNNNNNNNNNNNNNNNNNNNNNNNNNNNNNNNNNNNNNNNNNNNNNNNNNNNNNNNNNNNNNNNNNNNNNNNNNNNNNNNNNNNNNNNNNNNNNNNNNNNNNNNNCACGGAGGCTATGACYAGGTACTCTGCCCCCCCCGGGGACCCGCCCAAACCAGAATACGACTTGGAGCTGATAACATCATGCTCCTCCAATGTGTCGGTCGCGCACGATGCATCTGGCAAAAGGGTGTACTACCYCACCCGTGACCCCACCACCCCACTTGCACGGGCTGCGTGGGAGACAGCTAGACACACTCCGGTCAACTCCTGGCTAGGCAACATCATCATGTATGCGCCCACCTTATGGGCAAGGATGATCCTGATGACTCACTTCTTCTCCATCCTTCTAGCTCAGGAACAACTTGAAAAGGCTCTGGATTGTCAGATCTACGGGGCCTGTTACTCCATTGAGCCACTTGACCTACCTCARATCATTCAGCGACTCCATGGTCTTAGCGCATTTTCACTCCATAGTTACTCTCCAGGTGAAATCAATAGGGTGGCTGCATGCCTCAGAAAACTTGGGGTACCACCCTTGCGAGYCTGGAGACATCGGGCCAGAAGTGTCCGCGCTAAGCTCCTGTCCCAGGGGGGGAGGGCTGCCAACTGTGGCAAATACCTCTCCAACTGGGCAGTAAGGACCAAGCNNNNNNNNNNNNNNNNNNNNNNNNNNNNNNNNNNNNNNNNNNNNNNNNNNNNNNNNNNNNNNNNNNNNNNNNNNNNNNNNNNNNNNNNNNNNNNNNNNNNNNNNNNNNNNNNNNNNNNNNNNNNNNNNNNNNNNNNNNNNNNNNNNNNNNNNNNNNNNNNNNNNNNNNNNNNN

>HM106887

TCGATGTCCTACACATGGACGGGCGCCCTGATCACGCCATGCTCCGCGGAGGAAAGCAAGCTGCCCATCAATGCGTTGAGCAACTCTTTGCTGCGTCACCACAACATGGTCTATGCCACAACATCTCGCAGCGCAAGCCAGCGGCAGAAGAAGGTCACCTTTGACAGACTGCAGGTCCTGGACGACCATTACCGGGACGTGCTCAAGGAGATGAAGGCGAAGGCGTCCACGGTTAAGGCTAAACTTCTATCCGTAGAAGAAGCCTGCARGCTGACGCCCCCACATTCGGCCAAATCCAAATTTGGCTATGGGGCAAAGGACGTCCGGAACCTGTCCAGCAAGGCCGTTAACCACATCCACTCCGTGTGGAAGGACTTGCTGGAAGACACTGAGACACCAATTGACACCACCATCATGGCAAAAAATGAGGTTTTCTGCATCCAACCAGAGAAAGGAGGCCGCAAGCCAGCTCGCCTTATCGTATTCCCAGATTTGGGGGTTCGCGTGTGCGAGAAAATGGCCCTTTATGACGTGGTCTCCACCCTTCCTCAGGCCGTGATGGGCTCCTCATACGGGTTCCAGTACTCTCCTGGGCAGCGGGTCGAGTTCCTGGTGAATGCCTGGAAAAAAAAGAAAAACCCTATGGGCTTCGCATATGACACCCGCTGTTTTGACTCAACGGTCACCGAGAGTGACATCCGTGTTGAGGAGTCAATCTACCAATGTTGTGACTTGGCCCCCGAAGCCAGACAGGCCATAAGGTCGCTCACAGAGCGGCTTTATATCGGGGGTCCCCTGACTAACTCAAAAGGGCAGAACTGCGGTTATCGCCGGTGCCGCGCGAGCGGTGTGCTGACGACTAGCTGCGGTAATACCCTCACATGCTACTTGAAGGCCTCTGCRGCCTGTCGAGCTGCCAAGCTCCAGGACTGCACGATGCTCGTGTGCGGAGACGACCTTGTCGTTATCTGTGAAAGTGCGGGAGTCCAGGAGGACGCGGCGAGCCTACGAGTCTTCACGGAGGCTATGACTAGGTACTCCGCCCCCCCCGGAGACCCGCCCAAACCAGAGTACGACTTGGAGTTGATAACATCATGCTCCTCCAATGTGTCGGTCGCGCACGATGCATCTGGCAAAAGGGTGTACTACCTCACCCGTGACCCCACCACCCCACTTGCACGGGCTGCGTGGGAGACAGCTAGACACACTCCAGTTAACTCCTGGCTAGGCAACATCATCATGTACGCGCCCACCTTATGGGCAAGGATGATCCTGATGACTCACTTCTTCTCCATCCTCCTAGCTCAGGAACAACTTGAAAAGGCTCTAGATTGTCAGATCTACGGGGCCTGTTACTCCATTCAGCCACTTGACCTACCTCAGATCATTCAGCGACTCCATGGNNNNNNNNNNNNNNNNNNNNNNNNNNNNNNNNNNNNNNNNNNNNNNNNNNNNNNNNNNNNNNNNNNNNNNNNNNNNNNNNNNNNNNNNNNNNNNNNNNNNNNNNNNNNNNNNNNNNNNNNNNNNNNNNNNNNNNNNNNNNNNNNNNNNNNNNNNNNNNNNNNNNNNNNNNNNNNNNNNNNNNNNNNNNNNNNNNNNNNNNNNNNNNNNNNNNNNNNNNNNNNNNNNNNNNNNNNNNNNNNNNNNNNNNNNNNNNNNNNNNNNNNNNNNNNNNNNNNNNNNNNNNNNNNNNNNNNNNNNNNNNNNNNNNNNNNNNNNNNNNNNNNNNNNNNNNNNNNNNNNNNNNNNNNNNNNNNNNNNNNNNNNNNNNNN

>HM106888

TCGATGTCCTACACATGGACAGGCGCCCTGATCACGCCATGCTCCGCGGAGGAAAGCAAGCTGCCCATTAACGCGTTGAGCAAYTCTTTGCTGCGCCACCACAACATGGTCTATGCCACAACATCTCGCAGCGCAAGCCAGCGGCAGAAGAAGGTCACCTTTGACAGACTGCAGGTCCTGGACGACCAYTACCGGGACGTGCTCAAGGAGATGAAGGCGAAGGCGTCCACAGTTAAGGCTAAACTTCTATCCGTAGAGGAAGCCTGCATGCTGACGCCCCCACACTCGGCCAAATCCAAATTTGGCTATGGGGCAAAGGACGTCCGGAGCCTATCCAGCAAGGCCATTAACCACATCCGCTCCGTKTGGAAGGACTTGCTGGAAGACACTGAAACACCAATTGACACCACCATCATGGCAAAAAATGAGGTTTTCTGCGTCCAACCAGAGAAAGGAGGCCGCAAGCCAGCTCGCCTTATCGTATTCCCAGACTTGGGGGTTCGTGTGTGCGAGAAAATGGCCCTTTATGACGTGGTCTCCACCCTTCCTCAGGCCGTGATGGGTTCCTCATACGGGTTCCAGTACTCTCCTGGACAGCGGGTCGAGTTCCTGGTGAATGCCTGGAAAAAAAAGAAGAACCCTATGGGCTTCGCATATGACACCCGCTGTTTTGACTCAACGGTCACCGAGAGTGAYATCCGTGTTGAGGAATCAATCTACCAATGTTGTGACTTGGCCCCCGAAGCCAGACAGGCCATAAGGTCGCTCACAGAGCGGCTTTATATCGGGGGTCCCCTGACTAACTCAAAAGGGCAGAACTGCGGTTATCGCCGGTGCCGCGCGAGCGGTGTGCTGACGACCAGCTGCGGTAATACCCTCACATGTTACTTGAAGGCCTCTGCAGCCTGTCGAGCTGCAAAGCTCCAGGACTGCACGATGCTCGTGTGCGGAGACGACCTYGTCGTTATCTGTGAAAGCGCGGGAACCCAGGAGGACGCGGCGAGCCTACGAGTCTTCACGGAGGCTATGACTAGGTACTCTGCCCCCCCCGGGGACCCGCCCAAACCAGAATACGACTTGGAGTTGATAACATCATGCTCCTCCAATGTGTCGGTCGCGCACGATGCGTCTGGCAAAAGGGTGTACTACCTCACCCGTGACCCCACCACCCCACTTGCACGGGCTGCGTGGGAGACAGCTAGACACACTCCAGTCAACTCCTGGCTAGGCAACATCATYATGTATGCGCCCACCTTRTGGGCAAGGATGATCCTGATGACTCACTTCTTTTCCATCCTTCTAGCCCAGGAGCAACTTGAAAAGGCTCTAGATTGCCAGATCTACGGAGCCTGTTACTCCATTGARCCACTTGACCTACCTNNNNNNNNNNNNNNNNNNNNNNNNNNNNNNNNNNNNNNNNNNNNNNNNNNNNNNNNNNNNNNNNNNNNNNNNNNNNNNNNNNNNNNNNNNNNNNNNNNNNNNNNNNNNNNNNNNNNNNNNNNNNNNNNNNNNNNNNNNNNNNNNNNNNNNNNNNNNNNNNNNNNNNNNNNNNNNNNNNNNNNNNNNNNNNNNNNNNNNNNNNNNNNNNNNNNNNNNNNNNNNNNNNNNNNNNNNNNNNNNNNNNNNNNNNNNNNNNNNNNNNNNNNNNNNNNNNNNNNNNNNNNNNNNNNNNNNNNNNNNNNNNNNNNNNNNNNNNNNNNNNNNNNNNNNNNNNNNNNNNNNNNNNNNNNNNNNNNNNNNNNNNNNNNNNNNNNNNNNNNNNNNNNNNNNNNN

>HM106889

NNNATGTCCTACACATGGACGGGCGCCCTGATCACGCCATGCTCCGCGGAGGAAAGCAAGCTGCCCATCAACGCGTTGAGCAACTCTTTGCTGCGTCACCACAACATGGTTTATGCCACAACATCTCGCAGCGCAAGCCAGCGGCAGAAGAAGGTTACCTTTGACAGACTGCAGGTCCTGGACGACCATTACCGGGACGTGCTCAAGGAGATGAAGGCGAAGGCGTCCACGGTTAAGGCTAAACTTCTATCCGTAGAAGAAGCCTGCAAGCTGACGCCCCCACACTCGGCCAAATCCAAATTYGGCTATGGGGCAAAGGACGTCCGGAACCTATCCAGCAAGGCCATTAACCACATCCACTCCGTGTGGAAGGACTTGCTGGAAGACACTGAGACACCAATTGACACCACCATCATGGCGAAAAATGAGGTTTTCTGCGTCCAGCCAGAGAAAGGAGGCCGCAAGCCAGCTCGCCTTATCGTATTCCCAGACTTGGGGGTTCGTGTGTGCGAAAAAATGGCCCTTTATGACGTGGTCTCCACCCTTCCTCAGGCCGTGATGGGCTCCTCATACGGGTTCCAGTAYTCTCCTGGACAGCGGGTCGAGTTCCTGGTGAATGCCTGGAAGAAAAAGAAAAACCCTATGGGTTTCGCATATGACACCCGCTGTTTTGACTCAACGGTCACCGAGAGTGACATCCGTGTTGAGGAGTCAATCTACCAATGTTGTGACTTGGCCCCAGAAGCCAGACAGGCCATAAGGTCGCTCACGGAGCGGCTTTATATCGGGGGTCCCCTGACCAACTCAAAAGGGCAGAACTGCGGCTATCGCCGGTGCCGCGCGAGCGGTGTGTTGACGACTAGCTGCGGTAATACCCTCACATGTTACTTGAAGGCCTCTGCAGCCTGTCGAGCYGCCAAGCTCCAGGACTGCACGATGCTCGTGTGCGGGGACGACCTTGTCGTTATCTGTGAAAGCGCGGGAACCCAGGAGGACGCGGCGAGCCTACGAGTCTTCACGGAGGCTATGACTAGGTACTCTGCCCCCCCCGGGGACCCGCCCAAACCAGAATACGACTTGGAGTTGATAACATCATGCTCCTCCAATGTGTCGGTCGCGCACGATGCATCTGGCAAAAGGGTGTACTACCTCACCCGTGACCCCACCACCCCGCTTGCACGGGCTGCGTGGGAGACYGCTAGACACACTCCAGTCAACTCCTGGCTAGGCAACATCATCATGTATGCGCCCACCTTATGGGCAAGGATGATCCTGATGACCCACTTCTTCTCCATCCTTCTAGCYCAGGAACAACTTGAAAAGGCTCTAGAGTGTCAGATCTACGGGGCCTGTTACTCCATTGAGCCACTTGACCTACCTCAGATCATTCAGCGACTCCATGGNNNNNNNNNNNNNNNNNNNNNNNNNNNNNNNNNNNNNNNNNNNNNNNNNNNNNNNNNNNNNNNNNNNNNNNNNNNNNNNNNNNNNNNNNNNNNNNNNNNNNNNNNNNNNNNNNNNNNNNNNNNNNNNNNNNNNNNNNNNNNNNNNNNNNNNNNNNNNNNNNNNNNNNNNNNNNNNNNNNNNNNNNNNNNNNNNNNNNNNNNNNNNNNNNNNNNNNNNNNNNNNNNNNNNNNNNNNNNNNNNNNNNNNNNNNNNNNNNNNNNNNNNNNNNNNNNNNNNNNNNNNNNNNNNNNNNNNNNNNNNNNNNNNNNNNNNNNNNNNNNNNNNNNNNNNNNNNNNNNNNNNNNNNNNNNNNNNNNNNNNNNNNNNNNN

>HM106890

TCGATGTCCTACACATGGACAGGCGCCCTGATTACGCCATGCTCCGCGGAGGAAAGCAAGCTGCCCATCAACGCGTTGAGCAACTCCTTGCTGCGTCACCACAACATGGTCTATGCCACAACATCACGCAGCGCAAGCCAGCGGCAGAAAAAGGTCACCTTTGACAGACTGCAGGTCCTGGACRACCATTACCGGGACGTGCTCAAGGAGATGAAGGCGAAGGCGTCCACGGTTAAGGCTAAACTTTTATCCGTAGAAGAAGCCTGCAAACTGACGCCCCCACACTCGGCCAGATCCAAGTTTGGCTACGGGGCGAAGGACGTCCGGAACCTATCCAGCAAGGCCATTAACCACATCCGCTCCGTGTGGCAGGACTTGCTGGAAGACACTGAGACACCAATTGACACCACCATCATGGCAAAAAATGAGGTTTTCTGCATCCAACCAGAGAAAGGAGGCCGCAAGCCAGCTCGCCTCATCGTGTTCCCAGACTTGGGGGTTCGTGTGTGCGAGAAAATGGCCCTTTACGACGTGGTCTCCACCCTTCCTCAGGCCGTGATGGGCTCCTCATACGGGTTCCAGTACTCTCCTGGACAGCGGGTCGAGTTCCTGGTGAATGCCTGGAAAAAAAAGAAAAACCCTATGGGCTTCGCATATGACACCCGCTGTTTTGACTCAACGGTCACCGAGAGTGACATCCGTGTTGAGGAGTCAATCTACCAATGTTGTGACTTGGCTCCCGAAGCCAGACAGGCCATAAGGTCGCTCACAGAGCGGCTTTATATCGGGGGTCCCCTGACCAACTCAAAAGGGCAGAACTGCGGTTATCGCCGGTGCCGCGCGAGCGGTGTGCTGACAACTAGCTGCGGYAATACCCTCACATGTTACTTGAAGGCCACTGCAGCCTGTCGAGCTGCTAAGCTCCGGGACTGCGAGATGCTCGTGTGCGGAGACGACCTTGTCGTTATCTGCGAAAGCGCGGGAACCCAGGAGGACGCGGCGAGCCTACGAGTCTTCACGGAGGCTATGACTAGGTACTCTGCCCCCCCCGGGGACCCGCCCAAACCAGAATACGACTTGGAGTTAATAACATCATGCTCCTCCAACGTGTCGGTCGCGCACGATGCGTCTGGCAAAAGGGTGTACTACCTCACCCGTGATCCCACCACCCCACTTGCACGGGCTGCGTGGGAGACAGCTAGACACACTCCAGTCAACTCCTGGCTAGGCAACATCATCATGTATGCGCCCACCTTATGGGCAAGGATGATCCTGATGACTCACTTCTTCTCCATCCTTCTAGCTCAGGAACAACTTGAAAAGGCTCTAGATTGTCAGATCTACGGGGCCTGCTACTCCATTGAGCCACTTGACCTACCTCAGATCATTCAGCGACTCCATGGNNNNNNNNNNNNNNNNNNNNNNNNNNNNNNNNNNNNNNNNNNNNNNNNNNNNNNNNNNNNNNNNNNNNNNNNNNNNNNNNNNNNNNNNNNNNNNNNNNNNNNNNNNNNNNNNNNNNNNNNNNNNNNNNNNNNNNNNNNNNNNNNNNNNNNNNNNNNNNNNNNNNNNNNNNNNNNNNNNNNNNNNNNNNNNNNNNNNNNNNNNNNNNNNNNNNNNNNNNNNNNNNNNNNNNNNNNNNNNNNNNNNNNNNNNNNNNNNNNNNNNNNNNNNNNNNNNNNNNNNNNNNNNNNNNNNNNNNNNNNNNNNNNNNNNNNNNNNNNNNNNNNNNNNNNNNNNNNNNNNNNNNNNNNNNNNNNNNNNNNNNNNNNNNNN

>HM106891

NNNATGTCCTACACATGGACRGGCGCCCTRATCACGCCATGCTCCGCGGAGGAAAGCAAGCTGCCYATCAACGCGYTGAGCAAYTCYTTGCTGCGTCACCACAACATGGTYTATGCCACAACATCTCGCAGCGCAAGCCAGCGGCAGAAAAAGGTCACYTTTGACAGACTGCARGTCCTGGACGACCATTACCGGGACGTGCTYAAGGAGATGAAGGCGAAGGCGTCCACAGTTAAGGCTAAACTTCTATCCGTAGAAGAAGCCTGCATGCTGACGCCCCCACACTCGGCCAAATCCAAATTTGGCTATGGGGCAAAGGACGTCCGGAACCTATCCAGCAAGGCCRYTAACCACATCCRCTCCGTGTGGAAGGACTTRCTGGAAGACACTGAGACACCAATTGACACCACCATCATGGCAAARAATGAGGTTTTCTGCGTYCAACCAGAGAAAGGAGGCCGCAAGCCAGCTCGCCTTATCGTATTCCCAGACTTGGGGGTTCGTGTGTGCGAGAAAATGGCCCTTTATGACGTGGTCTCCACCCTTCCTCAGGCCGTGATGGGCTCCTCATACGGGTTCCAGTACTCTCCTGGACAGCGGGTCGAGTTCCTGGTGAAYGCCTGGAAAAAAAAGAARAAYCCYATGGGCTTCGCATATGACACCCGCTGTTTTGACTCAACGGTCACCGAGAGTGACATCCGTGTTGAGGAGTCAATCTAYCAATGYTGTGACTTGGCCCCCGAAGCCAGACAGGCCATAAGGTCGCTCACAGAGCGGCTTTATATCGGGGGTCCCCTGACTAACTCAAAAGGGCAGAACTGCGGYTATCGCCGGTGCCGCGCRAGCGGTGTRCTGACGACTAGCTGCGGTAATACCCTCACATGTTACTTGAAGGCCTCTGCAGCCTGTCGAGCTGCCAAGCTCCAGGACTGCACGATGCTCGTGTGCGGAGACGACCTTGTCGTTATCTGTGAAAGCGCGGGAACCCAGGAGGAYGCGGCGAGCCTACGAGTCTTCACGGAGGCTATGACTAGGTACTCTGCCCCCCCCGGGGACCCGCCCAAACCAGAATACGACTTGGAGTTGATAACATCATGCTCCTCCAAYGTGTCRGTCGCGCACGAYGCATCTGGCAAAAGGGTGTACTACCTCACCCGTGACCCCACCACCCCRCTTGCACGGGCTGCGTGGGAGACAGCTAGACACACTCCAGTCAACTCCTGGCTAGGCAACATCATCATGTATGCGCCCACCTTATGGGCAAGGATGATCCTGATGACTCACTTTTTCTCCATCCTTCTAGCTCAGGAACAACTTGAAAAGGCTCTRGATTGTCAGATCTACGGGGCCTGTTACTCCATTGAGCCACTTGACCTACCTCAGATCATTCAACGACTCCATGGNNNNNNNNNNNNNNNNNNNNNNNNNNNNNNNNNNNNNNNNNNNNNNNNNNNNNNNNNNNNNNNNNNNNNNNNNNNNNNNNNNNNNNNNNNNNNNNNNNNNNNNNNNNNNNNNNNNNNNNNNNNNNNNNNNNNNNNNNNNNNNNNNNNNNNNNNNNNNNNNNNNNNNNNNNNNNNNNNNNNNNNNNNNNNNNNNNNNNNNNNNNNNNNNNNNNNNNNNNNNNNNNNNNNNNNNNNNNNNNNNNNNNNNNNNNNNNNNNNNNNNNNNNNNNNNNNNNNNNNNNNNNNNNNNNNNNNNNNNNNNNNNNNNNNNNNNNNNNNNNNNNNNNNNNNNNNNNNNNNNNNNNNNNNNNNNNNNNNNNNNNNNNNNNNN

>HM106892

TCGATGTCCTACACATGGACAGGCGCCCTGATCACGCCRTGCGCCGCGGAGGAAAGCAAGCTGCCCATCAACGCGYTGAGCAACTCTTTGCTGCGTCACCACAACATGGTGTATGCCACAACATCTCGCAGCGCAACCCAGCGGCAGAAGAAGGTCACCTTTGACAGACTGCAGGTCCTGGACGAYCATTACCGGGACGTGCTCAAGGAGATGAAGGCGAAGGCGTCCACAGTTAAGGCTAAACTTCTATCCGTAGAAGAAGCCTGTAAGCTGACGCCCCCACAYTCGGCCAGATCCAARTTTGGCTATGGGGCAAAGGACGTCCGGAACCTATCCAGCAAGGCCGTCAACCACATCCGCTCCGTGTGGAAGGACTTGCTGGAAGACRCTGAGACACCAATTGACACCACCATCATGGCAAARAATGAGGTTTTCTGCGTCCAACCAGAGAAAGGAGGCCGCAAGCCAGCCCGCCTTATCGTATTCCCAGACTTGGGGGTTCGTGTGTGCGAGAAAATGGCCCTTTATGACGTGGTCTCCACCCTTCCTCAGGCCGTGATGGRCTCCTCATACGGGTTTCAGTACTCTCCTGGACAGCGGGTCGAGTTCCTGGTGAATGCCTGGAAAAAAAAGAAAAACCCCATGGGCTTCGCATATGACACCCGCTGTTTTGACTCAACGGTCACCGAGAGTGATATCCGTGTTGAGGAGTCAATCTACCAATGTTGTGACTTGGCCCCCGAAGCCAGACAGGCCATAAAGTCGCTCACAGAGCGGCTTTACATCGGGGGTCCCCTGACTAACTCAAAAGGGCAGAACTGCGGTTATCGCCGGTGCCGCGCGAGCGGCGTGCTGACGACCAGCTGCGGTAATACCCTCACATGTTACTTGAAGGCCTCTGCAGCCTGTCGAGCTGCCAAGCTCCAGGACTGCACGATGCTCGTGTGCGGAGACGACCTTGTCGTTATCTGTGAGAGCGCGGGAACCCAGGAGGACGCGGCGAGCCTACGAGTCTTCACGGAGGCTATGACTAGGTACTCTGCCCCCCCCGGGGACCCGCCCAAACCAGAGTACGACTTGGAGTTGATAACATCATGCTCCTCCAATGTATCGGTCGCGCACGATGCATCTGGCAAAAGGGTGTACTACCTCACCCGYGACCCCACCACCCCGCTTGCACGGGCTGCGTGGGAGACAGCAAGACACACTCCAGTCAATTCCTGGCTAGGCAACATCATCATGTATGCGCCCACCTTATGGGCAAGGATGATTCTGATGACTCACTTCTTCTCCATCCTTCTAGCTCAGGAACAACTTGAAAAGGCCCTGGATTGTCAGATCTACGGGGCCTGCTACTCCATTGAGCCACTTGACCTACCTCAAATCATTCAGCGACTCCATGGNNNNNNNNNNNNNNNNNNNNNNNNNNNNNNNNNNNNNNNNNNNNNNNNNNNNNNNNNNNNNNNNNNNNNNNNNNNNNNNNNNNNNNNNNNNNNNNNNNNNNNNNNNNNNNNNNNNNNNNNNNNNNNNNNNNNNNNNNNNNNNNNNNNNNNNNNNNNNNNNNNNNNNNNNNNNNNNNNNNNNNNNNNNNNNNNNNNNNNNNNNNNNNNNNNNNNNNNNNNNNNNNNNNNNNNNNNNNNNNNNNNNNNNNNNNNNNNNNNNNNNNNNNNNNNNNNNNNNNNNNNNNNNNNNNNNNNNNNNNNNNNNNNNNNNNNNNNNNNNNNNNNNNNNNNNNNNNNNNNNNNNNNNNNNNNNNNNNNNNNNNNNNNNNNN

>HM106893

NNNNTGTCCTACACATGGACAGGCGCCCTRATCACGCCATGCKCCGCGGAGGARAGCAMGCTGCCCATCAACGCGTTGAGCAACTCTTTGCTGCGTCACCACAACATGGTYTATGCCACAACATCTCGCAGCGCAAGCCAGCGGCAGAAGAAGGTCACCTTTGACAGACTGCARGTCCTGGACGACCATTACCGGGACGTGCTCAAGGAGATGAAGGCGAAGGCGTCCACRGTTAAGGCTAAACTTCTATCCGTAGAAGAAGCCTGYATGCTGACGCCCCCACACTCGGCCAGATCCAAATTTGGCTATGGGGCAAAGGACGTCCGRARCCTATCCAGCAAGGCCACTAACCACATCCGCTCCGTGTGGAAGGACTTGCTGGAAGACACTGAGACACCAATTGACACCACCATCATGGCAAAAAATGAGGTTTTCTGCGTCCAACCAGAGAAAGGAGGCCGCAAGCCAGCTCGCCTTATCGTATTCCCAGACTTGGGGGTTCGTGTGTGCGAGAAAATGGSCCTTTATGACGTGGTCTCCACCCTTCCTCAGGCCGTGATGGGCTCCTCATACGGGTTCCAGTACTCTCCTGGACAGCGGGTCGAGTTCCTGGTGAATGCCTGGAARAARAAGAARAACCCTATGGGCTTCKCATATGACACCCGCTGTTTTGACTCAACGGTCACCGAGAGTGACATCCGTGTTGAGGAGTCAATCTACCAATGTTGTGACTTGGCCCCCGAAGCCAGACAGGCCATAAGGTCGCTCACRGAGCGGCTTTATATCGGGGGTCCYCTGACTAAYTCAAAAGGGCAGAACTGCGGTTAYCGCCGGTGCCGCGCGAGCGGTGTGCTGACGACYAGCTGCGGTAATACCCTCACATGTTACTTGAAGGCCTCTGCAGCCTGTCGAGCTGCCAAGCTCCAGGACTGCACGATGCTCGTGTGCGGAGACGACCTTGTCGTTATCTGTGARAGCGCGGGAACCCAGGAGGACGCGGCGAGCCTACGAGTCTTCACGGAGGCTATGACTAGGTACTCTGCCCCCCCCGGGGACCCGCCCAAACCAGAATACGACTTGGAGTTGATAACATCATGCTCCTCCAAYGTGTCGGTCGCGCACGATGCATCYGGCAAAAGGGTGTACTACCTCACCCGTGACCCCACCACCCCGCTTGCACGGGCTGCGTGGGAGACAGCTAGACACACTCCAGTCAAYTCCTGGCTAGGCAACATCATCATGTATGCGCCCACCTTATGGGCAAGGATGATCCTGATGACTCACTTCTTCTCCATCCTTCTAGCTCARGAACAACTTGAAAAGGCTCTAGATTGTCMAATCTACGGGGCCTGTTACTCCATTGAGCCACTTGACCTNNNNNNNNNNNNNNNNNNNNNNNNNNNNNNNNNNNNNNNNNNNNNNNNNNNNNNNNNNNNNNNNNNNNNNNNNNNNNNNNNNNNNNNNNNNNNNNNNNNNNNNNNNNNNNNNNNNNNNNNNNNNNNNNNNNNNNNNNNNNNNNNNNNNNNNNNNNNNNNNNNNNNNNNNNNNNNNNNNNNNNNNNNNNNNNNNNNNNNNNNNNNNNNNNNNNNNNNNNNNNNNNNNNNNNNNNNNNNNNNNNNNNNNNNNNNNNNNNNNNNNNNNNNNNNNNNNNNNNNNNNNNNNNNNNNNNNNNNNNNNNNNNNNNNNNNNNNNNNNNNNNNNNNNNNNNNNNNNNNNNNNNNNNNNNNNNNNNNNNNNNNNNNNNNNNNNNNNNNNNNNNNNNNNNNNNNNNNN

>HM106894

NNNNNNNNNNNNNNNNNNNNNNNNNNNNNGATCACGCCATGTTCCGCGGAGGAAAGCAAGCTGCCTATCAACGCGCTGAGCAACTCTTTGCTGCGTCACCACAACATGGTCTATGCCACGACATCTCGCAGCGCAAGCCAGCGGCAGAAAAAGGTCACCTTTGACAGACTGCAGGTCCTGGACGACCATTACCGGGACGTGCTCAAGGAGATGAAGGCGAAGGCGTCCACGGTTAAGGCTAAACTTCTATCCRTAGAAGAAGCCTGCATGCTGACGCCCCCACACTCGGCCAAATCAAAATTTGGCTATGGGGCAAAGGACGTCCGGAACCTATCCCGCAAGGCCACTGACCACATCCGCTCCGTGTGGAAGGACTTGCTGGAAGACACTGAGACACCAATTGACACCACCATCATGGCAAAAAATGAGGTTTTCTGCGTCCAACCAGAGAAAGGAGGCCGCAAGCCAGCTCGCCTTATCGTGTTCCCAGACTTGGGGGTTCGTGTGTGCGAGAAAATGGCCCTTTATGACGTGGTCTCCACCCTTCCTCAGGCCGTGATGGGCTCCTCATACGGTTTCCAGTACTCTCCTGGACAGCGGGTCGAGTTCCTGGTGAATGCCTGGANNNNNNNNNNNNNNNNNNNNNNNNNNNNNNNNNNNNNNNNNNGTTTTGACTCAACRGTCACTGAGAGTGACATCCGTGTTGAGGAGTCAATCTACCAATGTTGTGACTTGGCCCCCGAAGCCAGACAGGCCATAAGGTCGCTCACAGAGCGGCTTTATATCGGGGGCCCCCTGACTAACTCAAAAGGGCAGAACTGCGGCTATCGCCGGTGCCGCGCGAGCGGTGTGCTGACGACCAGCTGCGGTAATACCCTTACATGTTACTTGAAGGCTTCTGCAGCCTGTCGAGCTGCCAAGCTCCAGGACTGCACAATGCTCGTGTGCGGAGACGACCTTGTCGTTATCTGTGARAGCGCGGGAACCCAGGAGGACGCGGCGAGCCTACGAGTCTTCACGGAGGCYATGACTAGGTACTCTGCCCCCCCYGGGGACCCGCCCAAACCAGAATACGACTTGGAGYTGATAACATCATGCTCCTCCAATGTGTCGGTCGCGCACGATGCATCTGGCAAAAGGGTGTACTACCTCACCCGTGACCCCACCACCCCACTTGCACGGGCTGCGTGGGAGACAGCTAGACACACTCCAGTCAACTCCTGGCTAGGCAACATCATTATGTAYGCGCCCACCCTATGGGCAAGGATGATCCTGATGACTCACTTCTTCTCCATCCTTCTAGCTCAGGAACAACTTGAAAAGGCTCTAGATTGTCAGATCTACGGGGCCTGTTACTCCATTGAGCCACTTGACCTACCTCAGATCATTCAGMGACTCCATGGNNNNNNNNNNNNNNNNNNNNNNNNNNNNNNNNNNNNNNNNNNNNNNNNNNNNNNNNNNNNNNNNNNNNNNNNNNNNNNNNNNNNNNNNNNNNNNNNNNNNNNNNNNNNNNNNNNNNNNNNNNNNNNNNNNNNNNNNNNNNNNNNNNNNNNNNNNNNNNNNNNNNNNNNNNNNNNNNNNNNNNNNNNNNNNNNNNNNNNNNNNNNNNNNNNNNNNNNNNNNNNNNNNNNNNNNNNNNNNNNNNNNNNNNNNNNNNNNNNNNNNNNNNNNNNNNNNNNNNNNNNNNNNNNNNNNNNNNNNNNNNNNNNNNNNNNNNNNNNNNNNNNNNNNNNNNNNNNNNNNNNNNNNNNNNNNNNNNNNNNNNNNNNNNNN

>HM106895

NNNATGTCCTACACGTGGACAGGCGCCCTGATTACGCCATGCTCCGCGGAGGARAGCAAGCTGCCCATCAACGCGTTGRGCAACTCTTTGCTGCGTCACCACAACATGGTCTATGCCACAACATCTCGCAGCGCAAGCCAGCGGCAGAAGAAGGTCACTTTTGACAGACTGCAGGTCCTGGACGACCATTACCGGGACGTGCTTAAGGAGATGAAGGCGAAGGCGTCCACAGTTAAGGCTAAACTTCTATCCGTAGAGGAAGCCTGCATGCTGACGCCCCCACACTCGGCCAAATCCAAATTTGGCTATGGGGCAAAGGACGTCCGGAACCTATCCAGCAAGGCCATTAACCACATCCGCTCCGTGTGGAAGGACTTGCTGGAAGACTCTGAGACACCAATTGACACCACCATCATGGCAAAAAATGAGGTTTTCTGCGTCCAACCAGAGAAAGGAGGTCGCAAGCCAGCTCGCCTTATCGTATTCCCAGACTTGGGGGTTCGTGTGTGCGAGAAAATGGCTCTTTATGATGTGGTCTCCACCCTTCCTCAGGCCGTGATGGGCTCCTCATACGGGTTCCAGTACTCTCCTGGACAGCGGGTCGAGTTCCTGGTGAATGCTTGGAAAAAAAAGAAGAACCCCATGGGCTTCGCATATGACACCCGCTGTTTTGACTCAACGGTCACTGAGAGCGACATCCGTGTTGAGGAGTCAATTTACCAATGCTGTGACTTGGCCCCCGAAGCCAGACAGGCCATAAGGTCGCTCACAGAGCGGCTTTATATCGGGGGTCCCCTGACTAACTCAAAAGGGCAGAACTGCGGTTATCGCCGGTGCCGCGCGAGCGGTGTGCTGACGACTAGCTGCGGTAATACCCTCACATGTTACTTGAAGGCCTCTGCAGCCTGTCGAGCTGCCAAGCTCCAGGACTGCACGATGCTCGTGTGCGGAGACGACCTTGTCGTTATCTGTGAAAGCGCGGGAACCCAGGAGGACGCGGCGAGCCTACGAGTCTTCACGGAGGCTATGACTAGGTACTCTGCCCCCCCCGGGGACCCGCCCAAACCAGAATACGACTTGGAGTTGATAACATCATGCTCCTCCAATGTGTCGGTCGCGCACGATGCATCTGGCAAAAGGGTGTACTACCTCACCCGCGATCCCACCACCCCACTTGCACGGGCTGCGTGGGAGACAGCTAGACACACTCCAGTCAACTCCTGGCTAGGCAACATCATCATGTATGCGCCCACCTTATGGGCAAGGATGATCCTGATGACTCACTTCTTCTCCATCCTTCTAGCTCAGGAACAACTTGAAAAGGCTCTAGATTGTCAGATCTACGGGGCCTGTTACTCCATTGAGCCACTTGACCTACCTCAAATCATTCAGCGACTCCATGGNNNNNNNNNNNNNNNNNNNNNNNNNNNNNNNNNNNNNNNNNNNNNNNNNNNNNNNNNNNNNNNNNNNNNNNNNNNNNNNNNNNNNNNNNNNNNNNNNNNNNNNNNNNNNNNNNNNNNNNNNNNNNNNNNNNNNNNNNNNNNNNNNNNNNNNNNNNNNNNNNNNNNNNNNNNNNNNNNNNNNNNNNNNNNNNNNNNNNNNNNNNNNNNNNNNNNNNNNNNNNNNNNNNNNNNNNNNNNNNNNNNNNNNNNNNNNNNNNNNNNNNNNNNNNNNNNNNNNNNNNNNNNNNNNNNNNNNNNNNNNNNNNNNNNNNNNNNNNNNNNNNNNNNNNNNNNNNNNNNNNNNNNNNNNNNNNNNNNNNNNNNNNNNNNN

>HM106896

NNNNTGTCCTACACATGGACRGGCGCCCTGATCACGCCATGCTCCGCGGAGGAAAGCAAGCTGCCCATCAACGCGYTGAGCAACTCTYTGCTGCGTCACCAYAACATGGTCTATGCYACAACATCTCGCAGCGCAAGCCAGCGGCAGAARAAAGTYACYTTTGACAGACTGCARGTCCTGGACGACCAYTACCGGGACGTGCTCAAGGAGATGAAGGCGAAGGCGTCCACRGTTAAGGCTAAACTTCTATCCRTAGARGAAGCCTGCATGCTGACGCCCCCACACTCGGCCAAATCCAAATTTGGCTATGGGGCAAARGACGTCCGGAACCTATCCAGCAAGGCCAYTAACCACATCCGCTCCGTGTGGAAGGACTTGCTGGAAGACACTGAGACACCAATTGACACCACCATCATGGCAAARAATGAGGTTTTCTGCGTCCAACCAGAGAAAGGRGGCCGCAAGCCAGCTCGCCTTATCGTATTCCCAGACTTGGGGGTTCGTGTGTGCGAGAAAATGGCCCTTTATGACGTGGTCTCCACCCTTCCTCAGGCCGTGATGGGCTCCTCATACGGGTTCCAGTACTCTCCTGGACAGCGGGTCGAGTTCCTGGTGAATGCCTGGAAAAAAAAGAAGAACCCYATGGGCTTCGCATATGACACCCGCTGTTTTGACTCAACGGTCACCGAGAGTGACATCCGTGTTGAGGAGTCAATCTAYCAATGTTGTGACTTGGCYCCCGAAGCCAGACAGGCCATAAGGTCGCTCACAGAGCGGCTTTAYATCGGGGGTCCCCTGACTAACTCAAAAGGGCAGAACTGCGGTTATCGCCGGTGCCGCGCKAGCGGTGTGCTGACGACCAGCTGCGGTAATACCCTCACATGTTACTTGAAGGCCTCTGCAGCCTGTCGAGCTGCCAAGCTCCAGGACTGCACRATGCTCGTGTGCGGAGACGACCTTGTCGTTATCTGTGARAGCGCGGGAACCCAGGAGGACGCGGCGAGCCTACGAGTCTTCACGGAGGCTATGACTAGGTACTCTGCCCCCCCCGGGGACCCGCCCAAACCAGAATACGACTTGGAGTTGATAACATCATGCTCCTCCAAYGTGTCGGTYGCGCACGATGCATCWGGCAAAAGGGTGTAYTACCTCACCCGTGACCCCACCACCCCACTTGCACGGGCTGCGTGGGAGACAGCTAGACACACTCCAGTCAACTCCTGGCTAGGCAACATCATCATGTATGCGCCCACCTTRTGGGCAAGGATGATCCTGATGACTCACTTCTTCTCCATCCTTCTAGCYCAGGAACAACTTGAAAAGGCTCTAGATTGTCAGATCTAYGGGGCCTGTTACTCCATTGAGCCACTTGACCTACCTCAGATCATTCAGCGACTCCATGGNNNNNNNNNNNNNNNNNNNNNNNNNNNNNNNNNNNNNNNNNNNNNNNNNNNNNNNNNNNNNNNNNNNNNNNNNNNNNNNNNNNNNNNNNNNNNNNNNNNNNNNNNNNNNNNNNNNNNNNNNNNNNNNNNNNNNNNNNNNNNNNNNNNNNNNNNNNNNNNNNNNNNNNNNNNNNNNNNNNNNNNNNNNNNNNNNNNNNNNNNNNNNNNNNNNNNNNNNNNNNNNNNNNNNNNNNNNNNNNNNNNNNNNNNNNNNNNNNNNNNNNNNNNNNNNNNNNNNNNNNNNNNNNNNNNNNNNNNNNNNNNNNNNNNNNNNNNNNNNNNNNNNNNNNNNNNNNNNNNNNNNNNNNNNNNNNNNNNNNNNNNNNNNNNN

>HM106897

TCGATGTCCTACACRTGGACAGGCGCCATGATCACGCCGTGCKCCGCGGAGGAAAGCAAGCTGCCCATCAACGCGCTGAGCAACTCTTTGCTGCGTCACCACAACATGGTCTATGCCACAACATCTCGCAGCGCAAGCCAGCGGCAGAAGAAGGTCACCTTTGACAGACTGCAGGTCCTGGACGACCATTACCGGGACGTGCTCAAGGAGATGAAGGCGAAGGCGTCCACRGTTAAGGCTAAACTTCTATCCGTAGAAGAAGCCTGCAAACTGACGCCCCCACACTCGGCCAGATCCAAATTTGGCTATGGGGCAAAGGACGTCCGGAACCTATCCAGCAAGGCCRTCAACCACATCCACTCCGTGTGGAAGGACTTGCTGGAAGACACYGAAACACCAATTGACACCACCATCATGGCAAAAAATGAGGTTTTCTGCGTCCAACCAGAGAAAGGAGGCCGCAAGCCAGCTCGCCTTATCGTATACCCMGACTTGGGGGTTCGTGTGTGCGAGAAAATGGCCCTTTAYGACGTGGTCTCCACCCTTCCTCAGGCCGTGATGGGCTCCTCATACGGATTCCAGTACTCTCCTGGACAGCGGGTCGAGTTCCTGGTGAATGCCTGGAAAAAAAAGAAAAMCCCTATGGGCTTCGCATATGACACCCGCTGTTTTGACTCAACGGTCACCGAGAGTGACATCCGTGTTGAGGAGTCAATCTACCAATGTTGTGACTTGGCCCCCGAAGCCAGACAGGCCATAAGGTCGCTCACAGAGCGGCTTTATATCGGGGGTCCCCTGACTAACTCAAAAGGGCAGAACTGCGGTTATCGCCGGTGCCGCGCRAGCGGTGTACTGACGACCAGCTGCGGTAATACCCTCACATGTTACTTGAAGGCCTCTGCAGCCTGTCGAGCTGCCAAGCTCCAGGACTGCACGATGCTCGTGTGCGGAGACGACCTTGTCGTTATCTGTGAGAGCGCGGGAACCCAGGAGGACGCGGCGAGCCTACGAGTCTTCACGGAGGCTATGACTAGGTACTCTGCCCCCCCCGGGGACCCGCCCAAACCAGAATACGACTTGGAGTTGATAACATCATGCTCCTCCAATGTGTCGGTCGCGCACGATGCATCCGGCAAACGGGTGTACTACCTCACCCGTGAYCCCACCACCCCACTTGCACGGGCTGCGTGGGARACAGCTAGACACACTCCAGTCAACTCCTGGCTAGGCAACATCATCATGTATGCGCCCACCTTATGGGCAAGGATGATTCTGATGACTCACTTCTTCTCCATCCTTCTAGCTCAGGAACAACTTGAAAAGGCTCTAGATTGTCAGATCTACGGGGCCTGTTACTCCATTGAACCACTTGACCTACCTCAGATCATTCAGCGACTCCATGGNNNNNNNNNNNNNNNNNNNNNNNNNNNNNNNNNNNNNNNNNNNNNNNNNNNNNNNNNNNNNNNNNNNNNNNNNNNNNNNNNNNNNNNNNNNNNNNNNNNNNNNNNNNNNNNNNNNNNNNNNNNNNNNNNNNNNNNNNNNNNNNNNNNNNNNNNNNNNNNNNNNNNNNNNNNNNNNNNNNNNNNNNNNNNNNNNNNNNNNNNNNNNNNNNNNNNNNNNNNNNNNNNNNNNNNNNNNNNNNNNNNNNNNNNNNNNNNNNNNNNNNNNNNNNNNNNNNNNNNNNNNNNNNNNNNNNNNNNNNNNNNNNNNNNNNNNNNNNNNNNNNNNNNNNNNNNNNNNNNNNNNNNNNNNNNNNNNNNNNNNNNNNNNNNN

>HM106898

ACGATGTCCTACACATGGACAGGCGCCCTGATCACGCCGTGCTCCGCGGAGGAAAGCAAGCTGCCCATCAACGCGTTGAGCAACTCTYTGCTGCGTCACCACAACATGGTCTATGCCACAACATCTCGCAGCGCAAGCCAGCGGCAGAAGAAGGTCACTTTYGACAGACTGCAGGTCCTGGACGACCATTACCGGGACGTGCTCAAGGAGATGAAGGCGAAGGCGTCCACGGTTAAGGCTAAACTTCTATCTGTAGAAGAGGCCTGCATGCTGACGCCCCCACACTCGGCCAAATCYAAATTTGGCTATGGGGCRAAGGACGTCCGGAACCTATCCAGCAAGGCCATTAACCACATCCGCTCCGTGTGGAAGGACTTGCTGGAAGACACTGAGACACCAATYGACACCACCATCATGGCAAAAAATGAGGTTTTCTGYGTCCAACCAGAGAAAGGAGGCCGCAAGGCAGCTCGCCTTATCGTATTCCCAGACTTGGGRGTTCGTGTGTGCGAGAAAATGGCCCTTTAYGACGTRGTCTCCACTCTTCCTCAGGCCGTGATGGGCCCCTCATACGGGTTCCAGTACTCTCCTGGACAGCGGGTCGAGTTCCTAGTGAATGCCTGGAARAAAAAGAAAAATCCTATGGGCTTCGCATATGACACCCGCTGTTTTGACTCAACGGTCACYGAGAGTGACATCCGTATTGAGGAGTCAATYTACCAATGTTGTGACTTGGCCCCCGAAGCCAGACAGGCCATAARGTCGCTCACAGAGCGGCTTTAYATCGGGGGTCCCTTGACTAACTCAAAAGGGCAGAACTGCGGTTATCGCCGGTGCCGCGCGAGCGGTGTGCTGACGACCAGCTGCGGTAATACCCTCACATGTTACTTGAAGGCCTCTGCAGCCTGTCGAGCCGCCAAGCTCCAGGACTGCACGATGCTCGTGTGCGGAGACGACCTTGTCGTTATCTGTGAAAGCGCGGGRACCCAGGAGGACGCGGCGAGCCTACGAGTCTTCACGGAGGCTATGACTAGGTACTCTGCCCCCCCCGGGGACCCGCCCAAACCAGAATACGACTTGGAGTTGATAACATCATGCTCCTCCAATGTGTCGGTCGCGCACGATGCATCTGGCAAAAGGGTGTATTACCTCACCCGTGACCCCACCACCCCACTTGCACGGGCTGCGTGGGAGACAGCTAGACACACTCCAGTCAACTCCTGGCTAGGCAACATCATCATGTAYGCGCCCACCTTATGGGCAAGGATGATCCTGATGACCCACTTCTTYTCCATCCTTCTAGCRCAGGAACAACTTGAAAAGGCTCTAGATTGTCAGATCTAYGGGGCCTGTTACTCCATTGAGCCACTTGACCTACCTCAGATCATCCAGCGACTCCATGGNNNNNNNNNNNNNNNNNNNNNNNNNNNNNNNNNNNNNNNNNNNNNNNNNNNNNNNNNNNNNNNNNNNNNNNNNNNNNNNNNNNNNNNNNNNNNNNNNNNNNNNNNNNNNNNNNNNNNNNNNNNNNNNNNNNNNNNNNNNNNNNNNNNNNNNNNNNNNNNNNNNNNNNNNNNNNNNNNNNNNNNNNNNNNNNNNNNNNNNNNNNNNNNNNNNNNNNNNNNNNNNNNNNNNNNNNNNNNNNNNNNNNNNNNNNNNNNNNNNNNNNNNNNNNNNNNNNNNNNNNNNNNNNNNNNNNNNNNNNNNNNNNNNNNNNNNNNNNNNNNNNNNNNNNNNNNNNNNNNNNNNNNNNNNNNNNNNNNNNNNNNNNNNNNN

>HM106899

TCGATGTCCTACACATGGACAGGCGCCCTGGTTACGCCATGCTCCGCGGAGGAGAGCAAGCTGCCCATCAACGCGTTGAGCAACTCTTTGCTGCGTTACCACAACATGGTCTATGCTACAACATCTCGCAGCGCAAGCCAGCGGCAGAAGAAGGTCACTTTTGACAGACTGCAGGTCCTGGATGACCATTACCGGGACGTGCTCAAGGAGATGAAGGCGAAGGCGTCCACAGTTAAGGCTAGACTTCTATCCGTAGAAGAAGCCTGCAAGCTGACGCCCCCACACTCGGCCAAATCCAAATTTGGCTATGGGGCAAAGGACGTCCGGAACCTATCCAGCAAGGCCATCAACCACATCCGCTCCGTGTGGGAGGACTTGCTGGAAGACACTGAAACACCAATCGACACCACCATCATGGCAAAAAATGAGGTTTTCTGCCTCCAACCAGAGAAAGGAGGCCGCAAACCAGCTCGCCTTATCGTATTCCCAGACTTGGGGGTTCGTGTGTGCGAGAAAATGGCCCTTTATGACGTGGTCTCCACCCTTCCTCAGGCCGTGATGGGCTCCTCATACGGGTTCCAGTACTCTCCTGGACAGCGGGTCGAGTTCCTGGTGAATGCCTGGAAAAAAAAGAAAAACCCCATGGGCTTCGCATATGACACCCGCTGTTTTGACTCAACGGTCACYGAGAGTGACATACGTGTTGAGGAGTCAATCTACCAATGTTGTGACTTGGCCCCCGAAGCCAGACAGGCCATAAGGTCGCTCACAGAGCGGCTTTATATCGGGGGTCCYCTRACTAACTCAAAGGGGCAGAACTGCGGTTATCGCCGGTGCCGCGCRAGCGGTGTGCTGACGACYAGCTGCGGTAATACCCTCACATGTTACTTGAAGGCCTCTGCAGCCTGTCGAGCTGCCAAGCTCCAGGACTGCACAATGCTCGTGTGCGGAGACGACCTTGTCGTTATCTGTGAGAGCGCGGGAACCCAGGAGGACGCGGCGAGCCTACGAGTCTTCACGGAGGCTATGACTAGGTATTCTGCCCCCCCCGGGGACCCGCCYAAACCAGAATACGACTTGGAGTTGATAACATCATGCTCCTCCAAYGTGTCGGTCGCGCACGATGCATCTGGCAAAAGGGTGTACTACCTCACCCGTGACCCCAGCAACCCACTWGCTCGGGCTGCGTGGGAGACAGCTAGACACACTCCAGTCAACTCCTGGCTAGGCAACATCATCATGTATGCGCCCACCTTATGGGCAAGGATGATCCTGATGACTCACTTCTTCTCCATCCTTCTAGCTCAGGAACAACTTGAAAAGGCTCTAGATTGTCAGATCTACGGGGCCTGTTACTCCATTGAGCCACTTGACCTACCTCAGATCATTCAGCGACTCCATGGNNNNNNNNNNNNNNNNNNNNNNNNNNNNNNNNNNNNNNNNNNNNNNNNNNNNNNNNNNNNNNNNNNNNNNNNNNNNNNNNNNNNNNNNNNNNNNNNNNNNNNNNNNNNNNNNNNNNNNNNNNNNNNNNNNNNNNNNNNNNNNNNNNNNNNNNNNNNNNNNNNNNNNNNNNNNNNNNNNNNNNNNNNNNNNNNNNNNNNNNNNNNNNNNNNNNNNNNNNNNNNNNNNNNNNNNNNNNNNNNNNNNNNNNNNNNNNNNNNNNNNNNNNNNNNNNNNNNNNNNNNNNNNNNNNNNNNNNNNNNNNNNNNNNNNNNNNNNNNNNNNNNNNNNNNNNNNNNNNNNNNNNNNNNNNNNNNNNNNNNNNNNNNNNNN

>HM106900

NNNATGTCCTACACATGGACAGGCGCCYTGATCACGCCATGCTCCGCGGAGGAAAGCAAGCTGCCCATCAACGCGTTGAGCAACTCTTTGCTGCGTCACCACAACATGGTCTATGCCACAACRTCTCGCAGCGCAAGCCAGCGGCAGAAGAAGGTCACYTTTGAYAGACTGCAGGTCCTGGACGACCATTACCGGGACGTGCTCAAGGAGATGAAGGCGAAGGCGTCCACGGTCAAGGCTAAACTTCTATCYGTRGAAGAGGCCTGCATGCTGACGCCCCCACACTCRGCCAAATCCAAATTTGGCTATGGGGCAAAGGACGTCCGGAACCTATCCAGCAAGGCCATCAACCACATCCRCTCCGTGTGGAAGGACTTGCTGGAAGACACTGAGACACCAATTGAYACCACYATCATGGCAAARAATGAGGTTTTCTGCGTCCAACCAGAGAAAGGAGGCCGCAAGCCAGCTCGCCTTATCGTATTCCCAGACTTGGGGGTTCGTGTGTGCGAGAAAATGGCCCTTTATGACGTGGTCTCCACCCTTCCTCAGGCCGTGATGGGCTCCTCATACGGGTTCCAGTACTCTCCTGGACAGCGGGTCGAGTTCCTGGTGAATGCCTGGAAAAARAAGAARAACCCTATGGGYTTCGCATATGACACCCGCTGTTTTGACTCAACGGTCACYGAGAGTGAYATCCGYGTTGAGGAGTCAATCTACCAATGTTGTGACTTGGCCCCCGAGGCCAGACAGGCCATAAGGTCGCTCACAGAGCGGCTTTATATCGGGGGTCCCCTGACTAACTCAAARGGGCAGAACTGCGGTTATCGYCGGTGCCGCGCRAGCGGYGTGCTGACGACCAGCTGCGGTAATACCCTCACATGCTAYTTGAAGGCYTCTGCAGCCTGTCGAGCTGCCAAGCTCCAGGACTGCACGATGCTCGTGTGCGGRGACGACCTTGTCGTYATCTGTGAAAGCGCGGGAACYCAGGAGGACGCGGCGAGCCTACGAGTCTTYACGGAGGCTATGACTAGGTACTCTGCCCCCCCYGGGGACCCGCCCAAACCAGAATACGACTTGGAGTTGATAACATCATGCTCCTCCAATGTGTCGGTCGCGCACGATGCATCTGGCAAAAGGGTRTACTAYCTCACCCGTGACCCCACCACCCCACTTGCACGGGCTGCGTGGGAGACAGCTAGACACACTCCAGTCAACTCCTGGCTAGGCAACATCATCATGTATGCGCCCACCTTRTGGGCAAGGATGATCCTGATGACTCACTTCTTCTCCATCCTYCTAGCTCAGGAACAACTTGAAAAGGCTCTAGATTGTCAGATCTACGGGGCCTGTTACTCCATTGAGCCACTTGACCTACCTCAGATCATTCAGCGACTCCATGGNNNNNNNNNNNNNNNNNNNNNNNNNNNNNNNNNNNNNNNNNNNNNNNNNNNNNNNNNNNNNNNNNNNNNNNNNNNNNNNNNNNNNNNNNNNNNNNNNNNNNNNNNNNNNNNNNNNNNNNNNNNNNNNNNNNNNNNNNNNNNNNNNNNNNNNNNNNNNNNNNNNNNNNNNNNNNNNNNNNNNNNNNNNNNNNNNNNNNNNNNNNNNNNNNNNNNNNNNNNNNNNNNNNNNNNNNNNNNNNNNNNNNNNNNNNNNNNNNNNNNNNNNNNNNNNNNNNNNNNNNNNNNNNNNNNNNNNNNNNNNNNNNNNNNNNNNNNNNNNNNNNNNNNNNNNNNNNNNNNNNNNNNNNNNNNNNNNNNNNNNNNNNNNNNN

>HM106901

TCGATGTCCTACACATGGACAGGCGCCYTGATCACGCCATGYTCCGCGGAGGAAAGYAAGCTGCCCATCAAYGCGTTGAGCAACTCYTTGCTGCGTCACCACAACATGGTCTATGCCACAACATCTCGCAGCGCAAGCCAGCGGCAGAAGAAGGTCACYTTTGACAGACTGCAGGTCCTGGAYGACCAYTACCGGGACGTGCTTAAGGAGATGAAGGCGAAGGCGTCCACRGTCAARGCTAGACTTCTATCCGTAGAAGAAGCCTGCATGCTGACGCCCCCACACTCAGCCAAATCCAAATTTGGCTATGGGGCAAAGGACGTCCGGAACCTATCCAGCAAGGCCATTAACCACATCCGCTCCGTGTGGAAGGACTTGCTGGAAGACACTGAGACACCAATTGACACCACCATCATGGCMAARAATGAGGTTTTCTGCGTCCAACCAGAGAAAGGAGGCCGCAAGCCAGCTCGCCTTATCGTATTCCCAGACTTGGGRGTTCGYGTGTGCGAGAAAATGGCCCTTTATGACGTGGTCTCCACCCTTCCTCARGCCGTGATGGGCTCCTCATACGGTTTCCAGTACTCYCCTGGRCAGCGGGTCGAGTTCCTGGTGAATGCCTGGAAAAARAAGAAAAACCCTATGGGCTTCGCATATGACACCCGCTGTTTTGACTCAACGGTCACCGAGAGTGAYATCCGTGTYGAGGAGTCAATCTACCAATGTTGTGACTTGGCCCCCGAAGCCAGACAGGCCATAAGGTCGCTCACAGAGCGGCTTTATATCGGRGGTCCCCTGACYAACTCAAAAGGCCAGAACTGCGGTTATCGCCGGTGCCGCGCGAGCGGTGTGCTGACGACCAGCTGCGGTAATACCCTCACATGCTACTTGAAGGCCTCTGCAGCCTGTCGAGCTGCCAAGCTCCAGGACTGCACAATGCTCGTGTGCGGAGACGACCTTGTCGTTATCTGTGARAGCGCGGGAACCCAGGAGGACGCGGCGAGCCTACGAGTCTTCACGGAGGCTATGACTAGGTACTCTGCCCCCCCCGGGGACCCGCCCACACCAGAATACGACTTGGAGYTGATAACATCATGCTCCTCCAATGTGTCGGTCGCRCACGATGCATCTGGCAAAAGGGTGTACTACCTCACCCGCGACCCCACCACCCCRCTTGCACGGGSTGCGTGGGAGACAGCTAAACACACTCCAGTCAACTCCTGGCTAGGCAACATCATCATGTACGCGCCCACCTTRTGGGCAAGGATGATCCTGATGANNNNNNNNNNNNNNNNNNNNNNNNNNNNNNNNNNNNNNNNNNNNNNNNNNNNNNNNNNNNNNNNNNNNNNNNNNNNNNNNNNNNNNNNNNNNNNNNNNNNNNNNNNNNNNNNNNNNNNNNNNNNNNNNNNNNNNNNNNNNNNNNNNNNNNNNNNNNNNNNNNNNNNNNNNNNNNNNNNNNNNNNNNNNNNNNNNNNNNNNNNNNNNNNNNNNNNNNNNNNNNNNNNNNNNNNNNNNNNNNNNNNNNNNNNNNNNNNNNNNNNNNNNNNNNNNNNNNNNNNNNNNNNNNNNNNNNNNNNNNNNNNNNNNNNNNNNNNNNNNNNNNNNNNNNNNNNNNNNNNNNNNNNNNNNNNNNNNNNNNNNNNNNNNNNNNNNNNNNNNNNNNNNNNNNNNNNNNNNNNNNNNNNNNNNNNNNNNNNNNNNNNNNNNNNNNNNNNNNNNNNNNNNNNNNNNNNNNNNNNNNNNNNNNNNNNNNNNNNNNNNNNNNNNNNNNNN

>HM106902

NNNATGTCCTACACATGGACGGGCGCCTTGATYACGCCATGCTCCGCGGAGGAAAGCAAGCTGCCCATCAACGCGTTGAGCAACTCTTTGCTGCGTCACCACAACATGGTCTATGCCACAACATCTCGCAGCGCAAGCCAGCGGCAGAAGAAGGTCACTTTTGACAGACTGCAGGTCCTGGATGACCATTACCGGGACGTGCTCAAGGAGATGAAGGCGAAGGCGTCCACAGTTAAGGCTAAACTTCTATCCGTAGAAGAAGCCTGCATGCTGACGCCCCCACACTCGGCCAAATCCAAATTTGGCTATGGGGCAAAGGACGTCCGGAACCTATCCAGCAAGGCCATTAACCACATCAACTCCGTGTGGAAGGACTTGCTGGAAGACACTGAGACACCAATTGACACCACCATCATGGCGAAAAATGAGGTTTTCTGTGTCCAACCAGAGAAAGGAGGCCGCAAGCCAGCTCGCCTTATCGTATTCCCAGACTTGGGGGTTCGTGTGTGCGAGAAAATGGCCCTYTATGACGTGGTCTCCACCCTTCCTCAGGCCGTGATGGGCTCCTCATACGGGTTCCAGTACTCTCCTGGACAGCGGGTCGAGTTCCTGGTGAATGCCTGGAAAAARAAGAARAACCCTATGGGCTTCGCATATGACACCCGCTGYTTTGACTCAACGGTCACCGAGAGTGAYATCCGTGTTGAGGAGTCAATCTACCAATGTTGTGACTTGGCCCCCGAAGCCAGACAGGCCATAAAGTCGCTCACAGARCGGCTYTAYATCGGGGGTCCCCTGACTAAYTCAAAAGGGCAGAACTGCGGTTATCGYCGGTGCCGCGCGAGCGGTGTRCTGACGACCAGCTGCGGTAAYACCCTCACATGTTACTTGAAGGCCTCKGCAGCCTGTCGAGCTGCTAAGCTCCAGGACTGCACRATGCTCGTGTGCGGAGACGACCTTGTCGTCATCTGTGAGAGCGCGGGAACCCAGGAGGACGCGGCGAGCCTACGAGTCTTCACGGAGGCTATGACTAGGTACTCTGCCCCCCCCGGGGACCCGCCCAAACCAGAATACGACTTGGAGCTGATAACATCATGCTCCTCCAATGTGTCGGTCGCGCACGATGCATCTGGCAAAAGGGTGTACTACCTCACCCGTGACCCCACCACCCCACTTGCACGGGCTGCGTGGGAGACAGCTAGACACACTCCAGTCAACTCCTGGCTAGGCAACATCATCATGTATGCGCCCACCTTGTGGGCAAGGATGATCCTGATGACTCACTTCTTCTCCATCCTTCTAGCTCAGGAACAACTTGAAAAGGCTCTAGATTGTCAAATCTACGGGGCCTGTTACTCCATTGAGCCACTTGACCTACCTCAGATCATTCAACGACTCCATGGNNNNNNNNNNNNNNNNNNNNNNNNNNNNNNNNNNNNNNNNNNNNNNNNNNNNNNNNNNNNNNNNNNNNNNNNNNNNNNNNNNNNNNNNNNNNNNNNNNNNNNNNNNNNNNNNNNNNNNNNNNNNNNNNNNNNNNNNNNNNNNNNNNNNNNNNNNNNNNNNNNNNNNNNNNNNNNNNNNNNNNNNNNNNNNNNNNNNNNNNNNNNNNNNNNNNNNNNNNNNNNNNNNNNNNNNNNNNNNNNNNNNNNNNNNNNNNNNNNNNNNNNNNNNNNNNNNNNNNNNNNNNNNNNNNNNNNNNNNNNNNNNNNNNNNNNNNNNNNNNNNNNNNNNNNNNNNNNNNNNNNNNNNNNNNNNNNNNNNNNNNNNNNNNNN

>HM106903

NNNATGTCCTACACATGGACAGGCGCCCTGATCACGCCATGCTCCGCGGAGGAAAGCAAGCTGCCCATCAACGCGTTGAGCAACTCTTTGCTGCGTCACCACAACATGGTCTATGCCACAACATCTCGCAGCGCAAGCCAGCGGCAGAAGAAGGTCACCTTTGACAGACTGCAGGTCCTGGATGACCATTACCGGGACGTGCTCAAGGAGATGAAGGCGAAGGCGTCCACGGTTAAGGCTAAACTTCTATCCGTAGAGGAAGCCTGCATGCTGACGCCCCCACACTCRGCCAAATCCAAATTTGGCTATGGGGCAAAGGACGTCCGGAACCTATCCAGCAAGGCCATTAACCACATCCGCTCCGTGTGGAAGGACTTGTTGGAAGACACYGAAACACCRATTGACACCACCATCATGGCRAAAAATGAGGTTTTCTGCGTCCAACCAGAGAAAGGAGGCCGCAAGCCAGCTCGCCTTATCGTATTCCCAGACTTGGGGGTTCGTGTGTGCGAGAAAATGGCCCTTTATGATGTGGTCTCCACCCTTCCTCAGGCCGTGATGGGCTCCTCGTACGGGTTCCAGTACTCTCCTGGGCAGCGGGTCGARTTTCTGGTGAACGCCTGGAAAAAAAAGAAAAACCCTATGGGCTTCGCATATGACACCCGCTGTTTTGACTCAACGGTCACCGAGAGTGACATCCGTGTTGAGGAGTCAATCTACCAATGTTGTGACTTGGCCCCCGAAGCCAGACAGGCCATAAGGTCACTCACAGAGCGGCTTTATATCGGGGGTCCCCTGACTAACTCAAAAGGGCAGAACTGCGGTTATCGCCGGTGCCGCGCGAGCGGAGTGCTGACGACCAGCTGCGGTAATACCCTCACATGTTACTTGAAGGCTGCTGCAGCCTGTCGAGCTGCCAAGCTCCAGGACTGCACGATGCTCGTGTGCGGAGACGACCTTGTCGTTATCTGTGAGAGCGCGGGGACCCAGGAGGATGCGGCGAGCCTACGAGTCTTCACGGAGGCYATGACTAGGTACTCTGCCCCCCCCGGGGACCCGCCCAAACCAGAATACGACTTGGAGTTAATAACATCATGCTCCTCCAACGTGTCGGTCGCGCACGACGCATCTGGCAAAARGGTGTACTACCTCACCCGTGACCCCACCACCCCACTTGCACGGGCTGCGTGGGAGACAGCTAGACACACTCCAATCAATTCCTGGCTAGGCAACATCATCATGTACGCGCCCACYCTATGGGCAAGGATGATCCTGATGACTCACTTCTTCTCCATCCTTCTAGCTCAGGAACAACTTGAAAAGGCTCTAGATTGTCAGATATACGGGGCCTGTTACTCCATTGAGCCACTTGACCTACCTCAGATCATTCARCGACTCCATGGNNNNNNNNNNNNNNNNNNNNNNNNNNNNNNNNNNNNNNNNNNNNNNNNNNNNNNNNNNNNNNNNNNNNNNNNNNNNNNNNNNNNNNNNNNNNNNNNNNNNNNNNNNNNNNNNNNNNNNNNNNNNNNNNNNNNNNNNNNNNNNNNNNNNNNNNNNNNNNNNNNNNNNNNNNNNNNNNNNNNNNNNNNNNNNNNNNNNNNNNNNNNNNNNNNNNNNNNNNNNNNNNNNNNNNNNNNNNNNNNNNNNNNNNNNNNNNNNNNNNNNNNNNNNNNNNNNNNNNNNNNNNNNNNNNNNNNNNNNNNNNNNNNNNNNNNNNNNNNNNNNNNNNNNNNNNNNNNNNNNNNNNNNNNNNNNNNNNNNNNNNNNNNNNNN

>HM106904

NNNATGTCCTACACATGGACAGGCGCTCTGATCACGCCATGCTCCGCGGAGGAAAGCAAGCTGCCCATCAACGCGTTGAGCAACTCTTTGCTGCGTCACCACAACATGGTCTATGCCACAACATCTCGCAGCGCAAGCCAGCGGCAGAAGAAGGTCACCTTTGACAGACTGCAGGTCCTGGACGACCATTACCGGGACGTGCTCAAGGAGATGAAGGCGAAGGCGTCCACRGTTAAGGCTAAGCTTCTATCCGTAGAGGAAGCCTGCATGCTGACGCCCCCACACTCGGCCAAATCCAAATTTGGCTATGGGGCAAAGGACGTCCGGAACCTATCCAGCAAGGCCGTCAACCACATCCGCTCCGTGTGGAAGGACTTGCTGGAAGACACTGAGACACCAATTGACACCACCATCATGGCAAAAAATGAGGTTTTCTGCGTCCAACCAGAGAAAGGAGGCCGCAAGCCAGCTCGCCTTATCGTATTCCCAGACTTGGGGGTTCGTGTGTGCGAGAAAATGGCCCTTTATGACGTGGTCTCCACCCTTCCTCAGGCCGTGATGGGCTCCTCATACGGGTTCCAGTACTCTCCTGGACAGCGGGTCGAGTTCCTGGTGAATGCCTGGAAAAAAAAGAAAAACCCTATGGGTTTCGCATATGACACCCGCTGTTTTGACTCAACGGTCACCGAGAGTGACATCCGTGTTGAGGAGTCGATCTACCAATGTTGTGACTTGGCCCCCGAAGCCAGACAAGCCATAAGGTCGCTCACAGAGCGGCTTTACATCGGGGGTCCCCTGACTAACTCAAAAGGGCAGAACTGCGGTTATCGCCGGTGCCGCGCGAGCGGTGTGCTGACGACCAGCTGCGGTAATACCCTTACATGTTACTTGAAGGCCTCTGCAGCCTGTCGAGCTGCCAAGCTCCAGGACTGTACGATGCTCGTGTGCGGAGACGACCTTGTCGTTATCTGTGAAAGCGCGGGGACCCAGGAGGACGCGGCGAGCCTACGAGTCTTCACGGAGGCTATGACCAGGTACTCTGCCCCCCCCGGGGACCCGCCCAAACCAGAATACGACTTGGAGTTGATAACATCATGCTCCTCCAATGTGTCGGTCGCGCACGATGCATCTGGCAAAAGGGTGTACTACCTCACCCGTGACCCCACCACCCCACTTGCACGGGCTGCGTGGGAGACAGCTAGACACACTCCAGTCAACTCCTGGCTAGGCAACATCATCATGTATGCGCCCACCTTATGGGCAAGGATGATCCTGATGACTCACTTCTTCTCCATCCTTCTAGCTCAGGAGCAGCTTNNNNNNNNNNNNNNNNNNNNNNNNNNNNNNNNNNNNNNNNNNNNNNNNNNNNNNNNNNNNNNNNNNNNNNNNNNNNNNNNNNNNNNNNNNNNNNNNNNNNNNNNNNNNNNNNNNNNNNNNNNNNNNNNNNNNNNNNNNNNNNNNNNNNNNNNNNNNNNNNNNNNNNNNNNNNNNNNNNNNNNNNNNNNNNNNNNNNNNNNNNNNNNNNNNNNNNNNNNNNNNNNNNNNNNNNNNNNNNNNNNNNNNNNNNNNNNNNNNNNNNNNNNNNNNNNNNNNNNNNNNNNNNNNNNNNNNNNNNNNNNNNNNNNNNNNNNNNNNNNNNNNNNNNNNNNNNNNNNNNNNNNNNNNNNNNNNNNNNNNNNNNNNNNNNNNNNNNNNNNNNNNNNNNNNNNNNNNNNNNNNNNNNNNNNNNNNNNNNNNNNNNNNNNNNNNNNNNNNNNNNNNNNNNNNNNNNNN

>HM106905

NNNNTGTCCTACACATGGACAGGCGCCCTGATCACGCCATGCTCCGCGGAGGAAAGCAAGCTGCCCATCAACGCGTTGAGCAACTCTTTGCTGCGCCACCACAACATGGTCTACGCCACAACATCTCGCAGCGCAAGCCAGCGGCAGAAGAAGGTCACCTTTGACAGACTGCAGGTCCTGGACGACCATTACCGGGACGTGCTCAAGGAGATRAAGGCGAAGGCGTCCACRGTTAAGGCTAAACTTCTATCCGTAGAAGAAGCCTGCATGCTGACGCCCCCACACTCGGCCAAATCCAAATTTGGCTATGGGGCAAAGGACGTCCGGAGCCTATCCAGCAAGGCCGTTAACCACATCCGCTCCGTGTGGAAGGACTTGCTGGAAGACACTGAGACACCAATTGACACCACCATCATGGCAAAAAATGAGGTTTTCTGCGTCCAACCAGAGAAAGGAGGCCGCAAGCCAGCTCGCCTTATCGTATTCCCAGACTTGGGGGTTCGTGTGTGCGAGAAAATGGCCCTTTATGACGTGGTCTCCACCCTTCCTCAGGCCGTGATGGGCTCCTCATACGGGTTCCAGTACTCTCCTGGACAGCGGGTCGAGTTCCTGGTGAATGCCTGGAAAAAAAAGAAGAACCCCATGGGCTTCGCATATGACACCCGCTGTTTTGACTCAACGGTCACCGAGAGTGACATCCGTGTTGAGGAGTCAATCTACCAATGTTGTGACTTGGCCCCCGAAGCCAGACAGGCCATAAGGTCGCTCACAGAGCGGCTTTATATCGGGGGTCCCCTGACTAACTCAAAAGGGCAGAACTGCGGTTATCGCCGGTGCCGCGCRAGCGGTGTGCTGACGACCAGCTGCGGTAATACCCTCACATGTTACTTGAAGGCCTCTGCAGCCTGTCGAGCTGCMAAGCTCCAGGACTGCACGATGCTCGTGTGCGGAGATGACCTTGTCGTTATCTGTGAAAGCGCGGGAACCCAGGAGGACGCGGCGAGCCTACGAGTCTTCACGGAGGCTATGACTAGGTACTCTGCCCCCCCCGGGGACCCGCCCAAACCAGAATACGACTTGGAGCTGATAACATCATGCTCCTCCAATGTGTCGGTCGCGCACGATGCATCTGGCAAAAGGGTGTACTACCTCACCCGTGACCCCACCACCCCACTTGCACGGGCTGCGTGGGAGACAGCTAGACACACTCCAGTCAACTCCTGGCTAGGCAACATCATCATGTACGCGCCCACCTTATGGGCAAGGATGATTCTGATGACTCACTTCTTCTCCATCCTTCTAGCCCAGGAACAACTTGAAAAGGCTCTAGATTGTCAGATCTACGGGGCCTGTTACTCCATTGAGCCACTTGACCTACCTCAGATCATTCAGCGACTCCATGGNNNNNNNNNNNNNNNNNNNNNNNNNNNNNNNNNNNNNNNNNNNNNNNNNNNNNNNNNNNNNNNNNNNNNNNNNNNNNNNNNNNNNNNNNNNNNNNNNNNNNNNNNNNNNNNNNNNNNNNNNNNNNNNNNNNNNNNNNNNNNNNNNNNNNNNNNNNNNNNNNNNNNNNNNNNNNNNNNNNNNNNNNNNNNNNNNNNNNNNNNNNNNNNNNNNNNNNNNNNNNNNNNNNNNNNNNNNNNNNNNNNNNNNNNNNNNNNNNNNNNNNNNNNNNNNNNNNNNNNNNNNNNNNNNNNNNNNNNNNNNNNNNNNNNNNNNNNNNNNNNNNNNNNNNNNNNNNNNNNNNNNNNNNNNNNNNNNNNNNNNNNNNNNNNN

>HM106906

NNNNNGTCCTACACATGGACAGGCGCCCTGATTACGCCGTGCTCCGCGGAGGAAAGCAAGCTGCCCATCAACGCGTTGAGCAACTCTTTGCTGCGTCACCACAACATGGTCTATGCCACAACATCTCGCAGCGCAAGCCAGCGGCAGAAGAAGGTCACCTTTGACAGACTGCANNNNNNNNNNNNNNNNNNNNNNNNNNNNNNNNNNNNNNNNNNNNNNNNNNNNNNNNNNNNNNNNNNNNNNNNNNNNNNNNNNNNNNNNNNNNNNNNNNNNNNNNNNNNNNNNNNNNNNNNNNNNNNNNNNNNNNNNNNNNNNNNNNNNNNNNNNNNNNNNNNNNNNNNNNNNNNNNNNNNNNNNNNNNNNNNNNNNNNNNNNNNGCTGGAAGACACTGAGACACCGATTGACACCACCATCATGGCGAAAAATGAGGTTTTCTGCGTCCAACCAGAGAAGGGAGGCCGCAAGCCAGCCCGCCTTATCGTATTCCCAGACTTGGGGGTTCGTGTGTGCGAGAAGATGGCCCTYTATGACGTAGTCTCCACCCTTCCTCAGGCCGTGATGGGCTCCTCATACGGGTTYCARTACTCTCCTGGACAGCGGGTCGAGTTCCTGGTGAAWGCCTGGAAANNNNNNNNNNNNNNNNNNNNNNNNNNNNNNNNNNNNNNNNNNNNNNNNNNNNNNNNCACTGAGAGTGACATCCGTGTTGAGGAGTCAATCTACCAATGTTGTGACTTGGCCCCCGAAGCCAGACAGGCTATAAGGTCGCTCACAGAGCGGCTTTATATCGGGGGCCCCCTGACTAATTCAAAGGGGCAGAACTGCGGCTATCGCCGGTGCCGCGCGAGCGGCGTGCTGACGACCAGCTGCGGTAATACCCTCACATGTTACTTGAAGGCCTCTGCGGCCTGTCGAGCTGCCAAGCTCCAGGACTGCACGATGCTCGTGTGCGGGGACGACCTCGTCGTTATCTGTGAAAGCGCGGGAACCCAGGAGGACGCGGCGAGCCTACGAGCCTTCACGGAGGCTATGACTAGGTACTCTGCCCCCCCCGGGGACCCGCCCAAACCAGAATACGACTTGGAGTTGATAACATCATGCTCCTCCAATGTGTCGGTCGCGCACGATGCATCTGGTAAAAGGGTATACTACCTCACCCGTGACCCCACCACCCCACTTGCGCGGGCTGCGTGGGAGACAGCTAGGCACACTCCAGTTAACTCCTGGCTAGGCAACATCATCATGTATGCGCCCACCTTATGGGCAAGGATGATCCTGATGACTCATTTCTTCTCCATCCTTCTAGCTNNNNNNNNNNNNNNNNNNNNNNNNNNNNNNNNNNNNNNNNNNNNNNNNNNNNNNNNNNNNNNNNNNNNNNNNNNNNNNNNNNNNNNNNNNNNNNNNNNNNNNNNNNNNNNNNNNNNNNNNNNNNNNNNNNNNNNNNNNNNNNNNNNNNNNNNNNNNNNNNNNNNNNNNNNNNNNNNNNNNNNNNNNNNNNNNNNNNNNNNNNNNNNNNNNNNNNNNNNNNNNNNNNNNNNNNNNNNNNNNNNNNNNNNNNNNNNNNNNNNNNNNNNNNNNNNNNNNNNNNNNNNNNNNNNNNNNNNNNNNNNNNNNNNNNNNNNNNNNNNNNNNNNNNNNNNNNNNNNNNNNNNNNNNNNNNNNNNNNNNNNNNNNNNNNNNNNNNNNNNNNNNNNNNNNNNNNNNNNNNNNNNNNNNNNNNNNNNNNNNNNNNNNNNNNNNNNNNNNNNNNNNNNNNNNNNNNNNNNNNNNNNNNNN

>HM106907

TCGATGTCCTACACRTGGACAGGCGCCCTGATCACGCCATGCTCCGCGGAGGAAAGCAAGCTGCCCATCAATGCGTTGAGCAACTCTTTGCTGCGYCAYCACAACATGGTCTAYGCYACAACATCTCGCAGCGCAAGCCAGCGGCAGAAGAAGGTCACCTTTGACAGACTGCAGGTCCTGGACGACCATTACCGGGACGTGCTCAAGGAGATGAAGGCGAAGGCGTCCACRGTTAAGGCTAAACTTCTATCCGTAGAAGAAGCCTGCATGCTGACGCCCCCACAYTCGGCCARATCCAAATTTGGCTAYGGGGCRAAGGACGTCCGGAACCTRTCCAGCAAGGCCGTTAACCACATCCGCTCCGTGTGGAAGGACTTGCTGGAAGACACTGAGACACCAATTGACACCACCGTCATGGCAAAAAATGAGGTTTTCTGCGTCGAACCAGARAAAGGAGGCCGCAAGCCAGCTCGCCTTATCGTATTCCCAGACTTGGGRGTTCGTGTGTGCGAGAARATGGCCCTTTAYGACGTGGTCTCCACCCTTCCTCAGGCCGTGATGGGCTCCTCATACGGRTTCCAGTACTCTCCTGGACAGCGGGTCGAGTTCCTGGTGAATGCCTGGAARAARAAGAARAACCCYATGGGCTTCGCATATGACACCCGCTGCTTTGACTCAACRGTCACYGAGAATGACATCCGTGTTGAGGAGTCAATCTACCARTGTTGTGACTTGGCCCCCGAAGCCAGACAGGCCATAAGGTCGCTCACAGAGCGRCTTTATATCGGGGGTCCCCTGACTAACTCAAAAGGGCAGAACTGCGGTTATCGCCGGTGCCGCGCGAGCGGTGTGCTGACGACCAGCTGCGGTAATACCCTCACATGTTACTTGAAGGCCTCTGCRGCCTGTCGAGCTGCCAAGCTCCAGGACTGCACGATGCTCGTGTGCGGAGACGACCTTGTCGTTATCTGTGARAGCGCGGGAACCCAGGAGGACGCGGCGAGCCTACGAGTCTTCACGGAGGCTATGACTAGGTACTCTGCCCCCCCCGGGGACCCGCCCAAACCAGAATACGACTTGGAGTTAATAACATCATGCTCCTCYAACGTGTCGGTCGCGCACGATGCATCYGGCAAAAGRGTGTACTACCTCACCCGTGACCCCACCACCCCGCTTGCACGGGCTGCGTGGGAGACAGCTAGACACACTCCAGTYAACTCCTGGCTAGGCAACATCATCATGTATGCGCCCACCTTATGGGCAAGGATGATCCTGATGACTCACTTCTTCTCCATCCTTCTAGCYCAGGAACAACTTGAAAAGGCTCTAGATTGYCAGATCTACGGGGCCTGTTACTCCATTGAGCCACTTGACCTACCTCAGATCATTCAGCGACTCCATGGNNNNNNNNNNNNNNNNNNNNNNNNNNNNNNNNNNNNNNNNNNNNNNNNNNNNNNNNNNNNNNNNNNNNNNNNNNNNNNNNNNNNNNNNNNNNNNNNNNNNNNNNNNNNNNNNNNNNNNNNNNNNNNNNNNNNNNNNNNNNNNNNNNNNNNNNNNNNNNNNNNNNNNNNNNNNNNNNNNNNNNNNNNNNNNNNNNNNNNNNNNNNNNNNNNNNNNNNNNNNNNNNNNNNNNNNNNNNNNNNNNNNNNNNNNNNNNNNNNNNNNNNNNNNNNNNNNNNNNNNNNNNNNNNNNNNNNNNNNNNNNNNNNNNNNNNNNNNNNNNNNNNNNNNNNNNNNNNNNNNNNNNNNNNNNNNNNNNNNNNNNNNNNNNNN

>HM106908

TCGATGTCCTACACATGGACAGGCGCCCTGATCACGCCATGCTCCGCGGAGGAAAGCAAGCTGCCCATCAACGCGTTGAGCAACTCTTTGCTGCGTCACCACAACATGGTTTATGCCACAACATCTCGCAGCGCAAGCCAGCGGCAGAAGAAGGTCACCTTTGACAGAYTGCAGGTCCTGGACGACCATTACCGGGACGTGCTCAAGGAGATGAAGGCGAAGGCGTCCACAGTTAAGGCTAAACTYCTATCCGTAGAAGAAGCCTGCATGCTGACGCCCCCACACTCGGCCAAATCCAAATTTGGCTATGGGGCAAAGGACGTCCGGAACCTATCCAGCAAGGCCATTAACCACATCCGCTCCGTGTGGAAGGACTTACTGGAAGACACTGAGACACCAATTGACACCACCATCATGGCAAAAAATGAGGTTTTCTGCGTCCAACCAGAGAAAGGAGGYCGCAAGCCGGCTCGCCTTATCGTATTCCCAGACTTGGGGGTTCGTGTGTGCGAGAAAATGGCCCTTTATGACGTGGTCTCCACCCTTCCCCAGGCCGTGATGGGCTCCTCATACGNNNNNNNNNNNNNNNNNNNNNNNNNNNNNNNNNNNNNNNNNNNNNNNNNNNNNNNNNNNNNNNNNNNNNNNNNNNNNNNNNNNNNNNNNNNNNNNNNNNNNNNNNNNNNNNNNNNNNNNNNCATCCGTGTTGAGGAGTCAATCTACCAATGTTGTGACTTGGCCCCCGAAGCCAGACAGGCCATAAGGTCGCTCACAGAGCGGCTTTACATCGGGGGTCCCCTGACTAACTCAAAAGGGCAGAACTGCGGCTATCGCCGGTGCCGCGCGAGCGGTGTGCTGACGACCAGCTGCGGTAATACCCTCACATGTTACTTGAAGGCCTCTGCAGCCTGTCGAGCTGCCAAGCTCCAGGACTGCACGATGCTTGTGTGCGGAGACGACCTTGTCGTTATCTGTGAAAGCGCGGGAACCCAGGAGGACGCGGCGAGCCTACGAGTCTTCACGGAGGCTATGACTAGGTACTCTGCCCCCCCCGGGGACCCGCCCAAACCAGAATACGACTTGGAGTTGATAACATCATGCTCCTCCAACGTGTCGGTCGCGCACGATGCATCTGGCAAAAGGGTATACTACCTCACCCGTGACCCCACCACCCCACTTGCACGGGCTGCGTGGGAGACAGCTAGACACACTCCAGTTAACTCCTGGCTAGGCAACATCATCATGTATGCGCCCACCTTGTGGGCAAGGATGATCCTGATGACTCATTTCTTCTCCATCCTTCTAGCTCAGGAACAACTTGAAAAGGCTCTAGATTGCCAGATCTACGGGGCCTGTTACTCCATTGAGCCACTTGANNNNNNNNNNNNNNNNNNNNNNNNNNNNNNNNNNNNNNNNNNNNNNNNNNNNNNNNNNNNNNNNNNNNNNNNNNNNNNNNNNNNNNNNNNNNNNNNNNNNNNNNNNNNNNNNNNNNNNNNNNNNNNNNNNNNNNNNNNNNNNNNNNNNNNNNNNNNNNNNNNNNNNNNNNNNNNNNNNNNNNNNNNNNNNNNNNNNNNNNNNNNNNNNNNNNNNNNNNNNNNNNNNNNNNNNNNNNNNNNNNNNNNNNNNNNNNNNNNNNNNNNNNNNNNNNNNNNNNNNNNNNNNNNNNNNNNNNNNNNNNNNNNNNNNNNNNNNNNNNNNNNNNNNNNNNNNNNNNNNNNNNNNNNNNNNNNNNNNNNNNNNNNNNNNNNNNNNNNNNNNNNNNNNNNNNNNNNNNNN

>HM106909

NNNATGTCCTATACATGGACAGGCGCCCTGATCACGCCATGCTCCGCGGAGGAAAGCAAGCTGCCCATCAACGCGTTGAGCAACTCTTTGCTGCGTCACCACAACATGGTCTATGCCACAACATCTCGCAGCGCAAGCCAGCGGCAGAAGAAGGTCACCTTTGACAGACTGCAGGTCCTGGATGACCATTACCGGGACGTGCTCAAGGAGATGAAGGCGAAGGCGTCCACAGTTAAGGCTAAGCTTCTATCCGTAGAAGAAGCCTGCATGCTGACGCCCCCACACTCGGCCAAATCCAAATTTGGCTATGGGGCAAAGGACGTCCGGAGCCTATCCAGCAAGGCCGTTAACCACATCCGCTCCGTGTGGAAGGACTTGCTGGAAGACACTGAGACACCAATTGACACCACCATCATGGCAAAGAATGAGGTTTTCTGCGTCCAACCAGAGAAAGGAGGCCGCAAGCCAGCTCGCCTTATCGTATTCCCAGACTTGGGGGTTCGTGTGTGCGAGAAAATGGCCCTTTATGACGTGGTCTCCACCCTTCCTCAGGCCGTGATGGGCTCCTCATACGGGTTCCAATACTCTCCTGGACAGCGGGTCGAGTTCCTGGTGAATGCCTGGAAAAAAAAGAAAAACCCTATGGGCTTCGCATATGACACCCGCTGTTTTGACTCAACGGTCACCGAGAGTGACATCCGTGTTGAGGAGTCAATCTACCAATGTTGTGACTTGGCCCCCGAAGCCAGACAGGCCATAAGGTCGCTCACAGAGCGGCTTTATATCGGGGGTCCCCTGACTAACTCAAAAGGGCAGAACTGCGGTTATCGCCGGTGCCGCGCGAGCGGAGTGCTGACGACCAGCTGCGGTAATACCCTCACATGTTACTTGAAGGCCTCTGCAGCCTGTCGAGCTGCCAAGCTCCAGGACTGCACGATGCTTGTGTGCGGAGACGACCTTGTCGTTATCTGTGAAAGCGCGGGAACCCAGGAGGACGCGGCGAGCCTACGAGTCTTCACGGAGGCTATGACTAGGTACTCTGCCCCCCCCGGGGACCCGCCCAAACCAGAATACGACTTGGAGCTGATAACATCATGCTCCTCCAATGTGTCGGTCGCGCACGATGCATCTGGCAAAAGGGTGTACTACCTCACCCGTGACCCCACCACCCCACTTGCACGGGCTGCGTGGGAGACAGCTAGACACACTCCAGTCAATTCCTGGCTAGGCAACATCATCATGTATGCGCCCACCTTATGGGCAAGGATGATCCTGATGACTCATTTCTTCTCCATCCTTCTAGCTCAGGAACAACTTGAAAAGGCTCTAGATTGTCAGATCTACGGGGCCTGTTACTCCATTGAGCCACTTGACCTACCTCAGATCATTCAACGACTCCATGGNNNNNNNNNNNNNNNNNNNNNNNNNNNNNNNNNNNNNNNNNNNNNNNNNNNNNNNNNNNNNNNNNNNNNNNNNNNNNNNNNNNNNNNNNNNNNNNNNNNNNNNNNNNNNNNNNNNNNNNNNNNNNNNNNNNNNNNNNNNNNNNNNNNNNNNNNNNNNNNNNNNNNNNNNNNNNNNNNNNNNNNNNNNNNNNNNNNNNNNNNNNNNNNNNNNNNNNNNNNNNNNNNNNNNNNNNNNNNNNNNNNNNNNNNNNNNNNNNNNNNNNNNNNNNNNNNNNNNNNNNNNNNNNNNNNNNNNNNNNNNNNNNNNNNNNNNNNNNNNNNNNNNNNNNNNNNNNNNNNNNNNNNNNNNNNNNNNNNNNNNNNNNNNNNN

>HM106910

NNNATGTCCTACACATGGACRGGCGCYCTGATCACGCCATGCKCCGCGGAGGAAAGCAAGCTGCCCATCAACGCGTTGAGCAAYTCYTTGCTGCGTCACCACAACATGGTCTATGCCACAACATCTCGCAGCGCAAGCCAGCGGCAGAAAAAGGTCACCTTTGACAGACTGCARGTCCTGGACGACCATTACCGGGACGTGCTCAAGGAGATGAAGGCGAAGGCGTCCACAGTTAAGGCTAAACTTCTATCCGTAGARGAAGCCTGYAGGCTGACGCCCCCACACTCGGCCAGATCMAAATTTGGCTATGGGGCRAAGGACGTCCGGAACCTATCCAGCAAGGCCATTAACCACATCCRCTCCGTGTGGAAGGACTTGCTGGAAGACACTGAGACACCRATTGATACYACCATCATGGCAAAAAATGAGGTTTTCTGCGTCCAACCAGAGAAAGGAGGCCGCAAGCCAGCTCGCCTTATCGTATTCCCAGACTTGGGGGTTCGTGTGTGCGAGAAAATGGCCCTTTATGACGTGGTCTCCACCCTTCCTCAGGCCGTGATGGGCTCCTCATACGGRTTCCAGTACTCTCCTGGACAGCGGGTCGAGTTCCTGGTGAATGCCTGGAAAAARAAGAARAACCCTATGGGCTTCGCATATGACACCCGCTGYTTTGACTCAACGGTCACCGAGAGTGACATCCGTGTTGAGGAGTCAATCTACCAATGTTGTGACTTGGCCCCCGAAGCCAGACAGGCCATAAGGTCGCTCACAGAGCGGCTTTAYATCGGGGGTCCCCTGACTAACTCAAAAGGGCARAACTGCGGTTATCGCCGRTGCCGCGCGAGCGGTGTGCTGACGACYAGCTGCGGTAATACCCTCACATGTTACYTGAAGGCCTCTGCAGCCTGTCGAGCTGCCAAGCTCCAGGACTGCACGATGCTCGTGTGCGGAGACGACCTTGTCGTTATCTGTGAGAGCGCGGGAACCCAGGAGGAYGCGGCGAGCCTACGAGTCTTCACGGAGGCTATGACTAGGTACTCTGCCCCCCCCGGGGACCCGCCCAAACCAGAATACGACTTGGAGYTGATAACATCATGCTCCTCCAATGTGTCGGTCGCGCACGATGCAWCTGGCAAAAGGGTGTACTACCTCACCCGTGACCCCACCACCCCACTTGCACGGGCTGCGTGGGAGACAGCTAGACACACTCCAGTCAACTCCTGGCTAGGCAACATCATCATGTATGCGCCCACCTTATGGGCAAGGATGATCCTGATGACCCACTTCTTCTCCATCCTTCTAGCTCAGGAACAACTTGAAAAGGCTCTAGATTGTCAGATCTACGGGGCCTGTTACTCCATTGAGCCACTTGACCTACCTCAGATCATTCARCGACTCCATGGNNNNNNNNNNNNNNNNNNNNNNNNNNNNNNNNNNNNNNNNNNNNNNNNNNNNNNNNNNNNNNNNNNNNNNNNNNNNNNNNNNNNNNNNNNNNNNNNNNNNNNNNNNNNNNNNNNNNNNNNNNNNNNNNNNNNNNNNNNNNNNNNNNNNNNNNNNNNNNNNNNNNNNNNNNNNNNNNNNNNNNNNNNNNNNNNNNNNNNNNNNNNNNNNNNNNNNNNNNNNNNNNNNNNNNNNNNNNNNNNNNNNNNNNNNNNNNNNNNNNNNNNNNNNNNNNNNNNNNNNNNNNNNNNNNNNNNNNNNNNNNNNNNNNNNNNNNNNNNNNNNNNNNNNNNNNNNNNNNNNNNNNNNNNNNNNNNNNNNNNNNNNNNNNNN

>HM106911

NNNNNNNNNNNNNNNNNNNNNNNNNNNNNNNNNNNNNNNNNNNNNNNNNNNNNNNNNNNNNNNNNNNNNNNNNNNNNNNNNNNNNNNNNNNNNNNNNNNNNNNNNNNNNNNNNNNNNNNNNNNNNNNNNNNNNNNNNNNNNNNNNNNNNNNNNNNNNNNNNNNNNNNNNNNNNNNNNNNNNNNNNNNNNNNNNNNNNNNNNNNNNNNNNNNNNNNNNNNNNNNNNNNNNNNNNNNNNNNNNNNNNNNNNNNNNNNNNNNNNNNNNNNNNNNNNNNNNNNNNNNNNNNNNNNNNNNNNNNNNNNNNNNNNNNNNNNNNNNNNNNNNNNNNNNNNNNNNNNNNNNNNNNNNNNNNNNNNNNNNNNNNNNNNNNNNNNNNNNNNNNNNNNNNNNNNNNNNNNNNNNNNNNNNNNNNNNNNNNNNNNNNNNNNNNNNNNNNNNNNNNNNNNNNNNNNNNNNNNNNNNNNNNNNNNNNNNNNNNNNNNNNNNNNNNNNNNNNNNNNNNNNNNNNNNNNNNNNNNNNNNNNNNNNNNNNNNNNNNNNNNNNNNNNNNNNNNNNNNNNNNNNNNNNNNNNNNNNNNNNNNNNNNNNNNNNNNNNNNNNNNNNNNNNNNNNNNNNNNNNNNNNNNNNNNNNNNNNNNNNNNNNNNNNNNNNNNNNNNNNNNNNNNNNNNNNNNNNNNNNNNNNNNNNGAGTGACATCCGTGTAGAGGAGTCAATCTACCAATGTTGTGACTTGGCCCCCGAAGCCAGACAGGCCATAAGGTCGCTCACAGAGCGGCTTTATATCGGGGGTCCCCTGACTAATTCAAAAGGGCAGAACTGCGGTTATCGCCGGTGCCGCGCGAGCGGTGTGCTGACGACCAGCTGCGGTAATACCCTCACATGTTACCTGAAGGCCTCTGCAGCCTGTCGAGCTGCCAAGCTCCAGGACTGCACGATGCTCGTGTGCGGAGACGACCTTGTCGTTATCTGTGAGAGCGCGGGAACCCAGGAGGACGCGGCGAGCCTACGAGTCTTCACGGAGGCTATGACTAGGTACTCTGCCCCCCCCGGGGACCCGCCCAAACCAGAATACGACTTGGAGTTGATAACATCATGCTCCTCCAACGTGTCGGTCGCGCACGATGCAACTGGCAAAAGGGTGTACTACCTCACCCGTGACCCCACCACCCCACTTGCACGGGCTGCGTGGGAGACAGCTAGACACACTCCAGTCAACTCCTGGCTAGGCAACATCATCATGTATGCGCCCACCTTATGGGCAAGGATGATCCTGATGACCCACTTCTTCTCCATCCTTCTAGCTCAGGAACAACTTGAAAAGGCTCTAGATTGTCAGATCTACGGGGCCTGTTACTCCATTGAACCACTTGACCTACCTCAGATCATTCAACGACTCCATGGNNNNNNNNNNNNNNNNNNNNNNNNNNNNNNNNNNNNNNNNNNNNNNNNNNNNNNNNNNNNNNNNNNNNNNNNNNNNNNNNNNNNNNNNNNNNNNNNNNNNNNNNNNNNNNNNNNNNNNNNNNNNNNNNNNNNNNNNNNNNNNNNNNNNNNNNNNNNNNNNNNNNNNNNNNNNNNNNNNNNNNNNNNNNNNNNNNNNNNNNNNNNNNNNNNNNNNNNNNNNNNNNNNNNNNNNNNNNNNNNNNNNNNNNNNNNNNNNNNNNNNNNNNNNNNNNNNNNNNNNNNNNNNNNNNNNNNNNNNNNNNNNNNNNNNNNNNNNNNNNNNNNNNNNNNNNNNNNNNNNNNNNNNNNNNNNNNNNNNNNNNNNNNNNNN

>HM106912

TCGATGTCCTACACATGGACAGGCGCYYTGATCACGCCATGTTCCGCGGAGGAAAGCAAGCTGCCCATCAACGCAYTGAGCAACTCTTTGCTGCGTCACCACAAYATGGTCTATGCCACAACATCTCGCAGCGCRAGCCAGCGGCAGAARAAGGTCACCTTTGACAGACTGCAGGTCCTGGACGACCATTACCGGGACGTGCTCAAGGAGATGAAGGCGAAGGCGTCCACRGTTAAGGCTAAACTTCTATCCGTAGAAGAAGCCTGCATGCTAACGCCCCCACACTCGGCCAAATCCAARTTYGGCTAYGGGGCAAAGGACGTCCGGAACCTATCCAGCAAGGCCATTAACCACATCCACTCCGTGTGGAAGGACTTGCTGGAAGACACTGTGACACCAATTGACACCACCATCATGGCAAAAAATGAGGTTTTCTGCGTYCAACCAGAGAAAGGAGGCCGCAAGCCAGCTCGCCTYATCGTATTCCCAGACTTGGGGGTTCGTGTGTGCGAGAAAATGGCCCTTTATGAYGTGGTCTCCACCCTTCCTCAGGCCGTGATGGGCTCCTCATACGGGTTCCAGTACTCTCCTGGACAGCGGGTCGAGTTCCTGGTGAATGCCTGGAAAAAGAAGAAAAACCCTATGGGCTTCGCATATGACACCCGCTGTTTTGACTCAACGGTCACCGAGAGTGACATCCGTGTTGAGGAGTCAATCTACCAATGTTGTGACTTGGCCCCCGAAGCCAGACAGGCCATAAGGTCGCTCACAGAGCGGCTTTATATCGGGGGTCCCCTGACTAACTCAAAAGGGCAGAACTGCGGYTATCGCCGGTGCCGCGCAAGCGGTGTGCTGACGACCAGCTGCGGTAATACCCTCACATGTTACTTGAAGGCCTCTGCAGCCTGTCGAGCTGCCAAGCTCCAGGACTGCACGATGCTCGTGTGCGGRGACGACCTTGTCGTTATCTGTGARAGCGCGGGAACCCAGGAGGACGCGGCGAGCCTACGAGTCTTCACGGAGGCTATGACTAGGTACTCTGCCCCCCCTGGGGACCCGCCCAAACCAGAATACGACTTGGAGTTGATAACATCATGCTCCTCCAATGTGTCGGTCGCRCACGATGCRTCTGGCAAAAGGGTGTACTACCTCACCCGTGACCCCACCACCCCRCTYGCACGGGCTGCGTGGGAGACAGCTAGACACACTCCAGTCAAYTCCTGGYTAGGCAACATCATCATGTATGCGCCCACCTTATGGGCGAGGATGATTYTGATGACYCATTTCTTCTCCATCCTCCTAGCTCAGGAACAACTTGAAAAGGCYCTAGATTGTCAGATCTACGGAGCCTGTTACTCCATTGAGCCACTTGACCNNNNNNNNNNNNNNNNNNNNNNNNNNNNNNNNNNNNNNNNNNNNNNNNNNNNNNNNNNNNNNNNNNNNNNNNNNNNNNNNNNNNNNNNNNNNNNNNNNNNNNNNNNNNNNNNNNNNNNNNNNNNNNNNNNNNNNNNNNNNNNNNNNNNNNNNNNNNNNNNNNNNNNNNNNNNNNNNNNNNNNNNNNNNNNNNNNNNNNNNNNNNNNNNNNNNNNNNNNNNNNNNNNNNNNNNNNNNNNNNNNNNNNNNNNNNNNNNNNNNNNNNNNNNNNNNNNNNNNNNNNNNNNNNNNNNNNNNNNNNNNNNNNNNNNNNNNNNNNNNNNNNNNNNNNNNNNNNNNNNNNNNNNNNNNNNNNNNNNNNNNNNNNNNNNNNNNNNNNNNNNNNNNNNNNNNNNNNNN

>HM106913

NNNATGTCCTACACATGGACAGGCGCCCTGATCACGCCATGCTCCGCGGAGGAAAGTAAGCTGCCCATCAACGCGTTGAGCAACTCYTTGCTGCGTCACCACAACATGGTSTATGCCACAACATCTCGCAGCGCAAGCCAGCGGCAGAAGAAGGTCACCTTTGACAGACTGCAGGTCCTGGACGACCATTACCGGGACGTGCTCAAGGAGATGAAGGCGAAGGCGTCCACGGTKAAGGCTAAACTTCTATCCGTAGAAGAAGCCTGCATGCTGACGCCCCCACACTCGGCCAAATCTAAATTTGGCTATGGGGCRAAGGACGTCCGGAACCTATCCAGCAAGGCCATTAACCACATCCRCTCCGTGTGGAAGGACTTGCTGGAAGACACTGAGACACCAATTGACACCACCATCATGGCAAAAAATGAGGTTTTCTGCGTCCAACCAGAGAAAGGAGGCCGCAARCCAGCTCGCCTTATCGTATTCCCAGACTTGGGGGTTCGYGTGTGCGAGAAAATGGCCCTTTATGACGTGGTCTCCACCCTTCCTCAGGCCGTGATGGGCTCCTCATACGGGTTCCAGTACTCTCCTGGACAGCGGGTCGAGTTCCTGGTGAATGCCTGGAAAAAAAAGAAGAACCCTATGGGCTTCGCRTATGACACCCGCTGTTTTGACTCAACGGTCACCGAGAGTGACATCCGTGTTGAGGAGTCRATYTACCAATGTTGTGACTTGGCCCCCGAAGCCAGACAGGCCATAAGGTCGCTCACAGAGCGGCTTTATATCGGGGGTCCCCTGACYAACTCAAAAGGGCAGAACTGCGGTTATCGCCGGTGCCGCGCGAGCGGTGTGCTGACGACCAGCTGCGGTAATACCCTYACATGTTACTTGAAGGCCTCTGCAGCCTGTCGAGCTGCCAAGCTCCAGGACTGCACGATGCTCGTGTGCGGAGACGACCTTGTCGTTATCTGTGAAAGYGCGGGAACCCAGGAGGACGCGGCGARCCTACGAGTCTTCACGGAGGCTATGACTAGGTACTCTGCCCCCCCCGGGGACCCGCCCAAACCAGAATACGACTTGGAGCTGATAACATCATGCTCCTCCAATGTGTCGGTCGCACACGATGCATCTGGCAAAAGGGTGTACTACCTCACCCGTGACCCCACCACCCCRCTTGCACGGGCTGCGTGGGAGACAGCTAGACACACTCCAGTCAACTCCTGGCTAGGCAACATCATCATGTATGCGCCCACCTTRTGGGCAAGGATGATCCTGATGACTCACTTCTTYTCCATCCTTCTAGCTCAGGAACAACTTGGAAAGGCTCTAGATTGTCAGATCTACGGGGCCTGTTACTCCATTGAGCCACTTGACCTACCTCAGATCATACAGMGACTCCATGGNNNNNNNNNNNNNNNNNNNNNNNNNNNNNNNNNNNNNNNNNNNNNNNNNNNNNNNNNNNNNNNNNNNNNNNNNNNNNNNNNNNNNNNNNNNNNNNNNNNNNNNNNNNNNNNNNNNNNNNNNNNNNNNNNNNNNNNNNNNNNNNNNNNNNNNNNNNNNNNNNNNNNNNNNNNNNNNNNNNNNNNNNNNNNNNNNNNNNNNNNNNNNNNNNNNNNNNNNNNNNNNNNNNNNNNNNNNNNNNNNNNNNNNNNNNNNNNNNNNNNNNNNNNNNNNNNNNNNNNNNNNNNNNNNNNNNNNNNNNNNNNNNNNNNNNNNNNNNNNNNNNNNNNNNNNNNNNNNNNNNNNNNNNNNNNNNNNNNNNNNNNNNNNNNN

>HM106914

NNNNNNNNNNNNNNNNNNNNNNNNNNNNNNNNNNNNNNNNNNNNNNNNNNNNNNNNNNNNNNNNNNNNNNNNNNNNNNNNNNNNTCTTTGCTGCGTCACCACAACATGGTATATGCCACAACATCTCGCAGCGCAAGCCAGCGGCAGAAGAAGGTCACCTTTGACAGACTGCAAGTCCTGGACGACCATTACCGGGACGTGCTCAAGGAGATGAAGGCGAAGGCGTCCACAGTTAAGGCTAAACTTCTATCCGTAGAAGARGCCTGCATGCTGACGCCCCCACATTCGGCCAAATCCAAATTTGGCTATGGGGCAAAGGACGTCCGGAACCTATCCAGCAAGGCCATTAACCACATCCGCTCCGTGTGGAAGGACTTGCTGGAAGACACTGAGACACCAATTGACACCACCATCATGGCAAAAAATGAGGTTTTCTGCGTCCAACCGGAGAAAGGAGGCCGCAAGCCAGCTCGCCTTATCGTATTCCCAGACTTGGGGGTTCGTGTGTGCGAGAAAATGGCCCTTTATGACGTGGTCTCCACCCTTCCTCAGGCCGTGATGGGCCCCTCATACGGATTCCAGTACTCTCCTGGACAGCGGGTCGAGTTCCTGGTGAATGCCTGGAAAAAGAAGAAAAACCCTATGGGCTTCGCATATGACACCCGCTGTTTTGACTCAACGGTCACCGAGAGTGATATCCGTGTTGAGGAGTCAATCTACCAATGTTGTGACTTGGCCCCCGAAGCCAGACAGGCCATAAGGTCGCTCACAGAGCGGCTTTATATCGGGGGTCCCCTGACTAACTCAAAAGGGCAGAACTGCGGTTATCGCCGGTGCCGCGCGAGCGGTGTGCTGACGACCAGCTGCGGTAATACCCTCACATGTTACTTGAAGGCCTCTGCAGCCTGTCGAGCTGCCAAGCTCCAGGACTGCACGATGCTCGTGTGCGGAGACGACCTTGTCGTTATCTGTGAGAGCGCGGGAACCCAGGAGGACGCGGCGAGCCTACGAGTCTTCACGGAGGCTATGACTAGGTACTCTGCCCCCCCCGGGGACCCGCCCAAACCAGAATACGACTTGGAGTTGATAACATCATGCTCCTCCAATGTGTCGGTCGCGCACGATGCATCTGGCAAAAGGGTGTACTACCTCACCCGTGACCCCACCACCCCACTTGCACGGGCTGCGTGGGAGACAGCTAGACACACTCCAGTTAACTCCTGGCTAGGCAACATCATCATGTATGCGCCCACCTTATGGGCAAGGATGATCCTGATGACTCACTTCTTCTCCATCCTTCTAGCTCAGGARCAACTTGAAAAGGCTCTAGATTGTCAGATCTACGGAGCCTGTTACTCCATTGAACCACTTGACCTACCTCAGATCATTCAGCGACTCCATGGNNNNNNNNNNNNNNNNNNNNNNNNNNNNNNNNNNNNNNNNNNNNNNNNNNNNNNNNNNNNNNNNNNNNNNNNNNNNNNNNNNNNNNNNNNNNNNNNNNNNNNNNNNNNNNNNNNNNNNNNNNNNNNNNNNNNNNNNNNNNNNNNNNNNNNNNNNNNNNNNNNNNNNNNNNNNNNNNNNNNNNNNNNNNNNNNNNNNNNNNNNNNNNNNNNNNNNNNNNNNNNNNNNNNNNNNNNNNNNNNNNNNNNNNNNNNNNNNNNNNNNNNNNNNNNNNNNNNNNNNNNNNNNNNNNNNNNNNNNNNNNNNNNNNNNNNNNNNNNNNNNNNNNNNNNNNNNNNNNNNNNNNNNNNNNNNNNNNNNNNNNNNNNNNNN

>HM106915

NNNNTGTCCTACACATGGACAGGCGCCCTGATCACGCCATGCTCCGCGGAGGAAAGCAAGCTGCCCATCAACGCGTTGAGCAACTCTTTGCTGCGTCAYCACAACATGATCTACGCCACAACATCTCGYAGTGCAAGCCAGCGGCAGAAGAAGGTCACCTTTGACAGACTGCAGGTCCTGGACGACCATTACCGGGACGTGCTCAAGGAGATGAAGGCGAAGGCGTCCACRGTTAAGGCTAAACTTCTATCCGTRGAAGARGCCTGCAAGCTGACGCCCCCACACTCGGCCAAATCCAAATTTGGCTATGGGGCAAAGGACGTCCGGAACCTATCCAGCAAGGCCATTAACCACATCCACTCCGTGTGGAAGGACTTGCTGGAAGACACTGAGACACCAATTGAYACCACCATCATGGCAAAAAATGAGGTYTTCTGCGTCCAACCAGAGAAAGGAGGCCGCAAGCCAGCTCGCCTYATCGTRTTCCCAGACTTGGGGGTKCGTGTGTGCGAGAAAATGGCCCTTTATGACGTGGTCTCCACCCTTCCTCAGGCCGTGATGGGCTCCTCATACGGGTTCCAGTACTCTCCTGGACAGCGGGTCGAGTTCCTGGTGAATGCCTGGAARAAAAAGAARAACCCTATGGGCTTCGCATATGACACCCGCTGTTTTGACTCAACGGTCACCGAGAGTGACATCCGTGTTGAGGAGTCAATCTACCAATGTTGTGACTTGGCCCCCGAAGCCAGACAGGCCATAAGGTCGCTCACRGAGCGGCTTTAYATCGGGGGTCCCCTGACTAACTCAAAAGGGCAGAACTGCGGTTATCGCCGGTGCCGCGCGAGCGGCGTGCTGACGACYAGCTGCGGTAATACCCTCACATGTTACTTGAAGGCCTCTGCAGCCTGTCGAGCKGCCAAGCTCCAGGACTGCACGATGCTCGTGTGCGGRGACGACCTTGTCGTTATCTGTGAAAGCGCGGGAACCCAGGAGGACGCGGCGAGCCTACGAGCCTTCACGGAGGCTATGACTAGGTACTCTGCCCCYCCCGGGGACCCGCCYAAACCAGAATACGACTTGGAGTTGATAACATCATGCTCCTCCAATGTGTCGGTCGCGCACGATGCATCYGGCAAAAGGGTRTACTACCTCACCCGWGACCCCACCACCCCACTTGCRCGGGCTGCGTGGGAGACAGCTAGACACACTCCAGTCAACTCCTGGCTAGGCAACATYATCATGTATGCGCCCACCTTRTGGGCAAGGATGATCYTGATGACYCACTTCTTCTCCATCCTTCTAGCTCAGGAACAACTTGAAAAGGCTCTRGATTGYCAGATCTACGGGGCCTGTTACTCCATTGAGCCACTTGACCTACCTCAGATCATTCAGCGACTCCATGGNNNNNNNNNNNNNNNNNNNNNNNNNNNNNNNNNNNNNNNNNNNNNNNNNNNNNNNNNNNNNNNNNNNNNNNNNNNNNNNNNNNNNNNNNNNNNNNNNNNNNNNNNNNNNNNNNNNNNNNNNNNNNNNNNNNNNNNNNNNNNNNNNNNNNNNNNNNNNNNNNNNNNNNNNNNNNNNNNNNNNNNNNNNNNNNNNNNNNNNNNNNNNNNNNNNNNNNNNNNNNNNNNNNNNNNNNNNNNNNNNNNNNNNNNNNNNNNNNNNNNNNNNNNNNNNNNNNNNNNNNNNNNNNNNNNNNNNNNNNNNNNNNNNNNNNNNNNNNNNNNNNNNNNNNNNNNNNNNNNNNNNNNNNNNNNNNNNNNNNNNNNNNNNNNN

>HM106916

NNNATGTCCTACACATGGACGGGCGCCCTGATCACGCCATGCTCCGCGGAGGAAAGTAAGCTGCCCATCAAYGCGTTGAGCAACTCCTTGCTGCGTCAYCACAACATGGTCTATGCYACAACATCTCGCAGCGCAAGCCAGCGGCAGARGAAGGTCACCTTTGACAGACTGCAGGTCCTGGACGACCATTACCGGGACGTGCTCAAGGAGATGAAGGCAAAGGCGTCCACRGTTAAGGCTAAACTYCTATCCGTAGAAGAAGCCTGCAWGCTGACGCCCCCACACTCRGCCAAATCCAAATTTGGCTAYGGGGCGAAGGACGTCCGGAGCCTATCCAGCAAGGCCATTAACCACATCCACTCCGTGTGGAAGGACTTGCTGGAAGACACTGAGACACCAATTGACACCACCATCATGGCRAAAAATGAGGTTTTCTGCGTCCAACCAGAGAAAGGAGGCCGCAAGCCAGCTCGCCTTATCGTATTCCCAGACTTGGGGGTTCGHGTGTGCGAGAAAATGGCCCTTTATGACGTGGTCTCCACCCTTCCTCAGGCCGTGATGGGCTCCTCATACGGGTTCCAGTACTCTCCTGGRCAGCGGGTCGAGTTCYTGGTGAATGCCTGGAAGAAAAAGAAAAACCCTATGGGCTTYGCATATGACACYCGCTGTTTTGACTCGACRGTCACCGAGAGTGACATCCGTGTTGAGGAGTCAATCTAYCAATGTTGTGACTTRGCCCCCGAAGCCAGACAGGCCATAAGGTCGCTCACAGAGCGGCTTTAYATCGGGGGTCCCCTGACYAACTCAAAAGGGCAGAACTGCGGTTATCGCCGGTGCCGCGCGAGCGGCGTGCTGACGACCAGCTGCGGTAATACCCTYACATGTTACTTGAAGGCCTCTGCAGCCTGTCGAGCTGCCAAGCTCCAGGACTGCACGATGCTCGTGTGCGGAGACGACCTTGTCGTTATCTGTGAAAGCGCGGGAACCCAGGAGGACGCGGCGAGCCTACGAGTCTTCACGGAGGCTATGACTAGGTAYTCTGCCCCCCCCGGGGACCCGCCCAAACCAGAATACGACTTGGAGYTGATAAYATCATGCTCCTCCAATGTGTCGGTCGCGCACGATGCATCTGGCAAAAGGGTGTACTACCTCACCCGTGACCCCACCACCCCACTTGCACGGGCYGCGTGGGAGACAGCYWGACACACTCCAGTYAACTCCTGGCTAGGCAACATCATHATGTATGCGCCCACCCTATGGGCAAGGATGATCYTGATGACTCACTTCTTCTCCATCCTYCTAGCTCAGGAACAACTTGAAAAGGCTCTAGATTGCCAGATCTACGGGGCCTGTTACTCCATTGAGCCACTTGACCTACCTCAGATCATTCAGCGACTCCACGGTCTTAGCGCATTTTCACTCCATAGTTACTCTCCAGGTGAAATCAATAGGGTGGNNNNNNNNNNNNNNNNNNNNNNNNNNNNNNNNNNNNNNNNNNNNNNNNNNNNNNNNNNNNNNNNNNNNNNNNNNNNNNNNNNNNNNNNNNNNNNNNNNNNNNNNNNNNNNNNNNNNNNNNNNNNNNNNNNNNNNNNNNNNNNNNNNNNNNNNNNNNNNNNNNNNNNNNNNNNNNNNNNNNNNNNNNNNNNNNNNNNNNNNNNNNNNNNNNNNNNNNNNNNNNNNNNNNNNNNNNNNNNNNNNNNNNNNNNNNNNNNNNNNNNNNNNNNNNNNNNNNNNNNNNNNNNNNNNNNNNNNNNNNNNNNNNNNNNNNNNNNN

>HM106917

TCGATGTCCTACACATGGACAGGCGCCCTGATCACGCCATGCNNNNNNNNNNNNNNNNNNNNNNNNNNNNNNNNNNNNNNNAACTCTTTGCTGCGTCACCACAACATGGTCTATGCCACAACATCTCGCAGCGCAAGCCAGCGGCAGAAGAAGGTCACCTTTGACAGACTGCAGGTCCTGGACGACCATTACCGGGACGTGCTCAAGGAGATGAAGGCGAAGGCGTCCACRGTTAAGGCTAAACTTCTATCCGTAGAAGAAGCCTGCAKGCTGACGCCCCCACACTCGGCCAAATCCAARTTTGGCTATGGGGCAAAGGACGTCCGGAACCTATCCAGCAAGGCCATTAACCACATCCTCTCCGTGTGGAAGGACTTGCTGGAAGACACTGAGACACCAATTGACACCACCATCATGGCAAAAAAYGAGGTTTTCTGYGTCCAACCAGAGAAAGGAGGCCGCAAGCCAGCTCGCCTTATCGTATTCCCAGACTTGGGGGTTCGTGTGTGCGAGAAAATGGCCCTTTATGACGTGGTCTCCACCCTTCCTCAGGCCGTGATGGGCTCCTCATACGGGTTCCAGTACTCTCCTGGACAGCGGGTCGAGTTCCTGGTGAATGCCTGGAAAAAAAAGAAGAACCCTATGGGCTTCGCATATGACACCCGCTGTTTYGACTCAACRGTCACCGAGAGTGACATCCGTGTTGAGGAGTCAATCTACCAATGTTGTGACTTGGCCCCCGAAGCCAGACAGGCCATAAGGTCGCTCACAGAGCGGCTTTATATCGGGGGTCCCCTGACTAACTCAAAAGGGCARAACTGCGGTTATCGCCGGTGCCGCGCGAGCGGTGTGCTGACGACCAGCTGCGGTAATACCCTCACATGTTACTTGAAGGCCTCTGCAGCCTGTCGAGCTGCCAAGCTCCAGGACTGCACGATGCTCGTGTGCGGAGACGACCTTGTCGTTATCTGTGAAAGCGCGGGAACCCAGGAGGACGCGGCGAGCCTACGAGTYTTCACGGAGGCTATGACTAGGTACTCTGCCCCCCCCGGGGACCCGCCCAAACCAGAATACGACTTGGAGTTGATAACATCATGCTCCTCCAATGTGTCGGTCGCGCACGATGCATCTGGCAAAAGGGTGTACTACCTCACCCGTGACCCCACCACCCCACTTGCACGGGCTGCGTGGGAGACAGCTAGACACACTCCAGTCAACTCCTGGCTAGGCAACATCATCATGTATGCGCCCACCTTATGGGCAAGGATGATCCTGATGACTCACTTCTTCTCCATCCTTCTAGCTCAGGAACAACTTGAAAAGGCTYTAGATTGTCAGATCTACGGGGCCTGTTACTCCATTGAGCCACTTGACCTACCTCARATCATTCAGCGACTCCATGGTCTTAGCGCATTTTCACTCCATAGTTACTCTCCAGGTGAGATCAATAGGGTGGCTGCATGCCTCAGAAAACTTGGGGTACCACCCTTGCGAGTCTGGAGACATCGGGCCAGAAGTGTCCGCGCTAAGCTRCTGTCCCAGGGGGGGAGGGCTGCCAACTGTGGCAAATACCTCTTCAACTGGNNNNNNNNNNNNNNNNNNNNNNNNNNNNNNNNNNNNNNNNNNNNNNNNNNNNNNNNNNNNNNNNNNNNNNNNNNNNNNNNNNNNNNNNNNNNNNNNNNNNNNNNNNNNNNNNNNNNNNNNNNNNNNNNNNNNNNNNNNNNNNNNNNNNNNNNNNNNNNNNNNNNNNNNNNNNNNNNNNNNNNNNNNNNN

>HM106918

NNNATGTCCTACACATGGACAGGCGCCCTGATCACACCATGCTCCGCGGAGGAAAGCAAGTTGCCCATCAACGCGTTGAGCAACTCTTTGCTGCGTCACCACAACATGGTCTATGCCACAACATCTCGCAGCGCAAGCCAGCGGCAGAAGAAGGTCACTTTTGACAGACTGCAGGTCCTGGACGACCATTACCGGGACGTGCTTAAGGAGATGAAGGCAAAGGCGTCCACGGTTAAGGCTAAACTTCTATCCGTAGAAGAAGCCTGCAAGCTGACGCCCCCACACTCGGCCAAATCYAAATTTGGCTACGGGGCAAAGGACGTCCGGAACCTGTCCAGCAAGGCCACTAACCACATCCGCTCCGTGTGGAAGGACTTGCTGGAAGACACTGAGACACCAATTGACACCACCATCATGGCGAAAAATGAGGTTTTCTGCGTCCAACCAGAGAAAGGAGGCCGCAAGCCAGCTCGCCTTATCGTATTCCCAGACTTGGGGGTTCGTGTGTGTGAGAAAATGGCCCTTTATGACGTGGTCTCCACCCTTCCTCAGGCCGTGATGGGCTCCTCATACGGGTTCCAGTACTCTCCTGGACAGCGGGTCGAGTTCCTGGTGAATGCCTGGAAAAAGAAGAARAACCCTATGGGCTTCGCATATGACACCCGCTGTTTTGACTCAACGGTCACCGAGAGTGACATCCGTGTTGAGGAGTCAATCTACCAATGTTGTGACTTGGCCCCCGAAGCCAGACAGGCCATAAGGTCGCTCACAGAGCGGCTTTATATCGGGGGTCCCCTGACTAACTCAAAAGGGCAAAACTGCGGCTATCGCCGGTGCCGCGCGAGCGGTGTGCTGACGACYAGCTGCGGTAATACCCTCACATGTTACTTGAAGGCCTCTGCAGCCTGTCGAGCYGCCAAGCTCCAGGACTGCACGATGCTCGTGTGCGGAGACGAYCTTGTCGTTATCTGTGAAAGCGCGGGAACCCAGGAGGACGCGGCGAGCCTACGAGTCTTCACGGAGGCTATGACTAGGTACTCTGCCCCCCCCGGGGATCCGCCCAAACCAGAATACGACTTGGAGTTGATAACATCATGCTCCTCCAACGTGTCGGTCGCGCACGATGCATCTGGCAAAAGGGTGTACTATCTCACCCGTGACCCCACCACCCCACTCGCACGGGCTGCGTGGGAGACAGCTAGACACACTCCAGTCAATTCCTGGCTAGGSAACATCATCATGTATGCGCCCACCTTATGGGCAAGGATGATCCTGATGACTCACTTCTTCTCCATCCTTCTAGCTCAGGAACAACTTGAAAAGGCTCTAGATTGTCANNNNNNNNNNNNNNNNNNNNNNNNNNNNNNNNNNNNNNNNNNNNNNNNNNNNNNNNNNNNNNNNNNNNNNNNNNNNNNNNNNNNNNNNNNNNNNNNNNNNNNNNNNNNNNNNNNNNNNNNNNNNNNNNNNNNNNNNNNNNNNNNNNNNNNNNNNNNNNNNNNNNNNNNNNNNNNNNNNNNNNNNNNNNNNNNNNNNNNNNNNNNNNNNNNNNNNNNNNNNNNNNNNNNNNNNNNNNNNNNNNNNNNNNNNNNNNNNNNNNNNNNNNNNNNNNNNNNNNNNNNNNNNNNNNNNNNNNNNNNNNNNNNNNNNNNNNNNNNNNNNNNNNNNNNNNNNNNNNNNNNNNNNNNNNNNNNNNNNNNNNNNNNNNNNNNNNNNNNNNNNNNNNNNNNNNNNNNNNNNNNNNNNNNNNNNNNNNNNNNNNNNNNNNNNNNNNNN

>HM106919

NNNNTGTCCTACACATGGACAGGCGCCCTGATCACGCCATGCTCCGCGGAGGAAAGCAAGCTGCCCATCAACGCGTTGAGCAACTCTTTGCTGCGTCAYCACAACATGGTCTATGCCACAACATCYCGCAGCGCAAGYCAGCGGCAGAAGAAGGTCACYTTTGACAGACTGCAGGTCCTGGAYGACCATTACCGGGACGTGCTYAAGGAGATGAAGGCGAAGGCGTCCACAGTTAAGGCTAAACTCCTATCCGTAGAAGAAGCCTGCATGCTGACGCCCCCACACTCGGCCAAATCCAAATTTGGCTATGGGGCAAAGGACGTCCGGAACCTATCCAGCAAGGCCACTGGCCACATCCGCTCCGTGTGGAAGGACTTGCTGGAAGACACTGAGACACCAATTGACACCACCATCATGGCGAAAAATGAGGTTTTCTGCGTCCAACCAGAGAAAGGAGGCCGCAAGCCAGCTCGCCTTATCGTATTCCCAGACTTGGGGGTTCGTGTGTGCGAGAAAATGGCCCTTTATGACGTGGTCTCCACCCTTCCTCAGGCCGTGATGGGCTCCTCATACGGGTTCCAGTACTCTCCTGGACAGCGGGTCGAGTTCCTGGTGAATGCCTGGAAAAAAAAGAAGAACCCTATGGGCTTCGCATATGACACCCGCTGTTTTGACTCAACRGTCACCGAGAGTGACATCCGTGTTGAGGAGTCAATCTACCAATGTTGTGACTTGGCCCCCGAAGCCAGACAGGCCATAAGGTCGCTCACAGAGCGGCTTTATATCGGGGGTCCCCTGACTAACTCAAAAGGGCAGAACTGCGGTTATCGCCGGTGCCGCGCGAGCGGYGTGCTGACGACCAGCTGCGGTAATACCCTCACATGTTACTTGAAGGCCTCTGCAGCCTGTCGAGCTGCCAAGCTCCAGGACTGCACGATGCTCGTGTGCGGAGACGACCTYGTCGTTATCTGTGARAGCGCGGGAACCCAGGAGGAYGCGGCGAGCCTACGAGTCTTCACGGAGGCTATGACTAGGTACTCTGCCCCCCCCGGGGACCCGCCCAAACCAGAATACGACTTGGAGTTGATAACATCATGCTCCTCCAATGTATCGGTCGCGCACGATGCATCTGGCAAAAGGGTGTACTACCTCACCCGTGACCCCACCACCCCACTTGCACGGGCTACGTGGGAGACAGCTAGACACACTCCAGTCAACTCCTGGCTAGGCAACATCATCATGTATGCGCCCACCTTATGGGCAAGGATGATCCTGATGACTCACTTCTTCTCCATCCTTCTAGCTCAGGAACAACTTGAAAAGGCTCTAGATTGTCAGATCTACGGGGCCTGTTACTCCATTGAGCCACTTGACCTACCTCAGATCATTCAGCGACTCCATGGNNNNNNNNNNNNNNNNNNNNNNNNNNNNNNNNNNNNNNNNNNNNNNNNNNNNNNNNNNNNNNNNNNNNNNNNNNNNNNNNNNNNNNNNNNNNNNNNNNNNNNNNNNNNNNNNNNNNNNNNNNNNNNNNNNNNNNNNNNNNNNNNNNNNNNNNNNNNNNNNNNNNNNNNNNNNNNNNNNNNNNNNNNNNNNNNNNNNNNNNNNNNNNNNNNNNNNNNNNNNNNNNNNNNNNNNNNNNNNNNNNNNNNNNNNNNNNNNNNNNNNNNNNNNNNNNNNNNNNNNNNNNNNNNNNNNNNNNNNNNNNNNNNNNNNNNNNNNNNNNNNNNNNNNNNNNNNNNNNNNNNNNNNNNNNNNNNNNNNNNNNNNNNNNNN

>HM106920

NNNNNNNNNNNNNNNNNNNNNNNNNNNNNNNNNNNNNNNNNNNNNNNNNNNNNNNNNNNNNNNNNNNNNNNNNNNNNNNNNNNCTCTTTGCTGCGTCACCACAACATGATCTATGCTACAACATCTCGCAGCGCAAGCCAGCGGCAGAGGAAGGTCACCTTTGATAGACTGCAGGTCCTGGATGACCATTACCGGGACGTGCTCAAGGAGATGAAGGCGAAGGCGTCCACGGTTAAGGCTAAACTTCTATCCGTAGAAGARGCCTGCATGCTGACGCCCCCACACTCGGCCAAATCCAAATTTGGCTATGGGGCAAAGGACGTCCGGAGCCTRTCCAGCAAGGCCATTAACCACATCCGCTCCGTGTGGAAGGACTTGCTGGAAGACGCTGAGACACCAATTGACACCACCATCATGGCAAAAAATGAGGTTTTCTGCGTCCAACCCGAGAAGGGAGGCCGCAAGCCAGCTCGCCTTATCGTATTCCCAGACTTGGGGGTTCGTGTGTGCGAGAAAATGGCCCTTTATGACGTGGTCTCCACCCTTCCTCAGGCCGTGATGGGCTCCTCATACGGATTCCAGTACTCTCCTGGACAACGGGTCGAGTTCCTGGTGAATGCCTGGAAAAAAAAGAAAAACCCTATGGGCTTCGCATATGACACCCGCTGTTTTGACTCAACGGTCACCGAGAGTGACATCCGTGTTGAGGAGTCAATCTACCAGTGCTGTGACTTGGCCCCCGAAGCCAGACAGGCTATAAGGTCGCTCACAGAGCGGCTTTATATCGGGGGTCCCCTGACTAACTCAAAAGGACAGAACTGCGGTTATCGCCGGTGCCGCGCGAGCGGTGTGCTGACGACCAGCTGCGGTAATACCCTCACATGTTACCTGAAGGCCTCTGCAGCCTGTCGAGCTGCCAAGCTCCAGGACTGCACGATGCTCGTGTGCGGAGACGACCTTGTCGTTATCTGTGAAAGTGCGGGAACCCAGGAGGACGCGGCGAGCCTACGAGCCTTCACGGAGGCTATGACTAGGTACTCTGCCCCCCCCGGGGACCCGCCCAAACCAGAATACGACTTGGAGCTGATAACGTCATGCTCCTCCAATGTGTCGGTCGCGCACGAYGCATCTGGCAAAAGGGTGTACTACCTCACCCGTGACCCCACCACCCCACTTGCGCGGGCTGCGTGGGAGACAGCTAGACACACTCCAGTCAACTCCTGGCTAGGCAACATCATCATGTACGCGCCCACCTTATGGGCAAGGATGATTCTGATGACTCACTTCTTCTCCATCCTTCTAGCTCAGGARCAACTTGAGAAGGCTCTAGATTGTCAGATCTACGGGGCCTGTTACTCCATTGAGCCACTTGACCTACCTCAGATCATTNNNNNNNNNNNNNNNNNNNNNNNNNNNNNNNNNNNNNNNNNNNNNNNNNNNNNNNNNNNNNNNNNNNNNNNNNNNNNNNNNNNNNNNNNNNNNNNNNNNNNNNNNNNNNNNNNNNNNNNNNNNNNNNNNNNNNNNNNNNNNNNNNNNNNNNNNNNNNNNNNNNNNNNNNNNNNNNNNNNNNNNNNNNNNNNNNNNNNNNNNNNNNNNNNNNNNNNNNNNNNNNNNNNNNNNNNNNNNNNNNNNNNNNNNNNNNNNNNNNNNNNNNNNNNNNNNNNNNNNNNNNNNNNNNNNNNNNNNNNNNNNNNNNNNNNNNNNNNNNNNNNNNNNNNNNNNNNNNNNNNNNNNNNNNNNNNNNNNNNNNNNNNNNNNNNNNNNNNNNNNNNN

>HM106921

NNNNNNNNNNNNNNNNNNNNNNNNNNNNNNNNNNNNNNNNNNNNNNNNNNNNNNNNNNNNNNNNNNNNNNNNNNNNNNNNCAACTCTTTGTTGCGTCACCATAACATGGTCTATGCYACAACATCTCGCAGCGCAAGCCAGCGGCAGAAGAAGGTCACCTTTGACAGACTGCAGGTCCTGGACGACCATTACCGGGACGTGCTCAAGGAGATGAAGGCGAAGGCGTCCACGGTGAAGGCTAAACTTCTGTCCGTAGAAGAAGCCTGCATGCTGACGCCCCCACACTCGGCCAAATCCAAATTTGGCTATGGGGCAAAGGACGTCCGGAACCTRTCCAGCAAGGCCATTAACCACATCCACTCCGTGTGGAAGGACTTGCTGGAAGACACTGAGACACCAATTGACACTACCATCATGGCAAAGAATGAAGTTTTCTGCATCCAACCAGAGAAAGGAGGCCGCAAGCCAGCTCGCCTTATCGTATTCCCAGACTTGGGGGTTCGTGTGTGCGAGAAAATGGCCCTTTATGACGTGGTCTCCACCCTTCCTCAGGCCGTGATGGGCCCCTCATACGGGTTCCAGTACTCTCCTGGACAGCGGGTCGAGTTCCTGGTGAATGCCTGGAAAAAAAAGAAGAACCCTATGGGCTTYGCATATGACACCCGCTGTTTTGACTCAACGGTCACCGAGAGTGACATCCGTGTTGAGGAGTCAATCTATCAATGTTGTGACTTGGCCCCCGAAGCCAGACAGGCTATAAGGTCGCTCACAGAGCGGCTTTATATCGGGGGTCCCCTGACTAACTCAAAAGGGCAGAACTGCGGTTATCGCCGGTGCCGCGCGAGCGGTGTGCTGACGACCAGCTGCGGTAATACCCTCACATGTTACTTGAAAGCCTCTGCAGCCTGTCGAGCTGCCAAGCTCCAGGACTGCACGATGCTCGTGTGCGGAGACGACCTTGTCGTTATCTGTGAAAGCGCGGGAACCCAGGAGGACGCGGCGAGCCTACGAGTCTTCACGGAGGCTATGACTAGGTACTCTGCCCCYCCCGGGGACCCGCCCAAACCAGAATACGACTTGGAGTTGATAACATCATGCTCCTCCAATGTGTCGGTCGCGCACGATGCATCTGGCAAAAGGGTGTACTACCTCACCCGTGACCCYACCACCCCACTTGCACGGGCTGCGTGGGAGACAGCTAGACACACTCCAGTCAACTCCTGGCTAGGCAACATCATCATGTATGCGYCCACTTTATGGGCAAGGATGATTCTGATGACYCACTTCTTCTCCATCCTTCTAGCTCAGGAACAACTTGAAAAGGCTCTAGATTGTCAGATCTACGGGGCCTGYTACTCCATTGAGCCACTTGACCTACCTCAGATCATTCAGCGACTCCATGGNNNNNNNNNNNNNNNNNNNNNNNNNNNNNNNNNNNNNNNNNNNNNNNNNNNNNNNNNNNNNNNNNNNNNNNNNNNNNNNNNNNNNNNNNNNNNNNNNNNNNNNNNNNNNNNNNNNNNNNNNNNNNNNNNNNNNNNNNNNNNNNNNNNNNNNNNNNNNNNNNNNNNNNNNNNNNNNNNNNNNNNNNNNNNNNNNNNNNNNNNNNNNNNNNNNNNNNNNNNNNNNNNNNNNNNNNNNNNNNNNNNNNNNNNNNNNNNNNNNNNNNNNNNNNNNNNNNNNNNNNNNNNNNNNNNNNNNNNNNNNNNNNNNNNNNNNNNNNNNNNNNNNNNNNNNNNNNNNNNNNNNNNNNNNNNNNNNNNNNNNNNNNNNNNN

>HM106922

NNNNTGTCCTAYACATGGACAGGCGCCYTGATCACGCCATGCTCCGCGGAGGAGAGCAAGCTGCCCATCAACGCGTTGAGCAACTCTTTGCTGCGYCACCATAACATGGTCTATGCYACAACATCTCGCAGCGCAAGCCAGCGGCAGAAGAAGGTCACCTTTGACAGACTGCARGTCCTGGACGACCATTACCGGGACGTGCTCAAGGAGATGAAGGCGAAGGCGTCCACGGTTAAGGCTAAACTYCTATCCGTAGAAGAAGCCTGCATGCTGACGCCCCCACACTCGGCCAAATCCAAATTTGGCTATGGGGCRAAGGACGTCCGGAACCTATCCAGCAAGGCCRTTAACCACATCCACTCCGTGTGGAAGGACTTGCTGGAAGACACTGAGACACCAATTGACACCACCATCATGGCAAAAAAYGAGGTTTTCTGCGTCCAACCAGAGAAAGGAGGCCGCAAGCCAGCTCGCCTTATCGTATTCCCAGACTTGGGGGTTCGTGTGTGCGAGAAAATGGCCCTTTATGAYGTGGTCTCCACCCTTCCTCAGGCCGTGATGGGCYCCTCATACGGGTTCCAGTACTCTCCTGGACAACGGGTCGAGTTCCTGGTGAATGCCTGGAAAAAGAAGAAAAACCCYATGGGCTTCGCATATGACACCCGCTGTTTTGACTCAACGGTCACCGAGAGTGACATCCGTGTTGAGGAGTCAATCTAYCAATGTTGTGACTTGGCCCCCGAAGCCAGACAGGCCATAAGGTCGCTCACAGAGCGGCTTTATATCGGGGGYCCCCTGACTAACTCAAAAGGGCAGAACTGCGGTTATCGCCGGTGCCGCGCGAGCGGTGTGCTGACGACCAGCTGYGGTAATACCCTCACATGCTACTTGAAGGCCTCTGCAGCCTGTCGAGCTGCCAAGCTCCAGGACTGCACGATGCTCGTGTGCGGAGACGACCTTGTCGTTATCTGTGAAAGCGCGGGAACCCAGGARGACGCRGCGAGCCTACGAGTCTTCACGGGGGCTATGACTAGGTACTCTGCCCCCCCCGGGGACCCGCCCAAACCAGAATACGATTTGGAGTTGATAACATCATGCTCCTCCAATGTGTCGGTCGCGCACGATGCATCTGGTAAAAGGGTGTACTACCTCACCCGTGACCCCACCACCCCACYTGCACGGGCTGCGTGGGAGACAGCTAGACACACTCCAGTCAACTCCTGGCTAGGCAACATCATCATGTATGCGCCCACCTTGTGGGCAAGGATGATCCTGATGACTCATTTCTTCTCCATCCTTCTAGCTCAGGAACAACTTGAAAAGGCTCTAGATTGTCAGATCTATGGGGCCTGTTACTCCATTGAGCCACTTGACCTACCTCAGATCATTCAGCGACTCCATGGNNNNNNNNNNNNNNNNNNNNNNNNNNNNNNNNNNNNNNNNNNNNNNNNNNNNNNNNNNNNNNNNNNNNNNNNNNNNNNNNNNNNNNNNNNNNNNNNNNNNNNNNNNNNNNNNNNNNNNNNNNNNNNNNNNNNNNNNNNNNNNNNNNNNNNNNNNNNNNNNNNNNNNNNNNNNNNNNNNNNNNNNNNNNNNNNNNNNNNNNNNNNNNNNNNNNNNNNNNNNNNNNNNNNNNNNNNNNNNNNNNNNNNNNNNNNNNNNNNNNNNNNNNNNNNNNNNNNNNNNNNNNNNNNNNNNNNNNNNNNNNNNNNNNNNNNNNNNNNNNNNNNNNNNNNNNNNNNNNNNNNNNNNNNNNNNNNNNNNNNNNNNNNNNNN

>HM106923

TCGATGTCCTACACATGGACAGGCGCCCTGATCACGCCATGCTCYGCGGAGGAAAGTAAGCTGCCCATCAACGCGTTGAGCAACTCTTTGCTGCGTCACCACAACATGGTCTATGCYACAACATCTCGCAGCGCAAGCCAGCGGCAGAAGAAGGTCACCTTTGACAGACTGCAGGTCCTGGACGACCATTACCGGGACGTGCTCAAGGAGATGAAGGCGAAGGCGTCCACRGTTAAGGCTAARCTTCTATCCGTAGAAGAAGCCTGCATGCTGACGCCCCCACACTCGGCCAAATCCAARTTTGGCTATGGGGCAAAGGACGTCCGGAGCCTATCCAGCAAGGCCGTTARACACATCCGCTCCGTGTGGAAGGACTTGCTGGAAGACACTGAAACACCAATTGACACCACCATCATGGCAAAAAAYGAGGTYTTCTGTGTYCAACCAGAGAAAGGAGGCCGCAAGCCAGCTCGCCTTATCGTATTCCCAGATTTGGGGGTTCGTGTGTGCGAGAAAATGGCCCTCTATGACGTGGTCTCCACYCTCCCTCAGGCCGTGATGGGCTCCTCATACGGATTCCARTACTCTCCTGGACAGCGGGTCGAGTACCTGGTGARTGCCTGGAAGAAAAAGAAGAACCCCATGGGCTTCKCATATGACACCCGCTGTTTTGACTCGACRGTCACCGAGARTGACATCCGTGTTGAGGAGTCAATCTACCAATGYTGTGACTTGGCCCCCGAAGCCAGACAGGCCATAAGGTCGCTCACAGAGCGGCTTTATATTGGGGGTCCCCTGACTAACTCAAAAGGGCAGAACTGCGGTTATCGCCGGTGCCGCGCGAGCGGYGTGCTGACGACCAGCTGYGGTAATACCCTCACATGTTACTTGAAAGCCTCTGCGGCCTGTCGAGCTGCYAAGCTCCAGGACTGCACGATGCTCGTGTGCGGAGACGACCTTGTCGTTATCTGTGAAAGTGCGGGAACCCAGGAGGACGCGGCGAGCCTACGAGCCTTCACGGAGGCTATGACTAGGTACTCTGCCCCCCCCGGGGACCCGCCCAAACCAGARTACGACTTGGAGTTGATAACATCATGCTCCTCCAATGTGTCGGTCGCGCACGATGCRTCTGGCAARAGGGTGTACTACCTCACCCGTGACCCCACCACCCCGCTTGCACGGGCTGCGTGGGAAACATCTAAACACACTCCAGTCAACTCCTGGCTAGGCAACATCATCATGTATGCGCCCACCTTATGGGCAAGGATGATCTTGATGACTCACTTCTTTTCCATCCTTCTAGCYCAGGAACAACTTGAAAAGGCYCTAGATTGTCAGATCTATGGGGCCTGTTACTCCATTGAGCCACTTGACCTANNNNNNNNNNNNNNNNNNNNNNNNNNNNNNNNNNNNNNNNNNNNNNNNNNNNNNNNNNNNNNNNNNNNNNNNNNNNNNNNNNNNNNNNNNNNNNNNNNNNNNNNNNNNNNNNNNNNNNNNNNNNNNNNNNNNNNNNNNNNNNNNNNNNNNNNNNNNNNNNNNNNNNNNNNNNNNNNNNNNNNNNNNNNNNNNNNNNNNNNNNNNNNNNNNNNNNNNNNNNNNNNNNNNNNNNNNNNNNNNNNNNNNNNNNNNNNNNNNNNNNNNNNNNNNNNNNNNNNNNNNNNNNNNNNNNNNNNNNNNNNNNNNNNNNNNNNNNNNNNNNNNNNNNNNNNNNNNNNNNNNNNNNNNNNNNNNNNNNNNNNNNNNNNNNNNNNNNNNNNNNNNNNNNNNNNNNNN

>HM106924

NNNNTGTCCTACACTTGGACAGGCGCTCTGATCACGCCATGCTCCGCGGAGGAAAGCAAGCTGCCCATCAACGCGTTGAGCAACTCTTTGCTGCGTCACCACAACATGGTCTATGCCACAACATCTCGCAGCGCAAGCCAGCGGCAGAAGAAGGTCACCTTTGACAGACTGCAAGTCCTGGACRACCATTACCGGGACGTGCTYAAGGAGATGAAGGCGAAGGCGTCCACAGTTAAGGCTAAACTTCTATCCGTAGAAGAAGCCTGCATGCTGACGCCCCCACAYTCRGCCAAATCCAAATTTGGCTATGGGGCAAAGGACGTCCGGAACCTATCCAGCAAGGCCCTTAACCACATCCGCTCCGTGTGGGAGGACTTACTGGARGACACTGAGACACCAATTGACACCACCATCATGGCAAAAAATGAGGTTTTCTGCGTCCAACCAGAGAAAGGAGGCCGCAAGCCAGCTCGCCTTATCGTATTCCCAGACTTGGGGGTTCGTGTGTGCGAGAAAATGGCCCTTTATRACGTGGTCTCCACCCTCCCTCAGGCCGTGATGGGCTCCTCATACGGGTTCCAGTACTCTCCTGGACAGCGGGTCGAGTTCCTGGTGAATGCCTGGAAAAAAAAGAAGAACCCTATGGGCTTCGCATATGACACCCGCTGTTTTGACTCAACGGTCACCGAGAGTGACATCCGTGTTGAGGAGTCAATCTACCAATGTTGTGACTTGGCCCCCGAAGCCAGACAGGCCATAAGGTCGCTCACAGAGCGGCTTTATATCGGGGGTCCCCTGACTAACTCAAAAGGGCAGAACTGYGGTTATCGCCGGTGCCGCGCGAGCGGTGTGCTGACGACYAGCTGTGGTAATACCCTCACATGCTACTTGAAGGCCTCTGCAGCCTGTCGAGCTGCCAAGCTCCAGGACTGCACGATGCTCGTGTGCGGAGACGACCTTGTCGTTATCTGTGAAAGCGCGGGAACCCAGGAGGACGCGGCGAGCCTACGAGTCTTCACGGAGGCTATGACTAGGTACTCTGCCCCCCCCGGGGACCCGCCCAAACCAGAATACGACTTGGAGCTAATAACATCATGCTCCTCYAATGTGTCGGTCGCGCACGATGCATCTGGCAAAAGGGTGTACTACCTCACCCGCGACCCCACCACCCCRCTTGCACGGGCTGCGTGGGAGACAGCTAGACACACTCCAGTCAACTCCTGGCTAGGCAACATCATTATGTATGCGCCCACCTTATGGGCAAGGATGATCCTGATGACTCACTTCTTCTCCATCCTTCTAGCTCANNNNNNNNNNNNNNNNNNNNNNNNNNNNNNNNNNNNNNNNNNNNNNNNNNNNNNNNNNNNNNNNNNNNNNNNNNNNNNNNNNNNNNNNNNNNNNNNNNNNNNNNNNNNNNNNNNNNNNNNNNNNNNNNNNNNNNNNNNNNNNNNNNNNNNNNNNNNNNNNNNNNNNNNNNNNNNNNNNNNNNNNNNNNNNNNNNNNNNNNNNNNNNNNNNNNNNNNNNNNNNNNNNNNNNNNNNNNNNNNNNNNNNNNNNNNNNNNNNNNNNNNNNNNNNNNNNNNNNNNNNNNNNNNNNNNNNNNNNNNNNNNNNNNNNNNNNNNNNNNNNNNNNNNNNNNNNNNNNNNNNNNNNNNNNNNNNNNNNNNNNNNNNNNNNNNNNNNNNNNNNNNNNNNNNNNNNNNNNNNNNNNNNNNNNNNNNNNNNNNNNNNNNNNNNNNNNNNNNNNNNNNNNNNNNNNNNNNNNNNNNNNNN

>HM106925

TCGATGTCCTACACATGGACGGGCGCTCTGATYACGCCATGCTCCGCGGAGGAAAGCAAACTGCCCATCAACGCGTTGAGCAACTCTTTGCTGCGTCACCACAACATGGTCTATGCCACAACATCTCGCAGCGCAGGCCAGCGGCAGAAGAAGGTCACCTTTGACAGACTGCAGGTCCTGGACGACCATTACCGGGACGTGCTCAAGGAGATGAAGGCGAAGGCGTCCACGGTCAAGGCTAAACTTCTATCCGTAGAAGAAGCCTGCAGGCTGACGCCCCCACATTCGGCCAAATCCAAATTTGGCTATGGGGCAAAGGACGTCCGGAACCTATCCAGCAAGGCCATTAAACACATCCGCTCCGTGTGGAAGGACTTGCTGGAAGACACTGAGACACCAATTGACACCACCATCATGGCGAAAAACGAGGTTTTCTGCGTCCAACCAGAGAAAGGAGGCCGCAAGCCAGCTCGCCTTATCGTATTCCCAGACTTGGGGGTTCGTGTGTGCGAGAAAATGGCCCTTTATGACGTGGTCNNNNNNNNNNNNNNNNNNNNNNNNNNNNNNNNNNNNNNNNNNNNNNNNNNNNNNNNNNNNNNNNNNNNNNNNNNNNNNNNNNNNNNNNNNNNNNNNNNNNNNNNNNNNNNNNNNNNNNNNNNNNNNNNNNNNNNNNNNNNNNNNNTCACTGAGAGTGACATCCGCGTTGAGGAGTCAATCTACCAATGYTGTGACTTGSCCCCCGAAGCCAGACAGGCCATAAGGTCGCTCACRGAGCGGCTTTATATCGGGGGTCCCCTGACTAACTCAAAAGGGCAGAACTGCGGTTATCGCCGGTGCCGCGCGAGCGGTGTGCTGACGACTAGCTGCGGTAATACCCTCACATGTTACTTGAAGGCCTCTGCAGCCTGTCGAGCTGCCAAGCTCCAGGACTGCACGATGCTCGTGTGCGGAGACGACCTTGTCGTTATCTGTGAAAGCGCGGGGACCCARGAGGACGCGGCGAGCCTACGAGTCTTCACGGAGGCTATGACTAGGTACTCCGCCCCCCCCGGGGACCCGCCCAAACCAGAATACGACTTGGAGCTGATAACATCATGCTCCTCCAATGTGTCGGTCGCRCAYGACGCATCTGGCAAAAGGGTATACTACCTCACCCGTGACCCCACCAYCCCACTTGCGCGGGCTGCGTGGGAGACAGCTAGACACACTCCAGTCAATTCCTGGCTAGGCAACATCATCATGTATGCGCCCACCTTATGGGCAAGGATGATTCTGATGACTCACTTTTTCTCCATCCTTCTAGCTCAGGAACAACTTGAAAAGGCTCTAGATTGTCAGATCTACGGGGCCTGTTACTCCATTGAGCCACTTGACCTACCTCAGATCATTCAGCGACTCCATGGNNNNNNNNNNNNNNNNNNNNNNNNNNNNNNNNNNNNNNNNNNNNNNNNNNNNNNNNNNNNNNNNNNNNNNNNNNNNNNNNNNNNNNNNNNNNNNNNNNNNNNNNNNNNNNNNNNNNNNNNNNNNNNNNNNNNNNNNNNNNNNNNNNNNNNNNNNNNNNNNNNNNNNNNNNNNNNNNNNNNNNNNNNNNNNNNNNNNNNNNNNNNNNNNNNNNNNNNNNNNNNNNNNNNNNNNNNNNNNNNNNNNNNNNNNNNNNNNNNNNNNNNNNNNNNNNNNNNNNNNNNNNNNNNNNNNNNNNNNNNNNNNNNNNNNNNNNNNNNNNNNNNNNNNNNNNNNNNNNNNNNNNNNNNNNNNNNNNNNNNNNNNNNNNNN

>HM106926

TCGATGTCCTACACATGKACAGGCGCCCTGATCACACCATGCTCCGCGGAGGAAAGCAAGCTGCCCATCAACGCGTTGAGCAACTCTTTGCTGCGTCACCACAACATGGTCTATGCCACAACATCTCGCAGCGCAAGCCAGCGGCAGAAGAAGGTCACCTTTGACAGACTGCAGGTCCTGGACGACCATTACCGGGACGTGCTCAAGGAGATGAGGGCGAAGGCGTCCACGGTTAAGGCTAAACTTCTATCTGTAGAAGAAGCCTGTATGCTGACGCCCCCACACTCRGCCAAATCCAAATTTGGCTATGGGGCAAAGGACGTCCGGAACCTATCCAGCAAGGCCATTAACCACATCCACTCCGTGTGGAAGGACTTGCTGGAAGACACTGAGACACCAATTGACACCACCATCATGGCAAAAAATGAGGTTTTCTGCGTCCAGCCAGAGAAAGGAGGCCGCAAGCCAGCTCGCCTTATCGTATTCCCAGATTTGGGGGTTCGYGTGTGCGAGAAAATGGCYCTTTATGACGTGGTCTCCACCCTTCCTCAGGCCGTGATGGGCTCCTCATACGGGTTCCAGTACTCYCCTGGACAGCGGGTTGAGTTCCTGGTGAATGCCTGGAAAAARAAGAAAAACCCTATGGGCTTCGCATATGACACCCGCTGTTTTGACTCRACGGTCACCGAGAGTGACATCCGTGTTGAGGAGTCAATCTACCAATGTTGTGACTTGGCCCCCGAAGCCAGACAGGCCATAAGGTCGCTCACAGAGCGGCTTTAYATCGGGGGTCCCCTGACTAACTCAAAGGGRCAAAACTGCGGTTATCGCCGGTGCCGCGCGAGCGGTGTGCTGACGACCAGCTGCGGTAATACCCTCACATGTTACTTGAAGGCTTCTGCAGCCTGTCGAGCTGCCAAGCTCCAGGACTGCACGATGCTCGTGTGCGGAGACGACCTTGTCGTCATCTGTGAAAGCGCGGGAACCCAGGAGGACGCGGCGAGCCTACGAGTCTTCACGGAGGCTATGACTAGGTACTCTGCCCCCCCCGGGGACCCGCCCAAACCAGAATACGACTTGGAGTTGATAACATCATGCTCCTCCAATGTGTCRGTCGCGCACGATGCATCTGGCAAAAGGGTGTAYTACCTCACCCGTGACCCCACCACCCCACTTGCACGAGCAGCGTGGGAGACAGCCAGACACACTCCAGTCAACTCCTGGCTAGGCAACATCATCATGTATGCGCCCACTCTATGGGCAAGGATGATCCTGATGACTCACTTCTTTTCCATCCTTCTAGCTCAGGAACAACTTGAAAAGGCTCTGGACTGTCAGATCTACGGAGCCTGTTACTCCATTGAGCCACTTGACCTACCTCAGATCATTCAGCGACTCCATGGNNNNNNNNNNNNNNNNNNNNNNNNNNNNNNNNNNNNNNNNNNNNNNNNNNNNNNNNNNNNNNNNNNNNNNNNNNNNNNNNNNNNNNNNNNNNNNNNNNNNNNNNNNNNNNNNNNNNNNNNNNNNNNNNNNNNNNNNNNNNNNNNNNNNNNNNNNNNNNNNNNNNNNNNNNNNNNNNNNNNNNNNNNNNNNNNNNNNNNNNNNNNNNNNNNNNNNNNNNNNNNNNNNNNNNNNNNNNNNNNNNNNNNNNNNNNNNNNNNNNNNNNNNNNNNNNNNNNNNNNNNNNNNNNNNNNNNNNNNNNNNNNNNNNNNNNNNNNNNNNNNNNNNNNNNNNNNNNNNNNNNNNNNNNNNNNNNNNNNNNNNNNNNNNNN

>HM106927

NNNATGTCCTACACATGGACRGGCGCCCTGATCACGCCATGCTCCGCGGAGGAAAGCAAGCTGCCCATCAACGCGTTGAGCAACTCTTTGCTGCGTCACCAYAATATGGTCTATGCCACAACRTCTCGCAGCGCAAGCCAGCGGCAGAAGAAGGTCACCTTTGACAGACTGCAGGTCCTGGACGACCATTACCGGGACGTGCTCAAGGAGATGAAGGCGAAGGCGTCCACRGTTAAGGCYAAACTTCTATCCGTAGAAGAAGCCTGCATGCTGACGCCCCCACACTCRGCCARATCCAAATTTGGCTAYGGGGCAAAGGACGTCCGGAACCTATCCAGTAAGGCCATTAACCACATCCGCTCCGTGTGGAAGGASTTGCTGGAAGACACTGAGACACCAATTGACACCACCATCATGGCAAAAAATGAGGTTTTCTGCGTCCAACCAGAGAAAGGAGGCCGCAAGCCAGCTCGCCTTATCGTATTCCCAGACTTGGGGGTTCGTGTGTGCGAGAAAATGGCCCTTTATGACGTGGTCTCCACCCTTCCTCAGGCCGTGATGGGCTCCTCATACGGGTTYCAGTACTCTCCYGGACAGCGGGTCGAGTTCCTGGTGAATGCCTGGAAAAAAAAGAAGAACCYTATGGGCTTCGCATATGACACCCGCTGTTTTGACTCAACGGTCACCGAGAGTGACATCCGTGTTGAGGAGTCAATYTACCAATGTTGTGACTTGGCCCCCGAAGCCAGACAGGCCATAAGGTCGCTCACAGAGCGGCTTTACATCGGGGGWCCCCTGACTAAYTCAAAAGGGCAGAACTGCGGTTATCGCCGGTGCCGCGCGAGCGGTGTGCTGACGACCAGCTKCGGTWATACCCTCACATGTTACTTGAAGGCCTCTGCAGCCTGTCGAGCTKCCAAGCTCCMRGACTGCACGATGCTCGTGTGCGGAGACGACCTTGTCGTTATCTGTGARAGCGCGGGAACCCAGGAGGACGCGGCGAGCCTACGANNNNNNNNNNNNNNNNNNNNNNNNNNNNNNNNNNNNNNNNNNNNNNNNNNNNNNNNNNNNNNNNNNNNNNNNNNNNNNNNNNNNNNNNNNNNNNNNNNNNNNNNNNNNNNNNNNNNNNNNNNNNNNNNNNNNNNNNNNNNNNNNNNNNNNNNNNNNNNNNNNNNNNNNNNNNNNNNNNNNNNNNNNNNNNNNNNNNNNNNNNNNNNNNNNNNNNNNNNNNNNNNNNNNNNNNNNNNNNNNNNNNNNNNNNNNNNNNNNNNNNNNNNNNNNNNNNNNNNNNNNNNNNNNNNNNNNNNNNNNNNNNNNNNNNNNNNNNNNNNNNNNNNNNNNNNNNNNNNNNNNNNNNNNNNNNNNNNNNNNNNNNNNNNNNNNNNNNNNNNNNNNNNNNNNNNNNNNNNNNNNNNNNNNNNNNNNNNNNNNNNNNNNNNNNNNNNNNNNNNNNNNNNNNNNNNNNNNNNNNNNNNNNNNNNNNNNNNNNNNNNNNNNNNNNNNNNNNNNNNNNNNNNNNNNNNNNNNNNNNNNNNNNNNNNNNNNNNNNNNNNNNNNNNNNNNNNNNNNNNNNNNNNNNNNNNNNNNNNNNNNNNNNNNNNNNNNNNNNNNNNNNNNNNNNNNNNNNNNNNNNNNNNNNNNNNNNNNNNNNNNNNNNNNNNNNNNNNNNNNNNNNNNNNNNNNNNNNNNNNNNNNNNNNNNNNNNNNNNNNNNNNNNNNNNNNNNNNNNNNNNNNNNNNNNNNNNNNNNNNNNNNNNNNNNNNNNNNNNNNNN

>HM106928

NNNNNNNNNNNNNNNNNNNNNNNNNNNNNNNNNNNNNNNNNNNNNNNNNNNNNNNNNNNNNNNNNNNNNNNNNNNNNNNNCAACTCTTTGCTGCGTCACCACAACATGGTCTATGCCACAACATCTCGCAGCGCAAGCCAGCGGCARAAGAAGGTCACCTTTGACAGACTGCAGGTCCTGGACGACCAYTACCGGGACGTGCTYAAGGAGATGAAGGCGAAGGCGTCCACAGTTAAGGCTAAACTTCTATCCGTAGAAGAAGCCTGCATGCTGACGCCCCCACACTCGGCCAAATCYAAATTTGGCTATGGGGCAAAGGACGTCCGGAGCCTATCCAGCAAGGCYATTASCCACATCMACTCCGTGTGGAAGGACTTGYTGGAAGACACTGAGACACCAATTGACACCACCGTCATGGCAAAAAATGAGGTYTTCTGCGTCGAACCAGAGAAAGGAGGCCGCAAGCCAGCTCGCCTTATCGTATTCCCAGAYTTGGGGGTTCGTGTGTGCGAGAAAATGGCCCTTTATGACGTRGTCTCCACCCTTCCTCAGGCCGTGATGGGCTCCTCATACGGGTTCCAGTACTCTCCTGGACAGCGGGTCGAGTTCCTGGTGAATGCCTGGAAAAARAAGAAAAACCCYATGGGCTTCGCATATGACACCCGCTGTTTYGACTCAACGGTCACCGAGAAYGACATCCGTGTTGAGGAGTCAATCTACCAATGTTGTGACTTGGCCCCCGAAGCCAGACAGGCYATAAGGTCGCTCACAGAGCGGCTTTATATCGGGGGYCCCCTGACTAACTCAAAAGGGCAGAACTGCGGYTATCGCCGGTGCCGCGCRAGCGGTGTGCTGACGACCAGCTGCGGTAATACCCTCACATGTTACTTGAAGGCCTCTGCAGCCTGTCGAGCTGCCAAGCTCCAGGACTGCACGATGCTCGTGTGCGGAGACGACCTTGTCGTTATCTGTGAAAGCGCGGGAACTCAGGAGGACGCGGCAAGCCTACGAGTCTTCACGGAGGCTATGACTAGGTACTCTGCCCCCCCCGGGGACCCGCCCAAACCAGAATACGACTTGGAGYTGATAACATCATGCTCCTCCAAYGTGTCGGTCGCGCACGAYGCATCTGGCAAAAGGGTRTACTACCTCACCCGTGACCCCACCACCCCACTTGCACGGGCTGCGTGGGAGACAGCTAGACACACTCCAGTCAACTCCTGGCTAGGCAACATYATCATGTATGCGCCCACCTTATGGGCAAGGATGATCYTGATGACTCACTTCTTCTCCATCCTTCTAGCTCAGGAACAACTTGAAAAGGCTCTAGATTGTCAGATCTACGGGGCCTGTTACTCCATTGAGCCACTTGACCTACCTCAAATCATTCAGCGACTCCATGGNNNNNNNNNNNNNNNNNNNNNNNNNNNNNNNNNNNNNNNNNNNNNNNNNNNNNNNNNNNNNNNNNNNNNNNNNNNNNNNNNNNNNNNNNNNNNNNNNNNNNNNNNNNNNNNNNNNNNNNNNNNNNNNNNNNNNNNNNNNNNNNNNNNNNNNNNNNNNNNNNNNNNNNNNNNNNNNNNNNNNNNNNNNNNNNNNNNNNNNNNNNNNNNNNNNNNNNNNNNNNNNNNNNNNNNNNNNNNNNNNNNNNNNNNNNNNNNNNNNNNNNNNNNNNNNNNNNNNNNNNNNNNNNNNNNNNNNNNNNNNNNNNNNNNNNNNNNNNNNNNNNNNNNNNNNNNNNNNNNNNNNNNNNNNNNNNNNNNNNNNNNNNNNNNN

>HM106929

NNNATGTCCTACACATGGACAGGCGCCCTGATTACGCCATGCTCCGCGGAGGAAAGCAAGCTGCCCATCAACGCGTTGAGCAACTCTTTGCTGCGTCACCACAACATGGTCTATGCCACAACATCTCGCAGCGCAAGCCAACGGCAGAAGAAGGTCACCTTTGACAGRCTGCAGGTCCTGGACGACCATTACCGGGACGTGCTYAAGGAGATGAAGGCGAAGGCGTCCTYGGTTAAGGCTAARCTTCTATCCGTAGAAGAAGCCTGCATGCTGACGCCCCCACACTCGGCCAAATCCAAGTTTGGCTATGGGGCRAAGGACGTCCGGAACCTATCCMGCAAGGCCATTAACCACATCMRCTCCGTGTGGAAGGACTTGCTGGAAGACACYGAGACACCAATTGACACCACCATCATGGCAAAGAATGAAGTTTTCTGCGTCCAACCAGAGAAAGGAGGCCGCAAGCCAGCTCGCCTTATTGTATTCCCAGACTTGGGGGTTCGTGTGTGCGAGAAGATGGCCCTTTATGAYGTGGTCTCCACCCTTCCTCAGGCCGTGATGGGCTCCTCATACGGRTTCCAGTACTCTCCTGGACAGCGGGTCGAGTTCCTGGTGAATGCCTGGAAAAARAAGAARAACCCTATGGGYTTCGCATATGACACCCGCTGTTTTGACTCAACRGTCACCGAGAGTGACATCCGTGTYGAGGAGTCAATCTAYCAATGTTGTGACTTGGCCCCCGAAGCCAGACAGGCCATAAGGTCGCTCACRGARCGGCTTTATATCGGGGGYCCCCTGACTAACTCAAAAGGGCAGAACTGCGGTTATCGCCGGTGCCGCGCGAGCGGRGTGCTGACGACCAGCTGCGGTAATACCCTCACATGTTACTTRAAGGCCKCTGCAGCCTGTCGAGCTGCCAAGCTCCAGGACTGCACAATGCTCGTGTGCGGRGACGACCTTGTCGTRATCTGTGAAAGCGCGGGAACCCAGGAGGAYGCGGCGAGCCTACGAGTCTTCACGGAGGCTATGACTAGGTACTCTGCCCCCCCCGGGGACCCGCCCAAACCGGAATACGACTTGGAGTTGATAACATCATGCTCCTCCAATGTGTCGGTCGCRCACGATGCATCTGGCAAAAGGGTATACTACCTCACCCGTGACCCCACCACCCCACTTGCACGGGCTGCGTGGGAGACAGCTAGACACACTCCAGTYAACTCCTGGCTAGGCAACATCATCATGTATGCGCCCACYTTATGGGCRAGGATGATCCTGATGACTCACTTCTTCTCCATCCTTCTAGCTCAGGAACAACTYGAAAAGGCNNNNNNNNNNNNNNNNNNNNNNNNNNNNNNNNNNNNNNNNNNNNNNNNNNNNNNNNNNNNNNNNNNNNNNNNNNNNNNNNNNNNNNNNNNNNNNNNNNNNNNNNNNNNNNNNNNNNNNNNNNNNNNNNNNNNNNNNNNNNNNNNNNNNNNNNNNNNNNNNNNNNNNNNNNNNNNNNNNNNNNNNNNNNNNNNNNNNNNNNNNNNNNNNNNNNNNNNNNNNNNNNNNNNNNNNNNNNNNNNNNNNNNNNNNNNNNNNNNNNNNNNNNNNNNNNNNNNNNNNNNNNNNNNNNNNNNNNNNNNNNNNNNNNNNNNNNNNNNNNNNNNNNNNNNNNNNNNNNNNNNNNNNNNNNNNNNNNNNNNNNNNNNNNNNNNNNNNNNNNNNNNNNNNNNNNNNNNNNNNNNNNNNNNNNNNNNNNNNNNNNNNNNNNNNNNNNNNNNNNNNNNNNNN

>HM106930

NNNATGTCCTACACATGGACAGGCGCCCTGATCACGCCATGCTCCGCGGAGGAAAGCAAGCTGCCCATYAACGCGTTGAGCAACTCTTTGCTGCGTCACCACAACATGGTTTATGCCACAACATCTCGCAGCGCAAGCCAGCGGCAGAAGAAGGTCACCTTTGACAGACTGCAGGTCCTGGACGACCATTACCGGGACGTGCTCAAGGAGATGAAGGCGAAGGCGTCCACRGTYAAGGCCAAACTTCTATCCGTAGAAGAAGCCTGCATGCTGACGCCCCCACACTCGGCCAAATCCAAATTTGGCTAYGGGGCRAAGGACGTCCGGAACCTATCCAGCAAGGCCATTAACCACATCCACTCCGTGTGGAAGGACTTGCTGGAAGACACTGAGACACCAATTGACACCACCATCATGGCAAAAAATGAGGTTTTCTGYGTCCAACCAGAGAAAGGAGGCCGCAAGCCRGCTCGCCTTATCGTATTCCCAGACTTGGGGGTTCGTGTGTGCGAGAAAATGGCCCTTTAYGACGTGGTCTCCACCCTTCCYCAGGCCGTGATGGGCTCCTCATACGGGTTCCAGTACTCTCCYGGACAGCGGGTCGARTTCCTGGTGAATGCTTGGAAAAAAAAGAAGAACCCTATGGGCTTCGCATATGACACCCGCTGTTTTGACTCAACGGTCACSGAGAGYGACATCCGTGTTGAGGAGTCAATYTACCAATGTTGTGACTTRGCCCCCGAAGCCAGACAGGCTATAAGGTCGCTCACAGAGCGGCTTTATATCGGGGGYCCCCTGACTAACTCAAAAGGGCAGAACTGCGGTTATCGCCGGTGCCGCGCGAGCGGTGTGCTGACGACCAGCTGCGGTAAYACCCTYACATGCTACTTGAAGGCCTCTGCAGCCTGTCGAGCTGCYAAGCTCCAGGACTGCACRATGCTCGTGTGCGGAGACGACCTTGTCGTTATCTGTGAGAGCGCGGGAACCCAGGAGGACGCGGCGAGCCTACGAGTCTTCACGGAGGCTATGACTAGGTACTCTGCCCCCCCCGGGGACCCGCCCAAACCAGAATACGACTTGGAGTTGATAACATCATGCTCCTCCAATGTGTCGGTCGCGCACGATGCATCTGGCAAAAGGGTGTACTACCTCACCCGTGACCCCACCACCCCACTTGCACGGGCTGCGTGGGAGACAGCTAGACACACTCCAGTCAAYTCCTGGCTAGGCAAYATCATCATGTATGCGYCYACCTTATGGGCAAGGATGATYCTGATGACTCACTTCTTCTCCATCCTTCTAGCTCAGGAACAACTTGAAAAGGCTCTAGATTGTCAGATCTACGGGGCCTGTTACTCCATTGAACCACTTGACCTACCTCAGATCATTCAGCGACTCCATGGNNNNNNNNNNNNNNNNNNNNNNNNNNNNNNNNNNNNNNNNNNNNNNNNNNNNNNNNNNNNNNNNNNNNNNNNNNNNNNNNNNNNNNNNNNNNNNNNNNNNNNNNNNNNNNNNNNNNNNNNNNNNNNNNNNNNNNNNNNNNNNNNNNNNNNNNNNNNNNNNNNNNNNNNNNNNNNNNNNNNNNNNNNNNNNNNNNNNNNNNNNNNNNNNNNNNNNNNNNNNNNNNNNNNNNNNNNNNNNNNNNNNNNNNNNNNNNNNNNNNNNNNNNNNNNNNNNNNNNNNNNNNNNNNNNNNNNNNNNNNNNNNNNNNNNNNNNNNNNNNNNNNNNNNNNNNNNNNNNNNNNNNNNNNNNNNNNNNNNNNNNNNNNNNNN

>HM106931

TCGATGTCCTACACATGGACGGGCGCCCTGATCACGCCATGCTCCGCGGAGGAGAGCAAGCTGCCCATCAACGCGTTGAGCAACTCTTTGCTGCGTCACCACAACATGGTCTACGCCACAACATCTCGCAGCGCAAGCCAGCGGCAGAAGAAGGTTACCTTTGACAGACTGCAGGTCCTGGACGACCATTACCGGGACGTGCTCAAGGAGATAAAGGCGAAGGCGTCCACGGTTAAGGCTAGACTTCTATCCGTAGAAGAAGCCTGCAAGCTGACGCCCCCACACTCGGCCAGATCYAAATTTGGCTATGGGGCAAAGGACGTCCGGAACCTATCCAGCAAGGCCGTTAGCCACATTAGCTCCGTGTGGAAGGACTTGCTGGAAGACACTGAGACACCAATTGACACCACCATCATGGCAAAAAATGAGGTTTTCTGCGTCCAACCAGAGAAAGGAGGCCGCAAGCCAGCTCGCCTTATCGTATACCCAGATTTGGGGGTTCGTGTGTGCGAGAAAATGGCCCTATATGACGTGGTCTCCACCCTCCCTCAGGCCGTGATGGGCTCCTCATACGGGTTCCAGTACTCTCCTGGACAGCGGGTCGAGTTCCTGGTGAATGCCTGGAAAAAAAAGAAAAACCCTATGGGCTTCGCATATGACACCCGCTGTTTTGACTCAACRGTCACCGAGAGTGACATCCGTGTTGAGGAGTCAATCTACCAATGTTGTGACTTGGCCCCCGAAGCCAGACAGGCCATAAGGTCGCTCACAGAGCGGCTTTATATCGGGGGTCCCCTGACTAACTCAAAAGGGCAGAACTGCGGCTATCGCCGGTGCCGCGCAAGCGGTGTGCTGACGACCAGCTGCGGYAATACCCTCACATGTTACTTGAAGGCCTCTGCAGCCTGTCGAGCTGCCAAGCTCCAGGACTGCACGATGCTCGTGTGCGGAGACGACCTCGTCGTTATCTGTGAAAGCGCGGGAACCCAGGAGGACGCGGCGAGCCTACGAGTCTTCACGGAGGCTATGACTAGGTACTCTGCCCCCCCCGGGGACCCGCCCAAACCAGAATACGACTTGGAGCTGATAACATCATGCTCCTCCAATGTGTCGGTCGCGCACGATGCATCTGGCAAAAGGGTGTACTACCTCACCCGTGACCCCACCACCCCACTTGCACGGGCTGCGTGGGAGACAGCTAGACACACTCCAGTCAACTCCTGGCTAGGCAACATCATCATGTATGCGCCCACCTTATGGGCAAGGATGATTCTGATGACTCACTTCTTCTCCATCCTTCTAGCTCAGGAACAACTTGAAAAGGCTCTAGATTGCCAGATCTACGGGGCCTGTTACTCCATTGAACCACTTGACCTACCTCANNNNNNNNNNNNNNNNNNNNNNNNNNNNNNNNNNNNNNNNNNNNNNNNNNNNNNNNNNNNNNNNNNNNNNNNNNNNNNNNNNNNNNNNNNNNNNNNNNNNNNNNNNNNNNNNNNNNNNNNNNNNNNNNNNNNNNNNNNNNNNNNNNNNNNNNNNNNNNNNNNNNNNNNNNNNNNNNNNNNNNNNNNNNNNNNNNNNNNNNNNNNNNNNNNNNNNNNNNNNNNNNNNNNNNNNNNNNNNNNNNNNNNNNNNNNNNNNNNNNNNNNNNNNNNNNNNNNNNNNNNNNNNNNNNNNNNNNNNNNNNNNNNNNNNNNNNNNNNNNNNNNNNNNNNNNNNNNNNNNNNNNNNNNNNNNNNNNNNNNNNNNNNNNNNNNNNNNNNNNNNNNNNNNNNN
