# Supplementary material for: Effect of Immune Pressure on Hepatitis C Virus Evolution: Insights From a Single-Source Outbreak
Source: Hepatology. 2011 Feb;53(2):396–405. doi: 10.1002/hep.24076 (PMC3044208; doi:10.1002/hep.24076)
Supplement: Supplementary file 11 [file hep0053-0396-SD11.doc]

>HM106932

GCCTTAGAGAACTTGGTGGTCCTCAACGCGGCGTCCGTGGCCGGAACGCATGGCATYTTCTCTTTCCTTGTGTTCTTCTGTGCTGCCTGGTACATCAAGGGCAGGCTGGTCCCTGGGGCGGCATATGCTTTCTATGGCGTATGGCCGCTGCTCCTGCTTCTGCTGGCGTTGCCACCACGAGCATACGCC

>HM106933

GCCTTAGAGAACTTGGTGGTCCTCAATGCGGCGTCCGTGGCCGGAACGCATGGCATTCTCTCCTTCCTTGTGTTCTTCTGTGCTGCCTGGTACATCAAGGGCAGGCTGGTCCCTGGGGCGGCATATGCYTTTTATGGCGTATGGCCGCTGCTCCTGCTCCTGCTGGCGTTGCCACCACGRGCATACGCC

>HM106934

NNNNNNNNNNNNNNNNNNNNNNNNNNNGCGGCGTCCGTGGCCGGAACGCATGGCATTCTCCCTTTCCTTGCGTTTTTCTGTGCTGCCTGGTACATCAAGGGCAGGCTGGTCCCTGGGGCGGCATATGCTTTCTATGGCGTATGGCCGCTGCTCCTGCTCCTGCTGGCGTTACCACCACGAGCATACGCT

>HM106935

NNNNNNNNNNNNNNNNNNNNNNNNNNNNNNNNNNNNNNNNNNNNNNCGCATGGCATTCTCTCTTTCCTTGTGTTCTTTTGTGCTGCCTGGTACATTAAGGGTAGGCTGGTCCCTGGGGCGGCATATGCTTTCTATGGCGTRTGGCCGCTGCTCCTGCTCCTGCTGGCGTTGCCACCACGAGCATACGCC

>HM106936

GCCTTAGAGAACTTGGTGGTCCTCAATGCGGCGTCCGTCGCCGGAACGCATGGCATTYTCYCTTTCCTTGTGTTCTTCTGTGCTGCCTGGTACATCAAGGGCAGGCTGGTCCCTGGRGCGGCATATGCTTTCTATGGCRTATGGCCGCTGCTCCTGCTCCTGCTGGCGTTGCCACCACGAGCRTACGCC

>HM106937

GCCTTAGAGAACTTGGTGRTCCTCAATGCGGCGTCCGTGGCCGGAACGCATGGCATTCTCTCTTTCCTWGTGTTCTTCTGTGCTGCCTGGTACATCAAGGGCAGGCTGGTCCCTGGGGCGGCATATGCYTTCTATGGCGTATGGCCGCTGCTCCTGCTCCTGCTGGCGYTGCCACCACGAGCATACGCC

>HM106938

NNNNNNNNNNNCCTGGTGGTCCTCAATGCGGCGTCCGTGGCCGGAACGCATGGCATTCTCTCCTTTCTTGTGTTCTTCTGTGCTGCCTGGTACATCAAGGGCAGGCTGGTTCCTGGGGCGGCATATGCTTTCTATGGCGTATGGCCGCTGCTCCTGCTCCTGTTGGCGTTACCACCACGAGCATACGCC

>HM106939

GCCTTAGAGAACTTGGTGGTCCTCAATGCGGCGTCCGTGGCCGGAACGCAYGGCATTCTCYCTTTYCTWGTGTTCTTCTGTGCTGCCTGGTACATCAAGGGCAGGCTGGTCCCTGGRGCGGCATATGCTATCTATGGYGTATGGCCGCTGCTCCTGCTCCTGCTGGCGTTGCCACCACGAGCATACGCC

>HM106940

GCCTTAGAGAACTTGGTGGTCCTCAKAGCGGCGTCCGTGGCCGGAACGCATGGCATTCTCTCTTTCCTTGTGTTCTTCTGTGCAGCCTGGTACATCAAGGGCAGGCTGGTCCCCGGGGCGGCATATGCTCTCTATGGCGTGTGGCCGCTGCTCCTGCTCCTGCTGGCGTTGCCACCACGAGCATACGCC

>HM106941

GCCTTAGAGAACTTGGTNNNNNNNNNNGCGGCGTCCGTGGCCGGAACGCATGGCATTCTCTCTTTCCTTGTGTTCTTCTGTGCTGCCTGGTACATCAAGGGTAGGCTGGTCCCTGGGGTGGCATATGCTTTYTATRGCGTATGGCCGCTGCTTCTGCTCCTGCTGGCGTTGCCACCACGAGCATACGCC

>HM106942

GCCCTAGAGAACTTGGTGGTCCTCAATGCGGCGTCCGTAGCCGGAACGCATGGCATTCTCTCYTTCTTTGTGTTCTTCTGTGCTGCCTGGTACATCAAGGGCAGGCTGGTCCCTGGGGCGGCATATGCACTCTATGGCGTATGGCCGCTGCTCCTGCTCCTGCTGGCGTTGCCACCACGAGCATACGCC

>HM106943

GCCTTAGAGAACTTGGTGGTCCTCAATGCGGCGTCCGTGGCCGGAACGCATGGCATTCTYTCTTTCCTTGTGTTCTTCTGTGCTGCYTGGTACATCAAGGGTAGGCTGGTCCCTGGGGCGGCATATGCTCTCTATGGCGTATGGCCGCTGCTCCTGCTCCTGCTGGCGTTACCACCACGAGCRTACGCC

>HM106944

GCCTTAGAGAACTTGGTGGTCCTYAATGCGGCGTCCGTGGCCGGAACGCAYGGCAYTCTCYCYTTYCTTGTGTTCTTCTGTGCTGCCTGGTACATCAAGGGCAGGCTGGTCCCTGGGGCGGYATATGCTCTCTATGGCGTATGGCCGCTRCTCCTGCTCYTGCTGGCGYTGCCACCACGRGCATACGCC

>HM106945

GCCTTAGAGAACTTGGTGGTCCTCAATGCGGCGTCCGTGGCCGGAACGCATGGCATTCTCTCCTTCCTTGTGTTCTTCTGTGCTGCCTGGTACATCAAGGGCAGGCTGGTCCCCGGGGCGGYATAYGCTGTCTATGGCGTATGGCCACTGCTCCTGCTCCTGCTGGCGTTGCCACCACGAGCATACGCC

>HM106946

GCCTTAGAGAACTTGGTGRTCCTCAATGCGGCGTCCGTGGCCGGAACGCATGGCATTCTCTCTTTCCTTGTGTTCTTCTGTGCTGCCTGGTACATCAAGGGCAGGCTGGTCCCTGGGGCGGCATATGCTTTCTATGGCRTATGGCCGYTGCTCCTGCTCCTGCTGGCGTTGCCACCACGAGCATACGCC

>HM106947

GCCTTAGAGAACNNNNNNNNNNTCAATGCGGCGTCCGTGGCTGGAACGCATGGCATTATTTCTTTCCTTGTGTTCTTCTGTGCTGCCTGGTACATCAAGGGCAGGCTGGTCCCTGGGGCGGCATATGCTTTCTATGGCGTATGGCCGCTGCTTCTGCTCCTGCTGGCGTTGCCACCACGAGCATACGCC

>HM106948

GCCTTAGAGAACTTGGTGGTCCTCAATGCGGCGTCCGTGGCCGGARCGCATGGCATTCTCTCTTTCCTTGTGTTCTTCTGTGCTGCCTGGTACATCAAGGGCAGGCTGGTCCCTGGGGCGGCATATGCTCTCTATGGCGTATGGCCGCTGCTCCTGCTCCTGCTGGCGTTGCCACCGCGAGCGTACGCC

>HM106949

GCCTTAGAGAACTTGGTGGTCCTCAATGCGGCGTCCGTGGCCGGAACGCATGGCATTCTCTCTTTCCTTGTGTTCTTCTGTGCTRCCTGGTACATCAAGGGTAGGCTGGTCCCTGGGGCGGCATATGCTCTCTATGGTGTATGGCCGCTGCTCCTGCTCCTGCTGGCGTTACCACCYCGAGCATACGCC

>HM106950

NNNNNNNNNNNNNNNNNNNTCCTCAATGCGGCGTCCGTGGCCGGAACGCATGGCATTCTTCCTTTCCTTGTGTTCTTCTGTGCTGCCTGGTACATTAAGGGCAGGTTGGTCCCTGGGGCGGCATATGCTCTCTATGGCGTATGGCCGCTGCTCCTGCTCCTGCTGGCGTTGCCACCACGAGCTTACGCC

>HM106951

GCCTTAGAGAACTTGGTGGTCCTCAATGCGGCGTCCGTGGCCGGAACGCATGGYATYCTCYCTTTCCTTGTGTTCTTCTGTGCTGCCTGGTACATCAAGGGCAGGTTGGTYCCTGGGGCGGCRTAYGCTATCTATGGCATATGGCCGMTGCTCCTGCTCCTGCTGGCRTYRCCACCACGAGCATAYGCC

>HM106952

GCCTTAGAGAACTTGGTGNNNNNNNNNNNNNNNNNNNNNNNNNNNNNNNNNNNNNNNNNNNNNNNNNNNNNNNNNNNNNNNNNNNNNNNNNNNNNNNNNNNNNGGCTGGTCCCTGGGGCGGCATATGCTATCTACGGCGTATGGCCGCTGCTCCTGCTCCTGCTGGCGTTGCCACCACGAGCATACGCC

>HM106953

NNNNNNNNNNNNNTGGTGGTCCTTAATGCGGCGTCCGTGGCTGGAACGCATGGCATTCTCTCTTTCCTTGTGTTCTTCTGTGCTGCCTGGTACATCAAGGGCAGGCTGGTCCCTGRGGCGGCATATGCTTTCTACAGCGTATGGCCGCTGCTCCTGCTYCTGCTGGCGTTGCCACCACGAGCATACGCC

>HM106954

NNNNNNNNNNNNNNNNNNNNNNTCAATGCGGCATCCGTGGCCGGAACGCATGGCATTTTCTCTTTCCTTGTGTTCTTCTGTGCTGCCTGGTACATCAAGGGCAGGCTGGTCCCTGGGGCGRCATATGCTTTCTAYGGCGTATGGCCGCTGCTCCTGCTCCTGCTGGCTTTGCCACCACGAGCATACGCC

>HM106955

GCTTTGGAGAACTTGGTGGTCCTCAATGCGGCGTCCGTGGCCGGAACGCATGGTATTCTCTCTTTCCTTGTGTTCTTCTGTGCTRMCTGGTACATCAAGGGCAGGCTGGTCCCTGGGGCGGCATATGCTTTCTTTGGCGTATGGCCGCTGCTCCTGCTCCTGCTGGCGTTGCCACCACGAGCGTACGCC

>HM106956

GCCTTAGAGAACTTGGTGGTCCTCAATGCGGCGTCCGTGGCCGGAACGCATGGCATTCTCYCYTTCCTTGTGTTCTYCTGTGCTGCCTGGTACATCAAGGGCAGGCTGGTCCCTGGGGCGGCATATGCTTTCTATGGCGTATGGCCGCTGCTCCTGCTCCTGYTGGCGTTGCCACCACGAGCATACGCC

>HM106957

NNNNNNNNNNACTTGGTGGTCCTCAATGCGGCGTCCGTGGCCGGAACGCATAGCATCTTCTCTTTCCTTGTGTTCTTCTGTGCTGCCTGGTACATTAAGGGCAGGCTGGTCCCTGGGGCGGCATATGCTTTCTTTGGCGTATGGCCGCTGCTCCTGCTCCTACTGGCGTTGCCACCACGAGCRTACGCC

>HM106958

NNNNNNNNNNNNNTGGTGGTCCTCAACGCGGCGTCCGTGGCCGGAACGCATGGCATCTTCCCGTTCCTTGTGTTCTTCTGTGCTGCCTGGTACATCAAGGGCAGGCTGGTCCCTGGGGCGGCATATGCTCTCTATGGCGTATGGCCGCTGCTCCTGCTCCTGCTGGCGTTGCCACCACGAGCATACGCC

>HM106959

GCCTTAGAGAACNNNNNNNNNNNNNNNNNGGCGTCCGTGGCCGGAACGCATGGCATTCTCTCCTTCCTTGTGTTCTTCTGTGCTGCCTGGTACATCAAGGGCAGGCTGGTCCCTGGGGCGGCATAYGCTTTCTATGGCGTATGGCCGCTGCTCCTGCTCCTGCTGGCGTTGCCACCACGAGCATACGCC

>HM106960

NNNNNNNNNNNNNNNGTGGTCCTCAATGCGGCGTCCGTGGCCGGAATGCATGGTATTTTCYCTTTCTTTGTGTTCTTCTGTGCCGCCTGGTACATCAAGGGCAGGTTGGTYCCTGGGGCGGCATATGCTTTCTATGGCGTATGGCCGYTGYTCCTGCTCCTGCTGGCGTTGCCACCACGAGCATACGCC

>HM106961

GCCTTAGAGAACTTGGTGNNNNNNNNNNNNNNNNNNNNNNNNNNNNNNNNNNNNNNNNNNNNNNNNNNNNNNNNCTTTTGTGCTGCCTGGTACATCAAGGGCAGGCTGGTCCCTGGGGCGGCGTATGCTTTTTATGGCGTATGGCCGCTGCTCCTGCTCCTGCTGGCGTTGCCACCACGAGCATACGCC

>HM106962

GCCTTAGAGAACTTGGTGGTCCTCAATGCAGCGTCCGTGGCCGGAACGCATGGCATTCTCCCCTWCCTTGTGTTCTTCTGTGCTGCCTGGTACATCAAGGGCAGGCTGGTCCCTGGGGCGGCATATGCTTTCTATGGCGTATGGCCGCTGCTCCTGCTCCTGCTGGCGTTGCCACCACGCGCATACGCC

>HM106963

NNNNNNNNNNNNNNNNTGGTCCTCAATGCGGCGTCTGTGGCCGGAACGCATGGCTTTCTCTCCTTCCTTGTGTTCTTCTGTGCTGCCTGGTACATCAAGGGCAGGCTGGTCCCTGGGGCGGCATATGCTTTTTATAGCGTATGGCCGCTGCTCCTGCTCCTGCTGGCGTTGCCACCACGAGCATATGCC

>HM106964

GCCTTAGAGAACTTGGTNNNNNNNNNNNNNNNNNNNNNNNNNNNNNNNNNTGGCATTCTCTCYTTCCTTRTGTTCTTCTGTGCTGCCTGGTACATCAAGGGCAGGCTGGTCCCYGGGGCGGCATACGCTTTCTATGGCGTATGGCCGCTGCTCCTGCTCCTGCTGGCGTTACCACCACGAGCATACGCC

>HM106965

GCCTTAGAGAACTTGGTGGTCCTCAATGCGNNNNNNNNNNNNNNNNNNNNNNNNNNNNNNNNNNNNNNNNNNNNNNNNNNNNNNNNNNNNNNNNTCAAGGGCAGGCTGGTCCCTGGGGCGGCATATGCTTTCTATGGCGTATGGCCGCTRCTCCTGCTTCTGCTGGCGTTGCCACCRCGAGCATACGCC

>HM106966

NNNNNNNNNNNNNTGGTGGTCCTCAATGCGGCGTCCGTGGCCGGAACGCATGGCATTCTCTCCTTCCTTGTGTTCTTCTGTGCTGCCTGGTACATCAAGGGCAGGCTGGTCCCTGGGGCGGCATATGCTTTCTATGGCGTATGGCCGCTGCTTCTGCTCCTGCTGGCGTTGCCACCACGAGCATACGCC

>HM106967

NNNNNNNNNNNNNNNNNNNNNNNNNNNNCGGCGTCCGTGGCCGGAACGCATGGCATTCTMTCCTTCCTTGTGTTCTTCTGTGCTGCCTGGTACATCAAAGGCAGGCTGGTCCCTGGGGCGGCGTATGCTTTCTATGGCGTATGGCCGCTGCTCCTGCTCCTGCTGGCGTTGCCACCACGGGCATATGCC

>HM106968

GCCCTAGAGAANNNNNNNNNNNNNNNNNNNNNNNCCGTGGCCGGGAAGCATGGCATTCTCTCTTTCCTTGTGTTCTTCTGCGCTGCCTGGTACATCAAGGGCAGGCTGGTCCCTGGGGCGGCATATGCTTTCTATGGCGTATGGCCGCTGCTCCTGCTCCTACTGGCRTTGCCACCACGAGCRTACGCC

>HM106969

GCCTTAGAGANNNNNNNNNNNNNNNNNNNNNNNNNNNNGGCCGGAACGCATGGCATTCTCCCTTTTCTTGTGTTCTTCTGTGCTGCCTGGTACATCAAGGGCAGGCTGGTCCCTGGGGCGGCATATGCTTTCTATGGCGTATGGCCGCTGCTCCTGCTCCTGCTGGCGTTGCCACCACGAGCGTACGCC

>HM106970

GCNNNNNNNNNNNNNNNNNNNNNNNNNNNNNNNNNNNNNNNNNNNNNNNNNNNNNNNNNNNNNNNNNNNNNNTTCTTCTGTGCTGCCTGGTACATCAAGGGCAGGCTGGCCCCTGGGATGGCATATGCCTTCTACGGCGTATGGCCGCTGCTCCTGCTCCTGCTGGCGTTGCCACCACGAGCATACGCC

>HM106971

NNNNNNNNNNNNNNGGTGGTCCTCAATGCGGCGTCCGTGGCCGGAACGCACGGCATTCTCTCTTTCCTTGTGTTTTTCTGTGCTGCCTGGTACATCAAGGGCAGGCTGGTCCCTGGGGCGGCATATGCCTTCTATGGCGTATGGCCGCTGCTTCTGCTCCTGCTGGCGTTACCACCACGAGCATACGCC

>HM106972

GCCTTAGAGAACTTGGTGGTCCTCAATGCGGCGTCCGTGGCCGGAACGCRTGGCATTCTYYCTTTCCTTGTGTTCTTCTGTGCTRCCTGGTACATCAAGGGCAGGCTGGTCCCTGGGGCGGCATATGCCTTCTATGGCGTATGGCCGCTGCTCCTGCTCCTGTTGGCGTTGCCACCACGAGCATACGCC

>HM106973

GCCTTGGAGAACTTGGTGGTCCTCAATGCGGCGTCCGTGGCCGGAACGCATGGCATTCTCTCCTTCCTTGTGTTCTTCTGTGCTGCCTGGTACATCAAGGGCAGGCTGGTCCCTGGRGCGGCATATGCCTTCTATGGCGTATGGCCGCTGCTCCTGCTCCTRTTGGCGTTACCACCRCGAGCRTACGCC

>HM106974

NNNNNNNNNNNNNNNNNGGTCCTCAATGCGGCGTCCGTGGCCGGAACGCATGGCATCCTCTCTTTCCTSGTGTTCTTCTGTGCTGCCTGGTACATCAAGGGCAGGCTGGTCCCYGGGGCAGCATACGCCTTCTATGGCGTRTGGCCGCTGCTCTTGCTCCTGCTGGCGYTGCCACCACGAGCRTACGCT

>HM106975

GCCTTAGAGAACNNNNNNNNNNNNNNNNNNNNNNCCGTGGCCGGAACGCATGGCATTCTCTCTTTCCTTGTGTTCTTCTGTGCTGCCTGGTACATCAAGGGCAGGCTGGTCCCTGGGGCGGCATATGCCTTCTATGGCGTATGGCCGCTACTCCTGCTCCTGCTGGCGTTGCCACCACGAGCATACGCC

>HM106976

GCCTTAGAGAACTTGGTGGTNNNNNNNNNNNNNNNNNNNNNNNNNNNNNNTGGCATTCTCTCTTTCCTTGTGTTCTTCTGTGCTGCCTGGTACATCAAGGGCAGGCTGGTCCCTGGGGCGGCATATGCTTTCTATGGCGTGTGGCCGCTGCTCCTGCTCCTGCTGGCATTGCCACCACGAGCATACGCC

>HM106977

GCCTTAGAGAACTTGGTGGTCCTCAATGCGGCKTCCGTGGCCGGAACGCATGGCATTCTCTCCTTCCTTGTGTTCTTCTGTGCTGCCTGGTACATCAAGGGCAGGCTGGTCCCCGGGGCGGCATATGCTTTCTATGGCGTATGGCCGCTGCTCCTGCTCCTGCTGGCRTTGCCACCGCGAGCATACGCC

>HM106978

GCCTTAGAGAACNNNNNNNNNNNNNNNNNNNNNNNNNNNNNNNNNNNNNNNNNNNNTCTCTCTTTCCTAGTGTTCTTCTGTGCTGCCTGGTACATCAAGGGCAGGCTGGTCCCTGGGGCGGCATATGCTTTTTATGGCGTATGGCCGCTGCTCCTACTCCTGCTGGCGTTGCCACCTCGAGCGTACGCC

>HM106979

GCCTTRGAGAACTTGGTGGTCCTCAATGCGGCGTCYGTGGCCGGAACGCATGGCATYYTCTCYTTCCTTGTGTTCTTCTGTGCTGCCTGGTACATCAAGGGCAGGCTGGTCCCTGGGGCGGCATACGCTTTCTAYGGCGTATGGCCGCTGCTCCTGCTCCTGCTGGCGYTRCCACCACGAGCATACGCC

>HM106980

GCCTTAGAGAACTTGGTGGTCCTCAATGCGGCTTCCGTGGCCGGAATGCATGGCATTTTCTCCTTCCTTGTRTTCTTCTGTGCTGCCTGGTACATCAAGGGCAGGCTGGTCCCTGGGGCGGCATATGCTTTCTATGGCGTATGGCCGCTGCTCCTGCTCCTGCTGGCRTTGCCACCACGAGCATACGCC

>HM106981

GCCTTAGAGAACTTGGTGGTCCTCAATGCGGCGTCCGTGGCCGGAACGCATGGCATTCTCTCTTTCCTAGTGTTCTTCTGTGCTGCCTGGTACATCAAGGGCAGGCTGGTCCCTGGGGCGGCATATGCTTTTTATGGCGTATGGCCGCTACTCCTACTCCTGCTGGCGTTACCACCTCGAGCGTACGCC
